# Unintentional injuries in Mexico, 1990–2017: findings from the Global Burden of Disease Study 2017

**DOI:** 10.1136/injuryprev-2019-043532

**Published:** 2020-04-01

**Authors:** Martha Híjar, Ricardo Pérez-Núñez, Elisa Hidalgo-Solórzano, Bernardo Hernández Prado, Rosario Valdez-Santiago, Erin B Hamilton, Spencer L James, Gregory J Bertolacci, Matthew Cunningham, Zachary V Dingels, Jack T Fox, Zichen Liu, Nicholas L S Roberts, Dillon O Sylte, Marcela Agudelo-Botero, Guilherme Borges, Lucero Cahuana-Hurtado, Ismael R Campos-Nonato, Rosario Cárdenas, Claudio Alberto Dávila-Cervantes, Edgar Denova-Gutiérrez, Daniel Diaz, Van C Lansingh, Gabriel Martinez, Pablo A Montero-Zamora, Edson Serván-Mori, Rafael Lozano

**Affiliations:** 1 Research Coordination, AC Environments Foundation, Cuernavaca, Mexico; 2 CISS, National Institute of Public Health, Cuernavaca, Mexico; 3 Center for Health Systems Research, National Institute of Public Health, Cuernavaca, Mexico; 4 Institute for Health Metrics and Evaluation, University of Washington, Seattle, WA, USA; 5 Department of Health Metrics Sciences, School of Medicine, University of Washington, Seattle, WA, USA; 6 School of Medicine, Center for Politics, Population and Health Research, National Autonomous University of Mexico, Mexico City, Mexico; 7 Department of Epidemiology and Psychosocial Research, Ramón de la Fuente Muñiz National Institute of Psychiatry, Mexico City, Mexico; 8 National Institute of Public Health, Cuernavaca, Mexico; 9 Department of Population and Health, Metropolitan Autonomous University, Mexico City, Mexico; 10 Population and Development, Facultad Latinoamericana de Ciencias Sociales Mexico, Mexico City, Mexico; 11 Center for Nutrition and Health Research, National Institute of Public Health, Cuernavaca, Mexico; 12 Center of Complexity Sciences, National Autonomous University of Mexico, Mexico City, Mexico; 13 Facultad de Medicina Veterinaria y Zootecnia, Autonomous University of Sinaloa, Culiacan Rosales, Mexico; 14 HelpMeSee, New York, NY, USA; 15 International Relations, Mexican Institute of Ophthalmology, Queretaro, Mexico; 16 Department of Economics, Autonomous Technology Institute of Mexico, Mexico City, Mexico; 17 Department of Public Health Sciences, University of Miami, Miami, FL, USA

**Keywords:** epidemiology, descriptive epidemiology, burden of disease, global

## Abstract

**Background:**

To date, the burden of injury in Mexico has not been comprehensively assessed using recent advances in population health research, including those in the Global Burden of Disease Study 2017 (GBD 2017).

**Methods:**

We used GBD 2017 for burden of unintentional injury estimates, including transport injuries, for Mexico and each state in Mexico from 1990 to 2017. We examined subnational variation, age patterns, sex differences and time trends for all injury burden metrics.

**Results:**

Unintentional injury deaths in Mexico decreased from 45 363 deaths (44 662 to 46 038) in 1990 to 42 702 (41 439 to 43 745) in 2017, while age-standardised mortality rates decreased from 65.2 (64.4 to 66.1) in 1990 to 35.1 (34.1 to 36.0) per 100 000 in 2017. In terms of non-fatal outcomes, there were 3 120 211 (2 879 993 to 3 377 945) new injury cases in 1990, which increased to 5 234 214 (4 812 615 to 5 701 669) new cases of injury in 2017. We estimated 2 761 957 (2 676 267 to 2 859 777) disability-adjusted life years (DALYs) due to injuries in Mexico in 1990 compared with 2 376 952 (2 224 588 to 2 551 004) DALYs in 2017. We found subnational variation in health loss across Mexico’s states, including concentrated burden in Tabasco, Chihuahua and Zacatecas.

**Conclusions:**

In Mexico, from 1990 to 2017, mortality due to unintentional injuries has decreased, while non-fatal incident cases have increased. However, unintentional injuries continue to cause considerable mortality and morbidity, with patterns that vary by state, age, sex and year. Future research should focus on targeted interventions to decrease injury burden in high-risk populations.

## Introduction

The burden of disability and death due to injury can vary dramatically within countries. Understanding both national trends and subnational variation is therefore critical to good health system planning, particularly because injuries can result in long-term disabilities that present special challenges. Falls and road injuries, for instance, can lead to spinal cord injury and traumatic brain injury, which require specialised and costly medical attention.^1^

A comprehensive assessment of the burden of unintentional injuries across Mexico is timely. Mexico, which has a large population relative to other countries in Latin America and has densely populated urban centres such as Mexico City, has experienced significant economic growth in recent decades and may be in a position to make investments in infrastructure and injury prevention policies that could help mitigate the risks of injury. Systematic reviews have identified effective interventions for preventing road traffic and drowning deaths; however, there is still a gap in knowledge on the effectiveness of other injury interventions.^2^ Health policies, such as Mexico’s universal health coverage programme, can help to extend healthcare services to underserved populations and improve health outcomes related to injuries.^3 4^ Notably, the stability of this programme is in flux, as the current administration is restructuring the health system from the state-run programme (Seguro Popular) to a centralised, integrated federal health system. Government programmes focused on injury prevention and control may also encounter funding implications under a new administration.

The existing literature illuminates some aspects of the unintentional injury burden in Mexico. Past research on the epidemiology of injuries has focused on certain subtypes of unintentional injury such as road injuries or on select subpopulations within Mexico.^5–14^ More detailed and timely assessments of the distribution of morbidity and mortality from injuries—at both the national and subnational levels—could inform programme delivery efforts and help incentivise resource allocation to injury prevention. The Mexican government has also provided detailed information on the burden of injuries, including an extensive report from the National Institute of Public Health, as well as the annual number of deaths due to specific injuries reported by the National Institute of Statistics, Geography, and Informatics (INEGI).^15 16^ Because of findings and trends described in these sources, Mexico’s unintentional injury burden is of interest to broader international and academic communities, and it is important for Mexico’s unintentional injury estimates to be described in injury epidemiology literature.

The annual Global Burden of Diseases, Injuries, and Risk Factors Study (GBD), which produces comprehensive morbidity and mortality estimates by cause for 195 countries and territories, includes country profiles that detail important trends for each location in the GBD study, including Mexico.^17–22^ Drawing on estimates from GBD 2017, this study aimed to examine morbidity and mortality in Mexico from 1990 to 2017 due to unintentional injuries, and to describe burden in terms of subcauses, age patterns, time trends, sex differences and subnational differences.

## Methods

The GBD 2017 study design and methods specific to injury estimation have been described in extensive detail in existing GBD literature. This literature also explains derivation of the sociodemographic index (SDI), a composite measure combining income, education and fertility, which we use to report select results.^17–22^ A summary of key GBD methods is provided in online supplementary appendix 1. The summary in this section focuses on data and methods specific to injuries in Mexico.

10.1136/injuryprev-2019-043532.supp1 Supplementary data

### Cause of death estimation

In GBD 2017, cause of death is defined as the underlying disease or injury that led to a cascade of events leading to death. Cause of death data in GBD 2017 can include vital registration (VR), verbal autopsy, police report and mortuary data, among other records. For injuries in Mexico, GBD 2017 used VR data from 1990 to 2016. GBD 2013 was the first to include subnational locations (states) in Mexico. Cause of death data in the GBD study design undergo additional processing prior to being used analytically, a process that includes garbage code redistribution, where ill-defined causes of death are redistributed to more specific causes based on methods described in more detail in the GBD literature.^21 23^ Once all cause of death data for Mexico were assembled, statistical models using the Cause of Death Ensemble model (CODEm) were conducted for each cause of injury (eg, drowning, pedestrian road injuries). CODEm is described in more detail elsewhere. In summary, it develops a large suite of different classes of statistical models and then selects the best-performing model or ensemble of models to predict the final cause-specific mortality rates (CSMRs) for each cause of death in the GBD cause hierarchy.^24^

For GBD 2017, CODEm models were conducted for 38 different non-mutually exclusive causes of death, where models for both an overall cause such as road injuries as well as a subcause such as pedestrian road injuries were generated. Each model used covariates to help inform the posterior estimate; for instance, alcohol consumption is used as a covariate in many injury cause of death models. Once all models are conducted, a separate process to rescale each cause-specific estimate to fit within the estimate for the parent cause is conducted. This ensures internal consistency between all cause of death estimates and the overall all-cause mortality estimate and is a standard method used in GBD research. The output from this phase is the CSMR for each cause of injury in GBD 2017, specific to each age group, sex and location. Once these values are obtained, Years of Life Lost due to premature mortality (YLLs) are calculated by subtracting age-specific CSMRs from population life tables used in GBD 2017.

### Non-fatal health outcomes

Measuring morbidity in terms of incidence, prevalence and years lived with disability (YLDs) from injuries requires a different approach from the cause of death estimation process. Since each *cause of injury* (eg, road injury) can be associated with various *natures of injury* (eg, spinal cord injury, lower extremity amputation), this process must also account for the distributions of each nature of injury that occurs with each cause. A summary of these processes follows, and they are described in more detail in related publications.^18^

First, models to estimate cause-specific incidence for each cause of injury are conducted using Dismod-MR V.2.1, a Bayesian meta-regression approach that uses differential equations in a compartmental framework to reconcile incidence, prevalence, remission and cause-specific mortality. For injuries in Mexico, this modelling process used CSMRs from the above cause of death modelling process as DisMod inputs. In addition, the models for incidence used incidence data from the Mexico National Survey of Health and Nutrition (ENSANUT, formerly ENSA in 2000) and hospital admission rates from the Mexico Ministry of Health hospital discharges database. The ENSANUT 2000, 2006 and 2012 provided information on various health conditions and access to health services.^25–27^ ENSANUT data were collected in 45 000–50 000 households, depending on the year. The surveys included themes of demographic characteristics, health status, injuries, disability, maternal health and the use of health services. The hospital discharges database from the Mexico Ministry of Health included International Classification of Diseases (ICD) diagnosis coding for cause of injury for each hospital admission, which is used for injuries in GBD to approximate incidence of injuries requiring medical care. Since injuries are also treated in the outpatient setting, a ratio of outpatient to inpatient care for each cause of injury is estimated in locations where both inpatient and outpatient care are available, which is then used to produce separate incidence estimates for injuries receiving outpatient care.

A complex analytical pipeline following the initial incidence models was then executed, which estimated the incidence of each cause–nature combination of injury (eg, road injuries leading to traumatic brain injury) and then converted incidence to prevalence using the estimated duration of each nature of injury. Once prevalence of each cause–nature combination was estimated, YLDs were calculated using GBD disability weights described in more detail elsewhere.^28^ The output from the non-fatal estimation process includes incidence, prevalence and YLDs for each age group, sex, year from 1990 to 2017, and state in Mexico. Mexico City, a federal entity, is also included in the analysis.

### Disability-adjusted life years

On completion of cause-specific mortality estimation and non-fatal health outcomes estimation, disability-adjusted life years (DALYs) were calculated by adding YLLs and YLDs for each cause of injury.

### GATHER statement

GBD 2017 adheres to the Guidelines for Accurate and Transparent Health Estimates Reporting (GATHER).^29^ GATHER is described in more detail in online supplementary appendix 2.

10.1136/injuryprev-2019-043532.supp2 Supplementary data

## Results

### Injury types

The GBD defines three broad categories of injuries: transport injuries, non-transport unintentional injuries (abbreviated ‘unintentional injuries’, recognising that transport injuries are also unintentional), and self-harm and interpersonal violence. This study focuses only on transport and unintentional injuries; hence, ‘unintentional injuries’ in this study include both transport and unintentional categories of injury and exclude self-harm and interpersonal violence. Online supplementary appendix table 1 provides deaths, YLLs, incidence, prevalence, YLDs and DALYs due to unintentional injury in Mexico in 1990 and 2017 in terms of counts and age-standardised rates for each cause of injury. online supplementary appendix table 2 provides the same measures and metrics for each state in Mexico.

10.1136/injuryprev-2019-043532.supp3 Supplementary data

10.1136/injuryprev-2019-043532.supp4 Supplementary data

In 1990, there were 45 363 deaths (95% uncertainty interval 44 662 to 46 038) from unintentional injuries in Mexico, which amounted to 10.5% (10.3% to 10.7%) of all deaths in that year. By 2017, this number had decreased to 42 702 deaths (41 439 to 43 745) and the percentage of all deaths across all ages decreased to 6.0% (5.8% to 6.1%). These deaths caused 2 494 521 (2 438 341 to 2 542 254) YLLs in 1990 (13.5% (13.2% to 13.8%) of all YLLs) and 1 894 389 (1 841 591 to 1 945 556) YLLs in 2017 (9.5% (9.3% to 9.8%) of all YLLs). The age-standardised death rate from unintentional injuries was 65.2 (64.4 to 66.1) per 100 000 in 1990 and 35.1 (34.1 to 36.0) per 100 000 in 2017, slightly lower than the global rate of 40.8 (39.2 to 41.9) per 100 000 in 2017.

In terms of non-fatal outcomes, there were 3 120 211 (2 879 993 to 3 377 945) new cases of unintentional injury in Mexico in 1990, which increased to 5 234 214 (4 812 615 to 5 701 669) new cases of injury in 2017. These cases of injury equated to prevalence estimates of 9854 (9279 to 10 577) cases per 100 000 in 1990 and 9209 (8624 to 9966) cases per 100 000 in 2017, causing 267 435 (195 876 to 356 003) YLDs in 1990 and 482 563 (347 809 to 651 641) YLDs in 2017.

In 1990, there were 2 761 957 (2 676 267 to 2 859 777) DALYs due to unintentional injuries in Mexico, representing 10.9% (10.4% to 11.5%) of total DALYs and equating to an age-standardised rate of 3342 (3203 to 3469) DALYs per 100 000. In 2017, the total DALYs due to injury decreased to 2 376 952 (2 224 588 to 2 551 004), representing 7.4% (7.1% to 7.8%) of total DALYs and equating to an age-standardised rate of 1887 (1762 to 2029) per 100 000. Tabasco (2645 (2439 to 2880)), Chihuahua (2308 (2113 to 2524)) and Zacatecas (2299 (2118 to 2503)) were the states with the highest age-standardised rates of DALYs per 100 000 in 2017.

**Figure 1:**
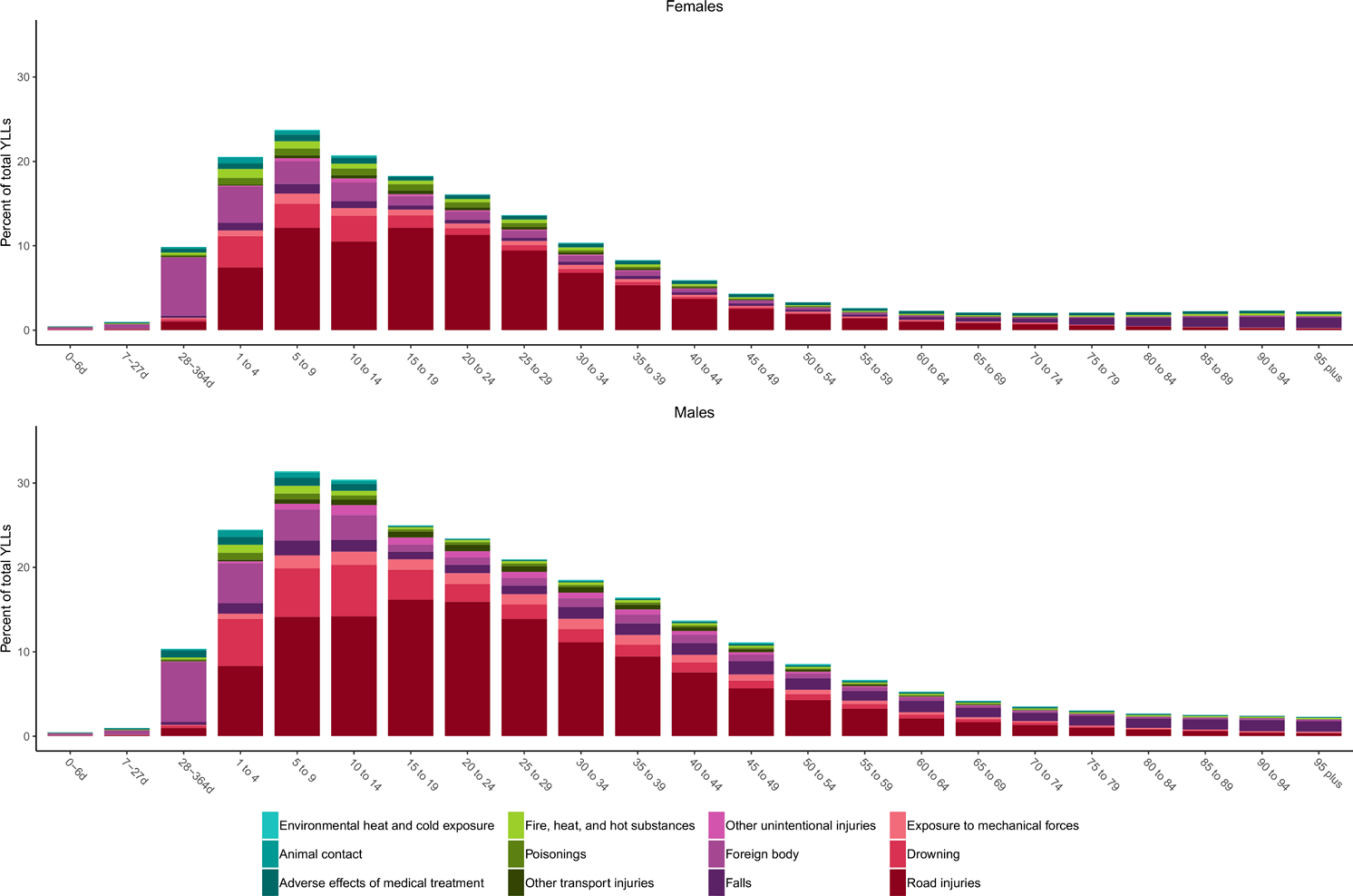
Percentage of YLLs by cause of unintentional injury and age group for males and females in 2017.

In terms of sex, males experienced higher mortality rates and DALYs for all causes of injury, with unintentional injuries causing more than 20% of overall mortality in males aged 15–29 years. The percentages of YLLs for each cause and by age group for 2017 are shown in figure 1 for females and males. This figure further highlights that YLLs for males are generally higher than for females, and fatal injury burden is skewed towards ages 1–19. In both males and females, road injuries are the primary cause of YLLs from ages 1 to 74. Drowning, foreign body injuries (which include pulmonary aspiration and foreign body in airway) and falls are more predominant causes of YLLs in children and the elderly.

### Transport and road injuries

**Figure 2:**
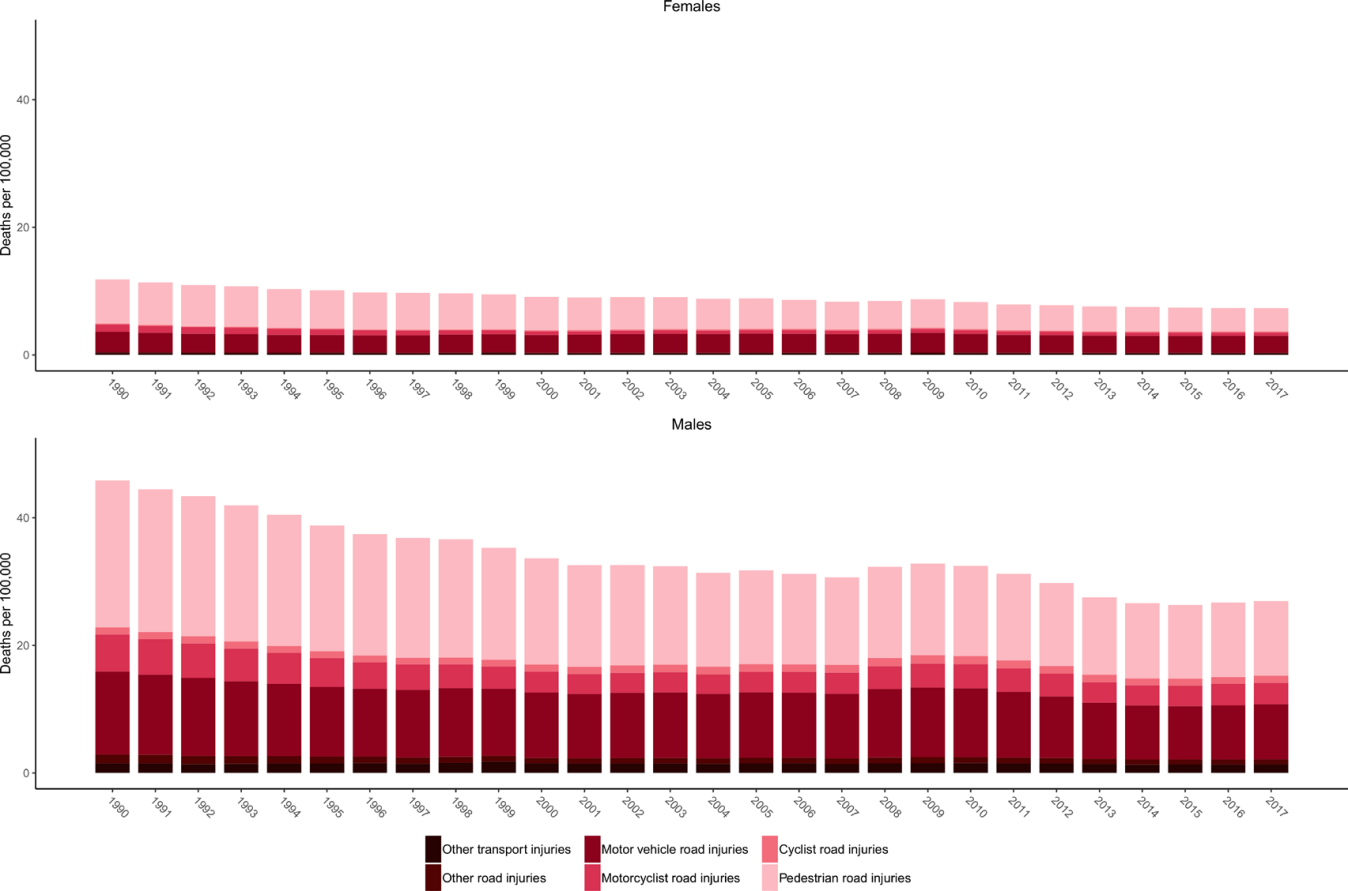
Age-standardised mortality rates due to transport injuries by subtype from 1990 to 2017 for females and males.

In 2017, 3.0% (2.9% to 3.1%) of all deaths in Mexico were attributed to transport injuries, which include road injuries and other transport injuries. In 1990, there were 20 643 (20 255 to 21 078) deaths due to transport injuries, causing 1 148 232 (1 123 211 to 1 173 667) YLLs. By 2017, there were 21 153 (20 361 to 21 908) deaths due to transport injuries in Mexico, causing 1 022 815 (988 111 to 1 057 465) YLLs. For road injuries, age-standardised DALYs per 100 000 decreased by 39.3% (37.0% to 42.0%) from 1990 to 2017. Figure 2 shows the age-standardised mortality rates due to transport injuries and their subcauses from 1990 to 2017 for females and males. These results show how pedestrian road injuries are a leading cause of death among transport injuries for both males and females, while motor vehicle injuries and motorcyclist road injuries generally are the second and third leading causes of transport injury deaths, respectively. Cyclist injuries, other road injuries and other transport injuries are less common causes of death among the transport subcauses. These patterns are generally true across the time span of the study and for males and females. Tabasco (26.6 (24.2 to 28.8)), Zacatecas (23.4 (21.5 to 25.3)) and Sonora (20.1 (18.6 to 21.6)) had the highest age-standardised mortality rates per 100 000 due to road injuries in 2017.

### Non-transport unintentional injuries

**Figure 3:**
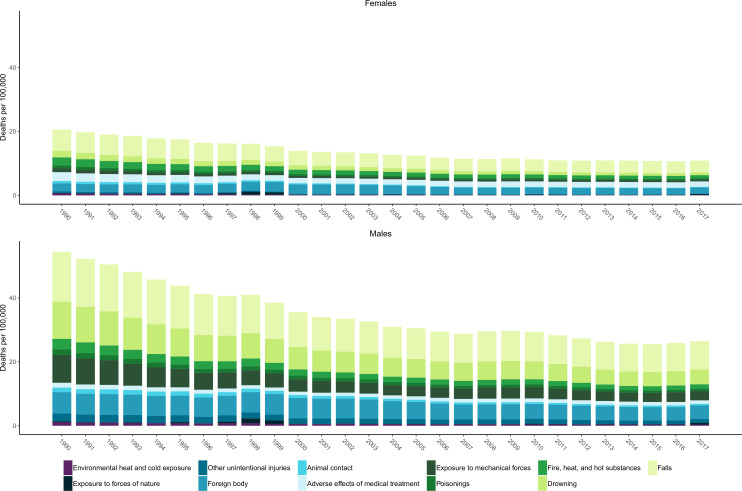
Age-standardised mortality rates due to non-transport unintentional injuries by subtype from 1990 to 2017 for females and males.

In 2017, 3.0% (3.0% to 3.1%) of all deaths in Mexico were attributed to unintentional injuries. In 1990, there were 24 719 (24 250 to 25 092) deaths due to unintentional injuries, resulting in 1 346 289 (1 310 694 to 1 375 751) YLLs. By 2017, there were 21 549 (20 944 to 22 220) deaths due to unintentional injuries, causing 871 574 (848 992 to 907 600) YLLs. Age-standardised DALYs per 100 000 due to unintentional injuries decreased by 46.7% (44.0% to 49.4%) from 1990 to 2017. Figure 3 shows the age-standardised mortality rates due to unintentional injuries from 1990 to 2017 for females and males. Similar to figure 2, this figure demonstrates how injury burden (expressed in CSMR in this case) over time has been higher in males than in females. Falls, drowning, foreign body injuries and exposure to mechanical forces are generally the unintentional injuries with the highest CSMRs. This figure also demonstrates that CSMRs for the subcauses of unintentional injuries have generally trended downward over time, with some variation by injury cause and with a general tapering and plateau of overall unintentional CSMRs in more recent years, with some slight increases appearing between 2016 and 2017. Online supplementary appendix table 1 also reveals the differential YLD and YLL distribution forming overall DALYs by cause, with DALY rates from certain causes such as falls being formed more by YLDs than YLLs, while other causes are more YLL-dominant, for example drowning and pulmonary aspiration and foreign body in airway.

### Injuries by state in Mexico

**Figure 4:**
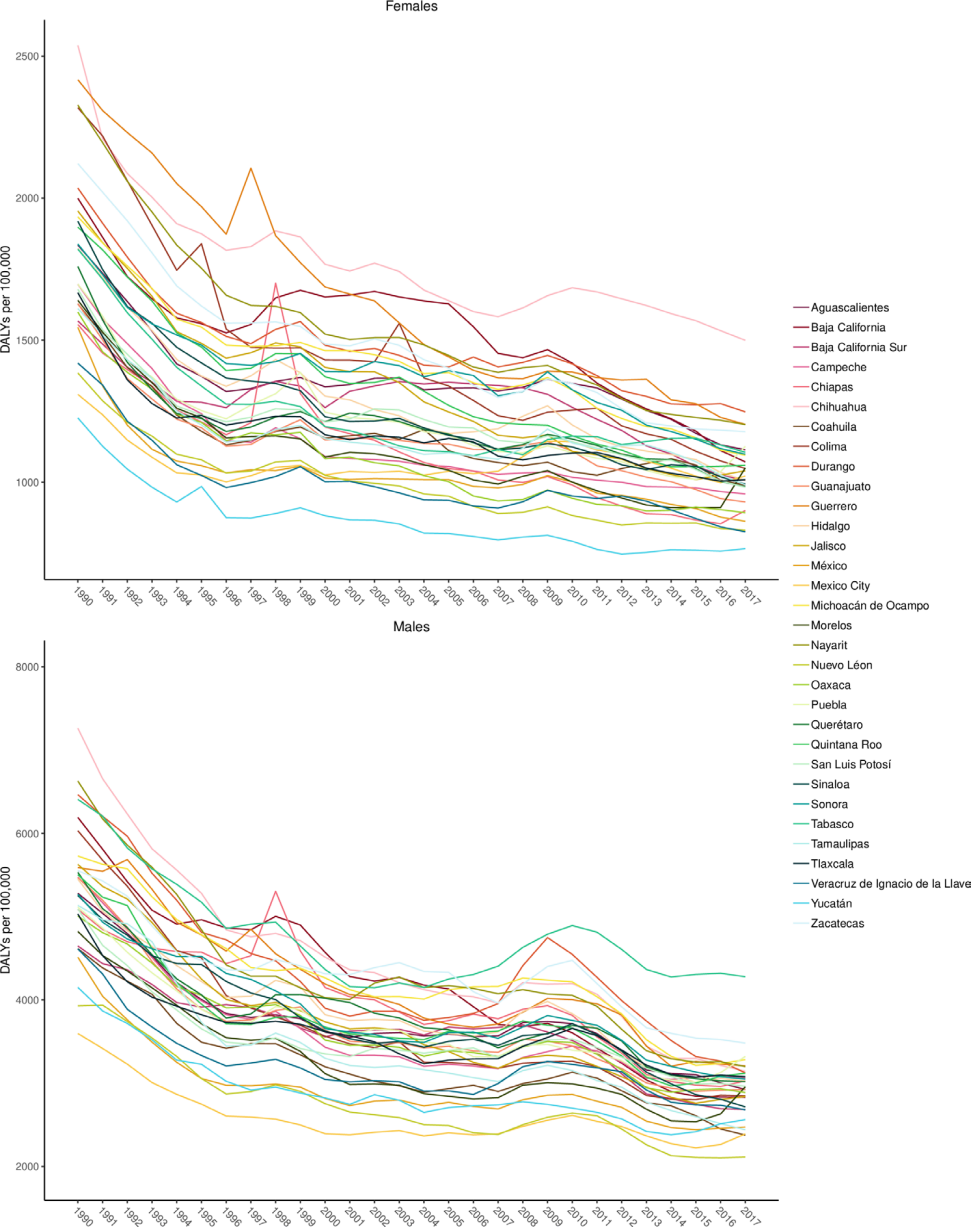
Age-standardised disability-adjusted life years (DALYs) due to unintentional injuries per 100 000 for males and for females by year for each state in Mexico.

**Figure 5:**
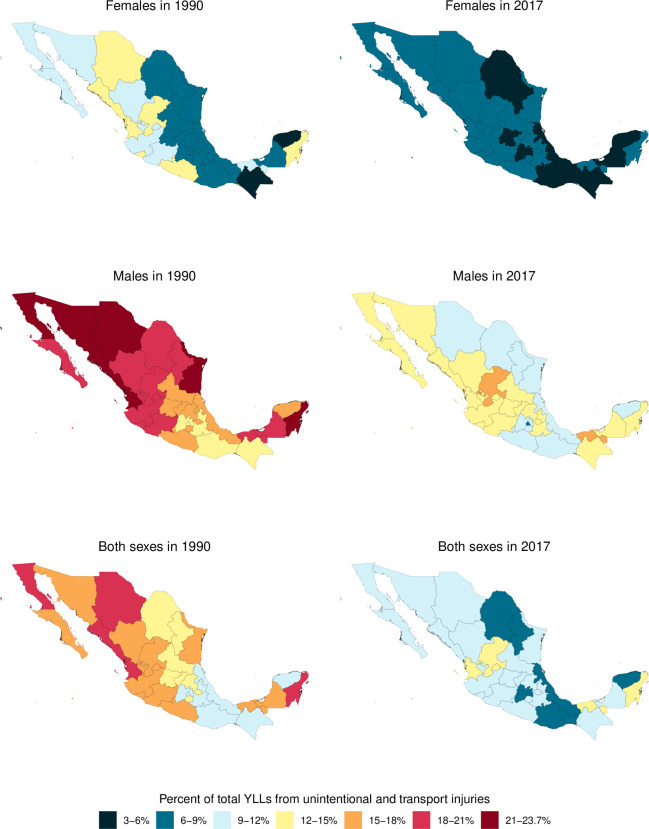
Percentage of total YLLs due to unintentional injuries for males, females and both sexes combined in 1990 and 2017, by state, in Mexico.

Figure 4 shows age-standardised DALYs per 100 000 for males and females by year for each state in Mexico. This figure demonstrates that the burden of injuries has steadily declined since 1990 in most states. In 2017, Chihuahua and Tabasco had the highest burden of transport and unintentional injuries for females and males, respectively. Figure 5 shows the percentage of all-cause YLLs that are due to injuries for males, females and both sexes combined in 1990 and 2017 by state in Mexico. This figure shows that there is considerable variation in the percentage of all-age YLLs due to injury between states in Mexico. Many states experienced dramatic decreases in YLLs over the past 27 years, particularly among males in Tamaulipas and Chihuahua. Notably, Mexico City had the lowest percentage of YLLs in males in 2017.

**Figure 6:**
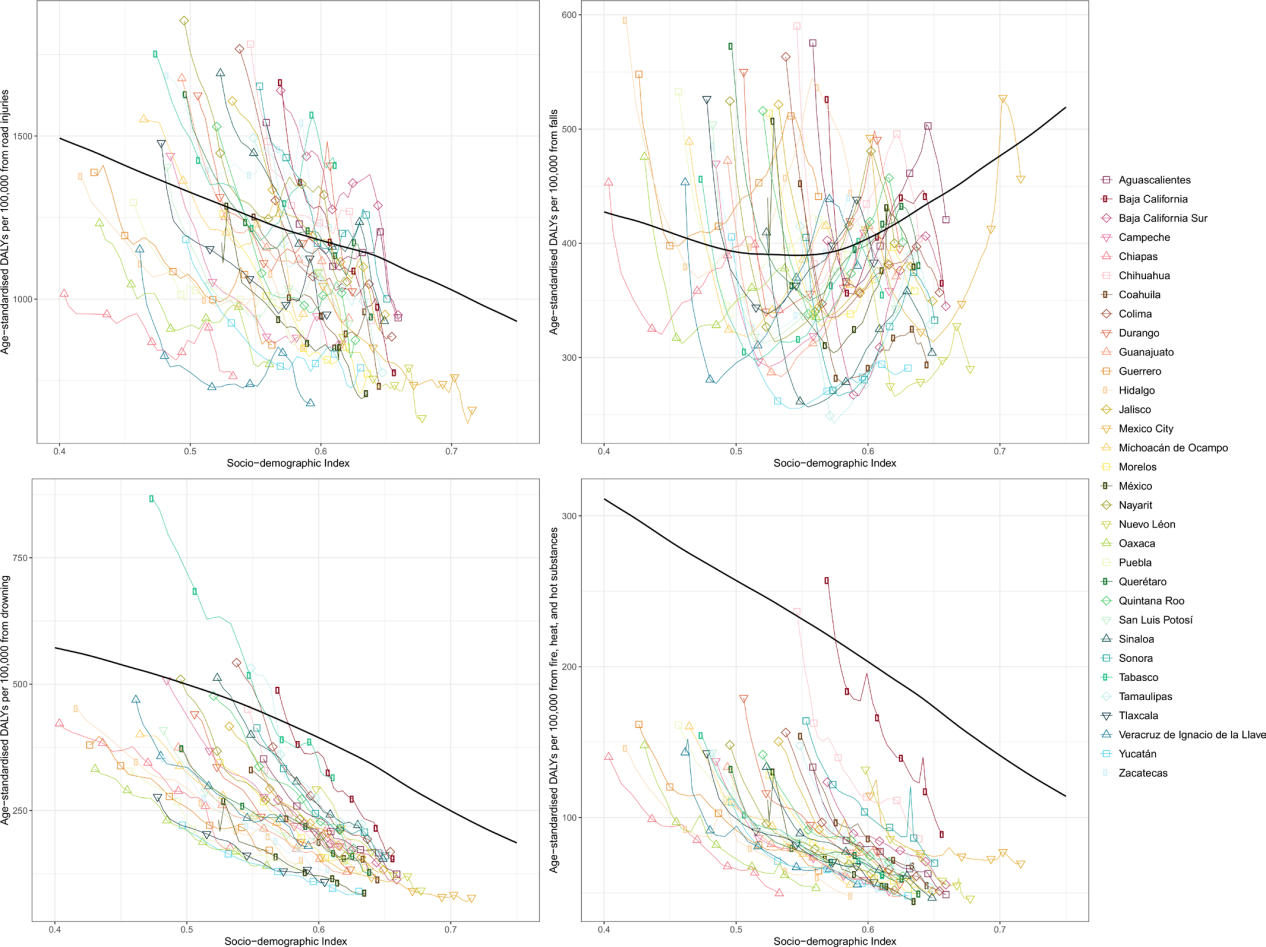
Age-standardised disability-adjusted life years (DALYs) by state from 1990 to 2017 by sociodemographic index (SDI) and global expected DALYs by SDI. The black line represents expected values based on the mean DALY rate for all locations at each SDI level. For each state, points from left to right depict estimates from each year from 1990 to 2017.

Figure 6 shows age-standardised DALY rates by state from 1990 to 2017 by SDI for select injuries: road injuries, falls, drowning, and fire, heat and hot substances. The black line represents the correlation of global, average age-standardised DALY rates by SDI. For road injuries, by 2017, DALY rates in all states other than Tabasco fell below the global average for their SDI. For falls, states experienced decreases in DALY rates in the 1990s, but as SDI continued to increase, rates steadily increased as well through the 2000s. DALY rates for drowning steadily decreased over time as SDI increased, resulting in all states arriving well below the global average in 2017. Finally, for fire, heat and hot substances, DALY rates have decreased for all states as SDI increased over time. Baja California has a notably higher burden than other states in Mexico.

## Discussion

In this study, we reported morbidity and mortality estimates of unintentional injuries in Mexico from GBD 2017, including subnational analysis by states in Mexico. Our review of the GBD 2017 estimates for injury burden in Mexico revealed a number of notable trends that vary dynamically by age, sex, year, subnational location and injury type. Some important key themes emerged that could help frame future health policy and research in Mexico.

First, GBD 2017 highlighted pronounced time trends in the burden of unintentional injury in Mexico. Age-standardised mortality rates have improved significantly over time, which may be associated with improvements in healthcare access and quality; however, incidence, prevalence and YLDs have remained relatively stagnant, particularly due to the burden of road traffic injuries. Online supplementary appendix table 1 shows how these trends have varied by specific subcause: for example, pedestrian road injuries had essentially the same YLDs per 100 000 in 1990 and 2017, while motor vehicle road injuries increased. Second, this study illustrated the extent to which injury disproportionately affects younger and working ages in Mexico. As the figures in this study indicated, a significant proportion of the injury burden is experienced in younger age groups, starting in the teenage years and continuing throughout adulthood. The health and well-being of the working-age population are critical, as Mexico is projected to experience a demographic shift that will increase the dependency of retirees on working-age individuals.^30^ The proportion of people aged 65 or older is expected to triple by 2050, which could also lead to increased mortality and incidence due to falls in Mexico.

Third, this study confirmed differences between sexes in terms of injury burden. GBD 2017 focused extensively on sex differences across all causes of health loss, and the burden of injuries is one area of burden where males experience much higher rates of death and disability than females.

Fourth, our analysis found considerable heterogeneity in the injury burden across states in Mexico. Specifically, we found concentrated burden of unintentional injuries in select states in Mexico, such as Zacatecas and Tabasco. Both Tabasco and Zacatecas have large rural populations, 43% and 41% rural, respectively, which have been found to have higher motor vehicle injury rates than urban areas.^31 32^ These findings underscore the importance of directing attention and resources to mitigating the injury burden in these particular areas, and also likely within the key populations described above in each of these areas. It is also important to note the heterogeneity of disease burden by state, where states have age-standardised mortality rates that are double those of other states. For example, the mortality rate due to falls in Quintana Roo is double that of Tamaulipas. As GBD research continues to add more detailed cause classifications, such patterns can be used to inform future research and policy to address burden at both the state and national levels. Additionally, lessons can be learnt from states such as Tamaulipas and Chihuahua, which have significantly decreased mortality due to drowning, falls and road injuries in the past 27 years.

It is important to note that GBD 2017 estimates for unintentional injury deaths and for all-cause mortality in Mexico are higher than official national statistics.^16^ This effect is partly due to a process in the GBD cause of death modelling framework, whereby deaths from level 1 causes are adjusted to match all-cause mortality, and subsequently deaths from level 2 causes are then adjusted to match the deaths from their level 1 causes, continuing down to the most detailed level of the GBD cause hierarchy. GBD estimates are also higher due to garbage code redistribution, where ill-defined deaths in VR data are redistributed to target underlying causes of death by age, sex, location and year. These results highlight the need to further analyse the burden of unintentional injuries among females, as the high rates of injury among males can mask the trends specific to females.

This study has several limitations beyond the limitations already noted in the GBD literature.^17–22^ With regard to non-fatal estimation, while Mexico has more data for injury incidence than many other countries due to the availability of ENSANUT and the national hospital discharge database, the data sources may still have limitations, such as reporting biases for less severe injuries or for areas that lack reliable access to healthcare. The hospital data used in this study are from the Ministry of Health and do not include discharges from the private sector as well as a portion of discharges from the public sector, which may have unidentified biases compared with comprehensive hospital discharge records across Mexico.

Trends in motorcycle fatalities estimates have also pointed to a limitation in accounting for potential changes to estimates due to differences in the ICD system used in a given year. In 1998, Mexico switched from ICD-9 to ICD-10, which includes more specific vehicle type codes, leading to changes in garbage code redistribution, which is likely influencing the observed decrease in motorcycle deaths and will require further analysis. An additional limitation is that our prevalence estimates rely on generalising probabilities of long-term disability and duration of short-term disability from injury causes from studies conducted outside of Mexico. Future injury burden research in Mexico would benefit from adding long-term follow-up studies that were collected in Mexico.

Despite these limitations, this study represents the most comprehensive review of unintentional injury burden in Mexico to date, providing a valuable case study for other Latin American countries. Future research in Mexico should focus on collecting detailed and contextual injury data for each state and monitor progress over time, given the recent changes to the healthcare system. Future efforts should also focus on more detailed injury burden measurements at the state level to measure both the cause and nature of injury to understand the prevention and treatment efforts that would benefit local injury burden.

## Conclusion

The burden of unintentional injury in Mexico is complex and constitutes an important and largely preventable portion of disease and injury burden in Mexico. This study showed select subpopulations and states within Mexico experience particularly high burden of injury and could benefit from more focused intervention efforts and resource allocation to reduce future burden of unintentional injury in these populations.

**What is already known on the subject**

- Previous assessments focusing on specific states, demographic groups and types of unintentional injury in Mexico have found notable variation in burden; however, unintentional injuries are known to pose risk to all members of society.
- Previous understanding of unintentional injuries in Mexico has called for different emphasis placed on transport policy, industry regulation and socioeconomic factors that drive certain behaviours.

**What this study adds**

- This research highlighted subnational variation of unintentional injuries across Mexico, with a disproportionate burden of injury in Tabasco, Chihuahua and Zacatecas.
- This study adds measures of health loss including disability-adjusted life years due to unintentional injuries in Mexico.
- This study confirms previous research findings that males experience much higher rates of disability and death from unintentional injuries than females in Mexico.

10.1136/injuryprev-2019-043532.supp5 Supplementary data

10.1136/injuryprev-2019-043532.supp6 Supplementary data

10.1136/injuryprev-2019-043532.supp7 Supplementary data

10.1136/injuryprev-2019-043532.supp8 Supplementary data

10.1136/injuryprev-2019-043532.supp9 Supplementary data

10.1136/injuryprev-2019-043532.supp10 Supplementary data

10.1136/injuryprev-2019-043532.supp11 Supplementary data

10.1136/injuryprev-2019-043532.supp12 Supplementary data

10.1136/injuryprev-2019-043532.supp13 Supplementary data

10.1136/injuryprev-2019-043532.supp14 Supplementary data

10.1136/injuryprev-2019-043532.supp15 Supplementary data

10.1136/injuryprev-2019-043532.supp16 Supplementary data

10.1136/injuryprev-2019-043532.supp17 Supplementary data

10.1136/injuryprev-2019-043532.supp18 Supplementary data

10.1136/injuryprev-2019-043532.supp19 Supplementary data

10.1136/injuryprev-2019-043532.supp20 Supplementary data

10.1136/injuryprev-2019-043532.supp21 Supplementary data

10.1136/injuryprev-2019-043532.supp22 Supplementary data

10.1136/injuryprev-2019-043532.supp23 Supplementary data

10.1136/injuryprev-2019-043532.supp24 Supplementary data

10.1136/injuryprev-2019-043532.supp25 Supplementary data

10.1136/injuryprev-2019-043532.supp26 Supplementary data

10.1136/injuryprev-2019-043532.supp27 Supplementary data

10.1136/injuryprev-2019-043532.supp28 Supplementary data

10.1136/injuryprev-2019-043532.supp29 Supplementary data

10.1136/injuryprev-2019-043532.supp30 Supplementary data

10.1136/injuryprev-2019-043532.supp31 Supplementary data

10.1136/injuryprev-2019-043532.supp32 Supplementary data

10.1136/injuryprev-2019-043532.supp33 Supplementary data

10.1136/injuryprev-2019-043532.supp34 Supplementary data

10.1136/injuryprev-2019-043532.supp35 Supplementary data

10.1136/injuryprev-2019-043532.supp36 Supplementary data
