# Correction: *Unintentional injuries in Mexico, 1990–2017: findings from the Global Burden of Disease Study 2017*

**DOI:** 10.1136/injuryprev-2019-043532corr1

**Published:** 2020-09-28

Híjar M, Pérez-Núñez R, Hidalgo-Solórzano E, *et al*. Unintentional injuries in Mexico, 1990–2017: findings from the Global Burden of Disease Study 2017. *Inj Prev* 2020. doi: 10.1136/injuryprev-2019-043532.

This article was previously published with error in affiliation for Elisa Hidalgo-Solórzano. The correct affiliation is below:

3 Center for Health Systems Research, National Institute of Public Health, Cuernavaca, Mexico

