# Supplementary material for: Unintentional injuries in Mexico, 1990–2017: findings from the Global Burden of Disease Study 2017
Source: Inj Prev. 2020 Apr 1;26(Suppl 1):i154–61. doi: 10.1136/injuryprev-2019-043532 (PMC7571365; doi:10.1136/injuryprev-2019-043532)
Supplement: Supplementary data [file injuryprev-2019-043532supp003.pdf]

Death, YLL, Incidence, prevalence, YLD, and DALY counts and rates per 100,000 for all injuries in Mexico in 1990 and 2017

| Cause | Deaths | | | | Years of life lost | | | |
|---|---|---|---|---|---|---|---|---|
| | All-age counts (95% UI) | | Age-standardised rates per 100,000 (95% UI) | | All-age counts (95% UI) | | Age-standardised rates per 100,000 (95% UI) | |
| | 1990 | 2017 | 1990 | 2017 | 1990 | 2017 | 1990 | 2017 |
| **Transport and unintentional injuries** | **45 363 (44 662 to 46 038)** | **42 702 (41 439 to 43 745)** | **65.2 (64.4 to 66.1)** | **35.1 (34.1 to 36.0)** | **2 494 521 (2 438 341 to 2 542 254)** | **1 894 389 (1 841 591 to 1 946 556)** | **2 897.3 (2 846.5 to 2 943.3)** | **1 490.8 (1 449.7 to 1 532.9)** |
| **Transport injuries** | **20 643 (20 255 to 21 078)** | **21 153 (20 361 to 21 906)** | **28.2 (27.8 to 28.9)** | **16.8 (16.2 to 17.4)** | **1 148 232 (1 123 211 to 1 173 667)** | **1 022 815 (988 111 to 1 057 465)** | **1 349.8 (1 323.4 to 1 378.7)** | **790.4 (763.6 to 817.1)** |
| Road injuries | 20 013 (19 636 to 20 435) | 20 170 (19 427 to 20 909) | 27.4 (26.9 to 27.9) | 16.0 (15.4 to 16.6) | 1 113 634 (1 089 220 to 1 138 820) | 976 016 (944 208 to 1 009 136) | 1 308.6 (1 283.2 to 1 337.4) | 754.4 (730.0 to 780.1) |
| Pedestrian road injuries | 9 998 (9 752 to 10 242) | 9 191 (8 886 to 9 619) | 14.7 (14.4 to 15.0) | 7.5 (7.2 to 7.8) | 534 843 (519 262 to 550 096) | 402 211 (387 943 to 419 863) | 640.6 (624.1 to 657.0) | 315.5 (304.2 to 329.2) |
| Cyclist road injuries | 471 (455 to 487) | 793 (755 to 838) | 0.6 (0.6 to 0.7) | 0.6 (0.6 to 0.7) | 26 238 (25 341 to 27 134) | 36 118 (34 440 to 38 036) | 31.4 (30.4 to 32.4) | 28.1 (26.8 to 29.6) |
| Motorcyclist road injuries | 2 759 (2 666 to 2 845) | 2 506 (2 401 to 2 648) | 3.4 (3.3 to 3.5) | 1.9 (1.8 to 2.0) | 162 249 (157 008 to 168 023) | 140 631 (134 554 to 148 441) | 185.3 (179.7 to 191.4) | 106.5 (101.9 to 112.4) |
| Motor vehicle road injuries | 6 256 (6 084 to 6 507) | 7 151 (6 803 to 7 393) | 8.0 (7.7 to 8.3) | 5.6 (5.3 to 5.7) | 360 150 (349 993 to 374 482) | 371 496 (355 586 to 384 266) | 416.7 (405.1 to 433.1) | 284.6 (272.5 to 294.2) |
| Other road injuries | 535 (470 to 567) | 529 (497 to 562) | 0.7 (0.6 to 0.8) | 0.4 (0.4 to 0.4) | 30 154 (26 376 to 32 062) | 25 561 (24 057 to 27 114) | 34.7 (30.5 to 36.8) | 19.7 (18.6 to 20.9) |
| Other transport injuries | 630 (609 to 654) | 984 (908 to 1 029) | 0.9 (0.9 to 0.9) | 0.8 (0.7 to 0.8) | 34 598 (33 374 to 35 939) | 46 799 (43 588 to 48 952) | 41.1 (39.7 to 42.7) | 36.1 (33.6 to 37.7) |
| **Unintentional injuries** | **24 719 (24 250 to 25 092)** | **21 549 (20 944 to 22 220)** | **37.0 (36.4 to 37.4)** | **18.4 (17.8 to 18.9)** | **1 346 289 (1 310 694 to 1 375 751)** | **871 574 (846 992 to 907 600)** | **1 547.6 (1 515.3 to 1 574.0)** | **700.4 (681.5 to 730.4)** |
| Falls | 5 210 (5 121 to 5 325) | 6 827 (6 405 to 7 016) | 10.9 (10.7 to 11.2) | 6.2 (5.8 to 6.4) | 178 068 (174 092 to 182 665) | 173 248 (162 372 to 178 651) | 271.4 (266.5 to 277.8) | 143.4 (134.5 to 147.7) |
| Drowning | 5 532 (5 402 to 5 663) | 3 308 (3 214 to 3 403) | 6.7 (6.6 to 6.9) | 2.6 (2.6 to 2.7) | 346 851 (336 897 to 356 814) | 173 325 (168 260 to 179 042) | 368.5 (359.2 to 377.7) | 136.1 (132.1 to 140.7) |
| Fire, heat, and hot substances | 1 866 (1 818 to 1 922) | 1 322 (1 229 to 1 365) | 2.9 (2.9 to 3.0) | 1.2 (1.1 to 1.2) | 100 718 (96 897 to 104 867) | 45 070 (41 842 to 46 596) | 114.5 (111.1 to 118.1) | 35.7 (34.1 to 36.9) |
| Poisonings | 1 071 (1 036 to 1 104) | 712 (675 to 737) | 1.3 (1.3 to 1.4) | 0.6 (0.5 to 0.6) | 67 269 (64 728 to 69 538) | 34 748 (33 009 to 36 084) | 71.6 (69.2 to 73.8) | 27.4 (26.1 to 28.5) |
| Poisoning by carbon monoxide | 404 (390 to 418) | 302 (285 to 314) | 0.5 (0.5 to 0.5) | 0.2 (0.2 to 0.3) | 24 446 (23 547 to 25 414) | 14 429 (13 684 to 15 039) | 26.6 (25.7 to 27.6) | 11.3 (10.7 to 11.8) |
| Poisoning by other means | 667 (643 to 689) | 410 (387 to 427) | 0.8 (0.8 to 0.8) | 0.3 (0.3 to 0.3) | 42 823 (41 024 to 44 384) | 20 318 (19 221 to 21 180) | 45.0 (43.2 to 46.5) | 16.1 (15.3 to 16.8) |
| Exposure to mechanical forces | 3 481 (3 401 to 3 555) | 2 092 (2 020 to 2 163) | 4.8 (4.7 to 4.9) | 1.7 (1.6 to 1.7) | 192 501 (187 587 to 196 929) | 93 786 (90 787 to 97 033) | 228.5 (223.2 to 233.4) | 72.9 (70.6 to 75.4) |
| Unintentional firearm injuries | 1 730 (1 653 to 1 876) | 768 (635 to 838) | 2.2 (2.1 to 2.4) | 0.6 (0.5 to 0.6) | 98 866 (94 175 to 106 466) | 37 263 (30 003 to 39 685) | 114.6 (109.5 to 124.1) | 28.6 (23.1 to 30.5) |
| Other exposure to mechanical forces | 1 751 (1 562 to 1 817) | 1 324 (1 265 to 1 445) | 2.5 (2.3 to 2.6) | 1.1 (1.0 to 1.2) | 93 635 (88 752 to 97 259) | 56 524 (53 883 to 62 743) | 114.0 (101.5 to 118.2) | 44.3 (42.3 to 49.1) |
| Adverse effects of medical treatment | 1 217 (1 071 to 1 312) | 1 601 (1 513 to 2 044) | 2.2 (2.0 to 2.4) | 1.4 (1.4 to 1.8) | 55 963 (48 099 to 60 529) | 50 410 (47 033 to 68 678) | 69.3 (60.5 to 75.0) | 41.9 (39.1 to 57.0) |
| Animal contact | 909 (848 to 972) | 396 (382 to 430) | 1.1 (1.0 to 1.1) | 0.3 (0.3 to 0.3) | 63 961 (58 851 to 69 209) | 17 400 (16 653 to 18 312) | 62.3 (57.8 to 66.8) | 14.2 (13.6 to 15.0) |
| Venomous animal contact | 745 (676 to 805) | 266 (258 to 281) | 0.8 (0.8 to 0.9) | 0.2 (0.2 to 0.2) | 54 537 (48 790 to 59 649) | 12 079 (11 087 to 12 970) | 51.8 (46.7 to 56.2) | 9.9 (9.1 to 10.7) |
| Non venomous animal contact | 164 (136 to 189) | 129 (121 to 160) | 0.2 (0.2 to 0.3) | 0.1 (0.1 to 0.1) | 9 424 (7 503 to 12 044) | 5 321 (4 963 to 6 602) | 10.5 (8.6 to 12.4) | 4.3 (4.0 to 5.3) |
| Foreign body | 3 663 (3 537 to 3 779) | 3 622 (3 489 to 3 811) | 4.5 (4.4 to 4.6) | 3.0 (2.9 to 3.2) | 248 615 (232 812 to 259 214) | 202 178 (192 428 to 217 980) | 247.0 (237.7 to 255.5) | 164.6 (156.4 to 177.4) |
| Pulmonary aspiration and foreign body in airway | 3 496 (3 377 to 3 607) | 3 534 (3 405 to 3 705) | 4.3 (4.2 to 4.4) | 2.9 (2.8 to 3.1) | 232 688 (222 380 to 241 918) | 197 297 (187 736 to 211 192) | 235.4 (226.6 to 243.5) | 160.8 (152.9 to 172.4) |
| Foreign body in eyes | | | | | | | | |
| Foreign body in other body part | 167 (130 to 177) | 87 (78 to 137) | 0.2 (0.2 to 0.2) | 0.1 (0.1 to 0.1) | 10 977 (8 325 to 11 659) | 4 881 (4 383 to 7 897) | 11.6 (8.9 to 12.3) | 3.8 (3.4 to 6.1) |
| Environmental heat and cold exposure | 509 (496 to 527) | 283 (259 to 293) | 0.9 (0.9 to 0.9) | 0.2 (0.2 to 0.2) | 23 548 (22 697 to 24 840) | 10 330 (9 482 to 10 688) | 31.4 (30.5 to 32.4) | 8.2 (7.5 to 8.5) |
| Exposure to forces of nature | 143 (134 to 154) | 528 (507 to 550) | 0.2 (0.2 to 0.2) | 0.4 (0.4 to 0.4) | 8 417 (7 824 to 9 118) | 27 248 (25 938 to 28 653) | 9.4 (8.8 to 10.1) | 21.4 (20.5 to 22.5) |
| Other unintentional injuries | 1 118 (1 092 to 1 144) | 864 (834 to 897) | 1.4 (1.4 to 1.5) | 0.7 (0.7 to 0.7) | 65 378 (63 744 to 67 012) | 43 882 (42 236 to 45 624) | 73.7 (71.9 to 75.4) | 33.6 (32.4 to 35.0) |

| Cause | Incidence | | | | Prevalence | | | |
|---|---|---|---|---|---|---|---|---|
| | All-age counts (95% UI) | | Age-standardised rates per 100,000 (95% UI) | | All-age counts (95% UI) | | Age-standardised rates per 100,000 (95% UI) | |
| | 1990 | 2017 | 1990 | 2017 | 1990 | 2017 | 1990 | 2017 |
| **Transport and unintentional injuries** | **3 120 210 (2 879 993 to 3 377 945)** | **5 234 215 (4 812 615 to 5 701 670)** | **4 223.1 (3 888.9 to 4 576.6)** | **4 296.6 (3 946.1 to 4 674.3)** | **6 098 715 (5 729 397 to 6 530 631)** | **11 230 232 (10 500 874 to 12 132 022)** | **9 854.4 (9 278.8 to 10 576.6)** | **9 209.3 (8 624.0 to 9 966.1)** |
| **Transport injuries** | **272 476 (231 107 to 316 625)** | **600 530 (521 236 to 687 285)** | **355.9 (310.4 to 405.6)** | **471.6 (409.9 to 538.5)** | **656 993 (612 811 to 699 213)** | **1 706 211 (1 589 541 to 1 832 077)** | **1 096.6 (1 026.6 to 1 172.6)** | **1 379.5 (1 285.8 to 1 480.8)** |
| Road injuries | 258 653 (221 388 to 303 024) | 587 608 (507 029 to 675 684) | 339.2 (294.1 to 388.5) | 461.1 (399.3 to 528.3) | 593 709 (552 981 to 635 485) | 1 639 241 (1 522 198 to 1 763 199) | 1 002.9 (934.7 to 1 074.6) | 1 325.0 (1 232.2 to 1 425.8) |
| Pedestrian road injuries | 45 145 (36 594 to 55 581) | 83 008 (67 490 to 100 601) | 62.3 (52.0 to 74.1) | 66.0 (53.9 to 79.8) | 162 387 (139 206 to 190 828) | 371 081 (322 198 to 432 250) | 278.2 (241.6 to 323.6) | 303.3 (263.9 to 351.6) |
| Cyclist road injuries | 38 165 (28 927 to 49 950) | 77 639 (61 335 to 97 667) | 46.5 (36.6 to 58.7) | 61.8 (48.9 to 77.5) | 63 056 (53 011 to 74 427) | 168 350 (142 638 to 196 851) | 100.5 (85.3 to 116.3) | 135.7 (115.8 to 157.8) |
| Motorcyclist road injuries | 34 496 (27 123 to 43 509) | 60 596 (46 621 to 76 879) | 42.9 (34.4 to 53.2) | 46.9 (36.2 to 59.4) | 120 905 (104 699 to 139 896) | 274 228 (234 943 to 321 608) | 199.5 (172.8 to 230.4) | 218.5 (187.4 to 255.3) |
| Motor vehicle road injuries | 99 272 (76 134 to 123 364) | 282 745 (236 392 to 335 893) | 125.0 (103.4 to 147.8) | 219.8 (183.6 to 260.1) | 161 042 (141 127 to 186 832) | 629 439 (548 640 to 737 016) | 281.4 (244.5 to 327.4) | 508.1 (442.4 to 588.0) |
| Other road injuries | 47 576 (35 357 to 59 838) | 83 614 (65 207 to 104 071) | 62.4 (48.9 to 76.3) | 66.6 (52.4 to 82.5) | 86 319 (71 809 to 104 203) | 196 144 (164 098 to 236 912) | 143.4 (119.9 to 171.5) | 159.5 (133.6 to 192.7) |
| Other transport injuries | 13 823 (10 217 to 19 226) | 12 927 (9 879 to 16 947) | 16.7 (13.2 to 21.2) | 10.5 (8.0 to 13.7) | 63 284 (52 174 to 75 748) | 66 970 (57 214 to 76 794) | 95.6 (80.4 to 111.4) | 54.5 (46.8 to 62.3) |
| **Unintentional injuries** | **2 847 735 (2 612 316 to 3 106 534)** | **4 633 685 (4 220 100 to 5 081 917)** | **3 867.2 (3 537.6 to 4 217.6)** | **3 825.0 (3 481.9 to 4 199.3)** | **5 441 723 (5 073 749 to 5 866 183)** | **9 524 021 (8 828 088 to 10 422 116)** | **8 755.9 (8 176.6 to 9 448.1)** | **7 829.8 (7 263.0 to 8 566.5)** |
| Falls | 1 264 558 (1 115 643 to 1 433 736) | 2 697 567 (2 389 537 to 3 048 225) | 1 893.6 (1 698.1 to 2 124.4) | 2 276.0 (2 016.2 to 2 565.6) | 2 207 527 (1 949 640 to 2 524 046) | 5 357 266 (4 779 128 to 6 112 974) | 3 809.4 (3 403.4 to 4 352.4) | 4 481.6 (4 002.3 to 5 130.5) |
| Drowning | 4 618 (4 028 to 5 292) | 2 913 (2 374 to 3 628) | 5.6 (4.9 to 6.3) | 2.3 (1.9 to 2.9) | 20 850 (18 363 to 24 051) | 17 887 (15 572 to 20 782) | 34.9 (30.9 to 39.8) | 14.7 (13.0 to 17.0) |
| Fire, heat, and hot substances | 112 674 (90 272 to 140 255) | 103 991 (85 433 to 126 800) | 116.2 (95.4 to 140.9) | 84.4 (69.3 to 103.0) | 1 081 686 (898 974 to 1 358 357) | 1 257 156 (1 042 974 to 1 588 421) | 1 536.0 (1 294.8 to 1 878.7) | 1 002.5 (832.1 to 1 262.4) |
| Poisonings | 35 835 (27 017 to 47 682) | 39 088 (28 916 to 51 860) | 33.9 (25.9 to 44.2) | 32.1 (23.7 to 42.6) | 31 111 (23 715 to 38 281) | 46 348 (34 388 to 57 939) | 39.2 (30.2 to 48.0) | 36.5 (27.1 to 45.6) |
| Poisoning by carbon monoxide | 6 855 (4 454 to 10 378) | 5 926 (3 487 to 9 393) | 7.1 (4.9 to 10.1) | 4.8 (2.9 to 7.6) | 4 667 (3 582 to 6 046) | 4 641 (3 595 to 5 909) | 6.1 (4.7 to 7.8) | 3.7 (2.9 to 4.7) |
| Poisoning by other means | 28 980 (21 311 to 39 307) | 33 162 (23 829 to 44 850) | 26.7 (19.9 to 35.8) | 27.2 (19.5 to 36.9) | 26 445 (19 226 to 33 291) | 41 706 (29 806 to 52 975) | 33.1 (24.4 to 41.6) | 32.8 (23.4 to 41.6) |
| Exposure to mechanical forces | 663 372 (563 355 to 783 125) | 744 235 (640 356 to 867 053) | 801.7 (688.8 to 932.7) | 583.4 (502.4 to 680.2) | 1 262 293 (1 124 833 to 1 474 416) | 1 846 909 (1 615 171 to 2 136 885) | 2 093.7 (1 847.7 to 2 385.3) | 1 483.7 (1 297.0 to 1 713.4) |
| Unintentional firearm injuries | 52 438 (38 805 to 69 722) | 38 538 (28 262 to 51 393) | 62.0 (46.7 to 80.5) | 30.0 (22.0 to 39.9) | 186 359 (148 503 to 222 877) | 188 888 (152 824 to 227 168) | 310.5 (249.4 to 373.6) | 152.4 (123.8 to 183.8) |
| Other exposure to mechanical forces | 610 934 (518 070 to 725 584) | 705 697 (606 392 to 826 331) | 739.7 (634.9 to 863.9) | 553.4 (476.7 to 646.2) | 1 095 934 (929 299 to 1 297 060) | 1 657 421 (1 417 759 to 1 956 606) | 1 783.2 (1 523.7 to 2 098.0) | 1 331.2 (1 140.9 to 1 566.7) |
| Adverse effects of medical treatment | 66 103 (55 934 to 76 528) | 140 282 (119 707 to 161 488) | 97.8 (83.9 to 112.1) | 113.7 (97.4 to 130.7) | 5 059 (3 940 to 6 284) | 10 787 (8 244 to 13 320) | 7.5 (5.8 to 9.2) | 8.7 (6.7 to 10.7) |
| Animal contact | 240 071 (199 786 to 293 568) | 238 704 (202 262 to 273 657) | 293.7 (251.4 to 343.1) | 189.6 (163.9 to 220.3) | 250 250 (215 344 to 290 894) | 291 444 (255 531 to 332 893) | 387.9 (339.3 to 441.5) | 237.2 (208.4 to 270.5) |
| Venomous animal contact | 88 938 (68 012 to 115 523) | 85 807 (67 806 to 106 872) | 108.3 (84.5 to 136.7) | 69.4 (54.8 to 86.1) | 59 452 (48 913 to 71 162) | 64 726 (54 443 to 74 996) | 86.4 (72.5 to 101.1) | 52.1 (44.0 to 60.3) |
| Non venomous animal contact | 151 133 (121 134 to 187 816) | 147 898 (123 280 to 179 582) | 185.4 (153.1 to 223.7) | 120.3 (100.3 to 146.4) | 190 798 (157 321 to 230 420) | 226 719 (191 420 to 268 323) | 301.4 (254.2 to 354.1) | 185.1 (156.8 to 217.9) |
| Foreign body | 256 720 (142 760 to 399 461) | 424 291 (197 259 to 703 496) | 372.0 (181.2 to 596.1) | 344.9 (159.8 to 576.2) | 121 174 (84 296 to 159 672) | 154 269 (113 783 to 214 315) | 184.3 (145.4 to 239.7) | 127.4 (94.0 to 175.6) |
| Pulmonary aspiration and foreign body in airway | 8 721 (6 192 to 11 919) | 4 796 (3 507 to 6 492) | 8.7 (6.4 to 11.5) | 4.0 (2.9 to 5.3) | 6 095 (4 330 to 8 333) | 4 327 (3 468 to 5 348) | 7.9 (5.8 to 10.5) | 3.5 (2.8 to 4.3) |
| Foreign body in eyes | 225 674 (113 150 to 370 559) | 402 845 (175 834 to 685 985) | 338.8 (145.8 to 562.8) | 327.3 (141.6 to 560.8) | 44 687 (22 795 to 77 913) | 87 870 (48 628 to 144 049) | 76.9 (43.6 to 123.4) | 73.9 (41.8 to 118.0) |
| Foreign body in other body part | 22 325 (15 270 to 33 973) | 16 649 (12 263 to 21 656) | 24.5 (17.3 to 34.4) | 13.6 (10.0 to 17.7) | 70 392 (56 045 to 89 404) | 62 072 (51 493 to 76 362) | 99.5 (80.8 to 124.5) | 50.0 (41.8 to 61.4) |
| Environmental heat and cold exposure | 39 716 (28 316 to 52 325) | 30 606 (24 575 to 38 448) | 50.5 (39.9 to 62.6) | 25.2 (20.1 to 31.9) | 138 494 (112 656 to 168 289) | 118 562 (101 253 to 138 988) | 211.4 (176.9 to 250.8) | 96.8 (83.2 to 113.5) |
| Exposure to forces of nature | 13 449 (11 194 to 16 436) | 36 247 (30 593 to 43 518) | 15.0 (12.8 to 17.7) | 28.5 (24.1 to 34.3) | 87 188 (61 303 to 121 828) | 104 394 (73 035 to 145 131) | 109.9 (78.8 to 152.3) | 82.4 (57.9 to 114.4) |
| Other unintentional injuries | 150 625 (124 450 to 182 596) | 180 760 (152 762 to 214 427) | 187.4 (158.4 to 221.1) | 144.8 (122.0 to 171.1) | 216 092 (185 904 to 250 589) | 320 175 (278 078 to 367 863) | 341.9 (296.1 to 390.9) | 258.8 (224.4 to 295.7) |

| Cause | Years lived with disability | | | | Disability adjusted life years | | | |
|---|---|---|---|---|---|---|---|---|
| | All-age counts (95% UI) | | Age-standardised rates per 100,000 (95% UI) | | All-age counts (95% UI) | | Age-standardised rates per 100,000 (95% UI) | |
| | 1990 | 2017 | 1990 | 2017 | 1990 | 2017 | 1990 | 2017 |
| **Transport and unintentional injuries** | **267 435 (195 876 to 356 093)** | **482 563 (347 810 to 651 641)** | **426.9 (311.6 to 564.9)** | **396.9 (285.3 to 533.5)** | **2 761 957 (2 676 267 to 2 859 777)** | **2 376 952 (2 224 588 to 2 551 004)** | **3 324.2 (3 203.3 to 3 468.7)** | **1 886.8 (1 742.2 to 2 028.9)** |
| **Transport injuries** | **44 806 (32 812 to 58 973)** | **103 473 (74 934 to 137 916)** | **72.2 (52.6 to 95.2)** | **82.9 (60.0 to 110.5)** | **1 193 039 (1 165 860 to 1 222 893)** | **1 126 288 (1 081 212 to 1 177 016)** | **1 421.9 (1 389.7 to 1 457.8)** | **873.3 (836.5 to 913.4)** |
| Road injuries | 40 754 (29 933 to 53 942) | 99 919 (72 369 to 133 354) | 66.3 (48.5 to 88.1) | 80.0 (58.0 to 106.7) | 1 154 388 (1 128 583 to 1 182 902) | 1 075 935 (1 033 221 to 1 125 009) | 1 375.0 (1 344.0 to 1 409.0) | 834.4 (801.2 to 873.3) |
| Pedestrian road injuries | 11 618 (8 209 to 15 666) | 23 482 (16 618 to 31 987) | 19.1 (13.6 to 25.6) | 19.0 (13.5 to 25.9) | 546 461 (530 047 to 562 438) | 425 693 (409 035 to 446 038) | 659.7 (641.9 to 677.4) | 334.6 (321.7 to 350.4) |
| Cyclist road injuries | 4 387 (3 085 to 5 775) | 10 109 (7 030 to 13 408) | 6.8 (4.8 to 8.8) | 8.1 (5.6 to 10.8) | 30 626 (29 182 to 32 337) | 46 227 (42 759 to 50 383) | 38.1 (36.1 to 40.5) | 36.2 (33.4 to 39.5) |
| Motorcyclist road injuries | 8 393 (5 883 to 11 298) | 16 778 (11 445 to 22 775) | 13.4 (9.3 to 18.2) | 13.3 (9.0 to 18.0) | 170 642 (164 774 to 176 969) | 157 409 (149 555 to 166 830) | 198.7 (192.1 to 206.4) | 119.8 (113.6 to 127.0) |
| Motor vehicle road injuries | 11 453 (8 262 to 15 151) | 39 707 (28 671 to 52 915) | 19.2 (13.9 to 25.2) | 31.7 (23.0 to 42.3) | 371 603 (360 870 to 386 265) | 411 203 (391 704 to 430 353) | 435.9 (422.9 to 452.2) | 316.3 (301.3 to 331.3) |
| Other road injuries | 4 902 (3 495 to 6 629) | 9 842 (7 007 to 13 520) | 7.8 (5.7 to 10.6) | 7.9 (5.6 to 10.9) | 35 056 (31 117 to 37 691) | 35 402 (32 131 to 39 736) | 42.6 (38.0 to 46.1) | 27.7 (25.1 to 31.1) |
| Other transport injuries | 4 052 (2 863 to 5 413) | 3 554 (2 538 to 4 792) | 5.8 (4.2 to 7.7) | 2.9 (2.1 to 3.9) | 38 651 (36 950 to 40 389) | 50 353 (47 043 to 52 663) | 47.0 (44.9 to 49.2) | 38.9 (36.4 to 40.7) |
| **Unintentional injuries** | **222 629 (161 899 to 296 629)** | **379 091 (271 459 to 513 959)** | **354.7 (255.9 to 473.1)** | **313.0 (224.2 to 423.1)** | **1 568 918 (1 502 683 to 1 648 025)** | **1 250 664 (1 136 563 to 1 383 438)** | **1 902.3 (1 802.9 to 2 022.1)** | **1 013.4 (918.4 to 1 123.2)** |
| Falls | 130 767 (95 912 to 171 006) | 272 504 (195 339 to 361 744) | 219.1 (159.8 to 284.7) | 227.5 (162.5 to 301.7) | 308 834 (275 107 to 348 580) | 445 752 (368 909 to 534 652) | 490.5 (433.2 to 557.6) | 370.9 (307.6 to 446.0) |
| Drowning | 1 187 (833 to 1 628) | 940 (644 to 1 308) | 1.9 (1.4 to 2.6) | 0.8 (0.5 to 1.1) | 348 037 (337 936 to 357 900) | 174 266 (169 124 to 180 062) | 370.4 (361.0 to 378.7) | 136.8 (132.8 to 141.5) |
| Fire, heat, and hot substances | 23 286 (14 508 to 36 422) | 24 266 (14 480 to 38 212) | 32.0 (19.9 to 50.2) | 19.3 (11.5 to 31.1) | 124 004 (114 467 to 137 735) | 69 336 (59 036 to 84 267) | 146.6 (134.1 to 164.9) | 56.0 (47.8 to 67.8) |
| Poisonings | 3 671 (2 276 to 5 442) | 5 352 (3 271 to 8 087) | 4.5 (2.8 to 6.6) | 4.2 (2.6 to 6.3) | 70 941 (67 945 to 73 882) | 40 100 (37 457 to 42 991) | 76.1 (73.1 to 79.1) | 31.6 (29.6 to 33.9) |
| Poisoning by carbon monoxide | 502 (307 to 775) | 457 (278 to 720) | 0.6 (0.4 to 0.9) | 0.4 (0.2 to 0.6) | 24 948 (23 975 to 25 924) | 14 887 (14 116 to 15 546) | 27.2 (26.2 to 28.3) | 11.7 (11.1 to 12.2) |
| Poisoning by other means | 3 169 (1 946 to 4 829) | 4 895 (2 954 to 7 515) | 3.9 (2.4 to 5.9) | 3.8 (2.3 to 5.9) | 45 993 (43 734 to 48 197) | 25 213 (22 985 to 27 789) | 48.8 (46.5 to 51.4) | 20.0 (18.2 to 22.0) |
| Exposure to mechanical forces | 30 272 (20 830 to 43 188) | 39 885 (26 973 to 57 999) | 48.4 (33.3 to 69.3) | 32.0 (21.6 to 46.5) | 222 773 (211 929 to 237 297) | 133 671 (120 463 to 152 308) | 276.9 (260.7 to 300.4) | 104.9 (94.9 to 119.7) |
| Unintentional firearm injuries | 3 909 (2 583 to 5 901) | 3 529 (2 284 to 5 539) | 6.3 (4.2 to 9.6) | 2.8 (1.8 to 4.4) | 102 774 (97 925 to 110 164) | 40 790 (33 441 to 43 922) | 120.9 (115.2 to 130.4) | 31.4 (25.8 to 33.9) |
| Other exposure to mechanical forces | 26 363 (18 200 to 37 740) | 36 356 (24 476 to 53 162) | 42.1 (29.0 to 60.4) | 29.1 (19.7 to 42.6) | 119 999 (108 659 to 133 016) | 92 881 (80 576 to 110 859) | 156.1 (140.1 to 175.9) | 73.4 (63.6 to 87.6) |
| Adverse effects of medical treatment | 674 (413 to 1 021) | 1 432 (885 to 2 159) | 1.0 (0.6 to 1.5) | 1.2 (0.7 to 1.7) | 56 638 (48 646 to 61 153) | 51 842 (48 342 to 70 244) | 70.3 (61.4 to 76.0) | 43.1 (40.2 to 58.2) |
| Animal contact | 6 700 (4 474 to 9 396) | 6 693 (4 497 to 9 623) | 9.4 (6.3 to 13.2) | 5.4 (3.6 to 7.7) | 70 661 (64 977 to 76 566) | 24 093 (21 563 to 27 155) | 71.7 (66.1 to 77.7) | 19.6 (17.5 to 22.1) |
| Venomous animal contact | 3 757 (2 416 to 5 428) | 3 490 (2 294 to 5 072) | 4.8 (3.2 to 6.9) | 2.8 (1.8 to 4.0) | 58 294 (52 991 to 63 641) | 15 569 (13 940 to 17 315) | 56.6 (51.1 to 61.5) | 12.7 (11.4 to 14.1) |
| Non venomous animal contact | 2 943 (1 777 to 4 731) | 3 203 (1 891 to 5 168) | 4.6 (2.8 to 7.3) | 2.6 (1.6 to 4.2) | 12 367 (9 939 to 15 491) | 8 524 (7 088 to 10 822) | 15.1 (12.1 to 19.1) | 6.9 (5.7 to 8.8) |
| Foreign body | 7 575 (5 020 to 11 110) | 8 958 (5 475 to 14 399) | 11.3 (7.5 to 16.4) | 7.4 (4.5 to 11.8) | 251 190 (240 597 to 261 070) | 211 136 (200 212 to 226 940) | 258.3 (248.7 to 268.1) | 172.0 (162.9 to 185.1) |
| Pulmonary aspiration and foreign body in airway | 326 (219 to 449) | 183 (127 to 252) | 0.4 (0.3 to 0.5) | 0.1 (0.1 to 0.2) | 232 964 (222 669 to 242 296) | 197 480 (187 934 to 211 326) | 235.8 (227.0 to 243.9) | 160.9 (153.0 to 172.6) |
| Foreign body in eyes | 2 972 (1 508 to 5 368) | 5 858 (3 115 to 10 587) | 5.2 (2.8 to 9.1) | 4.9 (2.6 to 8.7) | 2 972 (1 508 to 5 368) | 5 858 (3 115 to 10 587) | 5.2 (2.8 to 9.1) | 4.9 (2.6 to 8.7) |
| Foreign body in other body part | 4 277 (2 934 to 5 676) | 2 907 (2 079 to 3 841) | 5.7 (4.0 to 7.6) | 2.3 (1.7 to 3.1) | 15 254 (12 864 to 17 065) | 7 788 (6 676 to 10 752) | 17.3 (14.4 to 19.4) | 6.1 (5.2 to 8.5) |
| Environmental heat and cold exposure | 5 438 (3 592 to 7 686) | 4 456 (2 928 to 6 407) | 8.2 (5.6 to 11.6) | 3.6 (2.4 to 5.2) | 28 987 (26 936 to 31 394) | 14 786 (13 035 to 15 880) | 39.6 (36.6 to 43.2) | 11.8 (10.4 to 13.6) |
| Exposure to forces of nature | 4 587 (3 271 to 6 312) | 3 915 (2 853 to 5 319) | 5.7 (4.1 to 7.8) | 3.1 (2.3 to 4.1) | 13 004 (11 546 to 14 938) | 31 163 (29 440 to 33 205) | 15.1 (13.5 to 17.3) | 24.5 (23.2 to 26.1) |
| Other unintentional injuries | 8 472 (6 101 to 11 294) | 10 688 (7 517 to 14 684) | 13.1 (9.4 to 17.6) | 8.6 (6.1 to 11.9) | 73 850 (70 982 to 77 108) | 54 520 (50 973 to 59 018) | 86.8 (82.7 to 91.4) | 42.2 (39.4 to 45.8) |
