# Supplementary material for: Unintentional injuries in Mexico, 1990–2017: findings from the Global Burden of Disease Study 2017
Source: Inj Prev. 2020 Apr 1;26(Suppl 1):i154–61. doi: 10.1136/injuryprev-2019-043532 (PMC7571365; doi:10.1136/injuryprev-2019-043532)
Supplement: Supplementary data [file injuryprev-2019-043532supp004.pdf]

Death, YLL, incidence, prevalence, YLD, and DALY counts and rates per 100,000 for all injuries in Aguascalientes in 1990 and 2017

Cause	Deaths: All-age counts (95% UI) 1990	Deaths: All-age counts (95% UI) 2017	Deaths: Age-standardised rates per 100,000 (95% UI) 1990	Deaths: Age-standardised rates per 100,000 (95% UI) 2017
**Transport and unintentional injuries**	**414 (396 to 435)**	**480 (449 to 514)**	**73.4 (71.1 to 75.9)**	**38.7 (36.3 to 41.3)**
**Transport injuries**	**199 (189 to 210)**	**257 (237 to 279)**	**32.3 (31.0 to 33.8)**	**19.1 (17.7 to 20.7)**
Road injuries	194 (184 to 205)	246 (227 to 268)	31.4 (30.1 to 32.9)	18.4 (17.0 to 19.9)
Pedestrian road injuries	91 (85 to 97)	103 (95 to 112)	16.1 (15.4 to 17.0)	8.2 (7.5 to 8.8)
Cyclist road injuries	7 (7 to 8)	16 (14 to 18)	1.2 (1.1 to 1.3)	1.2 (1.0 to 1.3)
Motorcyclist road injuries	29 (26 to 32)	33 (29 to 38)	4.1 (3.7 to 4.4)	2.3 (2.0 to 2.6)
Motor vehicle road injuries	62 (57 to 68)	88 (79 to 98)	9.3 (8.6 to 10.0)	6.3 (5.7 to 7.0)
Other road injuries	5 (4 to 5)	6 (5 to 7)	0.8 (0.7 to 0.9)	0.5 (0.4 to 0.5)
Other transport injuries	5 (5 to 6)	11 (9 to 12)	0.9 (0.8 to 1.0)	0.8 (0.7 to 0.9)
**Unintentional injuries**	**215 (203 to 228)**	**223 (209 to 239)**	**41.0 (39.6 to 42.6)**	**19.5 (18.3 to 20.8)**
Falls	53 (51 to 56)	78 (72 to 84)	15.2 (14.3 to 16.0)	7.8 (7.2 to 8.4)
Drowning	46 (42 to 51)	33 (30 to 36)	6.5 (6.1 to 7.0)	2.4 (2.2 to 2.7)
Fire, heat, and hot substances	15 (14 to 16)	13 (12 to 14)	3.1 (2.9 to 3.2)	1.2 (1.1 to 1.2)
Poisonings	10 (9 to 11)	8 (7 to 9)	1.4 (1.3 to 1.6)	0.6 (0.6 to 0.7)
Poisoning by carbon monoxide	4 (4 to 5)	4 (4 to 4)	0.6 (0.6 to 0.7)	0.3 (0.3 to 0.3)
Poisoning by other means	5 (5 to 6)	4 (4 to 5)	0.8 (0.7 to 0.9)	0.3 (0.3 to 0.3)
Exposure to mechanical forces	20 (19 to 22)	15 (14 to 17)	3.4 (3.2 to 3.6)	1.2 (1.1 to 1.3)
Unintentional firearm injuries	7 (6 to 9)	4 (3 to 5)	1.1 (1.0 to 1.4)	0.3 (0.2 to 0.3)
Other exposure to mechanical forces	13 (11 to 14)	11 (10 to 13)	2.3 (2.0 to 2.5)	0.9 (0.8 to 1.0)
Adverse effects of medical treatment	13 (10 to 14)	19 (17 to 21)	3.0 (2.4 to 3.2)	1.7 (1.6 to 2.0)
Animal contact	3 (3 to 4)	2 (2 to 2)	0.5 (0.4 to 0.5)	0.2 (0.1 to 0.2)
Venomous animal contact	2 (2 to 2)	1 (1 to 1)	0.3 (0.2 to 0.3)	0.1 (0.1 to 0.1)
Non-venomous animal contact	1 (1 to 1)	1 (1 to 1)	0.2 (0.2 to 0.2)	0.1 (0.1 to 0.1)
Foreign body	43 (38 to 49)	46 (41 to 52)	6.0 (5.5 to 6.6)	3.6 (3.3 to 4.1)
Pulmonary aspiration and foreign body in airway	42 (37 to 48)	45 (40 to 51)	5.9 (5.4 to 6.4)	3.6 (3.2 to 4.0)
Foreign body in eyes				
Foreign body in other body part	1 (1 to 2)	1 (1 to 2)	0.2 (0.1 to 0.2)	0.1 (0.0 to 0.1)
Environmental heat and cold exposure	3 (3 to 3)	2 (2 to 2)	0.7 (0.7 to 0.8)	0.2 (0.2 to 0.2)
Exposure to forces of nature	0 (0 to 0)	0 (0 to 0)	0.1 (0.1 to 0.1)	0.0 (0.0 to 0.0)
Other unintentional injuries	8 (7 to 9)	8 (7 to 9)	1.2 (1.1 to 1.3)	0.6 (0.5 to 0.6)

Cause	Years of life lost: All-age counts (95% UI) 1990	Years of life lost: All-age counts (95% UI) 2017	Years of life lost: Age-standardised rates per 100,000 (95% UI) 1990	Years of life lost: Age-standardised rates per 100,000 (95% UI) 2017
**Transport and unintentional injuries**	**22 564 (21 090 to 24 213)**	**21 632 (20 010 to 23 361)**	**3 015.8 (2 876.5 to 3 181.0)**	**1 556.6 (1 441.7 to 1 680.5)**
**Transport injuries**	**11 128 (10 471 to 11 860)**	**12 635 (11 604 to 13 752)**	**1 500.1 (1 424.1 to 1 588.1)**	**883.2 (811.9 to 959.2)**
Road injuries	10 840 (10 208 to 11 558)	12 119 (11 124 to 13 187)	1 460.7 (1 386.9 to 1 547.6)	847.2 (778.1 to 920.2)
Pedestrian road injuries	4 852 (4 482 to 5 321)	4 589 (4 166 to 5 094)	666.1 (622.9 to 715.6)	329.7 (300.0 to 360.8)
Cyclist road injuries	412 (365 to 463)	729 (646 to 831)	57.3 (51.5 to 63.8)	51.5 (45.8 to 58.6)
Motorcyclist road injuries	1 701 (1 518 to 1 902)	1 886 (1 645 to 2 183)	222.1 (201.9 to 245.4)	126.9 (111.0 to 146.5)
Motor vehicle road injuries	3 610 (3 301 to 3 988)	4 614 (4 124 to 5 152)	480.0 (441.2 to 523.2)	317.6 (282.9 to 353.9)
Other road injuries	266 (223 to 311)	307 (262 to 360)	35.2 (30.0 to 40.8)	21.4 (18.4 to 24.9)
Other transport injuries	288 (258 to 323)	515 (451 to 585)	39.5 (35.8 to 43.6)	36.0 (31.7 to 40.8)
**Unintentional injuries**	**11 435 (10 458 to 12 541)**	**8 998 (8 253 to 9 841)**	**1 515.7 (1 421.2 to 1 609.4)**	**673.4 (618.2 to 735.2)**
Falls	1 663 (1 563 to 1 774)	1 903 (1 747 to 2 072)	312.9 (297.9 to 328.3)	156.7 (144.5 to 169.7)
Drowning	2 912 (2 580 to 3 299)	1 758 (1 566 to 1 961)	350.3 (318.6 to 386.5)	123.6 (110.7 to 138.0)
Fire, heat, and hot substances	770 (673 to 891)	442 (399 to 487)	103.2 (93.8 to 114.8)	33.8 (30.7 to 37.3)
Poisonings	606 (539 to 684)	405 (363 to 447)	73.8 (66.8 to 81.7)	28.9 (26.0 to 31.9)
Poisoning by carbon monoxide	255 (225 to 289)	201 (177 to 223)	31.9 (28.8 to 35.5)	14.2 (12.6 to 15.8)
Poisoning by other means	351 (306 to 401)	205 (181 to 230)	41.9 (37.5 to 46.9)	14.7 (13.0 to 16.5)
Exposure to mechanical forces	1 104 (1 008 to 1 201)	707 (639 to 786)	153.5 (142.1 to 165.2)	50.1 (45.5 to 55.5)
Unintentional firearm injuries	401 (350 to 506)	199 (165 to 234)	53.8 (47.4 to 68.0)	13.8 (11.4 to 16.1)
Other exposure to mechanical forces	703 (598 to 781)	508 (456 to 566)	99.8 (85.2 to 109.4)	36.3 (32.8 to 40.4)
Adverse effects of medical treatment	560 (450 to 637)	598 (528 to 732)	82.3 (67.3 to 90.7)	47.4 (42.1 to 57.5)
Animal contact	202 (167 to 242)	83 (72 to 92)	23.1 (19.8 to 27.1)	6.1 (5.4 to 6.9)
Venomous animal contact	145 (114 to 181)	42 (32 to 52)	15.7 (12.7 to 19.2)	3.2 (2.4 to 3.9)
Non-venomous animal contact	58 (49 to 67)	39 (34 to 53)	7.4 (6.5 to 8.4)	2.9 (2.5 to 3.3)
Foreign body	2 997 (2 547 to 3 488)	2 612 (2 229 to 3 080)	331.3 (289.6 to 378.2)	192.2 (164.0 to 226.6)
Pulmonary aspiration and foreign body in airway	2 918 (2 477 to 3 408)	2 567 (2 194 to 3 031)	321.8 (280.7 to 368.3)	189.1 (161.5 to 223.3)
Foreign body in eyes				
Foreign body in other body part	79 (66 to 93)	45 (36 to 56)	9.5 (8.1 to 12.0)	3.1 (2.5 to 6.0)
Environmental heat and cold exposure	144 (129 to 158)	78 (69 to 86)	22.6 (20.6 to 24.3)	5.8 (5.2 to 6.5)
Exposure to forces of nature	25 (22 to 28)	11 (10 to 12)	3.1 (2.9 to 3.4)	0.8 (0.7 to 0.9)
Other unintentional injuries	453 (411 to 505)	406 (358 to 466)	59.5 (54.4 to 65.6)	28.0 (24.8 to 32.0)

Cause	Incidence: All-age counts (95% UI) 1990	Incidence: All-age counts (95% UI) 2017	Incidence: Age-standardised rates per 100,000 (95% UI) 1990	Incidence: Age-standardised rates per 100,000 (95% UI) 2017
**Transport and unintentional injuries**	**29 229 (26 817 to 31 926)**	**59 872 (54 524 to 66 008)**	**4 702.1 (4 313.1 to 5 141.5)**	**4 692.5 (4 281.9 to 5 150.9)**
**Transport injuries**	**2 868 (2 466 to 3 336)**	**7 807 (6 794 to 8 925)**	**432.9 (378.3 to 493.1)**	**561.8 (490.4 to 637.8)**
Road injuries	2 746 (2 341 to 3 205)	7 655 (6 656 to 8 762)	415.7 (362.2 to 474.2)	550.3 (479.4 to 627.0)
Pedestrian road injuries	445 (360 to 544)	1 013 (820 to 1 238)	71.0 (58.9 to 84.4)	74.8 (60.9 to 89.0)
Cyclist road injuries	534 (420 to 678)	1 372 (1 107 to 1 685)	75.1 (60.4 to 93.0)	99.4 (80.6 to 120.7)
Motorcyclist road injuries	346 (273 to 429)	760 (595 to 953)	49.6 (39.6 to 61.2)	53.1 (41.6 to 66.2)
Motor vehicle road injuries	973 (799 to 1 164)	3 503 (2 935 to 4 150)	152.1 (125.8 to 179.3)	249.1 (210.2 to 293.8)
Other road injuries	447 (338 to 563)	1 007 (794 to 1 235)	67.9 (54.2 to 82.3)	73.8 (58.9 to 90.1)
Other transport injuries	123 (93 to 162)	151 (124 to 186)	17.2 (14.0 to 21.5)	11.5 (9.5 to 14.1)
**Unintentional injuries**	**26 360 (23 970 to 29 061)**	**52 065 (46 712 to 58 096)**	**4 269.2 (3 874.9 to 4 699.8)**	**4 130.6 (3 720.7 to 4 592.6)**
Falls	13 249 (11 624 to 15 067)	32 965 (29 395 to 37 248)	2 356.3 (2 104.9 to 2 643.5)	2 705.9 (2 406.1 to 3 048.3)
Drowning	43 (37 to 51)	32 (26 to 41)	6.0 (5.2 to 6.9)	2.4 (1.9 to 3.0)
Fire, heat, and hot substances	961 (759 to 1 214)	912 (718 to 1 171)	112.1 (91.4 to 137.4)	66.9 (52.8 to 85.5)
Poisonings	373 (277 to 492)	451 (329 to 606)	39.2 (30.0 to 50.5)	32.8 (24.0 to 44.0)
Poisoning by carbon monoxide	74 (48 to 112)	91 (49 to 150)	8.7 (6.1 to 12.4)	6.7 (3.7 to 10.9)
Poisoning by other means	297 (218 to 405)	360 (251 to 507)	30.5 (22.9 to 40.8)	26.1 (18.2 to 36.8)
Exposure to mechanical forces	5 234 (4 382 to 6 269)	7 046 (5 981 to 8 319)	730.5 (623.3 to 861.7)	502.2 (427.0 to 589.9)
Unintentional firearm injuries	329 (233 to 456)	220 (145 to 318)	43.6 (31.8 to 58.2)	15.8 (10.5 to 22.9)
Other exposure to mechanical forces	4 905 (4 111 to 5 890)	6 826 (5 790 to 8 037)	687.0 (586.5 to 812.9)	486.4 (415.3 to 570.6)
Adverse effects of medical treatment	682 (578 to 788)	1 772 (1 496 to 2 042)	118.9 (102.9 to 135.6)	137.1 (116.3 to 157.9)
Animal contact	1 545 (1 212 to 1 970)	1 684 (1 414 to 2 029)	215.6 (175.5 to 261.6)	127.3 (106.9 to 151.9)
Venomous animal contact	539 (384 to 742)	515 (390 to 665)	74.8 (55.6 to 99.6)	38.9 (29.8 to 50.0)
Non-venomous animal contact	1 006 (761 to 1 306)	1 169 (949 to 1 449)	140.8 (110.5 to 175.8)	88.3 (72.3 to 109.0)
Foreign body	2 645 (1 424 to 4 207)	5 480 (2 937 to 9 720)	457.1 (206.9 to 755.0)	426.2 (176.8 to 739.1)
Pulmonary aspiration and foreign body in airway	113 (81 to 152)	64 (47 to 84)	12.4 (9.3 to 16.3)	4.8 (3.6 to 6.3)
Foreign body in eyes	2 313 (1 098 to 3 862)	5 222 (2 077 to 9 431)	418.1 (165.2 to 712.3)	406.6 (156.0 to 718.7)
Foreign body in other body part	219 (148 to 328)	195 (141 to 257)	26.5 (18.8 to 37.0)	14.7 (10.7 to 19.2)
Environmental heat and cold exposure	313 (219 to 425)	237 (174 to 327)	46.2 (35.6 to 58.1)	18.8 (14.0 to 25.4)
Exposure to forces of nature	65 (53 to 81)	15 (13 to 18)	8.0 (6.7 to 9.6)	1.1 (0.9 to 1.3)
Other unintentional injuries	1 252 (1 006 to 1 545)	1 470 (1 189 to 1 817)	179.3 (149.3 to 216.3)	110.0 (88.8 to 135.8)

Cause	Prevalence: All-age counts (95% UI) 1990	Prevalence: All-age counts (95% UI) 2017	Prevalence: Age-standardised rates per 100,000 (95% UI) 1990	Prevalence: Age-standardised rates per 100,000 (95% UI) 2017
**Transport and unintentional injuries**	**53 610 (50 146 to 57 769)**	**119 701 (111 490 to 129 427)**	**10 416.5 (9 768.9 to 11 227.2)**	**9 563.8 (8 940.3 to 10 361.3)**
**Transport injuries**	**6 600 (6 142 to 7 019)**	**21 032 (19 545 to 22 592)**	**1 317.3 (1 228.2 to 1 405.5)**	**1 636.0 (1 520.8 to 1 760.1)**
Road injuries	6 066 (5 636 to 6 489)	20 290 (18 834 to 21 813)	1 222.4 (1 139.1 to 1 308.2)	1 577.7 (1 463.7 to 1 698.0)
Pedestrian road injuries	1 570 (1 346 to 1 838)	4 378 (3 787 to 5 117)	320.3 (279.7 to 372.1)	346.8 (301.7 to 403.0)
Cyclist road injuries	866 (731 to 1 023)	2 871 (2 425 to 3 357)	164.8 (140.3 to 191.2)	221.3 (188.4 to 257.5)
Motorcyclist road injuries	1 178 (1 021 to 1 358)	3 297 (2 761 to 3 812)	232.9 (200.9 to 267.2)	246.6 (210.9 to 289.4)
Motor vehicle road injuries	1 651 (1 442 to 1 929)	7 508 (6 540 to 8 809)	347.0 (302.3 to 402.5)	583.5 (507.0 to 683.0)
Other road injuries	800 (665 to 958)	2 236 (1 914 to 2 794)	157.9 (132.2 to 189.3)	179.5 (150.4 to 216.5)
Other transport injuries	534 (441 to 645)	742 (631 to 855)	94.9 (80.2 to 110.9)	58.3 (50.1 to 66.7)
**Unintentional injuries**	**47 010 (43 629 to 51 082)**	**98 669 (90 800 to 108 637)**	**9 099.2 (8 461.6 to 9 889.8)**	**7 927.8 (7 304.4 to 8 745.7)**
Falls	22 226 (19 627 to 25 394)	62 780 (55 852 to 71 417)	4 606.2 (4 108.5 to 5 264.2)	5 186.1 (4 645.5 to 5 930.3)
Drowning	188 (164 to 217)	187 (163 to 217)	37.1 (32.8 to 42.4)	14.9 (13.1 to 17.2)
Fire, heat, and hot substances	8 727 (7 200 to 10 949)	10 421 (8 500 to 13 479)	1 460.8 (1 223.8 to 1 780.8)	782.1 (640.2 to 998.0)
Poisonings	308 (236 to 374)	505 (374 to 627)	44.7 (34.8 to 54.1)	36.5 (27.1 to 45.2)
Poisoning by carbon monoxide	43 (37 to 63)	71 (54 to 92)	7.3 (5.7 to 9.4)	5.2 (4.0 to 6.7)
Poisoning by other means	259 (190 to 325)	434 (309 to 556)	37.4 (27.8 to 46.8)	31.3 (22.3 to 40.0)
Exposure to mechanical forces	9 763 (8 521 to 11 227)	16 828 (14 522 to 19 662)	1 899.6 (1 662.8 to 2 178.8)	1 286.5 (1 114.0 to 1 497.5)
Unintentional firearm injuries	1 183 (938 to 1 408)	1 101 (885 to 1 307)	230.9 (185.4 to 276.1)	86.1 (70.0 to 101.6)
Other exposure to mechanical forces	8 580 (7 237 to 10 151)	15 727 (13 377 to 18 600)	1 668.7 (1 429.2 to 1 958.1)	1 200.4 (1 025.7 to 1 416.7)
Adverse effects of medical treatment	52 (41 to 65)	136 (104 to 167)	9.1 (7.1 to 11.1)	10.5 (8.1 to 12.9)
Animal contact	1 571 (1 343 to 1 841)	2 098 (1 794 to 2 462)	286.6 (249.9 to 327.3)	162.3 (139.3 to 189.0)
Venomous animal contact	344 (282 to 405)	350 (299 to 426)	58.3 (48.7 to 67.9)	27.2 (22.9 to 32.1)
Non-venomous animal contact	1 227 (1 013 to 1 479)	1 738 (1 442 to 2 089)	228.3 (192.0 to 268.2)	135.0 (112.3 to 161.4)
Foreign body	1 199 (920 to 1 601)	1 863 (1 324 to 2 692)	214.9 (166.7 to 285.7)	149.9 (107.7 to 212.2)
Pulmonary aspiration and foreign body in airway	75 (52 to 103)	55 (45 to 68)	11.3 (8.1 to 15.0)	4.2 (3.4 to 5.1)
Foreign body in eyes	455 (227 to 794)	1 108 (595 to 1 931)	95.3 (54.0 to 155.9)	92.2 (51.6 to 152.0)
Foreign body in other body part	667 (525 to 860)	700 (580 to 866)	108.3 (87.4 to 136.2)	53.5 (44.6 to 65.7)
Environmental heat and cold exposure	1 064 (854 to 1 316)	897 (753 to 1 077)	191.2 (158.6 to 228.3)	71.6 (60.7 to 85.2)
Exposure to forces of nature	178 (125 to 248)	487 (338 to 675)	23.9 (17.3 to 32.5)	35.6 (24.8 to 49.2)
Other unintentional injuries	1 786 (1 483 to 2 031)	2 478 (2 127 to 2 850)	326.0 (282.0 to 372.7)	193.2 (166.5 to 219.7)

Cause	Years lived with disability: All-age counts (95% UI) 1990	Years lived with disability: All-age counts (95% UI) 2017	Years lived with disability: Age-standardised rates per 100,000 (95% UI) 1990	Years lived with disability: Age-standardised rates per 100,000 (95% UI) 2017
**Transport and unintentional injuries**	**2 458 (1 784 to 3 248)**	**5 437 (3 953 to 7 252)**	**471.5 (343.5 to 624.4)**	**434.2 (315.4 to 578.9)**
**Transport injuries**	**449 (329 to 592)**	**1 286 (934 to 1 709)**	**86.0 (62.9 to 112.6)**	**98.4 (71.4 to 130.7)**
Road injuries	415 (304 to 548)	1 246 (905 to 1 655)	80.3 (58.8 to 105.9)	95.4 (69.2 to 126.7)
Pedestrian road injuries	112 (79 to 151)	280 (198 to 381)	21.9 (15.7 to 29.4)	21.9 (15.5 to 29.7)
Cyclist road injuries	60 (42 to 79)	178 (120 to 240)	10.9 (7.8 to 14.4)	13.3 (9.2 to 17.5)
Motorcyclist road injuries	81 (57 to 111)	199 (137 to 272)	15.5 (10.9 to 21.1)	15.0 (10.3 to 20.5)
Motor vehicle road injuries	117 (84 to 155)	478 (347 to 635)	23.5 (17.0 to 31.1)	36.5 (26.6 to 48.6)
Other road injuries	45 (32 to 61)	115 (82 to 159)	8.5 (6.2 to 11.5)	8.9 (6.3 to 12.2)
Other transport injuries	34 (24 to 45)	40 (28 to 53)	5.7 (4.1 to 7.5)	3.1 (2.2 to 4.1)
**Unintentional injuries**	**2 010 (1 461 to 2 653)**	**4 151 (2 982 to 5 586)**	**385.5 (278.7 to 512.7)**	**335.8 (241.2 to 450.0)**
Falls	1 306 (956 to 1 705)	3 209 (2 322 to 4 255)	262.5 (191.5 to 342.3)	264.0 (190.9 to 350.4)
Drowning	11 (7 to 15)	10 (7 to 14)	2.0 (1.4 to 2.8)	0.8 (0.5 to 1.1)
Fire, heat, and hot substances	187 (116 to 289)	202 (117 to 328)	30.2 (18.6 to 46.9)	15.0 (8.8 to 24.3)
Poisonings	36 (22 to 55)	58 (34 to 88)	5.1 (3.2 to 7.6)	4.1 (2.5 to 6.3)
Poisoning by carbon monoxide	5 (3 to 8)	7 (4 to 11)	0.7 (0.5 to 1.2)	0.5 (0.3 to 0.8)
Poisoning by other means	31 (19 to 47)	51 (30 to 77)	4.3 (2.6 to 6.5)	3.6 (2.1 to 5.5)
Exposure to mechanical forces	229 (157 to 327)	361 (249 to 527)	43.6 (29.8 to 62.1)	27.5 (18.5 to 40.0)
Unintentional firearm injuries	24 (16 to 37)	20 (13 to 32)	4.5 (3.0 to 7.0)	1.6 (1.0 to 2.5)
Other exposure to mechanical forces	205 (141 to 292)	341 (228 to 500)	39.0 (26.8 to 55.9)	25.9 (17.4 to 38.0)
Adverse effects of medical treatment	7 (4 to 11)	18 (11 to 27)	1.2 (0.8 to 1.8)	1.4 (0.9 to 2.1)
Animal contact	42 (28 to 60)	50 (33 to 73)	6.9 (4.6 to 9.8)	3.8 (2.5 to 5.4)
Venomous animal contact	23 (15 to 34)	26 (16 to 38)	3.4 (2.2 to 5.0)	1.9 (1.2 to 2.8)
Non-venomous animal contact	19 (11 to 30)	24 (14 to 40)	3.4 (2.1 to 5.4)	1.9 (1.1 to 3.1)
Foreign body	74 (49 to 110)	109 (66 to 177)	13.1 (8.6 to 19.4)	8.8 (5.3 to 14.2)
Pulmonary aspiration and foreign body in airway	4 (3 to 6)	2 (2 to 3)	0.6 (0.4 to 0.8)	0.2 (0.1 to 0.2)
Foreign body in eyes	30 (15 to 57)	74 (38 to 135)	6.4 (3.4 to 11.3)	6.1 (3.2 to 10.9)
Foreign body in other body part	40 (28 to 54)	33 (23 to 44)	6.2 (4.4 to 8.2)	2.5 (1.8 to 3.3)
Environmental heat and cold exposure	42 (27 to 59)	34 (22 to 49)	7.4 (4.9 to 10.5)	2.7 (1.8 to 4.0)
Exposure to forces of nature	9 (6 to 13)	16 (12 to 23)	1.2 (0.9 to 1.7)	1.2 (0.9 to 1.6)
Other unintentional injuries	67 (48 to 90)	84 (59 to 114)	12.3 (8.9 to 16.6)	6.5 (4.6 to 8.9)

Cause	Disability adjusted life years: All-age counts (95% UI) 1990	Disability adjusted life years: All-age counts (95% UI) 2017	Disability adjusted life years: Age-standardised rates per 100,000 (95% UI) 1990	Disability adjusted life years: Age-standardised rates per 100,000 (95% UI) 2017
**Transport and unintentional injuries**	**25 022 (23 410 to 26 938)**	**27 069 (24 902 to 29 514)**	**3 487.3 (3 281.8 to 3 713.8)**	**1 990.9 (1 830.9 to 2 175.8)**
**Transport injuries**	**11 577 (10 889 to 12 333)**	**13 920 (12 838 to 15 150)**	**1 586.2 (1 504.0 to 1 674.2)**	**981.7 (906.3 to 1 067.4)**
Road injuries	11 255 (10 594 to 12 004)	13 365 (12 345 to 14 555)	1 541.0 (1 461.8 to 1 627.2)	942.6 (870.9 to 1 025.5)
Pedestrian road injuries	4 964 (4 590 to 5 445)	4 864 (4 437 to 5 325)	688.0 (643.3 to 738.2)	351.6 (321.7 to 384.6)
Cyclist road injuries	471 (421 to 524)	903 (797 to 1 019)	68.2 (61.4 to 75.5)	64.7 (57.1 to 72.9)
Motorcyclist road injuries	1 782 (1 598 to 1 981)	2 085 (1 837 to 2 385)	237.6 (215.5 to 260.9)	141.9 (125.2 to 161.9)
Motor vehicle road injuries	3 727 (3 423 to 4 104)	5 093 (4 576 to 5 664)	503.5 (466.4 to 547.3)	354.1 (318.6 to 392.7)
Other road injuries	310 (263 to 361)	422 (366 to 487)	43.7 (37.8 to 50.0)	30.3 (26.3 to 34.9)
Other transport injuries	322 (290 to 360)	555 (490 to 625)	45.2 (41.1 to 49.7)	39.1 (34.7 to 43.9)
**Unintentional injuries**	**13 445 (12 246 to 14 657)**	**13 148 (11 771 to 14 802)**	**1 901.1 (1 751.2 to 2 063.1)**	**1 009.2 (900.0 to 1 137.3)**
Falls	2 969 (2 606 to 3 388)	5 112 (4 207 to 6 183)	575.4 (504.9 to 654.9)	420.7 (344.5 to 507.8)
Drowning	2 922 (2 593 to 3 311)	1 768 (1 577 to 1 971)	352.3 (320.8 to 389.1)	124.4 (111.5 to 138.7)
Fire, heat, and hot substances	956 (829 to 1 110)	643 (550 to 774)	133.4 (117.3 to 153.2)	48.8 (41.8 to 58.6)
Poisonings	643 (574 to 719)	463 (415 to 515)	78.9 (71.8 to 86.8)	33.1 (29.7 to 36.8)
Poisoning by carbon monoxide	260 (230 to 294)	208 (184 to 230)	32.7 (29.5 to 36.1)	14.7 (13.1 to 16.3)
Poisoning by other means	382 (337 to 434)	255 (223 to 293)	46.2 (41.6 to 51.6)	18.3 (16.0 to 21.0)
Exposure to mechanical forces	1 333 (1 214 to 1 461)	1 068 (934 to 1 248)	197.1 (179.5 to 218.8)	77.6 (67.6 to 91.0)
Unintentional firearm injuries	425 (375 to 528)	219 (183 to 255)	58.3 (51.8 to 72.5)	15.3 (12.8 to 17.8)
Other exposure to mechanical forces	907 (782 to 1 020)	849 (722 to 1 014)	138.8 (120.9 to 157.7)	62.3 (52.9 to 74.4)
Adverse effects of medical treatment	567 (457 to 644)	617 (548 to 747)	83.6 (68.4 to 92.1)	48.8 (43.5 to 58.5)
Animal contact	244 (206 to 291)	132 (112 to 156)	29.9 (25.5 to 35.1)	9.9 (8.4 to 11.7)
Venomous animal contact	168 (136 to 207)	68 (55 to 83)	19.1 (15.8 to 23.3)	5.1 (4.1 to 6.2)
Non-venomous animal contact	76 (63 to 92)	64 (52 to 81)	10.8 (9.0 to 13.4)	4.8 (3.9 to 6.1)
Foreign body	3 071 (2 626 to 3 575)	2 721 (2 331 to 3 189)	344.4 (301.9 to 391.1)	201.0 (172.2 to 235.3)
Pulmonary aspiration and foreign body in airway	2 922 (2 483 to 3 413)	2 569 (2 196 to 3 034)	322.4 (281.3 to 368.9)	189.3 (161.7 to 223.5)
Foreign body in eyes	30 (15 to 57)	74 (38 to 135)	6.4 (3.4 to 11.3)	6.1 (3.2 to 10.9)
Foreign body in other body part	119 (102 to 144)	78 (63 to 119)	15.7 (13.4 to 18.8)	5.6 (4.5 to 8.6)
Environmental heat and cold exposure	185 (164 to 209)	112 (97 to 130)	30.0 (26.8 to 33.5)	8.5 (7.4 to 9.9)
Exposure to forces of nature	34 (30 to 39)	28 (23 to 34)	4.4 (3.9 to 4.9)	2.0 (1.7 to 2.5)
Other unintentional injuries	520 (471 to 574)	490 (433 to 556)	71.8 (65.6 to 78.5)	34.4 (30.5 to 39.0)

Death, YLL, incidence, prevalence, YLD, and DALY counts and rates per 100,000 for all injuries in Baja California Sur in 1990 and 2017

Cause	Deaths			
	All-age counts (95% UI)		Age-standardised rates per 100,000 (95% UI)	
	1990	2017	1990	2017
**Transport and unintentional injuries**	**165 (159 to 171)**	**256 (237 to 274)**	**66.5 (64.3 to 68.5)**	**36.0 (33.5 to 38.3)**
**Transport injuries**	**96 (92 to 101)**	**155 (141 to 169)**	**35.0 (33.5 to 36.5)**	**19.8 (18.2 to 21.4)**
Road injuries	94 (89 to 98)	147 (135 to 160)	33.9 (32.5 to 35.3)	18.8 (17.3 to 20.3)
Pedestrian road injuries	30 (28 to 32)	43 (40 to 48)	12.9 (12.2 to 13.7)	6.3 (5.7 to 6.8)
Cyclist road injuries	2 (2 to 2)	6 (5 to 6)	0.8 (0.7 to 0.9)	0.7 (0.7 to 0.8)
Motorcyclist road injuries	17 (15 to 18)	18 (16 to 21)	5.1 (4.6 to 5.6)	2.1 (1.8 to 2.3)
Motor vehicle road injuries	43 (40 to 46)	76 (68 to 85)	14.3 (13.4 to 15.3)	9.3 (8.4 to 10.3)
Other road injuries	2 (2 to 2)	3 (3 to 4)	0.8 (0.7 to 0.9)	0.5 (0.4 to 0.5)
Other transport injuries	3 (3 to 3)	7 (6 to 8)	1.1 (1.0 to 1.2)	0.9 (0.8 to 1.1)
**Unintentional injuries**	**69 (66 to 72)**	**101 (95 to 108)**	**31.5 (30.2 to 32.6)**	**16.2 (15.2 to 17.2)**
Falls	14 (13 to 15)	27 (25 to 29)	9.4 (8.9 to 9.9)	5.3 (4.9 to 5.7)
Drowning	16 (14 to 17)	18 (16 to 20)	5.5 (5.1 to 5.9)	2.3 (2.1 to 2.5)
Fire, heat, and hot substances	6 (5 to 6)	7 (7 to 8)	2.9 (2.8 to 3.1)	1.2 (1.1 to 1.3)
Poisonings	2 (2 to 2)	2 (2 to 3)	0.7 (0.6 to 0.7)	0.3 (0.3 to 0.3)
Poisoning by carbon monoxide	0 (0 to 0)	1 (1 to 1)	0.2 (0.1 to 0.2)	0.1 (0.1 to 0.1)
Poisoning by other means	1 (1 to 2)	2 (2 to 2)	0.5 (0.5 to 0.6)	0.2 (0.2 to 0.3)
Exposure to mechanical forces	8 (7 to 9)	8 (7 to 9)	3.0 (2.8 to 3.2)	1.1 (1.0 to 1.2)
Unintentional firearm injuries	3 (2 to 3)	2 (2 to 2)	0.9 (0.8 to 1.1)	0.2 (0.2 to 0.3)
Other exposure to mechanical forces	5 (5 to 6)	6 (6 to 7)	2.1 (1.9 to 2.2)	0.8 (0.8 to 0.9)
Adverse effects of medical treatment	5 (4 to 6)	11 (9 to 12)	3.1 (2.1 to 3.4)	2.0 (1.6 to 2.2)
Animal contact	1 (1 to 1)	1 (1 to 1)	0.4 (0.4 to 0.5)	0.2 (0.1 to 0.2)
Venomous animal contact	1 (1 to 1)	1 (0 to 1)	0.3 (0.2 to 0.3)	0.1 (0.1 to 0.1)
Non-venomous animal contact	0 (0 to 0)	0 (0 to 1)	0.1 (0.1 to 0.2)	0.1 (0.1 to 0.1)
Foreign body	10 (9 to 11)	19 (17 to 21)	3.8 (3.6 to 4.1)	2.7 (2.4 to 3.0)
Pulmonary aspiration and foreign body in airway	10 (9 to 11)	18 (16 to 20)	3.7 (3.4 to 4.0)	2.7 (2.4 to 2.9)
Foreign body in eyes				
Foreign body in other body part	0 (0 to 0)	0 (0 to 1)	0.1 (0.1 to 0.1)	0.0 (0.0 to 0.1)
Environmental heat and cold exposure	2 (2 to 2)	2 (2 to 3)	1.2 (1.1 to 1.3)	0.4 (0.3 to 0.4)
Exposure to forces of nature	0 (0 to 0)	0 (0 to 0)	0.1 (0.1 to 0.1)	0.0 (0.0 to 0.0)
Other unintentional injuries	4 (4 to 5)	6 (5 to 6)	1.4 (1.3 to 1.6)	0.7 (0.6 to 0.8)

Cause	Years of life lost			
	All-age counts (95% UI)		Age-standardised rates per 100,000 (95% UI)	
	1990	2017	1990	2017
**Transport and unintentional injuries**	**8 716 (8 330 to 9 113)**	**11 584 (10 670 to 12 493)**	**2 725.0 (2 619.5 to 2 832.5)**	**1 416.7 (1 308.9 to 1 524.0)**
**Transport injuries**	**5 274 (5 001 to 5 552)**	**7 441 (6 794 to 8 121)**	**1 599.0 (1 521.1 to 1 675.7)**	**875.6 (802.5 to 953.1)**
Road injuries	5 126 (4 861 to 5 393)	7 091 (6 484 to 7 759)	1 551.8 (1 476.7 to 1 625.1)	834.7 (763.8 to 907.6)
Pedestrian road injuries	1 488 (1 360 to 1 626)	1 851 (1 674 to 2 054)	490.9 (456.6 to 529.8)	231.3 (209.2 to 256.3)
Cyclist road injuries	115 (102 to 130)	255 (225 to 287)	35.8 (32.2 to 39.8)	30.8 (27.3 to 35.0)
Motorcyclist road injuries	979 (872 to 1 089)	970 (851 to 1 111)	274.9 (248.5 to 304.8)	109.0 (95.5 to 125.1)
Motor vehicle road injuries	2 444 (2 255 to 2 632)	3 852 (3 443 to 4 310)	716.5 (667.8 to 766.8)	444.2 (398.7 to 495.4)
Other road injuries	110 (95 to 129)	162 (141 to 190)	33.6 (29.3 to 38.7)	19.4 (16.9 to 22.5)
Other transport injuries	149 (133 to 167)	350 (300 to 402)	47.3 (42.2 to 52.5)	40.9 (35.3 to 46.7)
**Unintentional injuries**	**3 442 (3 221 to 3 668)**	**4 144 (3 811 to 4 492)**	**1 126.0 (1 070.1 to 1 183.1)**	**541.1 (498.3 to 589.6)**
Falls	435 (410 to 462)	712 (640 to 781)	198.5 (188.5 to 209.6)	105.0 (95.5 to 114.4)
Drowning	920 (829 to 1 007)	925 (828 to 1 030)	269.8 (247.2 to 292.3)	113.0 (101.4 to 126.0)
Fire, heat, and hot substances	283 (254 to 316)	258 (228 to 285)	94.9 (87.3 to 103.5)	34.9 (31.1 to 38.3)
Poisonings	110 (99 to 122)	112 (97 to 126)	32.7 (30.0 to 35.9)	13.9 (12.1 to 15.5)
Poisoning by carbon monoxide	25 (22 to 29)	30 (25 to 34)	7.4 (6.6 to 8.3)	3.6 (3.1 to 4.1)
Poisoning by other means	85 (76 to 94)	83 (72 to 93)	25.3 (23.0 to 27.8)	10.2 (8.9 to 11.4)
Exposure to mechanical forces	415 (381 to 453)	375 (338 to 417)	132.1 (122.4 to 141.9)	44.6 (40.4 to 49.3)
Unintentional firearm injuries	152 (131 to 184)	101 (77 to 119)	45.5 (39.9 to 54.3)	11.5 (8.8 to 13.5)
Other exposure to mechanical forces	263 (233 to 289)	275 (245 to 311)	86.6 (78.2 to 93.8)	33.1 (29.7 to 37.2)
Adverse effects of medical treatment	220 (162 to 247)	355 (292 to 402)	81.7 (58.1 to 90.9)	51.6 (42.5 to 58.1)
Animal contact	67 (57 to 79)	45 (39 to 50)	19.7 (17.3 to 22.6)	6.1 (5.3 to 6.9)
Venomous animal contact	48 (39 to 60)	25 (19 to 30)	13.8 (11.4 to 16.5)	3.5 (2.7 to 4.2)
Non-venomous animal contact	18 (16 to 22)	19 (16 to 26)	5.8 (5.1 to 7.0)	2.6 (2.2 to 3.4)
Foreign body	655 (582 to 738)	974 (842 to 1 129)	186.8 (169.1 to 206.7)	128.7 (110.5 to 150.7)
Pulmonary aspiration and foreign body in airway	632 (560 to 714)	953 (825 to 1 106)	180.5 (162.9 to 200.1)	126.3 (108.2 to 147.8)
Foreign body in eyes				
Foreign body in other body part	22 (18 to 27)	20 (16 to 40)	6.4 (5.3 to 7.7)	2.4 (1.9 to 4.8)
Environmental heat and cold exposure	91 (84 to 98)	93 (80 to 106)	36.4 (33.6 to 39.4)	11.7 (10.1 to 13.2)
Exposure to forces of nature	14 (13 to 16)	7 (6 to 7)	4.2 (3.8 to 4.6)	0.8 (0.8 to 0.9)
Other unintentional injuries	232 (209 to 257)	283 (251 to 330)	69.4 (63.2 to 75.8)	33.0 (28.9 to 37.8)

Cause	Incidence			
	All-age counts (95% UI)		Age-standardised rates per 100,000 (95% UI)	
	1990	2017	1990	2017
**Transport and unintentional injuries**	**11 897 (10 858 to 13 043)**	**35 674 (32 147 to 39 572)**	**4 351.3 (3 970.9 to 4 779.8)**	**4 855.7 (4 419.4 to 5 339.9)**
**Transport injuries**	**1 456 (1 262 to 1 701)**	**5 692 (4 881 to 6 600)**	**495.1 (434.4 to 562.8)**	**690.9 (599.2 to 791.6)**
Road injuries	1 395 (1 199 to 1 635)	5 598 (4 786 to 6 508)	475.0 (414.9 to 542.2)	678.4 (586.3 to 778.4)
Pedestrian road injuries	178 (144 to 218)	646 (518 to 794)	65.5 (54.1 to 78.2)	81.3 (65.9 to 98.8)
Cyclist road injuries	159 (120 to 210)	555 (438 to 713)	51.5 (40.8 to 65.7)	70.2 (54.7 to 88.6)
Motorcyclist road injuries	182 (143 to 226)	523 (393 to 672)	57.3 (46.0 to 69.5)	61.7 (47.5 to 78.3)
Motor vehicle road injuries	661 (550 to 789)	3 266 (2 757 to 3 882)	225.5 (191.5 to 262.7)	387.1 (326.6 to 456.4)
Other road injuries	216 (163 to 277)	609 (457 to 768)	75.2 (58.7 to 92.3)	78.0 (59.2 to 97.6)
Other transport injuries	60 (47 to 79)	94 (78 to 115)	20.2 (16.5 to 24.9)	12.5 (10.4 to 15.3)
**Unintentional injuries**	**10 442 (9 410 to 11 602)**	**29 982 (26 721 to 33 703)**	**3 856.2 (3 474.0 to 4 278.0)**	**4 164.8 (3 735.8 to 4 641.0)**
Falls	4 527 (3 926 to 5 184)	16 574 (14 518 to 18 789)	1 876.6 (1 664.7 to 2 108.8)	2 451.5 (2 147.9 to 2 755.1)
Drowning	15 (13 to 17)	19 (16 to 24)	4.9 (4.3 to 5.6)	2.5 (2.1 to 3.0)
Fire, heat, and hot substances	401 (319 to 505)	710 (581 to 864)	112.3 (92.0 to 136.7)	92.9 (76.1 to 114.0)
Poisonings	109 (77 to 154)	232 (156 to 325)	27.8 (20.2 to 38.7)	31.4 (21.3 to 44.4)
Poisoning by carbon monoxide	22 (13 to 37)	47 (28 to 80)	6.2 (3.8 to 9.6)	6.2 (3.1 to 10.7)
Poisoning by other means	86 (60 to 127)	185 (123 to 269)	21.6 (15.2 to 31.1)	25.2 (16.6 to 36.9)
Exposure to mechanical forces	2 310 (1 946 to 2 759)	4 900 (4 154 to 5 766)	720.6 (616.8 to 851.7)	592.6 (504.6 to 694.9)
Unintentional firearm injuries	140 (98 to 192)	213 (135 to 303)	41.1 (29.9 to 55.0)	25.1 (16.2 to 35.4)
Other exposure to mechanical forces	2 170 (1 830 to 2 599)	4 689 (3 964 to 5 547)	679.4 (583.6 to 800.2)	567.5 (482.0 to 667.2)
Adverse effects of medical treatment	381 (321 to 438)	1 355 (1 156 to 1 558)	148.4 (128.9 to 167.6)	178.9 (154.0 to 203.2)
Animal contact	665 (530 to 838)	1 359 (1 159 to 1 598)	217.0 (179.3 to 262.4)	177.7 (152.5 to 207.7)
Venomous animal contact	235 (175 to 317)	489 (385 to 619)	76.3 (57.9 to 100.0)	63.6 (50.2 to 79.7)
Non-venomous animal contact	431 (327 to 550)	870 (715 to 1 063)	140.7 (111.4 to 174.5)	114.1 (93.7 to 139.5)
Foreign body	1 205 (590 to 2 091)	3 507 (1 412 to 6 407)	476.0 (209.4 to 793.2)	466.4 (182.1 to 805.5)
Pulmonary aspiration and foreign body in airway	34 (25 to 46)	36 (26 to 47)	9.5 (7.2 to 12.3)	4.9 (3.6 to 6.6)
Foreign body in eyes	1 075 (460 to 1 908)	3 304 (1 208 to 6 191)	438.6 (168.8 to 752.5)	439.3 (154.8 to 782.9)
Foreign body in other body part	95 (70 to 130)	168 (129 to 215)	27.9 (21.3 to 36.3)	22.2 (17.0 to 28.0)
Environmental heat and cold exposure	159 (116 to 210)	178 (129 to 233)	56.0 (43.6 to 68.5)	25.0 (18.5 to 32.7)
Exposure to forces of nature	38 (31 to 47)	9 (7 to 11)	10.7 (9.0 to 12.9)	1.1 (0.9 to 1.3)
Other unintentional injuries	632 (523 to 780)	1 138 (948 to 1 379)	205.9 (172.7 to 245.6)	144.9 (119.5 to 175.4)

Cause	Prevalence			
	All-age counts (95% UI)		Age-standardised rates per 100,000 (95% UI)	
	1990	2017	1990	2017
**Transport and unintentional injuries**	**22 418 (21 025 to 23 916)**	**75 940 (70 922 to 82 068)**	**9 720.1 (9 161.8 to 10 398.2)**	**10 306.2 (9 673.6 to 11 113.4)**
**Transport injuries**	**3 328 (3 097 to 3 557)**	**15 449 (14 257 to 16 760)**	**1 484.1 (1 379.6 to 1 590.7)**	**2 015.4 (1 863.6 to 2 186.4)**
Road injuries	3 060 (2 839 to 3 283)	14 985 (13 811 to 16 254)	1 375.4 (1 275.0 to 1 479.5)	1 952.7 (1 808.1 to 2 119.2)
Pedestrian road injuries	643 (554 to 755)	2 884 (2 476 to 3 389)	296.4 (259.3 to 348.9)	385.2 (332.9 to 449.5)
Cyclist road injuries	268 (221 to 310)	1 202 (1 011 to 1 427)	112.1 (94.9 to 129.4)	156.6 (132.6 to 183.1)
Motorcyclist road injuries	622 (538 to 719)	2 335 (1 995 to 2 738)	268.9 (233.0 to 310.0)	294.0 (251.6 to 343.9)
Motor vehicle road injuries	1 126 (938 to 1 315)	7 099 (6 199 to 8 346)	520.0 (452.3 to 601.4)	921.6 (802.6 to 1 077.2)
Other road injuries	406 (337 to 496)	1 465 (1 215 to 1 786)	178.1 (148.9 to 214.0)	195.3 (162.6 to 236.6)
Other transport injuries	268 (222 to 322)	465 (396 to 537)	108.7 (92.3 to 126.4)	62.7 (54.2 to 71.6)
**Unintentional injuries**	**19 090 (17 724 to 20 582)**	**60 490 (55 844 to 66 507)**	**8 236.0 (7 681.3 to 8 884.4)**	**8 290.8 (7 673.5 to 9 105.3)**
Falls	7 898 (6 928 to 9 037)	33 278 (29 256 to 38 051)	3 737.9 (3 326.6 to 4 242.7)	4 791.6 (4 253.4 to 5 497.5)
Drowning	66 (58 to 76)	114 (100 to 132)	30.5 (27.1 to 34.7)	15.5 (13.7 to 17.9)
Fire, heat, and hot substances	3 789 (3 150 to 4 805)	8 528 (7 026 to 10 771)	1 418.6 (1 194.7 to 1 743.2)	1 077.9 (893.6 to 1 358.6)
Poisonings	97 (74 to 120)	301 (220 to 381)	31.5 (24.4 to 38.9)	36.7 (26.9 to 46.5)
Poisoning by carbon monoxide	15 (12 to 20)	43 (32 to 56)	5.2 (4.0 to 6.7)	5.3 (4.0 to 6.9)
Poisoning by other means	82 (59 to 103)	258 (180 to 337)	26.3 (19.3 to 33.3)	31.5 (22.0 to 41.1)
Exposure to mechanical forces	4 391 (3 882 to 5 089)	11 886 (10 292 to 13 870)	1 876.2 (1 645.5 to 2 147.5)	1 523.1 (1 326.3 to 1 771.0)
Unintentional firearm injuries	514 (405 to 616)	1 036 (820 to 1 246)	218.7 (176.2 to 261.8)	134.0 (107.0 to 159.7)
Other exposure to mechanical forces	3 877 (3 275 to 4 535)	10 850 (9 236 to 12 914)	1 657.6 (1 418.7 to 1 938.4)	1 389.1 (1 188.7 to 1 640.9)
Adverse effects of medical treatment	29 (23 to 36)	104 (80 to 128)	11.4 (8.8 to 14.0)	13.7 (10.6 to 16.9)
Animal contact	700 (597 to 822)	1 691 (1 472 to 1 944)	285.6 (249.0 to 326.9)	224.5 (196.3 to 256.4)
Venomous animal contact	155 (127 to 183)	363 (305 to 421)	59.2 (49.8 to 68.7)	47.1 (39.7 to 54.3)
Non-venomous animal contact	544 (448 to 663)	1 328 (1 115 to 1 573)	226.4 (190.8 to 266.9)	177.4 (150.0 to 208.5)
Foreign body	537 (406 to 790)	1 331 (977 to 1 912)	218.4 (169.2 to 294.4)	184.2 (137.1 to 257.3)
Pulmonary aspiration and foreign body in airway	25 (18 to 34)	34 (27 to 42)	8.6 (6.4 to 11.4)	4.3 (3.4 to 5.4)
Foreign body in eyes	207 (100 to 384)	679 (344 to 1 264)	98.7 (55.1 to 164.2)	99.5 (54.7 to 169.2)
Foreign body in other body part	304 (241 to 388)	618 (515 to 746)	111.1 (89.6 to 138.8)	80.5 (67.5 to 96.5)
Environmental heat and cold exposure	557 (448 to 680)	718 (594 to 858)	226.4 (188.4 to 268.6)	97.8 (82.8 to 115.4)
Exposure to forces of nature	122 (88 to 167)	547 (375 to 778)	44.1 (32.0 to 60.0)	65.8 (45.6 to 92.8)
Other unintentional injuries	905 (778 to 1 058)	1 999 (1 720 to 2 307)	375.4 (323.4 to 430.1)	260.9 (226.0 to 298.9)

Cause	Years lived with disability			
	All-age counts (95% UI)		Age-standardised rates per 100,000 (95% UI)	
	1990	2017	1990	2017
**Transport and unintentional injuries**	**976 (709 to 1 310)**	**3 315 (2 391 to 4 478)**	**419.5 (302.4 to 556.8)**	**449.8 (323.1 to 601.4)**
**Transport injuries**	**220 (161 to 293)**	**943 (679 to 1 258)**	**94.0 (68.8 to 124.7)**	**120.2 (86.4 to 160.2)**
Road injuries	204 (148 to 271)	918 (661 to 1 226)	87.7 (64.0 to 117.1)	117.0 (84.0 to 156.3)
Pedestrian road injuries	45 (31 to 60)	184 (129 to 252)	19.6 (14.0 to 26.7)	24.0 (16.9 to 32.9)
Cyclist road injuries	18 (12 to 23)	72 (49 to 96)	7.2 (5.1 to 9.5)	9.2 (6.4 to 12.2)
Motorcyclist road injuries	42 (29 to 56)	142 (96 to 194)	17.3 (12.0 to 23.5)	17.5 (11.9 to 23.9)
Motor vehicle road injuries	78 (57 to 104)	450 (324 to 600)	34.2 (25.0 to 45.5)	56.9 (41.2 to 76.1)
Other road injuries	22 (16 to 30)	72 (51 to 99)	9.4 (6.8 to 12.7)	9.4 (6.7 to 13.0)
Other transport injuries	16 (11 to 22)	25 (17 to 33)	6.3 (4.5 to 8.4)	3.2 (2.3 to 4.3)
**Unintentional injuries**	**756 (548 to 1 020)**	**2 372 (1 696 to 3 225)**	**325.5 (234.2 to 439.8)**	**329.5 (235.3 to 445.8)**
Falls	446 (325 to 590)	1 674 (1 210 to 2 225)	204.0 (147.8 to 268.1)	239.9 (171.4 to 319.6)
Drowning	4 (3 to 5)	6 (4 to 8)	1.6 (1.1 to 2.3)	0.8 (0.6 to 1.1)
Fire, heat, and hot substances	79 (48 to 125)	164 (96 to 266)	28.6 (17.1 to 45.2)	20.6 (12.1 to 33.5)
Poisonings	11 (7 to 17)	35 (21 to 54)	3.6 (2.2 to 5.2)	4.3 (2.5 to 6.5)
Poisoning by carbon monoxide	2 (1 to 3)	5 (3 to 7)	0.5 (0.3 to 0.8)	0.5 (0.3 to 0.9)
Poisoning by other means	10 (6 to 15)	31 (18 to 48)	3.0 (1.8 to 4.6)	3.7 (2.2 to 5.8)
Exposure to mechanical forces	101 (68 to 145)	255 (172 to 371)	42.1 (28.6 to 60.5)	32.5 (21.9 to 47.2)
Unintentional firearm injuries	10 (7 to 16)	19 (12 to 30)	4.2 (2.7 to 6.6)	2.5 (1.6 to 3.8)
Other exposure to mechanical forces	90 (61 to 130)	236 (158 to 346)	37.9 (25.7 to 54.4)	30.0 (20.2 to 43.8)
Adverse effects of medical treatment	4 (2 to 6)	14 (9 to 21)	1.5 (0.9 to 2.3)	1.8 (1.1 to 2.8)
Animal contact	18 (12 to 26)	39 (26 to 56)	6.7 (4.5 to 9.5)	5.0 (3.3 to 7.1)
Venomous animal contact	10 (6 to 15)	21 (13 to 30)	3.4 (2.2 to 4.9)	2.5 (1.6 to 3.6)
Non-venomous animal contact	8 (5 to 13)	18 (11 to 30)	3.3 (2.0 to 5.3)	2.5 (1.4 to 4.0)
Foreign body	32 (21 to 49)	75 (46 to 121)	12.9 (8.4 to 19.5)	10.4 (6.3 to 16.8)
Pulmonary aspiration and foreign body in airway	1 (1 to 2)	1 (1 to 2)	0.4 (0.3 to 0.6)	0.2 (0.1 to 0.2)
Foreign body in eyes	14 (7 to 25)	45 (22 to 85)	6.6 (3.4 to 11.8)	6.6 (3.4 to 12.1)
Foreign body in other body part	17 (12 to 23)	28 (20 to 38)	5.9 (4.2 to 7.8)	3.6 (2.5 to 4.8)
Environmental heat and cold exposure	21 (14 to 31)	27 (17 to 38)	8.7 (5.8 to 12.3)	3.7 (2.4 to 5.2)
Exposure to forces of nature	6 (4 to 8)	17 (13 to 25)	2.1 (1.6 to 2.8)	2.1 (1.5 to 2.9)
Other unintentional injuries	34 (24 to 46)	66 (46 to 90)	13.6 (9.7 to 18.5)	8.6 (6.0 to 11.7)

Cause	Disability adjusted life years			
	All-age counts (95% UI)		Age-standardised rates per 100,000 (95% UI)	
	1990	2017	1990	2017
**Transport and unintentional injuries**	**9 692 (9 208 to 10 211)**	**14 900 (13 527 to 16 347)**	**3 144.5 (2 973.5 to 3 321.6)**	**1 868.5 (1 694.9 to 2 064.1)**
**Transport injuries**	**5 494 (5 208 to 5 771)**	**8 384 (7 643 to 9 122)**	**1 693.0 (1 612.9 to 1 773.8)**	**995.8 (910.2 to 1 080.5)**
Road injuries	5 329 (5 052 to 5 602)	8 009 (7 300 to 8 722)	1 639.5 (1 560.9 to 1 717.8)	951.6 (869.6 to 1 034.4)
Pedestrian road injuries	1 528 (1 409 to 1 670)	2 034 (1 848 to 2 253)	510.5 (476.3 to 549.5)	255.2 (232.5 to 280.9)
Cyclist road injuries	133 (119 to 147)	326 (289 to 367)	43.0 (38.9 to 47.2)	40.0 (35.4 to 45.0)
Motorcyclist road injuries	1 015 (912 to 1 130)	1 112 (977 to 1 261)	292.2 (264.8 to 322.9)	126.5 (111.5 to 143.4)
Motor vehicle road injuries	2 522 (2 328 to 2 716)	4 302 (3 857 to 4 780)	750.7 (701.2 to 802.0)	501.1 (449.7 to 555.9)
Other road injuries	133 (115 to 154)	234 (204 to 270)	43.0 (37.8 to 48.7)	28.8 (25.1 to 33.3)
Other transport injuries	165 (148 to 183)	375 (322 to 428)	53.5 (48.3 to 59.0)	44.2 (38.4 to 49.9)
**Unintentional injuries**	**4 198 (3 895 to 4 541)**	**6 516 (5 729 to 7 456)**	**1 451.5 (1 341.2 to 1 578.8)**	**872.6 (761.0 to 1 004.4)**
Falls	882 (762 to 1 027)	2 386 (1 906 to 2 957)	402.5 (347.3 to 468.3)	344.9 (274.6 to 429.4)
Drowning	924 (834 to 1 011)	931 (834 to 1 036)	271.4 (248.7 to 293.9)	113.8 (102.2 to 126.8)
Fire, heat, and hot substances	361 (318 to 417)	422 (347 to 531)	123.4 (110.2 to 142.3)	55.4 (46.1 to 69.2)
Poisonings	121 (110 to 135)	148 (126 to 169)	36.3 (33.2 to 39.6)	18.1 (15.6 to 20.7)
Poisoning by carbon monoxide	27 (24 to 31)	34 (29 to 39)	8.0 (7.1 to 8.9)	4.2 (3.5 to 4.7)
Poisoning by other means	94 (85 to 105)	113 (96 to 132)	28.3 (25.7 to 31.1)	14.0 (11.9 to 16.2)
Exposure to mechanical forces	515 (467 to 567)	630 (536 to 760)	174.2 (157.6 to 193.3)	77.0 (65.3 to 93.4)
Unintentional firearm injuries	162 (141 to 196)	120 (95 to 141)	49.7 (43.8 to 58.8)	13.9 (11.0 to 16.4)
Other exposure to mechanical forces	353 (311 to 397)	510 (425 to 631)	124.5 (109.7 to 141.3)	63.1 (52.5 to 77.9)
Adverse effects of medical treatment	224 (166 to 250)	369 (306 to 415)	83.2 (59.5 to 91.9)	53.4 (44.6 to 59.9)
Animal contact	85 (74 to 99)	84 (68 to 102)	26.4 (23.1 to 30.4)	11.1 (9.1 to 13.5)
Venomous animal contact	59 (49 to 70)	46 (37 to 56)	17.2 (14.6 to 20.2)	6.0 (4.9 to 7.3)
Non-venomous animal contact	26 (22 to 33)	38 (29 to 51)	9.2 (7.6 to 11.4)	5.0 (3.9 to 6.8)
Foreign body	687 (612 to 771)	1 048 (910 to 1 211)	199.7 (181.3 to 220.7)	139.0 (120.0 to 162.1)
Pulmonary aspiration and foreign body in airway	634 (562 to 715)	955 (827 to 1 108)	180.9 (163.3 to 200.5)	126.5 (108.3 to 147.9)
Foreign body in eyes	14 (7 to 25)	45 (22 to 85)	6.6 (3.4 to 11.8)	6.6 (3.4 to 12.1)
Foreign body in other body part	39 (33 to 47)	48 (38 to 69)	12.3 (10.3 to 14.5)	6.0 (4.7 to 8.5)
Environmental heat and cold exposure	112 (102 to 124)	120 (103 to 138)	45.0 (40.9 to 49.3)	15.4 (13.3 to 17.7)
Exposure to forces of nature	20 (18 to 23)	24 (19 to 32)	6.3 (5.7 to 7.1)	2.9 (2.3 to 3.7)
Other unintentional injuries	266 (240 to 293)	354 (313 to 404)	83.0 (75.8 to 90.8)	41.6 (35.8 to 47.3)

Death, YLL, incidence, prevalence, YLD, and DALY counts and rates per 100,000 for all injuries in Baja California in 1990 and 2017

Cause	Deaths				Years of life lost			
	All-age counts (95% UI)		Age-standardised rates per 100,000 (95% UI)		All-age counts (95% UI)		Age-standardised rates per 100,000 (95% UI)	
	1990	2017	1990	2017	1990	2017	1990	2017
**Transport and unintentional injuries**	**1 150 (1 104 to 1 199)**	**1 375 (1 283 to 1 467)**	**87.4 (84.6 to 90.0)**	**40.0 (37.5 to 42.6)**	**62 600 (59 012 to 66 513)**	**61 191 (56 580 to 65 794)**	**3 639.6 (3 472.4 to 3 817.1)**	**1 588.3 (1 472.3 to 1 708.3)**
**Transport injuries**	**513 (488 to 541)**	**602 (557 to 656)**	**34.0 (32.4 to 35.5)**	**15.8 (14.6 to 17.1)**	**28 621 (26 966 to 30 323)**	**28 797 (26 410 to 31 371)**	**1 611.1 (1 529.2 to 1 700.4)**	**708.8 (652.0 to 770.3)**
Road injuries	505 (480 to 534)	585 (540 to 636)	33.4 (31.9 to 35.0)	15.3 (14.2 to 16.6)	28 205 (26 546 to 29 876)	27 973 (25 699 to 30 457)	1 586.6 (1 505.8 to 1 676.6)	688.6 (633.6 to 748.0)
Pedestrian road injuries	185 (172 to 199)	229 (208 to 253)	14.5 (13.7 to 15.4)	6.4 (5.9 to 7.0)	9 627 (8 805 to 10 558)	9 899 (8 919 to 10 980)	588.4 (545.8 to 635.5)	251.5 (228.8 to 278.4)
Cyclist road injuries	10 (9 to 11)	22 (19 to 25)	0.7 (0.6 to 0.7)	0.6 (0.5 to 0.6)	587 (480 to 604)	964 (852 to 1 103)	31.2 (28.2 to 34.5)	24.1 (21.4 to 27.3)
Motorcyclist road injuries	90 (81 to 101)	90 (79 to 102)	5.2 (4.7 to 5.7)	2.2 (1.9 to 2.4)	5 277 (4 726 to 5 943)	4 812 (4 180 to 5 485)	281.5 (254.7 to 313.5)	114.1 (99.4 to 130.3)
Motor vehicle road injuries	217 (200 to 234)	239 (215 to 264)	12.9 (12.0 to 13.8)	6.0 (5.4 to 6.6)	12 552 (11 504 to 13 602)	12 041 (10 827 to 13 400)	673.6 (622.6 to 725.5)	292.5 (264.1 to 324.2)
Other road injuries	4 (3 to 4)	5 (5 to 6)	0.3 (0.2 to 0.3)	0.1 (0.1 to 0.2)	212 (178 to 252)	257 (220 to 300)	12.0 (10.3 to 13.9)	6.4 (5.5 to 7.4)
Other transport injuries	8 (7 to 9)	18 (15 to 20)	0.6 (0.5 to 0.6)	0.5 (0.4 to 0.5)	416 (370 to 469)	824 (714 to 952)	24.5 (22.0 to 27.2)	20.2 (17.6 to 23.3)
**Unintentional injuries**	**637 (601 to 674)**	**772 (719 to 824)**	**53.5 (51.3 to 55.3)**	**24.2 (22.7 to 25.7)**	**33 979 (31 326 to 37 055)**	**32 394 (29 810 to 34 897)**	**2 028.5 (1 903.6 to 2 166.9)**	**879.4 (807.9 to 946.7)**
Falls	106 (102 to 112)	195 (175 to 212)	14.0 (13.3 to 14.7)	7.2 (6.5 to 7.9)	3 693 (3 472 to 3 925)	5 487 (4 832 to 6 000)	308.7 (294.3 to 324.2)	159.7 (142.8 to 173.8)
Drowning	150 (140 to 162)	121 (110 to 132)	9.4 (8.9 to 10.0)	3.2 (2.9 to 3.5)	8 999 (8 207 to 9 848)	6 011 (5 454 to 6 603)	485.1 (448.6 to 524.5)	155.5 (139.3 to 168.4)
Fire, heat, and hot substances	68 (63 to 74)	68 (60 to 74)	6.4 (6.0 to 6.7)	2.3 (2.0 to 2.5)	3 490 (3 112 to 3 881)	2 471 (2 179 to 2 721)	215.6 (197.0 to 237.5)	68.0 (60.6 to 74.4)
Poisonings	31 (28 to 34)	30 (27 to 34)	2.1 (1.9 to 2.2)	0.8 (0.8 to 0.9)	1 817 (1 635 to 2 013)	1 437 (1 281 to 1 584)	100.6 (91.9 to 109.7)	37.0 (33.1 to 40.6)
Poisoning by carbon monoxide	16 (14 to 17)	17 (15 to 19)	1.1 (1.0 to 1.1)	0.5 (0.4 to 0.5)	917 (820 to 1 026)	803 (704 to 897)	50.7 (46.1 to 55.7)	20.4 (18.0 to 22.7)
Poisoning by other means	15 (14 to 17)	13 (12 to 15)	1.0 (0.9 to 1.1)	0.4 (0.3 to 0.4)	900 (798 to 1 016)	633 (559 to 708)	49.8 (44.8 to 55.4)	16.5 (14.7 to 18.4)
Exposure to mechanical forces	89 (84 to 96)	90 (83 to 98)	6.4 (6.0 to 6.7)	2.4 (2.2 to 2.6)	4 782 (4 441 to 5 180)	4 028 (3 634 to 4 432)	282.6 (264.9 to 302.3)	99.6 (90.4 to 109.4)
Unintentional firearm injuries	43 (39 to 53)	37 (32 to 42)	2.9 (2.6 to 3.5)	0.9 (0.8 to 1.1)	2 393 (2 146 to 2 883)	1 784 (1 550 to 2 029)	136.4 (123.4 to 164.8)	43.1 (37.6 to 49.1)
Other exposure to mechanical forces	46 (35 to 53)	53 (46 to 59)	3.5 (2.8 to 3.8)	1.5 (1.3 to 1.6)	2 390 (1 792 to 2 668)	2 244 (1 950 to 2 537)	146.2 (111.6 to 161.0)	56.6 (49.5 to 63.5)
Adverse effects of medical treatment	31 (29 to 35)	58 (49 to 64)	3.6 (2.5 to 3.9)	2.1 (1.8 to 2.3)	1 364 (975 to 1 560)	1 920 (1 616 to 2 176)	96.1 (69.0 to 107.1)	56.5 (48.3 to 64.1)
Animal contact	4 (4 to 5)	4 (3 to 4)	0.3 (0.3 to 0.4)	0.1 (0.1 to 0.1)	269 (226 to 322)	150 (135 to 168)	14.9 (12.9 to 17.4)	4.2 (3.8 to 4.7)
Venomous animal contact	2 (2 to 3)	1 (1 to 1)	0.1 (0.1 to 0.2)	0.0 (0.0 to 0.0)	136 (103 to 182)	48 (35 to 65)	7.0 (5.4 to 9.2)	1.4 (1.0 to 1.9)
Non-venomous animal contact	2 (2 to 3)	3 (2 to 3)	0.2 (0.2 to 0.2)	0.1 (0.1 to 0.1)	133 (102 to 154)	103 (84 to 119)	7.9 (6.3 to 9.0)	2.8 (2.3 to 3.3)
Foreign body	120 (107 to 136)	170 (154 to 188)	8.2 (7.6 to 8.9)	5.1 (4.6 to 5.6)	7 729 (6 644 to 9 074)	9 308 (8 177 to 10 730)	407.8 (358.9 to 467.5)	261.5 (228.1 to 303.4)
Pulmonary aspiration and foreign body in airway	113 (100 to 128)	164 (148 to 182)	7.8 (7.2 to 8.5)	4.9 (4.5 to 5.5)	7 308 (6 249 to 8 588)	9 003 (7 888 to 10 381)	384.6 (337.0 to 442.1)	253.9 (221.0 to 295.4)
Foreign body in eyes								
Foreign body in other body part	7 (4 to 8)	6 (4 to 7)	0.4 (0.2 to 0.5)	0.1 (0.1 to 0.2)	421 (255 to 495)	305 (243 to 360)	23.1 (13.9 to 26.8)	7.6 (6.2 to 8.9)
Environmental heat and cold exposure	20 (19 to 21)	18 (16 to 21)	2.1 (1.9 to 2.2)	0.5 (0.5 to 0.6)	872 (801 to 947)	698 (590 to 789)	63.6 (59.3 to 68.1)	18.0 (15.4 to 20.2)
Exposure to forces of nature	1 (1 to 1)	1 (1 to 1)	0.1 (0.1 to 0.1)	0.0 (0.0 to 0.0)	68 (61 to 76)	31 (28 to 34)	3.8 (3.4 to 4.1)	0.8 (0.8 to 0.9)
Other unintentional injuries	16 (14 to 17)	17 (15 to 20)	1.0 (0.9 to 1.1)	0.4 (0.4 to 0.5)	896 (817 to 994)	854 (751 to 968)	49.8 (45.8 to 54.6)	20.6 (18.3 to 23.3)

Cause	Incidence				Prevalence			
	All-age counts (95% UI)		Age-standardised rates per 100,000 (95% UI)		All-age counts (95% UI)		Age-standardised rates per 100,000 (95% UI)	
	1990	2017	1990	2017	1990	2017	1990	2017
**Transport and unintentional injuries**	**67 517 (61 793 to 74 489)**	**146 981 (133 474 to 163 663)**	**4 709.4 (4 310.3 to 5 152.9)**	**4 198.5 (3 812.3 to 4 624.5)**	**133 983 (125 842 to 143 383)**	**309 052 (289 089 to 333 543)**	**11 037.5 (10 386.9 to 11 813.4)**	**8 794.0 (8 251.6 to 9 492.8)**
**Transport injuries**	**6 560 (5 610 to 7 667)**	**20 112 (17 515 to 23 049)**	**423.2 (367.1 to 480.7)**	**517.5 (449.0 to 590.3)**	**15 176 (14 115 to 16 191)**	**54 264 (50 423 to 58 520)**	**1 272.7 (1 183.2 to 1 361.2)**	**1 486.9 (1 383.9 to 1 603.6)**
Road injuries	6 329 (5 404 to 7 429)	19 822 (17 200 to 22 746)	409.2 (353.6 to 466.5)	509.4 (441.8 to 583.0)	14 092 (13 072 to 15 087)	52 800 (48 998 to 57 043)	1 193.2 (1 107.3 to 1 280.3)	1 445.8 (1 341.6 to 1 560.8)
Pedestrian road injuries	1 013 (815 to 1 240)	3 506 (2 862 to 4 244)	71.0 (58.8 to 84.6)	92.6 (75.9 to 111.4)	3 597 (3 091 to 4 200)	14 692 (12 689 to 17 209)	312.0 (272.5 to 362.7)	411.1 (356.8 to 477.3)
Cyclist road injuries	770 (585 to 1 013)	2 149 (1 667 to 2 769)	47.7 (37.6 to 60.8)	57.7 (45.0 to 73.7)	1 244 (1 035 to 1 471)	4 385 (3 693 to 5 165)	99.3 (83.7 to 115.1)	119.9 (102.3 to 139.7)
Motorcyclist road injuries	963 (744 to 1 212)	1 847 (1 393 to 2 337)	58.0 (46.6 to 71.5)	46.4 (35.3 to 58.7)	3 204 (2 749 to 3 709)	7 864 (6 727 to 9 282)	262.5 (226.7 to 303.3)	208.8 (178.9 to 245.3)
Motor vehicle road injuries	2 744 (2 231 to 3 287)	10 442 (8 681 to 12 377)	177.7 (147.3 to 207.9)	262.5 (221.5 to 309.2)	4 491 (3 944 to 5 251)	21 624 (18 840 to 25 350)	391.6 (341.3 to 455.5)	587.1 (512.5 to 686.0)
Other road injuries	833 (587 to 1 135)	1 879 (1 339 to 2 644)	54.8 (40.6 to 70.6)	50.3 (37.3 to 70.4)	1 556 (1 285 to 1 889)	4 233 (3 506 to 5 163)	127.8 (106.2 to 153.7)	118.8 (98.2 to 143.5)
Other transport injuries	231 (161 to 323)	290 (226 to 373)	13.9 (10.4 to 18.4)	8.1 (6.4 to 10.4)	1 084 (876 to 1 333)	1 463 (1 253 to 1 687)	79.5 (66.0 to 94.6)	41.1 (35.7 to 47.0)
**Unintentional injuries**	**60 958 (55 591 to 67 652)**	**126 870 (113 705 to 142 759)**	**4 286.2 (3 892.1 to 4 740.1)**	**3 681.0 (3 303.2 to 4 094.4)**	**118 807 (110 691 to 127 955)**	**254 788 (236 071 to 279 169)**	**9 764.8 (9 113.1 to 10 513.2)**	**7 307.0 (6 795.4 to 7 999.1)**
Falls	24 632 (21 802 to 27 963)	70 071 (61 790 to 79 042)	1 985.8 (1 782.8 to 2 235.0)	2 153.9 (1 916.2 to 2 428.8)	42 867 (37 625 to 48 789)	138 139 (122 374 to 157 927)	3 896.4 (3 479.7 to 4 447.1)	4 147.1 (3 709.9 to 4 741.5)
Drowning	124 (108 to 143)	102 (83 to 128)	7.8 (6.8 to 8.9)	2.8 (2.3 to 3.4)	558 (491 to 643)	599 (526 to 700)	48.3 (42.7 to 55.0)	17.3 (15.2 to 20.1)
Fire, heat, and hot substances	2 948 (2 429 to 3 541)	3 466 (2 908 to 4 125)	159.7 (133.4 to 187.1)	95.6 (80.0 to 114.7)	28 470 (23 887 to 35 111)	40 856 (33 791 to 50 715)	2 034.1 (1 736.1 to 2 426.3)	1 090.2 (908.3 to 1 344.9)
Poisonings	870 (657 to 1 165)	884 (588 to 1 243)	43.7 (33.4 to 57.5)	25.1 (16.8 to 35.3)	770 (592 to 946)	1 084 (799 to 1 367)	48.2 (37.6 to 59.0)	28.0 (20.7 to 35.3)
Poisoning by carbon monoxide	174 (118 to 253)	182 (99 to 301)	9.6 (6.9 to 13.5)	5.1 (2.8 to 8.5)	119 (92 to 153)	150 (114 to 192)	7.8 (6.1 to 9.8)	3.9 (3.0 to 5.0)
Poisoning by other means	696 (515 to 963)	703 (451 to 1 022)	34.0 (25.5 to 46.6)	20.0 (12.7 to 29.4)	651 (478 to 826)	934 (657 to 1 212)	40.5 (29.9 to 51.2)	24.1 (17.0 to 31.2)
Exposure to mechanical forces	17 353 (14 635 to 20 596)	20 770 (17 242 to 25 152)	1 039.1 (894.3 to 1 209.9)	529.3 (443.7 to 632.0)	32 454 (28 254 to 37 268)	49 385 (43 149 to 57 079)	2 667.1 (2 349.8 to 3 046.2)	1 335.8 (1 172.0 to 1 537.5)
Unintentional firearm injuries	1 558 (1 149 to 2 093)	1 268 (902 to 1 748)	90.1 (68.2 to 117.3)	31.8 (22.5 to 43.1)	5 175 (4 087 to 6 242)	5 747 (4 631 to 6 935)	432.2 (347.7 to 521.8)	156.6 (126.6 to 189.4)
Other exposure to mechanical forces	15 805 (13 367 to 18 734)	19 502 (16 248 to 23 486)	949.0 (813.7 to 1 109.9)	497.5 (416.3 to 592.2)	27 279 (22 950 to 32 349)	43 638 (37 585 to 51 605)	2 235.0 (1 905.7 to 2 634.6)	1 179.2 (1 015.8 to 1 386.6)
Adverse effects of medical treatment	1 826 (1 558 to 2 095)	5 859 (4 948 to 6 708)	137.8 (119.8 to 156.3)	161.9 (136.2 to 183.1)	140 (108 to 173)	448 (344 to 559)	10.5 (8.2 to 12.9)	12.4 (9.5 to 15.2)
Animal contact	3 151 (2 394 to 4 161)	4 284 (3 377 to 5 421)	195.0 (153.6 to 244.3)	119.8 (95.5 to 149.8)	3 361 (2 863 to 3 940)	5 533 (4 734 to 6 436)	263.2 (227.6 to 300.9)	153.6 (131.8 to 177.7)
Venomous animal contact	1 125 (780 to 1 605)	1 369 (985 to 1 847)	68.4 (48.6 to 93.8)	37.9 (27.4 to 50.9)	745 (608 to 886)	1 033 (857 to 1 230)	53.7 (44.6 to 62.6)	27.7 (23.0 to 32.8)
Non-venomous animal contact	2 025 (1 470 to 2 705)	2 915 (2 239 to 3 733)	126.7 (96.8 to 163.0)	81.9 (63.5 to 104.8)	2 616 (2 158 to 3 173)	4 500 (3 722 to 5 401)	207.5 (174.8 to 246.6)	125.9 (104.7 to 149.8)
Foreign body	6 505 (3 250 to 11 292)	15 794 (6 585 to 28 781)	487.1 (226.0 to 840.2)	438.9 (186.8 to 752.9)	3 067 (2 370 to 4 094)	5 552 (3 995 to 8 013)	240.7 (189.7 to 311.5)	160.6 (117.5 to 225.6)
Pulmonary aspiration and foreign body in airway	290 (212 to 384)	211 (156 to 283)	15.3 (11.7 to 19.7)	6.1 (4.5 to 8.4)	210 (150 to 285)	195 (155 to 244)	13.7 (10.1 to 18.2)	5.3 (4.2 to 6.5)
Foreign body in eyes	5 655 (2 461 to 10 458)	14 975 (5 785 to 28 016)	439.1 (174.5 to 792.9)	415.6 (162.9 to 731.3)	1 086 (632 to 2 050)	3 102 (1 628 to 5 556)	99.3 (56.0 to 166.2)	93.6 (52.0 to 157.2)
Foreign body in other body part	560 (430 to 757)	608 (476 to 763)	32.8 (25.7 to 42.8)	17.2 (13.4 to 21.4)	1 771 (1 421 to 2 230)	2 255 (1 887 to 2 743)	127.8 (104.7 to 157.9)	61.7 (51.9 to 74.7)
Environmental heat and cold exposure	863 (674 to 1 080)	853 (679 to 1 034)	68.4 (53.9 to 74.7)	25.1 (20.2 to 30.2)	2 916 (2 399 to 3 504)	3 212 (2 739 to 3 774)	240.2 (204.7 to 279.9)	92.5 (79.8 to 108.1)
Exposure to forces of nature	179 (146 to 224)	42 (35 to 50)	9.9 (8.2 to 11.9)	1.1 (0.9 to 1.3)	576 (412 to 794)	1 693 (1 170 to 2 396)	34.0 (25.0 to 46.5)	42.5 (29.6 to 59.0)
Other unintentional injuries	2 500 (2 022 to 3 109)	4 748 (3 944 to 5 694)	156.9 (129.4 to 188.9)	127.8 (105.3 to 152.6)	3 629 (3 101 to 4 241)	8 288 (7 206 to 9 525)	284.0 (245.7 to 326.2)	227.1 (198.8 to 258.5)

Cause	Years lived with disability				Disability-adjusted life-years			
	All-age counts (95% UI)		Age-standardised rates per 100,000 (95% UI)		All-age counts (95% UI)		Age-standardised rates per 100,000 (95% UI)	
	1990	2017	1990	2017	1990	2017	1990	2017
**Transport and unintentional injuries**	**5 546 (4 038 to 7 386)**	**13 044 (9 348 to 17 664)**	**451.6 (328.3 to 600.4)**	**372.0 (266.0 to 503.8)**	**68 146 (64 159 to 72 543)**	**74 234 (68 034 to 80 602)**	**4 091.2 (3 873.3 to 4 316.4)**	**1 960.3 (1 791.3 to 2 132.6)**
**Transport injuries**	**1 019 (748 to 1 346)**	**3 252 (2 344 to 4 365)**	**81.3 (59.8 to 107.2)**	**87.1 (62.9 to 116.4)**	**29 640 (27 950 to 31 427)**	**32 049 (29 449 to 34 986)**	**1 692.4 (1 605.9 to 1 785.9)**	**796.1 (733.4 to 865.8)**
Road injuries	951 (700 to 1 259)	3 176 (2 290 to 4 267)	76.5 (56.2 to 101.0)	85.2 (61.5 to 113.7)	29 155 (27 485 to 30 898)	31 150 (28 664 to 34 020)	1 663.2 (1 578.4 to 1 753.5)	773.8 (712.0 to 843.1)
Pedestrian road injuries	259 (179 to 341)	916 (659 to 1 260)	20.8 (14.9 to 27.9)	25.1 (17.7 to 34.2)	9 880 (9 058 to 10 837)	10 815 (9 849 to 11 940)	609.1 (566.1 to 657.3)	276.7 (252.4 to 304.7)
Cyclist road injuries	84 (59 to 110)	255 (178 to 339)	6.4 (4.5 to 8.4)	6.8 (4.8 to 9.1)	620 (558 to 691)	1 219 (1 075 to 1 380)	37.6 (34.0 to 41.6)	30.9 (27.3 to 34.9)
Motorcyclist road injuries	216 (152 to 294)	468 (317 to 637)	16.9 (11.9 to 23.1)	12.2 (8.3 to 16.5)	5 493 (4 931 to 6 151)	5 280 (4 670 to 5 988)	298.4 (270.5 to 330.7)	126.3 (112.2 to 143.4)
Motor vehicle road injuries	313 (229 to 416)	1 337 (959 to 1 800)	25.8 (18.9 to 34.2)	35.5 (25.4 to 47.5)	12 865 (11 775 to 13 929)	13 378 (12 057 to 14 847)	699.4 (646.4 to 751.5)	328.0 (295.9 to 363.1)
Other road injuries	85 (61 to 116)	201 (142 to 277)	6.6 (4.8 to 9.0)	5.5 (3.9 to 7.6)	297 (253 to 349)	458 (385 to 548)	18.6 (15.9 to 21.7)	11.9 (9.9 to 14.2)
Other transport injuries	68 (47 to 93)	76 (54 to 103)	4.7 (3.3 to 6.3)	2.1 (1.5 to 2.8)	484 (436 to 541)	899 (787 to 1 032)	29.2 (26.6 to 32.4)	22.3 (19.7 to 25.5)
**Unintentional injuries**	**4 527 (3 278 to 6 041)**	**9 791 (6 940 to 13 460)**	**370.4 (267.6 to 496.2)**	**284.7 (202.0 to 389.2)**	**38 506 (35 415 to 41 829)**	**42 185 (37 964 to 46 417)**	**2 398.9 (2 222.4 to 2 580.4)**	**1 164.2 (1 046.7 to 1 286.2)**
Falls	2 476 (1 835 to 3 255)	6 854 (4 918 to 9 129)	216.9 (158.7 to 283.6)	205.2 (146.6 to 273.5)	6 169 (5 440 to 7 020)	12 341 (10 142 to 14 704)	525.7 (464.2 to 594.9)	364.9 (302.1 to 433.9)
Drowning	31 (22 to 44)	31 (22 to 44)	2.6 (1.8 to 3.6)	0.9 (0.6 to 1.2)	9 031 (8 234 to 9 885)	6 042 (5 483 to 6 632)	487.7 (450.8 to 527.3)	154.4 (140.2 to 169.2)
Fire, heat, and hot substances	601 (372 to 940)	779 (454 to 1 269)	41.5 (25.4 to 64.6)	20.7 (12.1 to 33.7)	4 090 (3 630 to 4 606)	3 249 (2 783 to 3 818)	257.0 (232.0 to 287.6)	88.6 (76.5 to 104.0)
Poisonings	89 (55 to 135)	124 (73 to 196)	5.4 (3.4 to 8.1)	3.2 (1.9 to 5.0)	1 907 (1 725 to 2 104)	1 561 (1 396 to 1 713)	106.0 (97.2 to 115.7)	40.1 (36.1 to 43.9)
Poisoning by carbon monoxide	13 (8 to 19)	15 (9 to 24)	0.8 (0.5 to 1.2)	0.4 (0.2 to 0.6)	930 (833 to 1 040)	818 (718 to 913)	51.5 (46.9 to 56.4)	20.8 (18.4 to 23.1)
Poisoning by other means	77 (47 to 117)	109 (62 to 174)	4.6 (2.9 to 7.1)	2.8 (1.6 to 4.5)	977 (870 to 1 101)	743 (655 to 833)	54.5 (49.1 to 60.6)	19.3 (17.1 to 21.6)
Exposure to mechanical forces	755 (520 to 1 082)	1 052 (701 to 1 547)	60.4 (41.6 to 86.9)	28.3 (19.0 to 41.7)	5 537 (5 097 to 6 023)	5 080 (4 525 to 5 687)	343.0 (315.1 to 374.1)	127.9 (113.9 to 144.0)
Unintentional firearm injuries	109 (79 to 164)	108 (69 to 170)	8.7 (5.7 to 13.3)	2.9 (1.9 to 4.6)	2 502 (2 246 to 2 986)	1 893 (1 653 to 2 165)	145.1 (131.5 to 173.6)	46.0 (40.3 to 52.4)
Other exposure to mechanical forces	645 (441 to 931)	943 (631 to 1 389)	51.7 (35.2 to 74.4)	25.4 (17.0 to 37.5)	3 035 (2 443 to 3 424)	3 187 (2 758 to 3 715)	197.9 (162.1 to 224.4)	81.9 (71.1 to 95.5)
Adverse effects of medical treatment	19 (12 to 28)	60 (37 to 90)	1.4 (0.9 to 2.1)	1.7 (1.0 to 2.5)	1 382 (990 to 1 577)	1 980 (1 677 to 2 244)	97.5 (71.0 to 108.9)	58.2 (49.9 to 65.7)
Animal contact	88 (59 to 126)	131 (85 to 189)	6.2 (4.1 to 8.8)	3.5 (2.3 to 5.1)	357 (303 to 417)	281 (232 to 347)	21.1 (18.1 to 24.4)	7.7 (6.4 to 9.5)
Venomous animal contact	49 (31 to 73)	69 (42 to 104)	3.1 (2.0 to 4.5)	1.8 (1.1 to 2.7)	185 (148 to 234)	116 (87 to 156)	10.1 (8.2 to 12.6)	3.1 (2.4 to 4.2)
Non-venomous animal contact	40 (24 to 63)	62 (36 to 103)	3.1 (1.9 to 4.9)	1.8 (1.0 to 2.9)	172 (138 to 205)	165 (129 to 211)	11.0 (9.0 to 13.3)	4.6 (3.6 to 5.9)
Foreign body	187 (122 to 279)	317 (192 to 512)	14.3 (9.5 to 21.1)	9.2 (5.5 to 14.7)	7 916 (6 833 to 9 262)	9 625 (8 490 to 10 996)	422.1 (373.7 to 482.3)	270.6 (236.9 to 311.9)
Pulmonary aspiration and foreign body in airway	11 (7 to 15)	8 (6 to 11)	0.7 (0.4 to 0.9)	0.2 (0.1 to 0.3)	7 319 (6 258 to 8 601)	9 011 (7 897 to 10 388)	385.3 (337.6 to 442.9)	254.1 (221.2 to 295.6)
Foreign body in eyes	72 (35 to 135)	207 (105 to 378)	6.6 (3.5 to 12.0)	6.2 (3.3 to 11.1)	72 (35 to 135)	207 (105 to 378)	6.6 (3.5 to 12.0)	6.2 (3.3 to 11.1)
Foreign body in other body part	103 (72 to 138)	101 (71 to 136)	7.0 (4.9 to 9.3)	2.7 (1.9 to 3.6)	524 (365 to 605)	407 (338 to 480)	30.1 (21.2 to 34.3)	10.3 (8.7 to 12.1)
Environmental heat and cold exposure	114 (75 to 160)	120 (79 to 174)	9.4 (6.3 to 13.2)	3.5 (2.3 to 5.1)	985 (900 to 1 075)	818 (703 to 927)	73.0 (67.2 to 79.4)	21.5 (18.5 to 24.3)
Exposure to forces of nature	29 (21 to 40)	54 (38 to 76)	1.7 (1.2 to 2.3)	1.3 (1.0 to 1.9)	97 (86 to 110)	85 (69 to 107)	5.5 (4.9 to 6.2)	2.2 (1.8 to 2.7)
Other unintentional injuries	139 (100 to 187)	270 (188 to 378)	10.6 (7.6 to 14.3)	7.4 (5.1 to 10.2)	1 035 (944 to 1 141)	1 123 (989 to 1 279)	60.4 (55.3 to 66.1)	28.0 (24.6 to 31.9)

Death, YLL, incidence, prevalence, YLD, and DALY counts and rates per 100,000 for all injuries in Campeche in 1990 and 2017

Cause	Deaths: All-age counts (95% UI) 1990	Deaths: All-age counts (95% UI) 2017	Deaths: Age-standardised rates per 100,000 (95% UI) 1990	Deaths: Age-standardised rates per 100,000 (95% UI) 2017	Years of life lost: All-age counts (95% UI) 1990	Years of life lost: All-age counts (95% UI) 2017	Years of life lost: Age-standardised rates per 100,000 (95% UI) 1990	Years of life lost: Age-standardised rates per 100,000 (95% UI) 2017
**Transport and unintentional injuries**	**316 (297 to 338)**	**345 (319 to 369)**	**71.3 (68.5 to 74.5)**	**38.9 (36.1 to 41.6)**	**17 775 (16 297 to 19 571)**	**15 067 (13 875 to 16 260)**	**3 156.7 (2 961.4 to 3 391.3)**	**1 583.0 (1 459.2 to 1 705.8)**
**Transport injuries**	**145 (137 to 154)**	**177 (158 to 194)**	**31.2 (29.8 to 32.7)**	**19.0 (17.0 to 20.7)**	**8 145 (7 519 to 8 852)**	**8 498 (7 598 to 9 292)**	**1 483.9 (1 394.2 to 1 583.3)**	**873.1 (783.1 to 954.1)**
Road injuries	134 (126 to 142)	158 (141 to 173)	28.7 (27.4 to 30.2)	16.9 (15.3 to 18.5)	7 510 (6 923 to 8 160)	7 582 (6 831 to 8 306)	1 367.5 (1 282.8 to 1 459.4)	779.2 (703.1 to 851.3)
Pedestrian road injuries	65 (60 to 70)	67 (61 to 74)	15.1 (14.2 to 16.0)	7.5 (6.8 to 8.3)	3 521 (3 134 to 3 950)	2 893 (2 593 to 3 201)	650.6 (596.3 to 709.5)	303.3 (272.9 to 335.7)
Cyclist road injuries	5 (4 to 5)	9 (8 to 10)	1.0 (0.9 to 1.1)	1.0 (0.8 to 1.1)	266 (235 to 301)	407 (349 to 462)	49.3 (43.8 to 54.8)	42.3 (36.3 to 47.9)
Motorcyclist road injuries	22 (20 to 24)	25 (22 to 29)	4.2 (3.8 to 4.6)	2.5 (2.2 to 2.9)	1 294 (1 149 to 1 441)	1 390 (1 213 to 1 596)	229.6 (206.3 to 253.6)	139.0 (121.1 to 159.2)
Motor vehicle road injuries	38 (35 to 42)	52 (45 to 59)	7.7 (7.1 to 8.3)	5.4 (4.7 to 6.1)	2 212 (2 004 to 2 437)	2 692 (2 322 to 3 010)	399.2 (365.8 to 436.4)	273.4 (236.1 to 305.5)
Other road injuries	4 (3 to 4)	4 (4 to 5)	0.8 (0.7 to 0.9)	0.4 (0.4 to 0.5)	217 (183 to 255)	199 (170 to 231)	38.7 (32.6 to 44.6)	20.5 (17.5 to 23.7)
Other transport injuries	11 (10 to 13)	20 (17 to 22)	2.5 (2.3 to 2.7)	2.1 (1.8 to 2.4)	635 (567 to 707)	916 (782 to 1 054)	116.3 (105.1 to 128.5)	94.1 (80.8 to 107.9)
**Unintentional injuries**	**170 (158 to 185)**	**167 (157 to 179)**	**40.1 (38.2 to 42.2)**	**19.9 (18.6 to 21.2)**	**9 630 (8 619 to 10 868)**	**6 569 (6 081 to 7 116)**	**1 672.9 (1 541.8 to 1 830.8)**	**709.7 (657.7 to 765.9)**
Falls	30 (29 to 32)	50 (46 to 54)	11.0 (10.4 to 11.6)	6.6 (6.0 to 7.1)	1 083 (994 to 1 183)	1 230 (1 132 to 1 335)	255.4 (239.7 to 271.2)	141.8 (130.4 to 153.5)
Drowning	49 (44 to 55)	34 (31 to 37)	9.3 (8.6 to 10.1)	3.6 (3.3 to 4.0)	3 094 (2 709 to 3 572)	1 714 (1 547 to 1 894)	505.3 (454.0 to 565.7)	178.8 (161.6 to 197.5)
Fire, heat, and hot substances	11 (10 to 13)	9 (8 to 10)	2.7 (2.5 to 3.0)	1.1 (1.0 to 1.2)	635 (529 to 778)	297 (272 to 325)	107.1 (93.5 to 124.6)	32.8 (30.0 to 35.9)
Poisonings	7 (7 to 8)	5 (5 to 6)	1.4 (1.3 to 1.6)	0.6 (0.6 to 0.7)	484 (420 to 570)	261 (235 to 290)	77.1 (68.6 to 88.3)	27.5 (24.8 to 30.4)
Poisoning by carbon monoxide	2 (2 to 2)	2 (1 to 2)	0.4 (0.3 to 0.4)	0.2 (0.2 to 0.2)	110 (94 to 130)	75 (65 to 87)	18.4 (16.0 to 21.3)	7.9 (6.8 to 9.0)
Poisoning by other means	6 (5 to 7)	4 (4 to 4)	1.1 (1.0 to 1.2)	0.4 (0.4 to 0.5)	374 (320 to 444)	185 (168 to 206)	58.7 (51.6 to 67.7)	19.6 (17.7 to 21.8)
Exposure to mechanical forces	19 (18 to 21)	14 (13 to 15)	4.2 (4.0 to 4.5)	1.6 (1.4 to 1.7)	1 077 (982 to 1 184)	627 (564 to 692)	197.6 (182.8 to 214.5)	65.1 (58.8 to 71.6)
Unintentional firearm injuries	7 (6 to 9)	4 (3 to 4)	1.5 (1.3 to 1.8)	0.4 (0.3 to 0.5)	421 (368 to 500)	189 (158 to 221)	75.5 (66.7 to 90.3)	19.2 (16.0 to 22.3)
Other exposure to mechanical forces	12 (11 to 13)	10 (9 to 11)	2.7 (2.4 to 3.0)	1.1 (1.0 to 1.3)	656 (571 to 738)	438 (390 to 485)	122.1 (106.9 to 135.3)	45.9 (40.9 to 50.8)
Adverse effects of medical treatment	11 (7 to 13)	17 (14 to 19)	3.1 (2.9 to 3.5)	2.1 (1.8 to 2.4)	551 (451 to 671)	547 (460 to 629)	100.7 (87.5 to 116.9)	61.9 (52.0 to 70.4)
Animal contact	6 (5 to 7)	3 (3 to 4)	1.2 (1.0 to 1.4)	0.4 (0.4 to 0.5)	412 (328 to 527)	139 (124 to 156)	62.4 (51.7 to 77.1)	15.3 (13.7 to 17.2)
Venomous animal contact	5 (4 to 6)	3 (2 to 3)	1.0 (0.8 to 1.1)	0.3 (0.3 to 0.3)	356 (277 to 464)	101 (87 to 116)	52.7 (42.5 to 66.7)	11.2 (9.6 to 12.9)
Non-venomous animal contact	1 (1 to 1)	1 (1 to 1)	0.2 (0.2 to 0.3)	0.1 (0.1 to 0.1)	56 (42 to 71)	37 (31 to 50)	9.6 (7.2 to 11.7)	4.0 (3.4 to 5.5)
Foreign body	22 (19 to 26)	23 (21 to 26)	4.1 (3.8 to 4.6)	2.6 (2.4 to 2.9)	1 525 (1 292 to 1 823)	1 208 (1 052 to 1 405)	229.0 (199.4 to 266.6)	130.4 (113.3 to 151.8)
Pulmonary aspiration and foreign body in airway	21 (19 to 25)	23 (21 to 25)	4.0 (3.6 to 4.4)	2.6 (2.4 to 2.9)	1 468 (1 243 to 1 752)	1 179 (1 025 to 1 379)	220.0 (191.5 to 256.2)	127.4 (110.6 to 148.6)
Foreign body in eyes								
Foreign body in other body part	1 (1 to 1)	1 (0 to 1)	0.1 (0.1 to 0.2)	0.1 (0.0 to 0.1)	57 (44 to 71)	29 (23 to 35)	8.9 (7.1 to 10.8)	3.0 (2.4 to 3.6)
Environmental heat and cold exposure	3 (3 to 3)	2 (2 to 2)	0.9 (0.8 to 0.9)	0.3 (0.2 to 0.3)	149 (132 to 169)	78 (70 to 86)	29.9 (27.4 to 32.7)	8.4 (7.5 to 9.2)
Exposure to forces of nature	0 (0 to 0)	0 (0 to 0)	0.1 (0.1 to 0.1)	0.0 (0.0 to 0.0)	17 (15 to 19)	8 (7 to 8)	2.9 (2.6 to 3.1)	0.8 (0.7 to 0.9)
Other unintentional injuries	10 (9 to 11)	9 (8 to 10)	2.1 (1.9 to 2.2)	1.0 (0.9 to 1.1)	608 (541 to 666)	460 (404 to 523)	105.6 (95.5 to 115.8)	46.8 (41.2 to 53.1)

Cause	Incidence: All-age counts (95% UI) 1990	Incidence: All-age counts (95% UI) 2017	Incidence: Age-standardised rates per 100,000 (95% UI) 1990	Incidence: Age-standardised rates per 100,000 (95% UI) 2017	Prevalence: All-age counts (95% UI) 1990	Prevalence: All-age counts (95% UI) 2017	Prevalence: Age-standardised rates per 100,000 (95% UI) 1990	Prevalence: Age-standardised rates per 100,000 (95% UI) 2017
**Transport and unintentional injuries**	**20 576 (19 141 to 22 123)**	**41 278 (37 963 to 44 692)**	**4 278.1 (4 003.5 to 4 573.5)**	**4 575.0 (4 222.7 to 4 941.2)**	**39 778 (37 335 to 42 737)**	**88 213 (82 397 to 95 803)**	**10 193.4 (9 598.1 to 10 951.0)**	**9 943.8 (9 293.6 to 10 803.0)**
**Transport injuries**	**1 927 (1 659 to 2 226)**	**4 830 (4 200 to 5 531)**	**391.9 (340.2 to 443.3)**	**508.1 (443.8 to 578.3)**	**4 727 (4 402 to 5 032)**	**13 580 (12 622 to 14 565)**	**1 260.5 (1 176.2 to 1 341.7)**	**1 510.1 (1 401.2 to 1 619.7)**
Road injuries	1 759 (1 493 to 2 054)	4 664 (4 036 to 5 364)	359.0 (309.9 to 410.7)	489.9 (425.5 to 559.3)	3 998 (3 724 to 4 290)	12 757 (11 859 to 13 744)	1 077.8 (1 005.6 to 1 154.0)	1 417.2 (1 317.5 to 1 526.6)
Pedestrian road injuries	278 (222 to 343)	480 (384 to 590)	60.2 (49.0 to 72.1)	51.5 (41.2 to 62.5)	998 (858 to 1 174)	2 118 (1 838 to 2 470)	274.9 (240.7 to 320.8)	240.2 (208.8 to 279.8)
Cyclist road injuries	334 (260 to 425)	758 (610 to 935)	63.1 (51.0 to 77.0)	81.1 (65.2 to 99.2)	557 (465 to 660)	1 637 (1 385 to 1 914)	140.9 (119.3 to 163.6)	181.2 (154.5 to 210.6)
Motorcyclist road injuries	250 (199 to 309)	647 (508 to 808)	48.4 (39.0 to 58.6)	66.5 (52.3 to 82.7)	871 (752 to 1 004)	2 852 (2 438 to 3 348)	229.6 (199.0 to 265.1)	310.6 (266.4 to 364.2)
Motor vehicle road injuries	603 (492 to 720)	2 174 (1 811 to 2 593)	126.1 (104.6 to 148.6)	225.7 (188.4 to 266.9)	1 039 (913 to 1 209)	4 742 (4 122 to 5 551)	290.4 (253.1 to 337.6)	527.2 (457.8 to 615.4)
Other road injuries	294 (222 to 372)	604 (477 to 739)	61.2 (48.4 to 74.7)	65.1 (51.6 to 79.3)	532 (441 to 638)	1 409 (1 177 to 1 699)	142.0 (118.6 to 169.2)	158.0 (132.2 to 189.7)
Other transport injuries	167 (138 to 212)	166 (139 to 209)	32.9 (28.1 to 39.4)	18.3 (14.6 to 23.0)	729 (607 to 853)	823 (703 to 951)	182.7 (155.6 to 211.3)	93.0 (79.8 to 106.7)
**Unintentional injuries**	**18 649 (17 246 to 20 202)**	**36 449 (33 331 to 39 832)**	**3 886.2 (3 606.6 to 4 191.8)**	**4 066.9 (3 722.6 to 4 423.8)**	**35 051 (32 631 to 37 884)**	**74 633 (68 896 to 81 989)**	**8 932.9 (8 350.0 to 9 667.3)**	**8 433.6 (7 809.7 to 9 257.7)**
Falls	7 912 (6 879 to 9 053)	18 896 (16 747 to 21 393)	1 842.7 (1 637.5 to 2 068.2)	2 187.3 (1 937.7 to 2 465.9)	13 555 (11 192 to 15 481)	37 076 (33 027 to 42 165)	3 701.2 (3 311.9 to 4 223.1)	4 299.7 (3 835.3 to 4 904.0)
Drowning	40 (35 to 47)	30 (24 to 39)	7.7 (6.7 to 8.7)	3.3 (2.7 to 4.2)	182 (159 to 211)	183 (160 to 211)	49.0 (43.4 to 56.4)	21.0 (18.5 to 24.3)
Fire, heat, and hot substances	706 (562 to 887)	771 (633 to 940)	109.0 (89.3 to 133.1)	83.4 (68.4 to 101.5)	6 570 (5 417 to 8 366)	8 811 (7 302 to 11 367)	1 447.3 (1 207.3 to 1 783.0)	955.6 (795.5 to 1 227.2)
Poisonings	261 (195 to 337)	336 (254 to 433)	37.0 (28.2 to 46.9)	36.4 (27.4 to 46.8)	220 (171 to 269)	384 (286 to 477)	42.8 (33.6 to 52.1)	40.7 (30.4 to 50.5)
Poisoning by carbon monoxide	51 (33 to 77)	68 (39 to 106)	8.0 (5.6 to 11.4)	7.4 (4.3 to 11.5)	34 (27 to 44)	55 (42 to 72)	6.9 (5.4 to 8.8)	5.9 (4.5 to 7.7)
Poisoning by other means	210 (154 to 278)	268 (193 to 355)	28.9 (21.6 to 37.5)	29.0 (20.8 to 38.6)	185 (137 to 232)	329 (237 to 416)	35.8 (26.7 to 44.6)	34.8 (25.1 to 44.0)
Exposure to mechanical forces	5 020 (4 316 to 5 859)	8 684 (7 479 to 10 007)	936.6 (819.4 to 1 076.1)	912.5 (787.1 to 1 047.6)	9 606 (8 387 to 11 075)	21 159 (18 291 to 24 802)	2 489.4 (2 187.2 to 2 868.9)	2 330.5 (2 014.4 to 2 722.0)
Unintentional firearm injuries	242 (177 to 327)	255 (180 to 340)	44.1 (33.2 to 58.0)	26.5 (18.8 to 35.0)	939 (742 to 1 136)	1 335 (1 083 to 1 606)	248.8 (198.9 to 303.3)	148.7 (121.7 to 178.6)
Other exposure to mechanical forces	4 779 (4 093 to 5 575)	8 429 (7 281 to 9 718)	892.6 (778.7 to 1 025.7)	886.0 (766.4 to 1 018.0)	8 667 (7 383 to 10 202)	19 824 (16 856 to 23 559)	2 240.6 (1 920.6 to 2 628.0)	2 181.8 (1 853.9 to 2 582.3)
Adverse effects of medical treatment	411 (341 to 485)	886 (688 to 1 029)	95.9 (81.0 to 111.2)	93.2 (77.5 to 119.6)	31 (24 to 39)	64 (48 to 83)	7.3 (5.6 to 9.2)	7.1 (5.4 to 9.3)
Animal contact	1 599 (1 348 to 1 924)	1 992 (1 740 to 2 290)	307.3 (266.7 to 356.2)	218.0 (191.4 to 249.2)	1 640 (1 410 to 1 898)	2 448 (2 136 to 2 808)	405.7 (355.4 to 459.1)	273.3 (239.1 to 313.7)
Venomous animal contact	582 (450 to 750)	650 (514 to 806)	111.9 (88.3 to 139.4)	71.0 (56.5 to 87.8)	382 (315 to 457)	445 (376 to 517)	88.0 (74.1 to 102.3)	48.9 (41.3 to 56.6)
Non-venomous animal contact	1 017 (832 to 1 250)	1 341 (1 148 to 1 591)	195.4 (163.1 to 234.4)	147.0 (125.5 to 173.8)	1 258 (1 039 to 1 514)	2 002 (1 695 to 2 361)	317.7 (268.3 to 372.3)	224.9 (191.4 to 263.8)
Foreign body	1 319 (774 to 1 923)	3 373 (1 835 to 5 376)	281.2 (149.8 to 431.3)	365.5 (195.3 to 575.6)	668 (528 to 859)	1 165 (856 to 1 608)	156.4 (126.3 to 197.6)	131.5 (98.1 to 180.0)
Pulmonary aspiration and foreign body in airway	58 (40 to 80)	36 (26 to 49)	8.6 (6.3 to 11.4)	4.0 (2.8 to 5.4)	39 (27 to 54)	31 (25 to 39)	7.3 (5.7 to 10.5)	3.4 (2.8 to 4.2)
Foreign body in eyes	1 120 (602 to 1 719)	3 210 (1 660 to 5 212)	250.0 (117.0 to 402.0)	347.4 (176.4 to 558.2)	216 (110 to 378)	686 (392 to 1 123)	56.3 (32.7 to 89.6)	73.3 (46.5 to 124.5)
Foreign body in other body part	135 (88 to 207)	128 (95 to 166)	22.7 (15.6 to 32.6)	14.1 (10.5 to 18.1)	412 (328 to 526)	447 (376 to 530)	91.8 (74.8 to 114.6)	49.8 (42.1 to 59.1)
Environmental heat and cold exposure	268 (187 to 366)	206 (151 to 288)	52.6 (40.8 to 65.7)	23.3 (17.2 to 32.1)	917 (738 to 1 118)	797 (675 to 951)	221.3 (184.9 to 258.8)	90.5 (76.9 to 108.0)
Exposure to forces of nature	44 (36 to 55)	10 (9 to 12)	7.3 (6.1 to 8.8)	1.1 (0.9 to 1.3)	143 (102 to 196)	337 (239 to 466)	27.0 (19.8 to 36.8)	35.6 (25.2 to 49.0)
Other unintentional injuries	1 074 (895 to 1 299)	1 312 (1 115 to 1 546)	208.9 (177.7 to 244.1)	142.9 (121.4 to 167.3)	1 520 (1 311 to 1 759)	2 209 (1 921 to 2 517)	385.5 (334.9 to 441.8)	247.5 (215.9 to 280.7)

Cause	Years lived with disability: All-age counts (95% UI) 1990	Years lived with disability: All-age counts (95% UI) 2017	Years lived with disability: Age-standardised rates per 100,000 (95% UI) 1990	Years lived with disability: Age-standardised rates per 100,000 (95% UI) 2017	Disability adjusted life years: All-age counts (95% UI) 1990	Disability adjusted life years: All-age counts (95% UI) 2017	Disability adjusted life years: Age-standardised rates per 100,000 (95% UI) 1990	Disability adjusted life years: Age-standardised rates per 100,000 (95% UI) 2017
**Transport and unintentional injuries**	**1 732 (1 268 to 2 296)**	**3 620 (2 584 to 4 894)**	**437.4 (317.9 to 578.0)**	**408.0 (291.9 to 551.1)**	**19 507 (17 890 to 21 351)**	**18 686 (17 171 to 20 373)**	**3 594.1 (3 349.5 to 3 869.3)**	**1 991.0 (1 826.6 to 2 178.6)**
**Transport injuries**	**323 (235 to 426)**	**818 (596 to 1 092)**	**82.6 (60.4 to 108.7)**	**89.7 (65.4 to 119.7)**	**8 468 (7 848 to 9 172)**	**9 316 (8 417 to 10 156)**	**1 566.5 (1 473.4 to 1 665.5)**	**963.0 (870.4 to 1 052.0)**
Road injuries	276 (209 to 367)	775 (561 to 1 035)	71.6 (52.5 to 94.7)	84.9 (61.6 to 113.3)	7 786 (7 192 to 8 449)	8 356 (7 557 to 9 126)	1 439.1 (1 351.1 to 1 533.1)	864.1 (780.9 to 943.2)
Pedestrian road injuries	72 (51 to 97)	134 (95 to 184)	19.0 (13.5 to 25.6)	15.0 (10.6 to 20.6)	3 593 (3 216 to 4 024)	3 027 (2 719 to 3 330)	669.6 (615.7 to 729.7)	318.3 (287.1 to 350.4)
Cyclist road injuries	39 (28 to 51)	98 (68 to 131)	9.5 (6.7 to 12.4)	10.7 (7.5 to 14.3)	305 (271 to 339)	504 (440 to 568)	58.8 (52.7 to 64.9)	53.0 (46.4 to 59.6)
Motorcyclist road injuries	61 (43 to 82)	174 (120 to 237)	15.5 (10.8 to 20.9)	18.7 (12.9 to 25.5)	1 354 (1 210 to 1 507)	1 564 (1 371 to 1 777)	245.1 (221.7 to 269.5)	157.7 (138.4 to 179.1)
Motor vehicle road injuries	75 (54 to 99)	298 (216 to 399)	19.9 (14.4 to 26.3)	32.7 (23.6 to 44.0)	2 287 (2 082 to 2 511)	2 991 (2 621 to 3 335)	419.2 (385.1 to 455.7)	306.1 (268.3 to 340.9)
Other road injuries	30 (22 to 41)	70 (50 to 97)	7.8 (5.6 to 10.4)	7.8 (5.6 to 10.7)	248 (211 to 287)	269 (232 to 311)	46.5 (40.2 to 52.8)	28.3 (24.4 to 32.6)
Other transport injuries	46 (33 to 62)	43 (31 to 58)	11.0 (7.9 to 14.6)	4.8 (3.5 to 6.5)	682 (615 to 755)	959 (829 to 1 098)	127.4 (116.1 to 139.4)	99.0 (85.7 to 112.7)
**Unintentional injuries**	**1 409 (1 023 to 1 873)**	**2 802 (1 995 to 3 798)**	**354.8 (256.4 to 471.7)**	**318.3 (226.4 to 431.8)**	**11 039 (9 896 to 12 400)**	**9 371 (8 456 to 10 492)**	**2 027.7 (1 869.3 to 2 216.8)**	**1 028.0 (925.6 to 1 153.6)**
Falls	810 (596 to 1 067)	1 875 (1 353 to 2 487)	214.4 (156.5 to 279.4)	216.7 (155.3 to 289.4)	1 893 (1 650 to 2 163)	3 105 (2 572 to 3 729)	469.9 (411.2 to 537.1)	358.5 (295.8 to 429.9)
Drowning	10 (7 to 14)	10 (7 to 14)	2.7 (1.9 to 3.7)	1.1 (0.7 to 1.5)	3 104 (2 719 to 3 580)	1 728 (1 556 to 1 902)	508.0 (456.8 to 567.9)	179.9 (162.6 to 198.3)
Fire, heat, and hot substances	142 (88 to 225)	171 (101 to 276)	30.3 (18.9 to 48.0)	18.4 (10.9 to 29.7)	778 (654 to 932)	468 (392 to 571)	137.4 (119.1 to 160.8)	51.3 (43.0 to 62.3)
Poisonings	26 (16 to 38)	45 (27 to 66)	4.9 (3.1 to 7.3)	4.7 (2.8 to 7.0)	510 (440 to 595)	305 (275 to 341)	82.0 (72.8 to 93.0)	32.1 (28.9 to 35.8)
Poisoning by carbon monoxide	4 (2 to 6)	6 (3 to 9)	0.7 (0.5 to 1.1)	0.6 (0.4 to 0.9)	114 (97 to 135)	81 (71 to 93)	19.1 (16.7 to 22.0)	8.5 (7.4 to 9.7)
Poisoning by other means	22 (14 to 33)	39 (22 to 59)	4.2 (2.6 to 6.3)	4.1 (2.4 to 6.2)	396 (339 to 469)	224 (200 to 253)	62.8 (55.0 to 72.3)	23.7 (21.1 to 26.7)
Exposure to mechanical forces	227 (158 to 324)	456 (311 to 667)	57.5 (39.9 to 82.1)	50.0 (34.1 to 73.2)	1 304 (1 180 to 1 445)	1 083 (922 to 1 288)	255.1 (231.0 to 282.8)	115.1 (98.0 to 137.5)
Unintentional firearm injuries	19 (12 to 28)	24 (15 to 38)	4.7 (3.1 to 7.4)	2.6 (1.7 to 4.2)	440 (387 to 516)	213 (177 to 245)	80.3 (71.5 to 94.6)	21.9 (18.2 to 25.2)
Other exposure to mechanical forces	209 (145 to 298)	432 (293 to 632)	52.8 (36.4 to 75.3)	47.4 (32.1 to 69.6)	864 (754 to 984)	870 (722 to 1 061)	174.8 (152.5 to 200.0)	93.2 (77.0 to 114.5)
Adverse effects of medical treatment	4 (3 to 6)	9 (5 to 13)	1.0 (0.6 to 1.5)	1.0 (0.6 to 1.5)	555 (456 to 675)	555 (468 to 630)	101.7 (88.5 to 117.8)	62.9 (52.9 to 71.9)
Animal contact	44 (29 to 61)	55 (36 to 78)	9.7 (6.5 to 13.7)	6.0 (3.9 to 8.6)	456 (370 to 570)	193 (168 to 221)	72.0 (60.8 to 85.9)	21.2 (18.5 to 24.2)
Venomous animal contact	24 (15 to 35)	26 (17 to 39)	4.8 (3.1 to 7.0)	2.8 (1.8 to 4.1)	380 (300 to 490)	128 (110 to 146)	57.6 (47.1 to 71.3)	14.0 (12.0 to 16.0)
Non-venomous animal contact	19 (12 to 31)	28 (17 to 46)	4.8 (3.0 to 7.7)	3.2 (1.9 to 5.1)	76 (57 to 96)	65 (52 to 86)	14.5 (10.9 to 18.5)	7.2 (5.7 to 9.5)
Foreign body	42 (28 to 60)	68 (42 to 106)	9.6 (6.5 to 13.5)	7.6 (4.8 to 11.8)	1 567 (1 333 to 1 865)	1 276 (1 116 to 1 471)	238.6 (208.9 to 276.2)	138.1 (120.6 to 159.8)
Pulmonary aspiration and foreign body in airway	2 (1 to 3)	1 (1 to 2)	0.4 (0.3 to 0.5)	0.1 (0.1 to 0.2)	1 471 (1 245 to 1 755)	1 180 (1 027 to 1 375)	220.4 (191.8 to 256.6)	127.6 (110.8 to 148.8)
Foreign body in eyes	14 (7 to 26)	46 (25 to 81)	3.8 (2.1 to 6.5)	5.2 (2.9 to 9.1)	14 (7 to 26)	46 (25 to 81)	3.8 (2.1 to 6.5)	5.2 (2.9 to 9.1)
Foreign body in other body part	25 (18 to 34)	21 (15 to 27)	5.9 (3.8 to 7.0)	2.3 (1.6 to 3.0)	82 (68 to 97)	50 (40 to 75)	14.3 (11.9 to 16.8)	5.3 (4.3 to 7.3)
Environmental heat and cold exposure	36 (24 to 51)	30 (20 to 44)	8.7 (5.8 to 12.2)	3.4 (2.2 to 5.0)	185 (164 to 211)	108 (95 to 125)	38.5 (34.8 to 43.3)	11.9 (10.4 to 13.6)
Exposure to forces of nature	8 (5 to 11)	11 (8 to 15)	1.4 (1.0 to 2.0)	1.2 (0.9 to 1.6)	25 (22 to 28)	19 (16 to 23)	4.3 (3.8 to 4.9)	2.0 (1.7 to 2.4)
Other unintentional injuries	59 (42 to 80)	74 (52 to 101)	14.7 (10.4 to 19.8)	8.2 (5.8 to 11.3)	668 (599 to 729)	534 (475 to 602)	120.2 (109.6 to 131.6)	55.1 (48.0 to 61.9)

Death, YLL, incidence, prevalence, YLD, and DALY counts and rates per 100,000 for all injuries in Chiapas in 1990 and 2017

Cause	Deaths: All-age counts (95% UI) 1990	Deaths: All-age counts (95% UI) 2017	Deaths: Age-standardised rates per 100,000 (95% UI) 1990	Deaths: Age-standardised rates per 100,000 (95% UI) 2017	Years of life lost: All-age counts (95% UI) 1990	Years of life lost: All-age counts (95% UI) 2017	Years of life lost: Age-standardised rates per 100,000 (95% UI) 1990	Years of life lost: Age-standardised rates per 100,000 (95% UI) 2017
**Transport and unintentional injuries**	**1 663 (1 583 to 1 752)**	**1 849 (1 726 to 1 979)**	**63.0 (60.7 to 65.2)**	**36.3 (33.9 to 38.7)**	**97 344 (91 241 to 104 387)**	**90 003 (83 302 to 97 195)**	**2 890.8 (2 754.7 to 3 041.0)**	**1 549.5 (1 440.0 to 1 667.8)**
**Transport injuries**	**559 (525 to 591)**	**801 (728 to 875)**	**20.1 (19.0 to 21.1)**	**14.8 (13.4 to 16.1)**	**32 677 (30 360 to 34 786)**	**41 426 (37 555 to 45 379)**	**994.8 (936.9 to 1 050.9)**	**708.5 (643.2 to 775.7)**
Road injuries	537 (504 to 568)	766 (696 to 837)	19.3 (18.3 to 20.3)	14.1 (12.8 to 15.4)	31 353 (29 131 to 33 414)	39 619 (35 913 to 43 478)	956.2 (899.6 to 1 010.6)	677.4 (614.6 to 742.0)
Pedestrian road injuries	228 (210 to 245)	311 (281 to 339)	9.0 (8.4 to 9.6)	6.1 (5.5 to 6.6)	12 892 (11 794 to 14 117)	14 627 (13 155 to 16 073)	401.1 (368.6 to 432.1)	258.1 (232.1 to 283.5)
Cyclist road injuries	12 (11 to 14)	25 (22 to 28)	0.4 (0.4 to 0.5)	0.5 (0.4 to 0.5)	780 (643 to 824)	1 228 (1 060 to 1 390)	21.9 (19.6 to 24.3)	21.3 (18.6 to 24.1)
Motorcyclist road injuries	91 (81 to 101)	133 (115 to 153)	2.9 (2.6 to 3.3)	2.2 (1.9 to 2.6)	5 469 (4 863 to 6 131)	7 714 (6 601 to 8 964)	163.3 (145.8 to 181.5)	126.4 (109.0 to 146.0)
Motor vehicle road injuries	194 (175 to 219)	284 (253 to 318)	6.5 (5.9 to 7.4)	5.1 (4.5 to 5.7)	11 546 (10 336 to 12 961)	15 357 (13 590 to 17 208)	349.1 (314.8 to 393.9)	259.7 (230.3 to 288.5)
Other road injuries	12 (10 to 14)	13 (12 to 16)	0.4 (0.3 to 0.5)	0.2 (0.2 to 0.3)	716 (585 to 838)	693 (590 to 805)	20.8 (17.3 to 23.9)	11.9 (10.2 to 14.1)
Other transport injuries	22 (20 to 24)	35 (31 to 40)	0.8 (0.7 to 0.8)	0.7 (0.6 to 0.7)	1 324 (1 183 to 1 478)	1 806 (1 588 to 2 059)	38.6 (35.0 to 42.3)	31.1 (27.4 to 35.4)
**Unintentional injuries**	**1 104 (1 038 to 1 173)**	**1 048 (987 to 1 115)**	**42.9 (41.1 to 44.8)**	**21.5 (20.3 to 22.8)**	**64 667 (59 846 to 70 012)**	**48 577 (45 316 to 52 267)**	**1 896.0 (1 785.4 to 2 011.2)**	**841.0 (787.2 to 899.3)**
Falls	159 (150 to 169)	258 (239 to 277)	9.7 (9.2 to 10.4)	6.4 (5.9 to 6.8)	6 489 (5 997 to 7 018)	7 769 (7 105 to 8 441)	259.2 (244.3 to 277.7)	155.0 (142.5 to 167.9)
Drowning	248 (225 to 263)	188 (173 to 205)	7.7 (7.2 to 8.2)	3.3 (3.1 to 3.6)	15 577 (14 175 to 17 135)	10 646 (9 611 to 11 752)	419.7 (388.2 to 453.5)	175.1 (158.5 to 192.5)
Fire, heat, and hot substances	66 (59 to 75)	45 (41 to 48)	2.5 (2.3 to 2.7)	1.0 (0.9 to 1.1)	4 186 (3 583 to 4 891)	1 828 (1 645 to 2 018)	108.9 (96.9 to 123.4)	32.6 (29.6 to 35.7)
Poisonings	70 (64 to 76)	50 (45 to 55)	2.2 (2.1 to 2.4)	0.9 (0.8 to 1.0)	4 542 (4 090 to 5 014)	2 682 (2 402 to 2 993)	119.7 (110.0 to 130.0)	44.5 (40.3 to 49.4)
Poisoning by carbon monoxide	10 (9 to 12)	8 (7 to 9)	0.3 (0.3 to 0.3)	0.1 (0.1 to 0.2)	689 (600 to 799)	427 (368 to 487)	17.8 (15.8 to 20.1)	7.1 (6.2 to 8.1)
Poisoning by other means	59 (54 to 65)	42 (38 to 46)	1.9 (1.8 to 2.0)	0.8 (0.7 to 0.8)	3 853 (3 469 to 4 272)	2 255 (2 014 to 2 520)	102.0 (93.4 to 111.1)	37.4 (33.6 to 41.5)
Exposure to mechanical forces	265 (241 to 283)	132 (121 to 144)	9.8 (8.9 to 10.5)	2.6 (2.4 to 2.8)	15 076 (13 689 to 16 188)	6 352 (5 726 to 6 976)	476.6 (432.6 to 510.4)	110.8 (100.2 to 121.2)
Unintentional firearm injuries	141 (126 to 154)	47 (39 to 53)	4.9 (4.4 to 5.4)	0.9 (0.7 to 1.0)	8 191 (7 348 to 8 983)	2 354 (1 948 to 2 694)	253.9 (225.9 to 278.5)	40.5 (33.6 to 46.3)
Other exposure to mechanical forces	124 (96 to 138)	86 (77 to 95)	4.9 (3.7 to 5.5)	1.7 (1.6 to 1.9)	6 884 (5 427 to 7 713)	3 998 (3 576 to 4 505)	222.7 (169.5 to 248.6)	70.2 (63.0 to 78.6)
Adverse effects of medical treatment	55 (42 to 62)	71 (63 to 88)	2.4 (2.0 to 2.6)	1.6 (1.4 to 2.0)	3 209 (2 848 to 3 770)	2 791 (2 415 to 3 568)	88.9 (68.6 to 100.4)	49.9 (44.0 to 63.0)
Animal contact	21 (18 to 24)	12 (11 to 14)	0.6 (0.6 to 0.7)	0.2 (0.2 to 0.3)	1 446 (1 222 to 1 731)	600 (522 to 684)	34.3 (29.9 to 39.9)	10.1 (9.0 to 11.4)
Venomous animal contact	15 (12 to 18)	7 (6 to 9)	0.4 (0.4 to 0.5)	0.1 (0.1 to 0.2)	1 058 (865 to 1 301)	368 (280 to 497)	24.3 (20.5 to 28.9)	6.2 (4.7 to 7.2)
Non-venomous animal contact	6 (5 to 7)	5 (4 to 7)	0.2 (0.2 to 0.2)	0.1 (0.1 to 0.1)	387 (319 to 463)	231 (194 to 321)	10.1 (8.6 to 11.8)	4.0 (3.4 to 5.5)
Foreign body	138 (126 to 153)	143 (129 to 159)	4.4 (4.1 to 4.7)	2.6 (2.4 to 2.9)	9 231 (8 195 to 10 451)	8 240 (7 234 to 9 506)	233.5 (213.4 to 256.1)	132.5 (117.4 to 150.3)
Pulmonary aspiration and foreign body in airway	129 (117 to 142)	138 (125 to 154)	4.2 (3.9 to 4.5)	2.6 (2.3 to 2.8)	8 598 (7 629 to 9 702)	7 944 (6 941 to 9 186)	218.0 (199.0 to 239.3)	127.6 (113.2 to 145.3)
Foreign body in eyes								
Foreign body in other body part	9 (6 to 11)	5 (4 to 7)	0.2 (0.2 to 0.3)	0.1 (0.1 to 0.1)	633 (408 to 764)	296 (247 to 421)	15.5 (10.8 to 18.2)	4.8 (4.1 to 6.9)
Environmental heat and cold exposure	41 (39 to 43)	13 (12 to 15)	2.0 (1.9 to 2.1)	0.3 (0.3 to 0.3)	2 010 (1 869 to 2 178)	548 (492 to 603)	73.0 (69.6 to 76.6)	10.2 (9.2 to 11.3)
Exposure to forces of nature	2 (2 to 2)	99 (91 to 107)	0.1 (0.1 to 0.1)	1.9 (1.7 to 2.0)	105 (94 to 116)	5 106 (4 663 to 5 616)	3.0 (2.8 to 3.3)	86.5 (79.2 to 94.5)
Other unintentional injuries	45 (42 to 49)	37 (33 to 42)	1.5 (1.4 to 1.6)	0.7 (0.6 to 0.7)	2 802 (2 548 to 3 078)	2 015 (1 799 to 2 284)	79.1 (72.5 to 85.9)	33.8 (30.3 to 38.2)

Cause	Incidence: All-age counts (95% UI) 1990	Incidence: All-age counts (95% UI) 2017	Incidence: Age-standardised rates per 100,000 (95% UI) 1990	Incidence: Age-standardised rates per 100,000 (95% UI) 2017	Prevalence: All-age counts (95% UI) 1990	Prevalence: All-age counts (95% UI) 2017	Prevalence: Age-standardised rates per 100,000 (95% UI) 1990	Prevalence: Age-standardised rates per 100,000 (95% UI) 2017
**Transport and unintentional injuries**	**114 498 (103 981 to 125 608)**	**208 639 (189 154 to 229 318)**	**4 073.9 (3 683.2 to 4 489.4)**	**3 899.3 (3 536.1 to 4 300.4)**	**215 578 (202 070 to 230 894)**	**409 182 (384 343 to 440 637)**	**9 908.1 (9 321.6 to 10 641.5)**	**8 374.7 (7 880.6 to 9 018.0)**
**Transport injuries**	**8 335 (7 030 to 9 750)**	**26 054 (22 454 to 30 218)**	**290.3 (251.9 to 332.2)**	**463.5 (401.0 to 532.3)**	**18 971 (17 713 to 20 196)**	**67 922 (63 292 to 72 822)**	**930.3 (869.3 to 992.9)**	**1 393.2 (1 295.5 to 1 498.5)**
Road injuries	7 916 (6 660 to 9 353)	25 654 (22 039 to 29 840)	277.3 (239.5 to 319.0)	455.9 (393.5 to 525.1)	17 194 (15 988 to 18 345)	66 039 (61 379 to 70 875)	854.0 (792.8 to 914.8)	1 354.4 (1 259.6 to 1 458.9)
Pedestrian road injuries	1 282 (1 023 to 1 595)	3 923 (3 152 to 4 769)	47.8 (39.2 to 57.2)	72.0 (58.3 to 86.9)	4 446 (3 800 to 5 179)	16 224 (14 023 to 18 972)	225.9 (195.6 to 261.4)	338.4 (293.7 to 392.5)
Cyclist road injuries	1 122 (829 to 1 494)	3 090 (2 384 to 3 968)	35.1 (27.4 to 44.7)	53.2 (41.8 to 67.2)	1 755 (1 475 to 2 092)	5 997 (5 022 to 7 116)	79.5 (67.9 to 92.7)	118.9 (100.3 to 139.5)
Motorcyclist road injuries	1 077 (837 to 1 376)	2 886 (2 198 to 3 758)	35.6 (28.3 to 44.3)	49.1 (37.7 to 63.0)	3 628 (3 126 to 4 205)	11 809 (10 045 to 13 856)	175.4 (151.3 to 203.7)	236.8 (201.7 to 278.0)
Motor vehicle road injuries	3 062 (2 477 to 3 683)	12 718 (10 629 to 15 243)	110.9 (91.0 to 130.6)	226.3 (189.3 to 268.9)	5 015 (4 399 to 5 849)	25 625 (22 371 to 29 965)	258.6 (225.5 to 301.1)	528.5 (460.6 to 617.5)
Other road injuries	1 373 (991 to 1 800)	3 037 (2 334 to 3 846)	48.6 (37.8 to 60.5)	55.4 (43.5 to 68.5)	2 351 (1 952 to 2 848)	6 389 (5 357 to 7 681)	115.2 (96.0 to 137.3)	131.8 (110.7 to 157.7)
Other transport injuries	419 (309 to 572)	401 (308 to 523)	13.0 (10.3 to 16.2)	7.5 (5.9 to 9.6)	1 777 (1 467 to 2 114)	1 884 (1 616 to 2 163)	76.3 (64.6 to 88.5)	38.8 (33.7 to 44.1)
**Unintentional injuries**	**106 162 (96 019 to 117 474)**	**182 586 (164 346 to 202 944)**	**3 783.7 (3 393.5 to 4 201.0)**	**3 435.8 (3 084.6 to 3 836.6)**	**196 607 (183 599 to 211 771)**	**341 260 (317 286 to 372 825)**	**8 977.8 (8 396.3 to 9 685.3)**	**6 981.5 (6 518.2 to 7 603.2)**
Falls	38 135 (33 081 to 44 135)	86 918 (76 287 to 98 821)	1 521.1 (1 345.3 to 1 712.1)	1 711.5 (1 510.0 to 1 939.0)	62 689 (55 139 to 71 365)	162 516 (144 708 to 185 851)	3 146.7 (2 813.5 to 3 601.5)	3 494.3 (3 071.2 to 3 921.5)
Drowning	202 (173 to 237)	149 (119 to 185)	6.4 (5.5 to 7.4)	2.7 (2.2 to 3.3)	839 (731 to 975)	822 (719 to 948)	41.7 (36.9 to 47.7)	17.4 (15.3 to 20.1)
Fire, heat, and hot substances	4 167 (3 249 to 5 299)	4 261 (3 336 to 5 311)	101.4 (82.0 to 123.7)	68.8 (55.8 to 84.4)	36 849 (30 511 to 46 314)	44 724 (36 970 to 57 093)	1 437.7 (1 193.8 to 1 724.3)	847.3 (708.1 to 1 060.5)
Poisonings	2 050 (1 574 to 2 603)	3 211 (2 471 to 4 054)	45.9 (36.4 to 56.9)	49.6 (38.6 to 61.9)	1 564 (1 237 to 1 898)	3 023 (2 300 to 3 729)	52.6 (42.4 to 63.4)	53.8 (41.2 to 66.3)
Poisoning by carbon monoxide	341 (240 to 481)	305 (191 to 452)	8.9 (6.7 to 11.8)	5.3 (3.5 to 7.4)	202 (159 to 259)	188 (149 to 237)	7.2 (5.8 to 9.1)	3.5 (2.8 to 4.4)
Poisoning by other means	1 709 (1 291 to 2 208)	2 906 (2 220 to 3 709)	37.1 (28.8 to 46.7)	44.3 (34.3 to 55.8)	1 361 (1 020 to 1 680)	2 834 (2 105 to 3 534)	45.4 (34.6 to 55.9)	50.3 (37.6 to 62.6)
Exposure to mechanical forces	34 227 (29 268 to 40 628)	36 751 (31 595 to 42 553)	1 108.0 (953.9 to 1 290.4)	640.5 (553.3 to 739.1)	61 592 (53 955 to 70 532)	81 415 (71 116 to 93 795)	2 978.7 (2 626.7 to 3 402.5)	1 647.6 (1 448.8 to 1 899.5)
Unintentional firearm injuries	2 972 (2 225 to 3 910)	1 623 (1 199 to 2 129)	95.9 (72.6 to 123.7)	28.7 (21.5 to 37.2)	9 694 (7 764 to 11 748)	7 409 (6 009 to 8 970)	485.1 (392.0 to 591.7)	153.8 (125.1 to 186.8)
Other exposure to mechanical forces	31 255 (26 503 to 37 164)	35 127 (30 288 to 40 720)	1 012.1 (873.9 to 1 183.2)	611.7 (529.7 to 706.5)	51 838 (43 865 to 61 163)	74 006 (63 555 to 87 199)	2 493.6 (2 126.3 to 2 952.8)	1 493.9 (1 289.8 to 1 751.3)
Adverse effects of medical treatment	2 026 (1 681 to 2 380)	4 087 (3 445 to 4 876)	81.4 (69.5 to 94.7)	79.8 (67.7 to 95.2)	155 (120 to 195)	313 (239 to 396)	6.2 (4.8 to 7.7)	6.1 (4.7 to 7.7)
Animal contact	7 781 (6 338 to 9 777)	10 161 (8 720 to 12 021)	246.0 (206.0 to 291.1)	183.2 (156.4 to 211.1)	7 547 (6 449 to 8 829)	11 707 (10 111 to 13 500)	334.9 (292.1 to 382.7)	235.4 (204.5 to 269.7)
Venomous animal contact	2 692 (1 978 to 3 602)	3 061 (2 365 to 3 877)	84.8 (64.8 to 108.7)	54.5 (42.6 to 68.1)	1 605 (1 321 to 1 918)	1 848 (1 564 to 2 156)	64.9 (54.5 to 75.5)	35.6 (30.4 to 41.3)
Non-venomous animal contact	5 089 (3 992 to 6 499)	7 101 (5 946 to 8 586)	161.2 (131.5 to 196.4)	126.6 (107.0 to 151.7)	5 942 (4 893 to 7 176)	9 859 (8 296 to 11 619)	270.0 (226.6 to 318.5)	199.8 (170.2 to 233.2)
Foreign body	11 504 (5 516 to 18 352)	22 248 (10 115 to 37 342)	468.9 (195.3 to 782.8)	442.1 (187.0 to 764.3)	4 426 (3 271 to 6 076)	6 540 (4 421 to 9 744)	199.7 (151.4 to 264.5)	137.6 (94.6 to 199.8)
Pulmonary aspiration and foreign body in airway	261 (183 to 366)	143 (104 to 197)	6.3 (4.8 to 8.5)	2.5 (1.9 to 3.3)	157 (116 to 209)	104 (85 to 130)	5.6 (4.3 to 7.2)	2.0 (1.7 to 2.5)
Foreign body in eyes	10 229 (4 488 to 17 034)	21 534 (9 390 to 35 697)	440.0 (163.7 to 752.2)	429.5 (174.5 to 751.8)	1 954 (939 to 3 450)	4 570 (2 469 to 7 636)	101.1 (57.2 to 161.4)	99.0 (56.3 to 161.6)
Foreign body in other body part	814 (531 to 1 281)	571 (403 to 777)	22.6 (15.6 to 32.4)	10.2 (7.2 to 13.3)	2 315 (1 851 to 2 927)	1 866 (1 541 to 2 273)	93.0 (76.9 to 114.5)	36.6 (30.6 to 44.1)
Environmental heat and cold exposure	1 298 (978 to 1 676)	1 267 (1 040 to 1 500)	44.6 (36.5 to 53.5)	23.6 (19.7 to 27.7)	4 165 (3 377 to 5 059)	4 500 (3 833 to 5 282)	183.9 (155.3 to 216.5)	91.5 (79.4 to 106.4)
Exposure to forces of nature	273 (224 to 342)	6 791 (5 732 to 8 154)	7.7 (6.5 to 9.1)	114.6 (97.9 to 135.6)	10 582 (7 386 to 14 908)	14 879 (10 500 to 20 459)	330.3 (237.8 to 457.1)	291.2 (207.5 to 398.6)
Other unintentional injuries	4 699 (3 875 to 5 848)	6 742 (5 702 to 7 983)	152.2 (128.5 to 180.3)	121.6 (103.0 to 142.6)	6 258 (5 376 to 7 236)	10 821 (9 400 to 12 339)	285.3 (247.4 to 325.3)	219.2 (190.7 to 248.7)

Cause	Years lived with disability: All-age counts (95% UI) 1990	Years lived with disability: All-age counts (95% UI) 2017	Years lived with disability: Age-standardised rates per 100,000 (95% UI) 1990	Years lived with disability: Age-standardised rates per 100,000 (95% UI) 2017	Disability adjusted life years: All-age counts (95% UI) 1990	Disability adjusted life years: All-age counts (95% UI) 2017	Disability adjusted life years: Age-standardised rates per 100,000 (95% UI) 1990	Disability adjusted life years: Age-standardised rates per 100,000 (95% UI) 2017
**Transport and unintentional injuries**	**9 673 (7 168 to 12 640)**	**18 402 (13 516 to 24 405)**	**431.1 (318.2 to 563.4)**	**374.0 (274.8 to 496.3)**	**107 017 (100 184 to 115 018)**	**108 404 (100 241 to 117 389)**	**3 321.9 (3 143.5 to 3 524.3)**	**1 923.5 (1 774.6 to 2 086.1)**
**Transport injuries**	**1 387 (1 013 to 1 819)**	**4 380 (3 186 to 5 834)**	**64.7 (47.2 to 84.6)**	**88.0 (63.9 to 117.0)**	**34 064 (31 699 to 36 283)**	**45 806 (41 888 to 50 176)**	**1 059.5 (998.8 to 1 119.6)**	**796.5 (727.7 to 870.9)**
Road injuries	1 262 (924 to 1 663)	4 271 (3 100 to 5 694)	59.7 (43.6 to 78.2)	85.8 (62.2 to 114.3)	32 615 (30 370 to 34 739)	43 891 (40 117 to 48 084)	1 015.8 (957.0 to 1 074.5)	763.3 (697.6 to 834.4)
Pedestrian road injuries	337 (239 to 456)	1 084 (767 to 1 467)	16.2 (11.6 to 22.0)	22.1 (15.7 to 30.1)	13 229 (12 068 to 14 483)	15 711 (14 212 to 17 312)	417.3 (385.5 to 446.5)	280.3 (254.0 to 307.8)
Cyclist road injuries	132 (94 to 174)	385 (271 to 518)	5.8 (4.1 to 7.5)	7.5 (5.3 to 10.0)	862 (766 to 959)	1 608 (1 402 to 1 824)	27.7 (24.9 to 30.7)	28.8 (25.2 to 32.8)
Motorcyclist road injuries	269 (188 to 360)	761 (520 to 1 038)	12.4 (8.7 to 16.8)	15.0 (10.3 to 20.4)	5 737 (5 141 to 6 381)	8 475 (7 342 to 9 742)	175.7 (158.1 to 193.7)	141.4 (123.0 to 161.5)
Motor vehicle road injuries	383 (276 to 502)	1 709 (1 247 to 2 260)	18.7 (13.6 to 24.5)	34.5 (25.2 to 45.5)	11 929 (10 718 to 13 344)	17 067 (15 158 to 19 007)	367.8 (332.7 to 412.2)	294.2 (262.4 to 326.7)
Other road injuries	141 (101 to 192)	332 (239 to 458)	6.6 (4.8 to 8.9)	6.7 (4.8 to 9.2)	858 (726 to 983)	1 030 (888 to 1 208)	27.4 (23.5 to 31.1)	18.6 (16.0 to 21.9)
Other transport injuries	124 (88 to 166)	108 (78 to 145)	5.0 (3.6 to 6.6)	2.2 (1.6 to 2.9)	1 449 (1 311 to 1 612)	1 915 (1 693 to 2 171)	43.6 (39.9 to 47.6)	33.3 (29.5 to 37.5)
**Unintentional injuries**	**8 287 (6 139 to 10 828)**	**14 022 (10 200 to 18 660)**	**366.4 (269.1 to 479.0)**	**286.0 (207.7 to 381.3)**	**72 953 (67 262 to 79 197)**	**62 599 (57 714 to 68 416)**	**2 262.4 (2 110.5 to 2 424.5)**	**1 127.0 (1 034.7 to 1 237.0)**
Falls	4 045 (2 950 to 5 275)	8 942 (6 491 to 11 781)	193.9 (140.3 to 251.2)	186.6 (135.5 to 244.2)	10 534 (9 292 to 11 876)	16 711 (14 241 to 19 587)	453.1 (397.6 to 515.2)	341.6 (288.9 to 401.9)
Drowning	50 (34 to 68)	45 (31 to 61)	2.4 (1.7 to 3.2)	0.9 (0.6 to 1.3)	15 626 (14 219 to 17 179)	10 691 (9 646 to 11 793)	422.1 (390.9 to 455.9)	176.0 (159.3 to 193.3)
Fire, heat, and hot substances	847 (540 to 1 299)	914 (552 to 1 486)	31.1 (19.6 to 47.4)	17.1 (10.3 to 27.0)	5 033 (4 351 to 5 858)	2 742 (2 340 to 3 314)	140.0 (123.0 to 161.9)	49.6 (42.3 to 60.1)
Poisonings	183 (114 to 270)	343 (216 to 521)	5.9 (3.7 to 8.6)	6.0 (3.8 to 9.0)	4 725 (4 288 to 5 246)	3 025 (2 719 to 3 389)	125.6 (116.2 to 136.5)	50.5 (45.6 to 56.3)
Poisoning by carbon monoxide	21 (13 to 33)	18 (11 to 28)	0.7 (0.4 to 1.1)	0.3 (0.2 to 0.5)	710 (621 to 820)	444 (386 to 507)	18.5 (16.5 to 20.9)	7.4 (6.5 to 8.4)
Poisoning by other means	162 (100 to 241)	325 (200 to 498)	5.2 (3.2 to 7.7)	5.7 (3.5 to 8.6)	4 015 (3 637 to 4 466)	2 581 (2 312 to 2 891)	107.1 (98.8 to 117.0)	43.1 (38.8 to 48.0)
Exposure to mechanical forces	1 521 (1 059 to 2 165)	1 844 (1 260 to 2 666)	71.4 (49.4 to 101.4)	37.0 (25.2 to 53.6)	16 597 (15 112 to 17 913)	8 196 (7 351 to 9 241)	548.0 (493.5 to 593.2)	147.7 (132.2 to 167.8)
Unintentional firearm injuries	212 (142 to 319)	140 (91 to 221)	10.1 (6.7 to 15.5)	2.9 (1.9 to 4.5)	8 403 (7 550 to 9 208)	2 494 (2 072 to 2 857)	264.0 (236.2 to 289.3)	43.4 (36.1 to 49.4)
Other exposure to mechanical forces	1 309 (909 to 1 860)	1 703 (1 161 to 2 466)	61.3 (42.4 to 87.9)	34.1 (23.2 to 49.5)	8 193 (6 784 to 9 210)	5 702 (4 989 to 6 651)	283.9 (229.6 to 321.4)	104.3 (91.2 to 121.9)
Adverse effects of medical treatment	21 (13 to 31)	42 (26 to 64)	0.8 (0.5 to 1.2)	0.8 (0.5 to 1.2)	3 224 (2 878 to 3 791)	2 833 (2 459 to 3 611)	89.8 (70.4 to 101.2)	50.7 (44.8 to 63.7)
Animal contact	207 (138 to 292)	270 (182 to 386)	8.1 (5.4 to 11.3)	5.2 (3.5 to 7.4)	1 652 (1 415 to 1 942)	869 (743 to 1 024)	42.4 (37.3 to 48.3)	15.3 (13.2 to 18.0)
Venomous animal contact	110 (70 to 163)	122 (79 to 176)	3.8 (2.5 to 5.5)	2.2 (1.4 to 3.2)	1 169 (969 to 1 409)	491 (405 to 580)	28.1 (23.9 to 32.8)	8.4 (6.9 to 9.9)
Non-venomous animal contact	96 (59 to 150)	147 (89 to 233)	4.3 (2.7 to 6.6)	3.0 (1.8 to 4.7)	483 (400 to 577)	378 (304 to 486)	14.3 (11.9 to 17.2)	6.9 (5.5 to 8.9)
Foreign body	297 (196 to 442)	410 (242 to 677)	13.0 (8.5 to 19.3)	8.6 (5.1 to 14.2)	9 529 (8 498 to 10 706)	8 651 (7 597 to 9 914)	246.5 (225.8 to 270.0)	141.1 (125.5 to 159.4)
Pulmonary aspiration and foreign body in airway	9 (6 to 13)	5 (3 to 7)	0.3 (0.2 to 0.4)	0.1 (0.1 to 0.1)	8 608 (7 639 to 9 714)	7 949 (6 947 to 9 189)	218.3 (199.3 to 239.7)	127.7 (113.3 to 145.4)
Foreign body in eyes	130 (62 to 244)	305 (158 to 552)	6.8 (3.6 to 12.1)	6.6 (3.5 to 12.0)	130 (62 to 244)	305 (158 to 552)	6.8 (3.6 to 12.1)	6.6 (3.5 to 12.0)
Foreign body in other body part	158 (112 to 212)	101 (72 to 132)	5.9 (4.3 to 7.8)	1.9 (1.4 to 2.5)	791 (574 to 930)	397 (340 to 520)	21.4 (16.7 to 24.7)	6.8 (5.8 to 8.7)
Environmental heat and cold exposure	172 (116 to 239)	174 (116 to 246)	7.5 (5.2 to 10.5)	3.5 (2.4 to 5.1)	2 183 (2 026 to 2 361)	723 (638 to 814)	80.5 (76.3 to 85.6)	13.8 (12.1 to 15.5)
Exposure to forces of nature	679 (457 to 1 000)	647 (460 to 901)	20.7 (14.6 to 29.4)	12.5 (8.9 to 17.3)	783 (559 to 1 104)	5 753 (5 293 to 6 301)	23.7 (17.5 to 32.4)	98.9 (90.2 to 108.0)
Other unintentional injuries	265 (193 to 350)	391 (280 to 528)	11.7 (8.5 to 15.4)	7.8 (5.6 to 10.6)	3 068 (2 798 to 3 348)	2 406 (2 151 to 2 721)	90.8 (83.4 to 98.5)	41.7 (37.8 to 47.0)

Death, YLL, incidence, prevalence, YLD, and DALY counts and rates per 100,000 for all injuries in Chihuahua in 1990 and 2017

Cause	Deaths: All-age counts (95% UI) 1990	Deaths: All-age counts (95% UI) 2017	Deaths: Age-standardised rates per 100,000 (95% UI) 1990	Deaths: Age-standardised rates per 100,000 (95% UI) 2017
**Transport and unintentional injuries**	**2 064 (1 980 to 2 165)**	**1 502 (1 407 to 1 605)**	**98.4 (95.4 to 101.9)**	**44.9 (42.1 to 47.9)**
**Transport injuries**	**819 (776 to 868)**	**694 (642 to 751)**	**35.4 (33.8 to 37.2)**	**19.5 (18.1 to 21.1)**
Road injuries	798 (755 to 846)	661 (612 to 715)	34.4 (32.8 to 36.1)	18.6 (17.3 to 20.1)
Pedestrian road injuries	321 (295 to 351)	274 (251 to 298)	15.5 (14.5 to 16.5)	8.1 (7.4 to 8.7)
Cyclist road injuries	21 (19 to 23)	29 (26 to 33)	0.9 (0.8 to 1.0)	0.8 (0.7 to 0.9)
Motorcyclist road injuries	132 (119 to 148)	80 (71 to 90)	5.1 (4.7 to 5.7)	2.1 (1.9 to 2.4)
Motor vehicle road injuries	309 (283 to 336)	264 (241 to 290)	12.3 (11.4 to 13.2)	7.2 (6.6 to 7.9)
Other road injuries	15 (13 to 17)	14 (12 to 16)	0.7 (0.6 to 0.7)	0.4 (0.3 to 0.5)
Other transport injuries	21 (19 to 24)	33 (29 to 37)	1.0 (0.9 to 1.1)	0.9 (0.8 to 1.0)
**Unintentional injuries**	**1 245 (1 188 to 1 313)**	**807 (757 to 860)**	**63.0 (60.9 to 65.3)**	**25.3 (23.8 to 26.9)**
Falls	207 (197 to 217)	225 (208 to 241)	15.6 (14.9 to 16.4)	7.7 (7.2 to 8.3)
Drowning	204 (187 to 223)	97 (89 to 106)	8.2 (7.7 to 8.9)	2.8 (2.5 to 3.0)
Fire, heat, and hot substances	96 (88 to 106)	63 (58 to 69)	5.4 (5.1 to 5.7)	2.1 (1.9 to 2.2)
Poisonings	81 (74 to 89)	51 (47 to 55)	3.5 (3.2 to 3.8)	1.5 (1.4 to 1.6)
Poisoning by carbon monoxide	50 (45 to 55)	33 (31 to 36)	2.1 (2.0 to 2.3)	1.0 (0.9 to 1.1)
Poisoning by other means	31 (28 to 35)	18 (16 to 19)	1.3 (1.2 to 1.4)	0.5 (0.5 to 0.6)
Exposure to mechanical forces	256 (241 to 271)	93 (85 to 101)	11.0 (10.4 to 11.6)	2.7 (2.5 to 2.9)
Unintentional firearm injuries	164 (152 to 192)	40 (35 to 45)	6.6 (6.2 to 7.8)	1.1 (1.0 to 1.2)
Other exposure to mechanical forces	92 (81 to 102)	53 (48 to 59)	4.4 (4.1 to 4.8)	1.5 (1.4 to 1.7)
Adverse effects of medical treatment	47 (38 to 52)	59 (53 to 70)	3.0 (2.5 to 3.3)	2.0 (1.8 to 2.3)
Animal contact	14 (12 to 16)	8 (7 to 8)	0.6 (0.6 to 0.7)	0.2 (0.2 to 0.3)
Venomous animal contact	9 (7 to 11)	4 (3 to 4)	0.4 (0.3 to 0.4)	0.1 (0.1 to 0.1)
Non venomous animal contact	5 (4 to 6)	4 (3 to 5)	0.3 (0.2 to 0.3)	0.1 (0.1 to 0.1)
Foreign body	174 (155 to 195)	169 (150 to 192)	7.4 (6.8 to 8.1)	5.2 (4.6 to 5.9)
Pulmonary aspiration and foreign body in airway	165 (146 to 187)	164 (146 to 187)	7.1 (6.4 to 7.7)	5.1 (4.5 to 5.8)
Foreign body in eyes				
Foreign body in other body part	9 (6 to 10)	5 (4 to 6)	0.3 (0.2 to 0.4)	0.1 (0.1 to 0.2)
Environmental heat and cold exposure	36 (34 to 39)	21 (18 to 23)	2.2 (2.1 to 2.3)	0.6 (0.5 to 0.7)
Exposure to forces of nature	96 (87 to 106)	1 (1 to 1)	4.5 (4.1 to 4.9)	0.0 (0.0 to 0.0)
Other unintentional injuries	35 (32 to 38)	22 (20 to 24)	1.5 (1.4 to 1.6)	0.6 (0.5 to 0.7)

Cause	Years of life lost: All-age counts (95% UI) 1990	Years of life lost: All-age counts (95% UI) 2017	Years of life lost: Age-standardised rates per 100,000 (95% UI) 1990	Years of life lost: Age-standardised rates per 100,000 (95% UI) 2017
**Transport and unintentional injuries**	**113 798 (107 192 to 121 935)**	**66 603 (61 761 to 72 026)**	**4 367.8 (4 156.5 to 4 622.6)**	**1 875.6 (1 739.2 to 2 027.0)**
**Transport injuries**	**46 549 (43 510 to 50 120)**	**32 949 (30 266 to 35 776)**	**1 755.8 (1 656.9 to 1 868.8)**	**895.1 (822.6 to 972.1)**
Road injuries	45 385 (42 409 to 48 887)	31 387 (28 907 to 34 137)	1 710.1 (1 613.6 to 1 821.1)	853.0 (786.3 to 925.6)
Pedestrian road injuries	17 426 (15 602 to 19 609)	11 723 (10 660 to 12 848)	685.7 (626.6 to 754.9)	327.2 (297.7 to 358.4)
Cyclist road injuries	1 147 (1 030 to 1 295)	1 315 (1 169 to 1 471)	44.4 (40.1 to 49.4)	35.8 (31.8 to 40.0)
Motorcyclist road injuries	7 852 (6 971 to 8 959)	4 328 (3 805 to 4 901)	286.0 (257.7 to 319.5)	113.9 (100.3 to 129.1)
Motor vehicle road injuries	18 113 (16 502 to 19 863)	13 353 (12 069 to 14 703)	662.3 (607.7 to 717.4)	358.0 (323.9 to 392.8)
Other road injuries	846 (714 to 998)	668 (574 to 778)	31.8 (27.4 to 36.8)	18.1 (15.6 to 20.9)
Other transport injuries	1 164 (1 090 to 1 303)	1 562 (1 357 to 1 785)	45.6 (41.0 to 50.8)	42.1 (36.7 to 47.9)
**Unintentional injuries**	**67 250 (62 714 to 72 907)**	**33 654 (30 946 to 36 929)**	**2 612.0 (2 468.9 to 2 792.2)**	**980.6 (898.6 to 1 080.8)**
Falls	6 813 (6 412 to 7 276)	5 666 (5 182 to 6 120)	355.5 (338.2 to 373.4)	170.4 (156.5 to 183.1)
Drowning	12 523 (11 288 to 14 064)	4 958 (4 481 to 5 497)	448.5 (407.3 to 497.6)	138.8 (125.0 to 153.9)
Fire, heat, and hot substances	4 958 (4 330 to 5 684)	2 214 (2 001 to 2 442)	197.8 (177.6 to 222.0)	64.9 (58.6 to 71.6)
Poisonings	4 960 (4 407 to 5 575)	2 482 (2 258 to 2 727)	180.8 (163.6 to 200.9)	69.8 (63.3 to 76.6)
Poisoning by carbon monoxide	2 989 (2 656 to 3 371)	1 603 (1 451 to 1 773)	109.6 (98.6 to 121.7)	44.8 (40.6 to 49.8)
Poisoning by other means	1 971 (1 700 to 2 264)	879 (780 to 981)	71.2 (62.4 to 80.8)	25.0 (22.1 to 28.1)
Exposure to mechanical forces	14 335 (13 414 to 15 288)	4 076 (3 726 to 4 459)	544.0 (512.4 to 576.6)	111.5 (102.1 to 121.8)
Unintentional firearm injuries	9 488 (8 732 to 11 022)	1 902 (1 679 to 2 138)	349.8 (323.4 to 408.6)	51.1 (44.1 to 57.3)
Other exposure to mechanical forces	4 847 (4 206 to 5 485)	2 174 (1 941 to 2 488)	194.2 (180.1 to 217.4)	60.4 (54.1 to 68.8)
Adverse effects of medical treatment	2 065 (1 663 to 2 372)	1 912 (1 689 to 2 303)	89.9 (79.3 to 100.9)	57.4 (50.8 to 69.3)
Animal contact	880 (713 to 1 082)	323 (286 to 367)	31.7 (26.7 to 38.1)	9.5 (8.4 to 10.9)
Venomous animal contact	603 (464 to 782)	166 (132 to 205)	21.0 (16.6 to 26.7)	5.0 (4.0 to 6.2)
Non venomous animal contact	277 (228 to 325)	157 (135 to 188)	10.7 (8.9 to 12.4)	4.5 (3.9 to 5.4)
Foreign body	11 446 (9 876 to 13 299)	10 199 (8 616 to 12 110)	409.3 (353.5 to 461.3)	307.0 (258.8 to 370.0)
Pulmonary aspiration and foreign body in airway	10 875 (9 324 to 12 676)	9 870 (8 352 to 11 830)	382.6 (334.7 to 439.2)	299.6 (251.4 to 362.3)
Foreign body in eyes				
Foreign body in other body part	571 (383 to 684)	269 (208 to 321)	20.7 (14.0 to 24.5)	7.4 (5.7 to 8.8)
Environmental heat and cold exposure	1 607 (1 465 to 1 760)	769 (684 to 855)	73.9 (68.4 to 79.6)	21.4 (19.1 to 23.8)
Exposure to forces of nature	5 651 (5 048 to 6 340)	29 (27 to 32)	212.0 (191.7 to 235.5)	0.8 (0.7 to 0.9)
Other unintentional injuries	2 016 (1 840 to 2 222)	1 084 (969 to 1 208)	74.6 (68.8 to 81.5)	29.0 (25.9 to 32.4)

Cause	Incidence: All-age counts (95% UI) 1990	Incidence: All-age counts (95% UI) 2017	Incidence: Age-standardised rates per 100,000 (95% UI) 1990	Incidence: Age-standardised rates per 100,000 (95% UI) 2017
**Transport and unintentional injuries**	**120 412 (111 382 to 130 343)**	**169 673 (155 671 to 184 735)**	**5 415.5 (5 005.2 to 5 844.8)**	**4 973.3 (4 574.7 to 5 417.9)**
**Transport injuries**	**8 946 (7 729 to 10 403)**	**17 753 (15 410 to 20 365)**	**383.9 (336.6 to 436.1)**	**488.6 (424.0 to 558.8)**
Road injuries	8 548 (7 322 to 9 982)	17 395 (14 995 to 19 999)	366.9 (319.8 to 419.4)	478.1 (412.7 to 548.2)
Pedestrian road injuries	1 298 (1 044 to 1 611)	2 087 (1 686 to 2 532)	58.6 (48.1 to 70.9)	58.4 (47.3 to 70.8)
Cyclist road injuries	1 133 (852 to 1 496)	1 764 (1 327 to 2 284)	46.5 (35.3 to 59.5)	49.7 (37.4 to 63.7)
Motorcyclist road injuries	1 282 (1 007 to 1 616)	1 519 (1 152 to 1 938)	52.0 (42.0 to 64.0)	41.3 (31.2 to 52.6)
Motor vehicle road injuries	3 352 (2 765 to 3 995)	8 863 (7 436 to 10 498)	145.2 (121.5 to 170.8)	240.5 (202.4 to 283.9)
Other road injuries	1 484 (1 138 to 1 864)	3 162 (2 512 to 3 872)	64.6 (51.6 to 78.7)	88.2 (70.4 to 107.4)
Other transport injuries	397 (302 to 524)	358 (269 to 461)	16.9 (13.5 to 21.1)	10.4 (7.8 to 13.4)
**Unintentional injuries**	**111 466 (102 644 to 121 242)**	**151 920 (138 321 to 167 112)**	**5 031.7 (4 627.8 to 5 461.6)**	**4 484.7 (4 092.9 to 4 914.4)**
Falls	40 419 (35 771 to 45 782)	78 724 (70 192 to 88 822)	2 096.5 (1 879.3 to 2 351.9)	2 410.4 (2 147.6 to 2 714.5)
Drowning	151 (133 to 174)	82 (66 to 104)	6.2 (5.4 to 7.1)	2.3 (1.9 to 2.9)
Fire, heat, and hot substances	3 986 (3 234 to 4 883)	3 271 (2 724 to 3 888)	144.1 (119.8 to 172.7)	94.9 (79.0 to 112.6)
Poisonings	1 569 (1 219 to 1 956)	1 238 (936 to 1 650)	52.8 (41.6 to 65.2)	36.9 (27.8 to 49.4)
Poisoning by carbon monoxide	330 (239 to 448)	209 (142 to 300)	12.2 (9.1 to 15.9)	6.1 (4.2 to 8.8)
Poisoning by other means	1 239 (937 to 1 564)	1 028 (752 to 1 402)	40.6 (31.1 to 50.8)	30.7 (22.5 to 41.9)
Exposure to mechanical forces	30 048 (25 651 to 35 482)	24 003 (20 692 to 28 011)	1 198.1 (1 036.9 to 1 392.5)	659.4 (571.7 to 762.0)
Unintentional firearm injuries	3 201 (2 365 to 4 266)	1 331 (996 to 1 736)	124.0 (94.1 to 160.6)	36.4 (27.3 to 47.0)
Other exposure to mechanical forces	26 847 (22 996 to 31 869)	22 672 (19 513 to 26 357)	1 074.1 (929.8 to 1 256.1)	623.0 (539.8 to 723.2)
Adverse effects of medical treatment	2 211 (1 885 to 2 539)	4 552 (3 890 to 5 212)	109.8 (95.6 to 125.1)	130.4 (111.6 to 148.5)
Animal contact	5 277 (4 300 to 6 508)	5 852 (4 995 to 6 874)	221.1 (184.4 to 263.3)	167.8 (143.4 to 196.7)
Venomous animal contact	1 830 (1 358 to 2 443)	2 095 (1 641 to 2 635)	75.9 (58.0 to 98.6)	59.6 (46.8 to 74.8)
Non venomous animal contact	3 447 (2 711 to 4 352)	3 757 (3 105 to 4 570)	145.3 (117.2 to 178.2)	108.2 (89.2 to 131.3)
Foreign body	9 116 (4 758 to 14 628)	14 360 (5 919 to 24 577)	451.7 (209.1 to 751.3)	415.6 (172.4 to 709.0)
Pulmonary aspiration and foreign body in airway	344 (246 to 472)	178 (128 to 248)	12.1 (9.1 to 16.3)	5.4 (3.8 to 7.5)
Foreign body in eyes	8 082 (3 711 to 13 629)	13 500 (5 021 to 23 771)	412.7 (167.9 to 710.7)	390.1 (145.6 to 682.3)
Foreign body in other body part	691 (530 to 916)	682 (544 to 854)	26.9 (21.0 to 34.5)	20.2 (16.0 to 25.1)
Environmental heat and cold exposure	1 510 (1 176 to 1 912)	723 (550 to 918)	70.5 (57.1 to 84.4)	21.8 (16.8 to 28.0)
Exposure to forces of nature	7 317 (6 097 to 9 021)	39 (33 to 47)	274.2 (233.3 to 326.2)	1.1 (0.9 to 1.3)
Other unintentional injuries	9 869 (8 359 to 11 759)	19 079 (16 021 to 22 842)	406.6 (351.7 to 470.1)	544.1 (456.8 to 634.9)

Cause	Prevalence: All-age counts (95% UI) 1990	Prevalence: All-age counts (95% UI) 2017	Prevalence: Age-standardised rates per 100,000 (95% UI) 1990	Prevalence: Age-standardised rates per 100,000 (95% UI) 2017
**Transport and unintentional injuries**	**226 373 (211 983 to 240 331)**	**355 710 (334 755 to 381 805)**	**11 998.8 (11 308.5 to 12 811.7)**	**10 363.5 (9 764.6 to 11 130.5)**
**Transport injuries**	**21 879 (20 346 to 23 321)**	**49 718 (46 152 to 53 647)**	**1 183.8 (1 101.9 to 1 263.1)**	**1 417.6 (1 315.9 to 1 530.6)**
Road injuries	20 041 (18 581 to 21 457)	47 893 (44 317 to 51 805)	1 090.6 (1 011.7 to 1 166.8)	1 364.7 (1 263.1 to 1 474.3)
Pedestrian road injuries	4 896 (4 156 to 5 770)	9 543 (8 221 to 11 196)	268.3 (231.6 to 314.9)	275.6 (238.9 to 320.2)
Cyclist road injuries	1 928 (1 613 to 2 278)	3 819 (3 248 to 4 431)	100.4 (85.1 to 116.5)	109.3 (93.6 to 126.4)
Motorcyclist road injuries	4 574 (3 943 to 5 298)	6 996 (6 078 to 8 202)	243.8 (209.8 to 282.1)	196.2 (169.4 to 229.2)
Motor vehicle road injuries	5 896 (5 166 to 6 876)	20 040 (17 460 to 23 379)	330.4 (286.5 to 384.5)	569.4 (495.8 to 663.0)
Other road injuries	2 747 (2 287 to 3 318)	7 495 (6 235 to 9 083)	147.6 (123.5 to 177.4)	214.1 (178.5 to 258.8)
Other transport injuries	1 838 (1 521 to 2 181)	1 825 (1 558 to 2 104)	93.3 (78.7 to 108.2)	52.9 (45.6 to 60.5)
**Unintentional injuries**	**204 494 (190 246 to 218 103)**	**305 992 (285 611 to 331 772)**	**10 815.0 (10 120.0 to 11 606.3)**	**8 945.9 (8 310.2 to 9 715.4)**
Falls	71 435 (63 132 to 81 847)	154 668 (137 123 to 176 117)	4 126.3 (3 689.3 to 4 711.4)	4 656.0 (4 147.6 to 5 317.4)
Drowning	703 (618 to 811)	499 (438 to 583)	38.7 (34.2 to 44.5)	14.7 (12.9 to 17.0)
Fire, heat, and hot substances	39 904 (33 703 to 48 217)	38 929 (32 327 to 48 548)	1 875.1 (1 601.2 to 2 220.8)	1 092.0 (912.8 to 1 355.7)
Poisonings	1 415 (1 115 to 1 703)	1 515 (1 122 to 1 890)	59.3 (47.1 to 71.0)	41.8 (31.0 to 52.1)
Poisoning by carbon monoxide	228 (178 to 294)	163 (126 to 211)	9.9 (7.8 to 12.6)	4.5 (3.5 to 5.9)
Poisoning by other means	1 187 (890 to 1 454)	1 352 (960 to 1 718)	49.4 (37.4 to 60.3)	37.2 (26.4 to 47.3)
Exposure to mechanical forces	58 673 (51 333 to 66 645)	58 641 (51 452 to 67 756)	3 128.2 (2 753.7 to 3 550.4)	1 658.5 (1 457.0 to 1 905.0)
Unintentional firearm injuries	10 648 (8 375 to 13 076)	6 430 (5 210 to 7 741)	578.9 (461.0 to 703.3)	182.6 (148.6 to 219.5)
Other exposure to mechanical forces	48 026 (40 553 to 56 415)	52 211 (44 915 to 61 622)	2 549.3 (2 164.8 to 3 011.5)	1 475.9 (1 270.9 to 1 736.7)
Adverse effects of medical treatment	169 (130 to 209)	348 (269 to 430)	8.4 (6.5 to 10.4)	10.0 (7.7 to 12.2)
Animal contact	5 780 (4 910 to 6 651)	7 554 (6 571 to 8 631)	293.1 (255.1 to 336.0)	215.9 (188.4 to 246.4)
Venomous animal contact	1 218 (1 000 to 1 440)	1 624 (1 362 to 1 886)	58.1 (48.4 to 67.7)	45.8 (38.6 to 53.0)
Non venomous animal contact	4 512 (3 728 to 5 393)	5 930 (4 958 to 7 005)	235.0 (198.5 to 277.2)	170.1 (142.9 to 200.1)
Foreign body	4 102 (3 167 to 5 430)	5 689 (4 234 to 7 886)	210.8 (164.1 to 277.3)	167.1 (124.7 to 231.8)
Pulmonary aspiration and foreign body in airway	254 (180 to 343)	165 (131 to 208)	11.0 (7.9 to 14.5)	4.7 (3.7 to 5.9)
Foreign body in eyes	1 603 (830 to 2 824)	2 912 (1 574 to 4 941)	93.8 (53.1 to 153.4)	83.4 (49.3 to 144.0)
Foreign body in other body part	2 246 (1 812 to 2 844)	2 612 (2 142 to 3 270)	106.0 (87.2 to 131.2)	74.0 (60.9 to 92.5)
Environmental heat and cold exposure	5 217 (4 315 to 6 261)	2 714 (2 314 to 3 244)	274.4 (234.1 to 321.1)	80.2 (68.9 to 95.6)
Exposure to forces of nature	1 726 (1 340 to 2 179)	2 139 (1 503 to 2 951)	68.3 (53.6 to 85.8)	58.9 (41.3 to 81.1)
Other unintentional injuries	14 421 (12 306 to 16 901)	33 311 (28 533 to 39 088)	782.7 (692.6 to 842.2)	951.4 (818.2 to 1 109.5)

Cause	Years lived with disability: All-age counts (95% UI) 1990	Years lived with disability: All-age counts (95% UI) 2017	Years lived with disability: Age-standardised rates per 100,000 (95% UI) 1990	Years lived with disability: Age-standardised rates per 100,000 (95% UI) 2017
**Transport and unintentional injuries**	**9 441 (6 825 to 12 483)**	**14 812 (10 689 to 20 110)**	**495.8 (357.1 to 658.2)**	**432.0 (309.1 to 585.9)**
**Transport injuries**	**1 484 (1 088 to 1 961)**	**2 968 (2 159 to 3 992)**	**77.0 (56.3 to 101.2)**	**84.1 (60.9 to 112.3)**
Road injuries	1 368 (1 002 to 1 812)	2 893 (2 090 to 3 860)	71.4 (52.3 to 94.4)	81.3 (58.9 to 108.8)
Pedestrian road injuries	349 (244 to 475)	602 (429 to 824)	18.3 (12.9 to 24.7)	17.2 (12.2 to 23.4)
Cyclist road injuries	133 (93 to 174)	227 (157 to 303)	6.7 (4.7 to 8.7)	6.4 (4.5 to 8.5)
Motorcyclist road injuries	314 (221 to 423)	426 (291 to 584)	16.1 (11.3 to 21.9)	11.8 (8.0 to 16.2)
Motor vehicle road injuries	417 (301 to 555)	1 258 (909 to 1 671)	22.4 (16.2 to 29.6)	35.2 (25.4 to 46.8)
Other road injuries	154 (110 to 208)	379 (267 to 526)	7.9 (5.8 to 10.7)	10.7 (7.5 to 14.8)
Other transport injuries	116 (82 to 155)	95 (68 to 128)	5.6 (4.0 to 7.4)	2.7 (2.0 to 3.7)
**Unintentional injuries**	**7 958 (5 746 to 10 670)**	**11 824 (8 392 to 16 099)**	**418.8 (302.1 to 562.8)**	**347.9 (247.1 to 472.3)**
Falls	4 197 (3 083 to 5 473)	7 778 (5 574 to 10 415)	234.8 (171.6 to 305.7)	233.4 (166.9 to 311.9)
Drowning	40 (28 to 55)	26 (18 to 37)	2.1 (1.5 to 2.9)	0.8 (0.5 to 1.1)
Fire, heat, and hot substances	852 (532 to 1 339)	747 (442 to 1 193)	38.8 (24.0 to 61.1)	20.9 (12.4 to 33.3)
Poisonings	165 (109 to 245)	176 (107 to 264)	6.7 (4.2 to 9.9)	4.8 (2.9 to 7.3)
Poisoning by carbon monoxide	24 (15 to 37)	16 (10 to 26)	1.0 (0.6 to 1.5)	0.4 (0.3 to 0.7)
Poisoning by other means	141 (87 to 211)	160 (95 to 243)	5.7 (3.5 to 8.4)	4.4 (2.6 to 6.6)
Exposure to mechanical forces	1 373 (948 to 1 953)	1 258 (847 to 1 840)	71.5 (49.5 to 102.0)	35.4 (23.9 to 51.8)
Unintentional firearm injuries	229 (150 to 345)	120 (77 to 189)	12.0 (7.8 to 18.3)	3.4 (2.2 to 5.3)
Other exposure to mechanical forces	1 144 (794 to 1 633)	1 138 (765 to 1 678)	59.5 (41.2 to 85.7)	32.1 (21.5 to 47.3)
Adverse effects of medical treatment	23 (14 to 34)	46 (29 to 69)	1.1 (0.7 to 1.7)	1.3 (0.8 to 2.0)
Animal contact	150 (100 to 212)	174 (117 to 249)	7.0 (4.7 to 9.9)	4.9 (3.3 to 7.0)
Venomous animal contact	80 (51 to 118)	91 (58 to 134)	3.4 (2.2 to 4.9)	2.5 (1.6 to 3.7)
Non venomous animal contact	69 (42 to 111)	83 (48 to 134)	3.6 (2.2 to 5.6)	2.4 (1.4 to 3.8)
Foreign body	254 (169 to 376)	321 (198 to 512)	12.8 (8.5 to 18.9)	9.4 (5.8 to 14.9)
Pulmonary aspiration and foreign body in airway	13 (9 to 19)	7 (5 to 10)	0.5 (0.4 to 0.7)	0.2 (0.1 to 0.3)
Foreign body in eyes	107 (54 to 198)	194 (100 to 357)	6.8 (3.4 to 11.2)	5.9 (3.1 to 10.7)
Foreign body in other body part	134 (94 to 178)	120 (85 to 163)	6.0 (4.3 to 7.9)	3.4 (2.4 to 4.6)
Environmental heat and cold exposure	205 (136 to 289)	103 (67 to 148)	10.7 (7.2 to 15.1)	3.1 (2.0 to 4.4)
Exposure to forces of nature	132 (80 to 190)	67 (48 to 93)	5.1 (3.6 to 7.3)	1.8 (1.3 to 2.6)
Other unintentional injuries	568 (407 to 764)	1 129 (791 to 1 572)	28.1 (20.2 to 38.0)	32.1 (22.6 to 44.8)

Cause	Disability adjusted life years: All-age counts (95% UI) 1990	Disability adjusted life years: All-age counts (95% UI) 2017	Disability adjusted life years: Age-standardised rates per 100,000 (95% UI) 1990	Disability adjusted life years: Age-standardised rates per 100,000 (95% UI) 2017
**Transport and unintentional injuries**	**123 240 (115 745 to 131 732)**	**81 415 (74 546 to 88 923)**	**4 863.5 (4 595.4 to 5 162.0)**	**2 307.6 (2 113.0 to 2 523.8)**
**Transport injuries**	**48 032 (44 891 to 51 513)**	**35 917 (33 161 to 39 007)**	**1 832.7 (1 727.6 to 1 946.6)**	**979.2 (903.5 to 1 062.5)**
Road injuries	46 752 (43 691 to 50 205)	34 280 (31 586 to 37 290)	1 781.5 (1 679.3 to 1 892.9)	934.4 (862.0 to 1 013.7)
Pedestrian road injuries	17 775 (15 975 to 19 904)	12 325 (11 241 to 13 423)	703.9 (643.5 to 773.3)	344.3 (314.9 to 375.6)
Cyclist road injuries	1 280 (1 153 to 1 427)	1 542 (1 383 to 1 722)	51.1 (46.1 to 56.5)	42.3 (37.9 to 47.1)
Motorcyclist road injuries	8 167 (7 291 to 9 287)	4 754 (4 210 to 5 355)	302.1 (273.9 to 336.0)	125.8 (111.4 to 141.4)
Motor vehicle road injuries	18 530 (16 930 to 20 254)	14 611 (13 291 to 16 029)	684.6 (631.3 to 740.6)	393.2 (358.1 to 431.1)
Other road injuries	1 000 (849 to 1 169)	1 047 (886 to 1 224)	39.8 (34.3 to 45.5)	28.8 (24.6 to 33.6)
Other transport injuries	1 280 (1 148 to 1 421)	1 657 (1 452 to 1 889)	51.2 (46.3 to 56.3)	44.8 (39.4 to 50.9)
**Unintentional injuries**	**75 207 (69 762 to 81 632)**	**45 478 (40 949 to 50 812)**	**3 030.8 (2 832.7 to 3 264.7)**	**1 328.5 (1 196.5 to 1 488.8)**
Falls	11 010 (9 768 to 12 390)	13 444 (11 209 to 16 089)	590.3 (524.2 to 664.4)	403.8 (336.1 to 483.2)
Drowning	12 563 (11 275 to 14 105)	4 984 (4 508 to 5 523)	450.6 (409.1 to 500.1)	139.6 (125.8 to 154.7)
Fire, heat, and hot substances	5 806 (5 078 to 6 768)	2 961 (2 576 to 3 495)	236.6 (210.5 to 270.4)	85.8 (74.6 to 100.7)
Poisonings	5 125 (4 578 to 5 751)	2 658 (2 418 to 2 916)	187.5 (169.7 to 207.7)	74.6 (67.8 to 82.0)
Poisoning by carbon monoxide	3 013 (2 675 to 3 391)	1 619 (1 467 to 1 791)	110.6 (99.3 to 122.8)	45.3 (41.0 to 50.2)
Poisoning by other means	2 112 (1 846 to 2 413)	1 039 (920 to 1 177)	76.9 (68.3 to 86.7)	29.4 (26.0 to 33.2)
Exposure to mechanical forces	15 708 (14 667 to 16 880)	5 334 (4 748 to 6 009)	615.5 (575.0 to 660.3)	146.9 (130.8 to 165.6)
Unintentional firearm injuries	9 717 (8 952 to 11 216)	2 022 (1 799 to 2 261)	361.7 (335.1 to 419.6)	54.5 (47.0 to 60.8)
Other exposure to mechanical forces	5 991 (4 359 to 6 804)	3 312 (2 832 to 3 905)	253.8 (189.8 to 289.0)	92.5 (79.1 to 108.7)
Adverse effects of medical treatment	2 088 (1 688 to 2 400)	1 958 (1 796 to 2 345)	91.1 (79.7 to 102.9)	58.8 (52.1 to 70.6)
Animal contact	1 030 (854 to 1 248)	497 (426 to 582)	38.7 (32.8 to 46.1)	14.4 (12.3 to 16.8)
Venomous animal contact	684 (541 to 870)	257 (209 to 310)	24.4 (19.8 to 30.3)	7.5 (6.1 to 9.1)
Non venomous animal contact	346 (284 to 409)	240 (198 to 297)	14.3 (11.7 to 16.9)	6.9 (5.7 to 8.5)
Foreign body	11 700 (10 107 to 13 500)	10 460 (8 915 to 12 450)	416.1 (365.4 to 474.5)	316.4 (267.5 to 379.6)
Pulmonary aspiration and foreign body in airway	10 889 (9 338 to 12 691)	9 877 (8 361 to 11 837)	383.2 (335.3 to 439.7)	299.8 (251.6 to 362.5)
Foreign body in eyes	107 (54 to 198)	194 (100 to 357)	6.8 (3.4 to 11.2)	5.9 (3.1 to 10.7)
Foreign body in other body part	705 (513 to 825)	389 (322 to 458)	26.6 (19.9 to 30.8)	10.8 (8.9 to 12.6)
Environmental heat and cold exposure	1 812 (1 647 to 1 985)	872 (784 to 971)	84.6 (77.8 to 92.0)	24.5 (22.0 to 27.3)
Exposure to forces of nature	5 783 (5 177 to 6 480)	96 (77 to 125)	217.2 (197.0 to 240.8)	2.6 (2.1 to 3.4)
Other unintentional injuries	2 584 (2 339 to 2 857)	2 213 (1 859 to 2 645)	102.8 (92.8 to 113.7)	61.1 (51.2 to 73.3)

Death, YLL, incidence, prevalence, YLD, and DALY counts and rates per 100,000 for all injuries in Coahuila in 1990 and 2017

Cause	Deaths: All-age counts (95% UI) 1990	Deaths: All-age counts (95% UI) 2017	Deaths: Age-standardised rates per 100,000 (95% UI) 1990	Deaths: Age-standardised rates per 100,000 (95% UI) 2017
**Transport and unintentional injuries**	**1 039 (995 to 1 086)**	**981 (920 to 1 046)**	**66.0 (63.9 to 68.1)**	**33.6 (31.6 to 35.8)**
**Transport injuries**	**458 (435 to 484)**	**469 (434 to 506)**	**26.1 (25.0 to 27.2)**	**15.1 (14.0 to 16.3)**
Road injuries	445 (422 to 470)	448 (414 to 484)	25.2 (24.1 to 26.4)	14.4 (13.4 to 15.6)
Pedestrian road injuries	186 (173 to 200)	176 (162 to 192)	11.7 (11.0 to 12.3)	5.9 (5.4 to 6.4)
Cyclist road injuries	12 (11 to 13)	20 (18 to 23)	0.7 (0.6 to 0.7)	0.7 (0.6 to 0.7)
Motorcyclist road injuries	72 (65 to 79)	63 (56 to 71)	3.6 (3.3 to 4.0)	1.9 (1.7 to 2.2)
Motor vehicle road injuries	166 (154 to 179)	180 (163 to 198)	8.7 (8.2 to 9.4)	5.7 (5.1 to 6.2)
Other road injuries	9 (7 to 10)	9 (8 to 10)	0.5 (0.4 to 0.6)	0.3 (0.2 to 0.3)
Other transport injuries	14 (12 to 15)	22 (19 to 25)	0.8 (0.8 to 0.9)	0.7 (0.6 to 0.8)
**Unintentional injuries**	**581 (552 to 611)**	**512 (479 to 548)**	**39.9 (38.5 to 41.3)**	**18.5 (17.3 to 19.7)**
Falls	123 (117 to 130)	154 (143 to 166)	12.6 (12.0 to 13.3)	6.2 (5.7 to 6.7)
Drowning	121 (111 to 132)	69 (63 to 76)	6.2 (5.8 to 6.6)	2.2 (2.0 to 2.5)
Fire, heat, and hot substances	48 (45 to 52)	36 (33 to 39)	3.6 (3.4 to 3.8)	1.3 (1.3 to 1.4)
Poisonings	31 (29 to 34)	22 (20 to 23)	1.7 (1.6 to 1.8)	0.7 (0.7 to 0.8)
Poisoning by carbon monoxide	15 (14 to 16)	12 (11 to 13)	0.8 (0.8 to 0.9)	0.4 (0.4 to 0.4)
Poisoning by other means	16 (15 to 18)	10 (9 to 11)	0.9 (0.8 to 0.9)	0.3 (0.3 to 0.4)
Exposure to mechanical forces	87 (82 to 93)	54 (49 to 59)	5.1 (4.8 to 5.4)	1.8 (1.6 to 1.9)
Unintentional firearm injuries	43 (39 to 51)	19 (17 to 22)	2.3 (2.1 to 2.8)	0.6 (0.5 to 0.7)
Other exposure to mechanical forces	44 (35 to 48)	35 (31 to 38)	2.8 (2.3 to 3.0)	1.2 (1.0 to 1.3)
Adverse effects of medical treatment	33 (28 to 37)	45 (40 to 53)	2.9 (2.4 to 3.1)	1.7 (1.5 to 2.0)
Animal contact	6 (5 to 7)	3 (3 to 4)	0.4 (0.3 to 0.4)	0.1 (0.1 to 0.1)
Venomous animal contact	4 (3 to 4)	1 (1 to 2)	0.2 (0.2 to 0.2)	0.1 (0.0 to 0.1)
Non-venomous animal contact	3 (2 to 3)	2 (2 to 2)	0.2 (0.1 to 0.2)	0.1 (0.1 to 0.1)
Foreign body	92 (82 to 102)	102 (91 to 117)	5.0 (4.6 to 5.4)	3.5 (3.1 to 4.0)
Pulmonary aspiration and foreign body in airway	87 (78 to 97)	100 (88 to 114)	4.8 (4.4 to 5.2)	3.4 (3.1 to 3.9)
Foreign body in eyes				
Foreign body in other body part	5 (3 to 6)	3 (2 to 4)	0.2 (0.2 to 0.3)	0.1 (0.1 to 0.1)
Environmental heat and cold exposure	14 (13 to 15)	8 (7 to 9)	1.1 (1.0 to 1.2)	0.3 (0.2 to 0.3)
Exposure to forces of nature	1 (1 to 1)	0 (0 to 1)	0.1 (0.0 to 0.1)	0.0 (0.0 to 0.0)
Other unintentional injuries	25 (23 to 27)	18 (16 to 20)	1.4 (1.3 to 1.5)	0.6 (0.5 to 0.6)

Cause	Years of life lost: All-age counts (95% UI) 1990	Years of life lost: All-age counts (95% UI) 2017	Years of life lost: Age-standardised rates per 100,000 (95% UI) 1990	Years of life lost: Age-standardised rates per 100,000 (95% UI) 2017
**Transport and unintentional injuries**	**55 290 (51 910 to 58 994)**	**42 864 (39 718 to 46 427)**	**2 705.7 (2 572.9 to 2 848.2)**	**1 371.3 (1 269.4 to 1 483.5)**
**Transport injuries**	**25 296 (23 703 to 27 007)**	**22 397 (20 642 to 24 407)**	**1 224.5 (1 157.7 to 1 297.6)**	**698.0 (643.8 to 760.2)**
Road injuries	24 576 (23 085 to 26 235)	21 397 (19 724 to 23 255)	1 188.4 (1 122.4 to 1 258.4)	666.9 (614.7 to 724.3)
Pedestrian road injuries	9 828 (8 873 to 10 889)	7 566 (6 880 to 8 385)	489.0 (451.3 to 532.1)	240.3 (219.0 to 265.3)
Cyclist road injuries	657 (588 to 739)	896 (787 to 1 019)	32.5 (29.5 to 36.0)	28.1 (24.7 to 31.9)
Motorcyclist road injuries	4 205 (3 771 to 4 648)	3 429 (2 992 to 3 888)	196.2 (177.0 to 215.5)	104.5 (91.2 to 118.2)
Motor vehicle road injuries	9 421 (8 630 to 10 297)	9 103 (8 197 to 10 098)	448.2 (413.6 to 481.5)	281.4 (253.8 to 312.2)
Other road injuries	466 (398 to 544)	403 (350 to 470)	22.4 (19.5 to 25.6)	12.6 (11.0 to 14.6)
Other transport injuries	720 (647 to 809)	1 000 (876 to 1 141)	36.1 (32.8 to 39.9)	31.1 (27.3 to 35.5)
**Unintentional injuries**	**29 994 (27 729 to 32 441)**	**20 467 (18 681 to 22 578)**	**1 481.2 (1 393.6 to 1 576.1)**	**673.3 (613.8 to 743.9)**
Falls	3 776 (3 564 to 4 011)	3 668 (3 386 to 3 963)	258.3 (245.9 to 271.0)	127.9 (118.0 to 137.5)
Drowning	7 398 (6 664 to 8 264)	3 528 (3 141 to 3 995)	328.1 (299.5 to 362.4)	111.9 (99.4 to 124.8)
Fire, heat, and hot substances	2 400 (2 128 to 2 780)	1 206 (1 098 to 1 321)	121.3 (110.5 to 133.7)	40.3 (36.7 to 44.1)
Poisonings	1 881 (1 682 to 2 114)	1 029 (932 to 1 146)	85.7 (78.0 to 94.9)	32.9 (29.8 to 36.6)
Poisoning by carbon monoxide	874 (779 to 991)	546 (487 to 609)	40.3 (36.5 to 44.8)	17.4 (15.5 to 19.3)
Poisoning by other means	1 006 (880 to 1 143)	483 (430 to 541)	45.3 (40.4 to 50.5)	15.5 (13.8 to 17.4)
Exposure to mechanical forces	4 680 (4 366 to 5 081)	2 366 (2 127 to 2 620)	229.7 (216.1 to 244.5)	74.2 (66.8 to 81.9)
Unintentional firearm injuries	2 405 (2 174 to 2 863)	918 (794 to 1 057)	113.4 (103.2 to 135.4)	28.4 (24.6 to 32.6)
Other exposure to mechanical forces	2 275 (1 799 to 2 526)	1 448 (1 277 to 1 637)	116.3 (92.4 to 127.3)	45.8 (40.6 to 51.5)
Adverse effects of medical treatment	1 432 (1 169 to 1 621)	1 415 (1 261 to 1 766)	79.0 (64.5 to 87.4)	48.2 (42.9 to 58.7)
Animal contact	393 (330 to 465)	143 (126 to 164)	17.5 (15.0 to 20.3)	4.8 (4.2 to 5.4)
Venomous animal contact	253 (200 to 314)	66 (48 to 84)	10.7 (8.7 to 13.1)	2.2 (1.6 to 2.8)
Non-venomous animal contact	140 (116 to 168)	77 (65 to 97)	6.8 (5.8 to 7.7)	2.5 (2.1 to 3.2)
Foreign body	6 006 (5 215 to 6 895)	5 905 (4 977 to 7 091)	259.8 (230.0 to 293.4)	195.7 (164.6 to 236.1)
Pulmonary aspiration and foreign body in airway	5 697 (4 936 to 6 545)	5 757 (4 839 to 6 927)	246.0 (217.2 to 278.5)	191.1 (160.1 to 230.5)
Foreign body in eyes				
Foreign body in other body part	309 (222 to 369)	148 (124 to 212)	13.7 (9.8 to 16.2)	4.6 (3.9 to 6.6)
Environmental heat and cold exposure	578 (531 to 632)	277 (249 to 305)	33.7 (31.3 to 36.4)	8.9 (8.0 to 9.8)
Exposure to forces of nature	56 (51 to 62)	26 (23 to 28)	2.5 (2.3 to 2.8)	0.8 (0.7 to 0.9)
Other unintentional injuries	1 395 (1 265 to 1 524)	894 (798 to 1 019)	65.8 (60.2 to 71.4)	27.6 (24.5 to 31.4)

Cause	Incidence: All-age counts (95% UI) 1990	Incidence: All-age counts (95% UI) 2017	Incidence: Age-standardised rates per 100,000 (95% UI) 1990	Incidence: Age-standardised rates per 100,000 (95% UI) 2017
**Transport and unintentional injuries**	**72 623 (66 082 to 80 189)**	**102 134 (91 046 to 114 044)**	**4 138.9 (3 762.4 to 4 544.3)**	**3 422.1 (3 046.2 to 3 819.6)**
**Transport injuries**	**6 393 (5 428 to 7 510)**	**12 210 (10 320 to 14 313)**	**340.7 (292.6 to 390.3)**	**386.0 (327.9 to 450.4)**
Road injuries	6 036 (5 086 to 7 156)	11 830 (9 938 to 13 928)	322.4 (274.2 to 370.8)	373.4 (315.5 to 439.1)
Pedestrian road injuries	935 (749 to 1 165)	1 341 (1 037 to 1 760)	52.7 (42.5 to 64.3)	42.8 (33.4 to 54.3)
Cyclist road injuries	883 (653 to 1 176)	1 580 (1 162 to 2 019)	44.7 (34.4 to 57.3)	49.2 (37.6 to 64.4)
Motorcyclist road injuries	944 (731 to 1 199)	1 423 (1 052 to 1 887)	47.9 (37.9 to 59.6)	44.6 (32.9 to 59.2)
Motor vehicle road injuries	2 260 (1 806 to 2 743)	5 700 (4 549 to 6 900)	122.6 (98.8 to 146.1)	177.9 (142.4 to 214.4)
Other road injuries	1 015 (730 to 1 319)	1 886 (1 386 to 2 358)	54.6 (41.5 to 68.2)	58.8 (44.9 to 74.8)
Other transport injuries	357 (262 to 504)	380 (262 to 527)	18.3 (14.4 to 23.9)	12.6 (8.7 to 17.3)
**Unintentional injuries**	**65 231 (59 949 to 73 553)**	**89 923 (78 720 to 101 710)**	**3 798.2 (3 424.9 to 4 212.1)**	**3 036.1 (2 678.5 to 3 430.9)**
Falls	26 758 (23 392 to 30 620)	48 258 (42 478 to 55 306)	1 736.8 (1 546.4 to 1 967.1)	1 684.2 (1 483.3 to 1 925.0)
Drowning	108 (92 to 127)	71 (56 to 91)	5.5 (4.7 to 6.3)	2.3 (1.8 to 2.9)
Fire, heat, and hot substances	2 734 (2 171 to 3 440)	1 967 (1 565 to 2 496)	119.9 (97.4 to 147.6)	64.1 (51.0 to 81.5)
Poisonings	947 (710 to 1 253)	874 (625 to 1 188)	38.5 (29.3 to 50.1)	28.9 (20.8 to 39.6)
Poisoning by carbon monoxide	193 (125 to 288)	161 (93 to 254)	8.5 (5.9 to 12.2)	5.3 (3.1 to 8.3)
Poisoning by other means	755 (557 to 1 034)	713 (497 to 1 002)	30.0 (22.3 to 40.7)	23.6 (16.4 to 33.2)
Exposure to mechanical forces	17 822 (15 301 to 21 337)	15 640 (13 000 to 18 810)	884.7 (763.4 to 1 040.0)	493.6 (410.9 to 592.5)
Unintentional firearm injuries	1 524 (1 115 to 2 042)	734 (493 to 1 061)	73.3 (54.7 to 95.2)	23.3 (15.8 to 33.2)
Other exposure to mechanical forces	16 298 (13 802 to 19 538)	14 906 (12 405 to 17 967)	811.4 (698.6 to 954.6)	470.3 (392.4 to 566.8)
Adverse effects of medical treatment	1 517 (1 264 to 1 796)	2 659 (2 218 to 3 228)	94.1 (79.9 to 109.2)	87.6 (73.5 to 105.6)
Animal contact	4 045 (3 139 to 5 201)	3 886 (3 200 to 4 739)	205.3 (164.2 to 254.2)	127.0 (104.7 to 154.0)
Venomous animal contact	1 409 (986 to 1 970)	1 275 (970 to 1 621)	70.8 (51.0 to 95.6)	41.4 (31.6 to 52.6)
Non-venomous animal contact	2 636 (1 976 to 3 487)	2 612 (2 097 to 3 285)	134.5 (104.4 to 171.6)	85.6 (68.7 to 107.3)
Foreign body	7 679 (4 077 to 12 312)	12 959 (5 197 to 22 265)	469.5 (215.3 to 770.4)	428.7 (169.7 to 741.7)
Pulmonary aspiration and foreign body in airway	265 (187 to 359)	155 (110 to 214)	11.2 (8.2 to 14.8)	5.2 (3.7 to 7.1)
Foreign body in eyes	6 794 (3 225 to 11 528)	12 388 (4 648 to 21 695)	429.7 (172.5 to 784.4)	409.7 (149.5 to 722.1)
Foreign body in other body part	614 (413 to 843)	415 (289 to 562)	28.6 (20.0 to 40.9)	13.8 (9.5 to 18.5)
Environmental heat and cold exposure	1 057 (744 to 1 427)	654 (465 to 840)	58.1 (44.5 to 72.7)	22.3 (15.9 to 31.7)
Exposure to forces of nature	147 (120 to 184)	34 (29 to 41)	6.5 (5.5 to 7.9)	1.1 (0.9 to 1.3)
Other unintentional injuries	3 428 (2 769 to 4 251)	2 920 (2 292 to 3 764)	179.2 (148.7 to 215.8)	96.2 (75.8 to 123.3)

Cause	Prevalence: All-age counts (95% UI) 1990	Prevalence: All-age counts (95% UI) 2017	Prevalence: Age-standardised rates per 100,000 (95% UI) 1990	Prevalence: Age-standardised rates per 100,000 (95% UI) 2017
**Transport and unintentional injuries**	**142 532 (133 959 to 152 634)**	**211 800 (197 940 to 228 676)**	**9 481.2 (8 916.0 to 10 170.9)**	**7 105.7 (6 653.0 to 7 670.5)**
**Transport injuries**	**15 628 (14 573 to 16 625)**	**35 171 (32 630 to 37 880)**	**1 057.5 (987.0 to 1 126.8)**	**1 151.6 (1 067.8 to 1 241.3)**
Road injuries	13 962 (12 991 to 14 932)	33 144 (30 652 to 35 782)	954.8 (888.0 to 1 023.3)	1 084.6 (1 003.6 to 1 170.8)
Pedestrian road injuries	3 422 (2 930 to 4 036)	6 162 (5 287 to 7 228)	236.9 (205.2 to 278.6)	204.0 (175.5 to 239.1)
Cyclist road injuries	1 466 (1 229 to 1 727)	3 304 (2 807 to 3 875)	95.6 (80.5 to 110.7)	108.4 (92.3 to 126.8)
Motorcyclist road injuries	3 307 (2 857 to 3 808)	6 487 (5 585 to 7 600)	220.6 (190.9 to 254.5)	209.5 (180.4 to 245.0)
Motor vehicle road injuries	3 902 (3 402 to 4 538)	12 798 (11 119 to 14 997)	276.5 (240.9 to 321.2)	418.5 (363.9 to 488.7)
Other road injuries	1 865 (1 547 to 2 247)	4 394 (3 662 to 5 352)	125.2 (104.3 to 149.9)	144.3 (120.5 to 175.1)
Other transport injuries	1 666 (1 369 to 1 999)	2 027 (1 708 to 2 350)	102.7 (86.3 to 119.9)	67.0 (56.9 to 77.3)
**Unintentional injuries**	**126 904 (118 266 to 137 003)**	**176 629 (163 274 to 193 541)**	**8 423.7 (7 850.1 to 9 095.8)**	**5 954.1 (5 515.4 to 6 510.4)**
Falls	46 909 (41 905 to 53 857)	94 733 (84 902 to 108 823)	3 400.5 (3 041.4 to 3 890.9)	3 281.6 (2 929.5 to 3 757.2)
Drowning	486 (433 to 577)	436 (381 to 511)	33.7 (29.8 to 38.5)	14.6 (12.8 to 17.1)
Fire, heat, and hot substances	26 994 (22 487 to 33 948)	23 251 (19 220 to 29 091)	1 568.1 (1 321.6 to 1 933.1)	752.1 (624.5 to 935.4)
Poisonings	856 (660 to 1 057)	1 039 (762 to 1 294)	44.1 (34.3 to 54.3)	33.0 (24.2 to 41.0)
Poisoning by carbon monoxide	137 (105 to 176)	127 (97 to 164)	7.3 (5.7 to 9.2)	4.1 (3.1 to 5.2)
Poisoning by other means	719 (525 to 910)	912 (639 to 1 159)	36.9 (27.2 to 46.5)	28.9 (20.2 to 36.7)
Exposure to mechanical forces	34 593 (30 260 to 39 760)	38 388 (33 423 to 44 372)	2 292.7 (2 018.3 to 2 625.3)	1 250.3 (1 090.8 to 1 441.5)
Unintentional firearm injuries	5 338 (4 183 to 6 437)	3 641 (2 942 to 4 359)	359.0 (285.7 to 433.1)	119.6 (97.3 to 143.2)
Other exposure to mechanical forces	29 254 (24 622 to 34 767)	34 748 (29 619 to 40 838)	1 933.7 (1 647.2 to 2 277.4)	1 130.7 (967.7 to 1 326.2)
Adverse effects of medical treatment	116 (89 to 145)	204 (155 to 261)	7.2 (5.5 to 9.0)	6.7 (5.1 to 8.6)
Animal contact	4 338 (3 727 to 5 048)	5 026 (4 333 to 5 800)	275.1 (239.7 to 314.4)	165.1 (143.0 to 190.0)
Venomous animal contact	924 (752 to 1 089)	939 (791 to 1 096)	54.8 (45.7 to 63.6)	30.4 (25.7 to 35.4)
Non-venomous animal contact	3 414 (2 840 to 4 109)	4 088 (3 416 to 4 858)	220.3 (185.0 to 259.9)	134.7 (112.9 to 159.6)
Foreign body	3 523 (2 712 to 4 685)	4 380 (3 119 to 6 326)	223.3 (174.1 to 293.5)	148.6 (106.5 to 213.1)
Pulmonary aspiration and foreign body in airway	196 (138 to 267)	140 (112 to 174)	10.3 (7.5 to 13.8)	4.5 (3.6 to 5.6)
Foreign body in eyes	1 348 (687 to 2 381)	2 670 (1 439 to 4 528)	97.7 (55.1 to 160.3)	92.7 (52.0 to 152.3)
Foreign body in other body part	1 985 (1 574 to 2 532)	1 571 (1 285 to 1 943)	115.4 (93.3 to 143.8)	51.3 (42.4 to 63.2)
Environmental heat and cold exposure	3 755 (3 042 to 4 567)	2 624 (2 169 to 3 180)	238.5 (199.6 to 283.0)	88.1 (73.2 to 106.6)
Exposure to forces of nature	355 (253 to 486)	1 484 (1 035 to 2 067)	17.2 (12.6 to 23.1)	46.2 (32.3 to 64.4)
Other unintentional injuries	4 968 (4 248 to 5 778)	5 068 (4 369 to 5 798)	323.2 (280.1 to 368.7)	167.3 (145.7 to 190.3)

Cause	Years lived with disability: All-age counts (95% UI) 1990	Years lived with disability: All-age counts (95% UI) 2017	Years lived with disability: Age-standardised rates per 100,000 (95% UI) 1990	Years lived with disability: Age-standardised rates per 100,000 (95% UI) 2017
**Transport and unintentional injuries**	**6 045 (4 373 to 8 025)**	**9 057 (6 482 to 12 154)**	**398.2 (287.7 to 530.1)**	**304.1 (217.7 to 408.7)**
**Transport injuries**	**1 062 (774 to 1 403)**	**2 134 (1 526 to 2 839)**	**68.9 (50.3 to 90.8)**	**69.0 (49.4 to 92.0)**
Road injuries	956 (696 to 1 262)	2 025 (1 453 to 2 707)	62.7 (45.6 to 82.7)	65.5 (47.0 to 87.4)
Pedestrian road injuries	245 (174 to 331)	393 (277 to 540)	16.2 (11.5 to 21.9)	12.8 (9.0 to 17.7)
Cyclist road injuries	101 (71 to 133)	199 (139 to 265)	6.4 (4.5 to 8.3)	6.5 (4.5 to 8.6)
Motorcyclist road injuries	228 (160 to 310)	400 (272 to 547)	14.7 (10.2 to 20.0)	12.8 (8.7 to 17.4)
Motor vehicle road injuries	276 (199 to 369)	810 (581 to 1 083)	18.7 (13.5 to 24.8)	26.1 (18.7 to 34.9)
Other road injuries	105 (75 to 143)	223 (158 to 309)	6.8 (4.9 to 9.2)	7.2 (5.1 to 10.0)
Other transport injuries	106 (75 to 143)	109 (77 to 147)	6.2 (4.4 to 8.3)	3.5 (2.5 to 4.8)
**Unintentional injuries**	**4 984 (3 611 to 6 651)**	**6 923 (4 906 to 9 369)**	**329.3 (237.4 to 439.2)**	**235.1 (166.5 to 318.3)**
Falls	2 760 (2 020 to 3 620)	4 790 (3 432 to 6 351)	193.8 (140.4 to 253.6)	165.5 (117.9 to 218.2)
Drowning	28 (20 to 39)	23 (16 to 32)	1.8 (1.3 to 2.5)	0.8 (0.5 to 1.1)
Fire, heat, and hot substances	577 (355 to 906)	447 (262 to 726)	32.5 (20.1 to 51.0)	14.4 (8.5 to 23.4)
Poisonings	100 (69 to 150)	120 (71 to 183)	5.0 (3.2 to 7.4)	3.8 (2.2 to 5.8)
Poisoning by carbon monoxide	15 (9 to 23)	12 (7 to 20)	0.7 (0.5 to 1.1)	0.4 (0.2 to 0.6)
Poisoning by other means	86 (52 to 129)	107 (63 to 165)	4.3 (2.6 to 6.4)	3.4 (2.0 to 5.2)
Exposure to mechanical forces	812 (561 to 1 163)	827 (557 to 1 209)	52.6 (36.1 to 75.4)	26.9 (18.1 to 39.1)
Unintentional firearm injuries	113 (75 to 170)	68 (44 to 106)	7.3 (4.8 to 11.2)	2.2 (1.4 to 3.5)
Other exposure to mechanical forces	699 (482 to 1 005)	759 (512 to 1 109)	45.3 (31.2 to 64.9)	24.6 (16.6 to 36.0)
Adverse effects of medical treatment	15 (9 to 24)	27 (17 to 41)	1.0 (0.6 to 1.5)	0.9 (0.6 to 1.4)
Animal contact	113 (76 to 161)	115 (76 to 167)	6.5 (4.4 to 9.2)	3.7 (2.5 to 5.4)
Venomous animal contact	61 (39 to 89)	58 (37 to 86)	3.2 (2.1 to 4.6)	1.8 (1.2 to 2.7)
Non-venomous animal contact	52 (31 to 83)	57 (33 to 93)	3.3 (2.0 to 5.2)	1.9 (1.1 to 3.1)
Foreign body	219 (148 to 326)	257 (155 to 425)	13.6 (9.0 to 20.1)	8.7 (5.3 to 14.3)
Pulmonary aspiration and foreign body in airway	10 (7 to 15)	6 (4 to 8)	0.5 (0.3 to 0.7)	0.2 (0.1 to 0.3)
Foreign body in eyes	89 (45 to 162)	178 (93 to 328)	6.5 (3.5 to 11.6)	6.2 (3.2 to 11.1)
Foreign body in other body part	119 (82 to 161)	73 (52 to 97)	6.6 (4.6 to 8.7)	2.4 (1.7 to 3.1)
Environmental heat and cold exposure	147 (98 to 208)	98 (63 to 142)	9.3 (6.2 to 12.9)	3.3 (2.1 to 4.8)
Exposure to forces of nature	19 (13 to 26)	48 (34 to 67)	0.9 (0.6 to 1.2)	1.5 (1.1 to 2.1)
Other unintentional injuries	193 (139 to 258)	170 (119 to 238)	12.2 (8.8 to 16.4)	5.6 (3.9 to 7.7)

Cause	Disability adjusted life years: All-age counts (95% UI) 1990	Disability adjusted life years: All-age counts (95% UI) 2017	Disability adjusted life years: Age-standardised rates per 100,000 (95% UI) 1990	Disability adjusted life years: Age-standardised rates per 100,000 (95% UI) 2017
**Transport and unintentional injuries**	**61 335 (57 426 to 65 437)**	**51 911 (47 551 to 56 377)**	**3 103.9 (2 933.6 to 3 293.5)**	**1 675.4 (1 533.1 to 1 819.6)**
**Transport injuries**	**26 318 (24 697 to 28 119)**	**24 530 (22 521 to 26 620)**	**1 293.4 (1 222.6 to 1 371.5)**	**767.0 (704.7 to 832.0)**
Road injuries	25 532 (23 933 to 27 228)	23 422 (21 504 to 25 464)	1 251.1 (1 183.4 to 1 325.2)	732.4 (673.2 to 794.8)
Pedestrian road injuries	10 073 (9 125 to 11 107)	7 959 (7 270 to 8 781)	505.2 (467.6 to 547.0)	253.2 (231.5 to 279.1)
Cyclist road injuries	758 (683 to 846)	1 095 (973 to 1 230)	38.9 (35.4 to 43.1)	34.6 (30.7 to 38.7)
Motorcyclist road injuries	4 433 (3 985 to 4 881)	3 829 (3 350 to 4 317)	210.9 (191.1 to 230.7)	117.3 (102.8 to 132.1)
Motor vehicle road injuries	9 697 (8 894 to 10 545)	9 913 (8 993 to 10 960)	466.9 (432.4 to 502.1)	307.5 (278.8 to 339.5)
Other road injuries	571 (498 to 658)	626 (537 to 722)	29.2 (25.6 to 33.0)	19.8 (17.0 to 23.0)
Other transport injuries	826 (744 to 917)	1 109 (978 to 1 254)	42.3 (38.5 to 46.6)	34.7 (30.6 to 39.1)
**Unintentional injuries**	**34 978 (32 279 to 37 901)**	**27 381 (24 536 to 30 447)**	**1 810.5 (1 681.3 to 1 949.6)**	**908.4 (812.7 to 1 011.5)**
Falls	6 536 (5 778 to 7 413)	8 459 (7 045 to 10 068)	452.0 (400.9 to 511.5)	293.4 (245.1 to 347.7)
Drowning	7 426 (6 689 to 8 296)	3 551 (3 160 to 3 959)	330.0 (301.4 to 364.5)	112.7 (100.2 to 125.5)
Fire, heat, and hot substances	2 977 (2 613 to 3 429)	1 652 (1 428 to 1 948)	153.8 (136.8 to 176.6)	54.7 (47.5 to 64.6)
Poisonings	1 981 (1 780 to 2 217)	1 149 (1 041 to 1 269)	90.7 (82.8 to 99.9)	36.7 (33.2 to 40.5)
Poisoning by carbon monoxide	889 (794 to 1 007)	558 (499 to 621)	41.1 (37.2 to 45.4)	17.8 (15.9 to 19.8)
Poisoning by other means	1 092 (966 to 1 241)	590 (519 to 674)	49.6 (44.5 to 55.7)	18.9 (16.6 to 21.6)
Exposure to mechanical forces	5 492 (5 061 to 5 958)	3 193 (2 838 to 3 645)	282.3 (259.5 to 308.0)	101.1 (89.8 to 115.5)
Unintentional firearm injuries	2 518 (2 290 to 2 955)	986 (867 to 1 130)	120.7 (110.0 to 141.9)	30.6 (27.0 to 35.0)
Other exposure to mechanical forces	2 974 (2 465 to 3 334)	2 208 (1 908 to 2 599)	161.6 (136.1 to 182.7)	70.5 (60.9 to 83.0)
Adverse effects of medical treatment	1 447 (1 182 to 1 637)	1 443 (1 290 to 1 804)	80.0 (65.4 to 88.2)	49.1 (43.9 to 60.8)
Animal contact	505 (425 to 595)	259 (214 to 312)	24.0 (20.3 to 27.9)	8.5 (7.0 to 10.2)
Venomous animal contact	314 (257 to 380)	125 (97 to 159)	13.9 (11.5 to 16.7)	4.1 (3.2 to 5.2)
Non-venomous animal contact	192 (157 to 232)	134 (105 to 174)	10.1 (8.2 to 12.4)	4.4 (3.5 to 5.7)
Foreign body	6 225 (5 431 to 7 099)	6 163 (5 243 to 7 334)	273.4 (243.0 to 307.1)	204.5 (173.4 to 244.6)
Pulmonary aspiration and foreign body in airway	5 707 (4 947 to 6 554)	5 763 (4 843 to 6 932)	246.6 (217.7 to 279.0)	191.3 (160.2 to 230.7)
Foreign body in eyes	89 (45 to 162)	178 (93 to 328)	6.5 (3.5 to 11.6)	6.2 (3.2 to 11.1)
Foreign body in other body part	428 (335 to 495)	221 (188 to 286)	20.3 (16.1 to 23.2)	7.0 (5.9 to 9.1)
Environmental heat and cold exposure	725 (649 to 805)	375 (330 to 432)	43.0 (38.9 to 47.7)	12.2 (10.7 to 14.1)
Exposure to forces of nature	75 (67 to 84)	74 (60 to 92)	3.4 (3.1 to 3.8)	2.3 (1.9 to 2.9)
Other unintentional injuries	1 588 (1 454 to 1 733)	1 064 (946 to 1 191)	78.0 (71.8 to 84.7)	33.2 (29.5 to 37.2)

Death, YLL, incidence, prevalence, YLD, and DALY counts and rates per 100,000 for all injuries in Colima in 1990 and 2017

Deaths

Cause	All-age counts (95% UI) 1990	All-age counts (95% UI) 2017	Age-standardised rates per 100,000 (95% UI) 1990	Age-standardised rates per 100,000 (95% UI) 2017
**Transport and unintentional injuries**	**302 (288 to 318)**	**288 (269 to 309)**	**87.4 (84.3 to 90.8)**	**41.1 (38.4 to 43.9)**
**Transport injuries**	**143 (135 to 150)**	**146 (134 to 159)**	**38.0 (36.2 to 39.8)**	**19.4 (17.8 to 21.1)**
Road injuries	136 (128 to 143)	135 (124 to 148)	36.2 (34.5 to 37.9)	18.0 (16.6 to 19.6)
Pedestrian road injuries	68 (63 to 73)	61 (56 to 67)	19.5 (18.5 to 20.7)	8.5 (7.8 to 9.3)
Cyclist road injuries	3 (3 to 4)	6 (5 to 7)	0.9 (0.8 to 1.0)	0.8 (0.7 to 0.9)
Motorcyclist road injuries	20 (18 to 22)	18 (16 to 21)	4.7 (4.3 to 5.2)	2.3 (2.0 to 2.6)
Motor vehicle road injuries	42 (38 to 45)	47 (42 to 53)	10.2 (9.4 to 11.0)	6.0 (5.4 to 6.6)
Other road injuries	3 (3 to 4)	3 (3 to 4)	0.8 (0.7 to 1.0)	0.4 (0.4 to 0.5)
Other transport injuries	6 (6 to 7)	11 (9 to 12)	1.8 (1.6 to 2.0)	1.4 (1.1 to 1.6)
**Unintentional injuries**	**160 (150 to 170)**	**142 (133 to 151)**	**49.4 (47.3 to 51.6)**	**21.6 (20.4 to 22.9)**
Falls	34 (32 to 35)	47 (42 to 53)	15.5 (14.7 to 16.3)	8.0 (7.3 to 8.6)
Drowning	42 (38 to 47)	25 (23 to 28)	10.0 (9.2 to 10.8)	3.4 (3.1 to 3.7)
Fire, heat, and hot substances	11 (10 to 12)	8 (7 to 9)	3.6 (3.4 to 3.8)	1.3 (1.2 to 1.4)
Poisonings	5 (4 to 5)	3 (3 to 4)	1.2 (1.1 to 1.3)	0.5 (0.4 to 0.5)
Poisoning by carbon monoxide	1 (1 to 1)	1 (1 to 1)	0.3 (0.2 to 0.3)	0.1 (0.1 to 0.1)
Poisoning by other means	4 (3 to 4)	2 (2 to 3)	0.9 (0.8 to 1.0)	0.3 (0.3 to 0.4)
Exposure to mechanical forces	17 (16 to 18)	11 (10 to 13)	4.7 (4.4 to 5.0)	1.6 (1.4 to 1.7)
Unintentional firearm injuries	8 (7 to 9)	4 (3 to 5)	2.1 (1.9 to 2.3)	0.5 (0.4 to 0.6)
Other exposure to mechanical forces	9 (8 to 10)	7 (7 to 9)	2.6 (2.4 to 2.8)	1.0 (0.9 to 1.2)
Adverse effects of medical treatment	10 (7 to 11)	14 (12 to 16)	4.1 (2.6 to 4.4)	2.3 (1.9 to 2.6)
Animal contact	13 (11 to 16)	7 (6 to 7)	3.3 (2.9 to 3.7)	1.0 (0.9 to 1.1)
Venomous animal contact	12 (10 to 15)	6 (5 to 6)	2.9 (2.5 to 3.4)	0.9 (0.7 to 0.9)
Non-venomous animal contact	1 (1 to 2)	1 (1 to 1)	0.3 (0.2 to 0.6)	0.1 (0.1 to 0.2)
Foreign body	18 (16 to 20)	18 (16 to 19)	4.3 (4.0 to 4.7)	2.6 (2.4 to 2.8)
Pulmonary aspiration and foreign body in airway	17 (15 to 19)	17 (16 to 19)	4.2 (3.8 to 4.5)	2.5 (2.3 to 2.8)
Foreign body in eyes				
Foreign body in other body part	1 (1 to 1)	0 (0 to 1)	0.2 (0.1 to 0.2)	0.1 (0.0 to 0.1)
Environmental heat and cold exposure	2 (2 to 2)	1 (1 to 1)	0.7 (0.7 to 0.8)	0.2 (0.2 to 0.2)
Exposure to forces of nature	0 (0 to 0)	0 (0 to 0)	0.1 (0.1 to 0.1)	0.0 (0.0 to 0.0)
Other unintentional injuries	8 (7 to 9)	7 (6 to 7)	2.0 (1.8 to 2.1)	0.8 (0.7 to 1.0)

Years of life lost

Cause	All-age counts (95% UI) 1990	All-age counts (95% UI) 2017	Age-standardised rates per 100,000 (95% UI) 1990	Age-standardised rates per 100,000 (95% UI) 2017
**Transport and unintentional injuries**	**16 406 (15 258 to 17 707)**	**11 916 (11 026 to 12 849)**	**3 683.6 (3 478.4 to 3 910.5)**	**1 573.8 (1 458.0 to 1 694.2)**
**Transport injuries**	**7 836 (7 324 to 8 365)**	**6 748 (6 148 to 7 372)**	**1 771.7 (1 671.4 to 1 871.5)**	**866.0 (790.2 to 947.5)**
Road injuries	7 492 (7 002 to 8 007)	6 253 (5 695 to 6 839)	1 691.4 (1 593.8 to 1 789.7)	803.2 (733.5 to 879.3)
Pedestrian road injuries	3 577 (3 247 to 3 957)	2 503 (2 262 to 2 779)	829.9 (760.2 to 895.2)	328.8 (297.9 to 364.1)
Cyclist road injuries	192 (171 to 214)	270 (236 to 306)	44.1 (39.8 to 48.9)	34.8 (30.4 to 39.3)
Motorcyclist road injuries	1 172 (1 043 to 1 316)	997 (861 to 1 150)	256.5 (231.2 to 284.2)	124.6 (107.8 to 143.6)
Motor vehicle road injuries	2 375 (2 164 to 2 577)	2 328 (2 084 to 2 582)	527.4 (484.7 to 568.3)	295.2 (264.2 to 327.5)
Other road injuries	177 (149 to 207)	155 (132 to 182)	39.5 (33.7 to 45.7)	19.9 (17.0 to 23.4)
Other transport injuries	344 (306 to 385)	494 (382 to 578)	80.4 (72.5 to 88.9)	62.8 (49.0 to 73.1)
**Unintentional injuries**	**8 570 (7 761 to 9 431)**	**5 168 (4 786 to 5 584)**	**1 911.8 (1 773.7 to 2 061.1)**	**707.8 (656.4 to 763.6)**
Falls	1 071 (1 001 to 1 148)	1 067 (904 to 1 168)	325.7 (308.6 to 344.6)	152.2 (130.8 to 166.0)
Drowning	2 635 (2 341 to 2 986)	1 264 (1 134 to 1 415)	539.9 (486.8 to 600.6)	167.5 (150.5 to 187.8)
Fire, heat, and hot substances	557 (482 to 647)	256 (218 to 281)	124.2 (111.0 to 140.2)	35.6 (30.8 to 38.9)
Poisonings	289 (256 to 328)	153 (122 to 171)	60.1 (54.0 to 67.2)	20.2 (16.4 to 22.7)
Poisoning by carbon monoxide	65 (56 to 75)	43 (32 to 51)	13.9 (12.1 to 15.9)	5.7 (4.3 to 6.6)
Poisoning by other means	224 (196 to 258)	109 (90 to 123)	46.2 (41.3 to 52.1)	14.6 (12.1 to 16.4)
Exposure to mechanical forces	927 (848 to 1 010)	498 (446 to 559)	215.3 (199.7 to 231.5)	63.9 (57.5 to 71.6)
Unintentional firearm injuries	450 (398 to 503)	190 (145 to 225)	102.0 (90.9 to 113.7)	24.0 (18.3 to 28.3)
Other exposure to mechanical forces	477 (419 to 534)	308 (271 to 374)	113.4 (100.2 to 124.8)	39.9 (35.3 to 48.1)
Adverse effects of medical treatment	453 (323 to 523)	402 (340 to 454)	111.8 (77.5 to 125.9)	57.9 (49.3 to 65.7)
Animal contact	891 (723 to 1 101)	268 (240 to 300)	174.6 (146.1 to 210.2)	37.7 (33.6 to 42.2)
Venomous animal contact	831 (663 to 1 037)	229 (199 to 259)	161.2 (132.0 to 196.9)	32.4 (28.1 to 36.8)
Non-venomous animal contact	60 (38 to 127)	39 (30 to 57)	13.4 (8.3 to 26.1)	5.3 (4.1 to 7.9)
Foreign body	1 202 (1 094 to 1 389)	887 (779 to 1 013)	236.2 (207.1 to 268.6)	125.1 (108.8 to 145.0)
Pulmonary aspiration and foreign body in airway	1 148 (986 to 1 327)	863 (758 to 988)	225.3 (197.8 to 256.4)	122.0 (105.8 to 141.4)
Foreign body in eyes				
Foreign body in other body part	54 (37 to 64)	23 (19 to 37)	10.9 (7.6 to 12.8)	3.0 (2.4 to 4.8)
Environmental heat and cold exposure	90 (82 to 98)	43 (35 to 48)	23.5 (21.7 to 25.2)	5.7 (4.7 to 6.3)
Exposure to forces of nature	13 (12 to 15)	6 (6 to 7)	2.8 (2.6 to 3.1)	0.8 (0.7 to 0.9)
Other unintentional injuries	441 (399 to 491)	326 (284 to 375)	97.6 (89.3 to 107.7)	41.2 (36.0 to 47.3)

Incidence

Cause	All-age counts (95% UI) 1990	All-age counts (95% UI) 2017	Age-standardised rates per 100,000 (95% UI) 1990	Age-standardised rates per 100,000 (95% UI) 2017
**Transport and unintentional injuries**	**18 432 (16 972 to 20 183)**	**29 757 (26 878 to 32 982)**	**4 847.0 (4 444.9 to 5 295.7)**	**4 138.4 (3 748.3 to 4 567.6)**
**Transport injuries**	**1 664 (1 442 to 1 928)**	**3 728 (3 256 to 4 278)**	**414.8 (362.9 to 470.4)**	**485.2 (424.2 to 556.0)**
Road injuries	1 557 (1 346 to 1 822)	3 610 (3 136 to 4 154)	388.3 (339.7 to 444.5)	469.0 (408.3 to 538.9)
Pedestrian road injuries	274 (221 to 336)	390 (310 to 489)	71.5 (59.1 to 85.5)	51.7 (41.2 to 64.2)
Cyclist road injuries	207 (157 to 269)	394 (309 to 502)	48.5 (38.4 to 61.2)	53.0 (41.6 to 67.3)
Motorcyclist road injuries	209 (166 to 262)	426 (331 to 536)	49.5 (39.9 to 60.5)	54.8 (42.8 to 68.7)
Motor vehicle road injuries	587 (484 to 699)	1 802 (1 556 to 2 139)	149.9 (125.0 to 175.6)	230.8 (199.1 to 272.2)
Other road injuries	279 (216 to 353)	598 (470 to 743)	69.5 (55.2 to 84.2)	78.7 (62.1 to 96.6)
Other transport injuries	107 (85 to 139)	118 (90 to 153)	26.5 (22.0 to 32.0)	16.2 (12.4 to 21.1)
**Unintentional injuries**	**16 768 (15 340 to 18 530)**	**26 029 (23 289 to 29 214)**	**4 432.1 (4 031.3 to 4 884.1)**	**3 653.2 (3 281.9 to 4 087.1)**
Falls	7 152 (6 282 to 8 143)	14 679 (13 083 to 16 621)	2 087.8 (1 865.4 to 2 346.1)	2 130.0 (1 899.2 to 2 410.5)
Drowning	32 (28 to 36)	21 (17 to 25)	7.5 (6.6 to 8.5)	2.8 (2.3 to 3.4)
Fire, heat, and hot substances	588 (468 to 745)	497 (394 to 623)	116.5 (94.8 to 143.6)	70.1 (55.4 to 87.5)
Poisonings	171 (124 to 241)	165 (115 to 232)	31.3 (23.0 to 42.7)	23.7 (16.4 to 33.9)
Poisoning by carbon monoxide	35 (22 to 56)	48 (24 to 78)	7.1 (4.6 to 10.6)	6.7 (3.3 to 11.1)
Poisoning by other means	136 (95 to 197)	117 (76 to 173)	24.2 (17.4 to 34.5)	17.0 (11.0 to 25.4)
Exposure to mechanical forces	3 435 (2 921 to 4 078)	4 242 (3 646 to 4 907)	800.2 (687.5 to 929.3)	555.5 (480.7 to 641.6)
Unintentional firearm injuries	256 (189 to 343)	165 (117 to 230)	58.3 (43.6 to 76.5)	21.5 (15.4 to 29.7)
Other exposure to mechanical forces	3 178 (2 696 to 3 778)	4 077 (3 491 to 4 728)	741.9 (637.2 to 864.6)	534.0 (460.8 to 629.1)
Adverse effects of medical treatment	383 (328 to 439)	857 (727 to 997)	110.7 (95.8 to 125.8)	115.3 (97.9 to 133.5)
Animal contact	2 260 (1 912 to 2 675)	1 234 (1 007 to 1 488)	544.8 (473.5 to 623.9)	173.2 (140.4 to 205.5)
Venomous animal contact	1 003 (782 to 1 260)	476 (358 to 607)	241.2 (191.8 to 293.7)	65.8 (49.9 to 84.2)
Non-venomous animal contact	1 257 (1 014 to 1 562)	758 (597 to 955)	303.6 (249.9 to 367.6)	105.4 (83.4 to 132.2)
Foreign body	1 659 (843 to 2 856)	3 250 (1 253 to 5 689)	473.4 (210.6 to 827.5)	436.8 (168.3 to 745.2)
Pulmonary aspiration and foreign body in airway	47 (33 to 65)	32 (23 to 44)	9.0 (6.6 to 12.2)	4.6 (3.3 to 6.3)
Foreign body in eyes	1 495 (676 to 2 684)	3 111 (1 108 to 5 558)	439.8 (175.6 to 789.9)	417.0 (147.5 to 725.5)
Foreign body in other body part	117 (80 to 173)	108 (77 to 142)	24.6 (17.4 to 34.2)	15.1 (10.8 to 20.1)
Environmental heat and cold exposure	217 (153 to 298)	163 (116 to 222)	51.3 (39.0 to 65.6)	23.1 (16.7 to 31.6)
Exposure to forces of nature	35 (29 to 44)	8 (7 to 10)	7.2 (6.1 to 8.7)	1.1 (0.9 to 1.3)
Other unintentional injuries	835 (692 to 1 032)	912 (747 to 1 104)	201.0 (170.8 to 238.0)	123.6 (101.5 to 148.9)

Prevalence

Cause	All-age counts (95% UI) 1990	All-age counts (95% UI) 2017	Age-standardised rates per 100,000 (95% UI) 1990	Age-standardised rates per 100,000 (95% UI) 2017
**Transport and unintentional injuries**	**34 506 (32 375 to 36 992)**	**61 967 (58 119 to 67 020)**	**10 661.6 (10 070.2 to 11 450.5)**	**8 479.1 (7 969.7 to 9 157.2)**
**Transport injuries**	**4 083 (3 805 to 4 353)**	**10 832 (10 063 to 11 667)**	**1 294.2 (1 208.7 to 1 382.5)**	**1 435.0 (1 331.9 to 1 543.7)**
Road injuries	3 600 (3 346 to 3 856)	10 214 (9 466 to 11 029)	1 149.2 (1 073.9 to 1 232.1)	1 351.5 (1 251.9 to 1 458.9)
Pedestrian road injuries	998 (854 to 1 176)	1 805 (1 557 to 2 112)	321.1 (278.6 to 375.1)	242.8 (210.4 to 283.3)
Cyclist road injuries	341 (283 to 407)	880 (742 to 1 028)	103.6 (86.7 to 120.0)	117.2 (99.2 to 136.2)
Motorcyclist road injuries	737 (639 to 853)	1 967 (1 688 to 2 298)	230.8 (199.7 to 266.7)	256.4 (220.2 to 298.3)
Motor vehicle road injuries	1 019 (888 to 1 190)	4 120 (3 601 to 4 814)	335.8 (290.8 to 391.9)	543.5 (474.5 to 634.7)
Other road injuries	505 (420 to 609)	1 441 (1 203 to 1 761)	157.9 (132.3 to 189.2)	191.6 (160.0 to 233.3)
Other transport injuries	484 (402 to 565)	618 (528 to 714)	145.1 (123.7 to 167.4)	83.5 (71.7 to 95.9)
**Unintentional injuries**	**30 423 (28 342 to 32 872)**	**51 134 (47 439 to 55 931)**	**9 367.4 (8 765.7 to 10 110.9)**	**7 044.1 (6 531.8 to 7 716.3)**
Falls	12 483 (10 964 to 14 260)	28 361 (25 288 to 32 510)	4 139.2 (3 684.5 to 4 704.9)	4 024.6 (3 593.5 to 4 579.0)
Drowning	146 (128 to 168)	127 (112 to 147)	46.6 (41.2 to 53.0)	17.4 (15.3 to 20.0)
Fire, heat, and hot substances	5 704 (4 744 to 7 268)	6 172 (5 102 to 7 869)	1 535.2 (1 289.1 to 1 897.3)	818.3 (672.8 to 1 080.6)
Poisonings	152 (116 to 190)	204 (151 to 254)	36.5 (28.2 to 45.3)	26.7 (19.8 to 33.2)
Poisoning by carbon monoxide	25 (19 to 32)	42 (32 to 54)	6.1 (4.7 to 7.7)	5.5 (4.2 to 7.1)
Poisoning by other means	127 (92 to 163)	162 (114 to 209)	30.4 (22.1 to 38.7)	21.2 (14.9 to 27.3)
Exposure to mechanical forces	6 716 (5 886 to 7 701)	10 656 (9 281 to 12 359)	2 090.5 (1 842.5 to 2 384.3)	1 405.3 (1 226.8 to 1 624.4)
Unintentional firearm injuries	938 (747 to 1 122)	862 (704 to 1 032)	297.4 (239.0 to 358.6)	114.6 (94.1 to 137.1)
Other exposure to mechanical forces	5 778 (4 899 to 6 819)	9 793 (8 383 to 11 525)	1 793.0 (1 534.9 to 2 107.2)	1 290.7 (1 107.3 to 1 512.7)
Adverse effects of medical treatment	29 (23 to 36)	66 (50 to 82)	8.5 (6.5 to 10.4)	8.8 (6.7 to 11.0)
Animal contact	2 353 (2 035 to 2 719)	1 505 (1 319 to 1 707)	698.1 (614.4 to 789.1)	205.2 (180.6 to 232.9)
Venomous animal contact	718 (591 to 870)	348 (291 to 401)	202.9 (170.7 to 240.2)	47.1 (39.7 to 54.5)
Non-venomous animal contact	1 635 (1 387 to 1 973)	1 157 (980 to 1 357)	495.2 (412.9 to 585.3)	158.1 (133.7 to 184.8)
Foreign body	710 (538 to 969)	1 122 (798 to 1 624)	208.9 (159.8 to 283.3)	154.2 (110.1 to 219.9)
Pulmonary aspiration and foreign body in airway	34 (24 to 46)	31 (25 to 38)	8.3 (6.1 to 11.2)	4.1 (3.3 to 5.0)
Foreign body in eyes	300 (156 to 543)	672 (360 to 1 161)	100.1 (55.7 to 168.7)	94.1 (52.0 to 157.3)
Foreign body in other body part	376 (300 to 484)	420 (346 to 520)	99.9 (80.9 to 125.8)	56.0 (46.4 to 69.7)
Environmental heat and cold exposure	780 (631 to 955)	676 (556 to 804)	223.4 (186.5 to 268.0)	92.0 (77.5 to 109.1)
Exposure to forces of nature	140 (100 to 192)	618 (434 to 864)	34.1 (24.7 to 46.8)	78.8 (55.5 to 110.0)
Other unintentional injuries	1 210 (1 088 to 1 403)	1 629 (1 412 to 1 865)	367.9 (318.6 to 420.4)	218.0 (190.1 to 247.8)

Years lived with disability

Cause	All-age counts (95% UI) 1990	All-age counts (95% UI) 2017	Age-standardised rates per 100,000 (95% UI) 1990	Age-standardised rates per 100,000 (95% UI) 2017
**Transport and unintentional injuries**	**1 525 (1 108 to 2 025)**	**2 688 (1 945 to 3 600)**	**464.5 (338.8 to 614.7)**	**368.6 (265.9 to 495.3)**
**Transport injuries**	**277 (201 to 367)**	**656 (477 to 871)**	**84.5 (61.8 to 111.6)**	**85.9 (62.5 to 114.2)**
Road injuries	247 (179 to 328)	623 (452 to 827)	75.8 (55.4 to 100.6)	81.5 (59.2 to 108.4)
Pedestrian road injuries	72 (51 to 97)	115 (81 to 159)	22.0 (15.8 to 29.8)	15.3 (10.8 to 21.1)
Cyclist road injuries	24 (17 to 31)	53 (37 to 70)	6.9 (5.0 to 9.1)	7.0 (4.9 to 9.3)
Motorcyclist road injuries	51 (35 to 69)	121 (83 to 165)	15.4 (10.7 to 20.9)	15.6 (10.8 to 21.3)
Motor vehicle road injuries	72 (52 to 96)	261 (188 to 348)	22.8 (16.5 to 30.4)	34.0 (24.6 to 45.4)
Other road injuries	28 (20 to 38)	73 (52 to 100)	8.5 (6.2 to 11.6)	9.6 (6.9 to 13.1)
Other transport injuries	31 (22 to 40)	33 (23 to 44)	8.7 (6.3 to 11.5)	4.4 (3.1 to 5.9)
**Unintentional injuries**	**1 247 (906 to 1 666)**	**2 033 (1 451 to 2 733)**	**380.0 (274.5 to 507.9)**	**282.7 (201.5 to 380.8)**
Falls	743 (544 to 973)	1 446 (1 036 to 1 915)	237.6 (173.4 to 309.1)	204.7 (145.9 to 272.5)
Drowning	8 (6 to 11)	7 (5 to 9)	2.5 (1.8 to 3.5)	0.9 (0.6 to 1.3)
Fire, heat, and hot substances	123 (77 to 193)	119 (70 to 192)	32.1 (20.1 to 50.1)	15.7 (9.3 to 25.3)
Poisonings	18 (11 to 27)	23 (14 to 35)	4.2 (2.6 to 6.3)	3.1 (1.8 to 4.6)
Poisoning by carbon monoxide	3 (2 to 4)	4 (3 to 7)	0.6 (0.4 to 1.0)	0.6 (0.3 to 0.9)
Poisoning by other means	15 (9 to 24)	19 (11 to 29)	3.6 (2.1 to 5.4)	2.5 (1.4 to 3.8)
Exposure to mechanical forces	158 (108 to 224)	230 (154 to 335)	48.1 (33.1 to 68.9)	30.2 (20.3 to 44.1)
Unintentional firearm injuries	19 (13 to 30)	16 (10 to 25)	5.9 (3.9 to 9.1)	2.1 (1.3 to 3.3)
Other exposure to mechanical forces	139 (95 to 197)	214 (144 to 314)	42.2 (28.9 to 60.3)	28.1 (18.9 to 41.2)
Adverse effects of medical treatment	4 (2 to 6)	9 (5 to 13)	1.1 (0.7 to 1.7)	1.2 (0.7 to 1.8)
Animal contact	64 (43 to 90)	34 (23 to 50)	17.0 (11.5 to 24.0)	4.6 (3.0 to 6.7)
Venomous animal contact	39 (25 to 57)	18 (11 to 26)	9.6 (6.3 to 13.7)	2.4 (1.5 to 3.4)
Non-venomous animal contact	25 (15 to 40)	16 (10 to 27)	7.4 (4.5 to 12.0)	2.2 (1.3 to 3.7)
Foreign body	45 (30 to 68)	66 (39 to 109)	12.9 (8.5 to 19.6)	9.0 (5.4 to 14.8)
Pulmonary aspiration and foreign body in airway	2 (1 to 3)	1 (1 to 2)	0.4 (0.3 to 0.6)	0.2 (0.1 to 0.2)
Foreign body in eyes	20 (10 to 37)	45 (23 to 84)	6.7 (3.6 to 12.2)	6.8 (3.3 to 11.5)
Foreign body in other body part	23 (16 to 31)	20 (14 to 26)	5.8 (4.1 to 7.7)	2.6 (1.8 to 3.5)
Environmental heat and cold exposure	31 (20 to 44)	25 (17 to 37)	8.7 (5.8 to 12.2)	3.5 (2.3 to 5.0)
Exposure to forces of nature	7 (5 to 11)	20 (15 to 28)	1.8 (1.3 to 2.5)	2.5 (1.9 to 3.6)
Other unintentional injuries	47 (34 to 63)	54 (38 to 75)	13.9 (10.0 to 18.6)	7.3 (5.2 to 10.0)

Disability adjusted life years

Cause	All-age counts (95% UI) 1990	All-age counts (95% UI) 2017	Age-standardised rates per 100,000 (95% UI) 1990	Age-standardised rates per 100,000 (95% UI) 2017
**Transport and unintentional injuries**	**17 930 (16 676 to 19 280)**	**14 604 (13 421 to 15 920)**	**4 148.1 (3 906.4 to 4 421.2)**	**1 942.4 (1 785.4 to 2 120.6)**
**Transport injuries**	**8 113 (7 599 to 8 655)**	**7 403 (6 777 to 8 060)**	**1 856.3 (1 750.3 to 1 962.9)**	**951.9 (873.1 to 1 035.2)**
Road injuries	7 739 (7 236 to 8 265)	6 876 (6 305 to 7 497)	1 767.1 (1 664.3 to 1 867.6)	884.7 (811.7 to 963.1)
Pedestrian road injuries	3 648 (3 318 to 4 028)	2 618 (2 378 to 2 885)	845.9 (781.9 to 917.2)	344.1 (313.4 to 377.9)
Cyclist road injuries	215 (194 to 241)	323 (285 to 365)	51.0 (46.3 to 56.3)	41.8 (37.0 to 47.1)
Motorcyclist road injuries	1 223 (1 095 to 1 366)	1 118 (975 to 1 274)	271.9 (245.6 to 300.3)	140.2 (122.6 to 159.7)
Motor vehicle road injuries	2 447 (2 237 to 2 654)	2 589 (2 338 to 2 854)	550.2 (507.4 to 590.9)	329.2 (297.8 to 362.6)
Other road injuries	206 (176 to 236)	228 (194 to 267)	48.1 (41.6 to 54.6)	29.5 (25.2 to 34.5)
Other transport injuries	374 (337 to 417)	527 (416 to 610)	89.1 (81.5 to 97.8)	67.2 (53.3 to 77.4)
**Unintentional injuries**	**9 817 (8 946 to 10 813)**	**7 201 (6 519 to 8 031)**	**2 291.9 (2 112.1 to 2 491.2)**	**990.4 (897.6 to 1 106.6)**
Falls	1 814 (1 600 to 2 067)	2 512 (2 089 to 3 002)	563.3 (496.8 to 639.2)	356.9 (296.6 to 426.2)
Drowning	2 643 (2 351 to 2 996)	1 270 (1 139 to 1 420)	542.5 (489.3 to 602.9)	168.4 (151.4 to 188.7)
Fire, heat, and hot substances	680 (588 to 792)	375 (318 to 449)	156.4 (137.3 to 180.5)	51.2 (43.4 to 61.2)
Poisonings	307 (271 to 347)	176 (145 to 198)	64.3 (57.7 to 71.4)	23.3 (19.2 to 26.2)
Poisoning by carbon monoxide	67 (58 to 78)	48 (36 to 56)	14.5 (12.7 to 16.5)	6.2 (4.8 to 7.2)
Poisoning by other means	239 (211 to 273)	129 (108 to 145)	49.8 (44.6 to 55.7)	17.1 (14.4 to 19.3)
Exposure to mechanical forces	1 085 (990 to 1 197)	728 (633 to 850)	263.5 (241.2 to 290.8)	94.1 (82.1 to 109.8)
Unintentional firearm injuries	469 (416 to 522)	206 (160 to 242)	107.9 (96.6 to 120.0)	26.1 (20.4 to 30.5)
Other exposure to mechanical forces	616 (545 to 701)	521 (440 to 637)	155.6 (137.6 to 178.7)	68.1 (57.5 to 82.9)
Adverse effects of medical treatment	457 (327 to 528)	410 (347 to 464)	112.9 (78.6 to 127.0)	59.1 (50.2 to 67.1)
Animal contact	955 (784 to 1 159)	303 (272 to 339)	191.6 (162.5 to 226.1)	42.3 (37.8 to 47.4)
Venomous animal contact	870 (709 to 1 071)	247 (218 to 278)	170.8 (140.6 to 205.4)	34.7 (30.4 to 39.2)
Non-venomous animal contact	85 (57 to 158)	55 (43 to 77)	20.8 (14.0 to 35.5)	7.5 (5.8 to 10.5)
Foreign body	1 247 (1 075 to 1 433)	952 (839 to 1 087)	249.2 (218.8 to 282.1)	134.1 (117.0 to 154.4)
Pulmonary aspiration and foreign body in airway	1 150 (988 to 1 329)	865 (759 to 989)	225.8 (198.2 to 256.8)	122.2 (105.9 to 141.6)
Foreign body in eyes	20 (10 to 37)	45 (23 to 84)	6.7 (3.6 to 12.2)	6.8 (3.3 to 11.5)
Foreign body in other body part	77 (60 to 91)	43 (35 to 58)	16.7 (13.2 to 19.4)	5.6 (4.6 to 7.7)
Environmental heat and cold exposure	120 (107 to 136)	69 (57 to 83)	32.2 (28.6 to 36.2)	9.2 (7.7 to 11.1)
Exposure to forces of nature	21 (18 to 24)	26 (21 to 34)	4.6 (4.1 to 5.4)	3.4 (2.7 to 4.4)
Other unintentional injuries	488 (444 to 539)	380 (337 to 433)	111.5 (102.3 to 122.1)	48.4 (43.1 to 54.9)

Death, YLL, incidence, prevalence, YLD, and DALY counts and rates per 100,000 for all injuries in Durango in 1990 and 2017

Cause	Deaths				Years of life lost			
	All-age counts (95% UI)		Age-standardised rates per 100,000 (95% UI)		All-age counts (95% UI)		Age-standardised rates per 100,000 (95% UI)	
	1990	2017	1990	2017	1990	2017	1990	2017
**Transport and unintentional injuries**	**950 (898 to 1 011)**	**703 (656 to 748)**	**81.0 (77.9 to 84.3)**	**40.0 (37.4 to 42.6)**	**53 156 (48 826 to 58 026)**	**31 304 (28 855 to 33 782)**	**3 698.5 (3 472.5 to 3 959.1)**	**1 726.2 (1 592.2 to 1 862.8)**
**Transport injuries**	**397 (372 to 425)**	**366 (336 to 396)**	**32.4 (30.7 to 34.2)**	**20.5 (18.8 to 22.3)**	**22 729 (20 885 to 24 882)**	**17 732 (16 162 to 19 489)**	**1 600.6 (1 495.8 to 1 719.7)**	**967.4 (882.5 to 1 061.5)**
Road injuries	384 (360 to 412)	352 (322 to 382)	31.4 (29.8 to 33.1)	19.6 (18.0 to 21.4)	22 043 (20 238 to 24 106)	17 023 (15 524 to 18 671)	1 551.5 (1 448.0 to 1 666.7)	928.7 (847.3 to 1 016.5)
Pedestrian road injuries	170 (155 to 187)	134 (123 to 146)	14.6 (13.6 to 15.6)	7.7 (7.1 to 8.4)	9 507 (8 875 to 10 857)	5 746 (5 216 to 6 281)	659.1 (596.1 to 731.9)	318.9 (289.9 to 348.1)
Cyclist road injuries	11 (10 to 12)	15 (13 to 17)	0.9 (0.8 to 1.0)	0.9 (0.8 to 1.0)	610 (558 to 691)	683 (604 to 779)	44.3 (39.7 to 49.6)	37.7 (33.4 to 43.0)
Motorcyclist road injuries	61 (55 to 68)	46 (41 to 53)	4.7 (4.2 to 5.1)	2.5 (2.2 to 2.8)	3 697 (3 264 to 4 105)	2 575 (2 251 to 2 957)	257.6 (233.1 to 285.9)	137.8 (120.9 to 157.4)
Motor vehicle road injuries	130 (120 to 140)	145 (129 to 166)	10.2 (9.5 to 11.0)	8.0 (7.1 to 9.1)	7 566 (6 902 to 8 271)	7 519 (6 676 to 8 674)	539.9 (496.6 to 584.2)	407.0 (362.0 to 469.0)
Other road injuries	13 (11 to 15)	10 (9 to 12)	1.0 (0.9 to 1.2)	0.6 (0.5 to 0.7)	724 (613 to 842)	500 (424 to 585)	50.5 (43.4 to 58.3)	27.8 (23.2 to 31.8)
Other transport injuries	12 (11 to 14)	15 (13 to 17)	1.0 (1.0 to 1.1)	0.8 (0.7 to 0.9)	685 (608 to 770)	708 (623 to 805)	49.1 (44.4 to 54.4)	38.7 (34.1 to 44.1)
**Unintentional injuries**	**553 (520 to 593)**	**336 (314 to 359)**	**48.6 (46.6 to 50.8)**	**19.5 (18.2 to 20.8)**	**30 428 (27 746 to 33 668)**	**13 572 (12 410 to 14 856)**	**2 097.9 (1 954.9 to 2 269.4)**	**758.8 (694.7 to 830.6)**
Falls	102 (97 to 107)	101 (94 to 109)	12.5 (11.8 to 13.1)	6.1 (5.6 to 6.6)	3 357 (3 132 to 3 628)	2 376 (2 177 to 2 562)	309.8 (288.1 to 321.1)	138.0 (126.7 to 148.7)
Drowning	108 (97 to 121)	45 (41 to 50)	7.8 (7.2 to 8.5)	2.5 (2.3 to 2.8)	6 926 (6 057 to 7 952)	2 428 (2 169 to 2 716)	438.6 (391.9 to 492.7)	132.8 (119.0 to 148.7)
Fire, heat, and hot substances	40 (36 to 45)	21 (19 to 22)	3.6 (3.4 to 3.9)	1.2 (1.1 to 1.3)	2 162 (1 842 to 2 555)	699 (632 to 777)	144.9 (127.9 to 166.2)	39.6 (35.8 to 43.9)
Poisonings	25 (22 to 28)	12 (11 to 13)	1.9 (1.7 to 2.0)	0.7 (0.6 to 0.8)	1 606 (1 391 to 1 855)	604 (546 to 673)	101.9 (90.4 to 115.1)	33.3 (30.1 to 37.1)
Poisoning by carbon monoxide	10 (9 to 12)	6 (5 to 6)	0.8 (0.7 to 0.9)	0.3 (0.3 to 0.4)	642 (552 to 751)	282 (252 to 317)	42.0 (36.9 to 48.1)	15.5 (13.9 to 17.4)
Poisoning by other means	15 (13 to 17)	6 (6 to 7)	1.1 (1.0 to 1.2)	0.4 (0.3 to 0.4)	964 (819 to 1 135)	322 (286 to 364)	60.0 (52.3 to 68.6)	17.8 (15.8 to 20.1)
Exposure to mechanical forces	127 (120 to 135)	51 (46 to 56)	10.7 (10.1 to 11.3)	2.9 (2.6 to 3.1)	7 050 (6 577 to 7 567)	2 283 (2 044 to 2 526)	519.7 (488.8 to 551.7)	125.7 (112.6 to 139.0)
Unintentional firearm injuries	83 (77 to 92)	26 (22 to 29)	6.8 (6.3 to 7.6)	1.4 (1.3 to 1.6)	4 699 (4 342 to 5 187)	1 246 (1 079 to 1 407)	344.9 (318.8 to 382.1)	68.0 (59.0 to 76.7)
Other exposure to mechanical forces	44 (34 to 49)	25 (22 to 28)	4.0 (3.1 to 4.3)	1.4 (1.3 to 1.6)	2 351 (1 808 to 2 661)	1 037 (914 to 1 170)	174.8 (132.4 to 194.6)	57.7 (50.9 to 65.4)
Adverse effects of medical treatment	24 (20 to 27)	25 (22 to 31)	2.5 (2.1 to 2.7)	1.5 (1.3 to 1.8)	1 128 (874 to 1 312)	788 (688 to 1 043)	81.3 (64.8 to 91.4)	45.2 (39.5 to 59.5)
Animal contact	16 (13 to 19)	6 (5 to 6)	1.1 (1.0 to 1.3)	0.3 (0.3 to 0.4)	1 074 (875 to 1 338)	243 (211 to 279)	63.0 (52.7 to 77.0)	13.7 (11.9 to 15.7)
Venomous animal contact	12 (10 to 15)	4 (3 to 4)	0.9 (0.7 to 1.0)	0.2 (0.2 to 0.2)	888 (699 to 1 121)	162 (132 to 194)	50.7 (40.8 to 62.9)	9.2 (7.5 to 11.0)
Non-venomous animal contact	3 (3 to 4)	2 (2 to 3)	0.3 (0.2 to 0.3)	0.1 (0.1 to 0.2)	186 (151 to 243)	80 (68 to 100)	12.3 (10.3 to 15.7)	4.5 (3.8 to 6.2)
Foreign body	77 (68 to 88)	59 (52 to 67)	5.5 (5.1 to 6.1)	3.3 (3.0 to 3.8)	5 265 (4 501 to 6 213)	3 354 (2 848 to 3 990)	312.2 (272.8 to 359.8)	186.6 (158.5 to 221.9)
Pulmonary aspiration and foreign body in airway	73 (65 to 84)	57 (51 to 65)	5.3 (4.8 to 5.8)	3.3 (2.9 to 3.7)	5 007 (4 260 to 5 911)	3 265 (2 762 to 3 908)	296.0 (257.9 to 341.6)	181.7 (153.8 to 217.1)
Foreign body in eyes								
Foreign body in other body part	4 (3 to 5)	2 (1 to 2)	0.3 (0.2 to 0.3)	0.1 (0.1 to 0.1)	258 (170 to 318)	89 (74 to 113)	16.2 (10.9 to 19.4)	4.9 (4.0 to 6.2)
Environmental heat and cold exposure	11 (10 to 12)	4 (4 to 5)	1.1 (1.0 to 1.2)	0.3 (0.2 to 0.3)	500 (450 to 558)	162 (146 to 178)	39.4 (36.0 to 42.8)	9.2 (8.2 to 10.1)
Exposure to forces of nature	2 (1 to 2)	0 (0 to 0)	0.1 (0.1 to 0.1)	0.0 (0.0 to 0.0)	102 (75 to 144)	15 (13 to 16)	5.6 (4.2 to 7.6)	0.8 (0.7 to 0.9)
Other unintentional injuries	22 (20 to 23)	12 (11 to 14)	1.7 (1.6 to 1.8)	0.7 (0.6 to 0.8)	1 256 (1 148 to 1 376)	626 (554 to 701)	87.5 (80.1 to 94.9)	34.0 (30.1 to 38.0)

Cause	Incidence				Prevalence			
	All-age counts (95% UI)		Age-standardised rates per 100,000 (95% UI)		All-age counts (95% UI)		Age-standardised rates per 100,000 (95% UI)	
	1990	2017	1990	2017	1990	2017	1990	2017
**Transport and unintentional injuries**	**61 513 (56 519 to 67 093)**	**82 654 (75 532 to 90 874)**	**4 998.8 (4 584.0 to 5 433.9)**	**4 723.6 (4 317.3 to 5 181.5)**	**118 373 (111 174 to 127 377)**	**171 475 (160 977 to 185 598)**	**11 613.6 (10 934.5 to 12 490.3)**	**9 885.8 (9 281.6 to 10 722.3)**
**Transport injuries**	**4 932 (4 254 to 5 717)**	**9 850 (8 546 to 11 245)**	**387.6 (340.4 to 441.0)**	**546.3 (472.9 to 622.9)**	**12 010 (11 188 to 12 773)**	**28 073 (26 001 to 30 354)**	**1 208.8 (1 126.4 to 1 286.1)**	**1 611.5 (1 493.6 to 1 742.7)**
Road injuries	4 668 (4 007 to 5 470)	9 053 (8 345 to 11 053)	368.1 (320.6 to 422.3)	535.2 (462.9 to 612.9)	10 819 (10 062 to 11 581)	27 075 (25 032 to 29 308)	1 099.0 (1 021.4 to 1 176.0)	1 554.2 (1 438.4 to 1 682.3)
Pedestrian road injuries	702 (564 to 872)	1 086 (879 to 1 307)	56.8 (46.8 to 68.3)	60.9 (49.3 to 73.3)	2 612 (2 226 to 3 068)	5 151 (4 459 to 6 004)	264.2 (228.8 to 310.4)	297.6 (258.2 to 346.8)
Cyclist road injuries	708 (543 to 917)	1 213 (970 to 1 504)	51.3 (40.9 to 63.6)	67.8 (54.2 to 83.7)	1 192 (996 to 1 406)	2 760 (2 354 to 3 232)	114.6 (97.1 to 133.2)	157.6 (134.6 to 183.5)
Motorcyclist road injuries	617 (485 to 779)	748 (569 to 945)	47.0 (37.8 to 58.0)	40.9 (31.2 to 51.9)	2 240 (1 944 to 2 577)	3 482 (3 005 to 4 074)	225.5 (194.8 to 259.9)	198.5 (171.5 to 232.2)
Motor vehicle road injuries	1 727 (1 420 to 2 057)	4 818 (4 037 to 5 697)	140.8 (117.7 to 164.4)	265.8 (229.0 to 313.7)	3 106 (2 729 to 3 574)	11 270 (9 831 to 13 190)	326.7 (284.1 to 376.6)	647.6 (564.1 to 757.2)
Other road injuries	914 (732 to 1 132)	1 789 (1 418 to 2 208)	72.2 (58.1 to 86.1)	99.8 (79.0 to 122.3)	1 669 (1 388 to 2 014)	4 412 (3 665 to 5 317)	168.0 (141.0 to 201.0)	252.9 (210.2 to 304.7)
Other transport injuries	264 (197 to 360)	196 (150 to 257)	19.5 (15.4 to 24.6)	11.1 (8.5 to 14.4)	1 191 (987 to 1 420)	998 (856 to 1 145)	109.7 (92.4 to 127.3)	57.3 (49.3 to 65.6)
**Unintentional injuries**	**56 581 (51 866 to 62 265)**	**72 804 (65 891 to 80 678)**	**4 611.3 (4 194.1 to 5 050.7)**	**4 177.3 (3 777.8 to 4 622.9)**	**106 364 (99 042 to 115 052)**	**143 402 (132 692 to 157 573)**	**10 404.8 (9 713.1 to 11 250.5)**	**8 274.3 (7 655.5 to 9 086.1)**
Falls	23 883 (21 035 to 27 220)	43 786 (38 937 to 49 284)	2 117.3 (1 890.5 to 2 384.9)	2 545.1 (2 258.5 to 2 858.6)	41 221 (36 369 to 47 173)	85 518 (76 264 to 97 902)	4 234.6 (3 784.1 to 4 826.1)	4 973.2 (4 440.9 to 5 693.4)
Drowning	87 (75 to 101)	41 (33 to 52)	6.3 (5.5 to 7.3)	2.3 (1.8 to 2.9)	393 (344 to 455)	253 (222 to 295)	39.4 (34.8 to 45.1)	14.7 (12.9 to 17.0)
Fire, heat, and hot substances	2 037 (1 614 to 2 541)	1 280 (1 038 to 1 578)	124.3 (101.0 to 151.0)	71.7 (58.1 to 88.5)	19 312 (15 878 to 24 348)	14 868 (12 242 to 18 872)	1 641.3 (1 385.8 to 2 032.0)	839.5 (692.9 to 1 060.6)
Poisonings	695 (517 to 908)	572 (419 to 766)	38.7 (29.8 to 49.7)	31.9 (23.3 to 43.0)	595 (458 to 791)	656 (485 to 824)	45.4 (35.4 to 55.4)	36.4 (26.9 to 45.7)
Poisoning by carbon monoxide	136 (91 to 206)	92 (57 to 142)	8.4 (6.0 to 11.8)	5.1 (3.2 to 7.9)	92 (71 to 121)	67 (52 to 86)	7.3 (5.6 to 9.4)	3.8 (2.9 to 4.8)
Poisoning by other means	559 (410 to 745)	480 (344 to 659)	30.3 (22.7 to 39.8)	26.8 (19.1 to 37.0)	503 (368 to 628)	588 (420 to 759)	38.1 (28.1 to 47.4)	32.7 (23.3 to 41.8)
Exposure to mechanical forces	16 452 (13 959 to 19 264)	11 829 (10 127 to 13 761)	1 227.8 (1 056.1 to 1 428.6)	653.3 (560.7 to 760.3)	32 306 (28 457 to 36 914)	28 745 (25 303 to 32 964)	3 242.6 (2 863.6 to 3 691.5)	1 644.6 (1 447.5 to 1 884.0)
Unintentional firearm injuries	1 742 (1 299 to 2 274)	851 (633 to 1 096)	130.8 (98.8 to 167.5)	46.7 (34.6 to 59.8)	5 912 (4 708 to 7 128)	3 898 (3 154 to 4 785)	612.8 (491.3 to 743.8)	223.9 (181.1 to 275.2)
Other exposure to mechanical forces	14 710 (12 490 to 17 276)	10 972 (9 417 to 12 800)	1 097.0 (943.6 to 1 282.1)	606.6 (523.3 to 707.4)	26 394 (22 384 to 31 182)	24 847 (21 291 to 29 146)	2 629.8 (2 243.1 to 3 102.0)	1 420.7 (1 219.5 to 1 665.3)
Adverse effects of medical treatment	1 034 (870 to 1 209)	1 852 (1 572 to 2 142)	93.2 (79.6 to 106.7)	106.4 (90.8 to 123.0)	79 (61 to 99)	142 (109 to 175)	7.1 (5.4 to 8.8)	8.1 (6.3 to 10.1)
Animal contact	3 910 (3 228 to 4 736)	3 241 (2 817 to 3 758)	288.6 (245.9 to 336.9)	183.1 (159.3 to 211.9)	4 070 (3 472 to 4 739)	4 086 (3 545 to 4 700)	380.6 (329.7 to 434.5)	233.4 (202.7 to 267.7)
Venomous animal contact	1 410 (1 064 to 1 845)	1 112 (877 to 1 392)	103.6 (79.8 to 130.8)	62.6 (49.5 to 78.4)	939 (768 to 1 135)	837 (702 to 982)	81.9 (68.3 to 97.0)	47.4 (39.8 to 55.4)
Non-venomous animal contact	2 499 (1 996 to 3 103)	2 130 (1 784 to 2 552)	185.0 (153.3 to 223.9)	120.5 (101.2 to 144.3)	3 131 (2 566 to 3 776)	3 249 (2 735 to 3 855)	298.7 (249.4 to 351.0)	186.0 (157.3 to 219.8)
Foreign body	5 103 (2 657 to 8 347)	7 423 (3 229 to 12 797)	462.7 (205.4 to 787.7)	426.7 (179.8 to 728.1)	2 220 (1 709 to 2 981)	2 643 (1 908 to 3 744)	208.9 (161.0 to 278.1)	153.0 (110.5 to 215.4)
Pulmonary aspiration and foreign body in airway	188 (129 to 262)	78 (57 to 103)	10.9 (7.9 to 14.9)	4.4 (3.2 to 5.8)	128 (90 to 175)	68 (55 to 84)	10.0 (7.2 to 13.4)	3.8 (3.1 to 4.7)
Foreign body in eyes	4 535 (2 079 to 7 815)	7 078 (2 800 to 12 364)	426.6 (166.3 to 748.7)	407.2 (156.2 to 712.2)	922 (483 to 1 611)	1 578 (874 to 2 656)	97.8 (55.4 to 159.5)	92.4 (51.8 to 153.0)
Foreign body in other body part	379 (266 to 555)	267 (200 to 348)	25.1 (18.2 to 34.6)	15.1 (11.3 to 19.7)	1 169 (939 to 1 489)	1 009 (824 to 1 248)	101.2 (82.7 to 125.8)	56.8 (46.8 to 70.8)
Environmental heat and cold exposure	767 (558 to 1 018)	385 (271 to 561)	59.4 (46.0 to 73.1)	22.1 (15.6 to 32.4)	2 600 (2 130 to 3 137)	1 522 (1 267 to 1 844)	243.6 (205.6 to 287.6)	87.5 (73.0 to 106.1)
Exposure to forces of nature	225 (172 to 296)	20 (17 to 23)	12.4 (9.8 to 15.8)	1.1 (0.9 to 1.3)	377 (278 to 511)	946 (668 to 1 305)	32.3 (24.0 to 43.8)	52.8 (37.4 to 72.8)
Other unintentional injuries	2 389 (1 980 to 2 896)	2 381 (2 042 to 2 778)	180.7 (153.4 to 211.0)	133.5 (114.1 to 155.4)	3 391 (2 934 to 3 890)	4 044 (3 547 to 4 585)	329.1 (286.4 to 374.5)	231.7 (203.2 to 262.1)

Cause	Years lived with disability				Disability adjusted life years			
	All-age counts (95% UI)		Age-standardised rates per 100,000 (95% UI)		All-age counts (95% UI)		Age-standardised rates per 100,000 (95% UI)	
	1990	2017	1990	2017	1990	2017	1990	2017
**Transport and unintentional injuries**	**5 074 (3 708 to 6 667)**	**7 663 (5 572 to 10 271)**	**490.0 (357.7 to 648.2)**	**441.5 (320.7 to 592.4)**	**58 230 (53 873 to 63 263)**	**38 967 (35 853 to 42 592)**	**4 188.5 (3 929.2 to 4 486.1)**	**2 167.7 (1 994.5 to 2 371.0)**
**Transport injuries**	**822 (600 to 1 090)**	**1 722 (1 249 to 2 293)**	**79.9 (58.4 to 105.9)**	**98.3 (71.4 to 130.8)**	**23 551 (21 682 to 25 632)**	**19 454 (17 897 to 21 200)**	**1 680.5 (1 571.9 to 1 802.9)**	**1 065.7 (981.5 to 1 160.7)**
Road injuries	745 (544 to 988)	1 669 (1 212 to 2 226)	73.2 (53.7 to 97.0)	95.2 (69.2 to 126.8)	22 788 (20 965 to 24 879)	18 692 (17 188 to 20 384)	1 624.6 (1 517.9 to 1 743.0)	1 023.9 (943.0 to 1 116.3)
Pedestrian road injuries	189 (134 to 256)	331 (235 to 450)	18.3 (13.1 to 24.8)	19.0 (13.5 to 25.9)	9 695 (8 557 to 11 058)	6 078 (5 567 to 6 649)	677.4 (614.1 to 748.9)	338.0 (310.0 to 369.0)
Cyclist road injuries	83 (59 to 110)	169 (119 to 224)	7.8 (5.5 to 10.2)	9.6 (6.8 to 12.7)	693 (618 to 780)	852 (758 to 958)	52.1 (46.8 to 58.1)	47.3 (42.1 to 53.1)
Motorcyclist road injuries	156 (109 to 212)	217 (149 to 299)	15.2 (10.6 to 20.7)	12.3 (8.4 to 16.9)	3 793 (3 407 to 4 264)	2 792 (2 450 to 3 164)	272.9 (247.8 to 302.8)	150.1 (132.0 to 170.1)
Motor vehicle road injuries	222 (161 to 295)	729 (520 to 973)	22.5 (16.4 to 29.8)	41.3 (29.7 to 55.3)	7 788 (7 118 to 8 511)	8 241 (7 378 to 9 467)	562.5 (519.8 to 609.4)	448.2 (401.7 to 513.5)
Other road injuries	95 (69 to 128)	228 (163 to 317)	9.3 (6.8 to 12.5)	13.0 (9.3 to 18.1)	819 (700 to 948)	729 (633 to 851)	59.8 (51.7 to 67.7)	40.8 (35.1 to 46.9)
Other transport injuries	77 (54 to 103)	54 (39 to 72)	6.8 (4.8 to 8.9)	3.1 (2.2 to 4.1)	762 (681 to 851)	762 (679 to 863)	55.9 (50.6 to 61.4)	41.8 (37.4 to 47.3)
**Unintentional injuries**	**4 252 (3 081 to 5 619)**	**5 941 (4 272 to 8 025)**	**410.1 (297.3 to 547.9)**	**343.2 (246.9 to 461.5)**	**34 680 (31 645 to 38 214)**	**19 513 (17 413 to 21 844)**	**2 508.0 (2 317.3 to 2 725.7)**	**1 102.0 (980.8 to 1 233.8)**
Falls	2 467 (1 804 to 3 227)	4 430 (3 206 to 5 876)	246.3 (179.3 to 321.4)	257.2 (185.8 to 342.0)	5 824 (5 070 to 6 627)	6 806 (5 544 to 8 201)	550.1 (478.8 to 625.2)	395.2 (322.2 to 476.6)
Drowning	22 (16 to 31)	13 (9 to 19)	2.2 (1.5 to 3.0)	0.8 (0.5 to 1.1)	6 949 (6 081 to 7 974)	2 436 (2 180 to 2 727)	440.8 (394.3 to 494.8)	133.6 (119.8 to 149.5)
Fire, heat, and hot substances	414 (256 to 646)	290 (174 to 476)	34.4 (21.2 to 54.1)	16.3 (9.8 to 26.7)	2 576 (2 200 to 3 026)	988 (846 to 1 182)	179.3 (157.0 to 206.9)	55.9 (47.8 to 66.9)
Poisonings	71 (44 to 106)	76 (46 to 116)	5.2 (3.3 to 7.7)	4.2 (2.5 to 6.4)	1 676 (1 463 to 1 931)	680 (613 to 751)	107.1 (95.4 to 120.8)	37.5 (33.8 to 41.5)
Poisoning by carbon monoxide	10 (6 to 16)	7 (4 to 10)	0.7 (0.5 to 1.2)	0.4 (0.2 to 0.6)	652 (562 to 763)	289 (258 to 323)	42.7 (37.6 to 48.9)	15.9 (14.2 to 17.8)
Poisoning by other means	61 (36 to 92)	69 (41 to 106)	4.5 (2.7 to 6.7)	3.8 (2.3 to 5.9)	1 024 (879 to 1 197)	391 (346 to 446)	64.4 (56.5 to 73.4)	21.6 (19.2 to 24.7)
Exposure to mechanical forces	761 (524 to 1 090)	630 (426 to 917)	74.8 (51.7 to 108.1)	36.0 (24.3 to 52.3)	7 811 (7 280 to 8 446)	2 913 (2 601 to 3 278)	594.5 (556.2 to 640.0)	161.7 (144.9 to 181.9)
Unintentional firearm injuries	127 (84 to 192)	76 (50 to 118)	12.8 (8.4 to 19.5)	4.3 (2.8 to 6.8)	4 827 (4 465 to 5 306)	1 322 (1 150 to 1 482)	357.7 (331.3 to 394.5)	72.3 (63.0 to 81.0)
Other exposure to mechanical forces	633 (435 to 904)	554 (375 to 806)	62.0 (42.5 to 89.7)	31.6 (21.4 to 46.1)	2 984 (2 409 to 3 395)	1 592 (1 363 to 1 881)	236.8 (195.1 to 271.1)	89.3 (76.4 to 105.8)
Adverse effects of medical treatment	11 (6 to 16)	19 (12 to 29)	1.0 (0.6 to 1.4)	1.1 (0.7 to 1.6)	1 139 (883 to 1 323)	807 (709 to 1 060)	82.3 (65.7 to 92.3)	46.3 (40.6 to 60.4)
Animal contact	110 (73 to 155)	96 (63 to 136)	9.3 (6.2 to 13.2)	5.4 (3.6 to 7.7)	1 184 (980 to 1 450)	338 (292 to 392)	72.3 (61.4 to 86.4)	19.1 (16.5 to 22.1)
Venomous animal contact	61 (39 to 90)	50 (32 to 71)	4.7 (3.1 to 6.9)	2.8 (1.8 to 4.0)	950 (750 to 1 192)	212 (175 to 250)	55.5 (45.1 to 68.2)	11.9 (9.9 to 14.0)
Non-venomous animal contact	49 (29 to 77)	46 (27 to 74)	4.6 (2.8 to 7.2)	2.6 (1.6 to 4.2)	234 (189 to 301)	127 (103 to 161)	16.8 (13.7 to 21.3)	7.1 (5.8 to 9.1)
Foreign body	140 (91 to 211)	156 (95 to 251)	12.9 (8.5 to 19.3)	9.0 (5.5 to 14.4)	5 404 (4 690 to 6 338)	3 510 (2 987 to 4 189)	325.1 (285.2 to 372.0)	195.6 (166.5 to 233.0)
Pulmonary aspiration and foreign body in airway	7 (5 to 10)	3 (2 to 4)	0.5 (0.3 to 0.7)	0.2 (0.1 to 0.2)	5 014 (4 266 to 5 918)	3 268 (2 765 to 3 911)	296.5 (258.6 to 342.3)	181.9 (154.0 to 217.3)
Foreign body in eyes	61 (31 to 111)	105 (55 to 187)	6.5 (3.5 to 11.6)	6.1 (3.3 to 11.0)	61 (31 to 111)	105 (55 to 187)	6.5 (3.5 to 11.6)	6.1 (3.3 to 11.0)
Foreign body in other body part	71 (50 to 96)	48 (34 to 64)	5.9 (4.2 to 7.7)	2.7 (1.9 to 3.6)	329 (244 to 390)	137 (114 to 168)	22.1 (16.5 to 25.7)	7.6 (6.3 to 9.3)
Environmental heat and cold exposure	103 (69 to 146)	58 (37 to 84)	9.6 (6.5 to 13.4)	3.3 (2.1 to 4.9)	604 (537 to 675)	220 (194 to 251)	49.0 (44.3 to 54.3)	12.5 (11.0 to 14.3)
Exposure to forces of nature	21 (15 to 30)	34 (25 to 46)	1.8 (1.3 to 2.5)	1.9 (1.4 to 2.5)	124 (95 to 166)	49 (39 to 61)	7.4 (5.9 to 9.6)	2.7 (2.2 to 3.4)
Other unintentional injuries	133 (95 to 177)	139 (98 to 190)	12.6 (9.0 to 16.9)	8.0 (5.6 to 10.9)	1 389 (1 265 to 1 517)	765 (681 to 858)	100.1 (91.7 to 108.3)	41.9 (37.4 to 47.0)

Death, YLL, incidence, prevalence, YLD, and DALY counts and rates per 100,000 for all injuries in Guanajuato in 1990 and 2017

Cause	Deaths: All-age counts (95% UI) 1990	Deaths: All-age counts (95% UI) 2017	Deaths: Age-standardised rates per 100,000 (95% UI) 1990	Deaths: Age-standardised rates per 100,000 (95% UI) 2017	Years of life lost: All-age counts (95% UI) 1990	Years of life lost: All-age counts (95% UI) 2017	Years of life lost: Age-standardised rates per 100,000 (95% UI) 1990	Years of life lost: Age-standardised rates per 100,000 (95% UI) 2017
**Transport and unintentional injuries**	**2 269 (2 166 to 2 371)**	**2 064 (1 922 to 2 211)**	**72.0 (70.0 to 74.1)**	**35.1 (32.8 to 37.4)**	**123 988 (115 973 to 132 158)**	**91 864 (84 869 to 99 211)**	**3 023.0 (2 874.9 to 3 165.5)**	**1 454.9 (1 346.1 to 1 570.2)**
**Transport injuries**	**1 201 (1 140 to 1 259)**	**1 167 (1 071 to 1 268)**	**35.9 (34.5 to 37.3)**	**19.0 (17.5 to 20.6)**	**66 571 (62 378 to 70 705)**	**56 908 (51 852 to 61 895)**	**1 649.7 (1 565.5 to 1 731.9)**	**885.7 (808.7 to 963.0)**
Road injuries	1 163 (1 104 to 1 220)	1 107 (1 017 to 1 202)	34.7 (33.3 to 36.2)	18.1 (16.6 to 19.6)	64 493 (60 454 to 68 680)	54 009 (49 218 to 58 746)	1 597.7 (1 512.8 to 1 678.0)	840.7 (768.4 to 914.2)
Pedestrian road injuries	646 (604 to 688)	553 (507 to 604)	20.6 (19.6 to 21.6)	9.3 (8.6 to 10.2)	34 457 (31 556 to 37 551)	24 481 (22 242 to 26 832)	857.9 (800.0 to 916.8)	388.5 (354.5 to 425.5)
Cyclist road injuries	27 (25 to 30)	43 (38 to 49)	0.8 (0.7 to 0.9)	0.7 (0.6 to 0.8)	1 545 (1 360 to 1 718)	1 985 (1 740 to 2 260)	39.1 (35.2 to 43.0)	31.3 (27.6 to 35.6)
Motorcyclist road injuries	133 (123 to 147)	118 (102 to 137)	3.5 (3.3 to 3.9)	1.8 (1.6 to 2.1)	7 919 (7 124 to 8 793)	6 828 (5 851 to 7 904)	193.3 (176.1 to 212.4)	102.6 (88.0 to 118.5)
Motor vehicle road injuries	316 (290 to 344)	354 (315 to 392)	8.7 (8.0 to 9.4)	5.6 (5.0 to 6.2)	18 297 (16 659 to 20 030)	18 839 (16 776 to 21 067)	452.1 (414.7 to 493.2)	288.4 (257.5 to 322.4)
Other road injuries	40 (34 to 47)	39 (34 to 46)	1.2 (1.0 to 1.3)	0.6 (0.6 to 0.7)	2 274 (1 925 to 2 676)	1 925 (1 645 to 2 270)	55.2 (47.0 to 64.0)	29.8 (25.6 to 35.1)
Other transport injuries	38 (34 to 41)	60 (50 to 68)	1.2 (1.1 to 1.3)	1.0 (0.8 to 1.1)	2 078 (1 866 to 2 306)	2 899 (2 422 to 3 339)	52.0 (47.2 to 57.2)	45.0 (37.6 to 51.5)
**Unintentional injuries**	**1 069 (1 013 to 1 131)**	**897 (841 to 955)**	**36.1 (35.0 to 37.3)**	**16.1 (15.1 to 17.1)**	**57 417 (52 841 to 62 743)**	**34 946 (32 213 to 38 082)**	**1 373.3 (1 293.8 to 1 462.0)**	**569.2 (521.8 to 620.0)**
Falls	234 (224 to 246)	305 (279 to 329)	11.8 (11.3 to 12.4)	5.9 (5.4 to 6.4)	7 651 (7 155 to 8 193)	7 299 (6 495 to 7 866)	252.6 (240.9 to 265.0)	125.6 (113.2 to 136.4)
Drowning	272 (251 to 297)	152 (138 to 166)	6.9 (6.4 to 7.3)	2.5 (2.3 to 2.7)	17 302 (15 625 to 19 331)	8 171 (7 358 to 9 085)	372.5 (343.3 to 407.3)	127.9 (115.2 to 141.3)
Fire, heat, and hot substances	83 (76 to 91)	59 (53 to 63)	3.0 (2.9 to 3.2)	1.1 (1.0 to 1.2)	4 380 (3 805 to 5 023)	1 907 (1 698 to 2 074)	103.0 (93.1 to 114.0)	31.7 (28.4 to 34.4)
Poisonings	45 (41 to 50)	28 (25 to 31)	1.2 (1.1 to 1.3)	0.5 (0.4 to 0.5)	2 874 (2 541 to 3 264)	1 359 (1 192 to 1 505)	61.9 (56.2 to 68.6)	21.6 (19.0 to 23.9)
Poisoning by carbon monoxide	17 (16 to 19)	13 (11 to 14)	0.5 (0.4 to 0.5)	0.2 (0.2 to 0.2)	1 077 (954 to 1 220)	601 (529 to 676)	23.9 (21.6 to 26.6)	9.5 (8.3 to 10.6)
Poisoning by other means	27 (25 to 31)	16 (14 to 17)	0.7 (0.7 to 0.8)	0.3 (0.2 to 0.3)	1 798 (1 568 to 2 066)	757 (661 to 848)	38.0 (33.9 to 42.6)	12.1 (10.6 to 13.5)
Exposure to mechanical forces	145 (136 to 154)	80 (74 to 87)	4.3 (4.1 to 4.5)	1.3 (1.2 to 1.5)	8 112 (7 549 to 8 707)	3 529 (3 218 to 3 880)	201.0 (189.0 to 213.4)	55.8 (51.1 to 61.1)
Unintentional firearm injuries	69 (63 to 76)	26 (19 to 29)	1.9 (1.7 to 2.1)	0.4 (0.3 to 0.5)	4 049 (3 617 to 4 449)	1 232 (890 to 1 424)	97.7 (89.0 to 107.3)	19.2 (13.9 to 22.1)
Other exposure to mechanical forces	75 (69 to 83)	54 (49 to 61)	2.4 (2.2 to 2.6)	0.9 (0.8 to 1.0)	4 063 (3 677 to 4 488)	2 297 (2 057 to 2 648)	103.2 (94.8 to 113.7)	36.7 (33.0 to 42.0)
Adverse effects of medical treatment	51 (47 to 61)	65 (58 to 89)	2.2 (2.0 to 2.5)	1.2 (1.1 to 1.7)	2 252 (1 997 to 2 776)	1 920 (1 650 to 2 876)	59.0 (53.9 to 71.8)	32.9 (28.3 to 49.1)
Animal contact	35 (30 to 40)	17 (15 to 18)	0.9 (0.8 to 1.0)	0.3 (0.3 to 0.3)	2 336 (1 925 to 2 822)	685 (611 to 768)	46.5 (39.6 to 54.7)	11.3 (10.1 to 12.6)
Venomous animal contact	26 (22 to 31)	10 (8 to 11)	0.6 (0.6 to 0.7)	0.2 (0.1 to 0.2)	1 854 (1 477 to 2 309)	414 (354 to 484)	35.4 (29.1 to 42.9)	6.9 (5.9 to 8.0)
Non-venomous animal contact	9 (7 to 10)	7 (6 to 8)	0.3 (0.2 to 0.3)	0.1 (0.1 to 0.1)	482 (398 to 572)	271 (233 to 332)	11.0 (9.3 to 12.7)	4.4 (3.8 to 5.5)
Foreign body	123 (111 to 136)	134 (120 to 149)	3.4 (3.1 to 3.6)	2.3 (2.1 to 2.5)	7 997 (6 978 to 9 113)	7 900 (6 276 to 8 549)	166.6 (149.4 to 185.5)	118.3 (101.7 to 138.7)
Pulmonary aspiration and foreign body in airway	117 (105 to 130)	130 (117 to 145)	3.2 (3.0 to 3.4)	2.2 (2.0 to 2.5)	7 589 (6 599 to 8 673)	7 127 (6 118 to 8 306)	157.8 (141.2 to 175.8)	115.6 (99.2 to 134.8)
Foreign body in eyes								
Foreign body in other body part	6 (5 to 7)	3 (2 to 5)	0.1 (0.1 to 0.2)	0.0 (0.0 to 0.1)	408 (306 to 488)	173 (139 to 300)	8.8 (6.7 to 10.2)	2.7 (2.2 to 4.7)
Environmental heat and cold exposure	18 (16 to 19)	10 (8 to 11)	0.7 (0.7 to 0.8)	0.2 (0.1 to 0.2)	821 (735 to 918)	346 (298 to 382)	22.5 (20.7 to 24.4)	5.7 (4.9 to 6.3)
Exposure to forces of nature	2 (2 to 2)	1 (1 to 1)	0.1 (0.0 to 0.1)	0.0 (0.0 to 0.0)	112 (102 to 125)	51 (47 to 56)	2.6 (2.3 to 2.8)	0.8 (0.7 to 0.9)
Other unintentional injuries	61 (57 to 66)	47 (42 to 53)	1.7 (1.6 to 1.8)	0.8 (0.7 to 0.8)	3 579 (3 304 to 3 888)	2 440 (2 177 to 2 766)	85.3 (79.1 to 92.1)	37.5 (33.6 to 42.4)

Cause	Incidence: All-age counts (95% UI) 1990	Incidence: All-age counts (95% UI) 2017	Incidence: Age-standardised rates per 100,000 (95% UI) 1990	Incidence: Age-standardised rates per 100,000 (95% UI) 2017	Prevalence: All-age counts (95% UI) 1990	Prevalence: All-age counts (95% UI) 2017	Prevalence: Age-standardised rates per 100,000 (95% UI) 1990	Prevalence: Age-standardised rates per 100,000 (95% UI) 2017
**Transport and unintentional injuries**	**149 817 (138 380 to 161 970)**	**265 903 (244 835 to 288 764)**	**4 224.2 (3 894.9 to 4 573.0)**	**4 469.4 (4 120.4 to 4 856.6)**	**281 732 (265 009 to 301 315)**	**561 239 (523 387 to 607 541)**	**9 811.3 (9 265.8 to 10 509.4)**	**9 595.9 (8 975.8 to 10 388.2)**
**Transport injuries**	**15 329 (13 132 to 17 850)**	**36 257 (31 315 to 41 551)**	**421.0 (366.6 to 479.4)**	**577.6 (499.3 to 657.7)**	**35 866 (33 487 to 38 213)**	**99 844 (92 886 to 107 047)**	**1 298.4 (1 214.8 to 1 387.3)**	**1 689.3 (1 570.2 to 1 813.6)**
Road injuries	14 583 (12 414 to 17 126)	35 662 (30 728 to 41 028)	402.1 (349.2 to 460.2)	567.7 (490.0 to 649.1)	32 616 (30 410 to 34 982)	96 872 (89 918 to 104 016)	1 192.4 (1 113.7 to 1 276.9)	1 638.8 (1 522.9 to 1 761.4)
Pedestrian road injuries	2 574 (2 093 to 3 167)	4 809 (3 929 to 5 749)	74.2 (61.6 to 88.0)	78.2 (64.1 to 93.4)	9 115 (7 814 to 10 733)	21 292 (18 501 to 24 795)	336.3 (292.6 to 389.5)	365.3 (318.3 to 422.8)
Cyclist road injuries	2 538 (1 955 to 3 253)	6 149 (4 899 to 7 641)	63.5 (50.7 to 78.7)	98.3 (78.6 to 121.3)	4 129 (3 451 to 4 899)	13 366 (11 297 to 15 661)	141.1 (119.4 to 164.6)	223.8 (190.4 to 260.7)
Motorcyclist road injuries	1 654 (1 304 to 2 118)	3 184 (2 442 to 4 107)	43.7 (34.9 to 54.3)	49.1 (37.9 to 62.9)	5 767 (4 989 to 6 667)	14 233 (12 127 to 16 803)	207.7 (179.7 to 239.8)	236.2 (201.9 to 277.9)
Motor vehicle road injuries	4 899 (3 989 to 5 898)	15 623 (12 957 to 18 558)	140.4 (116.1 to 165.6)	247.0 (206.0 to 291.9)	8 471 (7 421 to 9 879)	34 976 (29 930 to 40 344)	322.7 (280.4 to 374.9)	589.1 (507.6 to 681.5)
Other road injuries	2 917 (2 262 to 3 640)	5 897 (4 664 to 7 177)	80.3 (64.5 to 96.4)	95.0 (74.9 to 114.4)	5 134 (4 274 to 6 199)	13 604 (11 358 to 16 497)	184.7 (155.1 to 221.2)	230.3 (192.8 to 278.7)
Other transport injuries	746 (567 to 1 007)	596 (483 to 749)	18.9 (15.4 to 23.3)	9.8 (8.0 to 12.4)	3 249 (2 687 to 3 870)	2 973 (2 543 to 3 404)	106.0 (89.6 to 122.7)	50.5 (43.5 to 57.5)
**Unintentional injuries**	**134 488 (123 247 to 147 010)**	**229 646 (209 186 to 251 872)**	**3 803.2 (3 490.6 to 4 152.8)**	**3 891.8 (3 548.6 to 4 272.3)**	**245 866 (229 341 to 265 028)**	**461 394 (426 231 to 507 974)**	**8 512.9 (7 954.8 to 9 195.1)**	**7 906.6 (7 300.4 to 8 703.6)**
Falls	61 121 (53 324 to 69 937)	140 423 (123 720 to 158 944)	1 899.2 (1 685.4 to 2 128.3)	2 440.8 (2 157.0 to 2 748.9)	103 032 (91 007 to 117 235)	272 424 (241 973 to 311 969)	3 793.2 (3 399.7 to 4 327.5)	4 757.1 (4 248.5 to 5 457.3)
Drowning	237 (203 to 275)	184 (109 to 167)	5.9 (5.1 to 6.7)	2.2 (1.8 to 2.7)	1 024 (897 to 1 183)	798 (700 to 924)	36.7 (32.5 to 41.9)	13.7 (12.1 to 15.9)
Fire, heat, and hot substances	5 282 (4 355 to 6 697)	4 763 (3 817 to 5 965)	109.0 (88.5 to 133.4)	76.7 (61.7 to 95.3)	47 564 (39 563 to 60 224)	55 481 (45 979 to 71 366)	1 447.0 (1 220.5 to 1 771.4)	908.7 (745.6 to 1 158.6)
Poisonings	1 743 (1 289 to 2 375)	2 028 (1 468 to 2 756)	32.4 (24.7 to 43.0)	32.7 (23.6 to 44.2)	1 424 (1 079 to 1 768)	2 371 (1 764 to 2 986)	37.7 (29.1 to 46.6)	37.8 (28.2 to 47.6)
Poisoning by carbon monoxide	335 (211 to 513)	261 (158 to 404)	7.0 (4.7 to 10.0)	4.3 (2.6 to 6.5)	218 (165 to 284)	194 (149 to 249)	6.0 (4.6 to 7.7)	3.1 (2.4 to 4.0)
Poisoning by other means	1 408 (1 017 to 1 981)	1 768 (1 259 to 2 431)	25.5 (18.8 to 34.9)	28.4 (20.2 to 38.8)	1 205 (869 to 1 533)	2 177 (1 547 to 2 787)	31.7 (23.1 to 40.2)	34.7 (24.7 to 44.4)
Exposure to mechanical forces	31 135 (26 318 to 36 855)	36 621 (31 220 to 42 703)	788.1 (673.0 to 922.6)	580.7 (497.1 to 675.1)	58 531 (51 465 to 67 424)	87 687 (75 949 to 101 948)	2 070.0 (1 826.2 to 2 371.3)	1 470.4 (1 277.7 to 1 700.8)
Unintentional firearm injuries	2 433 (1 765 to 3 227)	1 519 (1 101 to 2 054)	59.8 (44.5 to 78.3)	23.9 (17.3 to 31.9)	8 449 (6 655 to 10 090)	7 464 (6 046 to 9 012)	303.7 (242.0 to 364.4)	126.3 (103.0 to 152.4)
Other exposure to mechanical forces	28 702 (24 207 to 33 945)	35 102 (29 887 to 40 958)	728.3 (623.6 to 855.0)	556.8 (474.0 to 646.4)	50 082 (42 393 to 59 062)	80 224 (68 339 to 94 630)	1 766.4 (1 507.0 to 2 077.8)	1 344.1 (1 148.0 to 1 578.2)
Adverse effects of medical treatment	3 082 (2 599 to 3 585)	6 790 (5 785 to 7 814)	96.2 (82.8 to 109.6)	114.2 (97.8 to 130.4)	236 (183 to 291)	520 (402 to 644)	7.4 (5.7 to 9.0)	8.7 (6.8 to 10.7)
Animal contact	10 597 (8 720 to 13 116)	10 481 (9 104 to 12 243)	269.4 (229.7 to 316.9)	173.4 (150.6 to 201.8)	10 689 (9 303 to 12 436)	12 868 (11 153 to 14 817)	354.7 (307.8 to 408.2)	217.3 (188.9 to 249.1)
Venomous animal contact	3 675 (2 792 to 4 850)	3 741 (2 977 to 4 647)	93.4 (71.8 to 117.7)	61.8 (49.1 to 76.6)	2 326 (1 897 to 2 772)	2 819 (2 338 to 3 329)	71.7 (60.0 to 83.4)	46.7 (38.9 to 54.9)
Non-venomous animal contact	6 922 (5 545 to 8 699)	6 740 (5 636 to 8 163)	176.0 (145.4 to 212.5)	111.6 (93.9 to 134.9)	8 363 (6 862 to 10 067)	10 049 (8 434 to 11 987)	283.0 (236.6 to 332.0)	170.3 (143.2 to 201.8)
Foreign body	11 717 (6 242 to 17 864)	18 317 (8 167 to 29 789)	353.6 (165.4 to 579.9)	307.7 (133.2 to 513.9)	5 586 (4 313 to 7 372)	7 241 (5 415 to 9 882)	177.9 (139.6 to 231.6)	123.3 (92.4 to 167.5)
Pulmonary aspiration and foreign body in airway	362 (250 to 518)	189 (135 to 257)	7.1 (5.2 to 9.7)	3.1 (2.2 to 4.2)	286 (169 to 318)	161 (129 to 199)	6.4 (4.8 to 8.5)	2.7 (2.1 to 3.3)
Foreign body in eyes	10 246 (4 923 to 16 333)	17 248 (7 047 to 28 842)	322.6 (133.3 to 551.0)	290.1 (116.0 to 495.6)	2 016 (1 033 to 3 469)	3 729 (2 037 to 6 059)	73.8 (41.3 to 118.5)	65.5 (36.7 to 104.6)
Foreign body in other body part	1 108 (742 to 1 668)	880 (624 to 1 184)	23.9 (16.6 to 33.8)	14.4 (10.2 to 19.3)	3 334 (2 626 to 4 304)	3 352 (2 719 to 4 259)	98.2 (79.1 to 123.0)	55.2 (45.1 to 69.9)
Environmental heat and cold exposure	1 966 (1 364 to 2 684)	1 158 (808 to 1 780)	50.5 (38.5 to 63.9)	19.6 (13.8 to 30.1)	6 658 (5 348 to 8 182)	4 685 (3 853 to 5 617)	215.1 (178.6 to 257.1)	78.9 (65.8 to 95.1)
Exposure to forces of nature	293 (240 to 367)	68 (58 to 82)	6.6 (5.5 to 7.9)	1.1 (0.9 to 1.3)	994 (672 to 1 275)	2 046 (1 441 to 2 823)	25.5 (18.6 to 34.9)	33.4 (23.4 to 46.0)
Other unintentional injuries	7 314 (6 062 to 8 923)	8 863 (7 538 to 10 430)	192.3 (163.6 to 224.3)	142.9 (121.7 to 167.8)	10 083 (8 668 to 11 634)	15 332 (13 263 to 17 560)	347.7 (301.3 to 397.3)	257.5 (223.2 to 294.1)

Cause	Years lived with disability: All-age counts (95% UI) 1990	Years lived with disability: All-age counts (95% UI) 2017	Years lived with disability: Age-standardised rates per 100,000 (95% UI) 1990	Years lived with disability: Age-standardised rates per 100,000 (95% UI) 2017	Disability adjusted life years: All-age counts (95% UI) 1990	Disability adjusted life years: All-age counts (95% UI) 2017	Disability adjusted life years: Age-standardised rates per 100,000 (95% UI) 1990	Disability adjusted life years: Age-standardised rates per 100,000 (95% UI) 2017
**Transport and unintentional injuries**	**12 632 (9 289 to 16 787)**	**25 117 (18 153 to 33 583)**	**433.4 (317.2 to 576.4)**	**428.8 (309.9 to 573.7)**	**136 620 (128 062 to 146 117)**	**116 971 (107 267 to 127 981)**	**3 456.4 (3 276.7 to 3 667.7)**	**1 883.7 (1 722.3 to 2 065.9)**
**Transport injuries**	**2 460 (1 794 to 3 248)**	**6 134 (4 452 to 8 141)**	**85.7 (62.9 to 113.0)**	**102.6 (74.5 to 136.1)**	**69 030 (64 779 to 73 344)**	**63 042 (57 989 to 68 616)**	**1 735.4 (1 647.4 to 1 825.7)**	**988.3 (911.6 to 1 076.3)**
Road injuries	2 251 (1 642 to 2 984)	5 975 (4 338 to 7 939)	79.3 (57.9 to 105.1)	99.9 (72.5 to 132.8)	66 743 (62 630 to 70 928)	59 984 (55 233 to 65 320)	1 676.9 (1 591.4 to 1 764.1)	940.6 (867.4 to 1 028.7)
Pedestrian road injuries	657 (465 to 884)	1 370 (968 to 1 860)	23.2 (16.6 to 31.2)	23.2 (16.3 to 31.6)	35 114 (32 297 to 38 201)	25 802 (23 513 to 28 330)	881.2 (823.5 to 941.1)	411.7 (375.7 to 451.3)
Cyclist road injuries	289 (209 to 384)	814 (567 to 1 084)	9.5 (6.7 to 12.5)	13.5 (9.4 to 18.0)	1 834 (1 632 to 2 023)	2 799 (2 447 to 3 178)	48.6 (43.7 to 53.3)	44.8 (39.2 to 50.9)
Motorcyclist road injuries	404 (284 to 544)	886 (605 to 1 202)	14.1 (9.9 to 19.0)	14.5 (10.0 to 19.8)	8 323 (7 492 to 9 216)	7 714 (6 706 to 8 858)	207.4 (189.8 to 227.4)	117.1 (102.2 to 134.1)
Motor vehicle road injuries	606 (434 to 795)	2 204 (1 588 to 2 940)	22.2 (16.1 to 29.1)	36.9 (26.7 to 49.2)	18 903 (17 292 to 20 635)	21 043 (18 872 to 23 387)	474.3 (435.9 to 515.0)	325.3 (292.5 to 360.6)
Other road injuries	295 (212 to 399)	701 (497 to 966)	10.2 (7.4 to 13.8)	11.7 (8.4 to 16.2)	2 569 (2 179 to 2 987)	2 626 (2 254 to 3 060)	65.4 (56.5 to 74.5)	41.6 (35.8 to 48.3)
Other transport injuries	209 (147 to 279)	159 (114 to 214)	6.5 (4.7 to 8.6)	2.7 (1.9 to 3.6)	2 287 (2 068 to 2 523)	3 058 (2 573 to 3 511)	58.5 (53.4 to 63.8)	47.7 (40.2 to 54.5)
**Unintentional injuries**	**10 172 (7 389 to 13 585)**	**18 982 (13 610 to 25 444)**	**347.6 (251.3 to 463.7)**	**326.2 (233.6 to 436.1)**	**67 589 (62 255 to 73 502)**	**53 929 (47 520 to 60 994)**	**1 720.9 (1 596.5 to 1 866.5)**	**895.4 (788.0 to 1 016.6)**
Falls	6 147 (4 516 to 8 091)	14 112 (10 128 to 18 939)	219.5 (160.0 to 287.2)	245.4 (175.2 to 328.7)	13 797 (12 070 to 15 840)	21 351 (17 218 to 26 108)	472.1 (412.8 to 542.3)	371.1 (298.5 to 454.7)
Drowning	58 (41 to 80)	42 (29 to 59)	2.0 (1.4 to 2.8)	0.7 (0.5 to 1.0)	17 360 (15 686 to 19 407)	8 213 (7 392 to 9 073)	374.5 (345.6 to 409.4)	128.6 (115.8 to 141.9)
Fire, heat, and hot substances	1 034 (646 to 1 623)	1 083 (644 to 1 753)	30.3 (18.9 to 47.3)	17.6 (10.5 to 28.6)	5 414 (4 706 to 6 245)	2 989 (2 497 to 3 696)	133.3 (117.5 to 153.2)	49.4 (41.3 to 60.7)
Poisonings	169 (106 to 257)	278 (168 to 424)	4.3 (2.7 to 6.5)	4.4 (2.7 to 6.7)	3 043 (2 708 to 3 432)	1 636 (1 423 to 1 839)	66.2 (60.3 to 73.0)	26.0 (22.7 to 29.1)
Poisoning by carbon monoxide	24 (14 to 37)	19 (12 to 30)	0.6 (0.4 to 1.0)	0.3 (0.2 to 0.5)	1 100 (976 to 1 245)	620 (540 to 698)	24.5 (22.2 to 27.2)	9.8 (8.5 to 11.0)
Poisoning by other means	145 (90 to 225)	258 (154 to 399)	3.7 (2.3 to 5.7)	4.1 (2.5 to 6.3)	1 943 (1 700 to 2 222)	1 016 (862 to 1 172)	41.7 (37.4 to 46.6)	16.2 (13.8 to 18.7)
Exposure to mechanical forces	1 386 (958 to 1 975)	1 911 (1 288 to 2 759)	47.9 (33.0 to 68.6)	31.9 (21.5 to 46.2)	9 498 (8 734 to 10 266)	5 440 (4 681 to 6 375)	248.9 (229.4 to 272.8)	87.7 (75.9 to 102.8)
Unintentional firearm injuries	178 (116 to 269)	137 (88 to 218)	6.1 (4.0 to 9.4)	2.3 (1.5 to 3.7)	4 227 (3 780 to 4 635)	1 369 (1 013 to 1 577)	103.9 (94.3 to 113.3)	21.4 (15.9 to 24.6)
Other exposure to mechanical forces	1 208 (836 to 1 729)	1 774 (1 185 to 2 583)	41.8 (28.8 to 59.9)	29.6 (19.9 to 43.0)	5 271 (4 698 to 5 968)	4 070 (3 399 to 4 946)	145.0 (129.3 to 166.4)	66.3 (55.2 to 80.4)
Adverse effects of medical treatment	31 (19 to 47)	69 (43 to 104)	1.0 (0.6 to 1.5)	1.2 (0.7 to 1.8)	2 284 (2 024 to 2 810)	1 989 (1 714 to 2 956)	60.0 (55.0 to 72.6)	34.1 (29.5 to 50.5)
Animal contact	286 (193 to 411)	308 (202 to 442)	8.5 (5.8 to 12.2)	5.1 (3.3 to 7.3)	2 623 (2 206 to 3 133)	993 (861 to 1 147)	55.0 (47.5 to 64.0)	16.4 (14.2 to 19.0)
Venomous animal contact	157 (101 to 229)	165 (107 to 243)	4.2 (2.8 to 6.1)	2.6 (1.7 to 3.9)	2 010 (1 639 to 2 468)	579 (492 to 676)	39.6 (33.2 to 47.1)	9.5 (8.1 to 11.1)
Non-venomous animal contact	130 (79 to 207)	143 (85 to 234)	4.3 (2.7 to 6.8)	2.4 (1.4 to 3.9)	612 (500 to 744)	414 (339 to 523)	15.4 (12.7 to 18.6)	6.8 (5.6 to 8.7)
Foreign body	352 (231 to 512)	418 (264 to 648)	11.0 (7.3 to 16.0)	7.1 (4.5 to 11.1)	8 349 (7 327 to 9 495)	7 718 (6 670 to 8 968)	177.5 (160.6 to 196.2)	125.4 (108.3 to 145.9)
Pulmonary aspiration and foreign body in airway	13 (9 to 18)	7 (5 to 9)	0.3 (0.2 to 0.5)	0.1 (0.1 to 0.2)	7 601 (6 613 to 8 686)	7 133 (6 126 to 8 313)	158.1 (141.5 to 176.1)	115.7 (99.3 to 134.9)
Foreign body in eyes	134 (66 to 239)	249 (129 to 440)	4.9 (2.6 to 8.6)	4.4 (2.3 to 7.7)	134 (66 to 239)	249 (129 to 440)	4.9 (2.6 to 8.6)	4.4 (2.3 to 7.7)
Foreign body in other body part	205 (142 to 275)	162 (114 to 220)	5.7 (4.0 to 7.6)	2.6 (1.9 to 3.6)	614 (500 to 729)	335 (271 to 479)	14.5 (12.0 to 17.0)	5.3 (4.3 to 7.6)
Environmental heat and cold exposure	262 (175 to 373)	176 (113 to 252)	8.4 (5.6 to 11.8)	3.0 (1.9 to 4.3)	1 084 (956 to 1 235)	521 (440 to 615)	30.9 (27.4 to 35.0)	8.7 (7.3 to 10.3)
Exposure to forces of nature	50 (36 to 69)	70 (51 to 96)	1.3 (1.0 to 1.8)	1.1 (0.8 to 1.5)	162 (144 to 185)	121 (101 to 147)	3.9 (3.5 to 4.5)	1.9 (1.6 to 2.3)
Other unintentional injuries	396 (283 to 531)	517 (362 to 709)	13.3 (9.6 to 17.9)	8.6 (6.0 to 11.9)	3 975 (3 668 to 4 297)	2 957 (2 636 to 3 352)	98.6 (91.5 to 106.8)	46.2 (41.1 to 52.4)

Death, YLL, incidence, prevalence, YLD, and DALY counts and rates per 100,000 for all injuries in Guerrero in 1990 and 2017

Cause	Deaths				Years of life lost			
	All-age counts (95% UI)		Age-standardised rates per 100,000 (95% UI)		All-age counts (95% UI)		Age-standardised rates per 100,000 (95% UI)	
	1990	2017	1990	2017	1990	2017	1990	2017
**Transport and unintentional injuries**	**1 691 (1 537 to 1 867)**	**1 421 (1 322 to 1 516)**	**65.4 (61.5 to 70.1)**	**38.2 (35.7 to 40.8)**	**108 569 (95 802 to 122 994)**	**66 961 (61 734 to 73 170)**	**3 484.9 (3 166.9 to 3 861.7)**	**1 749.0 (1 614.9 to 1 907.6)**
**Transport injuries**	**643 (593 to 697)**	**606 (553 to 659)**	**25.9 (24.3 to 27.7)**	**16.3 (14.9 to 17.7)**	**39 320 (35 653 to 43 429)**	**31 241 (28 391 to 34 501)**	**1 367.3 (1 263.7 to 1 481.7)**	**820.0 (745.2 to 900.1)**
Road injuries	623 (576 to 677)	576 (526 to 627)	25.1 (23.5 to 26.8)	15.5 (14.2 to 16.9)	38 138 (34 563 to 42 124)	29 707 (27 014 to 32 749)	1 325.9 (1 223.9 to 1 438.0)	779.4 (709.6 to 856.2)
Pedestrian road injuries	332 (300 to 371)	252 (230 to 278)	13.4 (12.4 to 14.6)	6.9 (6.3 to 7.6)	20 473 (17 982 to 23 402)	11 974 (10 735 to 13 434)	685.9 (618.8 to 765.5)	315.8 (284.2 to 353.2)
Cyclist road injuries	10 (8 to 11)	16 (14 to 18)	0.4 (0.3 to 0.4)	0.4 (0.4 to 0.5)	578 (504 to 668)	785 (686 to 906)	20.6 (18.3 to 23.2)	20.8 (18.3 to 23.9)
Motorcyclist road injuries	62 (55 to 70)	63 (55 to 74)	2.4 (2.2 to 2.7)	1.7 (1.4 to 1.9)	3 836 (3 382 to 4 337)	3 669 (3 123 to 4 343)	138.2 (122.5 to 154.7)	94.7 (81.1 to 111.5)
Motor vehicle road injuries	204 (187 to 225)	229 (204 to 254)	8.2 (7.6 to 9.1)	6.1 (5.5 to 6.8)	12 301 (11 178 to 13 639)	12 452 (10 987 to 13 981)	448.2 (411.4 to 494.9)	326.3 (288.9 to 364.8)
Other road injuries	16 (13 to 18)	16 (14 to 19)	0.6 (0.5 to 0.7)	0.4 (0.4 to 0.5)	950 (800 to 1 137)	827 (693 to 995)	33.0 (28.1 to 38.9)	21.7 (18.3 to 26.1)
Other transport injuries	19 (17 to 22)	30 (25 to 34)	0.8 (0.7 to 0.9)	0.8 (0.7 to 0.9)	1 182 (1 045 to 1 345)	1 533 (1 296 to 1 791)	41.4 (37.1 to 46.4)	40.6 (34.3 to 47.3)
**Unintentional injuries**	**1 049 (931 to 1 182)**	**815 (763 to 872)**	**39.5 (36.5 to 42.9)**	**21.9 (20.5 to 23.4)**	**69 249 (59 469 to 80 806)**	**35 720 (32 542 to 39 135)**	**2 117.6 (1 879.8 to 2 393.4)**	**929.0 (850.3 to 1 012.5)**
Falls	175 (163 to 190)	284 (254 to 306)	9.6 (9.0 to 10.4)	7.8 (7.0 to 8.4)	7 773 (6 967 to 8 795)	7 417 (6 525 to 8 049)	314.0 (289.8 to 343.1)	203.5 (179.2 to 220.4)
Drowning	181 (158 to 212)	114 (103 to 126)	6.1 (5.5 to 6.8)	3.0 (2.7 to 3.3)	12 746 (10 786 to 15 348)	6 679 (5 914 to 7 506)	378.0 (329.7 to 441.5)	170.9 (151.8 to 191.4)
Fire, heat, and hot substances	65 (55 to 78)	42 (37 to 45)	2.4 (2.1 to 2.7)	1.1 (1.0 to 1.2)	4 458 (3 593 to 5 569)	1 715 (1 496 to 1 928)	130.0 (109.4 to 155.8)	44.9 (39.2 to 50.1)
Poisonings	44 (38 to 52)	30 (26 to 33)	1.5 (1.3 to 1.7)	0.8 (0.7 to 0.9)	3 160 (2 649 to 3 843)	1 664 (1 413 to 1 863)	92.2 (79.8 to 108.9)	42.8 (36.4 to 47.7)
Poisoning by carbon monoxide	6 (5 to 8)	5 (4 to 6)	0.2 (0.2 to 0.3)	0.1 (0.1 to 0.2)	434 (360 to 528)	276 (228 to 323)	13.4 (11.4 to 15.9)	7.2 (5.9 to 8.3)
Poisoning by other means	38 (33 to 45)	25 (21 to 27)	1.3 (1.1 to 1.4)	0.7 (0.6 to 0.7)	2 726 (2 265 to 3 331)	1 388 (1 186 to 1 561)	78.8 (68.0 to 93.5)	35.6 (30.5 to 39.9)
Exposure to mechanical forces	135 (125 to 145)	79 (71 to 86)	5.6 (5.2 to 5.9)	2.2 (1.9 to 2.4)	8 033 (7 328 to 8 756)	3 768 (3 347 to 4 153)	291.5 (270.7 to 313.7)	100.3 (89.3 to 110.4)
Unintentional firearm injuries	75 (62 to 82)	33 (27 to 38)	3.1 (2.6 to 3.4)	0.9 (0.6 to 1.0)	4 402 (3 664 to 4 885)	1 655 (1 105 to 1 922)	164.8 (138.6 to 180.3)	44.0 (29.3 to 51.1)
Other exposure to mechanical forces	60 (53 to 73)	46 (41 to 55)	2.5 (2.2 to 3.0)	1.2 (1.1 to 1.5)	3 632 (3 126 to 4 408)	2 113 (1 851 to 2 634)	126.7 (111.3 to 154.8)	56.2 (49.3 to 69.9)
Adverse effects of medical treatment	40 (34 to 46)	49 (44 to 62)	1.7 (1.5 to 2.0)	1.3 (1.2 to 1.7)	2 429 (1 929 to 2 898)	1 829 (1 584 to 2 341)	75.6 (63.0 to 87.3)	47.6 (41.5 to 60.8)
Animal contact	195 (152 to 247)	54 (48 to 61)	5.5 (4.5 to 6.8)	1.4 (1.3 to 1.6)	15 540 (11 883 to 20 040)	2 921 (2 488 to 3 485)	397.3 (309.2 to 505.6)	73.8 (63.5 to 86.2)
Venomous animal contact	184 (140 to 235)	45 (39 to 52)	5.1 (4.0 to 6.3)	1.2 (1.0 to 1.4)	14 815 (11 066 to 19 156)	2 512 (2 088 to 3 018)	375.4 (283.9 to 480.6)	63.3 (53.0 to 75.5)
Non-venomous animal contact	11 (6 to 29)	8 (7 to 12)	0.4 (0.2 to 0.9)	0.2 (0.2 to 0.3)	725 (377 to 2 227)	410 (306 to 572)	21.9 (12.2 to 59.0)	10.6 (7.9 to 14.7)
Foreign body	146 (123 to 172)	118 (104 to 134)	4.4 (3.9 to 5.0)	3.0 (2.7 to 3.4)	11 152 (9 165 to 13 461)	7 369 (6 232 to 8 619)	297.2 (251.2 to 351.3)	188.1 (155.9 to 212.3)
Pulmonary aspiration and foreign body in airway	140 (118 to 165)	115 (102 to 131)	4.2 (3.7 to 4.8)	3.0 (2.6 to 3.3)	10 735 (8 803 to 12 909)	7 189 (6 054 to 8 437)	284.9 (239.5 to 335.9)	178.4 (151.6 to 207.8)
Foreign body in eyes								
Foreign body in other body part	6 (4 to 7)	3 (2 to 4)	0.2 (0.1 to 0.2)	0.1 (0.1 to 0.1)	417 (282 to 540)	180 (145 to 256)	12.3 (8.8 to 15.2)	4.7 (3.8 to 6.6)
Environmental heat and cold exposure	24 (23 to 26)	8 (7 to 9)	1.2 (1.2 to 1.3)	0.2 (0.2 to 0.3)	1 219 (1 118 to 1 340)	335 (284 to 372)	49.9 (47.3 to 53.1)	9.2 (7.8 to 10.3)
Exposure to forces of nature	1 (1 to 1)	5 (4 to 5)	0.0 (0.0 to 0.1)	0.1 (0.1 to 0.1)	68 (61 to 75)	237 (216 to 261)	2.3 (2.1 to 2.6)	6.2 (5.7 to 6.8)
Other unintentional injuries	42 (38 to 46)	33 (29 to 37)	1.6 (1.5 to 1.7)	0.9 (0.8 to 1.0)	2 670 (2 397 to 2 971)	1 790 (1 580 to 2 017)	89.5 (81.5 to 98.5)	46.8 (41.5 to 52.6)

Cause	Incidence				Prevalence			
	All-age counts (95% UI)		Age-standardised rates per 100,000 (95% UI)		All-age counts (95% UI)		Age-standardised rates per 100,000 (95% UI)	
	1990	2017	1990	2017	1990	2017	1990	2017
**Transport and unintentional injuries**	**111 686 (102 628 to 121 964)**	**164 326 (149 792 to 179 776)**	**4 525.9 (4 125.2 to 4 956.1)**	**4 433.8 (4 036.3 to 4 877.8)**	**205 493 (192 500 to 219 617)**	**336 526 (315 389 to 363 614)**	**10 404.0 (9 809.5 to 11 142.6)**	**9 391.7 (8 808.7 to 10 137.5)**
**Transport injuries**	**7 697 (6 568 to 9 017)**	**16 757 (14 450 to 19 216)**	**314.1 (272.6 to 359.0)**	**450.0 (391.3 to 511.3)**	**18 378 (17 095 to 19 643)**	**46 981 (43 477 to 50 699)**	**968.4 (922.3 to 1 057.4)**	**1 328.5 (1 230.1 to 1 434.7)**
Road injuries	7 327 (6 208 to 8 664)	16 423 (14 120 to 18 838)	300.9 (259.8 to 344.4)	441.0 (380.4 to 504.2)	16 752 (15 542 to 17 979)	45 315 (41 954 to 48 957)	909.6 (845.4 to 976.0)	1 281.7 (1 186.7 to 1 387.3)
Pedestrian road injuries	1 314 (1 097 to 1 644)	1 887 (1 527 to 2 288)	54.6 (44.9 to 66.1)	51.9 (41.6 to 62.2)	4 754 (4 094 to 5 635)	8 725 (7 565 to 10 158)	256.2 (220.6 to 301.2)	246.3 (213.1 to 286.6)
Cyclist road injuries	859 (618 to 1 171)	1 556 (1 174 to 2 038)	31.5 (24.0 to 41.4)	40.4 (30.6 to 52.6)	1 391 (1 171 to 1 656)	3 399 (2 854 to 4 063)	70.5 (59.9 to 82.5)	94.2 (79.3 to 112.4)
Motorcyclist road injuries	832 (642 to 1 062)	1 493 (1 138 to 1 920)	32.8 (25.8 to 40.7)	39.1 (30.0 to 50.2)	2 987 (2 594 to 3 458)	6 773 (5 781 to 7 952)	160.1 (138.4 to 184.7)	191.5 (163.6 to 224.6)
Motor vehicle road injuries	2 915 (2 377 to 3 486)	8 572 (7 202 to 10 075)	125.0 (103.4 to 146.6)	232.9 (196.0 to 272.7)	5 127 (4 469 to 5 925)	19 580 (17 125 to 22 756)	289.3 (251.3 to 333.1)	557.5 (486.8 to 647.7)
Other road injuries	1 406 (1 062 to 1 787)	2 914 (2 307 to 3 558)	57.1 (45.2 to 68.8)	77.9 (62.5 to 94.9)	2 493 (2 071 to 2 995)	6 838 (5 726 to 8 233)	133.5 (111.2 to 159.8)	192.3 (160.9 to 232.2)
Other transport injuries	370 (272 to 509)	334 (263 to 429)	13.4 (10.6 to 16.8)	9.0 (7.1 to 11.4)	1 626 (1 350 to 1 928)	1 666 (1 436 to 1 898)	78.8 (66.5 to 91.6)	46.8 (40.4 to 53.4)
**Unintentional injuries**	**103 989 (94 847 to 114 250)**	**147 569 (133 738 to 161 406)**	**4 211.8 (3 820.5 to 4 644.5)**	**3 983.9 (3 596.6 to 4 423.8)**	**187 115 (174 812 to 201 146)**	**289 545 (268 556 to 316 533)**	**9 415.6 (8 826.7 to 10 131.3)**	**8 063.2 (7 480.9 to 8 804.0)**
Falls	42 801 (37 409 to 49 179)	84 470 (74 831 to 95 213)	1 869.1 (1 675.3 to 2 100.7)	2 287.7 (2 031.0 to 2 577.8)	73 116 (64 140 to 83 332)	160 174 (142 917 to 182 393)	3 895.9 (3 483.1 to 4 460.6)	4 450.3 (3 976.5 to 5 084.5)
Drowning	132 (113 to 153)	83 (66 to 103)	4.7 (4.1 to 5.3)	2.2 (1.8 to 2.7)	570 (498 to 662)	492 (435 to 571)	29.8 (26.3 to 34.2)	13.9 (12.2 to 16.1)
Fire, heat, and hot substances	3 733 (2 931 to 4 701)	3 233 (2 639 to 4 002)	109.9 (89.1 to 134.7)	82.8 (67.7 to 101.5)	33 295 (27 509 to 42 591)	35 282 (30 100 to 45 781)	1 480.2 (1 236.2 to 1 838.2)	998.0 (829.6 to 1 251.6)
Poisonings	1 403 (1 064 to 1 788)	1 706 (1 300 to 2 193)	37.6 (29.3 to 47.5)	42.5 (32.5 to 54.4)	1 138 (863 to 1 398)	1 759 (1 327 to 2 164)	45.1 (34.8 to 55.1)	47.2 (35.7 to 58.0)
Poisoning by carbon monoxide	229 (155 to 337)	177 (132 to 254)	6.9 (5.0 to 9.5)	4.6 (3.0 to 6.6)	146 (113 to 191)	114 (90 to 143)	6.0 (4.7 to 7.7)	3.1 (2.5 to 3.9)
Poisoning by other means	1 174 (872 to 1 519)	1 529 (1 157 to 2 005)	30.7 (23.4 to 39.2)	37.8 (28.6 to 49.5)	991 (722 to 1 242)	1 645 (1 203 to 2 046)	39.1 (28.7 to 48.9)	44.1 (32.3 to 54.8)
Exposure to mechanical forces	22 302 (18 838 to 26 368)	23 029 (19 822 to 26 653)	858.8 (728.9 to 995.0)	613.1 (528.2 to 705.9)	42 564 (37 372 to 48 729)	55 018 (48 434 to 63 573)	2 258.0 (1 987.8 to 2 582.3)	1 551.1 (1 362.6 to 1 787.9)
Unintentional firearm injuries	1 926 (1 443 to 2 539)	1 385 (1 046 to 1 785)	75.9 (56.9 to 99.1)	37.0 (27.9 to 47.3)	6 741 (5 398 to 8 133)	6 535 (5 318 to 7 841)	373.2 (298.2 to 452.7)	185.9 (151.2 to 223.7)
Other exposure to mechanical forces	20 376 (17 163 to 24 101)	21 638 (18 587 to 25 089)	777.9 (664.1 to 909.7)	576.1 (495.7 to 666.2)	35 823 (30 374 to 42 232)	48 478 (41 491 to 57 289)	1 884.8 (1 612.9 to 2 225.9)	1 365.2 (1 167.9 to 1 612.2)
Adverse effects of medical treatment	1 672 (1 384 to 1 976)	2 898 (2 399 to 3 361)	77.3 (65.4 to 90.4)	79.2 (66.7 to 93.8)	128 (98 to 162)	217 (167 to 274)	5.9 (4.5 to 7.3)	6.1 (4.6 to 7.6)
Animal contact	16 380 (13 863 to 19 270)	10 283 (9 029 to 11 735)	582.6 (512.3 to 663.6)	274.0 (241.4 to 310.9)	16 269 (13 732 to 19 135)	12 270 (10 835 to 13 899)	776.1 (674.2 to 895.3)	341.2 (301.3 to 385.5)
Venomous animal contact	6 883 (5 369 to 8 609)	3 966 (3 183 to 4 846)	243.1 (195.3 to 297.7)	105.5 (85.0 to 128.5)	4 812 (3 860 to 6 022)	2 974 (2 473 to 3 480)	215.2 (177.2 to 263.6)	81.9 (68.3 to 95.7)
Non-venomous animal contact	9 497 (7 772 to 11 552)	6 317 (5 385 to 7 494)	339.5 (287.1 to 401.6)	168.5 (143.5 to 199.5)	11 457 (9 315 to 14 231)	9 296 (7 898 to 10 882)	560.9 (462.1 to 672.2)	259.3 (220.7 to 303.5)
Foreign body	9 687 (5 004 to 15 579)	15 615 (6 909 to 27 179)	455.3 ([illegible] to 778.7)	435.9 (188.2 to 768.5)	3 828 (2 863 to 5 272)	5 158 (3 653 to 7 324)	193.1 (144.1 to 256.3)	143.9 (101.5 to 205.4)
Pulmonary aspiration and foreign body in airway	254 (167 to 374)	119 (88 to 162)	7.0 (4.8 to 9.9)	3.1 (2.3 to 4.1)	168 (119 to 223)	95 (77 to 116)	6.5 (4.7 to 8.8)	2.6 (2.1 to 3.2)
Foreign body in eyes	8 785 (4 119 to 14 644)	15 025 (6 241 to 26 643)	426.8 (164.5 to 748.5)	420.5 (170.4 to 752.5)	1 763 (912 to 3 055)	3 405 (1 913 to 5 730)	97.1 (54.1 to 157.8)	95.8 (54.2 to 157.2)
Foreign body in other body part	649 (425 to 1 059)	471 (327 to 687)	21.5 (14.5 to 32.7)	12.3 (8.6 to 16.6)	1 902 (1 529 to 2 409)	1 658 (1 370 to 2 054)	87.5 (71.9 to 107.9)	45.6 (37.8 to 56.3)
Environmental heat and cold exposure	1 172 (825 to 1 601)	613 (465 to 827)	48.9 (32.8 to 65.8)	16.3 (12.4 to 22.1)	3 943 (3 226 to 4 817)	2 245 (1 916 to 2 666)	192.6 (161.9 to 230.7)	62.2 (53.1 to 73.8)
Exposure to forces of nature	177 (145 to 222)	316 (266 to 379)	5.9 (5.0 to 7.2)	8.2 (7.0 to 9.8)	6 000 (4 181 to 8 513)	6 679 (4 664 to 9 323)	222.7 (160.1 to 309.7)	190.8 (133.1 to 266.3)
Other unintentional injuries	4 530 (3 745 to 5 533)	5 390 (4 572 to 6 321)	171.4 (145.6 to 202.3)	142.0 (120.6 to 165.6)	6 264 (5 370 to 7 214)	9 266 (8 026 to 10 644)	318.2 (275.4 to 362.8)	258.9 (224.5 to 297.3)

Cause	Years lived with disability				Disability adjusted life years			
	All-age counts (95% UI)		Age-standardised rates per 100,000 (95% UI)		All-age counts (95% UI)		Age-standardised rates per 100,000 (95% UI)	
	1990	2017	1990	2017	1990	2017	1990	2017
**Transport and unintentional injuries**	**9 236 (6 765 to 12 096)**	**14 951 (10 868 to 20 091)**	**459.8 (336.9 to 604.8)**	**416.2 (302.6 to 559.2)**	**117 805 (105 112 to 132 410)**	**81 912 (75 039 to 89 582)**	**3 944.7 (3 615.2 to 4 324.0)**	**2 165.2 (1 980.6 to 2 367.8)**
**Transport injuries**	**1 294 (941 to 1 709)**	**2 920 (2 124 to 3 881)**	**67.5 (49.4 to 88.7)**	**82.4 (60.1 to 109.4)**	**40 613 (36 882 to 44 766)**	**34 161 (31 117 to 37 672)**	**1 434.8 (1 328.5 to 1 554.1)**	**902.4 (822.3 to 991.2)**
Road injuries	1 186 (857 to 1 570)	2 828 (2 057 to 3 760)	62.5 (46.1 to 82.3)	79.8 (58.2 to 106.0)	39 324 (35 729 to 43 395)	32 535 (29 639 to 35 987)	1 388.4 (1 284.0 to 1 500.8)	859.2 (783.1 to 946.5)
Pedestrian road injuries	351 (246 to 477)	567 (400 to 775)	18.3 (13.0 to 24.6)	16.0 (11.3 to 21.9)	20 825 (18 307 to 23 759)	12 541 (11 279 to 14 055)	704.2 (634.9 to 783.1)	331.8 (299.4 to 370.4)
Cyclist road injuries	101 (70 to 133)	213 (148 to 281)	5.0 (3.5 to 6.5)	5.9 (4.1 to 7.8)	678 (601 to 768)	998 (883 to 1 139)	25.5 (22.9 to 28.4)	26.7 (23.6 to 30.3)
Motorcyclist road injuries	213 (151 to 289)	426 (293 to 581)	11.1 (7.9 to 14.9)	12.0 (8.3 to 16.4)	4 049 (3 589 to 4 557)	4 095 (3 546 to 4 800)	149.4 (133.8 to 165.7)	106.7 (93.0 to 123.6)
Motor vehicle road injuries	377 (273 to 501)	1 267 (915 to 1 675)	20.6 (14.9 to 27.2)	36.0 (26.0 to 47.6)	12 678 (11 533 to 14 030)	13 719 (12 176 to 15 220)	468.8 (430.6 to 517.1)	362.3 (323.6 to 399.9)
Other road injuries	145 (104 to 196)	355 (254 to 486)	7.5 (5.5 to 10.2)	9.9 (7.1 to 13.6)	1 094 (935 to 1 287)	1 182 (1 013 to 1 382)	40.5 (35.3 to 46.6)	31.7 (27.1 to 37.0)
Other transport injuries	107 (76 to 143)	92 (67 to 122)	5.0 (3.6 to 6.6)	2.6 (1.9 to 3.4)	1 289 (1 149 to 1 450)	1 625 (1 377 to 1 879)	46.4 (41.9 to 51.4)	43.2 (36.5 to 49.7)
**Unintentional injuries**	**7 942 (5 802 to 10 429)**	**12 031 (8 673 to 16 227)**	**392.3 (285.5 to 519.3)**	**333.8 (241.1 to 449.3)**	**77 192 (67 043 to 89 096)**	**47 751 (43 144 to 53 212)**	**2 509.9 (2 245.0 to 2 810.0)**	**1 262.8 (1 144.8 to 1 407.7)**
Falls	4 511 (3 303 to 5 857)	8 568 (6 197 to 11 326)	234.0 (170.5 to 303.8)	237.5 (171.7 to 313.6)	12 285 (10 793 to 13 996)	15 985 (13 390 to 18 788)	548.0 (479.4 to 625.5)	441.0 (370.1 to 517.9)
Drowning	33 (23 to 46)	26 (18 to 36)	1.7 (1.2 to 2.3)	0.7 (0.5 to 1.0)	12 780 (10 812 to 15 379)	6 705 (5 938 to 7 530)	379.7 (330.9 to 443.3)	171.7 (152.7 to 192.4)
Fire, heat, and hot substances	738 (464 to 1 146)	721 (480 to 1 160)	31.7 (19.9 to 49.3)	19.7 (11.8 to 31.8)	5 196 (4 268 to 6 380)	2 437 (2 056 to 2 924)	161.8 (136.7 to 193.8)	64.6 (54.3 to 77.9)
Poisonings	136 (84 to 204)	201 (124 to 302)	5.2 (3.2 to 7.8)	5.4 (3.3 to 8.1)	3 296 (2 780 to 3 973)	1 866 (1 611 to 2 090)	97.4 (84.4 to 113.9)	48.1 (41.5 to 53.7)
Poisoning by carbon monoxide	16 (10 to 25)	11 (6 to 17)	0.6 (0.4 to 1.0)	0.3 (0.2 to 0.4)	450 (375 to 547)	287 (239 to 334)	14.0 (12.0 to 16.6)	7.4 (6.2 to 8.6)
Poisoning by other means	120 (73 to 182)	191 (116 to 287)	4.6 (2.8 to 7.0)	5.1 (3.1 to 7.7)	2 846 (2 391 to 3 441)	1 579 (1 362 to 1 772)	83.5 (72.1 to 98.1)	40.7 (35.2 to 45.5)
Exposure to mechanical forces	1 025 (713 to 1 448)	1 220 (827 to 1 762)	53.3 (37.0 to 76.1)	34.3 (23.3 to 49.6)	9 059 (8 269 to 9 945)	4 988 (4 401 to 5 657)	344.8 (316.7 to 376.8)	134.6 (118.5 to 152.9)
Unintentional firearm injuries	144 (96 to 216)	125 (82 to 194)	7.8 (5.1 to 11.7)	3.5 (2.3 to 5.5)	4 546 (3 796 to 4 984)	1 780 (1 214 to 2 057)	172.5 (145.6 to 188.4)	47.6 (32.4 to 55.1)
Other exposure to mechanical forces	881 (609 to 1 246)	1 095 (743 to 1 594)	45.6 (31.4 to 64.5)	30.8 (20.9 to 44.9)	4 513 (3 933 to 5 387)	3 208 (2 753 to 3 887)	172.3 (151.1 to 204.7)	87.0 (74.4 to 105.5)
Adverse effects of medical treatment	17 (10 to 26)	29 (18 to 44)	0.8 (0.5 to 1.2)	0.8 (0.5 to 1.2)	2 446 (1 945 to 2 917)	1 852 (1 619 to 2 374)	76.4 (63.6 to 88.0)	48.4 (42.5 to 61.4)
Animal contact	472 (333 to 661)	288 (192 to 415)	20.3 (13.7 to 28.5)	7.9 (5.2 to 11.3)	16 012 (12 365 to 20 572)	3 209 (2 769 to 3 721)	417.6 (330.0 to 528.6)	81.7 (71.2 to 94.1)
Venomous animal contact	294 (187 to 430)	153 (99 to 219)	11.7 (7.5 to 16.9)	4.1 (2.7 to 5.9)	15 109 (11 309 to 19 579)	2 665 (2 224 to 3 168)	387.2 (294.4 to 496.8)	67.4 (56.6 to 79.6)
Non-venomous animal contact	178 (106 to 292)	135 (81 to 218)	8.6 (5.2 to 13.6)	3.8 (2.3 to 6.1)	903 (521 to 2 401)	545 (420 to 742)	30.5 (19.1 to 69.2)	14.3 (11.1 to 19.5)
Foreign body	247 (160 to 371)	314 (189 to 516)	12.2 (7.9 to 18.3)	8.8 (5.3 to 14.5)	11 399 (9 424 to 13 681)	7 683 (6 540 to 9 002)	309.3 (262.2 to 363.0)	191.9 (165.1 to 222.7)
Pulmonary aspiration and foreign body in airway	9 (6 to 13)	4 (3 to 6)	0.3 (0.2 to 0.5)	0.1 (0.1 to 0.2)	10 744 (8 816 to 12 918)	7 193 (6 058 to 8 441)	285.3 (239.8 to 337.4)	178.6 (151.7 to 207.9)
Foreign body in eyes	117 (59 to 216)	226 (121 to 406)	6.5 (3.5 to 11.6)	6.4 (3.4 to 11.6)	117 (59 to 216)	226 (121 to 406)	6.5 (3.5 to 11.6)	6.4 (3.4 to 11.6)
Foreign body in other body part	121 (84 to 161)	84 (60 to 112)	5.3 (3.8 to 7.0)	2.3 (1.6 to 3.0)	538 (401 to 667)	263 (215 to 350)	17.5 (13.7 to 21.0)	6.9 (5.7 to 9.1)
Environmental heat and cold exposure	159 (106 to 223)	87 (58 to 125)	7.7 (5.2 to 10.7)	2.4 (1.6 to 3.5)	1 377 (1 260 to 1 525)	421 (365 to 481)	57.6 (53.7 to 62.3)	11.6 (10.0 to 13.2)
Exposure to forces of nature	351 (238 to 499)	256 (184 to 352)	12.8 (9.1 to 17.7)	7.3 (5.3 to 10.0)	418 (306 to 564)	493 (417 to 588)	15.1 (11.4 to 20.0)	13.5 (11.4 to 16.2)
Other unintentional injuries	254 (182 to 338)	319 (226 to 437)	12.7 (9.1 to 16.8)	8.9 (6.3 to 12.2)	2 925 (2 636 to 3 235)	2 110 (1 868 to 2 370)	102.2 (93.0 to 111.5)	55.7 (48.3 to 62.4)

Death, YLL, incidence, prevalence, YLD, and DALY counts and rates per 100,000 for all injuries in Hidalgo in 1990 and 2017

Deaths

Cause	All-age counts (95% UI) 1990	All-age counts (95% UI) 2017	Age-standardised rates per 100,000 (95% UI) 1990	Age-standardised rates per 100,000 (95% UI) 2017
**Transport and unintentional injuries**	**1 135 (1 077 to 1 204)**	**1 054 (981 to 1 125)**	**68.7 (66.3 to 71.4)**	**35.1 (32.7 to 37.4)**
**Transport injuries**	**463 (438 to 492)**	**495 (456 to 536)**	**26.9 (25.6 to 28.4)**	**16.1 (14.9 to 17.4)**
Road injuries	454 (430 to 482)	482 (444 to 522)	26.4 (25.1 to 27.9)	15.7 (14.5 to 17.0)
Pedestrian road injuries	209 (194 to 226)	185 (168 to 209)	12.7 (11.9 to 13.5)	6.1 (5.6 to 6.7)
Cyclist road injuries	10 (9 to 11)	17 (15 to 19)	0.6 (0.5 to 0.7)	0.6 (0.5 to 0.6)
Motorcyclist road injuries	64 (58 to 71)	56 (49 to 65)	3.5 (3.2 to 3.9)	1.8 (1.6 to 2.1)
Motor vehicle road injuries	159 (147 to 176)	214 (193 to 234)	8.9 (8.2 to 9.8)	6.9 (6.2 to 7.5)
Other road injuries	12 (10 to 14)	10 (9 to 12)	0.7 (0.6 to 0.8)	0.3 (0.3 to 0.4)
Other transport injuries	9 (8 to 10)	13 (11 to 15)	0.5 (0.5 to 0.6)	0.4 (0.4 to 0.5)
**Unintentional injuries**	**672 (630 to 718)**	**560 (519 to 598)**	**41.7 (40.2 to 43.6)**	**19.0 (17.6 to 20.3)**
Falls	148 (141 to 157)	195 (178 to 210)	12.4 (11.8 to 13.1)	6.8 (6.2 to 7.4)
Drowning	158 (145 to 174)	89 (81 to 97)	8.3 (7.8 to 9.0)	2.9 (2.7 to 3.2)
Fire, heat, and hot substances	44 (40 to 50)	29 (27 to 31)	2.8 (2.6 to 3.0)	1.0 (0.9 to 1.1)
Poisonings	33 (30 to 37)	19 (18 to 21)	1.7 (1.6 to 1.9)	0.6 (0.6 to 0.7)
Poisoning by carbon monoxide	15 (13 to 16)	9 (9 to 10)	0.8 (0.7 to 0.8)	0.3 (0.3 to 0.3)
Poisoning by other means	19 (17 to 21)	10 (9 to 11)	1.0 (0.9 to 1.0)	0.3 (0.3 to 0.4)
Exposure to mechanical forces	89 (83 to 95)	50 (46 to 55)	5.3 (5.0 to 5.7)	1.6 (1.5 to 1.8)
Unintentional firearm injuries	41 (37 to 45)	17 (14 to 19)	2.3 (2.1 to 2.6)	0.6 (0.5 to 0.6)
Other exposure to mechanical forces	48 (43 to 53)	33 (30 to 37)	3.0 (2.7 to 3.3)	1.1 (1.0 to 1.2)
Adverse effects of medical treatment	29 (25 to 32)	35 (31 to 46)	2.0 (1.8 to 2.2)	1.2 (1.1 to 1.6)
Animal contact	17 (15 to 19)	9 (8 to 10)	0.9 (0.8 to 1.0)	0.3 (0.3 to 0.3)
Venomous animal contact	12 (10 to 14)	5 (4 to 6)	0.6 (0.5 to 0.7)	0.2 (0.1 to 0.2)
Non-venomous animal contact	5 (4 to 6)	4 (3 to 5)	0.3 (0.2 to 0.3)	0.1 (0.1 to 0.2)
Foreign body	105 (93 to 119)	106 (95 to 118)	5.3 (4.9 to 5.8)	3.5 (3.2 to 4.0)
Pulmonary aspiration and foreign body in airway	100 (88 to 114)	103 (93 to 115)	5.1 (4.7 to 5.6)	3.5 (3.1 to 3.9)
Foreign body in eyes				
Foreign body in other body part	5 (4 to 6)	2 (2 to 4)	0.2 (0.2 to 0.3)	0.1 (0.1 to 0.1)
Environmental heat and cold exposure	14 (13 to 15)	7 (6 to 8)	1.0 (0.9 to 1.1)	0.2 (0.2 to 0.3)
Exposure to forces of nature	4 (3 to 5)	0 (0 to 1)	0.2 (0.2 to 0.3)	0.0 (0.0 to 0.0)
Other unintentional injuries	31 (28 to 33)	21 (19 to 23)	1.7 (1.6 to 1.9)	0.7 (0.6 to 0.8)

Years of life lost

Cause	All-age counts (95% UI) 1990	All-age counts (95% UI) 2017	Age-standardised rates per 100,000 (95% UI) 1990	Age-standardised rates per 100,000 (95% UI) 2017
**Transport and unintentional injuries**	**62 937 (58 293 to 68 341)**	**46 544 (43 204 to 50 010)**	**3 122.7 (2 955.4 to 3 318.9)**	**1 514.4 (1 406.8 to 1 627.3)**
**Transport injuries**	**26 234 (24 499 to 28 177)**	**24 444 (22 415 to 26 436)**	**1 333.7 (1 258.1 to 1 419.4)**	**785.9 (721.3 to 850.0)**
Road injuries	25 741 (24 036 to 27 640)	23 829 (21 857 to 25 781)	1 308.4 (1 235.1 to 1 390.4)	766.1 (703.5 to 828.6)
Pedestrian road injuries	11 500 (10 447 to 12 730)	8 118 (7 305 to 9 041)	580.8 (536.6 to 630.5)	269.4 (237.4 to 299.0)
Cyclist road injuries	566 (505 to 637)	774 (680 to 888)	29.7 (26.7 to 33.3)	25.0 (21.9 to 28.7)
Motorcyclist road injuries	3 774 (3 375 to 4 223)	3 151 (2 728 to 3 650)	192.3 (173.6 to 213.4)	100.3 (86.7 to 115.9)
Motor vehicle road injuries	9 225 (8 400 to 10 274)	11 283 (10 202 to 12 374)	471.4 (434.3 to 521.5)	361.2 (326.7 to 396.1)
Other road injuries	675 (547 to 808)	502 (430 to 588)	34.2 (27.8 to 39.8)	16.1 (13.8 to 18.9)
Other transport injuries	493 (436 to 559)	616 (537 to 701)	25.3 (22.7 to 28.5)	19.8 (17.3 to 22.5)
**Unintentional injuries**	**36 703 (33 409 to 40 378)**	**22 100 (20 290 to 23 990)**	**1 788.9 (1 671.9 to 1 914.8)**	**728.6 (668.4 to 792.8)**
Falls	5 302 (4 932 to 5 705)	4 928 (4 489 to 5 361)	339.7 (321.8 to 360.3)	165.0 (150.5 to 179.4)
Drowning	9 850 (8 807 to 11 091)	4 624 (4 169 to 5 124)	449.1 (410.1 to 494.3)	150.2 (135.3 to 166.6)
Fire, heat, and hot substances	2 448 (2 091 to 2 860)	957 (871 to 1 044)	113.5 (101.8 to 128.2)	31.8 (29.0 to 34.7)
Poisonings	2 125 (1 868 to 2 426)	929 (841 to 1 026)	93.8 (85.1 to 104.4)	30.3 (27.4 to 33.5)
Poisoning by carbon monoxide	908 (796 to 1 046)	455 (406 to 509)	41.3 (37.1 to 46.4)	14.8 (13.2 to 16.6)
Poisoning by other means	1 217 (1 053 to 1 406)	473 (423 to 531)	52.6 (47.0 to 59.1)	15.5 (13.8 to 17.4)
Exposure to mechanical forces	4 888 (4 528 to 5 291)	2 245 (2 036 to 2 478)	255.1 (238.3 to 273.4)	72.4 (65.7 to 79.9)
Unintentional firearm injuries	2 321 (2 073 to 2 594)	831 (691 to 951)	119.3 (107.9 to 132.9)	26.6 (22.1 to 30.5)
Other exposure to mechanical forces	2 568 (2 282 to 2 856)	1 414 (1 264 to 1 609)	135.7 (122.0 to 149.6)	45.8 (41.0 to 52.1)
Adverse effects of medical treatment	1 386 (1 190 to 1 618)	1 095 (954 to 1 563)	68.7 (57.8 to 77.3)	36.8 (32.1 to 52.4)
Animal contact	1 061 (893 to 1 258)	360 (321 to 402)	45.2 (39.5 to 51.8)	12.0 (10.7 to 13.4)
Venomous animal contact	770 (624 to 944)	214 (173 to 247)	31.6 (26.4 to 37.4)	7.2 (5.8 to 8.3)
Non-venomous animal contact	291 (230 to 350)	146 (125 to 187)	13.6 (10.9 to 15.9)	4.8 (4.1 to 6.2)
Foreign body	6 945 (5 975 to 8 137)	5 623 (4 830 to 6 545)	287.3 (254.5 to 326.2)	186.9 (160.3 to 218.5)
Pulmonary aspiration and foreign body in airway	6 617 (5 688 to 7 753)	5 498 (4 720 to 6 419)	272.9 (241.8 to 310.8)	182.9 (156.6 to 214.1)
Foreign body in eyes				
Foreign body in other body part	329 (239 to 393)	125 (103 to 207)	14.4 (10.8 to 16.9)	4.0 (3.3 to 6.7)
Environmental heat and cold exposure	686 (619 to 760)	251 (224 to 277)	37.0 (34.3 to 40.2)	8.2 (7.3 to 9.0)
Exposure to forces of nature	238 (189 to 301)	25 (23 to 27)	11.5 (9.2 to 14.4)	0.8 (0.7 to 0.9)
Other unintentional injuries	1 778 (1 621 to 1 939)	1 058 (950 to 1 197)	88.1 (81.2 to 95.9)	34.1 (30.5 to 38.4)

Incidence

Cause	All-age counts (95% UI) 1990	All-age counts (95% UI) 2017	Age-standardised rates per 100,000 (95% UI) 1990	Age-standardised rates per 100,000 (95% UI) 2017
**Transport and unintentional injuries**	**81 143 (74 774 to 88 500)**	**148 034 (135 438 to 161 888)**	**4 621.1 (4 242.3 to 5 040.8)**	**4 942.2 (4 519.3 to 5 401.3)**
**Transport injuries**	**6 198 (5 303 to 7 222)**	**15 048 (13 007 to 17 344)**	**343.3 (296.5 to 391.0)**	**488.5 (423.2 to 563.0)**
Road injuries	5 914 (5 050 to 6 941)	14 838 (12 792 to 17 144)	329.9 (283.7 to 377.9)	481.5 (416.2 to 555.9)
Pedestrian road injuries	899 (720 to 1 118)	1 495 (1 199 to 1 825)	51.8 (42.8 to 62.6)	49.0 (39.2 to 59.8)
Cyclist road injuries	941 (721 to 1 225)	2 146 (1 696 to 2 693)	48.1 (38.1 to 60.3)	70.2 (55.5 to 87.8)
Motorcyclist road injuries	740 (585 to 935)	1 204 (930 to 1 534)	39.9 (31.7 to 49.2)	38.8 (29.9 to 49.4)
Motor vehicle road injuries	2 286 (1 869 to 2 740)	7 793 (6 456 to 9 168)	132.1 (110.0 to 155.6)	249.6 (209.0 to 296.0)
Other road injuries	1 049 (796 to 1 338)	2 260 (1 804 to 2 764)	57.9 (45.4 to 71.0)	74.0 (59.0 to 93.0)
Other transport injuries	284 (197 to 400)	210 (166 to 267)	13.5 (10.1 to 17.6)	7.0 (5.5 to 8.9)
**Unintentional injuries**	**74 945 (68 614 to 82 195)**	**132 986 (120 573 to 146 916)**	**4 277.8 (3 886.7 to 4 702.6)**	**4 453.7 (4 038.7 to 4 917.9)**
Falls	34 687 (30 620 to 39 331)	79 935 (70 489 to 89 788)	2 124.6 (1 898.0 to 2 382.6)	2 709.4 (2 398.1 to 3 044.0)
Drowning	136 (117 to 158)	73 (59 to 92)	7.0 (6.1 to 7.9)	2.4 (2.0 to 3.0)
Fire, heat, and hot substances	2 694 (2 127 to 3 378)	2 046 (1 629 to 2 578)	118.1 (92.2 to 137.3)	68.1 (54.2 to 86.0)
Poisonings	1 002 (744 to 1 327)	819 (596 to 1 119)	37.8 (28.7 to 48.6)	27.5 (19.9 to 37.5)
Poisoning by carbon monoxide	196 (132 to 295)	129 (77 to 201)	8.2 (5.8 to 11.5)	4.3 (2.6 to 6.7)
Poisoning by other means	806 (582 to 1 087)	691 (490 to 969)	29.6 (22.0 to 39.3)	23.2 (16.4 to 32.5)
Exposure to mechanical forces	16 572 (14 184 to 19 379)	21 332 (18 333 to 24 586)	865.9 (750.0 to 1 003.5)	693.2 (597.0 to 800.0)
Unintentional firearm injuries	1 212 (903 to 1 593)	744 (546 to 992)	62.3 (46.6 to 81.2)	24.1 (17.7 to 32.1)
Other exposure to mechanical forces	15 360 (13 113 to 17 990)	20 588 (17 718 to 23 752)	803.7 (695.0 to 929.1)	669.1 (576.2 to 769.4)
Adverse effects of medical treatment	1 416 (1 207 to 1 638)	3 249 (2 747 to 3 746)	88.4 (76.3 to 101.1)	107.4 (90.8 to 125.5)
Animal contact	6 200 (5 268 to 7 338)	8 123 (7 126 to 9 333)	322.4 (280.5 to 369.2)	269.0 (235.5 to 308.4)
Venomous animal contact	2 199 (1 728 to 2 781)	3 044 (2 426 to 3 744)	114.5 (90.7 to 141.8)	100.6 (80.4 to 123.0)
Non-venomous animal contact	4 001 (3 327 to 4 836)	5 079 (4 272 to 6 058)	207.8 (176.7 to 246.9)	168.5 (141.4 to 200.7)
Foreign body	7 220 (3 819 to 11 775)	12 151 (4 959 to 20 945)	457.1 (208.9 to 782.4)	403.5 (164.0 to 687.7)
Pulmonary aspiration and foreign body in airway	278 (192 to 372)	118 (89 to 157)	10.8 (7.8 to 14.0)	4.0 (3.0 to 5.3)
Foreign body in eyes	6 368 (2 965 to 10 926)	11 645 (4 415 to 20 509)	420.7 (169.3 to 745.2)	385.6 (145.7 to 672.2)
Foreign body in other body part	574 (397 to 847)	388 (282 to 514)	25.6 (18.3 to 35.8)	13.0 (9.4 to 17.0)
Environmental heat and cold exposure	1 028 (759 to 1 372)	467 (348 to 668)	54.0 (42.3 to 67.4)	15.8 (11.8 to 22.3)
Exposure to forces of nature	384 (291 to 499)	33 (28 to 40)	17.5 (13.9 to 21.7)	1.1 (0.9 to 1.3)
Other unintentional injuries	3 611 (3 019 to 4 343)	4 763 (4 078 to 5 516)	190.0 (163.0 to 220.1)	156.3 (134.1 to 180.4)

Prevalence

Cause	All-age counts (95% UI) 1990	All-age counts (95% UI) 2017	Age-standardised rates per 100,000 (95% UI) 1990	Age-standardised rates per 100,000 (95% UI) 2017
**Transport and unintentional injuries**	**151 228 (141 971 to 162 120)**	**308 312 (288 085 to 335 176)**	**10 564.6 (9 950.1 to 11 367.2)**	**10 289.8 (9 611.8 to 11 185.1)**
**Transport injuries**	**14 921 (13 898 to 15 866)**	**43 697 (40 494 to 47 247)**	**1 073.3 (1 000.6 to 1 144.8)**	**1 442.8 (1 336.0 to 1 559.2)**
Road injuries	13 660 (12 704 to 14 610)	42 626 (39 436 to 46 128)	993.6 (924.5 to 1 064.8)	1 407.2 (1 303.1 to 1 522.1)
Pedestrian road injuries	3 341 (2 867 to 3 942)	7 220 (6 295 to 8 371)	244.8 (212.5 to 286.9)	240.4 (209.8 to 278.1)
Cyclist road injuries	1 587 (1 324 to 1 881)	5 100 (4 305 to 5 999)	108.6 (91.1 to 126.4)	167.9 (142.3 to 197.1)
Motorcyclist road injuries	2 691 (2 328 to 3 110)	5 883 (5 018 to 6 936)	193.3 (167.3 to 223.7)	192.6 (164.6 to 226.9)
Motor vehicle road injuries	4 106 (3 579 to 4 756)	18 684 (16 338 to 21 811)	308.9 (268.4 to 358.8)	616.3 (538.2 to 718.5)
Other road injuries	1 935 (1 610 to 2 320)	5 739 (4 777 to 6 971)	138.0 (115.3 to 164.9)	189.9 (158.3 to 230.3)
Other transport injuries	1 260 (1 029 to 1 532)	1 071 (924 to 1 220)	79.7 (66.3 to 94.0)	35.6 (30.9 to 40.5)
**Unintentional injuries**	**136 308 (127 035 to 147 221)**	**264 615 (244 250 to 291 526)**	**9 491.3 (8 876.0 to 10 274.8)**	**8 847.0 (8 167.7 to 9 743.9)**
Falls	59 290 (52 546 to 67 590)	158 909 (141 693 to 181 255)	4 346.4 (3 889.2 to 4 957.9)	5 362.3 (4 790.7 to 6 122.0)
Drowning	609 (532 to 702)	450 (397 to 522)	44.1 (39.0 to 50.3)	15.1 (13.3 to 17.4)
Fire, heat, and hot substances	24 760 (20 596 to 30 957)	25 028 (20 577 to 31 859)	1 524.7 (1 283.8 to 1 852.6)	819.8 (675.4 to 1 041.1)
Poisonings	814 (630 to 1 009)	977 (718 to 1 227)	44.3 (34.9 to 54.3)	31.8 (23.3 to 39.9)
Poisoning by carbon monoxide	127 (98 to 165)	100 (77 to 129)	7.2 (5.6 to 9.1)	3.3 (2.5 to 4.2)
Poisoning by other means	687 (502 to 870)	877 (618 to 1 120)	37.2 (27.4 to 46.8)	28.5 (20.1 to 36.4)
Exposure to mechanical forces	31 962 (27 947 to 36 760)	53 095 (46 039 to 61 676)	2 270.0 (2 000.4 to 2 591.9)	1 749.5 (1 517.5 to 2 029.0)
Unintentional firearm injuries	4 428 (3 538 to 5 339)	3 967 (3 170 to 4 849)	320.1 (258.6 to 388.2)	131.0 (105.0 to 159.9)
Other exposure to mechanical forces	27 533 (23 278 to 32 571)	49 127 (41 979 to 57 905)	1 949.9 (1 666.9 to 2 286.5)	1 618.4 (1 386.9 to 1 904.5)
Adverse effects of medical treatment	108 (85 to 134)	248 (190 to 308)	6.8 (5.2 to 8.3)	8.2 (6.3 to 10.2)
Animal contact	6 268 (5 393 to 7 246)	10 044 (8 804 to 11 424)	423.9 (373.3 to 479.9)	333.9 (293.1 to 379.1)
Venomous animal contact	1 386 (1 142 to 1 642)	2 266 (1 894 to 2 630)	88.1 (73.8 to 102.5)	74.9 (62.6 to 87.1)
Non-venomous animal contact	4 882 (4 044 to 5 833)	7 778 (6 606 to 9 142)	335.8 (282.9 to 393.3)	259.0 (220.4 to 304.2)
Foreign body	3 166 (2 434 to 4 260)	4 194 (2 930 to 5 903)	210.2 (162.7 to 278.8)	138.8 (98.7 to 197.8)
Pulmonary aspiration and foreign body in airway	176 (125 to 241)	101 (82 to 125)	9.8 (7.2 to 13.1)	3.3 (2.7 to 4.1)
Foreign body in eyes	1 278 (651 to 2 238)	2 593 (1 434 to 4 285)	95.6 (53.4 to 158.9)	87.9 (49.3 to 144.2)
Foreign body in other body part	1 711 (1 366 to 2 174)	1 440 (1 199 to 1 786)	104.8 (85.2 to 130.6)	47.6 (39.6 to 58.8)
Environmental heat and cold exposure	3 454 (2 814 to 4 179)	1 769 (1 504 to 2 122)	229.5 (192.6 to 272.1)	59.3 (50.5 to 71.2)
Exposure to forces of nature	799 (579 to 1 098)	1 615 (1 138 to 2 239)	43.0 (31.6 to 58.8)	52.2 (36.9 to 72.3)
Other unintentional injuries	5 077 (4 390 to 5 870)	8 363 (7 270 to 9 544)	348.5 (301.8 to 398.0)	276.6 (240.9 to 314.9)

Years lived with disability

Cause	All-age counts (95% UI) 1990	All-age counts (95% UI) 2017	Age-standardised rates per 100,000 (95% UI) 1990	Age-standardised rates per 100,000 (95% UI) 2017
**Transport and unintentional injuries**	**6 813 (4 977 to 8 967)**	**13 670 (9 894 to 18 230)**	**468.9 (342.6 to 617.9)**	**456.3 (329.9 to 609.2)**
**Transport injuries**	**1 032 (754 to 1 354)**	**2 692 (1 954 to 3 611)**	**71.7 (52.3 to 94.1)**	**88.4 (64.3 to 118.5)**
Road injuries	949 (692 to 1 248)	2 634 (1 914 to 3 528)	66.7 (48.8 to 87.7)	86.5 (62.9 to 115.8)
Pedestrian road injuries	242 (172 to 328)	464 (328 to 639)	17.1 (12.2 to 23.0)	15.4 (10.9 to 21.1)
Cyclist road injuries	112 (79 to 148)	312 (218 to 416)	7.4 (5.2 to 9.8)	10.2 (7.2 to 13.6)
Motorcyclist road injuries	188 (133 to 253)	365 (249 to 499)	13.1 (9.2 to 17.7)	11.9 (8.1 to 16.3)
Motor vehicle road injuries	296 (214 to 393)	1 199 (866 to 1 604)	21.5 (15.5 to 28.3)	39.3 (28.4 to 52.5)
Other road injuries	111 (80 to 149)	294 (210 to 407)	7.6 (5.6 to 10.3)	9.7 (6.9 to 13.4)
Other transport injuries	83 (58 to 112)	58 (42 to 79)	5.0 (3.6 to 6.7)	1.9 (1.4 to 2.6)
**Unintentional injuries**	**5 781 (4 211 to 7 646)**	**10 978 (7 875 to 14 876)**	**397.1 (286.4 to 525.0)**	**367.9 (263.5 to 497.4)**
Falls	3 582 (2 629 to 4 659)	8 270 (5 981 to 11 023)	255.3 (185.4 to 332.9)	278.7 (200.8 to 370.7)
Drowning	35 (24 to 48)	24 (17 to 33)	2.4 (1.7 to 3.3)	0.8 (0.6 to 1.1)
Fire, heat, and hot substances	541 (338 to 842)	489 (291 to 794)	32.2 (20.0 to 49.7)	16.0 (9.5 to 26.0)
Poisonings	97 (60 to 141)	114 (68 to 174)	5.1 (3.2 to 7.4)	3.7 (2.2 to 5.7)
Poisoning by carbon monoxide	14 (8 to 22)	10 (6 to 16)	0.7 (0.5 to 1.1)	0.3 (0.2 to 0.5)
Poisoning by other means	83 (50 to 122)	104 (62 to 163)	4.4 (2.7 to 6.4)	3.4 (2.0 to 5.3)
Exposure to mechanical forces	761 (524 to 1 087)	1 167 (792 to 1 686)	52.9 (36.4 to 76.0)	38.4 (26.0 to 55.5)
Unintentional firearm injuries	92 (61 to 140)	72 (46 to 114)	6.4 (4.3 to 9.9)	2.4 (1.5 to 3.8)
Other exposure to mechanical forces	669 (459 to 953)	1 095 (738 to 1 590)	46.5 (32.0 to 66.6)	36.0 (24.4 to 52.3)
Adverse effects of medical treatment	14 (9 to 22)	33 (21 to 50)	0.9 (0.6 to 1.4)	1.1 (0.7 to 1.6)
Animal contact	165 (111 to 231)	225 (150 to 322)	10.1 (6.8 to 14.4)	7.4 (4.9 to 10.6)
Venomous animal contact	88 (57 to 128)	115 (75 to 169)	4.9 (3.2 to 7.1)	3.7 (2.5 to 5.3)
Non-venomous animal contact	76 (46 to 121)	111 (66 to 179)	5.2 (3.2 to 8.1)	3.7 (2.2 to 6.0)
Foreign body	202 (133 to 298)	248 (148 to 403)	13.1 (8.7 to 19.5)	8.3 (5.0 to 13.6)
Pulmonary aspiration and foreign body in airway	10 (6 to 13)	4 (3 to 6)	0.5 (0.3 to 0.7)	0.1 (0.1 to 0.2)
Foreign body in eyes	85 (43 to 156)	178 (89 to 311)	6.4 (3.5 to 11.5)	5.9 (3.1 to 10.6)
Foreign body in other body part	107 (74 to 143)	70 (50 to 92)	6.2 (4.4 to 8.2)	2.3 (1.6 to 3.0)
Environmental heat and cold exposure	138 (99 to 193)	68 (44 to 98)	9.1 (6.1 to 12.6)	2.3 (1.5 to 3.3)
Exposure to forces of nature	44 (32 to 62)	55 (40 to 75)	2.4 (1.7 to 3.2)	1.8 (1.3 to 2.4)
Other unintentional injuries	202 (146 to 268)	286 (201 to 388)	13.5 (9.8 to 18.1)	9.4 (6.6 to 12.8)

Disability adjusted life years

Cause	All-age counts (95% UI) 1990	All-age counts (95% UI) 2017	Age-standardised rates per 100,000 (95% UI) 1990	Age-standardised rates per 100,000 (95% UI) 2017
**Transport and unintentional injuries**	**69 750 (64 901 to 75 182)**	**60 215 (55 152 to 65 888)**	**3 591.5 (3 380.5 to 3 835.2)**	**1 970.7 (1 806.0 to 2 158.6)**
**Transport injuries**	**27 266 (25 503 to 29 355)**	**27 137 (25 042 to 29 403)**	**1 405.5 (1 329.3 to 1 490.9)**	**874.3 (807.3 to 947.4)**
Road injuries	26 690 (24 964 to 28 698)	26 463 (24 429 to 28 676)	1 375.1 (1 300.1 to 1 459.8)	852.6 (787.1 to 923.8)
Pedestrian road injuries	11 742 (10 688 to 12 984)	8 582 (7 781 to 9 522)	597.8 (553.2 to 649.2)	278.8 (253.4 to 309.1)
Cyclist road injuries	678 (610 to 752)	1 086 (952 to 1 232)	37.2 (33.7 to 41.1)	35.2 (30.9 to 40.0)
Motorcyclist road injuries	3 963 (3 557 to 4 407)	3 516 (3 086 to 4 018)	205.4 (186.8 to 225.8)	112.2 (98.5 to 128.1)
Motor vehicle road injuries	9 522 (8 687 to 10 538)	12 482 (11 296 to 13 709)	492.9 (454.5 to 541.2)	400.5 (362.6 to 439.6)
Other road injuries	786 (648 to 912)	796 (676 to 930)	41.8 (34.9 to 47.7)	25.8 (22.0 to 30.2)
Other transport injuries	576 (513 to 647)	674 (593 to 759)	30.3 (27.1 to 33.7)	21.7 (19.1 to 24.5)
**Unintentional injuries**	**42 483 (38 769 to 46 491)**	**33 078 (29 406 to 37 235)**	**2 186.1 (2 032.3 to 2 355.9)**	**1 096.4 (973.0 to 1 234.2)**
Falls	8 883 (7 853 to 10 085)	13 198 (10 821 to 15 920)	595.0 (524.1 to 672.8)	443.7 (363.3 to 535.2)
Drowning	9 885 (8 844 to 11 122)	4 648 (4 198 to 5 146)	451.5 (412.6 to 496.2)	151.0 (136.1 to 167.4)
Fire, heat, and hot substances	2 989 (2 566 to 3 479)	1 446 (1 227 to 1 759)	145.7 (128.1 to 167.3)	47.8 (40.6 to 58.0)
Poisonings	2 222 (1 961 to 2 526)	1 042 (944 to 1 150)	98.9 (89.7 to 109.5)	34.0 (30.8 to 37.5)
Poisoning by carbon monoxide	922 (808 to 1 059)	465 (415 to 520)	42.0 (37.7 to 47.1)	15.1 (13.5 to 16.9)
Poisoning by other means	1 300 (1 131 to 1 498)	577 (513 to 651)	56.9 (51.1 to 64.0)	18.9 (16.7 to 21.3)
Exposure to mechanical forces	5 650 (5 187 to 6 160)	3 412 (2 986 to 3 991)	308.0 (283.6 to 336.7)	110.8 (96.9 to 129.6)
Unintentional firearm injuries	2 413 (2 156 to 2 691)	903 (751 to 1 045)	125.8 (113.4 to 139.8)	29.0 (24.1 to 33.2)
Other exposure to mechanical forces	3 237 (2 877 to 3 644)	2 509 (2 217 to 3 036)	182.2 (162.5 to 206.8)	81.8 (68.9 to 99.1)
Adverse effects of medical treatment	1 401 (1 145 to 1 630)	1 128 (986 to 1 597)	69.6 (58.7 to 78.2)	37.9 (33.1 to 53.5)
Animal contact	1 226 (1 043 to 1 433)	585 (499 to 693)	55.3 (48.6 to 63.1)	19.4 (16.5 to 22.9)
Venomous animal contact	858 (710 to 1 025)	329 (273 to 388)	36.5 (31.2 to 42.4)	10.9 (9.1 to 12.9)
Non-venomous animal contact	367 (293 to 442)	257 (205 to 335)	18.8 (15.2 to 22.5)	8.5 (6.8 to 11.1)
Foreign body	7 147 (6 166 to 8 324)	5 871 (5 071 to 6 802)	300.4 (267.0 to 339.4)	195.3 (168.3 to 226.9)
Pulmonary aspiration and foreign body in airway	6 626 (5 697 to 7 764)	5 503 (4 724 to 6 423)	273.4 (242.2 to 311.4)	183.1 (156.8 to 214.3)
Foreign body in eyes	85 (43 to 156)	178 (89 to 311)	6.4 (3.5 to 11.5)	5.9 (3.1 to 10.6)
Foreign body in other body part	436 (344 to 510)	195 (163 to 283)	20.6 (17.0 to 23.8)	6.3 (5.3 to 9.2)
Environmental heat and cold exposure	823 (739 to 913)	319 (284 to 351)	46.1 (41.7 to 50.6)	10.5 (9.3 to 11.8)
Exposure to forces of nature	282 (232 to 346)	80 (65 to 100)	13.9 (11.4 to 17.0)	2.6 (2.1 to 3.2)
Other unintentional injuries	1 979 (1 810 to 2 161)	1 349 (1 208 to 1 513)	101.6 (93.4 to 110.8)	43.5 (38.9 to 48.8)

Death, YLL, incidence, prevalence, YLD, and DALY counts and rates per 100,000 for all injuries in Jalisco in 1990 and 2017

Deaths

Cause	All-age counts (95% UI) 1990	All-age counts (95% UI) 2017	Age-standardised rates per 100,000 (95% UI) 1990	Age-standardised rates per 100,000 (95% UI) 2017
**Transport and unintentional injuries**	**3 417 (3 245 to 3 597)**	**2 972 (2 784 to 3 174)**	**74.4 (71.9 to 76.9)**	**37.7 (35.4 to 40.2)**
**Transport injuries**	**1 593 (1 507 to 1 675)**	**1 630 (1 509 to 1 762)**	**33.0 (31.6 to 34.3)**	**19.9 (18.5 to 21.5)**
Road injuries	1 548 (1 464 to 1 628)	1 553 (1 436 to 1 679)	32.0 (30.7 to 33.3)	19.0 (17.6 to 20.5)
Pedestrian road injuries	768 (712 to 826)	783 (720 to 851)	16.8 (15.9 to 17.7)	9.8 (9.1 to 10.7)
Cyclist road injuries	34 (31 to 38)	59 (53 to 67)	0.7 (0.7 to 0.8)	0.7 (0.7 to 0.8)
Motorcyclist road injuries	239 (215 to 263)	177 (155 to 200)	4.5 (4.1 to 4.9)	2.1 (1.8 to 2.3)
Motor vehicle road injuries	450 (416 to 486)	480 (432 to 527)	8.8 (8.3 to 9.5)	5.7 (5.1 to 6.3)
Other road injuries	57 (48 to 65)	54 (48 to 62)	1.2 (1.0 to 1.3)	0.7 (0.6 to 0.7)
Other transport injuries	45 (42 to 49)	77 (67 to 88)	1.0 (0.9 to 1.1)	0.9 (0.8 to 1.1)
**Unintentional injuries**	**1 823 (1 712 to 1 946)**	**1 342 (1 258 to 1 422)**	**41.4 (39.7 to 43.1)**	**17.8 (16.6 to 18.8)**
Falls	416 (396 to 437)	432 (391 to 464)	13.1 (12.5 to 13.8)	6.1 (5.5 to 6.5)
Drowning	407 (373 to 454)	211 (195 to 230)	7.3 (6.8 to 8.0)	2.6 (2.4 to 2.8)
Fire, heat, and hot substances	133 (121 to 145)	90 (83 to 97)	3.2 (3.0 to 3.4)	1.2 (1.1 to 1.3)
Poisonings	64 (58 to 70)	39 (36 to 43)	1.2 (1.1 to 1.3)	0.5 (0.4 to 0.5)
Poisoning by carbon monoxide	22 (19 to 24)	16 (14 to 17)	0.4 (0.4 to 0.5)	0.2 (0.2 to 0.2)
Poisoning by other means	42 (38 to 47)	24 (22 to 26)	0.8 (0.7 to 0.9)	0.3 (0.3 to 0.3)
Exposure to mechanical forces	240 (227 to 254)	116 (106 to 125)	5.0 (4.8 to 5.3)	1.4 (1.3 to 1.6)
Unintentional firearm injuries	134 (123 to 148)	45 (34 to 53)	2.7 (2.5 to 3.0)	0.6 (0.4 to 0.6)
Other exposure to mechanical forces	106 (98 to 115)	71 (64 to 83)	2.4 (2.1 to 2.5)	0.9 (0.8 to 1.0)
Adverse effects of medical treatment	105 (84 to 114)	130 (118 to 148)	2.9 (2.3 to 3.1)	1.8 (1.6 to 2.1)
Animal contact	81 (69 to 97)	35 (32 to 38)	1.5 (1.3 to 1.7)	0.5 (0.4 to 0.5)
Venomous animal contact	68 (56 to 83)	24 (21 to 27)	1.2 (1.0 to 1.4)	0.3 (0.3 to 0.4)
Non-venomous animal contact	13 (10 to 17)	11 (9 to 13)	0.3 (0.2 to 0.3)	0.1 (0.1 to 0.2)
Foreign body	270 (237 to 309)	212 (192 to 233)	4.9 (4.5 to 5.4)	2.7 (2.5 to 3.1)
Pulmonary aspiration and foreign body in airway	260 (227 to 297)	207 (188 to 233)	4.7 (4.3 to 5.2)	2.7 (2.4 to 3.0)
Foreign body in eyes				
Foreign body in other body part	11 (8 to 13)	5 (4 to 8)	0.2 (0.2 to 0.2)	0.1 (0.1 to 0.1)
Environmental heat and cold exposure	26 (24 to 28)	14 (12 to 15)	0.7 (0.7 to 0.7)	0.2 (0.2 to 0.2)
Exposure to forces of nature	1 (1 to 1)	1 (1 to 1)	0.0 (0.0 to 0.0)	0.0 (0.0 to 0.0)
Other unintentional injuries	82 (76 to 87)	61 (54 to 68)	1.6 (1.5 to 1.7)	0.7 (0.7 to 0.8)

Years of life lost

Cause	All-age counts (95% UI) 1990	All-age counts (95% UI) 2017	Age-standardised rates per 100,000 (95% UI) 1990	Age-standardised rates per 100,000 (95% UI) 2017
**Transport and unintentional injuries**	**186 282 (172 643 to 201 471)**	**129 215 (119 738 to 139 153)**	**3 271.0 (3 087.8 to 3 473.1)**	**1 556.2 (1 444.8 to 1 676.6)**
**Transport injuries**	**88 887 (82 585 to 94 933)**	**77 689 (71 316 to 84 164)**	**1 584.0 (1 494.5 to 1 669.9)**	**919.9 (846.4 to 996.8)**
Road injuries	86 446 (80 296 to 92 379)	74 015 (68 097 to 80 220)	1 539.2 (1 451.2 to 1 623.9)	876.6 (807.7 to 949.1)
Pedestrian road injuries	41 265 (37 182 to 45 686)	33 929 (30 994 to 37 057)	734.4 (676.6 to 797.1)	408.4 (373.9 to 445.4)
Cyclist road injuries	1 915 (1 707 to 2 132)	2 681 (2 393 to 3 022)	35.3 (32.0 to 39.0)	31.9 (28.5 to 36.0)
Motorcyclist road injuries	14 234 (12 699 to 15 752)	10 003 (8 695 to 11 454)	248.9 (225.3 to 274.0)	115.5 (100.7 to 132.0)
Motor vehicle road injuries	25 841 (23 654 to 28 129)	24 835 (22 253 to 27 458)	463.1 (428.5 to 500.7)	290.5 (260.3 to 320.4)
Other road injuries	3 191 (2 675 to 3 716)	2 567 (2 231 to 2 968)	56.4 (47.8 to 65.0)	30.3 (26.4 to 34.9)
Other transport injuries	2 441 (2 201 to 2 705)	3 674 (3 152 to 4 208)	44.8 (40.8 to 49.0)	43.2 (37.2 to 49.5)
**Unintentional injuries**	**97 396 (88 417 to 107 775)**	**51 526 (47 560 to 55 929)**	**1 687.0 (1 570.6 to 1 822.3)**	**636.4 (588.5 to 692.8)**
Falls	12 818 (12 023 to 13 687)	10 659 (9 628 to 11 556)	297.2 (282.2 to 312.0)	135.6 (122.8 to 146.6)
Drowning	26 178 (23 196 to 30 128)	10 982 (9 982 to 12 111)	414.8 (375.7 to 466.6)	132.1 (120.1 to 146.0)
Fire, heat, and hot substances	6 844 (5 969 to 7 821)	2 957 (2 687 to 3 282)	118.8 (107.0 to 132.1)	36.9 (33.5 to 40.3)
Poisonings	3 965 (3 510 to 4 487)	1 847 (1 665 to 2 037)	64.4 (58.5 to 71.3)	22.3 (20.1 to 24.6)
Poisoning by carbon monoxide	1 297 (1 144 to 1 470)	723 (640 to 806)	21.6 (19.4 to 24.0)	8.7 (7.7 to 9.7)
Poisoning by other means	2 668 (2 333 to 3 053)	1 123 (1 005 to 1 253)	42.8 (38.4 to 48.0)	13.7 (12.2 to 15.2)
Exposure to mechanical forces	13 187 (12 285 to 14 130)	4 966 (4 549 to 5 454)	241.3 (227.3 to 255.8)	59.4 (54.5 to 65.1)
Unintentional firearm injuries	7 624 (6 930 to 8 429)	2 079 (1 536 to 2 379)	136.8 (125.7 to 151.6)	24.5 (18.2 to 28.0)
Other exposure to mechanical forces	5 563 (4 784 to 6 149)	2 887 (2 577 to 3 392)	104.5 (90.4 to 114.0)	34.9 (31.2 to 40.8)
Adverse effects of medical treatment	4 438 (3 388 to 5 112)	3 808 (3 420 to 4 441)	85.0 (67.0 to 94.7)	48.8 (43.8 to 57.0)
Animal contact	5 639 (4 540 to 6 957)	1 421 (1 275 to 1 598)	82.9 (69.2 to 100.1)	17.8 (16.0 to 20.1)
Venomous animal contact	4 890 (3 841 to 6 186)	1 007 (880 to 1 154)	70.4 (56.8 to 87.4)	12.7 (11.1 to 14.5)
Non-venomous animal contact	729 (540 to 1 027)	414 (358 to 530)	12.5 (9.6 to 16.2)	5.1 (4.4 to 6.5)
Foreign body	18 389 (15 505 to 21 600)	11 276 (9 807 to 13 396)	275.8 (238.9 to 317.8)	140.9 (122.0 to 168.3)
Pulmonary aspiration and foreign body in airway	17 680 (14 890 to 20 862)	10 987 (9 546 to 13 051)	264.3 (228.7 to 305.0)	137.5 (118.8 to 164.1)
Foreign body in eyes				
Foreign body in other body part	710 (540 to 850)	289 (234 to 452)	11.5 (8.9 to 13.4)	3.4 (2.8 to 5.4)
Environmental heat and cold exposure	1 150 (1 042 to 1 265)	492 (432 to 544)	23.3 (21.6 to 25.1)	6.0 (5.3 to 6.6)
Exposure to forces of nature	87 (55 to 129)	67 (61 to 74)	1.1 (0.7 to 1.6)	0.8 (0.7 to 0.9)
Other unintentional injuries	4 721 (4 353 to 5 104)	3 050 (2 723 to 3 416)	82.4 (76.5 to 88.1)	35.7 (31.9 to 39.9)

Incidence

Cause	All-age counts (95% UI) 1990	All-age counts (95% UI) 2017	Age-standardised rates per 100,000 (95% UI) 1990	Age-standardised rates per 100,000 (95% UI) 2017
**Transport and unintentional injuries**	**215 864 (198 498 to 235 209)**	**321 102 (290 923 to 352 442)**	**4 463.8 (4 072.9 to 4 863.4)**	**4 047.9 (3 672.0 to 4 437.0)**
**Transport injuries**	**18 490 (15 693 to 21 627)**	**37 365 (32 183 to 43 210)**	**366.2 (316.4 to 418.2)**	**449.4 (387.8 to 518.0)**
Road injuries	17 516 (14 827 to 20 610)	36 333 (31 214 to 42 225)	348.1 (297.8 to 399.0)	436.5 (374.3 to 505.3)
Pedestrian road injuries	3 240 (2 579 to 3 998)	5 095 (4 045 to 6 185)	66.0 (54.4 to 79.7)	61.5 (49.8 to 75.2)
Cyclist road injuries	2 392 (1 768 to 3 208)	4 673 (3 618 to 5 948)	44.6 (34.3 to 57.4)	57.0 (44.2 to 72.3)
Motorcyclist road injuries	2 472 (1 925 to 3 131)	4 114 (3 122 to 5 228)	47.1 (37.7 to 58.9)	48.5 (37.0 to 61.4)
Motor vehicle road injuries	5 754 (4 614 to 7 049)	17 038 (14 014 to 20 365)	118.1 (95.5 to 142.4)	202.8 (166.8 to 240.9)
Other road injuries	3 657 (2 756 to 4 657)	5 475 (4 313 to 6 808)	72.3 (56.4 to 88.4)	66.6 (52.6 to 82.4)
Other transport injuries	974 (719 to 1 355)	1 032 (745 to 1 401)	18.1 (14.3 to 23.3)	12.8 (9.2 to 17.4)
**Unintentional injuries**	**197 364 (180 437 to 216 369)**	**283 737 (255 255 to 315 486)**	**4 097.6 (3 713.2 to 4 496.5)**	**3 598.5 (3 235.3 to 4 002.3)**
Falls	87 944 (77 294 to 99 126)	166 760 (148 258 to 188 382)	1 988.5 (1 769.0 to 2 217.6)	2 163.5 (1 925.6 to 2 430.8)
Drowning	316 (273 to 367)	206 (170 to 246)	5.7 (5.0 to 6.5)	2.5 (2.1 to 3.0)
Fire, heat, and hot substances	7 376 (5 825 to 9 279)	5 055 (4 050 to 6 324)	115.4 (93.7 to 141.1)	62.5 (50.3 to 79.0)
Poisonings	2 243 (1 640 to 3 108)	1 869 (1 336 to 2 534)	32.0 (24.1 to 43.2)	23.5 (16.8 to 32.1)
Poisoning by carbon monoxide	443 (273 to 682)	380 (198 to 621)	7.0 (4.7 to 10.3)	4.7 (2.5 to 7.7)
Poisoning by other means	1 799 (1 286 to 2 559)	1 489 (1 014 to 2 111)	25.1 (18.2 to 35.1)	18.8 (12.8 to 26.6)
Exposure to mechanical forces	44 213 (37 502 to 52 694)	44 942 (38 201 to 53 072)	821.5 (703.5 to 961.9)	538.5 (456.9 to 634.5)
Unintentional firearm injuries	3 952 (2 902 to 5 245)	2 035 (1 462 to 2 774)	72.2 (54.0 to 93.8)	24.2 (17.6 to 32.9)
Other exposure to mechanical forces	40 262 (34 093 to 47 927)	42 907 (36 454 to 50 802)	749.3 (644.0 to 880.0)	514.2 (437.1 to 606.9)
Adverse effects of medical treatment	5 527 (4 726 to 6 329)	11 377 (9 759 to 13 017)	123.6 (107.1 to 140.7)	141.6 (121.6 to 162.0)
Animal contact	17 292 (14 316 to 21 051)	11 515 (9 400 to 14 050)	321.6 (274.2 to 375.9)	144.1 (118.0 to 174.6)
Venomous animal contact	6 280 (4 768 to 8 144)	4 396 (3 275 to 5 701)	116.4 (90.2 to 147.0)	54.8 (40.8 to 71.0)
Non-venomous animal contact	11 012 (8 880 to 13 665)	7 119 (5 592 to 9 011)	205.2 (168.9 to 248.5)	89.3 (70.4 to 113.5)
Foreign body	20 264 (10 947 to 31 773)	31 856 (18 854 to 55 573)	454.9 (206.0 to 746.4)	397.5 (170.6 to 696.7)
Pulmonary aspiration and foreign body in airway	664 (471 to 913)	335 (245 to 447)	9.8 (7.2 to 13.0)	4.3 (3.1 to 5.7)
Foreign body in eyes	18 047 (8 684 to 29 528)	30 540 (12 411 to 54 262)	419.5 (169.4 to 713.4)	383.1 (154.8 to 681.5)
Foreign body in other body part	1 552 (1 093 to 2 365)	981 (718 to 1 295)	25.6 (17.7 to 36.7)	12.2 (8.9 to 16.0)
Environmental heat and cold exposure	2 394 (1 702 to 3 257)	1 068 (808 to 1 359)	45.9 (35.1 to 58.1)	13.7 (10.4 to 19.8)
Exposure to forces of nature	25 (17 to 38)	90 (76 to 108)	0.3 (0.2 to 0.5)	1.1 (0.9 to 1.3)
Other unintentional injuries	9 769 (7 958 to 12 022)	9 000 (7 275 to 11 392)	188.2 (156.7 to 224.8)	110.1 (89.2 to 139.2)

Prevalence

Cause	All-age counts (95% UI) 1990	All-age counts (95% UI) 2017	Age-standardised rates per 100,000 (95% UI) 1990	Age-standardised rates per 100,000 (95% UI) 2017
**Transport and unintentional injuries**	**408 529 (383 681 to 438 081)**	**656 651 (613 336 to 712 564)**	**9 992.0 (9 422.1 to 10 720.0)**	**8 286.1 (7 739.7 to 8 975.1)**
**Transport injuries**	**45 262 (42 173 to 48 235)**	**106 230 (98 919 to 113 631)**	**1 133.7 (1 060.5 to 1 208.7)**	**1 319.9 (1 229.1 to 1 412.8)**
Road injuries	40 846 (37 994 to 43 742)	100 838 (93 670 to 108 000)	1 032.1 (960.9 to 1 104.8)	1 252.5 (1 164.4 to 1 343.1)
Pedestrian road injuries	11 650 (9 929 to 13 785)	22 346 (19 360 to 26 098)	293.4 (253.7 to 346.9)	281.2 (244.6 to 326.8)
Cyclist road injuries	3 894 (3 267 to 4 573)	9 940 (8 399 to 11 783)	94.2 (80.0 to 108.8)	123.3 (104.6 to 144.3)
Motorcyclist road injuries	8 708 (7 553 to 10 091)	18 572 (15 851 to 21 894)	217.6 (188.8 to 251.2)	226.7 (193.6 to 266.7)
Motor vehicle road injuries	10 060 (8 761 to 11 685)	37 450 (32 550 to 43 708)	264.1 (229.2 to 308.0)	465.2 (404.2 to 542.4)
Other road injuries	6 534 (5 450 to 7 859)	12 530 (10 465 to 15 175)	162.7 (136.6 to 195.5)	156.3 (131.1 to 189.1)
Other transport injuries	4 416 (3 665 to 5 265)	5 393 (4 577 to 6 240)	101.6 (85.8 to 118.1)	67.4 (57.4 to 77.7)
**Unintentional injuries**	**363 266 (338 613 to 392 890)**	**550 420 (507 351 to 605 568)**	**8 858.3 (8 282.2 to 9 565.0)**	**6 966.2 (6 434.2 to 7 661.9)**
Falls	151 775 (134 048 to 172 770)	327 794 (291 034 to 374 874)	3 920.7 (3 511.4 to 4 471.8)	4 215.3 (3 759.1 to 4 813.7)
Drowning	1 481 (1 251 to 1 650)	1 239 (1 096 to 1 434)	35.4 (31.1 to 40.6)	15.7 (13.9 to 18.1)
Fire, heat, and hot substances	70 239 (58 071 to 88 892)	60 358 (49 802 to 75 079)	1 521.8 (1 274.5 to 1 873.0)	789.5 (631.1 to 918.0)
Poisonings	1 952 (1 482 to 2 429)	2 242 (1 634 to 2 811)	37.7 (28.9 to 46.7)	27.0 (19.7 to 33.8)
Poisoning by carbon monoxide	303 (231 to 394)	304 (232 to 392)	6.0 (4.7 to 7.7)	3.7 (2.8 to 4.7)
Poisoning by other means	1 649 (1 183 to 2 118)	1 939 (1 354 to 2 493)	31.7 (23.0 to 40.6)	23.3 (16.3 to 30.0)
Exposure to mechanical forces	86 824 (76 174 to 99 756)	110 395 (96 227 to 128 216)	2 148.9 (1 897.2 to 2 454.9)	1 363.0 (1 189.7 to 1 578.6)
Unintentional firearm injuries	13 869 (11 049 to 16 684)	10 077 (8 195 to 12 186)	350.2 (281.9 to 421.4)	125.0 (101.9 to 150.9)
Other exposure to mechanical forces	72 955 (61 684 to 86 419)	100 318 (85 405 to 118 136)	1 798.6 (1 534.7 to 2 127.7)	1 238.0 (1 058.2 to 1 454.8)
Adverse effects of medical treatment	429 (331 to 527)	871 (671 to 1 080)	9.5 (7.4 to 11.6)	10.8 (8.3 to 13.5)
Animal contact	18 200 (15 494 to 21 252)	14 341 (12 523 to 16 400)	422.5 (367.0 to 483.3)	179.5 (157.1 to 204.7)
Venomous animal contact	4 289 (3 488 to 5 205)	3 386 (2 835 to 3 949)	93.2 (77.3 to 110.7)	41.9 (35.2 to 48.7)
Non-venomous animal contact	13 911 (11 387 to 16 808)	10 956 (9 179 to 12 903)	329.3 (275.4 to 388.1)	137.6 (115.8 to 161.2)
Foreign body	8 994 (6 931 to 11 926)	10 548 (7 488 to 15 173)	209.0 (161.9 to 276.6)	134.3 (95.7 to 193.1)
Pulmonary aspiration and foreign body in airway	461 (326 to 631)	308 (242 to 387)	9.0 (6.5 to 12.1)	3.8 (3.0 to 4.7)
Foreign body in eyes	3 675 (1 925 to 6 253)	6 666 (3 620 to 11 386)	95.9 (54.1 to 156.6)	86.3 (48.0 to 143.9)
Foreign body in other body part	4 859 (3 847 to 6 216)	3 575 (2 997 to 4 265)	104.2 (84.1 to 130.2)	44.3 (37.3 to 52.8)
Environmental heat and cold exposure	8 395 (6 815 to 10 245)	4 179 (3 506 to 5 011)	193.7 (161.8 to 231.6)	52.8 (44.5 to 63.3)
Exposure to forces of nature	1 056 (744 to 1 453)	2 627 (1 834 to 3 690)	20.1 (14.7 to 27.5)	31.5 (22.0 to 43.4)
Other unintentional injuries	13 977 (11 976 to 16 105)	15 825 (13 633 to 18 180)	339.0 (292.6 to 387.6)	196.7 (170.2 to 225.1)

Years lived with disability

Cause	All-age counts (95% UI) 1990	All-age counts (95% UI) 2017	Age-standardised rates per 100,000 (95% UI) 1990	Age-standardised rates per 100,000 (95% UI) 2017
**Transport and unintentional injuries**	**17 854 (12 978 to 23 764)**	**28 792 (20 802 to 38 642)**	**431.9 (312.7 to 573.8)**	**363.3 (262.2 to 486.2)**
**Transport injuries**	**3 065 (2 248 to 4 070)**	**6 426 (4 687 to 8 602)**	**74.3 (54.4 to 98.3)**	**79.1 (57.7 to 105.6)**
Road injuries	2 786 (2 043 to 3 712)	6 140 (4 473 to 8 227)	68.1 (49.8 to 90.8)	75.6 (55.0 to 101.2)
Pedestrian road injuries	835 (583 to 1 141)	1 418 (993 to 1 943)	20.2 (14.4 to 27.2)	17.7 (12.5 to 24.2)
Cyclist road injuries	269 (183 to 353)	596 (418 to 793)	6.3 (4.5 to 8.3)	7.3 (5.1 to 9.7)
Motorcyclist road injuries	602 (422 to 810)	1 138 (778 to 1 554)	14.6 (10.2 to 19.7)	13.8 (9.4 to 18.8)
Motor vehicle road injuries	709 (530 to 940)	2 360 (1 697 to 3 149)	18.0 (13.0 to 23.7)	29.0 (20.9 to 38.7)
Other road injuries	371 (263 to 501)	628 (460 to 853)	8.9 (6.4 to 12.1)	7.8 (5.6 to 10.7)
Other transport injuries	280 (199 to 372)	286 (206 to 384)	6.2 (4.4 to 8.2)	3.5 (2.5 to 4.8)
**Unintentional injuries**	**14 788 (10 676 to 19 734)**	**22 366 (15 978 to 30 182)**	**357.6 (257.8 to 479.3)**	**284.2 (203.0 to 384.0)**
Falls	8 902 (6 565 to 11 689)	16 658 (12 007 to 22 095)	224.3 (165.6 to 294.7)	213.7 (153.5 to 283.5)
Drowning	81 (57 to 111)	65 (45 to 92)	1.9 (1.4 to 2.7)	0.8 (0.6 to 1.2)
Fire, heat, and hot substances	1 502 (943 to 2 369)	1 161 (678 to 1 870)	31.6 (19.6 to 49.3)	14.2 (8.3 to 22.8)
Poisonings	232 (143 to 348)	260 (151 to 398)	4.3 (2.7 to 6.6)	3.1 (1.8 to 4.8)
Poisoning by carbon monoxide	33 (20 to 51)	30 (17 to 49)	0.6 (0.4 to 1.0)	0.4 (0.2 to 0.6)
Poisoning by other means	199 (119 to 304)	230 (132 to 357)	3.7 (2.2 to 5.7)	2.8 (1.6 to 4.3)
Exposure to mechanical forces	2 033 (1 398 to 2 886)	2 376 (1 597 to 3 462)	49.4 (34.2 to 70.5)	29.3 (19.7 to 42.6)
Unintentional firearm injuries	294 (195 to 440)	185 (119 to 291)	7.2 (4.7 to 10.9)	2.3 (1.5 to 3.6)
Other exposure to mechanical forces	1 739 (1 198 to 2 490)	2 191 (1 465 to 3 204)	42.2 (29.0 to 60.6)	27.0 (18.1 to 39.3)
Adverse effects of medical treatment	56 (35 to 85)	116 (72 to 176)	1.3 (0.8 to 1.9)	1.4 (0.9 to 2.2)
Animal contact	493 (323 to 693)	335 (221 to 477)	10.4 (6.9 to 14.8)	4.1 (2.7 to 5.9)
Venomous animal contact	279 (175 to 405)	182 (114 to 265)	5.4 (3.5 to 7.8)	2.2 (1.4 to 3.2)
Non-venomous animal contact	214 (129 to 339)	154 (90 to 249)	5.0 (3.1 to 7.9)	1.9 (1.1 to 3.1)
Foreign body	562 (367 to 840)	629 (368 to 1 028)	12.9 (8.5 to 19.2)	7.9 (4.7 to 13.0)
Pulmonary aspiration and foreign body in airway	25 (16 to 34)	13 (9 to 18)	0.5 (0.3 to 0.6)	0.2 (0.1 to 0.2)
Foreign body in eyes	244 (124 to 437)	444 (228 to 817)	6.4 (3.4 to 11.3)	5.7 (3.0 to 10.4)
Foreign body in other body part	293 (203 to 392)	166 (119 to 216)	6.0 (4.2 to 7.9)	2.0 (1.5 to 2.7)
Environmental heat and cold exposure	328 (216 to 468)	158 (104 to 227)	7.5 (5.0 to 10.6)	2.0 (1.3 to 2.9)
Exposure to forces of nature	54 (38 to 75)	87 (64 to 121)	1.0 (0.7 to 1.4)	1.0 (0.8 to 1.4)
Other unintentional injuries	544 (383 to 729)	526 (372 to 724)	12.9 (9.2 to 17.4)	6.5 (4.6 to 9.0)

Disability-adjusted life-years

Cause	All-age counts (95% UI) 1990	All-age counts (95% UI) 2017	Age-standardised rates per 100,000 (95% UI) 1990	Age-standardised rates per 100,000 (95% UI) 2017
**Transport and unintentional injuries**	**204 136 (189 121 to 220 112)**	**158 006 (145 080 to 172 165)**	**3 702.9 (3 478.2 to 3 947.5)**	**1 919.5 (1 758.8 to 2 090.7)**
**Transport injuries**	**91 952 (85 626 to 98 020)**	**84 115 (77 622 to 90 957)**	**1 658.3 (1 565.6 to 1 748.3)**	**999.0 (922.6 to 1 079.9)**
Road injuries	89 231 (83 028 to 95 208)	80 155 (74 043 to 86 722)	1 607.3 (1 516.5 to 1 693.4)	952.2 (880.0 to 1 028.3)
Pedestrian road injuries	42 100 (38 079 to 46 518)	35 347 (32 482 to 38 539)	754.7 (697.2 to 816.8)	426.0 (391.7 to 463.8)
Cyclist road injuries	2 184 (1 960 to 2 428)	3 277 (2 940 to 3 656)	41.7 (37.9 to 45.9)	39.3 (35.2 to 43.7)
Motorcyclist road injuries	14 835 (13 307 to 16 368)	11 141 (9 725 to 12 656)	264.5 (240.4 to 288.9)	129.3 (113.0 to 147.0)
Motor vehicle road injuries	26 550 (24 341 to 28 843)	27 195 (24 427 to 29 842)	481.1 (446.2 to 519.3)	319.5 (287.2 to 350.5)
Other road injuries	3 561 (3 030 to 4 109)	3 194 (2 779 to 3 659)	65.4 (56.2 to 74.2)	38.0 (33.2 to 43.4)
Other transport injuries	2 721 (2 468 to 2 991)	3 960 (3 428 to 4 499)	51.0 (46.7 to 55.4)	46.8 (40.5 to 53.0)
**Unintentional injuries**	**112 184 (102 212 to 123 119)**	**73 891 (66 096 to 81 205)**	**2 044.6 (1 882.1 to 2 221.6)**	**920.6 (822.0 to 1 039.7)**
Falls	21 720 (19 224 to 24 654)	27 317 (22 431 to 33 002)	521.5 (458.6 to 591.6)	349.4 (286.6 to 421.2)
Drowning	26 259 (23 277 to 30 249)	11 047 (10 047 to 12 186)	416.8 (377.8 to 469.2)	132.9 (120.9 to 146.7)
Fire, heat, and hot substances	8 346 (7 292 to 9 677)	4 118 (3 541 to 4 932)	150.4 (132.8 to 172.5)	51.1 (44.0 to 61.0)
Poisonings	4 197 (3 727 to 4 734)	2 106 (1 888 to 2 332)	68.8 (62.6 to 76.1)	25.5 (22.8 to 28.2)
Poisoning by carbon monoxide	1 330 (1 175 to 1 504)	753 (669 to 840)	22.3 (20.0 to 24.5)	9.1 (8.0 to 10.1)
Poisoning by other means	2 867 (2 506 to 3 275)	1 353 (1 202 to 1 526)	46.5 (41.6 to 52.2)	16.4 (14.6 to 18.6)
Exposure to mechanical forces	15 220 (14 164 to 16 402)	7 342 (6 428 to 8 600)	290.7 (270.3 to 315.4)	88.6 (77.4 to 103.9)
Unintentional firearm injuries	7 917 (7 251 to 8 686)	2 263 (1 693 to 2 579)	144.0 (132.6 to 158.2)	26.8 (20.1 to 30.5)
Other exposure to mechanical forces	7 302 (6 486 to 8 278)	5 079 (4 273 to 6 187)	146.7 (129.4 to 167.4)	61.8 (52.0 to 75.3)
Adverse effects of medical treatment	4 494 (3 447 to 5 172)	3 924 (3 523 to 4 565)	86.3 (68.3 to 95.9)	50.3 (45.1 to 58.4)
Animal contact	6 132 (5 030 to 7 402)	1 757 (1 569 to 1 969)	93.3 (78.8 to 110.6)	21.9 (19.6 to 24.6)
Venomous animal contact	5 170 (4 122 to 6 428)	1 189 (1 045 to 1 350)	75.8 (61.8 to 91.9)	14.9 (13.1 to 16.9)
Non-venomous animal contact	942 (707 to 1 273)	568 (478 to 717)	17.5 (13.5 to 22.7)	7.0 (5.9 to 8.9)
Foreign body	18 951 (16 069 to 22 182)	11 899 (10 337 to 14 056)	288.7 (251.1 to 330.2)	148.8 (128.7 to 176.9)
Pulmonary aspiration and foreign body in airway	17 705 (14 911 to 20 886)	11 000 (9 562 to 13 067)	264.8 (229.1 to 305.6)	137.6 (119.0 to 164.2)
Foreign body in eyes	244 (124 to 437)	444 (228 to 817)	6.4 (3.4 to 11.3)	5.7 (3.0 to 10.4)
Foreign body in other body part	1 003 (825 to 1 173)	455 (373 to 634)	17.5 (14.6 to 20.3)	5.5 (4.5 to 7.6)
Environmental heat and cold exposure	1 478 (1 332 to 1 657)	650 (560 to 742)	30.8 (27.9 to 34.3)	8.0 (6.9 to 9.2)
Exposure to forces of nature	141 (105 to 187)	155 (130 to 189)	2.1 (1.6 to 2.8)	1.9 (1.6 to 2.3)
Other unintentional injuries	5 266 (4 841 to 5 681)	3 576 (3 195 to 4 025)	95.3 (88.0 to 102.5)	42.2 (37.7 to 47.5)

Death, YLL, incidence, prevalence, YLD, and DALY counts and rates per 100,000 for all injuries in Mexico City in 1990 and 2017

Cause	Deaths			
	All-age counts (95% UI)		Age-standardised rates per 100,000 (95% UI)	
	1990	2017	1990	2017
**Transport and unintentional injuries**	**3 431 (3 337 to 3 540)**	**2 669 (2 476 to 2 883)**	**47.2 (46.1 to 48.5)**	**27.3 (25.5 to 29.5)**
**Transport injuries**	**1 729 (1 660 to 1 810)**	**1 271 (1 159 to 1 403)**	**22.3 (21.5 to 23.1)**	**13.0 (11.8 to 14.3)**
Road injuries	1 700 (1 631 to 1 778)	1 230 (1 122 to 1 357)	21.9 (21.1 to 22.7)	12.5 (11.4 to 13.8)
Pedestrian road injuries	1 009 (954 to 1 054)	642 (582 to 712)	13.9 (13.3 to 14.5)	6.5 (5.9 to 7.2)
Cyclist road injuries	29 (26 to 32)	41 (35 to 47)	0.4 (0.3 to 0.4)	0.4 (0.3 to 0.5)
Motorcyclist road injuries	211 (190 to 234)	131 (113 to 152)	2.3 (2.1 to 2.5)	1.4 (1.2 to 1.6)
Motor vehicle road injuries	431 (396 to 469)	396 (355 to 440)	5.0 (4.6 to 5.4)	4.1 (3.7 to 4.5)
Other road injuries	26 (23 to 30)	21 (18 to 25)	0.3 (0.3 to 0.4)	0.2 (0.2 to 0.2)
Other transport injuries	30 (27 to 33)	41 (31 to 48)	0.4 (0.4 to 0.4)	0.4 (0.3 to 0.5)
**Unintentional injuries**	**1 701 (1 648 to 1 764)**	**1 398 (1 280 to 1 493)**	**24.9 (24.2 to 25.7)**	**14.4 (13.2 to 15.3)**
Falls	544 (520 to 571)	518 (415 to 569)	9.7 (9.2 to 10.2)	5.1 (4.1 to 5.6)
Drowning	255 (240 to 272)	149 (134 to 167)	3.1 (2.9 to 3.2)	1.5 (1.4 to 1.7)
Fire, heat, and hot substances	146 (138 to 156)	100 (83 to 110)	2.2 (2.1 to 2.3)	1.0 (0.9 to 1.1)
Poisonings	55 (52 to 59)	36 (30 to 40)	0.7 (0.7 to 0.7)	0.4 (0.3 to 0.4)
Poisoning by carbon monoxide	25 (24 to 27)	18 (15 to 20)	0.3 (0.3 to 0.3)	0.2 (0.2 to 0.2)
Poisoning by other means	30 (28 to 32)	19 (15 to 21)	0.4 (0.4 to 0.4)	0.2 (0.2 to 0.2)
Exposure to mechanical forces	179 (169 to 190)	115 (103 to 129)	2.3 (2.2 to 2.4)	1.1 (1.0 to 1.3)
Unintentional firearm injuries	81 (73 to 89)	41 (26 to 48)	1.0 (0.8 to 1.0)	0.4 (0.3 to 0.5)
Other exposure to mechanical forces	98 (90 to 108)	74 (65 to 89)	1.3 (1.2 to 1.4)	0.7 (0.6 to 0.9)
Adverse effects of medical treatment	112 (105 to 134)	129 (107 to 165)	1.9 (1.8 to 2.2)	1.3 (1.1 to 1.7)
Animal contact	10 (9 to 11)	6 (6 to 7)	0.1 (0.1 to 0.1)	0.1 (0.1 to 0.1)
Venomous animal contact	4 (3 to 5)	2 (1 to 3)	0.1 (0.0 to 0.1)	0.0 (0.0 to 0.0)
Non-venomous animal contact	5 (5 to 7)	4 (3 to 5)	0.1 (0.1 to 0.1)	0.0 (0.0 to 0.1)
Foreign body	322 (298 to 352)	196 (177 to 217)	4.0 (3.7 to 4.2)	2.2 (2.0 to 2.5)
Pulmonary aspiration and foreign body in airway	311 (288 to 342)	191 (173 to 212)	3.8 (3.6 to 4.1)	2.2 (1.9 to 2.5)
Foreign body in eyes				
Foreign body in other body part	11 (9 to 14)	4 (3 to 8)	0.1 (0.1 to 0.2)	0.0 (0.0 to 0.1)
Environmental heat and cold exposure	23 (21 to 25)	12 (8 to 13)	0.4 (0.3 to 0.4)	0.1 (0.1 to 0.1)
Exposure to forces of nature	3 (3 to 3)	99 (91 to 108)	0.0 (0.0 to 0.0)	1.1 (1.0 to 1.2)
Other unintentional injuries	52 (48 to 56)	38 (33 to 44)	0.6 (0.6 to 0.7)	0.4 (0.3 to 0.4)

Cause	Years of life lost			
	All-age counts (95% UI)		Age-standardised rates per 100,000 (95% UI)	
	1990	2017	1990	2017
**Transport and unintentional injuries**	**167 014 (160 686 to 174 812)**	**106 926 (98 884 to 115 908)**	**1 922.3 (1 860.2 to 1 999.5)**	**1 153.9 (1 069.6 to 1 251.8)**
**Transport injuries**	**88 408 (84 028 to 93 357)**	**55 999 (50 894 to 61 945)**	**995.0 (952.6 to 1 043.6)**	**594.9 (541.4 to 658.5)**
Road injuries	86 919 (82 610 to 91 902)	54 202 (49 230 to 60 021)	978.0 (935.2 to 1 025.7)	576.2 (523.8 to 636.6)
Pedestrian road injuries	48 083 (44 976 to 51 270)	24 717 (22 306 to 27 680)	565.9 (534.4 to 597.3)	260.6 (234.4 to 291.4)
Cyclist road injuries	1 544 (1 386 to 1 740)	1 711 (1 479 to 1 987)	17.4 (15.7 to 19.3)	17.6 (15.3 to 20.4)
Motorcyclist road injuries	12 124 (10 812 to 13 537)	7 211 (6 160 to 8 384)	125.8 (113.5 to 139.3)	78.0 (66.4 to 90.7)
Motor vehicle road injuries	23 777 (21 616 to 26 102)	19 616 (17 491 to 21 999)	253.6 (233.3 to 276.7)	210.0 (187.4 to 235.8)
Other road injuries	1 390 (1 178 to 1 631)	947 (805 to 1 107)	15.3 (13.1 to 17.5)	10.0 (8.5 to 11.7)
Other transport injuries	1 490 (1 342 to 1 670)	1 797 (1 366 to 2 096)	17.1 (15.5 to 18.9)	18.7 (14.3 to 21.6)
**Unintentional injuries**	**78 606 (74 868 to 81 226)**	**50 927 (47 065 to 54 573)**	**927.2 (890.1 to 971.6)**	**559.1 (515.4 to 604.7)**
Falls	16 613 (15 870 to 17 502)	13 516 (10 416 to 14 993)	238.4 (228.2 to 250.6)	134.2 (104.3 to 148.1)
Drowning	14 573 (13 527 to 15 740)	6 976 (6 240 to 7 790)	156.6 (146.7 to 168.5)	77.1 (68.5 to 86.3)
Fire, heat, and hot substances	6 609 (6 115 to 7 262)	3 089 (2 482 to 3 414)	78.9 (73.9 to 85.4)	33.0 (26.8 to 36.6)
Poisonings	3 068 (2 846 to 3 322)	1 568 (1 289 to 1 749)	33.6 (31.4 to 36.4)	17.3 (14.3 to 19.4)
Poisoning by carbon monoxide	1 389 (1 266 to 1 524)	762 (627 to 865)	15.1 (13.9 to 16.5)	8.4 (6.9 to 9.5)
Poisoning by other means	1 679 (1 542 to 1 847)	806 (657 to 906)	18.5 (17.1 to 20.2)	9.0 (7.4 to 10.2)
Exposure to mechanical forces	9 312 (8 721 to 9 908)	4 737 (4 205 to 5 343)	104.3 (98.4 to 110.6)	49.1 (43.7 to 55.3)
Unintentional firearm injuries	4 479 (3 920 to 4 900)	1 858 (1 188 to 2 197)	48.0 (41.8 to 52.3)	19.3 (12.4 to 22.9)
Other exposure to mechanical forces	4 833 (4 435 to 5 364)	2 880 (2 510 to 3 549)	56.3 (52.0 to 62.2)	29.8 (26.0 to 36.8)
Adverse effects of medical treatment	4 092 (3 716 to 5 448)	3 472 (2 872 to 4 690)	54.0 (49.8 to 68.3)	37.9 (31.2 to 52.3)
Animal contact	545 (476 to 627)	228 (202 to 254)	6.1 (5.5 to 6.9)	2.6 (2.3 to 2.9)
Venomous animal contact	279 (194 to 347)	88 (47 to 112)	3.0 (2.0 to 3.7)	1.0 (0.6 to 1.3)
Non-venomous animal contact	266 (231 to 340)	139 (116 to 183)	3.2 (2.8 to 4.0)	1.5 (1.3 to 2.0)
Foreign body	19 935 (17 943 to 22 460)	10 095 (8 764 to 11 572)	211.1 (192.4 to 234.9)	125.1 (107.0 to 147.8)
Pulmonary aspiration and foreign body in airway	19 296 (17 345 to 21 819)	9 818 (8 518 to 11 312)	204.2 (185.7 to 227.4)	122.7 (104.7 to 145.0)
Foreign body in eyes				
Foreign body in other body part	639 (551 to 847)	217 (152 to 408)	6.9 (6.0 to 9.1)	2.5 (1.7 to 4.7)
Environmental heat and cold exposure	881 (816 to 946)	385 (279 to 435)	11.9 (11.0 to 12.7)	3.8 (2.8 to 4.3)
Exposure to forces of nature	162 (148 to 180)	5 131 (4 657 to 5 636)	1.7 (1.6 to 1.9)	60.3 (54.7 to 66.8)
Other unintentional injuries	2 815 (2 598 to 3 064)	1 789 (1 550 to 2 079)	30.5 (28.4 to 33.1)	18.7 (16.2 to 21.7)

Cause	Incidence			
	All-age counts (95% UI)		Age-standardised rates per 100,000 (95% UI)	
	1990	2017	1990	2017
**Transport and unintentional injuries**	**325 738 (302 919 to 352 089)**	**521 533 (477 984 to 566 257)**	**4 222.5 (3 924.6 to 4 549.9)**	**5 636.3 (5 155.4 to 6 146.5)**
**Transport injuries**	**29 109 (25 059 to 33 792)**	**50 098 (43 585 to 56 864)**	**358.0 (311.0 to 408.3)**	**529.6 (460.6 to 602.8)**
Road injuries	27 916 (23 904 to 32 543)	49 395 (42 910 to 56 177)	344.4 (294.4 to 394.1)	523.9 (452.4 to 595.8)
Pedestrian road injuries	6 058 (4 982 to 7 319)	11 413 (9 350 to 13 699)	79.5 (66.1 to 94.8)	119.7 (98.2 to 143.4)
Cyclist road injuries	3 671 (2 773 to 4 828)	5 712 (4 486 to 7 226)	48.6 (38.7 to 56.6)	64.6 (50.0 to 82.5)
Motorcyclist road injuries	3 388 (2 627 to 4 323)	4 189 (3 184 to 5 355)	39.2 (30.7 to 49.3)	45.7 (34.5 to 58.6)
Motor vehicle road injuries	10 199 (8 208 to 12 326)	21 920 (18 002 to 26 010)	125.1 (101.6 to 149.8)	227.2 (187.0 to 270.2)
Other road injuries	4 590 (3 278 to 6 088)	6 160 (4 356 to 8 308)	57.1 (42.7 to 72.6)	64.7 (45.2 to 88.9)
Other transport injuries	1 193 (779 to 1 736)	704 (482 to 999)	13.6 (9.4 to 19.1)	7.7 (5.1 to 11.2)
**Unintentional injuries**	**296 629 (273 710 to 321 952)**	**471 435 (428 806 to 515 378)**	**3 864.4 (3 567.8 to 4 196.8)**	**5 106.7 (4 622.8 to 5 590.2)**
Falls	162 328 (143 607 to 183 731)	311 729 (272 320 to 353 374)	2 292.5 (2 043.6 to 2 581.4)	3 356.8 (2 907.0 to 3 809.1)
Drowning	244 (211 to 284)	178 (135 to 217)	2.9 (2.5 to 3.3)	1.9 (1.5 to 2.3)
Fire, heat, and hot substances	12 805 (10 472 to 15 469)	13 909 (11 516 to 16 561)	139.2 (115.2 to 167.8)	171.1 (139.8 to 206.0)
Poisonings	2 855 (2 076 to 3 938)	2 733 (2 007 to 3 654)	29.5 (21.6 to 40.5)	37.0 (26.7 to 50.8)
Poisoning by carbon monoxide	529 (320 to 829)	414 (258 to 614)	5.8 (3.7 to 8.8)	5.0 (2.9 to 7.8)
Poisoning by other means	2 326 (1 652 to 3 280)	2 319 (1 676 to 3 181)	23.7 (17.0 to 33.5)	32.0 (22.5 to 44.9)
Exposure to mechanical forces	55 010 (46 174 to 66 316)	64 605 (55 694 to 74 279)	622.0 (530.1 to 737.3)	704.8 (605.8 to 811.2)
Unintentional firearm injuries	4 180 (3 068 to 5 516)	4 441 (3 322 to 5 776)	45.5 (34.0 to 59.4)	47.3 (35.3 to 61.8)
Other exposure to mechanical forces	50 830 (42 833 to 61 310)	60 164 (51 813 to 69 422)	576.5 (491.1 to 687.8)	657.5 (563.4 to 758.4)
Adverse effects of medical treatment	8 176 (6 961 to 9 425)	13 360 (11 565 to 15 310)	111.6 (95.7 to 127.6)	133.9 (115.8 to 151.8)
Animal contact	16 552 (12 578 to 21 671)	9 905 (8 087 to 12 035)	194.6 (150.9 to 245.7)	111.1 (90.4 to 137.6)
Venomous animal contact	5 863 (4 078 to 8 397)	3 059 (2 171 to 4 197)	68.1 (48.9 to 93.5)	34.1 (24.2 to 46.9)
Non-venomous animal contact	10 689 (7 851 to 14 250)	6 846 (5 349 to 8 487)	126.5 (95.6 to 166.3)	77.1 (60.3 to 97.4)
Foreign body	18 995 (11 292 to 27 931)	23 640 (14 426 to 36 800)	240.4 (142.8 to 358.4)	244.5 (153.8 to 364.7)
Pulmonary aspiration and foreign body in airway	752 (548 to 1 011)	383 (282 to 506)	8.0 (6.0 to 10.6)	5.0 (3.6 to 6.7)
Foreign body in eyes	16 058 (8 647 to 25 134)	22 062 (12 828 to 35 485)	208.6 (113.0 to 327.9)	225.3 (133.7 to 345.5)
Foreign body in other body part	2 124 (1 456 to 3 135)	1 196 (889 to 1 506)	23.8 (16.5 to 33.8)	14.2 (10.3 to 18.5)
Environmental heat and cold exposure	3 265 (2 284 to 4 558)	1 206 (902 to 1 675)	39.5 (28.6 to 52.3)	13.2 (9.8 to 18.9)
Exposure to forces of nature	424 (347 to 531)	6 826 (5 761 to 8 196)	4.6 (3.8 to 5.7)	81.8 (67.9 to 99.9)
Other unintentional injuries	16 035 (13 441 to 19 178)	23 347 (19 888 to 26 861)	187.7 (158.7 to 220.5)	250.7 (214.3 to 290.9)

Cause	Prevalence			
	All-age counts (95% UI)		Age-standardised rates per 100,000 (95% UI)	
	1990	2017	1990	2017
**Transport and unintentional injuries**	**743 649 (696 199 to 798 414)**	**1 303 943 (1 216 367 to 1 421 570)**	**10 507.8 (9 844.0 to 11 326.2)**	**12 885.6 (11 997.0 to 14 056.8)**
**Transport injuries**	**72 839 (68 091 to 77 852)**	**154 391 (143 783 to 166 336)**	**1 062.4 (992.8 to 1 137.0)**	**1 491.1 (1 391.6 to 1 605.5)**
Road injuries	66 699 (62 154 to 71 438)	150 421 (139 608 to 162 286)	982.6 (915.7 to 1 054.3)	1 451.8 (1 350.8 to 1 564.5)
Pedestrian road injuries	21 931 (19 047 to 25 418)	51 934 (45 167 to 60 231)	330.8 (289.2 to 381.8)	501.9 (437.0 to 582.4)
Cyclist road injuries	6 245 (5 268 to 7 405)	13 076 (11 092 to 15 500)	87.8 (74.6 to 101.8)	129.3 (109.7 to 153.7)
Motorcyclist road injuries	12 052 (10 451 to 14 048)	20 348 (17 336 to 23 993)	171.9 (149.2 to 198.5)	197.0 (168.1 to 231.9)
Motor vehicle road injuries	17 525 (15 240 to 20 553)	50 012 (43 290 to 58 466)	263.8 (228.9 to 309.6)	477.9 (414.7 to 558.0)
Other road injuries	8 946 (7 480 to 10 752)	15 050 (12 540 to 18 059)	129.0 (107.2 to 154.4)	145.7 (121.8 to 175.3)
Other transport injuries	6 140 (4 930 to 7 601)	3 970 (3 421 to 4 536)	79.8 (65.3 to 96.4)	39.3 (33.8 to 45.1)
**Unintentional injuries**	**670 810 (623 166 to 725 757)**	**1 149 552 (1 064 319 to 1 265 451)**	**9 445.4 (8 792.6 to 10 251.9)**	**11 394.5 (10 541.1 to 12 571.8)**
Falls	301 600 (266 268 to 344 075)	666 674 (592 675 to 761 347)	4 581.2 (4 080.4 to 5 241.5)	6 628.3 (5 874.3 to 7 599.6)
Drowning	1 201 (1 060 to 1 384)	1 214 (1 059 to 1 414)	17.6 (15.7 to 20.2)	11.8 (10.3 to 13.8)
Fire, heat, and hot substances	136 240 (113 866 to 169 235)	192 494 (159 290 to 239 376)	1 759.7 (1 477.7 to 2 154.7)	1 940.0 (1 608.7 to 2 446.3)
Poisonings	2 882 (2 170 to 3 608)	4 033 (2 935 to 5 104)	34.2 (25.9 to 42.6)	42.7 (30.9 to 54.2)
Poisoning by carbon monoxide	408 (310 to 534)	374 (286 to 479)	5.0 (3.8 to 6.4)	3.9 (3.0 to 5.0)
Poisoning by other means	2 474 (1 777 to 3 164)	3 659 (2 576 to 4 709)	29.2 (21.1 to 37.3)	38.8 (27.2 to 50.0)
Exposure to mechanical forces	112 773 (98 665 to 129 767)	180 140 (158 365 to 207 240)	1 576.5 (1 389.8 to 1 806.4)	1 752.2 (1 534.7 to 2 017.3)
Unintentional firearm injuries	15 730 (12 451 to 18 890)	23 300 (18 772 to 28 017)	222.2 (178.9 to 265.4)	223.2 (180.3 to 267.9)
Other exposure to mechanical forces	97 043 (82 277 to 114 569)	156 839 (134 130 to 185 117)	1 354.3 (1 159.7 to 1 587.3)	1 528.9 (1 306.4 to 1 806.6)
Adverse effects of medical treatment	626 (486 to 780)	1 029 (785 to 1 252)	8.5 (6.6 to 10.6)	10.2 (7.9 to 12.6)
Animal contact	19 296 (16 607 to 22 529)	14 158 (12 232 to 16 389)	263.4 (228.3 to 308.5)	141.5 (122.1 to 164.3)
Venomous animal contact	4 226 (3 468 to 5 014)	2 417 (2 091 to 2 840)	55.1 (45.8 to 64.5)	24.6 (20.6 to 28.9)
Non-venomous animal contact	15 070 (12 447 to 18 164)	11 735 (9 846 to 13 943)	208.3 (174.3 to 247.0)	116.9 (97.7 to 139.6)
Foreign body	11 276 (8 989 to 14 361)	10 477 (8 242 to 13 523)	149.2 (120.7 to 186.4)	105.9 (82.7 to 136.6)
Pulmonary aspiration and foreign body in airway	590 (418 to 805)	432 (340 to 549)	7.2 (5.3 to 9.7)	4.5 (3.5 to 5.7)
Foreign body in eyes	3 175 (1 660 to 5 384)	4 970 (2 958 to 7 650)	46.0 (25.1 to 72.4)	49.6 (29.0 to 76.6)
Foreign body in other body part	7 511 (5 924 to 9 640)	5 075 (4 173 to 6 347)	96.0 (76.7 to 121.4)	51.8 (42.3 to 65.2)
Environmental heat and cold exposure	13 096 (10 420 to 16 299)	5 004 (4 292 to 5 931)	174.8 (142.2 to 212.4)	49.8 (42.5 to 59.2)
Exposure to forces of nature	46 582 (32 375 to 66 049)	30 209 (20 777 to 42 640)	538.9 (376.2 to 756.6)	280.5 (192.3 to 393.2)
Other unintentional injuries	25 288 (21 402 to 29 369)	44 555 (38 402 to 51 014)	341.4 (292.8 to 393.7)	435.4 (376.7 to 498.6)

Cause	Years lived with disability			
	All-age counts (95% UI)		Age-standardised rates per 100,000 (95% UI)	
	1990	2017	1990	2017
**Transport and unintentional injuries**	**32 663 (24 104 to 43 039)**	**52 912 (37 863 to 71 829)**	**459.6 (336.5 to 607.0)**	**525.0 (377.1 to 714.0)**
**Transport injuries**	**4 785 (3 496 to 6 342)**	**8 960 (6 475 to 12 015)**	**67.7 (49.2 to 89.1)**	**86.9 (62.6 to 116.6)**
Road injuries	4 406 (3 232 to 5 848)	8 759 (6 334 to 11 759)	62.9 (46.1 to 83.4)	84.9 (61.2 to 114.1)
Pedestrian road injuries	1 505 (1 074 to 2 020)	3 150 (2 216 to 4 297)	22.0 (15.7 to 29.5)	30.6 (21.6 to 41.6)
Cyclist road injuries	414 (291 to 545)	737 (506 to 980)	5.7 (4.0 to 7.4)	7.3 (5.1 to 9.8)
Motorcyclist road injuries	806 (565 to 1 092)	1 180 (806 to 1 614)	11.1 (7.8 to 15.1)	11.5 (7.8 to 15.7)
Motor vehicle road injuries	1 195 (867 to 1 588)	2 988 (2 128 to 4 016)	17.4 (12.6 to 22.9)	28.7 (20.4 to 38.4)
Other road injuries	486 (349 to 658)	705 (502 to 975)	6.8 (4.9 to 9.2)	6.9 (4.9 to 9.4)
Other transport injuries	379 (266 to 516)	200 (143 to 271)	4.8 (3.4 to 6.4)	2.0 (1.4 to 2.7)
**Unintentional injuries**	**27 878 (20 541 to 36 762)**	**43 952 (31 376 to 59 846)**	**391.9 (287.7 to 519.0)**	**438.2 (312.7 to 596.9)**
Falls	17 077 (12 394 to 22 557)	32 309 (23 095 to 43 452)	253.9 (184.1 to 334.0)	322.5 (231.0 to 433.5)
Drowning	67 (47 to 93)	62 (42 to 87)	1.0 (0.7 to 1.3)	0.6 (0.4 to 0.8)
Fire, heat, and hot substances	2 823 (1 734 to 4 482)	3 597 (2 114 to 5 882)	35.7 (21.8 to 56.1)	36.5 (21.5 to 60.1)
Poisonings	337 (204 to 510)	464 (277 to 710)	3.9 (2.4 to 5.9)	5.0 (2.9 to 7.6)
Poisoning by carbon monoxide	43 (26 to 69)	36 (22 to 58)	0.5 (0.3 to 0.8)	0.4 (0.2 to 0.6)
Poisoning by other means	293 (175 to 448)	428 (250 to 668)	3.4 (2.0 to 5.2)	4.6 (2.7 to 7.1)
Exposure to mechanical forces	2 594 (1 775 to 3 738)	3 784 (2 549 to 5 529)	35.7 (24.5 to 51.1)	37.0 (25.0 to 53.6)
Unintentional firearm injuries	322 (230 to 490)	434 (281 to 688)	4.4 (2.9 to 6.7)	4.2 (2.7 to 6.6)
Other exposure to mechanical forces	2 272 (1 554 to 3 283)	3 349 (2 259 to 4 910)	31.3 (21.5 to 45.0)	32.8 (22.1 to 47.9)
Adverse effects of medical treatment	83 (51 to 126)	136 (85 to 205)	1.1 (0.7 to 1.7)	1.4 (0.8 to 2.1)
Animal contact	498 (328 to 715)	318 (210 to 460)	6.3 (4.2 to 9.1)	3.3 (2.1 to 4.7)
Venomous animal contact	274 (175 to 404)	158 (99 to 233)	3.3 (2.1 to 4.8)	1.7 (1.0 to 2.5)
Non-venomous animal contact	224 (133 to 359)	160 (93 to 262)	3.1 (1.8 to 4.9)	1.6 (0.9 to 2.6)
Foreign body	668 (454 to 941)	569 (365 to 856)	8.7 (5.9 to 12.2)	5.7 (3.7 to 8.6)
Pulmonary aspiration and foreign body in airway	29 (20 to 40)	17 (12 to 24)	0.3 (0.2 to 0.5)	0.2 (0.1 to 0.3)
Foreign body in eyes	212 (110 to 376)	333 (187 to 562)	3.1 (1.7 to 5.3)	3.3 (1.8 to 5.6)
Foreign body in other body part	426 (294 to 567)	219 (155 to 294)	5.2 (3.7 to 6.9)	2.3 (1.6 to 3.0)
Environmental heat and cold exposure	496 (325 to 700)	188 (121 to 270)	6.6 (4.4 to 9.4)	1.9 (1.2 to 2.7)
Exposure to forces of nature	2 287 (1 650 to 3 134)	1 101 (812 to 1 483)	26.4 (19.2 to 35.8)	10.4 (7.6 to 14.0)
Other unintentional injuries	947 (685 to 1 277)	1 424 (989 to 1 976)	12.6 (9.1 to 17.0)	14.0 (9.7 to 19.5)

Cause	Disability adjusted life years			
	All-age counts (95% UI)		Age-standardised rates per 100,000 (95% UI)	
	1990	2017	1990	2017
**Transport and unintentional injuries**	**199 677 (188 906 to 212 631)**	**159 838 (142 273 to 180 599)**	**2 381.9 (2 246.4 to 2 556.9)**	**1 679.0 (1 501.9 to 1 883.9)**
**Transport injuries**	**93 193 (88 591 to 98 373)**	**64 959 (59 263 to 71 609)**	**1 062.7 (1 016.2 to 1 113.5)**	**681.7 (621.8 to 753.3)**
Road injuries	91 324 (86 794 to 96 351)	62 961 (57 448 to 69 429)	1 040.9 (995.6 to 1 090.4)	661.1 (603.0 to 729.9)
Pedestrian road injuries	49 589 (46 594 to 52 824)	27 867 (25 200 to 30 976)	587.9 (556.9 to 619.9)	291.1 (263.4 to 323.9)
Cyclist road injuries	1 958 (1 770 to 2 187)	2 448 (2 102 to 2 809)	23.0 (20.9 to 25.6)	25.0 (21.6 to 28.6)
Motorcyclist road injuries	12 930 (11 608 to 14 268)	8 391 (7 317 to 9 661)	136.9 (124.4 to 150.2)	89.4 (77.7 to 102.9)
Motor vehicle road injuries	24 972 (22 772 to 27 181)	22 604 (20 360 to 24 903)	271.0 (250.0 to 293.7)	238.7 (214.6 to 264.0)
Other road injuries	1 876 (1 614 to 2 148)	1 652 (1 399 to 1 974)	22.1 (19.1 to 25.4)	16.8 (14.3 to 20.1)
Other transport injuries	1 869 (1 680 to 2 078)	1 998 (1 541 to 2 300)	21.8 (19.6 to 24.2)	20.6 (16.2 to 23.7)
**Unintentional injuries**	**106 484 (98 184 to 115 947)**	**94 879 (81 227 to 111 830)**	**1 319.1 (1 204.3 to 1 445.0)**	**997.3 (861.4 to 1 164.6)**
Falls	33 690 (28 965 to 39 092)	45 825 (35 873 to 57 120)	492.3 (422.6 to 572.6)	456.7 (356.7 to 568.7)
Drowning	14 640 (13 605 to 15 818)	7 039 (6 308 to 7 862)	157.6 (147.8 to 169.4)	77.7 (69.2 to 86.9)
Fire, heat, and hot substances	9 432 (8 156 to 11 164)	6 685 (5 111 to 9 008)	114.6 (99.3 to 135.8)	69.5 (53.4 to 92.5)
Poisonings	3 405 (3 136 to 3 697)	2 032 (1 693 to 2 341)	37.6 (34.8 to 40.8)	22.3 (18.8 to 25.7)
Poisoning by carbon monoxide	1 433 (1 312 to 1 571)	799 (656 to 899)	15.6 (14.4 to 17.1)	8.7 (7.2 to 9.9)
Poisoning by other means	1 972 (1 771 to 2 193)	1 233 (1 009 to 1 485)	21.9 (19.9 to 24.4)	13.6 (11.2 to 16.4)
Exposure to mechanical forces	11 906 (10 839 to 13 115)	8 521 (7 105 to 10 400)	140.0 (125.9 to 156.8)	86.0 (71.9 to 104.0)
Unintentional firearm injuries	4 800 (4 228 to 5 266)	2 292 (1 614 to 2 702)	52.4 (45.9 to 57.1)	23.4 (16.6 to 27.5)
Other exposure to mechanical forces	7 106 (6 220 to 8 290)	6 229 (4 996 to 7 914)	87.6 (76.0 to 102.1)	62.6 (50.4 to 78.8)
Adverse effects of medical treatment	4 175 (3 794 to 5 520)	3 609 (3 025 to 4 848)	55.1 (51.0 to 69.4)	39.3 (32.5 to 53.9)
Animal contact	1 043 (859 to 1 265)	545 (435 to 683)	12.5 (10.2 to 15.3)	5.8 (4.7 to 7.3)
Venomous animal contact	553 (424 to 711)	246 (179 to 322)	6.2 (4.8 to 8.0)	2.7 (2.0 to 3.5)
Non-venomous animal contact	490 (385 to 631)	299 (226 to 407)	6.2 (4.9 to 8.1)	3.1 (2.4 to 4.2)
Foreign body	20 603 (18 553 to 23 209)	10 604 (9 299 to 12 193)	219.8 (199.9 to 244.1)	130.9 (112.7 to 154.2)
Pulmonary aspiration and foreign body in airway	19 326 (17 370 to 21 854)	9 836 (8 536 to 11 330)	204.5 (186.0 to 227.7)	122.9 (104.9 to 145.1)
Foreign body in eyes	212 (110 to 376)	333 (187 to 562)	3.1 (1.7 to 5.3)	3.3 (1.8 to 5.6)
Foreign body in other body part	1 065 (900 to 1 328)	436 (330 to 630)	12.2 (10.3 to 15.0)	4.7 (3.6 to 7.3)
Environmental heat and cold exposure	1 377 (1 190 to 1 601)	573 (450 to 682)	18.5 (16.1 to 21.5)	5.7 (4.5 to 6.7)
Exposure to forces of nature	2 450 (1 813 to 3 296)	6 232 (5 671 to 6 847)	28.1 (21.0 to 37.6)	70.7 (64.3 to 77.7)
Other unintentional injuries	3 763 (3 418 to 4 145)	3 213 (2 715 to 3 828)	43.1 (39.0 to 47.8)	32.7 (27.7 to 38.8)

Death, YLL, Incidence, prevalence, YLD, and DALY counts and rates per 100,000 for all injuries in México in 1990 and 2017

Cause	Deaths: All-age counts (95% UI) 1990	Deaths: All-age counts (95% UI) 2017	Deaths: Age-standardised rates per 100,000 (95% UI) 1990	Deaths: Age-standardised rates per 100,000 (95% UI) 2017	Years of life lost: All-age counts (95% UI) 1990	Years of life lost: All-age counts (95% UI) 2017	Years of life lost: Age-standardised rates per 100,000 (95% UI) 1990	Years of life lost: Age-standardised rates per 100,000 (95% UI) 2017
**Transport and unintentional injuries**	**4 660 (4 496 to 4 846)**	**4 788 (4 390 to 5 235)**	**60.4 (58.8 to 62.1)**	**30.3 (27.9 to 33.0)**	**258 929 (246 638 to 272 675)**	**217 580 (197 791 to 241 182)**	**2 584.8 (2 485.7 to 2 694.0)**	**1 265.2 (1 150.0 to 1 402.3)**
**Transport injuries**	**2 269 (2 153 to 2 386)**	**2 430 (2 111 to 2 684)**	**27.2 (26.1 to 28.4)**	**14.4 (12.6 to 15.8)**	**125 308 (117 867 to 132 810)**	**118 960 (103 273 to 131 893)**	**1 249.9 (1 186.9 to 1 316.0)**	**671.1 (584.3 to 743.5)**
Road injuries	2 222 (2 107 to 2 337)	2 348 (2 040 to 2 593)	26.6 (25.5 to 27.8)	13.9 (12.2 to 15.3)	122 720 (115 298 to 130 084)	114 992 (99 851 to 127 740)	1 223.2 (1 161.2 to 1 288.6)	649.1 (564.1 to 719.1)
Pedestrian road injuries	1 279 (1 204 to 1 355)	1 239 (1 098 to 1 360)	16.7 (15.9 to 17.4)	7.6 (6.8 to 8.3)	68 523 (63 747 to 73 588)	56 149 (49 682 to 62 118)	709.3 (668.6 to 750.7)	321.7 (285.4 to 355.7)
Cyclist road injuries	42 (38 to 47)	72 (62 to 82)	0.5 (0.4 to 0.5)	0.4 (0.4 to 0.5)	2 344 (2 096 to 2 611)	3 338 (2 857 to 3 828)	23.3 (21.0 to 25.7)	18.9 (16.2 to 21.6)
Motorcyclist road injuries	264 (236 to 291)	227 (192 to 263)	2.7 (2.4 to 2.9)	1.3 (1.1 to 1.5)	15 403 (13 677 to 17 138)	12 940 (10 907 to 15 057)	144.0 (129.2 to 157.9)	71.3 (60.1 to 83.0)
Motor vehicle road injuries	576 (526 to 628)	745 (614 to 850)	6.1 (5.7 to 6.6)	4.2 (3.5 to 4.8)	32 947 (29 799 to 36 124)	39 310 (32 578 to 44 807)	313.3 (286.6 to 340.5)	218.9 (182.1 to 249.4)
Other road injuries	61 (50 to 71)	65 (55 to 76)	0.7 (0.6 to 0.8)	0.4 (0.3 to 0.4)	3 504 (2 802 to 4 125)	3 254 (2 724 to 3 823)	33.3 (27.3 to 38.5)	18.3 (15.3 to 21.4)
Other transport injuries	48 (44 to 52)	82 (69 to 94)	0.6 (0.6 to 0.6)	0.5 (0.4 to 0.5)	2 588 (2 374 to 2 887)	3 968 (3 295 to 4 640)	26.7 (24.7 to 28.9)	22.2 (18.5 to 25.8)
**Unintentional injuries**	**2 390 (2 295 to 2 499)**	**2 358 (2 186 to 2 585)**	**33.1 (32.2 to 34.4)**	**15.9 (14.8 to 17.5)**	**133 621 (125 946 to 142 539)**	**98 620 (90 160 to 110 864)**	**1 334.9 (1 274.0 to 1 406.0)**	**593.9 (542.3 to 669.0)**
Falls	538 (514 to 567)	823 (754 to 894)	11.2 (10.6 to 11.9)	6.2 (5.7 to 6.7)	20 993 (19 838 to 22 271)	23 117 (21 027 to 25 822)	284.7 (271.7 to 300.5)	145.1 (132.3 to 160.6)
Drowning	476 (441 to 514)	282 (257 to 310)	5.0 (4.7 to 5.3)	1.7 (1.5 to 1.8)	29 767 (27 096 to 32 829)	14 911 (13 467 to 16 499)	266.7 (245.9 to 289.7)	85.8 (77.4 to 94.9)
Fire, heat, and hot substances	181 (168 to 196)	130 (121 to 139)	2.6 (2.5 to 2.8)	0.9 (0.8 to 1.0)	10 337 (9 335 to 11 597)	4 569 (4 154 to 5 000)	101.7 (93.5 to 111.7)	28.0 (25.5 to 30.6)
Poisonings	98 (91 to 105)	68 (62 to 74)	1.1 (1.0 to 1.1)	0.4 (0.4 to 0.5)	6 130 (5 591 to 6 719)	3 285 (2 983 to 3 636)	55.4 (51.3 to 59.9)	19.1 (17.3 to 21.2)
Poisoning by carbon monoxide	46 (42 to 50)	35 (32 to 39)	0.5 (0.5 to 0.5)	0.2 (0.2 to 0.2)	2 829 (2 561 to 3 110)	1 688 (1 518 to 1 889)	25.6 (23.3 to 27.8)	9.7 (8.8 to 10.9)
Poisoning by other means	52 (47 to 56)	33 (30 to 36)	0.6 (0.5 to 0.6)	0.2 (0.2 to 0.2)	3 301 (2 963 to 3 666)	1 597 (1 428 to 1 787)	29.8 (27.2 to 32.6)	9.3 (8.4 to 10.5)
Exposure to mechanical forces	277 (260 to 293)	188 (171 to 204)	3.3 (3.1 to 3.5)	1.1 (1.0 to 1.2)	15 415 (14 391 to 16 449)	8 445 (7 638 to 9 238)	152.7 (143.6 to 161.7)	48.0 (43.4 to 52.4)
Unintentional firearm injuries	121 (107 to 132)	60 (49 to 68)	1.3 (1.2 to 1.4)	0.4 (0.3 to 0.4)	6 973 (6 111 to 7 695)	2 906 (2 085 to 3 334)	65.5 (57.7 to 71.8)	16.2 (11.7 to 18.6)
Other exposure to mechanical forces	156 (144 to 169)	128 (114 to 149)	2.0 (1.9 to 2.1)	0.8 (0.7 to 0.9)	8 441 (7 724 to 9 258)	5 540 (4 865 to 6 614)	87.2 (80.5 to 94.3)	31.7 (28.0 to 37.8)
Adverse effects of medical treatment	96 (88 to 126)	170 (143 to 285)	1.8 (1.6 to 2.3)	1.2 (1.0 to 2.0)	4 546 (4 054 to 5 994)	5 635 (4 561 to 9 905)	52.0 (47.5 to 68.6)	35.6 (29.0 to 61.9)
Animal contact	49 (43 to 56)	27 (24 to 29)	0.6 (0.5 to 0.6)	0.2 (0.2 to 0.2)	3 354 (2 864 to 3 848)	1 108 (993 to 1 242)	29.5 (25.7 to 34.0)	6.7 (6.1 to 7.6)
Venomous animal contact	33 (28 to 39)	12 (9 to 15)	0.3 (0.3 to 0.4)	0.1 (0.1 to 0.1)	2 440 (1 995 to 2 943)	526 (392 to 652)	20.5 (17.1 to 24.3)	3.3 (2.4 to 4.0)
Non-venomous animal contact	16 (14 to 18)	14 (13 to 18)	0.2 (0.2 to 0.2)	0.1 (0.1 to 0.1)	914 (777 to 1 056)	582 (496 to 753)	9.0 (7.6 to 10.2)	3.5 (3.0 to 4.5)
Foreign body	488 (446 to 531)	531 (470 to 609)	5.4 (5.1 to 5.8)	3.3 (2.9 to 3.8)	32 354 (28 880 to 35 990)	30 700 (26 136 to 36 511)	288.8 (262.8 to 316.6)	187.2 (157.5 to 224.8)
Pulmonary aspiration and foreign body in airway	471 (429 to 514)	520 (461 to 592)	5.2 (4.9 to 5.6)	3.3 (2.9 to 3.7)	31 276 (27 875 to 34 943)	30 116 (25 606 to 35 856)	279.2 (252.9 to 306.6)	183.9 (154.8 to 221.1)
Foreign body in eyes								
Foreign body in other body part	17 (15 to 21)	11 (8 to 21)	0.2 (0.1 to 0.2)	0.1 (0.0 to 0.1)	1 078 (924 to 1 311)	583 (427 to 1 196)	9.6 (8.4 to 12.0)	3.3 (2.4 to 6.8)
Environmental heat and cold exposure	35 (33 to 37)	21 (19 to 24)	0.6 (0.5 to 0.6)	0.1 (0.1 to 0.2)	1 698 (1 555 to 1 829)	787 (693 to 887)	19.8 (18.4 to 21.1)	4.6 (4.1 to 5.1)
Exposure to forces of nature	6 (6 to 7)	3 (3 to 3)	0.1 (0.1 to 0.1)	0.0 (0.0 to 0.0)	350 (315 to 391)	141 (129 to 153)	3.5 (3.1 to 3.9)	0.8 (0.7 to 0.9)
Other unintentional injuries	147 (138 to 157)	117 (105 to 131)	1.6 (1.5 to 1.7)	0.7 (0.6 to 0.8)	8 682 (8 115 to 9 272)	5 922 (5 288 to 6 704)	80.1 (75.4 to 85.0)	33.0 (29.5 to 37.2)

Cause	Incidence: All-age counts (95% UI) 1990	Incidence: All-age counts (95% UI) 2017	Incidence: Age-standardised rates per 100,000 (95% UI) 1990	Incidence: Age-standardised rates per 100,000 (95% UI) 2017	Prevalence: All-age counts (95% UI) 1990	Prevalence: All-age counts (95% UI) 2017	Prevalence: Age-standardised rates per 100,000 (95% UI) 1990	Prevalence: Age-standardised rates per 100,000 (95% UI) 2017
**Transport and unintentional injuries**	**339 687 (313 334 to 369 579)**	**654 236 (600 009 to 712 299)**	**4 004.1 (3 708.3 to 4 319.9)**	**4 047.5 (3 726.2 to 4 414.3)**	**651 718 (609 582 to 698 709)**	**1 402 299 (1 305 243 to 1 520 101)**	**9 314.8 (8 740.8 to 9 991.7)**	**8 689.0 (8 104.9 to 9 409.7)**
**Transport injuries**	**30 156 (25 589 to 35 707)**	**62 817 (53 306 to 73 692)**	**336.3 (288.5 to 386.4)**	**362.3 (308.2 to 424.5)**	**68 821 (64 224 to 73 561)**	**175 093 (163 005 to 188 057)**	**1 023.8 (957.6 to 1 095.2)**	**1 058.1 (986.6 to 1 135.8)**
Road injuries	28 748 (24 323 to 34 405)	61 330 (51 859 to 72 225)	322.3 (274.9 to 371.7)	353.2 (299.3 to 414.1)	62 255 (58 016 to 66 673)	167 213 (155 334 to 179 994)	943.9 (877.9 to 1 010.7)	1 010.4 (940.4 to 1 087.5)
Pedestrian road injuries	5 485 (4 387 to 6 821)	9 595 (7 542 to 12 063)	66.8 (55.0 to 80.5)	56.2 (44.3 to 70.1)	18 357 (15 709 to 21 623)	41 245 (35 730 to 48 359)	289.1 (251.0 to 336.5)	253.7 (220.8 to 295.0)
Cyclist road injuries	3 831 (2 759 to 5 205)	7 190 (5 288 to 9 624)	39.9 (30.1 to 52.3)	42.2 (31.3 to 55.8)	5 879 (4 932 to 6 948)	14 870 (12 500 to 17 585)	82.7 (70.3 to 95.6)	89.5 (76.0 to 105.2)
Motorcyclist road injuries	3 983 (3 033 to 5 098)	6 649 (4 793 to 9 112)	41.2 (32.6 to 51.0)	37.9 (27.3 to 52.0)	12 965 (11 213 to 15 058)	29 477 (25 152 to 34 912)	186.2 (161.4 to 214.7)	179.9 (148.8 to 205.5)
Motor vehicle road injuries	9 578 (7 585 to 11 805)	29 704 (23 988 to 36 063)	108.6 (87.3 to 131.3)	168.4 (136.8 to 203.4)	15 050 (13 159 to 17 651)	63 282 (55 057 to 74 414)	236.9 (205.4 to 277.2)	380.6 (330.8 to 447.4)
Other road injuries	5 871 (4 257 to 7 688)	8 193 (5 899 to 11 562)	65.8 (50.1 to 81.7)	48.5 (35.5 to 67.7)	10 003 (8 342 to 12 094)	18 339 (15 343 to 22 208)	146.9 (122.6 to 175.6)	112.6 (94.4 to 135.7)
Other transport injuries	1 408 (943 to 2 105)	1 486 (947 to 2 154)	14.1 (10.3 to 19.3)	9.0 (5.8 to 12.9)	6 566 (5 283 to 8 068)	7 881 (6 660 to 9 083)	81.9 (67.7 to 96.8)	47.9 (40.9 to 54.9)
**Unintentional injuries**	**309 531 (284 042 to 337 307)**	**591 419 (539 213 to 649 150)**	**3 667.7 (3 378.3 to 3 989.3)**	**3 685.2 (3 363.1 to 4 038.4)**	**582 897 (541 151 to 630 126)**	**1 227 206 (1 132 760 to 1 345 333)**	**8 291.0 (7 732.8 to 8 962.6)**	**7 630.8 (7 051.7 to 8 337.2)**
Falls	145 077 (126 303 to 165 684)	365 999 (323 946 to 413 096)	1 950.1 (1 743.0 to 2 194.3)	2 353.3 (2 092.4 to 2 644.2)	248 734 (218 324 to 282 756)	730 843 (647 166 to 831 144)	3 907.7 (3 490.3 to 4 463.9)	4 667.6 (4 162.5 to 5 329.2)
Drowning	408 (351 to 479)	294 (230 to 383)	4.2 (3.7 to 4.8)	1.7 (1.4 to 2.3)	1 766 (1 552 to 2 057)	1 773 (1 546 to 2 083)	26.3 (23.4 to 30.1)	11.0 (9.7 to 12.9)
Fire, heat, and hot substances	11 913 (9 888 to 15 807)	12 129 (9 872 to 14 877)	108.8 (84.0 to 128.8)	72.5 (58.9 to 89.2)	113 585 (93 184 to 144 865)	143 447 (117 021 to 186 231)	1 359.6 (1 149.2 to 1 684.4)	843.7 (691.0 to 1 082.6)
Poisonings	3 712 (2 679 to 5 203)	5 345 (3 820 to 7 292)	29.7 (22.0 to 40.6)	32.4 (23.0 to 44.2)	3 264 (2 464 to 4 037)	6 601 (4 831 to 8 395)	34.2 (26.1 to 42.0)	38.0 (27.8 to 48.3)
Poisoning by carbon monoxide	744 (457 to 1 170)	695 (389 to 1 124)	6.5 (4.3 to 9.6)	4.2 (2.4 to 6.7)	511 (391 to 666)	560 (429 to 727)	5.6 (4.3 to 7.1)	3.3 (2.5 to 4.2)
Poisoning by other means	2 968 (2 113 to 4 297)	4 650 (3 292 to 6 425)	23.2 (16.7 to 32.9)	28.2 (19.9 to 39.1)	2 753 (1 981 to 3 505)	6 041 (4 270 to 7 844)	28.6 (20.9 to 36.3)	34.7 (24.6 to 45.0)
Exposure to mechanical forces	68 139 (56 708 to 81 585)	83 594 (70 731 to 98 453)	679.0 (574.4 to 799.2)	476.7 (405.5 to 557.9)	124 704 (108 559 to 144 064)	204 187 (179 602 to 235 454)	1 757.9 (1 549.3 to 2 001.6)	1 215.2 (1 071.1 to 1 395.4)
Unintentional firearm injuries	5 047 (3 584 to 6 841)	6 697 (4 792 to 8 902)	48.2 (35.2 to 63.5)	37.8 (27.4 to 50.1)	17 143 (13 622 to 20 493)	29 644 (23 788 to 35 829)	245.2 (198.6 to 294.6)	177.5 (142.7 to 214.1)
Other exposure to mechanical forces	63 092 (52 500 to 76 350)	76 897 (64 668 to 90 569)	630.8 (535.0 to 743.2)	438.9 (372.0 to 515.9)	107 561 (90 509 to 127 052)	174 544 (147 884 to 205 902)	1 512.7 (1 295.7 to 1 765.9)	1 037.8 (884.9 to 1 222.1)
Adverse effects of medical treatment	7 836 (6 593 to 9 145)	20 156 (17 364 to 23 184)	100.1 (85.7 to 114.8)	122.1 (105.2 to 139.5)	600 (462 to 740)	1 543 (1 190 to 1 899)	7.7 (5.9 to 9.4)	9.3 (7.2 to 11.5)
Animal contact	21 310 (17 007 to 26 937)	24 322 (20 427 to 29 070)	228.1 (185.6 to 267.4)	147.4 (124.5 to 175.2)	21 958 (18 761 to 25 687)	30 720 (26 523 to 35 507)	296.1 (258.1 to 338.1)	187.0 (162.0 to 215.0)
Venomous animal contact	7 289 (5 363 to 9 884)	8 316 (6 395 to 10 615)	75.4 (56.9 to 97.4)	50.0 (38.6 to 63.9)	4 647 (3 795 to 5 502)	6 393 (5 356 to 7 526)	57.2 (47.9 to 66.7)	38.1 (32.1 to 44.6)
Non-venomous animal contact	14 022 (10 807 to 17 897)	16 007 (12 974 to 19 789)	147.8 (119.1 to 181.9)	97.3 (79.4 to 119.9)	17 310 (14 288 to 20 957)	24 327 (20 177 to 29 122)	238.9 (201.8 to 280.8)	149.0 (124.5 to 177.1)
Foreign body	25 782 (15 195 to 40 130)	46 781 (24 415 to 77 365)	307.4 (171.1 to 489.9)	282.7 (148.2 to 466.2)	13 388 (10 431 to 17 288)	15 895 (11 518 to 22 392)	170.8 (137.0 to 216.6)	99.5 (73.2 to 137.8)
Pulmonary aspiration and foreign body in airway	1 057 (757 to 1 415)	621 (445 to 857)	9.3 (7.1 to 12.1)	3.8 (2.7 to 5.2)	749 (538 to 1 016)	544 (438 to 674)	8.3 (6.2 to 10.9)	3.2 (2.6 to 4.0)
Foreign body in eyes	22 037 (11 783 to 36 021)	44 480 (22 161 to 75 140)	273.0 (136.8 to 453.3)	268.6 (134.8 to 450.6)	4 110 (2 010 to 7 280)	9 332 (5 141 to 15 689)	60.9 (34.8 to 98.3)	59.8 (33.9 to 95.8)
Foreign body in other body part	2 688 (1 804 to 4 154)	1 680 (1 224 to 2 249)	25.0 (17.4 to 35.6)	10.3 (7.5 to 13.6)	8 529 (6 717 to 10 941)	6 020 (5 036 to 7 205)	101.6 (82.2 to 128.1)	36.5 (30.7 to 43.5)
Environmental heat and cold exposure	6 321 (5 358 to 7 784)	11 537 (9 912 to 13 464)	67.0 (56.5 to 78.9)	70.3 (60.2 to 81.2)	21 656 (17 529 to 26 329)	44 520 (37 933 to 52 246)	280.5 (235.2 to 330.3)	272.7 (234.8 to 317.4)
Exposure to forces of nature	908 (754 to 1 105)	188 (158 to 225)	8.8 (7.4 to 10.4)	1.1 (0.9 to 1.3)	8 351 (5 900 to 11 767)	10 465 (7 978 to 14 623)	94.3 (67.8 to 131.2)	62.5 (48.8 to 87.5)
Other unintentional injuries	18 125 (14 812 to 22 240)	21 076 (17 808 to 25 014)	194.4 (164.2 to 229.3)	125.1 (105.7 to 147.9)	24 892 (21 201 to 28 977)	37 211 (32 208 to 42 727)	345.9 (299.1 to 395.2)	224.1 (194.6 to 255.8)

Cause	Years lived with disability: All-age counts (95% UI) 1990	Years lived with disability: All-age counts (95% UI) 2017	Years lived with disability: Age-standardised rates per 100,000 (95% UI) 1990	Years lived with disability: Age-standardised rates per 100,000 (95% UI) 2017	Disability adjusted life years: All-age counts (95% UI) 1990	Disability adjusted life years: All-age counts (95% UI) 2017	Disability adjusted life years: Age-standardised rates per 100,000 (95% UI) 1990	Disability adjusted life years: Age-standardised rates per 100,000 (95% UI) 2017
**Transport and unintentional injuries**	**29 054 (21 133 to 38 522)**	**60 532 (43 273 to 82 464)**	**409.8 (298.1 to 543.4)**	**375.4 (268.4 to 508.5)**	**287 983 (272 469 to 304 412)**	**278 112 (260 231 to 306 227)**	**2 994.6 (2 844.2 to 3 169.5)**	**1 640.6 (1 472.7 to 1 807.3)**
**Transport injuries**	**4 719 (3 440 to 6 265)**	**10 619 (7 623 to 14 255)**	**66.6 (48.8 to 88.5)**	**63.2 (45.4 to 84.9)**	**130 027 (122 488 to 137 342)**	**129 579 (113 694 to 143 064)**	**1 316.5 (1 247.1 to 1 382.4)**	**734.4 (645.6 to 809.5)**
Road injuries	4 293 (3 141 to 5 703)	10 201 (7 327 to 13 715)	61.6 (45.1 to 81.5)	60.7 (43.5 to 81.5)	127 013 (119 557 to 134 287)	125 193 (109 607 to 138 375)	1 284.8 (1 217.1 to 1 350.9)	709.7 (623.1 to 782.9)
Pedestrian road injuries	1 320 (939 to 1 786)	2 616 (1 826 to 3 576)	19.6 (14.1 to 26.6)	15.8 (11.1 to 21.7)	69 843 (65 068 to 74 938)	58 765 (52 158 to 64 921)	729.0 (687.4 to 771.2)	337.5 (300.6 to 371.5)
Cyclist road injuries	411 (288 to 545)	889 (616 to 1 183)	5.5 (3.9 to 7.3)	5.3 (3.7 to 7.0)	2 755 (2 485 to 3 049)	4 227 (3 651 to 4 773)	28.8 (26.1 to 31.7)	24.2 (20.9 to 27.3)
Motorcyclist road injuries	909 (639 to 1 224)	1 800 (1 232 to 2 462)	12.5 (8.7 to 16.9)	10.5 (7.2 to 14.4)	16 312 (14 646 to 18 068)	14 741 (12 693 to 16 846)	156.4 (141.5 to 171.4)	81.8 (70.5 to 93.5)
Motor vehicle road injuries	1 076 (776 to 1 432)	3 983 (2 869 to 5 346)	16.0 (11.7 to 21.1)	23.5 (17.0 to 31.4)	34 023 (30 786 to 37 315)	43 294 (36 346 to 48 914)	329.3 (301.4 to 357.5)	242.5 (203.9 to 274.0)
Other road injuries	576 (408 to 785)	912 (646 to 1 253)	8.0 (5.8 to 10.9)	5.5 (3.9 to 7.5)	4 080 (3 371 to 4 721)	4 166 (3 570 to 4 818)	41.3 (35.0 to 47.1)	23.8 (20.4 to 27.5)
Other transport injuries	427 (297 to 581)	418 (296 to 568)	5.0 (3.5 to 6.7)	2.5 (1.8 to 3.4)	3 014 (2 751 to 3 304)	4 386 (3 706 to 5 067)	31.7 (29.0 to 34.4)	24.7 (21.0 to 28.5)
**Unintentional injuries**	**24 334 (17 679 to 32 452)**	**49 913 (35 468 to 68 558)**	**343.2 (249.1 to 456.8)**	**312.3 (223.0 to 426.5)**	**157 955 (147 186 to 169 415)**	**148 533 (130 870 to 167 011)**	**1 678.1 (1 564.0 to 1 804.1)**	**906.2 (799.4 to 1 022.1)**
Falls	14 714 (10 825 to 19 427)	36 798 (26 461 to 49 617)	222.0 (161.4 to 290.6)	234.1 (166.7 to 314.4)	35 707 (31 460 to 40 450)	59 916 (49 279 to 71 839)	506.7 (446.2 to 573.7)	379.2 (312.8 to 454.6)
Drowning	101 (71 to 139)	94 (65 to 132)	1.4 (1.0 to 2.0)	0.6 (0.4 to 0.8)	29 868 (27 191 to 32 916)	15 004 (13 573 to 16 578)	268.1 (247.2 to 290.8)	86.4 (78.0 to 95.5)
Fire, heat, and hot substances	2 441 (1 487 to 3 840)	2 766 (1 600 to 4 538)	28.8 (17.4 to 44.4)	16.2 (9.4 to 26.5)	12 777 (11 335 to 14 556)	7 334 (6 072 to 9 123)	130.1 (116.4 to 148.6)	44.2 (36.7 to 54.6)
Poisonings	386 (234 to 586)	772 (465 to 1 197)	3.9 (2.4 to 5.9)	4.4 (2.7 to 6.8)	6 516 (5 959 to 7 108)	4 057 (3 616 to 4 602)	59.3 (54.8 to 64.1)	23.5 (21.0 to 26.6)
Poisoning by carbon monoxide	55 (33 to 88)	56 (33 to 89)	0.6 (0.3 to 0.9)	0.3 (0.2 to 0.5)	2 884 (2 609 to 3 166)	1 744 (1 568 to 1 946)	26.1 (23.9 to 28.4)	10.1 (9.1 to 11.2)
Poisoning by other means	331 (197 to 511)	716 (425 to 1 112)	3.3 (2.0 to 5.1)	4.1 (2.4 to 6.4)	3 632 (3 278 to 4 016)	2 312 (1 974 to 2 739)	33.1 (30.1 to 36.5)	13.4 (11.5 to 15.9)
Exposure to mechanical forces	2 939 (2 009 to 4 208)	4 382 (2 950 to 6 355)	40.3 (27.6 to 57.5)	26.0 (17.5 to 37.7)	18 353 (16 895 to 20 095)	12 827 (11 141 to 15 049)	193.0 (176.6 to 212.8)	73.9 (64.0 to 86.9)
Unintentional firearm injuries	358 (235 to 544)	577 (374 to 900)	4.9 (3.2 to 7.5)	3.4 (2.2 to 5.3)	7 331 (6 466 to 8 069)	3 483 (2 608 to 4 006)	70.4 (62.2 to 77.1)	19.6 (14.8 to 22.7)
Other exposure to mechanical forces	2 581 (1 763 to 3 697)	3 805 (2 554 to 5 572)	35.4 (24.3 to 50.7)	22.5 (15.2 to 32.9)	11 022 (9 894 to 12 415)	9 344 (7 845 to 11 269)	122.6 (109.1 to 139.8)	54.3 (45.5 to 65.6)
Adverse effects of medical treatment	80 (49 to 121)	206 (127 to 309)	1.0 (0.6 to 1.5)	1.2 (0.8 to 1.9)	4 626 (4 139 to 6 074)	5 841 (4 762 to 10 069)	53.1 (48.5 to 69.6)	36.9 (30.2 to 62.9)
Animal contact	589 (389 to 837)	735 (482 to 1 054)	7.1 (4.7 to 9.9)	4.4 (2.9 to 6.2)	3 943 (3 402 to 4 576)	1 843 (1 552 to 2 168)	36.5 (32.0 to 42.0)	11.1 (9.4 to 13.1)
Venomous animal contact	322 (201 to 479)	395 (252 to 576)	3.4 (2.2 to 5.0)	2.3 (1.5 to 3.3)	2 762 (2 306 to 3 286)	921 (715 to 1 151)	23.9 (20.4 to 28.0)	5.5 (4.3 to 6.9)
Non-venomous animal contact	267 (160 to 425)	340 (197 to 558)	3.6 (2.2 to 5.7)	2.1 (1.2 to 3.4)	1 181 (985 to 1 407)	922 (743 to 1 168)	12.6 (10.6 to 15.2)	5.6 (4.5 to 7.1)
Foreign body	833 (551 to 1 206)	929 (560 to 1 485)	10.3 (6.9 to 14.7)	5.8 (3.5 to 9.2)	33 187 (29 629 to 36 805)	31 628 (27 130 to 37 384)	299.1 (272.5 to 326.2)	193.0 (164.4 to 229.6)
Pulmonary aspiration and foreign body in airway	40 (27 to 55)	23 (16 to 31)	0.4 (0.3 to 0.6)	0.1 (0.1 to 0.2)	31 316 (27 913 to 34 978)	30 139 (25 626 to 35 881)	279.6 (253.2 to 307.1)	184.1 (155.0 to 221.2)
Foreign body in eyes	273 (138 to 508)	626 (327 to 1 118)	4.1 (2.2 to 7.2)	4.0 (2.1 to 7.0)	273 (138 to 508)	626 (327 to 1 118)	4.1 (2.2 to 7.2)	4.0 (2.1 to 7.0)
Foreign body in other body part	520 (355 to 696)	280 (199 to 372)	5.8 (4.1 to 7.7)	1.7 (1.2 to 2.2)	1 598 (1 363 to 1 915)	863 (674 to 1 448)	15.4 (13.1 to 18.4)	4.9 (3.9 to 8.4)
Environmental heat and cold exposure	833 (562 to 1 185)	1 648 (1 092 to 2 346)	10.8 (7.3 to 15.3)	10.1 (6.7 to 14.5)	2 531 (2 208 to 2 928)	2 435 (1 860 to 3 177)	30.5 (26.5 to 35.2)	14.7 (11.2 to 19.1)
Exposure to forces of nature	436 (316 to 607)	355 (264 to 482)	4.9 (3.5 to 6.7)	2.1 (1.6 to 2.8)	786 (658 to 954)	496 (393 to 623)	8.4 (7.0 to 10.2)	2.9 (2.4 to 3.6)
Other unintentional injuries	978 (703 to 1 303)	1 229 (862 to 1 692)	13.2 (9.5 to 17.7)	7.4 (5.2 to 10.2)	9 660 (9 031 to 10 374)	7 151 (6 402 to 7 986)	93.3 (87.4 to 100.0)	40.4 (36.1 to 45.0)

Death, YLL, incidence, prevalence, YLD, and DALY counts and rates per 100,000 for all injuries in Michoacán de Ocampo in 1990 and 2017

Cause	Deaths			
	All-age counts (95% UI)		Age-standardised rates per 100,000 (95% UI)	
	1990	2017	1990	2017
**Transport and unintentional injuries**	**2 252 (2 128 to 2 389)**	**1 889 (1 769 to 2 013)**	**71.4 (69.0 to 74.3)**	**39.4 (36.8 to 42.0)**
**Transport injuries**	**999 (944 to 1 061)**	**897 (828 to 972)**	**31.1 (29.7 to 32.6)**	**18.7 (17.3 to 20.3)**
Road injuries	968 (914 to 1 029)	848 (784 to 919)	30.1 (28.8 to 31.6)	17.7 (16.3 to 19.2)
Pedestrian road injuries	495 (459 to 537)	372 (341 to 405)	15.9 (15.0 to 16.8)	7.8 (7.2 to 8.5)
Cyclist road injuries	21 (19 to 23)	31 (27 to 34)	0.6 (0.6 to 0.7)	0.6 (0.6 to 0.7)
Motorcyclist road injuries	113 (102 to 124)	108 (94 to 123)	3.4 (3.1 to 3.7)	2.2 (1.9 to 2.5)
Motor vehicle road injuries	310 (284 to 339)	314 (284 to 346)	9.3 (8.6 to 10.1)	6.5 (5.9 to 7.2)
Other road injuries	29 (25 to 33)	24 (21 to 28)	0.9 (0.8 to 1.0)	0.5 (0.4 to 0.6)
Other transport injuries	31 (28 to 35)	49 (42 to 55)	1.0 (0.9 to 1.1)	1.0 (0.9 to 1.1)
**Unintentional injuries**	**1 252 (1 173 to 1 343)**	**992 (928 to 1 067)**	**40.3 (38.7 to 42.1)**	**20.7 (19.3 to 22.0)**
Falls	246 (235 to 259)	303 (274 to 327)	11.0 (10.5 to 11.6)	6.3 (5.7 to 6.8)
Drowning	259 (233 to 290)	157 (142 to 171)	7.0 (6.5 to 7.7)	3.3 (3.0 to 3.6)
Fire, heat, and hot substances	93 (85 to 103)	60 (54 to 64)	3.1 (2.9 to 3.3)	1.2 (1.1 to 1.3)
Poisonings	49 (44 to 54)	29 (26 to 32)	1.4 (1.3 to 1.5)	0.6 (0.5 to 0.7)
Poisoning by carbon monoxide	14 (12 to 15)	10 (8 to 11)	0.4 (0.4 to 0.4)	0.2 (0.2 to 0.2)
Poisoning by other means	35 (31 to 39)	20 (17 to 22)	1.0 (0.9 to 1.0)	0.4 (0.4 to 0.5)
Exposure to mechanical forces	187 (176 to 199)	125 (114 to 138)	6.0 (5.7 to 6.3)	2.6 (2.4 to 2.9)
Unintentional firearm injuries	104 (95 to 114)	61 (49 to 70)	3.2 (2.9 to 3.5)	1.3 (1.0 to 1.5)
Other exposure to mechanical forces	83 (75 to 93)	65 (58 to 75)	2.8 (2.5 to 3.0)	1.4 (1.2 to 1.6)
Adverse effects of medical treatment	58 (48 to 64)	63 (57 to 79)	2.2 (1.8 to 2.4)	1.3 (1.2 to 1.7)
Animal contact	76 (64 to 93)	28 (25 to 30)	1.9 (1.7 to 2.2)	0.6 (0.5 to 0.6)
Venomous animal contact	65 (53 to 78)	19 (17 to 22)	1.5 (1.3 to 1.8)	0.4 (0.4 to 0.5)
Non-venomous animal contact	12 (8 to 16)	8 (7 to 10)	0.4 (0.3 to 0.4)	0.2 (0.2 to 0.2)
Foreign body	195 (173 to 221)	176 (157 to 200)	5.0 (4.6 to 5.5)	3.6 (3.3 to 4.1)
Pulmonary aspiration and foreign body in airway	186 (164 to 210)	172 (153 to 195)	4.8 (4.4 to 5.3)	3.6 (3.2 to 4.0)
Foreign body in eyes				
Foreign body in other body part	9 (7 to 11)	4 (3 to 6)	0.2 (0.2 to 0.3)	0.1 (0.1 to 0.1)
Environmental heat and cold exposure	22 (21 to 24)	11 (9 to 12)	0.8 (0.8 to 0.9)	0.2 (0.2 to 0.2)
Exposure to forces of nature	4 (4 to 5)	1 (1 to 1)	0.1 (0.1 to 0.2)	0.0 (0.0 to 0.0)
Other unintentional injuries	62 (58 to 67)	41 (36 to 46)	1.8 (1.7 to 2.0)	0.8 (0.8 to 0.9)

Cause	Years of life lost			
	All-age counts (95% UI)		Age-standardised rates per 100,000 (95% UI)	
	1990	2017	1990	2017
**Transport and unintentional injuries**	**128 032 (118 261 to 139 464)**	**86 668 (80 363 to 93 635)**	**3 311.1 (3 120.3 to 3 528.3)**	**1 796.8 (1 666.4 to 1 939.7)**
**Transport injuries**	**57 398 (53 346 to 62 022)**	**45 254 (41 654 to 49 411)**	**1 533.8 (1 447.9 to 1 632.4)**	**935.4 (861.9 to 1 019.1)**
Road injuries	55 634 (51 674 to 60 214)	42 873 (39 423 to 46 739)	1 485.5 (1 401.4 to 1 582.1)	886.0 (815.0 to 965.6)
Pedestrian road injuries	27 864 (25 067 to 31 155)	17 160 (15 602 to 19 010)	725.9 (668.0 to 790.7)	357.3 (324.9 to 395.0)
Cyclist road injuries	1 184 (1 050 to 1 335)	1 466 (1 303 to 1 662)	33.0 (29.7 to 36.7)	30.5 (27.1 to 34.5)
Motorcyclist road injuries	6 740 (6 026 to 7 478)	6 212 (5 383 to 7 141)	185.4 (168.0 to 203.9)	126.9 (110.1 to 145.5)
Motor vehicle road injuries	18 181 (16 558 to 19 926)	16 830 (15 128 to 18 671)	496.9 (456.3 to 541.2)	346.5 (311.8 to 383.6)
Other road injuries	1 665 (1 371 to 1 948)	1 205 (1 033 to 1 416)	44.4 (37.8 to 51.2)	24.9 (21.3 to 29.1)
Other transport injuries	1 763 (1 574 to 1 970)	2 381 (2 027 to 2 719)	48.3 (43.4 to 53.6)	49.3 (42.0 to 56.4)
**Unintentional injuries**	**70 634 (64 027 to 78 149)**	**41 413 (37 993 to 45 439)**	**1 777.3 (1 652.1 to 1 918.4)**	**861.4 (790.3 to 944.4)**
Falls	8 058 (7 352 to 8 666)	7 147 (6 265 to 7 737)	273.0 (259.8 to 287.9)	151.1 (132.4 to 163.6)
Drowning	16 871 (14 772 to 19 416)	8 628 (7 706 to 9 656)	398.6 (358.9 to 445.3)	178.7 (160.0 to 199.7)
Fire, heat, and hot substances	5 186 (4 511 to 5 983)	2 062 (1 820 to 2 297)	127.4 (114.4 to 142.3)	43.1 (38.1 to 47.9)
Poisonings	3 150 (2 759 to 3 594)	1 484 (1 301 to 1 645)	74.7 (67.2 to 83.2)	30.8 (27.0 to 34.2)
Poisoning by carbon monoxide	850 (739 to 981)	475 (407 to 538)	20.9 (18.6 to 23.7)	9.8 (8.4 to 11.1)
Poisoning by other means	2 300 (2 001 to 2 658)	1 009 (882 to 1 137)	53.8 (48.1 to 60.4)	20.9 (18.3 to 23.6)
Exposure to mechanical forces	10 542 (9 801 to 11 304)	5 855 (5 265 to 6 511)	289.7 (271.7 to 307.5)	121.8 (109.5 to 135.4)
Unintentional firearm injuries	6 053 (5 445 to 6 603)	3 047 (2 449 to 3 556)	164.3 (149.4 to 179.3)	63.1 (50.8 to 73.6)
Other exposure to mechanical forces	4 489 (3 966 to 5 043)	2 809 (2 472 to 3 349)	125.4 (112.7 to 138.4)	58.7 (51.7 to 69.9)
Adverse effects of medical treatment	2 749 (2 191 to 3 159)	2 042 (1 793 to 2 620)	72.3 (58.8 to 80.5)	42.8 (37.7 to 55.0)
Animal contact	5 530 (4 501 to 6 743)	1 263 (1 103 to 1 464)	114.7 (95.4 to 136.9)	26.3 (23.1 to 30.5)
Venomous animal contact	4 842 (3 811 to 6 019)	903 (769 to 1 068)	97.8 (78.9 to 119.3)	18.8 (16.1 to 22.2)
Non-venomous animal contact	688 (465 to 1 001)	360 (305 to 430)	16.9 (11.9 to 22.8)	7.5 (6.4 to 9.0)
Foreign body	13 570 (11 672 to 15 756)	10 402 (8 847 to 12 439)	292.6 (258.9 to 332.3)	214.4 (182.8 to 256.1)
Pulmonary aspiration and foreign body in airway	12 927 (11 096 to 15 051)	10 166 (8 625 to 12 191)	277.4 (244.0 to 316.1)	209.5 (178.2 to 250.7)
Foreign body in eyes				
Foreign body in other body part	644 (454 to 773)	236 (197 to 329)	15.2 (11.0 to 17.8)	4.9 (4.1 to 6.8)
Environmental heat and cold exposure	1 054 (956 to 1 174)	395 (337 to 440)	30.4 (28.2 to 33.1)	8.4 (7.1 to 9.3)
Exposure to forces of nature	257 (214 to 308)	39 (36 to 43)	7.2 (6.0 to 8.8)	0.8 (0.7 to 0.9)
Other unintentional injuries	3 667 (3 368 to 3 980)	2 096 (1 858 to 2 361)	96.7 (90.1 to 104.4)	43.3 (38.5 to 48.8)

Cause	Incidence			
	All-age counts (95% UI)		Age-standardised rates per 100,000 (95% UI)	
	1990	2017	1990	2017
**Transport and unintentional injuries**	**144 110 (132 060 to 156 598)**	**184 266 (166 923 to 202 563)**	**4 431.0 (4 051.8 to 4 829.1)**	**3 877.7 (3 509.4 to 4 267.9)**
**Transport injuries**	**11 449 (9 803 to 13 443)**	**19 176 (16 609 to 21 946)**	**344.9 (299.7 to 395.4)**	**400.5 (347.7 to 457.7)**
Road injuries	10 748 (9 146 to 12 722)	18 564 (15 934 to 21 360)	325.4 (281.1 to 375.7)	387.7 (333.7 to 445.7)
Pedestrian road injuries	2 055 (1 629 to 2 577)	2 377 (1 885 to 2 932)	63.2 (51.9 to 75.9)	50.0 (39.7 to 61.6)
Cyclist road injuries	1 418 (1 041 to 1 863)	2 116 (1 646 to 2 702)	39.4 (30.1 to 50.5)	44.2 (34.3 to 56.3)
Motorcyclist road injuries	1 398 (1 096 to 1 764)	1 950 (1 457 to 2 536)	41.1 (32.5 to 51.2)	40.2 (30.3 to 51.7)
Motor vehicle road injuries	3 748 (3 049 to 4 545)	9 303 (7 776 to 11 031)	118.4 (97.6 to 140.8)	194.2 (162.5 to 229.6)
Other road injuries	2 128 (1 600 to 2 702)	2 818 (2 228 to 3 451)	63.3 (50.0 to 77.4)	59.0 (46.7 to 72.2)
Other transport injuries	701 (523 to 958)	612 (446 to 814)	19.5 (15.6 to 24.9)	12.8 (9.3 to 17.0)
**Unintentional injuries**	**132 661 (121 063 to 145 469)**	**165 090 (147 738 to 183 363)**	**4 086.1 (3 717.7 to 4 484.6)**	**3 477.2 (3 113.1 to 3 866.2)**
Falls	54 973 (48 249 to 62 887)	100 146 (89 158 to 112 104)	1 833.0 (1 631.3 to 2 063.9)	2 112.2 (1 880.5 to 2 367.0)
Drowning	216 (185 to 250)	127 (108 to 152)	5.9 (5.2 to 6.8)	2.7 (2.2 to 3.4)
Fire, heat, and hot substances	5 097 (4 010 to 6 398)	2 547 (1 927 to 3 388)	116.5 (94.8 to 142.1)	53.2 (40.2 to 70.8)
Poisonings	1 493 (1 099 to 2 024)	1 025 (746 to 1 406)	30.8 (23.3 to 40.4)	21.3 (15.5 to 29.3)
Poisoning by carbon monoxide	286 (187 to 434)	202 (115 to 321)	6.6 (4.6 to 9.5)	4.2 (2.4 to 6.7)
Poisoning by other means	1 207 (863 to 1 671)	822 (578 to 1 156)	24.2 (17.8 to 32.8)	17.0 (11.9 to 24.0)
Exposure to mechanical forces	31 705 (27 045 to 37 275)	25 700 (21 661 to 30 404)	899.3 (773.9 to 1 042.8)	536.2 (452.0 to 634.6)
Unintentional firearm injuries	2 920 (2 161 to 3 814)	1 566 (1 147 to 2 114)	82.5 (62.2 to 105.9)	32.5 (23.8 to 43.7)
Other exposure to mechanical forces	28 784 (24 333 to 34 063)	24 133 (20 479 to 28 563)	816.8 (702.3 to 952.7)	503.7 (426.4 to 596.1)
Adverse effects of medical treatment	2 429 (2 024 to 2 848)	3 899 (3 301 to 4 652)	83.4 (70.6 to 97.1)	83.0 (70.2 to 99.2)
Animal contact	14 405 (11 922 to 17 470)	6 460 (5 268 to 7 908)	393.4 (340.5 to 466.6)	135.6 (110.7 to 166.1)
Venomous animal contact	5 407 (4 117 to 7 096)	2 296 (1 728 to 2 995)	148.8 (115.8 to 187.4)	48.3 (36.3 to 62.9)
Non-venomous animal contact	8 998 (7 257 to 11 136)	4 164 (3 249 to 5 230)	249.7 (206.4 to 301.4)	87.4 (68.4 to 110.2)
Foreign body	19 212 (16 673 to 20 648)	19 644 (17 748 to 23 409)	455.4 (201.5 to 746.0)	417.7 (169.0 to 709.7)
Pulmonary aspiration and foreign body in airway	417 (287 to 585)	205 (149 to 280)	9.0 (6.5 to 12.3)	4.3 (3.1 to 5.8)
Foreign body in eyes	11 759 (5 196 to 19 165)	18 932 (6 873 to 32 704)	421.0 (165.3 to 714.6)	402.8 (144.2 to 695.4)
Foreign body in other body part	1 036 (697 to 1 569)	507 (338 to 687)	25.5 (18.0 to 35.8)	10.6 (7.5 to 14.4)
Environmental heat and cold exposure	1 778 (1 273 to 2 460)	692 (515 to 1 007)	50.6 (39.2 to 63.1)	14.5 (10.8 to 20.9)
Exposure to forces of nature	608 (502 to 730)	52 (44 to 62)	17.2 (14.1 to 20.6)	1.1 (0.9 to 1.3)
Other unintentional injuries	6 745 (5 514 to 8 268)	4 807 (3 735 to 6 298)	195.6 (164.4 to 233.0)	100.0 (77.5 to 131.1)

Cause	Prevalence			
	All-age counts (95% UI)		Age-standardised rates per 100,000 (95% UI)	
	1990	2017	1990	2017
**Transport and unintentional injuries**	**269 948 (253 694 to 289 131)**	**373 763 (350 031 to 404 330)**	**10 111.6 (9 535.9 to 10 828.3)**	**7 918.8 (7 414.0 to 8 574.2)**
**Transport injuries**	**27 975 (26 092 to 29 855)**	**56 034 (52 158 to 60 304)**	**1 085.1 (1 015.6 to 1 158.0)**	**1 191.2 (1 108.7 to 1 282.5)**
Road injuries	24 862 (23 131 to 26 626)	52 836 (49 079 to 56 981)	974.0 (907.3 to 1 043.4)	1 123.4 (1 043.6 to 1 212.0)
Pedestrian road injuries	7 903 (6 226 to 8 586)	11 031 (9 594 to 12 868)	283.6 (244.3 to 330.6)	234.7 (204.1 to 274.1)
Cyclist road injuries	2 271 (1 917 to 2 686)	4 637 (3 936 to 5 438)	84.6 (71.8 to 98.1)	98.1 (83.3 to 115.0)
Motorcyclist road injuries	4 976 (4 308 to 5 745)	9 109 (7 755 to 10 712)	194.2 (168.6 to 224.2)	193.1 (164.6 to 227.2)
Motor vehicle road injuries	6 565 (5 751 to 7 624)	21 364 (18 617 to 24 961)	267.5 (233.4 to 310.8)	455.3 (396.6 to 531.8)
Other road injuries	3 748 (3 117 to 4 483)	6 695 (5 623 to 8 048)	144.1 (120.6 to 172.3)	142.2 (119.5 to 171.0)
Other transport injuries	3 113 (2 586 to 3 668)	3 198 (2 729 to 3 687)	111.1 (93.9 to 128.6)	67.8 (57.9 to 78.2)
**Unintentional injuries**	**241 973 (226 112 to 260 333)**	**317 729 (294 675 to 348 283)**	**9 026.5 (8 441.2 to 9 710.0)**	**6 727.6 (6 234.0 to 7 379.8)**
Falls	94 092 (83 320 to 107 597)	194 076 (173 764 to 220 869)	3 680.8 (3 298.0 to 4 202.3)	4 105.9 (3 670.7 to 4 684.8)
Drowning	972 (852 to 1 119)	798 (706 to 918)	37.6 (33.2 to 43.3)	17.0 (15.0 to 19.5)
Fire, heat, and hot substances	45 935 (38 883 to 59 071)	30 049 (24 851 to 39 912)	1 560.2 (1 314.3 to 1 907.0)	633.9 (525.2 to 826.7)
Poisonings	1 229 (935 to 1 513)	1 147 (835 to 1 430)	36.1 (27.8 to 44.2)	24.0 (17.5 to 29.9)
Poisoning by carbon monoxide	185 (141 to 241)	150 (116 to 191)	5.6 (4.3 to 7.3)	3.2 (2.4 to 4.0)
Poisoning by other means	1 043 (752 to 1 321)	996 (700 to 1 275)	30.4 (22.7 to 38.4)	20.8 (14.7 to 26.7)
Exposure to mechanical forces	61 623 (54 265 to 70 479)	64 117 (56 102 to 73 755)	2 373.5 (2 090.5 to 2 697.4)	1 363.7 (1 191.9 to 1 568.8)
Unintentional firearm injuries	10 183 (8 322 to 12 280)	7 639 (6 158 to 9 235)	402.1 (323.5 to 485.2)	162.7 (131.2 to 196.9)
Other exposure to mechanical forces	51 440 (43 594 to 60 780)	56 478 (48 325 to 66 510)	1 971.4 (1 681.2 to 2 316.9)	1 201.0 (1 027.2 to 1 415.1)
Adverse effects of medical treatment	186 (144 to 232)	298 (228 to 377)	6.4 (4.9 to 7.9)	6.4 (4.9 to 8.0)
Animal contact	14 782 (12 685 to 17 296)	7 989 (6 994 to 9 142)	528.2 (459.0 to 601.8)	169.3 (147.9 to 193.4)
Venomous animal contact	3 618 (2 960 to 4 401)	1 702 (1 459 to 2 007)	120.8 (100.5 to 144.5)	35.9 (30.0 to 42.3)
Non-venomous animal contact	11 163 (9 093 to 13 581)	6 287 (5 315 to 7 436)	407.3 (341.4 to 482.0)	133.2 (112.7 to 157.5)
Foreign body	5 815 (4 450 to 7 713)	6 298 (4 961 to 9 127)	208.9 (160.7 to 276.0)	133.8 (92.9 to 194.5)
Pulmonary aspiration and foreign body in airway	274 (195 to 360)	177 (144 to 217)	8.2 (6.0 to 10.3)	3.7 (3.0 to 4.6)
Foreign body in eyes	2 416 (1 252 to 4 096)	4 308 (2 395 to 7 145)	96.8 (54.4 to 158.0)	91.7 (50.9 to 152.0)
Foreign body in other body part	3 125 (2 494 to 3 951)	1 813 (1 521 to 2 187)	103.8 (84.7 to 129.0)	38.3 (32.1 to 46.3)
Environmental heat and cold exposure	6 017 (4 932 to 7 333)	2 656 (2 241 to 3 154)	214.1 (179.8 to 255.4)	56.0 (47.3 to 66.5)
Exposure to forces of nature	850 (618 to 1 141)	1 879 (1 332 to 2 584)	24.4 (18.0 to 32.4)	39.7 (28.1 to 54.4)
Other unintentional injuries	9 471 (8 132 to 10 925)	8 423 (7 255 to 9 656)	356.4 (308.6 to 408.4)	178.3 (153.5 to 204.3)

Cause	Years lived with disability			
	All-age counts (95% UI)		Age-standardised rates per 100,000 (95% UI)	
	1990	2017	1990	2017
**Transport and unintentional injuries**	**11 719 (8 493 to 15 468)**	**16 665 (11 990 to 22 375)**	**433.2 (313.8 to 578.6)**	**352.8 (253.7 to 474.1)**
**Transport injuries**	**1 925 (1 410 to 2 542)**	**3 409 (2 474 to 4 535)**	**72.4 (53.1 to 96.0)**	**72.4 (52.6 to 96.3)**
Road injuries	1 724 (1 266 to 2 296)	3 237 (2 346 to 4 326)	65.5 (48.1 to 86.9)	68.7 (49.8 to 91.9)
Pedestrian road injuries	531 (375 to 723)	704 (496 to 960)	19.9 (14.1 to 26.7)	15.0 (10.6 to 20.4)
Cyclist road injuries	160 (114 to 211)	281 (196 to 374)	5.8 (4.1 to 7.6)	5.9 (4.2 to 7.9)
Motorcyclist road injuries	349 (243 to 470)	561 (385 to 766)	13.3 (9.2 to 17.9)	11.9 (8.1 to 16.2)
Motor vehicle road injuries	471 (342 to 626)	1 354 (979 to 1 807)	18.6 (13.4 to 24.6)	28.8 (20.7 to 38.5)
Other road injuries	214 (154 to 288)	337 (239 to 462)	8.0 (5.8 to 10.7)	7.2 (5.1 to 9.8)
Other transport injuries	201 (143 to 268)	172 (123 to 230)	6.9 (4.9 to 9.1)	3.6 (2.6 to 4.9)
**Unintentional injuries**	**9 794 (7 040 to 13 105)**	**13 256 (9 494 to 17 906)**	**360.8 (260.4 to 483.1)**	**280.4 (200.9 to 379.0)**
Falls	5 652 (4 098 to 7 396)	10 049 (7 285 to 13 376)	215.8 (156.6 to 280.8)	212.5 (154.0 to 283.0)
Drowning	55 (39 to 77)	42 (29 to 58)	2.1 (1.5 to 2.9)	0.9 (0.6 to 1.2)
Fire, heat, and hot substances	1 022 (699 to 1 506)	585 (349 to 958)	32.9 (20.5 to 52.0)	12.3 (7.3 to 20.1)
Poisonings	146 (89 to 221)	132 (78 to 206)	4.1 (2.5 to 6.2)	2.8 (1.6 to 4.3)
Poisoning by carbon monoxide	20 (12 to 32)	15 (9 to 23)	0.6 (0.3 to 0.9)	0.3 (0.2 to 0.5)
Poisoning by other means	126 (75 to 194)	117 (68 to 183)	3.6 (2.2 to 5.5)	2.5 (1.4 to 3.8)
Exposure to mechanical forces	1 462 (1 006 to 2 077)	1 393 (935 to 2 042)	55.3 (38.3 to 78.7)	29.6 (19.8 to 43.4)
Unintentional firearm injuries	218 (144 to 330)	144 (93 to 228)	8.4 (5.5 to 12.7)	3.1 (2.0 to 4.8)
Other exposure to mechanical forces	1 244 (854 to 1 757)	1 249 (843 to 1 826)	46.9 (32.2 to 67.5)	26.5 (17.9 to 38.9)
Adverse effects of medical treatment	25 (15 to 38)	40 (25 to 61)	0.9 (0.5 to 1.3)	0.8 (0.5 to 1.3)
Animal contact	400 (262 to 567)	185 (121 to 266)	13.0 (8.7 to 18.4)	3.9 (2.5 to 5.6)
Venomous animal contact	227 (144 to 335)	95 (58 to 142)	6.7 (4.3 to 9.8)	2.0 (1.2 to 3.0)
Non-venomous animal contact	173 (106 to 263)	90 (54 to 146)	6.2 (3.9 to 9.3)	1.9 (1.1 to 3.1)
Foreign body	369 (240 to 548)	382 (224 to 617)	13.1 (8.5 to 19.2)	8.1 (4.8 to 13.2)
Pulmonary aspiration and foreign body in airway	15 (10 to 21)	8 (5 to 10)	0.4 (0.3 to 0.6)	0.2 (0.1 to 0.2)
Foreign body in eyes	160 (82 to 286)	287 (150 to 502)	6.5 (3.5 to 11.4)	6.1 (3.2 to 10.7)
Foreign body in other body part	194 (136 to 258)	87 (62 to 115)	6.1 (4.4 to 8.1)	1.8 (1.3 to 2.4)
Environmental heat and cold exposure	240 (161 to 339)	101 (66 to 146)	8.5 (5.7 to 11.9)	2.1 (1.4 to 3.1)
Exposure to forces of nature	46 (32 to 64)	62 (45 to 86)	1.3 (0.9 to 1.8)	1.3 (0.9 to 1.8)
Other unintentional injuries	377 (271 to 500)	286 (202 to 392)	13.9 (9.9 to 18.5)	6.0 (4.3 to 8.3)

Cause	Disability adjusted life years			
	All-age counts (95% UI)		Age-standardised rates per 100,000 (95% UI)	
	1990	2017	1990	2017
**Transport and unintentional injuries**	**139 750 (128 513 to 151 750)**	**103 333 (95 096 to 112 500)**	**3 744.3 (3 510.2 to 3 988.9)**	**2 149.6 (1 977.1 to 2 340.8)**
**Transport injuries**	**59 323 (55 238 to 64 017)**	**48 663 (44 770 to 52 738)**	**1 606.2 (1 518.8 to 1 715.3)**	**1 007.8 (927.5 to 1 090.7)**
Road injuries	57 358 (53 307 to 62 013)	46 110 (42 321 to 50 039)	1 551.1 (1 466.7 to 1 656.7)	954.8 (877.0 to 1 034.5)
Pedestrian road injuries	28 395 (25 594 to 31 699)	17 864 (16 238 to 19 649)	745.8 (686.8 to 811.0)	372.2 (338.8 to 409.3)
Cyclist road injuries	1 344 (1 210 to 1 501)	1 747 (1 551 to 1 953)	38.8 (35.3 to 42.7)	36.5 (32.4 to 40.7)
Motorcyclist road injuries	7 089 (6 382 to 7 820)	6 772 (5 903 to 7 702)	198.7 (180.2 to 217.4)	138.8 (121.1 to 157.4)
Motor vehicle road injuries	18 652 (17 028 to 20 447)	18 184 (16 525 to 20 095)	515.5 (474.5 to 561.7)	375.3 (341.4 to 414.2)
Other road injuries	1 879 (1 593 to 2 178)	1 543 (1 328 to 1 785)	52.4 (45.3 to 59.5)	32.1 (27.7 to 37.1)
Other transport injuries	1 965 (1 763 to 2 179)	2 553 (2 196 to 2 885)	55.1 (50.1 to 60.5)	53.0 (45.6 to 59.9)
**Unintentional injuries**	**80 428 (72 994 to 88 716)**	**54 670 (49 207 to 60 669)**	**2 138.1 (1 973.4 to 2 318.4)**	**1 141.8 (1 026.1 to 1 267.1)**
Falls	13 711 (11 941 to 15 521)	17 196 (14 213 to 20 501)	488.8 (427.0 to 556.7)	363.6 (300.4 to 433.3)
Drowning	16 927 (14 831 to 19 467)	8 670 (7 737 to 9 692)	400.7 (360.8 to 447.5)	179.6 (160.7 to 200.6)
Fire, heat, and hot substances	6 208 (5 382 to 7 188)	2 647 (2 310 to 3 054)	160.3 (141.8 to 182.1)	55.4 (48.4 to 64.0)
Poisonings	3 296 (2 917 to 3 746)	1 616 (1 424 to 1 795)	78.9 (71.2 to 87.7)	33.5 (29.6 to 37.3)
Poisoning by carbon monoxide	870 (760 to 1 001)	490 (422 to 554)	21.5 (19.2 to 24.2)	10.1 (8.8 to 11.5)
Poisoning by other means	2 426 (2 139 to 2 790)	1 126 (984 to 1 263)	57.4 (51.7 to 64.2)	23.4 (20.5 to 26.2)
Exposure to mechanical forces	12 003 (11 115 to 12 996)	7 248 (6 511 to 8 089)	345.0 (319.3 to 373.6)	151.4 (136.0 to 169.4)
Unintentional firearm injuries	6 271 (5 662 to 6 809)	3 190 (2 568 to 3 705)	172.7 (157.1 to 187.3)	66.1 (53.2 to 76.7)
Other exposure to mechanical forces	5 732 (5 056 to 6 498)	4 057 (3 485 to 4 831)	172.3 (151.5 to 197.5)	85.2 (73.3 to 101.6)
Adverse effects of medical treatment	2 774 (2 219 to 3 184)	2 082 (1 830 to 2 665)	73.1 (59.6 to 81.3)	43.7 (38.4 to 55.8)
Animal contact	5 931 (4 886 to 7 161)	1 448 (1 283 to 1 657)	127.7 (108.5 to 151.0)	30.2 (26.8 to 34.6)
Venomous animal contact	5 069 (4 022 to 6 252)	998 (860 to 1 173)	104.5 (85.1 to 126.9)	20.8 (18.0 to 24.4)
Non-venomous animal contact	862 (616 to 1 183)	449 (380 to 544)	23.2 (16.9 to 30.2)	9.4 (8.0 to 11.4)
Foreign body	13 940 (12 043 to 16 173)	10 784 (9 239 to 12 822)	305.6 (270.5 to 346.6)	222.5 (190.9 to 264.2)
Pulmonary aspiration and foreign body in airway	12 942 (11 111 to 15 065)	10 174 (8 633 to 12 199)	277.8 (244.4 to 316.5)	209.6 (178.4 to 250.9)
Foreign body in eyes	160 (82 to 286)	287 (150 to 502)	6.5 (3.5 to 11.4)	6.1 (3.2 to 10.7)
Foreign body in other body part	838 (644 to 984)	323 (272 to 419)	21.3 (16.8 to 24.7)	6.7 (5.7 to 8.7)
Environmental heat and cold exposure	1 294 (1 158 to 1 434)	497 (428 to 561)	38.9 (35.1 to 42.7)	10.5 (9.0 to 11.8)
Exposure to forces of nature	303 (255 to 355)	101 (84 to 125)	8.5 (7.2 to 10.1)	2.1 (1.8 to 2.6)
Other unintentional injuries	4 043 (3 727 to 4 383)	2 382 (2 136 to 2 675)	110.6 (102.2 to 119.7)	49.4 (44.3 to 55.4)

Death, YLL, incidence, prevalence, YLD, and DALY counts and rates per 100,000 for all injuries in Morelos in 1990 and 2017

Cause	Deaths: All-age counts (95% UI) 1990	Deaths: All-age counts (95% UI) 2017	Deaths: Age-standardised rates per 100,000 (95% UI) 1990	Deaths: Age-standardised rates per 100,000 (95% UI) 2017
**Transport and unintentional injuries**	**662 (627 to 683)**	**785 (729 to 838)**	**63.6 (61.8 to 65.9)**	**39.6 (36.9 to 42.2)**
**Transport injuries**	**283 (268 to 300)**	**325 (288 to 358)**	**26.1 (24.9 to 27.4)**	**16.0 (14.2 to 17.6)**
Road injuries	275 (260 to 291)	309 (274 to 340)	25.3 (24.1 to 26.6)	15.3 (13.5 to 16.8)
Pedestrian road injuries	142 (133 to 152)	155 (140 to 172)	14.0 (13.3 to 14.8)	7.7 (7.0 to 8.6)
Cyclist road injuries	5 (4 to 5)	10 (8 to 11)	0.4 (0.4 to 0.5)	0.5 (0.4 to 0.5)
Motorcyclist road injuries	39 (35 to 43)	38 (32 to 44)	3.2 (2.9 to 3.6)	1.8 (1.6 to 2.1)
Motor vehicle road injuries	83 (76 to 91)	100 (83 to 113)	7.1 (6.5 to 7.7)	4.9 (4.1 to 5.5)
Other road injuries	6 (5 to 7)	7 (6 to 8)	0.5 (0.5 to 0.6)	0.4 (0.3 to 0.4)
Other transport injuries	8 (7 to 9)	16 (13 to 19)	0.8 (0.7 to 0.8)	0.8 (0.6 to 0.9)
**Unintentional injuries**	**369 (352 to 389)**	**460 (434 to 486)**	**37.6 (36.3 to 39.0)**	**23.6 (22.3 to 24.9)**
Falls	90 (85 to 94)	140 (126 to 152)	12.1 (11.5 to 12.8)	7.4 (6.7 to 8.0)
Drowning	76 (70 to 83)	55 (50 to 60)	6.3 (5.9 to 6.8)	2.7 (2.5 to 3.0)
Fire, heat, and hot substances	25 (23 to 27)	24 (22 to 26)	2.6 (2.5 to 2.8)	1.2 (1.1 to 1.3)
Poisonings	15 (14 to 17)	13 (11 to 14)	1.3 (1.2 to 1.4)	0.6 (0.6 to 0.7)
Poisoning by carbon monoxide	3 (3 to 4)	3 (3 to 4)	0.3 (0.3 to 0.3)	0.2 (0.1 to 0.2)
Poisoning by other means	12 (11 to 13)	10 (8 to 11)	1.0 (0.9 to 1.1)	0.5 (0.4 to 0.5)
Exposure to mechanical forces	54 (51 to 58)	41 (37 to 45)	5.1 (4.8 to 5.4)	2.0 (1.8 to 2.2)
Unintentional firearm injuries	28 (26 to 32)	16 (13 to 19)	2.5 (2.3 to 2.9)	0.8 (0.6 to 0.9)
Other exposure to mechanical forces	26 (22 to 28)	24 (22 to 28)	2.6 (2.2 to 2.8)	1.2 (1.1 to 1.4)
Adverse effects of medical treatment	19 (16 to 21)	30 (27 to 33)	2.2 (1.9 to 2.4)	1.6 (1.4 to 2.0)
Animal contact	18 (15 to 20)	11 (10 to 12)	1.5 (1.3 to 1.7)	0.6 (0.5 to 0.6)
Venomous animal contact	14 (12 to 17)	8 (7 to 9)	1.2 (1.0 to 1.4)	0.4 (0.4 to 0.5)
Non venomous animal contact	3 (2 to 4)	3 (3 to 4)	0.3 (0.2 to 0.4)	0.2 (0.1 to 0.2)
Foreign body	50 (45 to 55)	55 (50 to 61)	4.2 (3.8 to 4.5)	2.8 (2.6 to 3.1)
Pulmonary aspiration and foreign body in airway	47 (42 to 53)	54 (49 to 59)	4.0 (3.6 to 4.3)	2.8 (2.5 to 3.0)
Foreign body in eyes				
Foreign body in other body part	2 (2 to 3)	1 (1 to 2)	0.2 (0.1 to 0.2)	0.1 (0.1 to 0.1)
Environmental heat and cold exposure	6 (6 to 6)	4 (4 to 5)	0.7 (0.7 to 0.7)	0.2 (0.2 to 0.2)
Exposure to forces of nature	2 (1 to 2)	71 (66 to 77)	0.1 (0.1 to 0.2)	3.6 (3.3 to 3.9)
Other unintentional injuries	15 (14 to 17)	15 (13 to 17)	1.3 (1.2 to 1.5)	0.7 (0.6 to 0.8)

Cause	Years of life lost: All-age counts (95% UI) 1990	Years of life lost: All-age counts (95% UI) 2017	Years of life lost: Age-standardised rates per 100,000 (95% UI) 1990	Years of life lost: Age-standardised rates per 100,000 (95% UI) 2017
**Transport and unintentional injuries**	**34 560 (32 697 to 36 897)**	**32 850 (30 262 to 35 398)**	**2 760.0 (2 637.7 to 2 909.4)**	**1 630.1 (1 502.1 to 1 755.1)**
**Transport injuries**	**15 412 (14 496 to 16 496)**	**15 215 (13 442 to 16 931)**	**1 236.5 (1 171.7 to 1 315.2)**	**745.0 (659.4 to 828.4)**
Road injuries	14 973 (14 085 to 16 021)	14 464 (12 793 to 16 069)	1 201.0 (1 137.0 to 1 277.2)	708.4 (627.9 to 786.9)
Pedestrian road injuries	7 380 (6 834 to 8 040)	6 515 (5 841 to 7 250)	604.2 (566.3 to 647.6)	321.3 (288.4 to 357.5)
Cyclist road injuries	269 (239 to 304)	430 (373 to 495)	21.9 (19.7 to 24.3)	21.0 (18.2 to 24.2)
Motorcyclist road injuries	2 271 (2 025 to 2 559)	2 101 (1 765 to 2 484)	176.7 (159.1 to 197.2)	101.9 (85.7 to 120.3)
Motor vehicle road injuries	4 713 (4 286 to 5 233)	5 075 (4 230 to 5 828)	371.5 (339.7 to 409.0)	247.4 (207.0 to 283.5)
Other road injuries	340 (283 to 402)	343 (286 to 407)	26.7 (22.6 to 31.4)	16.7 (14.0 to 19.8)
Other transport injuries	439 (393 to 498)	751 (611 to 888)	35.5 (32.0 to 39.8)	36.6 (29.9 to 43.4)
**Unintentional injuries**	**19 148 (17 741 to 20 696)**	**17 635 (16 589 to 18 797)**	**1 523.5 (1 437.4 to 1 618.7)**	**885.1 (831.3 to 945.5)**
Falls	2 921 (2 747 to 3 118)	3 247 (2 898 to 3 542)	299.5 (284.5 to 315.7)	163.3 (146.1 to 177.8)
Drowning	4 698 (4 249 to 5 280)	2 740 (2 484 to 3 034)	342.8 (315.2 to 375.6)	136.5 (123.3 to 151.3)
Fire, heat, and hot substances	1 254 (1 117 to 1 416)	741 (663 to 808)	99.2 (90.4 to 109.0)	37.3 (33.4 to 40.7)
Poisonings	931 (837 to 1 039)	600 (531 to 667)	68.8 (62.6 to 75.7)	29.8 (26.4 to 33.2)
Poisoning by carbon monoxide	199 (175 to 227)	156 (133 to 180)	15.0 (13.3 to 17.0)	7.7 (6.6 to 8.9)
Poisoning by other means	732 (657 to 824)	444 (392 to 495)	53.8 (48.9 to 59.5)	22.1 (19.6 to 24.7)
Exposure to mechanical forces	2 897 (2 675 to 3 129)	1 759 (1 587 to 1 955)	238.0 (221.6 to 255.1)	86.1 (77.9 to 95.6)
Unintentional firearm injuries	1 561 (1 406 to 1 752)	778 (621 to 897)	126.2 (114.3 to 142.3)	37.9 (30.3 to 43.6)
Other exposure to mechanical forces	1 336 (1 141 to 1 494)	981 (867 to 1 148)	111.9 (95.4 to 123.8)	48.2 (42.7 to 56.1)
Adverse effects of medical treatment	892 (686 to 950)	859 (755 to 1 159)	70.1 (59.0 to 77.8)	43.9 (38.4 to 59.3)
Animal contact	1 142 (951 to 1 381)	445 (398 to 496)	79.2 (67.8 to 93.6)	22.7 (20.2 to 25.3)
Venomous animal contact	972 (782 to 1 205)	322 (282 to 367)	66.0 (54.6 to 80.1)	16.5 (14.4 to 18.9)
Non venomous animal contact	169 (118 to 220)	123 (105 to 149)	13.2 (9.1 to 16.4)	6.2 (5.2 to 7.5)
Foreign body	3 239 (2 826 to 3 713)	2 680 (2 355 to 3 068)	226.0 (201.0 to 254.3)	137.9 (120.5 to 159.7)
Pulmonary aspiration and foreign body in airway	3 086 (2 680 to 3 548)	2 602 (2 282 to 2 980)	214.9 (190.4 to 242.9)	134.0 (116.9 to 155.2)
Foreign body in eyes				
Foreign body in other body part	154 (116 to 182)	79 (65 to 123)	11.1 (8.5 to 12.9)	3.9 (3.2 to 6.2)
Environmental heat and cold exposure	267 (245 to 290)	151 (128 to 166)	24.1 (22.4 to 25.9)	7.4 (6.3 to 8.1)
Exposure to forces of nature	92 (72 to 121)	3 680 (3 356 to 4 043)	7.4 (5.8 to 10.0)	184.4 (168.2 to 202.9)
Other unintentional injuries	881 (802 to 969)	732 (637 to 841)	68.5 (62.7 to 75.3)	35.7 (31.0 to 40.9)

Cause	Incidence: All-age counts (95% UI) 1990	Incidence: All-age counts (95% UI) 2017	Incidence: Age-standardised rates per 100,000 (95% UI) 1990	Incidence: Age-standardised rates per 100,000 (95% UI) 2017
**Transport and unintentional injuries**	**45 856 (42 701 to 49 103)**	**76 146 (70 448 to 82 101)**	**4 122.1 (3 851.4 to 4 408.6)**	**3 856.7 (3 571.8 to 4 163.5)**
**Transport injuries**	**3 737 (3 207 to 4 336)**	**7 613 (6 554 to 8 772)**	**326.7 (282.8 to 373.4)**	**375.8 (324.3 to 433.0)**
Road injuries	3 529 (3 012 to 4 127)	7 390 (6 328 to 8 529)	310.1 (265.4 to 355.5)	364.5 (313.3 to 420.8)
Pedestrian road injuries	658 (532 to 815)	1 008 (788 to 1 244)	61.1 (50.6 to 73.8)	50.0 (39.2 to 61.5)
Cyclist road injuries	508 (377 to 654)	998 (777 to 1 276)	41.2 (32.1 to 52.7)	49.9 (38.7 to 64.0)
Motorcyclist road injuries	512 (403 to 647)	844 (684 to 1 075)	42.5 (33.8 to 52.6)	41.4 (31.3 to 52.8)
Motor vehicle road injuries	1 181 (946 to 1 435)	3 235 (2 617 to 3 898)	106.1 (85.8 to 127.8)	158.5 (128.3 to 191.0)
Other road injuries	677 (533 to 860)	1 305 (1 022 to 1 596)	59.2 (46.8 to 72.2)	64.7 (50.9 to 79.0)
Other transport injuries	207 (151 to 286)	223 (169 to 292)	16.6 (12.9 to 21.4)	11.3 (8.5 to 14.8)
**Unintentional injuries**	**42 119 (38 939 to 45 519)**	**68 534 (63 390 to 74 491)**	**3 795.4 (3 521.3 to 4 082.4)**	**3 480.9 (3 215.0 to 3 781.6)**
Falls	18 733 (16 480 to 21 329)	39 092 (34 739 to 44 296)	1 867.9 (1 662.1 to 2 099.2)	2006.8 (1 781.7 to 2 272.5)
Drowning	68 (59 to 79)	47 (38 to 58)	5.5 (4.8 to 6.3)	2.3 (1.9 to 2.9)
Fire, heat, and hot substances	1 506 (1 399 to 1 903)	1 280 (957 to 1 596)	105.4 (86.1 to 129.8)	63.7 (49.4 to 83.6)
Poisonings	510 (373 to 713)	454 (320 to 646)	32.9 (24.6 to 45.0)	23.8 (16.7 to 34.0)
Poisoning by carbon monoxide	107 (66 to 165)	117 (63 to 195)	7.5 (5.0 to 10.9)	6.0 (3.2 to 10.2)
Poisoning by other means	403 (288 to 577)	337 (221 to 498)	25.4 (18.4 to 35.5)	17.8 (11.7 to 26.4)
Exposure to mechanical forces	10 277 (8 786 to 12 125)	10 977 (9 404 to 12 808)	841.2 (724.0 to 977.0)	544.1 (467.4 to 634.9)
Unintentional firearm injuries	893 (657 to 1 177)	546 (398 to 726)	71.8 (54.1 to 93.7)	27.0 (19.7 to 35.8)
Other exposure to mechanical forces	9 386 (7 999 to 11 108)	10 431 (8 904 to 12 157)	769.4 (662.6 to 895.2)	517.1 (442.4 to 603.0)
Adverse effects of medical treatment	875 (725 to 1 039)	1 712 (1 421 to 2 099)	86.7 (72.9 to 102.3)	85.1 (70.7 to 104.5)
Animal contact	4 493 (3 744 to 5 462)	2 868 (2 330 to 3 488)	374.2 (320.4 to 436.8)	145.0 (117.0 to 177.0)
Venomous animal contact	1 789 (1 379 to 2 323)	1 031 (763 to 1 357)	148.3 (115.8 to 186.7)	52.1 (38.6 to 68.3)
Non venomous animal contact	2 704 (2 166 to 3 389)	1 838 (1 435 to 2 330)	225.8 (185.2 to 274.9)	93.0 (72.7 to 117.3)
Foreign body	2 818 (1 923 to 3 858)	4 449 (2 839 to 6 246)	246.5 (165.8 to 346.2)	222.6 (144.1 to 312.3)
Pulmonary aspiration and foreign body in airway	136 (97 to 187)	74 (55 to 98)	9.1 (6.7 to 12.2)	3.8 (2.8 to 5.1)
Foreign body in eyes	2 285 (1 413 to 3 354)	4 122 (2 530 to 5 949)	208.2 (129.8 to 307.7)	206.2 (128.6 to 297.0)
Foreign body in other body part	398 (260 to 619)	247 (157 to 350)	29.2 (19.8 to 42.7)	12.5 (8.0 to 17.9)
Environmental heat and cold exposure	583 (413 to 797)	426 (299 to 618)	49.3 (37.4 to 62.6)	21.3 (15.5 to 31.9)
Exposure to forces of nature	229 (178 to 292)	4 896 (4 132 to 5 878)	18.5 (14.2 to 23.5)	245.6 (207.2 to 295.9)
Other unintentional injuries	2 021 (1 652 to 2 483)	2 389 (2 000 to 2 918)	167.5 (133.6 to 201.7)	120.0 (100.1 to 146.5)

Cause	Prevalence: All-age counts (95% UI) 1990	Prevalence: All-age counts (95% UI) 2017	Prevalence: Age-standardised rates per 100,000 (95% UI) 1990	Prevalence: Age-standardised rates per 100,000 (95% UI) 2017
**Transport and unintentional injuries**	**89 969 (84 481 to 96 258)**	**155 480 (145 558 to 168 245)**	**9 629.5 (9 065.9 to 10 320.4)**	**7 752.5 (7 259.1 to 8 387.8)**
**Transport injuries**	**9 221 (8 601 to 9 816)**	**22 339 (20 857 to 23 937)**	**1 011.5 (947.2 to 1 079.9)**	**1 099.7 (1 027.0 to 1 178.5)**
Road injuries	8 261 (7 682 to 8 844)	21 165 (19 661 to 22 721)	918.3 (856.1 to 983.6)	1 041.5 (968.1 to 1 118.5)
Pedestrian road injuries	2 375 (2 097 to 2 802)	4 606 (4 021 to 5 388)	268.5 (234.1 to 312.6)	228.0 (199.3 to 266.1)
Cyclist road injuries	824 (693 to 976)	2 191 (1 859 to 2 564)	86.8 (73.4 to 100.9)	108.3 (92.0 to 126.2)
Motorcyclist road injuries	1 799 (1 554 to 2 079)	3 896 (3 327 to 4 573)	195.2 (168.5 to 225.2)	190.4 (162.8 to 223.5)
Motor vehicle road injuries	2 041 (1 784 to 2 380)	7 391 (6 428 to 8 669)	234.9 (203.9 to 274.5)	362.9 (315.6 to 425.2)
Other road injuries	1 222 (1 015 to 1 471)	3 080 (2 562 to 3 728)	133.1 (110.7 to 159.7)	152.0 (126.6 to 183.6)
Other transport injuries	960 (793 to 1 151)	1 174 (1 009 to 1 347)	95.2 (80.1 to 111.4)	58.2 (50.2 to 66.8)
**Unintentional injuries**	**80 748 (75 377 to 86 941)**	**133 140 (123 442 to 145 611)**	**8 616.0 (8 066.2 to 9 278.1)**	**6 652.8 (6 170.6 to 7 286.2)**
Falls	32 966 (29 130 to 37 645)	76 228 (68 022 to 86 922)	3 741.4 (3 339.1 to 4 292.9)	3 845.3 (3 437.7 to 4 380.5)
Drowning	315 (276 to 363)	295 (260 to 339)	34.4 (30.3 to 39.2)	14.7 (12.9 to 16.8)
Fire, heat, and hot substances	14 700 (12 249 to 18 494)	15 222 (12 496 to 20 041)	1 391.9 (1 173.9 to 1 732.6)	751.5 (617.0 to 991.0)
Poisonings	453 (338 to 559)	541 (400 to 677)	38.3 (28.9 to 47.0)	26.7 (19.7 to 33.5)
Poisoning by carbon monoxide	75 (58 to 98)	96 (74 to 123)	6.5 (5.1 to 8.4)	4.8 (3.7 to 6.1)
Poisoning by other means	378 (270 to 482)	444 (313 to 573)	31.8 (22.8 to 40.5)	22.0 (15.5 to 28.3)
Exposure to mechanical forces	20 285 (17 869 to 23 272)	27 826 (24 419 to 32 010)	2 193.5 (1 939.0 to 2 504.9)	1 367.3 (1 200.2 to 1 571.4)
Unintentional firearm injuries	3 191 (2 567 to 3 855)	2 800 (2 262 to 3 385)	353.6 (284.3 to 426.8)	137.6 (111.4 to 166.2)
Other exposure to mechanical forces	17 094 (14 535 to 20 272)	25 026 (21 613 to 29 252)	1 841.9 (1 578.2 to 2 174.4)	1 229.8 (1 063.2 to 1 437.0)
Adverse effects of medical treatment	67 (51 to 84)	131 (99 to 170)	6.6 (5.1 to 8.3)	6.5 (4.9 to 8.5)
Animal contact	4 741 (4 096 to 5 494)	3 570 (3 117 to 4 061)	487.4 (428.9 to 553.4)	177.6 (155.1 to 201.9)
Venomous animal contact	1 247 (1 029 to 1 504)	736 (621 to 861)	121.3 (101.3 to 143.3)	36.6 (30.9 to 42.9)
Non venomous animal contact	3 494 (2 868 to 4 217)	2 834 (2 394 to 3 315)	366.1 (307.6 to 430.9)	141.0 (119.2 to 164.8)
Foreign body	1 820 (1 468 to 2 272)	1 861 (1 460 to 2 381)	174.5 (143.5 to 214.1)	93.1 (72.9 to 118.4)
Pulmonary aspiration and foreign body in airway	97 (68 to 134)	65 (53 to 79)	8.4 (6.0 to 11.3)	3.2 (2.6 to 3.9)
Foreign body in eyes	443 (287 to 704)	903 (528 to 1 418)	46.2 (27.6 to 70.9)	45.5 (26.6 to 71.1)
Foreign body in other body part	1 280 (1 012 to 1 652)	893 (754 to 1 061)	119.9 (96.3 to 150.8)	44.3 (37.5 to 52.6)
Environmental heat and cold exposure	2 118 (1 714 to 2 584)	1 785 (1 454 to 2 075)	212.1 (176.5 to 252.9)	86.6 (72.7 to 103.6)
Exposure to forces of nature	323 (236 to 433)	1 502 (1 140 to 1 971)	27.7 (20.6 to 37.0)	74.1 (56.3 to 97.3)
Other unintentional injuries	2 959 (2 521 to 3 424)	4 228 (3 686 to 4 819)	303.1 (266.1 to 352.4)	209.2 (182.5 to 237.6)

Cause	Years lived with disability: All-age counts (95% UI) 1990	Years lived with disability: All-age counts (95% UI) 2017	Years lived with disability: Age-standardised rates per 100,000 (95% UI) 1990	Years lived with disability: Age-standardised rates per 100,000 (95% UI) 2017
**Transport and unintentional injuries**	**3 887 (2 830 to 5 157)**	**6 662 (4 791 to 8 946)**	**411.1 (299.7 to 545.4)**	**332.7 (239.3 to 446.7)**
**Transport injuries**	**625 (454 to 828)**	**1 335 (974 to 1 778)**	**66.4 (48.5 to 88.0)**	**65.5 (47.8 to 87.1)**
Road injuries	563 (413 to 748)	1 274 (927 to 1 707)	60.6 (44.3 to 80.3)	62.5 (45.4 to 83.5)
Pedestrian road injuries	169 (119 to 229)	290 (203 to 400)	18.4 (13.1 to 24.9)	14.3 (10.0 to 19.7)
Cyclist road injuries	57 (40 to 75)	130 (92 to 173)	5.8 (4.1 to 7.6)	6.4 (4.5 to 8.5)
Motorcyclist road injuries	124 (88 to 169)	237 (162 to 324)	13.1 (9.2 to 17.7)	11.6 (7.9 to 15.8)
Motor vehicle road injuries	144 (104 to 192)	462 (333 to 622)	16.0 (11.6 to 21.1)	22.6 (16.3 to 30.4)
Other road injuries	69 (50 to 95)	155 (110 to 213)	7.3 (5.3 to 9.8)	7.6 (5.4 to 10.5)
Other transport injuries	61 (43 to 81)	62 (44 to 82)	5.8 (4.1 to 7.7)	3.0 (2.2 to 4.1)
**Unintentional injuries**	**3 261 (2 347 to 4 318)**	**5 327 (3 799 to 7 171)**	**344.8 (247.4 to 460.4)**	**267.2 (190.6 to 359.6)**
Falls	1 939 (1 426 to 2 542)	3 864 (2 763 to 5 099)	214.5 (156.6 to 281.1)	194.9 (139.2 to 258.9)
Drowning	18 (12 to 25)	15 (11 to 21)	1.9 (1.3 to 2.6)	0.8 (0.5 to 1.0)
Fire, heat, and hot substances	314 (194 to 494)	293 (170 to 477)	28.9 (17.8 to 45.3)	14.5 (8.4 to 23.6)
Poisonings	54 (33 to 80)	62 (37 to 96)	4.4 (2.7 to 6.6)	3.1 (1.8 to 4.7)
Poisoning by carbon monoxide	8 (5 to 13)	10 (6 to 15)	0.7 (0.4 to 1.1)	0.5 (0.3 to 0.7)
Poisoning by other means	45 (27 to 70)	52 (31 to 82)	3.7 (2.2 to 5.7)	2.6 (1.5 to 4.0)
Exposure to mechanical forces	476 (329 to 680)	596 (399 to 869)	50.5 (34.9 to 72.2)	29.3 (19.6 to 42.6)
Unintentional firearm injuries	67 (44 to 101)	52 (33 to 82)	7.2 (4.7 to 10.9)	2.5 (1.6 to 4.0)
Other exposure to mechanical forces	409 (280 to 587)	545 (365 to 794)	43.3 (29.8 to 62.5)	26.8 (17.9 to 39.0)
Adverse effects of medical treatment	9 (5 to 14)	17 (11 to 27)	0.9 (0.5 to 1.3)	0.9 (0.5 to 1.3)
Animal contact	127 (84 to 178)	80 (53 to 114)	11.8 (8.0 to 16.7)	3.9 (2.6 to 5.7)
Venomous animal contact	73 (47 to 106)	39 (25 to 58)	6.3 (4.1 to 9.0)	2.0 (1.2 to 2.9)
Non venomous animal contact	53 (32 to 86)	40 (24 to 64)	5.5 (3.4 to 8.7)	2.0 (1.2 to 3.2)
Foreign body	112 (77 to 157)	105 (67 to 156)	10.4 (7.3 to 14.4)	5.2 (3.4 to 7.8)
Pulmonary aspiration and foreign body in airway	5 (3 to 7)	3 (2 to 4)	0.4 (0.3 to 0.6)	0.1 (0.1 to 0.2)
Foreign body in eyes	30 (15 to 51)	61 (34 to 99)	3.1 (1.7 to 5.1)	3.1 (1.7 to 5.0)
Foreign body in other body part	77 (54 to 104)	41 (30 to 54)	6.9 (4.8 to 9.2)	2.1 (1.5 to 2.7)
Environmental heat and cold exposure	83 (54 to 117)	66 (43 to 94)	8.2 (5.5 to 11.6)	3.3 (2.1 to 4.7)
Exposure to forces of nature	17 (12 to 23)	89 (61 to 128)	1.4 (1.0 to 2.0)	4.4 (3.0 to 6.4)
Other unintentional injuries	115 (82 to 154)	140 (99 to 192)	11.7 (8.4 to 15.7)	6.9 (4.9 to 9.5)

Cause	Disability adjusted life years: All-age counts (95% UI) 1990	Disability adjusted life years: All-age counts (95% UI) 2017	Disability adjusted life years: Age-standardised rates per 100,000 (95% UI) 1990	Disability adjusted life years: Age-standardised rates per 100,000 (95% UI) 2017
**Transport and unintentional injuries**	**38 447 (36 308 to 40 947)**	**39 513 (36 416 to 42 878)**	**3 171.1 (3 007.9 to 3 354.7)**	**1 962.8 (1 809.3 to 2 131.3)**
**Transport injuries**	**16 036 (15 102 to 17 138)**	**16 550 (14 682 to 18 236)**	**1 302.8 (1 234.7 to 1 379.9)**	**810.5 (720.6 to 893.2)**
Road injuries	15 536 (14 642 to 16 603)	15 738 (13 974 to 17 391)	1 261.6 (1 196.1 to 1 336.6)	770.8 (685.5 to 848.9)
Pedestrian road injuries	7 550 (6 994 to 8 225)	6 805 (6 115 to 7 592)	622.7 (584.2 to 667.0)	335.6 (302.0 to 371.8)
Cyclist road injuries	326 (293 to 365)	560 (491 to 636)	27.7 (25.0 to 31.0)	27.4 (24.0 to 31.0)
Motorcyclist road injuries	2 395 (2 140 to 2 677)	2 338 (2 004 to 2 733)	189.8 (170.6 to 210.1)	113.4 (97.3 to 132.4)
Motor vehicle road injuries	4 857 (4 404 to 5 400)	5 537 (4 682 to 6 290)	387.4 (355.1 to 425.4)	270.0 (228.9 to 306.9)
Other road injuries	409 (349 to 477)	497 (424 to 588)	34.0 (29.3 to 39.2)	24.4 (20.7 to 28.7)
Other transport injuries	500 (448 to 563)	813 (671 to 950)	41.3 (37.2 to 45.9)	39.7 (32.9 to 46.3)
**Unintentional injuries**	**22 411 (20 720 to 24 190)**	**22 962 (21 013 to 25 069)**	**1 868.3 (1 739.5 to 2 008.2)**	**1 152.3 (1 053.7 to 1 260.8)**
Falls	4 860 (4 316 to 5 502)	7 110 (6 004 to 8 419)	514.0 (453.9 to 582.6)	358.2 (302.5 to 423.8)
Drowning	4 711 (4 264 to 5 248)	2 756 (2 498 to 3 050)	344.7 (317.3 to 377.5)	137.2 (124.3 to 152.3)
Fire, heat, and hot substances	1 569 (1 384 to 1 813)	1 034 (880 to 1 226)	128.1 (114.4 to 146.8)	51.8 (44.6 to 61.2)
Poisonings	985 (890 to 1 098)	662 (590 to 735)	73.2 (66.8 to 80.5)	32.9 (29.3 to 36.5)
Poisoning by carbon monoxide	207 (182 to 236)	165 (143 to 190)	15.7 (13.9 to 17.8)	8.2 (7.1 to 9.4)
Poisoning by other means	777 (696 to 872)	497 (442 to 553)	57.5 (52.0 to 63.6)	24.7 (22.0 to 27.6)
Exposure to mechanical forces	3 373 (3 108 to 3 665)	2 355 (2 084 to 2 694)	288.5 (265.8 to 314.2)	115.4 (102.1 to 131.8)
Unintentional firearm injuries	1 628 (1 475 to 1 813)	829 (666 to 953)	133.3 (121.3 to 148.4)	40.4 (32.5 to 46.3)
Other exposure to mechanical forces	1 745 (1 517 to 1 970)	1 526 (1 302 to 1 822)	155.2 (134.7 to 177.4)	75.0 (64.1 to 89.5)
Adverse effects of medical treatment	841 (695 to 961)	877 (772 to 1 178)	70.9 (59.8 to 78.7)	44.8 (39.3 to 60.3)
Animal contact	1 268 (1 070 to 1 518)	525 (473 to 584)	91.0 (79.0 to 105.9)	26.6 (23.8 to 29.7)
Venomous animal contact	1 046 (848 to 1 279)	362 (317 to 410)	72.3 (60.2 to 86.3)	18.5 (16.2 to 21.0)
Non venomous animal contact	222 (160 to 288)	163 (138 to 201)	18.7 (13.6 to 23.5)	8.2 (6.9 to 10.1)
Foreign body	3 351 (2 934 to 3 830)	2 785 (2 456 to 3 169)	236.4 (211.5 to 265.4)	143.2 (125.3 to 164.7)
Pulmonary aspiration and foreign body in airway	3 091 (2 686 to 3 553)	2 604 (2 284 to 2 982)	215.3 (190.8 to 243.2)	134.2 (117.0 to 155.4)
Foreign body in eyes	30 (15 to 51)	61 (34 to 99)	3.1 (1.7 to 5.1)	3.1 (1.7 to 5.0)
Foreign body in other body part	231 (191 to 269)	120 (100 to 168)	18.0 (15.0 to 20.9)	6.0 (5.0 to 8.3)
Environmental heat and cold exposure	350 (314 to 393)	216 (184 to 253)	32.3 (29.1 to 36.3)	10.7 (9.1 to 12.5)
Exposure to forces of nature	108 (87 to 138)	3 769 (3 443 to 4 138)	8.9 (7.1 to 11.4)	188.9 (172.2 to 207.7)
Other unintentional injuries	996 (910 to 1 096)	873 (769 to 996)	80.2 (73.4 to 88.0)	42.6 (37.6 to 48.7)

Death, YLL, incidence, prevalence, YLD, and DALY counts and rates per 100,000 for all injuries in Nayarit in 1990 and 2017

Cause	Deaths				Years of life lost			
	All-age counts (95% UI)		Age-standardised rates per 100,000 (95% UI)		All-age counts (95% UI)		Age-standardised rates per 100,000 (95% UI)	
	1990	2017	1990	2017	1990	2017	1990	2017
**Transport and unintentional injuries**	**626 (589 to 666)**	**498 (464 to 534)**	**83.8 (80.1 to 87.6)**	**40.3 (37.6 to 43.2)**	**35 451 (32 493 to 38 738)**	**21 502 (19 880 to 23 215)**	**3 968.3 (3 698.6 to 4 256.1)**	**1 720.8 (1 591.8 to 1 858.1)**
**Transport injuries**	**284 (267 to 300)**	**268 (246 to 290)**	**37.3 (35.5 to 39.2)**	**21.4 (19.7 to 23.2)**	**16 115 (14 938 to 17 333)**	**12 726 (11 645 to 13 828)**	**1 855.7 (1 740.0 to 1 972.4)**	**1 009.9 (923.9 to 1 097.0)**
Road injuries	271 (255 to 287)	250 (229 to 272)	35.6 (33.9 to 37.3)	20.0 (18.4 to 21.8)	15 401 (14 268 to 16 570)	11 923 (10 903 to 12 950)	1 772.1 (1 659.9 to 1 886.2)	946.2 (865.5 to 1 027.5)
Pedestrian road injuries	123 (113 to 133)	102 (93 to 111)	16.8 (15.8 to 17.9)	8.3 (7.6 to 9.0)	6 757 (6 047 to 7 577)	4 292 (3 887 to 4 728)	765.0 (699.3 to 840.8)	344.1 (312.1 to 378.8)
Cyclist road injuries	8 (7 to 8)	11 (10 to 13)	1.0 (0.9 to 1.1)	0.9 (0.8 to 1.0)	431 (381 to 485)	506 (447 to 570)	50.7 (45.4 to 56.5)	40.3 (35.6 to 45.5)
Motorcyclist road injuries	40 (36 to 44)	34 (30 to 39)	5.0 (4.5 to 5.5)	2.7 (2.3 to 3.0)	2 409 (2 150 to 2 673)	1 889 (1 646 to 2 164)	278.9 (251.9 to 307.9)	148.1 (129.2 to 169.4)
Motor vehicle road injuries	90 (82 to 97)	95 (85 to 105)	11.5 (10.6 to 12.3)	7.5 (6.8 to 8.3)	5 229 (4 711 to 5 683)	4 835 (4 308 to 5 372)	611.5 (555.3 to 661.7)	381.9 (340.8 to 424.3)
Other road injuries	10 (9 to 12)	9 (7 to 10)	1.3 (1.1 to 1.5)	0.7 (0.6 to 0.8)	576 (480 to 676)	401 (339 to 475)	66.1 (55.5 to 76.6)	31.8 (26.9 to 37.6)
Other transport injuries	13 (12 to 14)	17 (15 to 20)	1.7 (1.6 to 1.9)	1.4 (1.2 to 1.6)	713 (637 to 801)	804 (703 to 920)	83.6 (75.3 to 93.3)	63.6 (55.8 to 72.9)
**Unintentional injuries**	**343 (317 to 370)**	**230 (215 to 246)**	**46.5 (44.1 to 49.1)**	**18.8 (17.6 to 20.1)**	**19 336 (17 305 to 21 677)**	**8 776 (8 074 to 9 587)**	**2 112.6 (1 932.5 to 2 315.2)**	**711.0 (653.1 to 776.7)**
Falls	62 (59 to 66)	73 (67 to 79)	11.6 (11.0 to 12.2)	6.0 (5.6 to 6.6)	2 046 (1 896 to 2 206)	1 677 (1 532 to 1 827)	289.5 (272.3 to 305.8)	137.1 (125.2 to 149.3)
Drowning	76 (68 to 85)	38 (35 to 42)	8.9 (8.1 to 9.7)	3.1 (2.8 to 3.3)	4 912 (4 271 to 5 667)	1 982 (1 793 to 2 220)	507.4 (447.9 to 572.3)	159.2 (144.1 to 178.6)
Fire, heat, and hot substances	20 (18 to 22)	12 (11 to 13)	2.8 (2.6 to 3.0)	1.0 (0.9 to 1.1)	1 085 (929 to 1 280)	392 (355 to 431)	117.3 (102.7 to 134.0)	31.9 (28.8 to 35.1)
Poisonings	13 (11 to 14)	7 (6 to 8)	1.5 (1.4 to 1.6)	0.6 (0.5 to 0.6)	808 (696 to 922)	333 (301 to 369)	84.1 (74.2 to 94.0)	26.8 (24.2 to 29.7)
Poisoning by carbon monoxide	3 (3 to 3)	2 (2 to 2)	0.3 (0.3 to 0.4)	0.2 (0.1 to 0.2)	176 (152 to 203)	89 (79 to 102)	18.9 (16.5 to 21.4)	7.1 (6.3 to 8.1)
Poisoning by other means	10 (9 to 11)	5 (5 to 5)	1.2 (1.1 to 1.3)	0.4 (0.4 to 0.4)	632 (543 to 726)	244 (219 to 270)	65.2 (57.3 to 73.4)	19.7 (17.7 to 21.8)
Exposure to mechanical forces	57 (53 to 61)	26 (24 to 29)	7.6 (7.1 to 8.1)	2.1 (1.9 to 2.3)	3 176 (2 916 to 3 484)	1 152 (1 042 to 1 277)	375.0 (345.7 to 402.4)	91.7 (83.0 to 101.6)
Unintentional firearm injuries	32 (29 to 37)	11 (9 to 12)	4.1 (3.8 to 4.8)	0.9 (0.8 to 1.0)	1 829 (1 648 to 2 109)	514 (444 to 588)	214.8 (194.8 to 249.5)	40.6 (35.0 to 46.5)
Other exposure to mechanical forces	25 (20 to 28)	16 (14 to 17)	3.5 (2.8 to 3.8)	1.3 (1.1 to 1.4)	1 347 (1 073 to 1 523)	638 (568 to 717)	160.2 (127.4 to 178.2)	51.1 (45.6 to 57.4)
Adverse effects of medical treatment	15 (12 to 17)	18 (16 to 22)	2.5 (2.1 to 2.7)	1.5 (1.3 to 1.8)	712 (534 to 834)	536 (470 to 714)	82.1 (63.9 to 93.5)	44.1 (38.6 to 53.1)
Animal contact	39 (33 to 47)	15 (13 to 16)	4.4 (3.9 to 5.1)	1.2 (1.1 to 1.3)	2 766 (2 215 to 3 487)	627 (549 to 712)	269.2 (215.6 to 323.5)	51.5 (45.0 to 58.7)
Venomous animal contact	36 (29 to 44)	12 (11 to 14)	4.0 (3.3 to 4.7)	1.0 (0.9 to 1.1)	2 567 (1 978 to 3 294)	528 (448 to 608)	242.0 (189.8 to 300.3)	43.5 (37.0 to 50.2)
Non-venomous animal contact	3 (2 to 7)	2 (2 to 4)	0.5 (0.3 to 0.9)	0.2 (0.2 to 0.3)	198 (110 to 488)	99 (82 to 157)	21.2 (11.9 to 47.0)	8.0 (6.7 to 12.8)
Foreign body	36 (32 to 42)	27 (24 to 30)	4.1 (3.7 to 4.6)	2.2 (2.0 to 2.5)	2 527 (2 123 to 3 000)	1 378 (1 195 to 1 602)	244.7 (210.0 to 284.7)	113.1 (97.6 to 132.0)
Pulmonary aspiration and foreign body in airway	34 (30 to 40)	26 (24 to 29)	3.9 (3.5 to 4.4)	2.1 (1.9 to 2.4)	2 392 (2 005 to 2 851)	1 327 (1 146 to 1 548)	231.1 (197.4 to 269.7)	109.1 (93.8 to 127.6)
Foreign body in eyes								
Foreign body in other body part	2 (1 to 2)	1 (1 to 1)	0.2 (0.1 to 0.3)	0.1 (0.1 to 0.1)	134 (87 to 163)	51 (42 to 68)	13.7 (9.0 to 16.3)	4.1 (3.3 to 5.4)
Environmental heat and cold exposure	4 (4 to 4)	2 (2 to 2)	0.7 (0.6 to 0.7)	0.2 (0.1 to 0.2)	193 (173 to 214)	72 (64 to 80)	24.1 (22.1 to 26.1)	5.8 (5.2 to 6.4)
Exposure to forces of nature	0 (0 to 0)	0 (0 to 0)	0.0 (0.0 to 0.1)	0.0 (0.0 to 0.0)	22 (20 to 25)	10 (9 to 11)	2.4 (2.2 to 2.7)	0.8 (0.7 to 0.9)
Other unintentional injuries	19 (17 to 20)	12 (11 to 14)	2.3 (2.2 to 2.5)	1.0 (0.9 to 1.1)	1 091 (999 to 1 197)	618 (541 to 704)	122.9 (113.0 to 133.8)	48.8 (42.8 to 55.5)

Cause	Incidence				Prevalence			
	All-age counts (95% UI)		Age-standardised rates per 100,000 (95% UI)		All-age counts (95% UI)		Age-standardised rates per 100,000 (95% UI)	
	1990	2017	1990	2017	1990	2017	1990	2017
**Transport and unintentional injuries**	**39 703 (36 734 to 42 960)**	**65 482 (60 206 to 71 113)**	**5 162.3 (4 773.9 to 5 584.8)**	**5 326.8 (4 896.7 to 5 785.8)**	**73 525 (69 166 to 78 872)**	**137 250 (128 822 to 148 368)**	**11 402.4 (10 781.2 to 12 226.5)**	**11 134.0 (10 452.7 to 12 054.0)**
**Transport injuries**	**3 411 (2 933 to 3 957)**	**7 789 (6 775 to 8 919)**	**436.0 (381.0 to 495.1)**	**622.4 (543.0 to 711.5)**	**8 509 (7 918 to 9 101)**	**22 696 (21 078 to 24 439)**	**1 360.7 (1 267.3 to 1 452.5)**	**1 833.9 (1 703.0 to 1 974.2)**
Road injuries	3 222 (2 747 to 3 757)	7 617 (6 600 to 8 746)	412.4 (358.2 to 471.4)	608.5 (528.7 to 697.7)	7 653 (7 111 to 8 222)	21 824 (20 215 to 23 530)	1 231.3 (1 145.4 to 1 320.6)	1 763.3 (1 632.6 to 1 900.7)
Pedestrian road injuries	484 (391 to 599)	736 (597 to 899)	63.0 (52.1 to 75.6)	59.2 (48.1 to 72.0)	1 849 (1 574 to 2 187)	3 523 (3 060 to 4 097)	295.6 (255.1 to 346.2)	286.0 (248.7 to 332.5)
Cyclist road injuries	470 (363 to 604)	897 (715 to 1 116)	55.6 (44.4 to 69.2)	72.4 (57.9 to 90.0)	824 (686 to 970)	2 062 (1 759 to 2 401)	126.2 (106.0 to 146.6)	166.6 (142.2 to 193.5)
Motorcyclist road injuries	387 (311 to 483)	763 (588 to 972)	48.2 (39.0 to 59.0)	60.5 (46.7 to 77.0)	1 457 (1 270 to 1 676)	3 666 (3 123 to 4 271)	233.1 (202.4 to 268.4)	294.5 (251.0 to 342.5)
Motor vehicle road injuries	1 190 (984 to 1 407)	3 660 (3 094 to 4 335)	157.4 (131.2 to 184.1)	290.8 (245.1 to 342.9)	2 228 (1 947 to 2 580)	8 731 (7 620 to 10 186)	369.8 (321.8 to 428.0)	705.4 (614.7 to 822.2)
Other road injuries	690 (547 to 854)	1 561 (1 252 to 1 888)	88.3 (72.1 to 105.2)	125.6 (100.7 to 152.5)	1 296 (1 078 to 1 555)	3 842 (3 192 to 4 647)	206.7 (173.2 to 248.2)	310.8 (258.4 to 375.2)
Other transport injuries	189 (155 to 237)	173 (142 to 210)	23.6 (19.9 to 28.3)	13.9 (11.5 to 17.1)	856 (716 to 1 001)	871 (750 to 999)	129.4 (110.2 to 148.5)	70.6 (60.9 to 80.8)
**Unintentional injuries**	**36 292 (33 424 to 39 499)**	**57 693 (52 544 to 63 231)**	**4 726.3 (4 335.9 to 5 157.3)**	**4 704.4 (4 280.8 to 5 152.1)**	**65 016 (60 781 to 70 166)**	**114 555 (106 303 to 125 441)**	**10 041.7 (9 421.5 to 10 840.0)**	**9 300.1 (8 624.4 to 10 183.3)**
Falls	14 375 (12 658 to 16 284)	31 691 (27 909 to 35 764)	2 018.7 (1 797.3 to 2 264.8)	2 605.2 (2 294.3 to 2 940.1)	25 226 (22 921 to 28 810)	62 838 (56 193 to 71 601)	4 063.8 (3 638.8 to 4 656.1)	5 127.0 (4 583.0 to 5 844.4)
Drowning	56 (48 to 64)	31 (25 to 38)	6.6 (5.8 to 7.5)	2.5 (2.1 to 3.0)	261 (230 to 299)	192 (170 to 221)	41.4 (36.7 to 47.2)	15.6 (13.8 to 18.0)
Fire, heat, and hot substances	1 114 (885 to 1 400)	1 072 (890 to 1 302)	111.8 (91.1 to 136.6)	87.7 (72.5 to 106.9)	10 696 (8 880 to 13 445)	12 760 (10 574 to 16 136)	1 478.1 (1 242.8 to 1 832.1)	1 026.8 (850.7 to 1 298.6)
Poisonings	419 (313 to 545)	464 (359 to 592)	38.8 (29.9 to 49.4)	38.2 (29.4 to 49.0)	362 (278 to 445)	518 (391 to 639)	45.2 (35.1 to 55.3)	41.5 (31.3 to 51.3)
Poisoning by carbon monoxide	72 (48 to 108)	58 (35 to 87)	7.4 (5.2 to 10.4)	4.8 (2.9 to 7.2)	49 (38 to 65)	43 (34 to 55)	6.3 (4.9 to 8.2)	3.5 (2.7 to 4.4)
Poisoning by other means	346 (255 to 458)	406 (309 to 524)	31.5 (23.5 to 40.6)	33.4 (25.3 to 43.4)	313 (231 to 392)	475 (348 to 594)	38.8 (29.0 to 48.7)	38.1 (27.8 to 47.6)
Exposure to mechanical forces	8 449 (7 273 to 9 738)	9 828 (8 489 to 11 357)	1 023.7 (889.0 to 1 171.7)	785.9 (678.4 to 906.9)	17 041 (14 993 to 19 460)	24 726 (21 566 to 28 686)	2 700.4 (2 386.0 to 3 075.5)	1 994.0 (1 739.4 to 2 310.6)
Unintentional firearm injuries	731 (563 to 953)	390 (290 to 510)	88.4 (68.2 to 113.1)	31.0 (23.3 to 40.5)	2 674 (2 151 to 3 240)	2 035 (1 645 to 2 449)	435.2 (352.9 to 526.9)	164.6 (133.2 to 198.1)
Other exposure to mechanical forces	7 718 (6 637 to 8 908)	9 438 (8 160 to 10 947)	935.2 (809.4 to 1 071.7)	754.8 (652.7 to 873.0)	14 367 (12 172 to 16 922)	22 691 (19 434 to 26 801)	2 265.1 (1 938.8 to 2 661.4)	1 829.5 (1 569.2 to 2 158.0)
Adverse effects of medical treatment	710 (605 to 822)	1 563 (1 326 to 1 776)	101.2 (87.5 to 115.8)	127.0 (107.7 to 144.3)	54 (42 to 67)	120 (92 to 147)	7.7 (6.0 to 9.6)	9.7 (7.5 to 12.0)
Animal contact	5 942 (5 353 to 6 798)	5 523 (4 853 to 6 300)	710.2 (632.4 to 792.9)	448.3 (394.8 to 510.0)	6 156 (5 339 to 7 111)	6 846 (6 060 to 7 718)	913.6 (802.9 to 1 032.5)	554.5 (490.9 to 624.4)
Venomous animal contact	2 748 (2 213 to 3 346)	2 511 (2 054 to 3 027)	327.2 (267.7 to 391.7)	203.5 (166.2 to 244.9)	2 010 (1 655 to 2 470)	2 057 (1 724 to 2 422)	286.3 (239.2 to 344.7)	166.1 (139.5 to 195.6)
Non-venomous animal contact	3 193 (2 611 to 3 824)	3 012 (2 516 to 3 588)	383.0 (318.5 to 450.6)	244.9 (204.4 to 291.6)	4 146 (3 354 to 5 021)	4 790 (4 051 to 5 614)	627.4 (521.6 to 741.1)	388.3 (329.2 to 454.7)
Foreign body	3 102 (1 512 to 5 191)	5 486 (2 459 to 8 833)	452.6 (192.3 to 780.7)	441.6 (200.2 to 716.5)	1 304 (888 to 1 757)	1 924 (1 403 to 2 696)	196.1 (149.9 to 263.3)	156.8 (114.8 to 218.5)
Pulmonary aspiration and foreign body in airway	85 (59 to 120)	45 (33 to 63)	8.2 (5.9 to 11.1)	3.7 (2.7 to 5.2)	60 (42 to 82)	40 (33 to 49)	7.6 (5.4 to 10.2)	3.3 (2.7 to 3.9)
Foreign body in eyes	2 805 (1 205 to 4 866)	5 200 (2 232 to 8 678)	421.6 (158.1 to 749.9)	422.2 (180.6 to 697.8)	579 (304 to 994)	1 159 (656 to 1 901)	96.2 (54.0 to 158.6)	95.1 (54.4 to 153.8)
Foreign body in other body part	211 (146 to 306)	191 (135 to 253)	22.8 (16.4 to 31.2)	15.7 (11.0 to 20.5)	665 (533 to 845)	725 (598 to 900)	92.3 (75.2 to 115.7)	58.5 (48.3 to 72.5)
Environmental heat and cold exposure	342 (238 to 458)	233 (178 to 318)	42.6 (32.3 to 53.6)	19.5 (14.5 to 26.1)	1 192 (972 to 1 444)	943 (795 to 1 125)	177.1 (148.0 to 210.3)	76.5 (64.5 to 91.3)
Exposure to forces of nature	58 (47 to 73)	13 (11 to 16)	6.1 (5.2 to 7.4)	1.1 (0.9 to 1.3)	212 (155 to 286)	453 (317 to 629)	27.5 (20.4 to 37.1)	36.0 (25.2 to 49.5)
Other unintentional injuries	1 727 (1 449 to 2 088)	1 833 (1 551 to 2 150)	214.1 (182.5 to 250.2)	147.4 (124.2 to 173.2)	2 518 (2 162 to 2 894)	3 252 (2 827 to 3 712)	390.8 (339.2 to 445.7)	262.4 (228.3 to 299.1)

Cause	Years lived with disability				Disability adjusted life years			
	All-age counts (95% UI)		Age-standardised rates per 100,000 (95% UI)		All-age counts (95% UI)		Age-standardised rates per 100,000 (95% UI)	
	1990	2017	1990	2017	1990	2017	1990	2017
**Transport and unintentional injuries**	**3 167 (2 315 to 4 194)**	**5 899 (4 285 to 7 936)**	**484.3 (353.0 to 640.2)**	**478.6 (347.3 to 643.8)**	**38 618 (35 506 to 42 025)**	**27 402 (25 024 to 29 937)**	**4 452.6 (4 147.9 to 4 768.1)**	**2 199.4 (2 009.5 to 2 403.2)**
**Transport injuries**	**577 (421 to 759)**	**1 381 (1 005 to 1 841)**	**89.7 (65.7 to 118.0)**	**111.3 (80.9 to 148.4)**	**16 691 (15 491 to 17 913)**	**14 108 (12 972 to 15 418)**	**1 945.4 (1 822.2 to 2 063.0)**	**1 121.1 (1 031.4 to 1 225.8)**
Road injuries	523 (382 to 689)	1 335 (969 to 1 784)	81.8 (59.8 to 108.2)	107.5 (78.1 to 143.8)	15 924 (14 757 to 17 089)	13 258 (12 192 to 14 468)	1 853.9 (1 737.9 to 1 966.0)	1 053.7 (970.5 to 1 149.3)
Pedestrian road injuries	132 (93 to 180)	225 (159 to 307)	20.5 (14.5 to 27.7)	18.2 (12.9 to 24.9)	6 889 (6 172 to 7 716)	4 517 (4 109 to 4 960)	785.5 (718.3 to 861.4)	362.3 (329.9 to 397.7)
Cyclist road injuries	57 (40 to 75)	126 (88 to 167)	8.5 (6.1 to 11.2)	10.1 (7.1 to 13.5)	488 (436 to 543)	631 (562 to 708)	59.3 (53.4 to 65.8)	50.5 (44.9 to 56.6)
Motorcyclist road injuries	101 (71 to 136)	227 (156 to 310)	15.8 (11.1 to 21.3)	18.2 (12.5 to 24.8)	2 510 (2 248 to 2 771)	2 116 (1 853 to 2 404)	294.6 (265.3 to 323.9)	166.3 (146.6 to 188.7)
Motor vehicle road injuries	158 (115 to 209)	556 (404 to 739)	25.5 (18.5 to 33.7)	44.8 (32.5 to 59.4)	5 387 (4 859 to 5 858)	5 392 (4 862 to 5 949)	636.9 (580.9 to 689.3)	426.7 (385.4 to 471.1)
Other road injuries	74 (54 to 100)	200 (143 to 276)	11.5 (8.4 to 15.5)	16.2 (11.5 to 22.2)	650 (547 to 756)	602 (514 to 703)	77.6 (65.9 to 89.3)	48.0 (41.0 to 56.0)
Other transport injuries	54 (39 to 72)	46 (33 to 62)	7.8 (5.7 to 10.3)	3.7 (2.7 to 5.0)	767 (687 to 857)	850 (748 to 965)	91.5 (82.9 to 101.3)	67.4 (59.4 to 76.4)
**Unintentional injuries**	**2 590 (1 894 to 3 434)**	**4 518 (3 245 to 6 115)**	**394.6 (286.8 to 526.4)**	**367.3 (263.7 to 496.8)**	**21 927 (19 646 to 24 260)**	**13 294 (11 798 to 14 978)**	**2 507.2 (2 277.3 to 2 742.0)**	**1 078.3 (967.1 to 1 213.5)**
Falls	1 492 (1 103 to 1 945)	3 225 (2 312 to 4 291)	235.0 (172.6 to 305.5)	263.0 (188.5 to 350.8)	3 538 (3 086 to 4 023)	4 902 (3 973 to 5 967)	524.4 (456.6 to 595.5)	400.1 (324.3 to 486.5)
Drowning	15 (10 to 20)	10 (7 to 14)	2.3 (1.6 to 3.1)	0.8 (0.6 to 1.1)	4 926 (4 285 to 5 682)	1 992 (1 804 to 2 229)	509.7 (450.2 to 574.5)	160.1 (144.9 to 179.3)
Fire, heat, and hot substances	230 (143 to 359)	247 (146 to 397)	30.9 (19.2 to 48.3)	19.9 (11.7 to 31.9)	1 315 (1 128 to 1 542)	639 (528 to 794)	148.1 (129.0 to 171.5)	51.8 (42.9 to 64.3)
Poisonings	42 (26 to 63)	58 (35 to 86)	5.1 (3.2 to 7.7)	4.7 (2.8 to 6.9)	850 (742 to 968)	391 (349 to 432)	89.2 (79.3 to 99.3)	31.4 (28.1 to 34.8)
Poisoning by carbon monoxide	5 (3 to 8)	4 (3 to 7)	0.6 (0.4 to 1.0)	0.3 (0.2 to 0.5)	181 (157 to 209)	93 (83 to 106)	19.5 (17.1 to 22.1)	7.5 (6.6 to 8.5)
Poisoning by other means	37 (23 to 56)	54 (32 to 82)	4.5 (2.8 to 6.8)	4.3 (2.6 to 6.5)	669 (578 to 763)	298 (263 to 333)	69.7 (61.2 to 78.1)	24.0 (21.1 to 26.8)
Exposure to mechanical forces	400 (277 to 572)	536 (362 to 785)	62.3 (43.1 to 89.7)	43.2 (29.2 to 63.2)	3 576 (3 269 to 3 898)	1 688 (1 475 to 1 949)	437.3 (402.3 to 476.6)	134.9 (118.0 to 155.7)
Unintentional firearm injuries	56 (37 to 85)	37 (24 to 59)	8.9 (5.8 to 13.6)	3.0 (1.9 to 4.8)	1 885 (1 701 to 2 166)	551 (483 to 624)	223.7 (203.0 to 259.0)	43.6 (38.0 to 49.4)
Other exposure to mechanical forces	344 (237 to 489)	499 (334 to 732)	53.4 (36.9 to 75.9)	40.2 (26.9 to 58.9)	1 691 (1 412 to 1 919)	1 137 (960 to 1 371)	213.6 (179.6 to 243.5)	91.3 (77.0 to 110.2)
Adverse effects of medical treatment	7 (5 to 11)	16 (10 to 24)	1.0 (0.6 to 1.6)	1.3 (0.8 to 2.0)	719 (540 to 842)	552 (484 to 731)	83.2 (65.0 to 94.5)	45.4 (39.8 to 60.3)
Animal contact	168 (113 to 237)	153 (101 to 221)	22.8 (15.4 to 32.0)	12.3 (8.1 to 17.7)	2 934 (2 371 to 3 603)	780 (687 to 893)	286.0 (236.9 to 345.5)	63.9 (56.2 to 73.2)
Venomous animal contact	105 (68 to 152)	86 (57 to 123)	13.4 (8.7 to 19.0)	6.9 (4.6 to 9.9)	2 673 (2 078 to 3 343)	614 (529 to 700)	255.4 (202.4 to 314.1)	50.4 (43.4 to 57.6)
Non-venomous animal contact	63 (37 to 102)	67 (39 to 110)	9.4 (5.7 to 15.2)	5.4 (3.2 to 8.9)	261 (159 to 560)	166 (130 to 241)	30.5 (19.0 to 58.5)	13.5 (10.5 to 19.6)
Foreign body	82 (53 to 121)	113 (69 to 180)	12.1 (7.9 to 18.3)	9.2 (5.6 to 14.7)	2 608 (2 207 to 3 080)	1 491 (1 300 to 1 712)	256.8 (222.1 to 297.3)	122.3 (106.4 to 140.3)
Pulmonary aspiration and foreign body in airway	3 (2 to 4)	2 (1 to 2)	0.4 (0.3 to 0.5)	0.1 (0.1 to 0.2)	2 395 (2 008 to 2 856)	1 329 (1 148 to 1 550)	231.4 (197.9 to 270.2)	109.2 (93.9 to 127.7)
Foreign body in eyes	39 (19 to 71)	77 (41 to 135)	6.4 (3.4 to 11.5)	6.3 (3.4 to 11.2)	39 (19 to 71)	77 (41 to 135)	6.4 (3.4 to 11.5)	6.3 (3.4 to 11.2)
Foreign body in other body part	40 (28 to 53)	34 (24 to 46)	5.3 (3.7 to 6.9)	2.8 (2.0 to 3.7)	174 (126 to 205)	85 (71 to 107)	19.0 (14.1 to 21.9)	6.8 (5.6 to 8.6)
Environmental heat and cold exposure	47 (32 to 67)	36 (24 to 51)	6.9 (4.7 to 9.8)	2.9 (1.9 to 4.2)	240 (218 to 268)	108 (92 to 125)	31.0 (27.9 to 34.4)	8.7 (7.4 to 10.1)
Exposure to forces of nature	10 (7 to 14)	15 (11 to 20)	1.3 (1.0 to 1.8)	1.2 (0.9 to 1.6)	33 (29 to 37)	25 (21 to 30)	3.8 (3.3 to 4.3)	2.0 (1.7 to 2.4)
Other unintentional injuries	97 (70 to 131)	110 (78 to 150)	14.9 (10.7 to 20.1)	8.8 (6.3 to 12.1)	1 188 (1 087 to 1 300)	727 (647 to 822)	137.7 (127.0 to 149.8)	57.6 (51.8 to 65.1)

Death, YLL, incidence, prevalence, YLD, and DALY counts and rates per 100,000 for all injuries in Nuevo León in 1990 and 2017

Cause	Deaths				Years of life lost			
	All-age counts (95% UI)		Age-standardised rates per 100,000 (95% UI)		All-age counts (95% UI)		Age-standardised rates per 100,000 (95% UI)	
	1990	2017	1990	2017	1990	2017	1990	2017
**Transport and unintentional injuries**	**1 416 (1 356 to 1 481)**	**1 494 (1 399 to 1 592)**	**54.7 (53.0 to 56.5)**	**29.7 (27.9 to 31.6)**	**74 038 (69 392 to 79 264)**	**62 460 (57 785 to 66 965)**	**2 287.8 (2 165.6 to 2 420.5)**	**1 171.2 (1 086.6 to 1 256.1)**
**Transport injuries**	**643 (608 to 676)**	**717 (657 to 776)**	**22.1 (21.1 to 23.1)**	**13.4 (12.3 to 14.4)**	**35 522 (33 228 to 37 692)**	**33 161 (30 312 to 35 854)**	**1 059.3 (998.1 to 1 119.7)**	**602.1 (550.0 to 650.7)**
Road injuries	621 (587 to 654)	682 (623 to 736)	21.3 (20.3 to 22.3)	12.7 (11.7 to 13.7)	34 383 (32 152 to 36 512)	31 551 (28 834 to 34 133)	1 024.5 (966.2 to 1 082.7)	573.0 (524.5 to 619.4)
Pedestrian road injuries	265 (246 to 285)	327 (299 to 358)	10.1 (9.5 to 10.7)	6.3 (5.8 to 6.8)	13 998 (12 741 to 15 459)	13 728 (12 479 to 15 025)	437.5 (403.4 to 477.3)	252.0 (228.9 to 275.5)
Cyclist road injuries	13 (12 to 14)	23 (21 to 26)	0.5 (0.4 to 0.5)	0.4 (0.4 to 0.5)	700 (623 to 783)	986 (867 to 1 098)	21.5 (19.3 to 23.9)	18.1 (16.0 to 20.1)
Motorcyclist road injuries	111 (100 to 123)	85 (75 to 96)	3.4 (3.1 to 3.7)	1.5 (1.3 to 1.7)	6 502 (5 772 to 7 282)	4 615 (4 027 to 5 241)	184.7 (165.8 to 203.6)	82.4 (73.9 to 93.8)
Motor vehicle road injuries	219 (203 to 237)	231 (208 to 258)	6.9 (6.4 to 7.4)	4.2 (3.8 to 4.7)	12 503 (11 368 to 13 621)	11 524 (10 328 to 12 913)	360.3 (329.3 to 390.0)	207.8 (186.4 to 232.7)
Other road injuries	13 (11 to 14)	15 (13 to 17)	0.5 (0.4 to 0.5)	0.3 (0.3 to 0.3)	680 (576 to 784)	698 (605 to 802)	20.5 (17.7 to 23.4)	12.7 (11.1 to 14.6)
Other transport injuries	22 (20 to 23)	36 (31 to 40)	0.8 (0.7 to 0.9)	0.7 (0.6 to 0.7)	1 139 (1 046 to 1 241)	1 611 (1 396 to 1 834)	34.8 (32.1 to 37.9)	29.1 (25.3 to 33.0)
**Unintentional injuries**	**773 (736 to 816)**	**777 (726 to 828)**	**32.6 (31.5 to 33.9)**	**16.3 (15.2 to 17.4)**	**38 517 (35 546 to 42 038)**	**29 298 (26 915 to 31 956)**	**1 228.4 (1 152.8 to 1 317.2)**	**569.0 (521.7 to 622.5)**
Falls	181 (172 to 189)	267 (244 to 289)	10.4 (9.9 to 10.9)	6.0 (5.5 to 6.5)	5 639 (5 312 to 5 976)	6 222 (5 669 to 6 770)	237.4 (225.5 to 249.0)	124.0 (113.5 to 134.6)
Drowning	170 (158 to 185)	99 (91 to 108)	5.6 (5.2 to 6.0)	1.9 (1.7 to 2.1)	10 184 (9 260 to 11 305)	4 849 (4 407 to 5 356)	290.6 (266.6 to 319.4)	91.1 (82.6 to 101.0)
Fire, heat, and hot substances	65 (60 to 72)	54 (49 to 59)	3.0 (2.8 to 3.2)	1.2 (1.1 to 1.3)	3 127 (2 725 to 3 663)	1 716 (1 547 to 1 876)	102.3 (91.8 to 116.4)	33.5 (30.3 to 36.5)
Poisonings	33 (30 to 36)	26 (23 to 28)	1.1 (1.1 to 1.2)	0.5 (0.5 to 0.5)	1 926 (1 704 to 2 195)	1 170 (1 047 to 1 290)	56.8 (50.8 to 64.0)	22.0 (19.7 to 24.3)
Poisoning by carbon monoxide	15 (13 to 16)	13 (11 to 14)	0.5 (0.5 to 0.6)	0.2 (0.2 to 0.3)	847 (742 to 969)	576 (512 to 646)	24.9 (22.3 to 28.2)	10.8 (9.6 to 12.1)
Poisoning by other means	18 (16 to 20)	13 (12 to 14)	0.6 (0.6 to 0.7)	0.2 (0.2 to 0.3)	1 079 (939 to 1 250)	594 (528 to 665)	31.9 (28.2 to 36.2)	11.3 (10.0 to 12.6)
Exposure to mechanical forces	96 (90 to 103)	63 (58 to 69)	3.5 (3.3 to 3.7)	1.2 (1.1 to 1.3)	5 078 (4 692 to 5 464)	2 656 (2 409 to 2 906)	157.3 (146.1 to 167.4)	48.7 (44.2 to 53.2)
Unintentional firearm injuries	38 (34 to 47)	18 (15 to 20)	1.3 (1.1 to 1.6)	0.3 (0.3 to 0.4)	2 086 (1 856 to 2 612)	813 (687 to 938)	61.5 (55.2 to 77.1)	14.7 (12.5 to 16.9)
Other exposure to mechanical forces	59 (47 to 64)	46 (41 to 50)	2.3 (1.9 to 2.4)	0.9 (0.8 to 1.0)	2 992 (2 336 to 3 319)	1 843 (1 644 to 2 032)	95.8 (75.7 to 104.8)	34.0 (30.3 to 37.4)
Adverse effects of medical treatment	41 (38 to 48)	59 (53 to 79)	2.1 (2.0 to 2.4)	1.3 (1.2 to 1.7)	1 629 (1 428 to 2 083)	1 708 (1 483 to 2 496)	58.9 (53.2 to 72.5)	34.2 (29.7 to 49.7)
Animal contact	8 (6 to 9)	5 (4 to 5)	0.3 (0.3 to 0.3)	0.1 (0.1 to 0.1)	458 (373 to 568)	191 (170 to 216)	13.6 (11.3 to 16.5)	3.8 (3.3 to 4.2)
Venomous animal contact	4 (3 to 5)	2 (1 to 2)	0.1 (0.1 to 0.2)	0.0 (0.0 to 0.0)	285 (215 to 374)	84 (58 to 104)	8.1 (6.3 to 10.6)	1.7 (1.2 to 2.1)
Non venomous animal contact	3 (3 to 4)	3 (3 to 4)	0.1 (0.1 to 0.2)	0.1 (0.1 to 0.1)	173 (140 to 208)	107 (92 to 136)	5.5 (4.6 to 6.5)	2.1 (1.8 to 2.6)
Foreign body	129 (116 to 144)	164 (146 to 184)	4.6 (4.3 to 5.0)	3.3 (3.0 to 3.7)	7 877 (6 805 to 9 137)	9 021 (7 633 to 10 584)	229.2 (201.7 to 261.1)	179.7 (150.9 to 212.2)
Pulmonary aspiration and foreign body in airway	121 (109 to 136)	160 (142 to 179)	4.4 (4.1 to 4.8)	3.3 (2.9 to 3.7)	7 428 (6 393 to 8 632)	8 799 (7 404 to 10 334)	216.3 (189.6 to 246.9)	175.7 (147.0 to 207.6)
Foreign body in eyes								
Foreign body in other body part	7 (5 to 8)	4 (3 to 5)	0.2 (0.2 to 0.3)	0.1 (0.1 to 0.1)	449 (312 to 538)	222 (183 to 303)	12.9 (8.9 to 15.1)	4.1 (3.4 to 5.6)
Environmental heat and cold exposure	16 (15 to 18)	11 (9 to 12)	0.8 (0.8 to 0.9)	0.2 (0.2 to 0.2)	665 (607 to 734)	363 (316 to 405)	25.1 (23.3 to 27.3)	6.7 (5.9 to 7.5)
Exposure to forces of nature	2 (1 to 2)	1 (1 to 1)	0.1 (0.0 to 0.1)	0.0 (0.0 to 0.0)	95 (86 to 106)	43 (40 to 48)	2.7 (2.5 to 3.0)	0.8 (0.7 to 0.9)
Other unintentional injuries	33 (30 to 35)	28 (25 to 31)	1.1 (1.0 to 1.2)	0.5 (0.5 to 0.6)	1 844 (1 678 to 2 029)	1 358 (1 199 to 1 523)	54.5 (50.1 to 59.3)	24.4 (21.6 to 27.3)

Cause	Incidence				Prevalence			
	All-age counts (95% UI)		Age-standardised rates per 100,000 (95% UI)		All-age counts (95% UI)		Age-standardised rates per 100,000 (95% UI)	
	1990	2017	1990	2017	1990	2017	1990	2017
**Transport and unintentional injuries**	**103 324 (94 623 to 112 320)**	**170 192 (153 036 to 189 244)**	**3 670.8 (3 383.5 to 3 965.8)**	**3 333.3 (2 997.1 to 3 703.4)**	**211 775 (198 877 to 226 628)**	**364 271 (339 396 to 395 801)**	**8 671.7 (8 137.3 to 9 310.3)**	**7 028.2 (6 547.0 to 7 641.0)**
**Transport injuries**	**9 709 (8 173 to 11 517)**	**21 059 (17 991 to 25 252)**	**323.2 (275.8 to 373.3)**	**388.0 (321.9 to 464.6)**	**23 799 (22 200 to 25 384)**	**61 078 (57 002 to 65 643)**	**991.0 (927.6 to 1 060.2)**	**1 146.2 (1 070.9 to 1 230.4)**
Road injuries	9 124 (7 626 to 10 913)	20 218 (16 650 to 24 274)	304.3 (256.3 to 354.5)	371.7 (305.4 to 446.8)	20 985 (19 517 to 22 464)	56 474 (52 423 to 60 965)	887.1 (826.5 to 949.5)	1 058.9 (983.5 to 1 142.9)
Pedestrian road injuries	1 417 (1 106 to 1 771)	2 685 (2 046 to 3 535)	50.3 (40.5 to 61.6)	49.8 (38.5 to 64.7)	5 127 (4 401 to 6 014)	11 860 (10 193 to 13 899)	221.6 (193.1 to 259.4)	224.6 (194.9 to 263.4)
Cyclist road injuries	1 249 (896 to 1 702)	2 327 (1 638 to 3 275)	40.1 (29.7 to 52.9)	43.9 (30.9 to 60.9)	2 100 (1 752 to 2 473)	4 853 (4 137 to 5 580)	84.8 (71.7 to 97.8)	92.0 (78.8 to 105.6)
Motorcyclist road injuries	1 436 (1 099 to 1 840)	2 502 (1 786 to 3 323)	45.1 (35.2 to 56.2)	46.1 (33.2 to 61.1)	4 922 (4 247 to 5 714)	11 291 (9 657 to 13 284)	201.8 (175.1 to 232.8)	208.3 (178.4 to 243.5)
Motor vehicle road injuries	3 251 (2 536 to 4 044)	9 392 (7 314 to 11 683)	109.0 (86.9 to 132.7)	169.8 (132.8 to 211.9)	5 524 (4 828 to 6 477)	20 782 (17 974 to 24 698)	241.6 (210.2 to 282.7)	387.0 (335.6 to 457.3)
Other road injuries	1 771 (1 280 to 2 347)	3 302 (2 295 to 4 605)	59.8 (44.6 to 75.7)	62.1 (43.6 to 86.1)	3 314 (2 742 to 4 008)	7 748 (6 448 to 9 413)	137.2 (113.7 to 164.4)	147.0 (122.4 to 177.8)
Other transport injuries	585 (411 to 856)	841 (599 to 1 188)	18.9 (14.0 to 25.7)	16.3 (11.4 to 23.0)	2 813 (2 292 to 3 396)	4 604 (3 863 to 5 336)	106.0 (88.2 to 124.9)	87.3 (73.6 to 100.9)
**Unintentional injuries**	**93 615 (85 196 to 102 234)**	**149 133 (132 298 to 168 258)**	**3 347.6 (3 061.0 to 3 646.1)**	**2 945.3 (2 617.1 to 3 310.8)**	**187 976 (174 778 to 202 674)**	**303 192 (279 338 to 333 945)**	**7 678.6 (7 138.4 to 8 309.7)**	**5 882.0 (5 423.6 to 6 486.8)**
Falls	40 225 (34 835 to 46 372)	85 619 (72 764 to 101 101)	1 624.7 (1 427.2 to 1 847.6)	1 740.3 (1 477.3 to 2 054.5)	72 183 (63 375 to 82 787)	170 018 (150 534 to 195 173)	3 230.1 (2 857.4 to 3 690.6)	3 382.8 (3 000.3 to 3 876.1)
Drowning	150 (128 to 175)	124 (97 to 160)	4.9 (4.2 to 5.6)	2.4 (1.9 to 3.0)	704 (618 to 808)	784 (685 to 930)	29.9 (26.4 to 34.0)	15.1 (13.2 to 17.8)
Fire, heat, and hot substances	4 012 (3 116 to 5 190)	2 951 (2 087 to 4 042)	115.0 (91.4 to 145.4)	58.4 (41.0 to 80.6)	40 592 (33 114 to 51 391)	36 108 (28 873 to 47 508)	1 470.1 (1 219.7 to 1 808.9)	675.3 (542.2 to 884.1)
Poisonings	1 326 (952 to 1 875)	1 530 (995 to 2 230)	35.5 (26.0 to 49.2)	31.0 (20.1 to 45.1)	1 277 (969 to 1 601)	1 935 (1 404 to 2 443)	41.1 (31.4 to 51.2)	35.9 (26.0 to 45.3)
Poisoning by carbon monoxide	283 (171 to 446)	336 (169 to 587)	8.1 (5.2 to 12.2)	6.6 (3.3 to 11.8)	211 (161 to 276)	288 (220 to 371)	7.0 (5.4 to 9.0)	5.3 (4.1 to 6.9)
Poisoning by other means	1 043 (729 to 1 512)	1 194 (772 to 1 824)	27.4 (19.4 to 39.4)	24.4 (15.5 to 37.5)	1 066 (758 to 1 379)	1 647 (1 130 to 2 160)	34.1 (24.6 to 43.9)	30.5 (21.0 to 40.0)
Exposure to mechanical forces	24 012 (20 110 to 28 857)	25 525 (19 986 to 32 055)	747.8 (636.2 to 885.3)	474.2 (371.5 to 595.7)	47 146 (41 023 to 54 580)	64 768 (56 050 to 75 822)	1 916.8 (1 676.6 to 2 201.7)	1 208.7 (1 046.1 to 1 408.2)
Unintentional firearm injuries	1 585 (1 103 to 2 193)	1 114 (625 to 1 878)	47.5 (34.4 to 63.4)	20.7 (11.7 to 34.5)	5 936 (4 689 to 7 084)	5 641 (4 537 to 6 657)	243.0 (196.1 to 289.1)	106.2 (86.2 to 125.1)
Other exposure to mechanical forces	22 427 (18 719 to 27 124)	24 411 (19 177 to 30 746)	700.3 (596.9 to 832.4)	453.5 (358.5 to 568.9)	41 210 (34 704 to 48 624)	59 127 (50 000 to 70 320)	1 673.8 (1 428.5 to 1 963.6)	1 102.5 (932.9 to 1 307.3)
Adverse effects of medical treatment	2 976 (1 965 to 2 805)	5 336 (4 448 to 6 348)	92.4 (78.0 to 107.7)	100.7 (84.3 to 118.2)	182 (138 to 229)	408 (308 to 517)	7.1 (5.4 to 8.8)	7.7 (5.8 to 9.7)
Animal contact	6 378 (4 826 to 8 385)	5 482 (4 425 to 6 645)	204.0 (160.8 to 257.1)	105.9 (86.0 to 128.2)	7 027 (6 004 to 8 225)	6 832 (5 996 to 7 794)	274.3 (237.5 to 316.7)	130.7 (114.8 to 148.8)
Venomous animal contact	2 173 (1 482 to 3 124)	2 009 (1 489 to 2 618)	68.8 (48.7 to 94.2)	38.5 (28.6 to 50.1)	1 456 (1 197 to 1 722)	1 513 (1 274 to 1 761)	53.2 (44.5 to 62.0)	28.5 (24.1 to 33.1)
Non venomous animal contact	4 205 (3 065 to 5 587)	3 473 (2 725 to 4 369)	135.3 (102.5 to 174.1)	67.5 (53.2 to 85.2)	5 571 (4 596 to 6 732)	5 319 (4 511 to 6 287)	221.1 (185.1 to 262.3)	102.2 (86.7 to 120.1)
Foreign body	8 224 (5 061 to 12 102)	15 774 (7 469 to 26 571)	295.6 (168.1 to 444.8)	301.0 (145.2 to 491.5)	4 933 (3 952 to 6 227)	6 150 (4 641 to 8 325)	185.9 (151.8 to 230.5)	119.3 (90.8 to 160.1)
Pulmonary aspiration and foreign body in airway	420 (307 to 559)	333 (241 to 472)	11.9 (9.0 to 15.6)	6.8 (4.8 to 9.6)	326 (230 to 443)	323 (258 to 403)	10.9 (7.9 to 14.5)	6.1 (4.9 to 7.6)
Foreign body in eyes	6 827 (3 758 to 10 684)	14 728 (6 449 to 25 389)	254.1 (125.6 to 404.2)	280.1 (123.8 to 470.8)	1 335 (689 to 2 244)	3 159 (1 771 to 5 225)	57.2 (33.2 to 89.8)	62.5 (35.4 to 99.5)
Foreign body in other body part	978 (674 to 1 373)	708 (500 to 957)	29.6 (20.8 to 42.2)	14.0 (9.8 to 18.8)	3 273 (2 599 to 4 202)	2 668 (2 204 to 3 297)	117.8 (95.3 to 147.2)	50.7 (42.2 to 62.7)
Environmental heat and cold exposure	1 537 (1 067 to 2 058)	1 104 (778 to 1 608)	52.6 (39.8 to 66.1)	22.1 (15.6 to 31.8)	5 617 (4 533 to 6 869)	4 540 (3 800 to 5 426)	217.5 (180.8 to 260.5)	87.4 (73.4 to 104.1)
Exposure to forces of nature	249 (204 to 311)	58 (49 to 69)	7.0 (5.9 to 8.5)	1.1 (0.9 to 1.3)	792 (526 to 1 001)	1 873 (1 128 to 2 638)	23.5 (17.2 to 32.1)	33.6 (23.0 to 47.2)
Other unintentional injuries	5 126 (4 303 to 6 369)	5 629 (4 515 to 7 139)	168.1 (137.3 to 201.4)	103.4 (87.0 to 135.8)	7 582 (6 447 to 8 883)	9 779 (8 467 to 11 190)	302.5 (261.6 to 346.8)	185.7 (161.7 to 210.8)

Cause	Years lived with disability				Disability adjusted life years			
	All-age counts (95% UI)		Age-standardised rates per 100,000 (95% UI)		All-age counts (95% UI)		Age-standardised rates per 100,000 (95% UI)	
	1990	2017	1990	2017	1990	2017	1990	2017
**Transport and unintentional injuries**	**8 716 (6 231 to 11 744)**	**15 391 (10 938 to 20 964)**	**354.8 (255.0 to 474.7)**	**297.4 (211.5 to 403.4)**	**82 754 (77 411 to 88 637)**	**77 850 (71 367 to 84 540)**	**2 642.6 (2 481.2 to 2 828.2)**	**1 468.6 (1 345.1 to 1 597.7)**
**Transport injuries**	**1 561 (1 127 to 2 084)**	**3 620 (2 623 to 4 882)**	**62.7 (45.1 to 83.7)**	**67.2 (48.5 to 90.4)**	**37 082 (34 674 to 39 336)**	**36 781 (33 769 to 39 586)**	**1 122.0 (1 055.4 to 1 181.7)**	**669.3 (614.2 to 720.6)**
Road injuries	1 390 (1 006 to 1 855)	3 382 (2 449 to 4 553)	56.6 (41.0 to 75.4)	62.7 (45.4 to 84.2)	35 772 (33 451 to 37 961)	34 933 (32 088 to 37 687)	1 081.1 (1 016.7 to 1 138.9)	635.7 (583.6 to 684.9)
Pedestrian road injuries	356 (250 to 484)	735 (511 to 1 019)	14.7 (10.4 to 20.1)	13.8 (9.6 to 19.2)	14 354 (13 074 to 15 777)	14 463 (13 201 to 15 709)	452.2 (417.6 to 490.2)	265.8 (242.9 to 288.7)
Cyclist road injuries	140 (99 to 185)	285 (199 to 382)	5.5 (3.9 to 7.2)	5.3 (3.7 to 7.2)	840 (751 to 930)	1 270 (1 129 to 1 425)	27.0 (24.3 to 29.9)	23.4 (20.8 to 26.2)
Motorcyclist road injuries	331 (231 to 448)	684 (460 to 939)	13.1 (9.1 to 17.8)	12.5 (8.4 to 17.2)	6 832 (6 075 to 7 613)	5 299 (4 655 to 5 942)	197.8 (177.6 to 217.3)	94.9 (83.5 to 106.4)
Motor vehicle road injuries	379 (270 to 509)	1 289 (920 to 1 736)	15.9 (11.4 to 21.0)	23.7 (16.9 to 31.9)	12 881 (11 736 to 14 024)	12 813 (11 568 to 14 151)	376.2 (344.9 to 405.7)	231.5 (208.1 to 255.3)
Other road injuries	185 (131 to 252)	390 (277 to 537)	7.4 (5.2 to 10.0)	7.3 (5.2 to 10.1)	865 (748 to 1 005)	1 088 (941 to 1 258)	27.8 (24.3 to 31.9)	20.1 (17.4 to 23.3)
Other transport injuries	171 (119 to 230)	238 (168 to 323)	6.2 (4.3 to 8.3)	4.5 (3.2 to 6.1)	1 310 (1 198 to 1 433)	1 849 (1 629 to 2 073)	41.0 (37.6 to 44.5)	33.6 (29.7 to 37.7)
**Unintentional injuries**	**7 155 (5 118 to 9 652)**	**11 771 (8 295 to 16 072)**	**292.1 (209.3 to 394.1)**	**230.2 (162.4 to 314.7)**	**45 672 (41 839 to 49 963)**	**41 069 (36 944 to 45 503)**	**1 520.6 (1 405.2 to 1 655.3)**	**799.3 (718.6 to 885.5)**
Falls	4 029 (2 951 to 5 285)	8 354 (5 948 to 11 242)	174.8 (127.1 to 229.1)	166.1 (117.9 to 222.8)	9 668 (8 515 to 10 992)	14 576 (12 064 to 17 455)	412.2 (363.1 to 468.7)	290.1 (240.7 to 347.3)
Drowning	39 (27 to 55)	41 (28 to 57)	1.6 (1.1 to 2.2)	0.8 (0.5 to 1.1)	10 223 (9 304 to 11 346)	4 890 (4 447 to 5 397)	292.2 (268.2 to 320.9)	91.9 (83.4 to 101.8)
Fire, heat, and hot substances	836 (506 to 1 338)	684 (392 to 1 128)	29.5 (17.9 to 46.7)	12.8 (7.3 to 21.0)	3 963 (3 428 to 4 715)	2 400 (2 060 to 2 855)	131.8 (115.4 to 154.3)	46.3 (38.8 to 54.7)
Poisonings	151 (94 to 226)	225 (132 to 347)	4.7 (2.9 to 7.0)	4.2 (2.5 to 6.5)	2 077 (1 838 to 2 362)	1 396 (1 238 to 1 557)	61.5 (55.1 to 69.0)	26.2 (23.2 to 29.2)
Poisoning by carbon monoxide	23 (14 to 37)	29 (18 to 46)	0.7 (0.4 to 1.1)	0.5 (0.3 to 0.9)	870 (765 to 993)	605 (542 to 674)	25.6 (23.0 to 28.9)	11.3 (10.1 to 12.6)
Poisoning by other means	128 (76 to 198)	197 (112 to 309)	4.0 (2.4 to 6.1)	3.6 (2.1 to 5.7)	1 207 (1 056 to 1 394)	791 (678 to 920)	35.9 (31.8 to 40.9)	14.9 (12.8 to 17.3)
Exposure to mechanical forces	1 082 (738 to 1 556)	1 369 (909 to 2 024)	41.2 (29.5 to 62.2)	25.5 (17.0 to 37.5)	6 159 (5 624 to 6 788)	4 025 (3 519 to 4 725)	200.5 (181.9 to 222.6)	74.2 (64.9 to 87.1)
Unintentional firearm injuries	120 (78 to 184)	105 (67 to 164)	4.7 (3.1 to 7.3)	2.0 (1.3 to 3.1)	2 206 (1 963 to 2 734)	918 (781 to 1 043)	66.3 (59.4 to 81.8)	16.7 (14.2 to 18.9)
Other exposure to mechanical forces	961 (656 to 1 384)	1 265 (838 to 1 865)	36.4 (26.1 to 55.2)	23.6 (15.7 to 34.7)	3 953 (3 262 to 4 480)	3 107 (2 645 to 3 760)	134.2 (112.1 to 153.9)	57.5 (48.9 to 69.4)
Adverse effects of medical treatment	24 (15 to 37)	54 (34 to 83)	0.9 (0.6 to 1.4)	1.0 (0.6 to 1.6)	1 647 (1 451 to 2 101)	1 763 (1 537 to 2 543)	59.8 (54.2 to 73.7)	35.3 (30.8 to 50.8)
Animal contact	180 (139 to 257)	152 (99 to 220)	6.4 (4.2 to 9.1)	2.9 (1.9 to 4.2)	638 (531 to 766)	343 (288 to 416)	20.0 (16.8 to 23.8)	6.6 (5.6 to 8.0)
Venomous animal contact	98 (62 to 144)	79 (49 to 116)	3.2 (2.0 to 4.6)	1.5 (0.9 to 2.1)	382 (304 to 478)	163 (126 to 205)	11.3 (9.1 to 13.9)	3.1 (2.4 to 3.9)
Non venomous animal contact	83 (50 to 133)	73 (43 to 120)	3.2 (2.0 to 5.2)	1.4 (0.8 to 2.3)	255 (206 to 316)	181 (144 to 230)	8.7 (7.3 to 10.8)	3.5 (2.8 to 4.4)
Foreign body	287 (194 to 404)	343 (213 to 535)	10.6 (7.2 to 14.9)	6.6 (4.1 to 10.3)	8 164 (7 068 to 9 419)	9 364 (7 989 to 10 892)	239.8 (211.2 to 272.4)	186.4 (157.5 to 218.4)
Pulmonary aspiration and foreign body in airway	16 (11 to 22)	13 (9 to 18)	0.5 (0.3 to 0.7)	0.2 (0.2 to 0.3)	7 444 (6 415 to 8 644)	8 812 (7 416 to 10 344)	216.8 (190.1 to 247.4)	175.9 (147.2 to 207.9)
Foreign body in eyes	89 (45 to 158)	212 (112 to 376)	3.8 (2.1 to 6.5)	4.2 (2.2 to 7.3)	89 (45 to 158)	212 (112 to 376)	3.8 (2.1 to 6.5)	4.2 (2.2 to 7.3)
Foreign body in other body part	182 (127 to 246)	118 (83 to 159)	6.2 (4.4 to 8.3)	2.2 (1.6 to 3.0)	631 (489 to 738)	340 (285 to 433)	19.1 (15.0 to 22.3)	6.3 (5.3 to 8.1)
Environmental heat and cold exposure	213 (139 to 309)	169 (110 to 245)	8.3 (5.4 to 11.8)	3.3 (2.1 to 4.7)	878 (780 to 992)	532 (457 to 625)	33.4 (29.9 to 37.5)	10.0 (8.6 to 11.8)
Exposure to forces of nature	33 (24 to 44)	57 (41 to 81)	1.1 (0.8 to 1.4)	1.0 (0.7 to 1.4)	128 (114 to 148)	100 (88 to 124)	3.8 (3.4 to 4.2)	1.8 (1.5 to 2.3)
Other unintentional injuries	281 (201 to 380)	321 (226 to 445)	11.0 (7.9 to 14.9)	6.1 (4.3 to 8.5)	2 125 (1 939 to 2 346)	1 680 (1 495 to 1 873)	65.5 (60.0 to 72.2)	30.5 (27.2 to 33.9)

Death, YLL, incidence, prevalence, YLD, and DALY counts and rates per 100,000 for all injuries in Oaxaca in 1990 and 2017

Cause	Deaths: All-age counts (95% UI) 1990	Deaths: All-age counts (95% UI) 2017	Deaths: Age-standardised rates per 100,000 (95% UI) 1990	Deaths: Age-standardised rates per 100,000 (95% UI) 2017	Years of life lost: All-age counts (95% UI) 1990	Years of life lost: All-age counts (95% UI) 2017	Years of life lost: Age-standardised rates per 100,000 (95% UI) 1990	Years of life lost: Age-standardised rates per 100,000 (95% UI) 2017
**Transport and unintentional injuries**	**1 616 (1 542 to 1 706)**	**1 392 (1 297 to 1 493)**	**62.7 (60.5 to 65.1)**	**32.9 (30.6 to 35.3)**	**88 038 (82 149 to 94 853)**	**62 461 (57 689 to 67 739)**	**2 835.1 (2 697.2 to 2 992.8)**	**1 479.6 (1 367.3 to 1 604.0)**
**Transport injuries**	**672 (632 to 712)**	**659 (601 to 718)**	**25.5 (24.2 to 26.8)**	**15.7 (14.3 to 17.1)**	**36 850 (34 269 to 39 400)**	**32 729 (29 716 to 35 701)**	**1 233.9 (1 155.5 to 1 304.8)**	**775.9 (703.6 to 846.2)**
Road injuries	641 (602 to 678)	619 (566 to 674)	24.4 (23.0 to 25.5)	14.7 (13.5 to 16.0)	35 070 (32 645 to 37 452)	30 795 (28 062 to 33 580)	1 176.3 (1 100.4 to 1 248.6)	729.9 (663.5 to 796.6)
Pedestrian road injuries	312 (290 to 331)	289 (264 to 316)	12.4 (11.7 to 13.1)	6.9 (6.3 to 7.5)	16 296 (14 827 to 17 602)	13 174 (11 849 to 14 582)	545.3 (503.1 to 582.2)	313.5 (282.1 to 346.4)
Cyclist road injuries	16 (14 to 17)	20 (18 to 23)	0.6 (0.5 to 0.6)	0.5 (0.4 to 0.5)	896 (786 to 1 003)	978 (858 to 1 103)	29.4 (26.3 to 32.6)	23.2 (20.4 to 26.2)
Motorcyclist road injuries	73 (66 to 83)	64 (55 to 74)	2.6 (2.4 to 2.9)	1.5 (1.3 to 1.7)	4 279 (3 816 to 4 789)	3 640 (3 092 to 4 216)	143.0 (128.6 to 159.2)	85.5 (72.7 to 98.9)
Motor vehicle road injuries	214 (194 to 232)	225 (201 to 250)	7.8 (7.1 to 8.5)	5.4 (4.8 to 5.9)	12 131 (10 998 to 13 208)	11 974 (10 611 to 13 456)	411.6 (372.4 to 447.3)	283.3 (251.2 to 318.1)
Other road injuries	27 (23 to 31)	21 (18 to 24)	1.0 (0.8 to 1.1)	0.5 (0.4 to 0.6)	1 529 (1 291 to 1 807)	1 034 (877 to 1 215)	49.0 (41.6 to 57.5)	24.5 (20.7 to 28.7)
Other transport injuries	31 (28 to 35)	40 (35 to 45)	1.2 (1.1 to 1.3)	0.9 (0.8 to 1.1)	1 779 (1 574 to 1 998)	1 934 (1 665 to 2 230)	57.6 (51.4 to 63.8)	46.0 (39.7 to 53.1)
**Unintentional injuries**	**944 (893 to 1 001)**	**733 (685 to 788)**	**37.1 (35.8 to 38.6)**	**17.2 (16.0 to 18.5)**	**51 188 (47 083 to 56 034)**	**29 722 (27 096 to 32 732)**	**1 601.2 (1 509.9 to 1 712.1)**	**703.7 (642.0 to 774.1)**
Falls	198 (189 to 208)	250 (232 to 269)	10.5 (10.0 to 11.0)	5.8 (5.4 to 6.2)	6 937 (6 509 to 7 412)	6 024 (5 496 to 6 522)	275.2 (261.2 to 289.2)	143.4 (130.7 to 155.1)
Drowning	181 (168 to 198)	106 (96 to 116)	6.1 (5.7 to 6.5)	2.5 (2.3 to 2.7)	11 141 (10 125 to 12 439)	5 945 (5 327 to 6 607)	330.2 (305.8 to 361.0)	140.1 (125.6 to 155.7)
Fire, heat, and hot substances	70 (64 to 78)	41 (38 to 44)	2.7 (2.6 to 2.9)	1.0 (0.9 to 1.0)	3 958 (3 405 to 4 681)	1 458 (1 301 to 1 621)	115.9 (108.1 to 131.5)	34.5 (30.8 to 38.4)
Poisonings	33 (30 to 37)	17 (15 to 18)	1.1 (1.0 to 1.2)	0.4 (0.4 to 0.4)	2 154 (1 892 to 2 479)	878 (779 to 982)	60.8 (54.6 to 68.5)	20.8 (18.4 to 23.2)
Poisoning by carbon monoxide	9 (8 to 11)	5 (5 to 6)	0.3 (0.3 to 0.3)	0.1 (0.1 to 0.1)	595 (517 to 689)	270 (234 to 306)	17.2 (15.2 to 19.7)	6.4 (5.5 to 7.2)
Poisoning by other means	24 (21 to 27)	12 (10 to 13)	0.8 (0.7 to 0.8)	0.3 (0.2 to 0.3)	1 560 (1 352 to 1 828)	608 (538 to 686)	43.6 (38.7 to 49.7)	14.4 (12.7 to 16.2)
Exposure to mechanical forces	166 (157 to 176)	79 (73 to 87)	6.2 (5.9 to 6.6)	1.9 (1.7 to 2.1)	9 296 (8 676 to 9 957)	3 678 (3 349 to 4 073)	307.8 (289.0 to 327.6)	87.6 (79.7 to 97.0)
Unintentional firearm injuries	82 (68 to 89)	30 (23 to 35)	3.0 (2.5 to 3.2)	0.7 (0.5 to 0.8)	4 699 (3 915 to 5 159)	1 519 (1 027 to 1 786)	155.6 (129.5 to 170.5)	36.1 (24.4 to 42.5)
Other exposure to mechanical forces	84 (77 to 99)	49 (44 to 59)	3.2 (3.0 to 3.8)	1.2 (1.1 to 1.4)	4 597 (4 134 to 5 468)	2 159 (1 920 to 2 653)	152.2 (138.7 to 180.7)	51.5 (45.9 to 63.3)
Adverse effects of medical treatment	47 (41 to 52)	49 (43 to 74)	2.0 (1.8 to 2.2)	1.1 (1.0 to 1.7)	2 947 (1 952 to 2 724)	1 614 (1 358 to 2 508)	72.2 (61.5 to 81.1)	38.2 (32.4 to 58.1)
Animal contact	66 (57 to 76)	29 (26 to 32)	2.1 (1.9 to 2.4)	0.7 (0.6 to 0.8)	4 351 (3 601 to 5 217)	1 319 (1 150 to 1 520)	115.9 (99.4 to 135.6)	31.2 (27.2 to 36.0)
Venomous animal contact	55 (46 to 65)	22 (20 to 25)	1.7 (1.5 to 2.0)	0.5 (0.5 to 0.6)	3 734 (3 021 to 4 557)	1 032 (881 to 1 204)	97.8 (81.5 to 116.4)	24.4 (20.9 to 28.5)
Non-venomous animal contact	11 (7 to 13)	7 (6 to 9)	0.4 (0.3 to 0.4)	0.2 (0.1 to 0.2)	618 (393 to 762)	287 (236 to 376)	18.0 (11.6 to 21.6)	6.8 (5.6 to 8.9)
Foreign body	107 (98 to 118)	121 (109 to 136)	3.7 (3.4 to 3.9)	2.9 (2.6 to 3.2)	6 716 (5 950 to 7 609)	6 860 (5 924 to 8 015)	189.7 (172.2 to 210.3)	161.7 (139.7 to 188.5)
Pulmonary aspiration and foreign body in airway	103 (94 to 113)	120 (108 to 134)	3.5 (3.3 to 3.8)	2.8 (2.5 to 3.2)	6 417 (5 683 to 7 281)	6 759 (5 842 to 7 918)	181.4 (164.1 to 201.3)	159.3 (137.8 to 186.4)
Foreign body in eyes								
Foreign body in other body part	4 (4 to 6)	2 (1 to 4)	0.1 (0.1 to 0.2)	0.0 (0.0 to 0.1)	299 (238 to 387)	101 (75 to 258)	8.3 (6.9 to 11.0)	2.4 (1.8 to 6.1)
Environmental heat and cold exposure	22 (20 to 23)	9 (8 to 10)	0.9 (0.9 to 1.0)	0.2 (0.2 to 0.2)	1 081 (970 to 1 218)	330 (295 to 361)	35.7 (32.8 to 39.0)	7.9 (7.1 to 8.7)
Exposure to forces of nature	1 (1 to 1)	2 (2 to 2)	0.0 (0.0 to 0.0)	0.0 (0.0 to 0.0)	75 (69 to 83)	86 (78 to 95)	2.3 (2.1 to 2.5)	2.0 (1.8 to 2.2)
Other unintentional injuries	52 (48 to 57)	30 (27 to 33)	1.8 (1.7 to 1.9)	0.7 (0.6 to 0.8)	3 137 (2 884 to 3 442)	1 530 (1 350 to 1 717)	95.8 (88.6 to 103.7)	36.2 (32.3 to 40.7)

Cause	Incidence: All-age counts (95% UI) 1990	Incidence: All-age counts (95% UI) 2017	Incidence: Age-standardised rates per 100,000 (95% UI) 1990	Incidence: Age-standardised rates per 100,000 (95% UI) 2017	Prevalence: All-age counts (95% UI) 1990	Prevalence: All-age counts (95% UI) 2017	Prevalence: Age-standardised rates per 100,000 (95% UI) 1990	Prevalence: Age-standardised rates per 100,000 (95% UI) 2017
**Transport and unintentional injuries**	**112 799 (103 906 to 122 632)**	**159 515 (145 633 to 174 504)**	**4 034.7 (3 697.2 to 4 377.9)**	**3 768.8 (3 439.9 to 4 132.5)**	**214 191 (201 444 to 229 025)**	**335 613 (315 583 to 362 501)**	**9 285.0 (8 747.2 to 9 916.6)**	**7 989.3 (7 515.2 to 8 624.6)**
**Transport injuries**	**8 120 (6 959 to 9 477)**	**16 498 (14 279 to 18 873)**	**290.5 (251.6 to 331.6)**	**391.1 (338.6 to 448.1)**	**20 453 (19 116 to 21 800)**	**48 627 (45 293 to 52 319)**	**923.8 (863.0 to 986.2)**	**1 166.8 (1 087.2 to 1 255.7)**
Road injuries	7 574 (6 435 to 8 897)	16 086 (13 856 to 18 479)	272.2 (234.3 to 313.6)	381.3 (328.3 to 438.7)	17 953 (16 720 to 19 201)	46 496 (43 292 to 50 106)	818.8 (762.5 to 876.2)	1 115.8 (1 037.4 to 1 202.8)
Pedestrian road injuries	1 225 (992 to 1 522)	2 009 (1 611 to 2 451)	46.0 (37.8 to 55.6)	47.7 (38.4 to 58.1)	4 607 (3 970 to 5 396)	9 605 (8 377 to 11 109)	213.9 (185.3 to 247.1)	229.8 (200.1 to 266.2)
Cyclist road injuries	1 078 (802 to 1 420)	2 043 (1 588 to 2 579)	35.0 (27.2 to 44.5)	48.1 (37.5 to 60.6)	1 838 (1 547 to 2 172)	4 640 (3 947 to 5 448)	78.7 (66.7 to 93.8)	110.8 (94.3 to 130.1)
Motorcyclist road injuries	924 (722 to 1 165)	1 531 (1 168 to 1 952)	32.2 (25.2 to 39.9)	36.0 (27.6 to 45.9)	3 506 (3 046 to 4 058)	7 251 (6 202 to 8 559)	157.1 (136.2 to 181.7)	174.6 (149.3 to 206.2)
Motor vehicle road injuries	2 762 (2 251 to 3 322)	7 989 (6 709 to 9 337)	102.7 (84.4 to 121.7)	190.9 (159.8 to 223.9)	5 081 (4 450 to 5 892)	18 906 (16 519 to 22 018)	238.1 (206.3 to 277.2)	455.0 (397.2 to 530.0)
Other road injuries	1 585 (1 210 to 1 992)	2 514 (2 011 to 3 071)	56.3 (44.7 to 68.8)	59.3 (47.4 to 72.3)	2 922 (2 449 to 3 526)	6 094 (5 141 to 7 320)	131.6 (110.1 to 157.6)	145.7 (122.6 to 175.4)
Other transport injuries	546 (425 to 718)	412 (317 to 529)	18.4 (15.2 to 22.4)	9.8 (7.5 to 12.5)	2 500 (2 077 to 2 926)	2 131 (1 830 to 2 439)	105.1 (89.1 to 121.4)	53.0 (48.8 to 58.3)
**Unintentional injuries**	**104 680 (96 133 to 114 522)**	**143 017 (129 683 to 158 076)**	**3 744.2 (3 415.7 to 4 093.4)**	**3 377.7 (3 059.4 to 3 729.4)**	**193 738 (181 093 to 207 804)**	**286 985 (266 925 to 313 363)**	**8 361.2 (7 826.5 to 8 979.0)**	**6 822.5 (6 348.0 to 7 461.5)**
Falls	41 960 (36 733 to 47 720)	76 946 (68 158 to 86 845)	1 623.7 (1 445.1 to 1 823.7)	1 808.3 (1 602.5 to 2 040.7)	72 845 (64 640 to 83 355)	151 167 (135 390 to 171 877)	3 312.8 (2 967.7 to 3 803.2)	3 579.3 (3 205.4 to 4 061.8)
Drowning	147 (127 to 167)	85 (67 to 105)	4.9 (4.3 to 5.6)	2.0 (1.6 to 2.5)	689 (609 to 790)	537 (472 to 617)	31.5 (27.9 to 35.9)	12.8 (11.3 to 14.8)
Fire, heat, and hot substances	4 014 (3 163 to 5 013)	3 296 (2 728 to 3 980)	106.9 (86.8 to 129.6)	77.8 (64.3 to 94.0)	38 472 (31 907 to 48 133)	39 996 (32 907 to 49 514)	1 476.3 (1 238.6 to 1 801.4)	939.8 (785.3 to 1 179.1)
Poisonings	1 059 (766 to 1 488)	1 010 (720 to 1 389)	25.5 (19.0 to 34.7)	23.7 (16.9 to 32.6)	882 (667 to 1 119)	1 154 (858 to 1 460)	30.4 (23.4 to 38.3)	27.4 (20.4 to 34.6)
Poisoning by carbon monoxide	185 (119 to 290)	141 (79 to 225)	5.1 (3.5 to 7.3)	3.3 (1.9 to 5.3)	120 (92 to 155)	104 (81 to 134)	4.3 (3.4 to 5.5)	2.5 (1.9 to 3.2)
Poisoning by other means	868 (610 to 1 260)	868 (600 to 1 237)	20.4 (14.7 to 28.8)	20.4 (14.1 to 28.6)	763 (549 to 991)	1 050 (749 to 1 350)	26.1 (19.0 to 33.6)	24.9 (17.8 to 32.0)
Exposure to mechanical forces	24 513 (20 737 to 29 044)	24 036 (20 642 to 27 979)	821.3 (708.4 to 963.6)	570.7 (491.1 to 665.5)	49 063 (43 206 to 56 193)	60 888 (53 369 to 70 086)	2 179.0 (1 920.1 to 2 475.6)	1 462.1 (1 282.0 to 1 682.6)
Unintentional firearm injuries	1 905 (1 419 to 2 516)	1 348 (1 008 to 1 744)	63.9 (47.8 to 83.2)	32.0 (23.9 to 41.4)	7 190 (5 720 to 8 711)	6 827 (5 535 to 8 236)	326.6 (263.8 to 398.2)	164.3 (133.0 to 198.1)
Other exposure to mechanical forces	22 609 (19 080 to 26 857)	22 688 (19 494 to 26 417)	757.3 (649.9 to 890.3)	538.8 (463.7 to 628.3)	41 873 (35 626 to 49 223)	54 061 (46 245 to 63 543)	1 852.4 (1 585.8 to 2 171.1)	1 297.8 (1 110.6 to 1 526.2)
Adverse effects of medical treatment	2 520 (2 149 to 2 886)	5 310 (4 550 to 6 040)	97.6 (84.9 to 110.9)	127.0 (107.5 to 144.6)	193 (150 to 240)	406 (314 to 499)	7.5 (5.7 to 9.2)	9.7 (7.5 to 11.9)
Animal contact	13 861 (11 970 to 16 117)	12 953 (11 478 to 14 715)	453.8 (402.3 to 513.8)	307.1 (272.2 to 349.3)	14 245 (12 374 to 16 357)	15 868 (14 028 to 17 883)	598.2 (526.1 to 674.2)	378.3 (334.3 to 426.3)
Venomous animal contact	5 504 (4 370 to 6 760)	5 289 (4 283 to 6 388)	180.6 (145.2 to 218.1)	125.4 (101.7 to 151.6)	3 692 (3 077 to 4 410)	4 014 (3 369 to 4 654)	147.7 (124.5 to 173.7)	95.7 (80.1 to 110.9)
Non-venomous animal contact	8 356 (6 976 to 9 960)	7 664 (6 487 to 9 078)	273.2 (232.1 to 321.7)	181.7 (153.7 to 214.9)	10 553 (8 727 to 12 643)	11 854 (10 127 to 13 831)	450.5 (379.3 to 526.8)	282.6 (241.5 to 329.5)
Foreign body	9 649 (4 955 to 15 553)	14 046 (6 022 to 24 879)	375.9 (171.1 to 640.8)	335.4 (142.5 to 594.8)	3 912 (2 929 to 5 332)	5 194 (3 735 to 7 199)	162.6 (123.3 to 219.3)	121.9 (88.2 to 171.9)
Pulmonary aspiration and foreign body in airway	212 (146 to 303)	115 (84 to 158)	5.7 (4.2 to 7.6)	2.7 (2.0 to 3.7)	141 (104 to 188)	97 (79 to 120)	5.0 (3.9 to 6.5)	2.3 (1.9 to 2.8)
Foreign body in eyes	8 787 (4 112 to 14 639)	13 402 (5 357 to 24 336)	351.3 (144.5 to 612.2)	320.2 (126.8 to 580.9)	1 771 (906 to 3 044)	3 058 (1 735 to 4 991)	80.1 (44.9 to 131.8)	72.5 (40.5 to 119.8)
Foreign body in other body part	650 (395 to 1 027)	529 (364 to 699)	19.0 (12.2 to 28.5)	12.5 (8.6 to 16.5)	2 000 (1 606 to 2 526)	1 979 (1 622 to 2 501)	77.5 (63.3 to 96.2)	47.0 (38.6 to 59.4)
Environmental heat and cold exposure	1 500 (1 117 to 1 997)	681 (540 to 878)	50.7 (40.2 to 62.4)	16.2 (12.7 to 20.6)	5 198 (4 259 to 6 279)	2 596 (2 236 to 3 047)	215.6 (181.4 to 255.3)	61.6 (53.0 to 72.3)
Exposure to forces of nature	197 (161 to 247)	114 (97 to 137)	5.7 (4.8 to 6.9)	2.7 (2.3 to 3.2)	748 (528 to 1 005)	1 790 (1 256 to 2 464)	23.9 (17.3 to 32.0)	43.1 (30.3 to 59.3)
Other unintentional injuries	5 265 (4 299 to 6 540)	4 581 (3 758 to 5 494)	178.2 (149.8 to 211.2)	106.7 (88.6 to 129.5)	7 500 (6 487 to 8 640)	8 048 (7 009 to 9 182)	323.3 (279.0 to 369.5)	192.5 (167.5 to 220.0)

Cause	Years lived with disability: All-age counts (95% UI) 1990	Years lived with disability: All-age counts (95% UI) 2017	Years lived with disability: Age-standardised rates per 100,000 (95% UI) 1990	Years lived with disability: Age-standardised rates per 100,000 (95% UI) 2017	Disability adjusted life years: All-age counts (95% UI) 1990	Disability adjusted life years: All-age counts (95% UI) 2017	Disability adjusted life years: Age-standardised rates per 100,000 (95% UI) 1990	Disability adjusted life years: Age-standardised rates per 100,000 (95% UI) 2017
**Transport and unintentional injuries**	**9 388 (6 818 to 12 368)**	**14 613 (10 611 to 19 557)**	**401.0 (290.4 to 529.1)**	**347.4 (252.3 to 464.4)**	**97 426 (91 014 to 105 675)**	**77 064 (70 520 to 84 194)**	**3 236.1 (3 055.9 to 3 451.0)**	**1 827.0 (1 671.2 to 1 997.2)**
**Transport injuries**	**1 436 (1 062 to 1 892)**	**3 032 (2 205 to 4 040)**	**62.7 (46.1 to 82.5)**	**72.8 (53.0 to 97.0)**	**38 285 (35 750 to 40 810)**	**35 761 (32 591 to 39 024)**	**1 296.6 (1 214.2 to 1 367.6)**	**848.7 (774.5 to 928.0)**
Road injuries	1 269 (936 to 1 675)	2 915 (2 119 to 3 878)	56.1 (41.3 to 73.8)	70.0 (50.9 to 93.2)	36 340 (33 916 to 38 731)	33 710 (30 839 to 36 855)	1 232.4 (1 153.2 to 1 299.9)	799.9 (731.1 to 873.9)
Pedestrian road injuries	338 (239 to 456)	626 (441 to 855)	15.1 (10.8 to 20.5)	15.0 (10.6 to 20.5)	16 573 (15 168 to 17 922)	13 800 (12 533 to 15 223)	558.4 (517.3 to 596.0)	328.4 (298.1 to 362.7)
Cyclist road injuries	134 (95 to 176)	290 (203 to 385)	5.6 (3.9 to 7.3)	6.9 (4.8 to 9.2)	1 030 (917 to 1 150)	1 264 (1 118 to 1 430)	35.0 (31.4 to 38.7)	30.1 (26.6 to 34.1)
Motorcyclist road injuries	252 (177 to 339)	457 (314 to 619)	11.0 (7.7 to 14.7)	11.0 (7.6 to 14.9)	4 531 (4 050 to 5 039)	4 097 (3 540 to 4 702)	153.9 (138.9 to 170.6)	96.5 (83.4 to 110.6)
Motor vehicle road injuries	373 (260 to 496)	1 227 (887 to 1 622)	16.9 (12.2 to 22.2)	29.6 (21.4 to 39.1)	12 504 (11 326 to 13 569)	13 201 (11 799 to 14 586)	428.5 (389.2 to 464.9)	312.8 (279.7 to 345.1)
Other road injuries	172 (124 to 233)	315 (223 to 432)	7.5 (5.4 to 10.1)	7.5 (5.3 to 10.3)	1 701 (1 445 to 1 982)	1 349 (1 164 to 1 572)	56.5 (48.5 to 64.9)	32.0 (27.7 to 37.1)
Other transport injuries	166 (118 to 221)	118 (84 to 156)	6.7 (4.8 to 8.8)	2.8 (2.0 to 3.7)	1 945 (1 737 to 2 161)	2 051 (1 774 to 2 348)	64.3 (57.7 to 71.1)	48.8 (42.2 to 56.1)
**Unintentional injuries**	**7 953 (5 761 to 10 489)**	**11 581 (8 314 to 15 581)**	**338.3 (244.6 to 448.0)**	**274.6 (197.1 to 368.7)**	**59 141 (54 446 to 64 883)**	**41 303 (37 071 to 46 214)**	**1 939.5 (1 807.0 to 2 099.6)**	**978.3 (877.8 to 1 094.7)**
Falls	4 540 (3 285 to 5 919)	8 122 (5 889 to 10 764)	200.4 (145.8 to 259.9)	191.9 (138.9 to 254.6)	11 476 (10 193 to 12 872)	14 146 (11 879 to 16 814)	475.6 (420.3 to 534.4)	335.3 (281.5 to 398.9)
Drowning	40 (28 to 54)	29 (20 to 39)	1.8 (1.2 to 2.4)	0.7 (0.5 to 0.9)	11 180 (10 165 to 12 472)	5 974 (5 351 to 6 641)	331.9 (307.2 to 363.0)	140.8 (126.3 to 156.6)
Fire, heat, and hot substances	850 (544 to 1 327)	785 (471 to 1 255)	31.9 (20.1 to 49.5)	18.7 (11.3 to 29.9)	4 813 (4 180 to 5 608)	2 243 (1 880 to 2 747)	147.8 (130.4 to 169.6)	53.2 (44.6 to 65.2)
Poisonings	104 (64 to 160)	133 (80 to 198)	3.5 (2.2 to 5.3)	3.1 (1.9 to 4.7)	2 259 (1 994 to 2 594)	1 011 (905 to 1 121)	64.3 (57.9 to 72.0)	23.9 (21.4 to 26.5)
Poisoning by carbon monoxide	13 (8 to 20)	10 (6 to 16)	0.4 (0.3 to 0.7)	0.2 (0.1 to 0.4)	607 (530 to 700)	280 (244 to 317)	17.7 (15.7 to 20.1)	6.6 (5.8 to 7.5)
Poisoning by other means	92 (55 to 141)	123 (73 to 186)	3.1 (1.9 to 4.7)	2.9 (1.7 to 4.4)	1 651 (1 450 to 1 918)	731 (644 to 824)	46.6 (41.7 to 52.8)	17.3 (15.2 to 19.5)
Exposure to mechanical forces	1 184 (821 to 1 689)	1 347 (920 to 1 942)	51.5 (35.6 to 73.1)	32.3 (22.1 to 46.7)	10 480 (9 796 to 11 327)	5 025 (4 443 to 5 726)	359.3 (335.7 to 390.2)	120.0 (106.1 to 137.0)
Unintentional firearm injuries	152 (101 to 227)	129 (84 to 203)	6.7 (4.4 to 10.1)	3.1 (2.0 to 4.9)	4 850 (4 049 to 5 312)	1 649 (1 137 to 1 916)	162.3 (135.6 to 177.4)	39.2 (27.1 to 45.6)
Other exposure to mechanical forces	1 033 (714 to 1 463)	1 218 (828 to 1 766)	44.9 (31.1 to 63.7)	29.2 (19.9 to 42.4)	5 630 (5 089 to 6 506)	3 376 (2 899 to 4 089)	197.0 (177.8 to 227.5)	80.8 (69.3 to 97.8)
Adverse effects of medical treatment	26 (16 to 39)	54 (34 to 82)	1.0 (0.6 to 1.5)	1.3 (0.8 to 2.0)	2 373 (1 978 to 2 744)	1 669 (1 419 to 2 566)	73.2 (62.4 to 82.2)	39.5 (33.6 to 60.5)
Animal contact	379 (255 to 531)	354 (234 to 510)	14.6 (9.8 to 20.5)	8.4 (5.6 to 12.1)	4 731 (3 974 to 5 639)	1 673 (1 463 to 1 919)	130.4 (113.4 to 151.3)	39.6 (34.6 to 45.4)
Venomous animal contact	212 (135 to 302)	182 (118 to 258)	7.5 (4.9 to 10.6)	4.3 (2.8 to 6.1)	3 946 (3 232 to 4 781)	1 214 (1 054 to 1 401)	105.3 (88.5 to 124.9)	28.8 (25.0 to 33.2)
Non-venomous animal contact	167 (104 to 263)	172 (103 to 277)	7.0 (4.4 to 11.1)	4.1 (2.5 to 6.6)	785 (531 to 966)	460 (366 to 601)	25.1 (17.4 to 31.3)	10.9 (8.7 to 14.3)
Foreign body	258 (168 to 383)	310 (188 to 502)	10.5 (6.8 to 15.7)	7.4 (4.5 to 12.0)	6 974 (6 194 to 7 876)	7 170 (6 209 to 8 335)	200.2 (181.6 to 221.3)	169.1 (146.6 to 196.2)
Pulmonary aspiration and foreign body in airway	8 (6 to 11)	4 (3 to 6)	0.3 (0.2 to 0.4)	0.1 (0.1 to 0.1)	6 425 (5 689 to 7 288)	6 763 (5 848 to 7 921)	181.7 (164.4 to 202.2)	159.4 (138.0 to 186.5)
Foreign body in eyes	118 (59 to 217)	204 (108 to 375)	5.4 (2.9 to 9.7)	4.9 (2.5 to 8.9)	118 (59 to 217)	204 (108 to 375)	5.4 (2.9 to 9.7)	4.9 (2.5 to 8.9)
Foreign body in other body part	132 (92 to 176)	101 (72 to 134)	4.8 (3.5 to 6.4)	2.4 (1.7 to 3.2)	431 (350 to 532)	203 (155 to 365)	13.2 (10.9 to 16.1)	4.8 (3.7 to 8.6)
Environmental heat and cold exposure	212 (141 to 297)	101 (67 to 145)	8.7 (5.9 to 12.1)	2.4 (1.6 to 3.4)	1 292 (1 160 to 1 449)	431 (380 to 485)	44.3 (40.3 to 49.1)	10.3 (9.1 to 11.6)
Exposure to forces of nature	39 (26 to 56)	64 (45 to 91)	1.3 (0.9 to 1.7)	1.5 (1.1 to 2.2)	115 (99 to 131)	150 (130 to 178)	3.5 (3.1 to 4.0)	3.6 (3.1 to 4.2)
Other unintentional injuries	311 (223 to 410)	282 (200 to 381)	13.1 (9.5 to 17.4)	6.7 (4.8 to 9.1)	3 448 (3 174 to 3 763)	1 812 (1 629 to 2 015)	108.9 (100.3 to 117.8)	43.0 (38.7 to 47.8)

Death, YLL, incidence, prevalence, YLD, and DALY counts and rates per 100,000 for all injuries in Puebla in 1990 and 2017

Cause	Deaths: All-age counts (95% UI) 1990	Deaths: All-age counts (95% UI) 2017	Deaths: Age-standardised rates per 100,000 (95% UI) 1990	Deaths: Age-standardised rates per 100,000 (95% UI) 2017	Years of life lost: All-age counts (95% UI) 1990	Years of life lost: All-age counts (95% UI) 2017	Years of life lost: Age-standardised rates per 100,000 (95% UI) 1990	Years of life lost: Age-standardised rates per 100,000 (95% UI) 2017
**Transport and unintentional injuries**	**2 312 (2 204 to 2 432)**	**2 499 (2 349 to 2 661)**	**65.6 (63.6 to 67.8)**	**39.9 (37.6 to 42.4)**	**125 886 (117 668 to 135 438)**	**117 013 (108 818 to 125 680)**	**2 895.2 (2 749.8 to 3 060.7)**	**1 761.2 (1 639.6 to 1 885.9)**
**Transport injuries**	**967 (913 to 1 021)**	**1 115 (1 024 to 1 211)**	**26.6 (25.4 to 27.9)**	**17.3 (15.9 to 18.7)**	**53 269 (49 628 to 56 799)**	**56 155 (51 215 to 61 102)**	**1 266.4 (1 192.9 to 1 340.4)**	**838.0 (765.4 to 912.8)**
Road injuries	941 (887 to 994)	1 076 (988 to 1 169)	25.9 (24.6 to 27.1)	16.7 (15.4 to 18.1)	51 796 (48 262 to 55 261)	54 236 (49 506 to 59 123)	1 231.7 (1 160.1 to 1 304.1)	809.3 (739.1 to 881.8)
Pedestrian road injuries	473 (443 to 503)	501 (457 to 547)	13.7 (13.1 to 14.4)	8.0 (7.3 to 8.7)	24 850 (22 716 to 27 066)	23 271 (20 978 to 25 579)	594.8 (554.7 to 636.1)	352.7 (318.7 to 387.2)
Cyclist road injuries	27 (24 to 29)	47 (41 to 53)	0.7 (0.7 to 0.8)	0.7 (0.6 to 0.8)	1 490 (1 333 to 1 669)	2 204 (1 943 to 2 513)	35.8 (32.4 to 39.5)	33.4 (29.5 to 38.0)
Motorcyclist road injuries	129 (116 to 142)	130 (114 to 150)	3.3 (3.0 to 3.6)	1.9 (1.7 to 2.2)	7 573 (6 774 to 8 378)	7 498 (6 538 to 8 745)	177.6 (160.9 to 195.3)	108.9 (95.2 to 126.8)
Motor vehicle road injuries	284 (258 to 316)	367 (326 to 409)	7.4 (6.8 to 8.1)	5.6 (5.0 to 6.2)	16 286 (14 670 to 18 258)	19 708 (17 467 to 22 019)	386.7 (351.0 to 430.3)	291.1 (258.7 to 325.4)
Other road injuries	28 (24 to 32)	31 (27 to 36)	0.8 (0.7 to 0.9)	0.5 (0.4 to 0.6)	1 597 (1 346 to 1 852)	1 555 (1 331 to 1 829)	36.8 (31.4 to 42.2)	23.2 (19.9 to 27.2)
Other transport injuries	27 (25 to 30)	39 (34 to 45)	0.8 (0.7 to 0.8)	0.6 (0.5 to 0.7)	1 473 (1 332 to 1 635)	1 920 (1 646 to 2 201)	34.7 (31.8 to 38.4)	28.8 (24.8 to 32.9)
**Unintentional injuries**	**1 345 (1 268 to 1 428)**	**1 384 (1 312 to 1 466)**	**39.0 (37.6 to 40.6)**	**22.6 (21.4 to 23.9)**	**72 617 (66 750 to 79 465)**	**60 858 (56 766 to 65 705)**	**1 628.8 (1 531.4 to 1 737.2)**	**923.1 (861.0 to 993.6)**
Falls	304 (290 to 320)	388 (355 to 418)	11.9 (11.4 to 12.5)	6.8 (6.3 to 7.4)	10 559 (9 879 to 11 342)	10 468 (9 581 to 11 336)	310.5 (295.0 to 329.6)	170.0 (154.9 to 188.8)
Drowning	251 (232 to 273)	167 (152 to 183)	6.1 (5.7 to 6.5)	2.6 (2.3 to 2.8)	15 541 (14 071 to 17 285)	9 335 (8 351 to 10 339)	326.1 (300.5 to 355.1)	138.3 (124.1 to 153.6)
Fire, heat, and hot substances	108 (99 to 120)	72 (66 to 77)	3.1 (2.9 to 3.3)	1.2 (1.1 to 1.3)	6 046 (5 248 to 6 959)	2 593 (2 338 to 2 868)	126.7 (114.3 to 141.5)	40.2 (36.4 to 44.3)
Poisonings	65 (59 to 71)	41 (38 to 45)	1.5 (1.4 to 1.7)	0.6 (0.6 to 0.7)	4 144 (3 665 to 4 681)	2 125 (1 913 to 2 351)	83.6 (75.7 to 92.1)	31.8 (28.7 to 35.2)
Poisoning by carbon monoxide	24 (22 to 27)	18 (16 to 19)	0.6 (0.5 to 0.6)	0.3 (0.3 to 0.3)	1 495 (1 315 to 1 686)	890 (792 to 990)	31.2 (28.0 to 34.7)	13.3 (11.9 to 14.8)
Poisoning by other means	41 (36 to 45)	24 (21 to 26)	0.9 (0.9 to 1.0)	0.4 (0.3 to 0.4)	2 649 (2 294 to 3 023)	1 235 (1 096 to 1 391)	52.5 (46.6 to 58.5)	18.5 (16.5 to 20.8)
Exposure to mechanical forces	223 (211 to 238)	110 (101 to 121)	6.2 (5.9 to 6.5)	1.8 (1.6 to 1.9)	12 263 (11 465 to 13 182)	5 068 (4 615 to 5 583)	295.9 (278.5 to 315.4)	76.8 (70.0 to 84.4)
Unintentional firearm injuries	111 (102 to 124)	36 (27 to 40)	3.0 (2.7 to 3.3)	0.6 (0.4 to 0.6)	6 291 (5 724 to 7 012)	1 764 (1 345 to 2 022)	151.0 (138.1 to 167.9)	26.4 (20.0 to 30.2)
Other exposure to mechanical forces	112 (99 to 122)	74 (68 to 84)	3.2 (2.9 to 3.5)	1.2 (1.1 to 1.3)	5 972 (5 279 to 6 546)	3 304 (2 980 to 3 768)	144.9 (128.4 to 157.5)	50.4 (45.5 to 57.2)
Adverse effects of medical treatment	70 (60 to 77)	81 (71 to 111)	2.2 (2.0 to 2.4)	1.4 (1.2 to 1.9)	3 456 (2 800 to 4 010)	2 795 (2 384 to 4 083)	76.9 (65.3 to 86.2)	43.6 (37.5 to 63.9)
Animal contact	68 (57 to 83)	27 (24 to 29)	1.4 (1.3 to 1.7)	0.4 (0.4 to 0.5)	4 864 (3 956 to 5 986)	1 244 (1 095 to 1 419)	85.5 (71.7 to 103.0)	18.9 (16.8 to 21.5)
Venomous animal contact	57 (47 to 70)	19 (16 to 21)	1.2 (1.0 to 1.4)	0.3 (0.3 to 0.3)	4 256 (3 393 to 5 324)	900 (751 to 1 059)	72.8 (59.3 to 89.3)	13.7 (11.5 to 16.0)
Non-venomous animal contact	10 (9 to 15)	8 (7 to 11)	0.3 (0.2 to 0.4)	0.1 (0.1 to 0.2)	608 (492 to 917)	344 (292 to 480)	12.7 (10.5 to 18.2)	5.3 (4.5 to 7.4)
Foreign body	167 (152 to 184)	208 (186 to 238)	4.0 (3.8 to 4.3)	3.2 (2.9 to 3.6)	10 876 (9 566 to 12 302)	12 403 (10 554 to 14 812)	211.5 (191.7 to 233.8)	181.1 (155.0 to 215.1)
Pulmonary aspiration and foreign body in airway	158 (144 to 174)	203 (182 to 233)	3.8 (3.5 to 4.1)	3.1 (2.8 to 3.5)	10 240 (9 025 to 11 573)	12 143 (10 337 to 14 498)	198.8 (180.3 to 220.1)	177.3 (151.8 to 210.3)
Foreign body in eyes								
Foreign body in other body part	9 (7 to 11)	4 (4 to 8)	0.2 (0.2 to 0.2)	0.1 (0.1 to 0.1)	636 (442 to 760)	260 (209 to 460)	12.7 (9.3 to 14.7)	3.8 (3.1 to 6.8)
Environmental heat and cold exposure	32 (30 to 35)	16 (14 to 17)	1.0 (1.0 to 1.1)	0.3 (0.2 to 0.3)	1 556 (1 401 to 1 727)	599 (535 to 663)	38.0 (34.9 to 41.4)	9.4 (8.4 to 10.4)
Exposure to forces of nature	2 (1 to 3)	231 (213 to 251)	0.1 (0.0 to 0.1)	3.6 (3.3 to 3.9)	124 (88 to 171)	11 924 (10 873 to 13 083)	2.7 (2.0 to 3.7)	178.8 (163.2 to 195.7)
Other unintentional injuries	54 (50 to 58)	44 (39 to 49)	1.4 (1.3 to 1.5)	0.7 (0.6 to 0.8)	3 189 (2 929 to 3 469)	2 304 (2 036 to 2 598)	71.4 (66.1 to 77.2)	34.2 (30.3 to 38.5)

Cause	Incidence: All-age counts (95% UI) 1990	Incidence: All-age counts (95% UI) 2017	Incidence: Age-standardised rates per 100,000 (95% UI) 1990	Incidence: Age-standardised rates per 100,000 (95% UI) 2017	Prevalence: All-age counts (95% UI) 1990	Prevalence: All-age counts (95% UI) 2017	Prevalence: Age-standardised rates per 100,000 (95% UI) 1990	Prevalence: Age-standardised rates per 100,000 (95% UI) 2017
**Transport and unintentional injuries**	**164 490 (151 882 to 178 174)**	**285 238 (263 196 to 307 926)**	**4 273.0 (3 944.5 to 4 620.6)**	**4 519.1 (4 180.7 to 4 883.0)**	**314 328 (295 885 to 336 337)**	**570 072 (535 030 to 614 251)**	**10 026.3 (9 452.3 to 10 714.1)**	**9 310.1 (8 735.8 to 10 012.4)**
**Transport injuries**	**12 922 (11 010 to 15 068)**	**30 888 (26 805 to 35 326)**	**332.8 (288.8 to 381.9)**	**471.3 (411.0 to 538.6)**	**31 286 (29 157 to 33 299)**	**84 943 (78 838 to 91 462)**	**1 037.5 (968.4 to 1 107.3)**	**1 382.8 (1 283.5 to 1 489.0)**
Road injuries	12 296 (10 443 to 14 490)	30 302 (26 225 to 34 713)	318.7 (275.0 to 365.8)	464.1 (403.1 to 529.1)	28 481 (26 536 to 30 460)	81 978 (75 941 to 88 393)	954.8 (888.6 to 1 020.3)	1 334.6 (1 237.2 to 1 437.9)
Pedestrian road injuries	1 990 (1 595 to 2 461)	3 934 (3 220 to 4 769)	54.2 (44.3 to 65.5)	61.4 (50.2 to 74.2)	7 320 (6 291 to 8 555)	17 610 (15 310 to 20 513)	249.0 (215.8 to 288.8)	289.6 (252.1 to 337.1)
Cyclist road injuries	2 219 (1 698 to 2 876)	5 198 (4 132 to 6 446)	52.3 (41.5 to 65.8)	79.2 (63.5 to 97.4)	3 733 (3 131 to 4 420)	11 308 (9 538 to 13 325)	117.2 (99.2 to 136.1)	181.4 (153.4 to 212.6)
Motorcyclist road injuries	1 595 (1 241 to 2 035)	2 601 (1 973 to 3 358)	39.9 (31.8 to 49.3)	38.8 (29.5 to 49.7)	5 772 (5 012 to 6 678)	11 612 (9 908 to 13 660)	193.0 (165.6 to 221.2)	186.2 (158.9 to 218.9)
Motor vehicle road injuries	4 949 (3 945 to 5 218)	14 971 (12 536 to 17 632)	116.8 (97.1 to 139.3)	228.6 (192.1 to 268.8)	7 734 (6 780 to 9 002)	33 083 (28 861 to 38 609)	268.1 (233.3 to 312.1)	540.2 (470.9 to 629.3)
Other road injuries	2 144 (1 616 to 2 716)	3 598 (2 818 to 4 468)	55.4 (43.4 to 67.8)	56.2 (44.0 to 69.5)	3 921 (3 269 to 4 722)	8 365 (7 014 to 10 053)	129.4 (108.0 to 154.6)	137.3 (115.1 to 164.6)
Other transport injuries	626 (444 to 890)	586 (417 to 807)	14.2 (10.9 to 18.5)	9.1 (6.6 to 12.5)	2 805 (2 311 to 3 370)	2 965 (2 520 to 3 428)	82.7 (69.2 to 96.8)	48.2 (41.3 to 55.5)
**Unintentional injuries**	**151 568 (139 337 to 164 818)**	**254 350 (233 694 to 276 521)**	**3 940.1 (3 624.1 to 4 290.8)**	**4 045.8 (3 715.3 to 4 395.2)**	**283 042 (264 580 to 304 981)**	**485 129 (449 818 to 529 466)**	**8 988.7 (8 401.8 to 9 673.1)**	**7 927.3 (7 358.1 to 8 638.6)**
Falls	64 646 (56 717 to 73 306)	131 716 (117 294 to 148 626)	1 833.7 (1 641.1 to 2 048.1)	2 152.7 (1 915.9 to 2 422.2)	110 965 (98 165 to 126 095)	253 828 (226 116 to 289 777)	3 731.4 (3 335.7 to 4 244.7)	4 224.8 (3 772.6 to 4 808.8)
Drowning	222 (193 to 254)	150 (116 to 190)	5.2 (4.6 to 6.0)	2.3 (1.8 to 2.9)	993 (877 to 1 137)	894 (783 to 1 046)	33.1 (29.5 to 37.5)	14.7 (12.9 to 17.2)
Fire, heat, and hot substances	6 221 (4 982 to 7 623)	6 629 (5 425 to 8 155)	119.4 (98.8 to 142.5)	99.8 (81.9 to 122.3)	57 540 (48 021 to 70 890)	75 536 (61 979 to 95 646)	1 612.0 (1 360.0 to 1 939.6)	1 188.6 (982.0 to 1 492.3)
Poisonings	1 970 (1 481 to 2 545)	2 338 (1 745 to 3 078)	34.3 (26.3 to 43.4)	34.5 (25.9 to 45.4)	1 614 (1 252 to 1 987)	2 588 (1 938 to 3 226)	40.3 (31.6 to 49.3)	39.7 (29.8 to 49.4)
Poisoning by carbon monoxide	361 (243 to 535)	275 (176 to 412)	7.0 (5.1 to 9.7)	4.2 (2.7 to 6.2)	284 (181 to 390)	196 (152 to 250)	6.0 (4.7 to 7.7)	3.1 (2.4 to 3.9)
Poisoning by other means	1 610 (1 182 to 2 112)	2 063 (1 507 to 2 723)	27.2 (20.5 to 35.2)	30.3 (22.2 to 40.2)	1 381 (1 018 to 1 733)	2 391 (1 727 to 3 020)	34.2 (25.6 to 42.6)	36.6 (26.5 to 46.2)
Exposure to mechanical forces	36 267 (31 097 to 42 454)	38 856 (33 368 to 44 959)	876.6 (759.7 to 1 016.2)	590.9 (509.4 to 681.5)	70 176 (61 754 to 80 621)	91 845 (79 879 to 106 078)	2 299.8 (2 021.9 to 2 629.4)	1 484.8 (1 292.0 to 1 711.0)
Unintentional firearm injuries	2 906 (2 353 to 3 859)	1 669 (1 228 to 2 217)	69.7 (52.3 to 90.6)	25.3 (18.6 to 33.5)	10 467 (8 366 to 12 708)	8 143 (6 625 to 9 864)	350.9 (282.6 to 427.0)	133.0 (108.5 to 161.0)
Other exposure to mechanical forces	33 361 (28 467 to 39 171)	37 187 (31 950 to 42 929)	807.0 (699.0 to 938.9)	565.6 (488.1 to 651.7)	59 709 (50 710 to 70 384)	83 703 (71 723 to 98 459)	1 948.9 (1 658.2 to 2 295.4)	1 351.8 (1 160.0 to 1 588.5)
Adverse effects of medical treatment	3 001 (2 552 to 3 482)	5 920 (5 039 to 6 882)	86.6 (74.9 to 99.2)	95.6 (81.4 to 110.6)	230 (179 to 285)	459 (350 to 566)	6.6 (5.1 to 8.2)	7.9 (5.6 to 9.1)
Animal contact	17 639 (15 068 to 20 728)	23 850 (20 628 to 27 543)	409.6 (359.5 to 466.3)	373.9 (322.1 to 427.9)	17 849 (15 347 to 20 673)	28 260 (24 937 to 32 127)	543.3 (474.7 to 632.3)	458.4 (405.7 to 519.0)
Venomous animal contact	7 211 (5 685 to 8 961)	10 553 (8 512 to 12 876)	167.2 (134.4 to 203.9)	164.1 (132.7 to 200.6)	4 894 (4 016 to 5 902)	8 217 (6 881 to 9 666)	140.9 (117.4 to 166.6)	131.9 (110.9 to 154.5)
Non-venomous animal contact	10 428 (8 622 to 12 646)	13 297 (10 921 to 16 117)	242.4 (203.2 to 289.3)	207.8 (170.8 to 250.5)	12 954 (10 598 to 15 684)	20 043 (16 333 to 23 707)	400.4 (335.5 to 469.7)	326.5 (276.6 to 383.3)
Foreign body	12 666 (6 938 to 19 719)	19 378 (8 775 to 32 275)	355.5 (163.6 to 585.7)	310.7 (136.9 to 526.4)	6 159 (4 792 to 7 990)	6 541 (4 686 to 9 218)	183.4 (145.3 to 237.4)	107.6 (77.5 to 150.3)
Pulmonary aspiration and foreign body in airway	374 (259 to 529)	207 (147 to 281)	6.9 (5.0 to 9.3)	3.2 (2.3 to 4.3)	240 (174 to 324)	170 (136 to 212)	6.2 (4.7 to 8.2)	2.7 (2.2 to 3.3)
Foreign body in eyes	11 065 (5 280 to 17 997)	18 484 (7 908 to 31 451)	323.4 (129.1 to 556.6)	295.8 (123.3 to 514.1)	2 210 (1 140 to 3 789)	4 001 (2 198 to 6 562)	73.5 (41.5 to 122.3)	66.8 (37.2 to 108.3)
Foreign body in other body part	1 228 (793 to 1 896)	688 (495 to 912)	25.2 (17.3 to 36.4)	10.7 (7.8 to 14.1)	3 709 (2 956 to 4 711)	2 370 (1 990 to 2 837)	103.7 (83.8 to 129.4)	38.1 (32.1 to 45.4)
Environmental heat and cold exposure	2 089 (1 476 to 2 721)	1 076 (801 to 1 485)	50.8 (38.7 to 62.4)	17.2 (13.0 to 23.2)	6 924 (5 658 to 8 331)	4 044 (3 426 to 4 821)	212.6 (178.3 to 251.0)	66.2 (56.3 to 78.6)
Exposure to forces of nature	74 (56 to 98)	15 861 (13 387 to 19 043)	2.3 (1.6 to 3.1)	238.5 (202.2 to 284.9)	998 (716 to 1 348)	6 296 (4 693 to 8 310)	25.6 (18.8 to 34.5)	96.7 (72.2 to 127.2)
Other unintentional injuries	6 821 (5 598 to 8 383)	8 577 (7 288 to 10 174)	166.1 (140.5 to 196.8)	133.6 (111.9 to 155.5)	9 594 (8 255 to 11 067)	14 846 (12 833 to 17 092)	302.7 (262.2 to 346.8)	238.6 (206.8 to 278.7)

Cause	Years lived with disability: All-age counts (95% UI) 1990	Years lived with disability: All-age counts (95% UI) 2017	Years lived with disability: Age-standardised rates per 100,000 (95% UI) 1990	Years lived with disability: Age-standardised rates per 100,000 (95% UI) 2017	Disability adjusted life years: All-age counts (95% UI) 1990	Disability adjusted life years: All-age counts (95% UI) 2017	Disability adjusted life years: Age-standardised rates per 100,000 (95% UI) 1990	Disability adjusted life years: Age-standardised rates per 100,000 (95% UI) 2017
**Transport and unintentional injuries**	**13 854 (10 168 to 18 396)**	**24 482 (17 728 to 33 061)**	**435.1 (317.9 to 575.0)**	**399.2 (288.6 to 538.2)**	**139 740 (130 809 to 150 015)**	**141 495 (131 044 to 153 286)**	**3 330.3 (3 144.5 to 3 528.0)**	**2 160.4 (1 999.8 to 2 343.7)**
**Transport injuries**	**2 189 (1 596 to 2 898)**	**5 240 (3 802 to 6 965)**	**70.1 (51.3 to 92.8)**	**84.4 (61.3 to 112.1)**	**55 458 (51 839 to 58 898)**	**61 396 (56 153 to 67 133)**	**1 336.5 (1 260.3 to 1 407.2)**	**922.5 (847.6 to 1 008.0)**
Road injuries	2 002 (1 465 to 2 661)	5 078 (3 693 to 6 775)	64.8 (47.2 to 86.0)	81.8 (59.4 to 109.3)	53 798 (50 286 to 57 150)	59 314 (54 386 to 64 687)	1 296.5 (1 222.9 to 1 364.6)	891.1 (818.4 to 972.6)
Pedestrian road injuries	535 (379 to 720)	1 132 (802 to 1 542)	17.5 (12.4 to 23.6)	18.4 (13.0 to 25.1)	25 385 (23 308 to 27 576)	24 403 (22 052 to 26 888)	612.3 (571.7 to 653.5)	371.1 (336.6 to 408.2)
Cyclist road injuries	268 (190 to 349)	691 (483 to 926)	8.1 (5.8 to 10.6)	11.0 (7.7 to 14.7)	1 757 (1 582 to 1 949)	2 895 (2 553 to 3 277)	44.0 (39.7 to 48.6)	44.4 (39.2 to 50.2)
Motorcyclist road injuries	410 (288 to 552)	720 (494 to 979)	13.1 (9.2 to 17.8)	11.4 (7.8 to 15.6)	7 983 (7 189 to 8 786)	8 218 (7 217 to 9 427)	190.7 (173.2 to 207.8)	120.4 (106.3 to 137.9)
Motor vehicle road injuries	563 (407 to 744)	2 118 (1 533 to 2 820)	18.8 (13.7 to 24.8)	34.2 (24.7 to 45.3)	16 849 (15 292 to 18 884)	21 826 (19 600 to 24 343)	405.5 (369.6 to 450.0)	325.3 (292.4 to 362.1)
Other road injuries	227 (163 to 305)	417 (298 to 570)	7.2 (5.2 to 9.7)	6.8 (4.8 to 9.2)	1 823 (1 571 to 2 094)	1 972 (1 706 to 2 290)	44.0 (38.2 to 49.9)	30.0 (26.0 to 34.7)
Other transport injuries	188 (133 to 249)	162 (118 to 218)	5.3 (3.8 to 7.0)	2.6 (1.9 to 3.5)	1 661 (1 509 to 1 828)	2 082 (1 801 to 2 373)	40.0 (36.5 to 44.0)	31.4 (27.3 to 35.6)
**Unintentional injuries**	**11 665 (8 448 to 15 623)**	**19 242 (13 742 to 26 019)**	**365.0 (263.3 to 488.7)**	**314.8 (224.3 to 425.7)**	**84 282 (77 728 to 91 654)**	**80 100 (73 077 to 88 419)**	**1 993.8 (1 858.9 to 2 140.5)**	**1 237.9 (1 128.6 to 1 365.0)**
Falls	6 809 (4 934 to 8 835)	13 284 (9 557 to 17 549)	222.1 (161.2 to 288.4)	220.1 (157.7 to 292.6)	17 367 (15 353 to 19 490)	23 752 (19 993 to 28 010)	532.6 (471.6 to 599.5)	390.1 (327.3 to 461.1)
Drowning	57 (40 to 78)	48 (33 to 67)	1.8 (1.3 to 2.5)	0.8 (0.5 to 1.1)	15 598 (14 122 to 17 346)	9 383 (8 415 to 10 442)	327.9 (302.3 to 356.7)	139.1 (124.8 to 154.3)
Fire, heat, and hot substances	1 279 (809 to 2 000)	1 490 (892 to 2 379)	34.6 (21.8 to 54.4)	23.3 (14.0 to 37.3)	7 325 (6 443 to 8 508)	4 083 (3 419 to 5 062)	161.3 (144.2 to 184.1)	63.6 (53.4 to 78.7)
Poisonings	191 (139 to 285)	301 (183 to 456)	4.6 (2.9 to 6.8)	4.6 (2.8 to 6.9)	4 335 (3 851 to 4 883)	2 425 (2 174 to 2 690)	88.2 (79.8 to 97.4)	36.4 (32.7 to 40.3)
Poisoning by carbon monoxide	25 (15 to 39)	19 (12 to 30)	0.6 (0.4 to 0.9)	0.3 (0.2 to 0.5)	1 520 (1 339 to 1 721)	909 (814 to 1 007)	31.8 (28.6 to 35.4)	13.6 (12.2 to 15.1)
Poisoning by other means	166 (102 to 253)	281 (171 to 433)	4.0 (2.5 to 6.0)	4.3 (2.6 to 6.6)	2 815 (2 462 to 3 212)	1 516 (1 325 to 1 727)	56.4 (50.4 to 62.9)	22.8 (19.9 to 25.8)
Exposure to mechanical forces	1 686 (1 173 to 2 406)	2 014 (1 362 to 2 916)	54.0 (37.5 to 77.3)	32.4 (21.9 to 47.0)	13 950 (12 998 to 14 963)	7 082 (6 297 to 8 087)	349.9 (326.9 to 376.0)	109.2 (96.7 to 125.5)
Unintentional firearm injuries	221 (147 to 332)	150 (97 to 236)	7.2 (4.7 to 11.0)	2.4 (1.6 to 3.8)	6 513 (5 936 to 7 227)	1 914 (1 473 to 2 178)	158.2 (144.7 to 175.0)	28.8 (22.2 to 32.7)
Other exposure to mechanical forces	1 465 (1 015 to 2 090)	1 864 (1 262 to 2 702)	46.9 (32.5 to 67.0)	30.0 (20.3 to 43.5)	7 437 (6 679 to 8 234)	5 168 (4 454 to 6 128)	191.8 (171.6 to 212.2)	80.4 (69.1 to 95.7)
Adverse effects of medical treatment	31 (19 to 46)	60 (37 to 92)	0.9 (0.5 to 1.3)	1.0 (0.6 to 1.5)	3 486 (2 837 to 4 039)	2 855 (2 445 to 4 147)	77.7 (66.0 to 87.0)	44.6 (38.5 to 64.9)
Animal contact	489 (328 to 692)	655 (441 to 935)	13.5 (9.1 to 19.0)	10.4 (7.0 to 14.8)	5 354 (4 445 to 6 536)	1 899 (1 624 to 2 257)	99.0 (84.5 to 117.3)	29.3 (25.2 to 34.9)
Venomous animal contact	286 (185 to 416)	370 (250 to 529)	7.3 (4.8 to 10.4)	5.7 (3.9 to 8.1)	4 543 (3 661 to 5 634)	1 271 (1 070 to 1 509)	80.1 (66.5 to 97.2)	19.4 (16.4 to 22.9)
Non-venomous animal contact	203 (123 to 326)	284 (168 to 467)	6.2 (3.8 to 9.8)	4.6 (2.7 to 7.5)	811 (655 to 1 098)	628 (492 to 850)	18.9 (15.1 to 24.5)	9.9 (7.7 to 13.5)
Foreign body	400 (267 to 579)	391 (235 to 631)	11.6 (7.8 to 16.8)	6.4 (3.8 to 10.3)	11 276 (9 958 to 12 799)	12 794 (10 950 to 15 212)	223.1 (202.6 to 245.6)	187.6 (161.5 to 221.5)
Pulmonary aspiration and foreign body in airway	14 (9 to 19)	8 (5 to 10)	0.3 (0.2 to 0.4)	0.1 (0.1 to 0.2)	10 253 (9 041 to 11 587)	12 151 (10 343 to 14 506)	199.1 (180.6 to 220.4)	177.4 (151.9 to 210.4)
Foreign body in eyes	147 (73 to 265)	267 (141 to 481)	4.9 (2.7 to 8.8)	4.5 (2.4 to 8.0)	147 (73 to 265)	267 (141 to 481)	4.9 (2.7 to 8.8)	4.5 (2.4 to 8.0)
Foreign body in other body part	240 (167 to 320)	117 (83 to 154)	6.3 (4.5 to 8.4)	1.9 (1.3 to 2.4)	875 (679 to 1 020)	377 (310 to 571)	19.0 (15.5 to 21.9)	5.7 (4.7 to 8.6)
Environmental heat and cold exposure	280 (188 to 393)	155 (102 to 221)	8.5 (5.8 to 11.8)	2.5 (1.7 to 3.6)	1 837 (1 655 to 2 038)	754 (666 to 855)	46.5 (42.5 to 51.0)	11.9 (10.5 to 13.5)
Exposure to forces of nature	51 (36 to 70)	341 (235 to 483)	1.3 (0.9 to 1.8)	5.2 (3.6 to 7.3)	174 (134 to 221)	12 264 (11 196 to 13 445)	4.0 (3.2 to 5.0)	184.0 (168.0 to 201.2)
Other unintentional injuries	391 (279 to 517)	504 (358 to 688)	12.0 (8.6 to 16.0)	8.1 (5.7 to 11.0)	3 580 (3 289 to 3 872)	2 809 (2 514 to 3 140)	83.4 (76.8 to 89.7)	42.3 (37.8 to 47.4)

Death, YLL, incidence, prevalence, YLD, and DALY counts and rates per 100,000 for all injuries in Querétaro in 1990 and 2017

Cause	Deaths				Years of life lost			
	All-age counts (95% UI)		Age-standardised rates per 100,000 (95% UI)		All-age counts (95% UI)		Age-standardised rates per 100,000 (95% UI)	
	1990	2017	1990	2017	1990	2017	1990	2017
**Transport and unintentional injuries**	**617 (593 to 641)**	**763 (710 to 819)**	**75.6 (73.5 to 77.9)**	**38.6 (36.1 to 41.3)**	**33 685 (31 902 to 35 591)**	**34 798 (32 316 to 37 595)**	**3 146.6 (3 025.7 to 3 273.1)**	**1 575.3 (1 464.6 to 1 699.7)**
**Transport injuries**	**304 (291 to 318)**	**418 (385 to 452)**	**35.1 (33.8 to 36.6)**	**19.6 (18.1 to 21.1)**	**16 731 (15 866 to 17 683)**	**20 564 (18 894 to 22 284)**	**1 606.1 (1 535.4 to 1 680.2)**	**905.8 (832.5 to 980.8)**
Road injuries	293 (280 to 307)	397 (366 to 430)	33.9 (32.5 to 35.3)	18.6 (17.2 to 20.1)	16 158 (15 322 to 17 072)	19 530 (17 955 to 21 211)	1 551.0 (1 483.5 to 1 624.6)	860.4 (792.2 to 933.3)
Pedestrian road injuries	145 (137 to 154)	174 (160 to 191)	18.3 (17.4 to 19.2)	8.6 (8.0 to 9.4)	7 642 (7 110 to 8 297)	7 797 (7 089 to 8 600)	753.3 (708.1 to 800.7)	352.6 (321.4 to 387.4)
Cyclist road injuries	7 (6 to 7)	15 (13 to 16)	0.8 (0.7 to 0.8)	0.7 (0.6 to 0.8)	366 (327 to 407)	673 (586 to 764)	36.1 (32.3 to 40.0)	30.1 (26.3 to 34.2)
Motorcyclist road injuries	37 (33 to 40)	42 (37 to 48)	3.7 (3.4 to 4.0)	1.8 (1.6 to 2.1)	2 139 (1 919 to 2 365)	2 364 (2 051 to 2 723)	197.7 (179.5 to 217.3)	100.7 (87.3 to 115.8)
Motor vehicle road injuries	92 (85 to 100)	150 (135 to 164)	9.7 (9.1 to 10.4)	6.7 (6.1 to 7.3)	5 306 (4 860 to 5 773)	7 916 (7 092 to 8 708)	497.5 (460.5 to 536.1)	342.6 (307.9 to 376.8)
Other road injuries	13 (10 to 15)	16 (14 to 18)	1.4 (1.1 to 1.6)	0.7 (0.7 to 0.8)	704 (556 to 826)	780 (667 to 908)	66.3 (52.4 to 76.5)	34.4 (29.5 to 39.7)
Other transport injuries	10 (9 to 11)	21 (19 to 24)	1.2 (1.1 to 1.3)	1.0 (0.9 to 1.1)	577 (518 to 640)	1 034 (899 to 1 182)	55.2 (49.8 to 60.7)	45.5 (39.6 to 51.8)
**Unintentional injuries**	**313 (298 to 329)**	**345 (323 to 373)**	**40.5 (39.2 to 41.9)**	**19.0 (17.9 to 20.5)**	**16 951 (15 768 to 18 211)**	**14 234 (13 060 to 15 794)**	**1 540.5 (1 468.0 to 1 620.2)**	**669.4 (614.7 to 742.0)**
Falls	75 (71 to 79)	117 (107 to 127)	14.3 (13.6 to 15.0)	7.4 (6.8 to 8.0)	2 586 (2 480 to 2 752)	3 094 (2 812 to 3 379)	333.9 (316.4 to 351.7)	158.4 (144.2 to 171.9)
Drowning	71 (66 to 77)	53 (48 to 59)	7.1 (6.7 to 7.5)	2.5 (2.3 to 2.8)	4 447 (4 051 to 4 898)	2 832 (2 528 to 3 186)	370.1 (343.3 to 399.9)	126.8 (113.5 to 142.7)
Fire, heat, and hot substances	21 (20 to 23)	19 (17 to 20)	2.9 (2.7 to 3.0)	1.1 (1.0 to 1.2)	1 158 (1 022 to 1 330)	650 (587 to 718)	101.6 (92.9 to 111.5)	31.5 (28.5 to 34.7)
Poisonings	13 (12 to 15)	11 (10 to 12)	1.3 (1.3 to 1.4)	0.5 (0.5 to 0.6)	847 (757 to 945)	541 (486 to 601)	68.4 (62.8 to 74.4)	24.5 (22.0 to 27.2)
Poisoning by carbon monoxide	5 (4 to 5)	5 (4 to 5)	0.5 (0.5 to 0.5)	0.2 (0.2 to 0.2)	296 (262 to 337)	225 (200 to 253)	24.8 (22.4 to 27.5)	10.1 (9.0 to 11.3)
Poisoning by other means	9 (8 to 9)	6 (6 to 7)	0.8 (0.8 to 0.9)	0.3 (0.3 to 0.3)	550 (489 to 620)	316 (283 to 355)	43.6 (39.6 to 48.0)	14.4 (12.9 to 16.1)
Exposure to mechanical forces	35 (33 to 38)	28 (25 to 30)	4.1 (3.9 to 4.4)	1.3 (1.2 to 1.5)	1 932 (1 788 to 2 102)	1 253 (1 124 to 1 389)	186.8 (174.2 to 200.8)	56.0 (50.4 to 61.8)
Unintentional firearm injuries	12 (10 to 14)	7 (5 to 8)	1.2 (1.1 to 1.5)	0.3 (0.3 to 0.4)	664 (586 to 788)	330 (269 to 385)	62.1 (55.2 to 75.0)	14.4 (11.7 to 16.8)
Other exposure to mechanical forces	24 (21 to 26)	21 (19 to 23)	2.9 (2.6 to 3.1)	1.0 (0.9 to 1.1)	1 268 (1 107 to 1 399)	923 (824 to 1 029)	124.7 (110.3 to 135.8)	41.6 (37.4 to 46.1)
Adverse effects of medical treatment	14 (13 to 16)	29 (20 to 30)	2.3 (2.1 to 2.5)	1.4 (1.2 to 1.6)	681 (599 to 779)	746 (638 to 1 121)	65.1 (58.6 to 73.5)	37.3 (32.1 to 55.1)
Animal contact	8 (7 to 9)	5 (5 to 6)	0.8 (0.7 to 0.9)	0.3 (0.3 to 0.3)	506 (429 to 593)	221 (195 to 250)	38.4 (33.8 to 43.6)	10.5 (9.3 to 11.9)
Venomous animal contact	5 (4 to 6)	3 (2 to 3)	0.5 (0.4 to 0.5)	0.1 (0.1 to 0.2)	354 (287 to 426)	116 (96 to 140)	25.0 (21.0 to 29.7)	5.6 (4.6 to 6.7)
Non-venomous animal contact	3 (2 to 3)	3 (2 to 3)	0.3 (0.3 to 0.4)	0.1 (0.1 to 0.2)	152 (120 to 178)	105 (90 to 128)	13.4 (10.5 to 15.2)	5.0 (4.2 to 6.0)
Foreign body	54 (49 to 59)	70 (62 to 79)	5.2 (4.8 to 5.5)	3.5 (3.2 to 4.0)	3 629 (3 213 to 4 078)	3 967 (3 388 to 4 720)	266.8 (242.1 to 293.4)	183.6 (156.9 to 218.4)
Pulmonary aspiration and foreign body in airway	52 (47 to 57)	69 (61 to 78)	5.0 (4.7 to 5.4)	3.5 (3.1 to 3.9)	3 505 (3 101 to 3 953)	3 896 (3 320 to 4 621)	256.9 (232.1 to 283.3)	180.5 (153.7 to 214.1)
Foreign body in eyes								
Foreign body in other body part	2 (1 to 2)	1 (1 to 2)	0.2 (0.1 to 0.2)	0.1 (0.0 to 0.1)	124 (96 to 147)	71 (57 to 135)	9.9 (7.7 to 11.4)	3.1 (2.5 to 6.0)
Environmental heat and cold exposure	5 (5 to 5)	4 (3 to 4)	0.8 (0.7 to 0.8)	0.2 (0.2 to 0.2)	234 (213 to 256)	133 (118 to 148)	24.9 (22.9 to 26.8)	6.3 (5.5 to 6.9)
Exposure to forces of nature	1 (1 to 1)	0 (0 to 0)	0.1 (0.1 to 0.1)	0.0 (0.0 to 0.0)	39 (36 to 44)	18 (17 to 20)	3.4 (3.1 to 3.7)	0.8 (0.7 to 0.9)
Other unintentional injuries	15 (14 to 17)	15 (14 to 17)	1.7 (1.5 to 1.8)	0.7 (0.6 to 0.8)	891 (815 to 978)	779 (685 to 884)	81.2 (74.7 to 88.4)	33.9 (29.9 to 38.4)

Cause	Incidence				Prevalence			
	All-age counts (95% UI)		Age-standardised rates per 100,000 (95% UI)		All-age counts (95% UI)		Age-standardised rates per 100,000 (95% UI)	
	1990	2017	1990	2017	1990	2017	1990	2017
**Transport and unintentional injuries**	**41 296 (37 945 to 45 101)**	**87 322 (79 335 to 96 284)**	**4 473.8 (4 092.2 to 4 896.6)**	**4 296.3 (3 913.4 to 4 709.8)**	**74 042 (69 350 to 79 317)**	**178 673 (167 182 to 192 816)**	**10 065.1 (9 475.9 to 10 842.2)**	**8 952.1 (8 393.2 to 9 657.3)**
**Transport injuries**	**3 868 (3 328 to 4 492)**	**11 003 (9 573 to 12 641)**	**404.4 (354.2 to 457.9)**	**499.6 (437.9 to 570.2)**	**8 806 (8 214 to 9 582)**	**29 715 (27 503 to 32 089)**	**1 246.3 (1 167.2 to 1 335.2)**	**1 450.0 (1 343.5 to 1 565.5)**
Road injuries	3 651 (3 135 to 4 275)	10 772 (9 342 to 12 415)	383.8 (334.6 to 436.6)	488.4 (426.5 to 558.3)	7 906 (7 353 to 8 462)	28 564 (26 460 to 30 917)	1 133.9 (1 054.8 to 1 216.6)	1 393.0 (1 291.1 to 1 507.4)
Pedestrian road injuries	602 (484 to 742)	1 180 (948 to 1 449)	67.1 (55.8 to 80.5)	54.9 (44.3 to 66.6)	2 089 (1 787 to 2 450)	5 279 (4 570 to 6 155)	305.5 (265.6 to 355.0)	262.7 (228.9 to 305.7)
Cyclist road injuries	491 (368 to 645)	1 157 (908 to 1 464)	47.3 (37.4 to 60.4)	53.7 (42.5 to 67.1)	769 (646 to 908)	2 497 (2 118 to 2 937)	103.0 (87.3 to 119.1)	121.3 (103.3 to 140.8)
Motorcyclist road injuries	460 (358 to 584)	868 (650 to 1 123)	45.3 (36.0 to 55.7)	38.7 (29.3 to 49.8)	1 537 (1 335 to 1 788)	3 879 (3 323 to 4 572)	213.6 (184.4 to 247.5)	184.9 (159.0 to 217.3)
Motor vehicle road injuries	1 369 (1 120 to 1 656)	5 819 (4 879 to 6 869)	147.6 (122.5 to 179.3)	259.8 (218.2 to 304.9)	2 267 (1 979 to 2 645)	12 830 (11 188 to 15 120)	337.1 (293.1 to 391.6)	624.0 (544.4 to 731.7)
Other road injuries	729 (562 to 912)	1 748 (1 396 to 2 126)	76.5 (61.4 to 91.9)	81.3 (65.3 to 98.2)	1 244 (1 040 to 1 499)	4 081 (3 393 to 4 942)	174.7 (146.8 to 208.7)	200.1 (167.0 to 241.3)
Other transport injuries	217 (163 to 295)	232 (176 to 298)	20.6 (16.7 to 25.8)	11.2 (8.5 to 14.4)	900 (742 to 1 072)	1 151 (979 to 1 331)	114.4 (96.7 to 132.2)	57.0 (49.1 to 65.4)
**Unintentional injuries**	**37 428 (34 158 to 41 104)**	**76 319 (68 733 to 85 241)**	**4 069.4 (3 687.4 to 4 498.9)**	**3 796.7 (3 418.7 to 4 201.4)**	**65 235 (60 573 to 70 385)**	**148 957 (138 175 to 163 248)**	**8 816.8 (8 230.3 to 9 573.2)**	**7 502.1 (6 958.7 to 8 197.7)**
Falls	17 259 (15 064 to 19 720)	43 459 (38 829 to 48 881)	2 066.6 (1 849.4 to 2 314.3)	2 257.8 (2 016.2 to 2 529.2)	28 332 (24 974 to 32 347)	83 303 (74 092 to 94 929)	4 121.3 (3 677.5 to 4 700.2)	4 348.9 (3 874.8 to 4 977.7)
Drowning	62 (54 to 73)	49 (39 to 62)	6.0 (5.2 to 6.8)	2.3 (1.9 to 2.9)	262 (229 to 301)	289 (253 to 337)	37.4 (33.1 to 42.5)	14.6 (12.8 to 16.8)
Fire, heat, and hot substances	1 426 (1 130 to 1 821)	1 692 (1 365 to 2 092)	108.5 (88.8 to 133.2)	78.2 (63.3 to 95.9)	12 472 (10 304 to 15 747)	19 527 (16 054 to 25 133)	1 446.6 (1 214.4 to 1 770.9)	917.1 (756.9 to 1 167.3)
Poisonings	491 (357 to 683)	506 (350 to 711)	33.3 (25.0 to 44.7)	23.4 (16.2 to 32.8)	384 (288 to 482)	589 (427 to 735)	38.4 (29.3 to 47.8)	26.8 (19.4 to 33.4)
Poisoning by carbon monoxide	98 (61 to 152)	108 (56 to 182)	7.5 (5.1 to 10.9)	5.0 (2.7 to 8.3)	61 (47 to 80)	87 (66 to 113)	6.4 (5.0 to 8.2)	4.0 (3.0 to 5.2)
Poisoning by other means	393 (277 to 564)	398 (267 to 587)	25.8 (18.6 to 36.2)	18.3 (12.3 to 27.1)	323 (231 to 415)	502 (348 to 648)	32.0 (23.2 to 41.0)	22.8 (15.8 to 29.4)
Exposure to mechanical forces	8 181 (6 963 to 9 655)	13 544 (11 604 to 15 842)	778.8 (673.4 to 908.4)	608.2 (525.4 to 707.2)	14 728 (12 826 to 17 010)	31 624 (27 636 to 36 789)	2 018.9 (1 767.1 to 2 323.7)	1 523.5 (1 331.3 to 1 763.9)
Unintentional firearm injuries	464 (336 to 616)	587 (420 to 788)	42.3 (31.6 to 54.9)	26.2 (19.0 to 34.8)	1 648 (1 305 to 1 961)	2 824 (2 291 to 3 393)	228.3 (184.4 to 273.1)	137.5 (112.3 to 164.6)
Other exposure to mechanical forces	7 717 (6 570 to 9 140)	12 957 (11 153 to 15 168)	736.5 (635.2 to 863.4)	582.0 (502.9 to 677.1)	13 080 (11 077 to 15 457)	28 800 (24 619 to 34 010)	1 790.6 (1 532.9 to 2 095.6)	1 386.0 (1 189.3 to 1 622.5)
Adverse effects of medical treatment	882 (749 to 1 024)	2 571 (2 154 to 2 966)	104.3 (89.6 to 118.2)	124.2 (104.5 to 142.2)	68 (52 to 84)	197 (152 to 243)	8.0 (6.1 to 9.8)	9.5 (7.3 to 11.8)
Animal contact	2 684 (2 377 to 3 316)	3 209 (2 699 to 3 895)	258.1 (218.2 to 304.1)	154.0 (130.3 to 184.9)	2 621 (2 238 to 3 064)	3 875 (3 332 to 4 450)	339.2 (295.8 to 387.8)	191.0 (165.2 to 218.8)
Venomous animal contact	908 (675 to 1 210)	993 (756 to 1 293)	87.0 (66.7 to 110.9)	47.5 (36.4 to 61.4)	546 (447 to 650)	671 (561 to 793)	64.7 (54.4 to 75.4)	32.0 (27.0 to 37.5)
Non-venomous animal contact	1 776 (1 400 to 2 242)	2 216 (1 820 to 2 740)	171.1 (139.8 to 207.0)	106.5 (88.1 to 130.6)	2 075 (1 715 to 2 498)	3 204 (2 685 to 3 785)	274.5 (231.9 to 321.6)	159.0 (134.3 to 186.5)
Foreign body	3 880 (2 221 to 6 104)	8 297 (3 418 to 14 813)	464.1 (217.3 to 771.9)	405.7 (164.2 to 694.2)	1 745 (1 331 to 2 341)	2 747 (1 927 to 4 031)	218.8 (170.3 to 291.2)	139.7 (99.4 to 198.6)
Pulmonary aspiration and foreign body in airway	143 (100 to 197)	89 (64 to 121)	10.0 (7.5 to 13.2)	4.3 (3.1 to 5.7)	88 (63 to 122)	77 (62 to 96)	9.2 (6.8 to 12.2)	3.7 (3.0 to 4.5)
Foreign body in eyes	3 389 (1 717 to 5 645)	7 930 (3 048 to 14 459)	426.6 (176.7 to 736.3)	388.1 (146.0 to 677.1)	659 (327 to 1 177)	1 671 (884 to 2 890)	97.2 (54.5 to 160.6)	87.8 (48.8 to 145.3)
Foreign body in other body part	347 (231 to 540)	278 (186 to 390)	27.4 (18.9 to 39.5)	13.3 (8.9 to 18.4)	997 (786 to 1 286)	999 (834 to 1 220)	112.4 (90.5 to 140.3)	48.2 (40.6 to 58.7)
Environmental heat and cold exposure	498 (343 to 687)	358 (248 to 505)	48.0 (36.4 to 60.1)	18.0 (12.7 to 25.1)	1 638 (1 314 to 2 019)	1 407 (1 173 to 1 722)	203.8 (170.1 to 248.7)	70.2 (58.9 to 85.0)
Exposure to forces of nature	103 (84 to 129)	24 (20 to 29)	8.7 (7.4 to 10.4)	1.1 (0.9 to 1.3)	369 (264 to 503)	1 019 (709 to 1 430)	36.9 (27.0 to 50.2)	46.7 (32.7 to 64.9)
Other unintentional injuries	1 961 (1 614 to 2 417)	2 611 (2 208 to 3 078)	193.0 (163.2 to 226.6)	123.8 (104.7 to 145.6)	2 617 (2 237 to 3 030)	4 383 (3 826 to 4 990)	347.5 (301.5 to 397.5)	214.3 (187.8 to 242.7)

Cause	Years lived with disability				Disability adjusted life years			
	All-age counts (95% UI)		Age-standardised rates per 100,000 (95% UI)		All-age counts (95% UI)		Age-standardised rates per 100,000 (95% UI)	
	1990	2017	1990	2017	1990	2017	1990	2017
**Transport and unintentional injuries**	**3 366 (2 472 to 4 463)**	**7 759 (5 578 to 10 464)**	**450.0 (330.1 to 595.1)**	**389.1 (279.5 to 521.6)**	**37 052 (35 087 to 39 191)**	**42 558 (39 093 to 46 235)**	**3 596.7 (3 422.5 to 3 786.8)**	**1 964.4 (1 803.2 to 2 140.8)**
**Transport injuries**	**607 (441 to 803)**	**1 823 (1 316 to 2 435)**	**82.2 (60.4 to 108.4)**	**87.4 (63.4 to 116.9)**	**17 342 (16 457 to 18 339)**	**22 387 (20 653 to 24 195)**	**1 688.3 (1 611.7 to 1 768.2)**	**993.2 (916.4 to 1 072.4)**
Road injuries	549 (401 to 727)	1 761 (1 274 to 2 352)	75.2 (55.0 to 99.4)	84.4 (61.1 to 112.8)	16 706 (15 851 to 17 651)	21 290 (19 628 to 23 006)	1 626.2 (1 553.4 to 1 702.6)	944.7 (873.4 to 1 021.2)
Pedestrian road injuries	151 (107 to 205)	339 (240 to 464)	21.0 (15.0 to 28.4)	16.6 (11.7 to 22.7)	7 793 (7 259 to 8 394)	8 135 (7 419 to 8 954)	774.3 (729.8 to 821.2)	369.2 (337.4 to 405.2)
Cyclist road injuries	54 (38 to 72)	152 (106 to 202)	7.0 (4.9 to 9.2)	7.3 (5.1 to 9.7)	420 (377 to 465)	825 (733 to 935)	43.1 (38.7 to 47.6)	37.4 (33.1 to 42.5)
Motorcyclist road injuries	108 (76 to 146)	241 (164 to 328)	14.4 (10.1 to 19.5)	11.3 (7.7 to 15.4)	2 247 (2 023 to 2 475)	2 604 (2 267 to 2 950)	212.1 (192.5 to 232.7)	112.0 (97.5 to 126.6)
Motor vehicle road injuries	163 (119 to 215)	821 (590 to 1 093)	23.1 (16.8 to 30.4)	39.1 (28.2 to 52.1)	5 470 (5 002 to 5 933)	8 736 (7 901 to 9 590)	520.7 (480.6 to 559.9)	381.7 (345.1 to 418.9)
Other road injuries	72 (51 to 97)	209 (148 to 288)	9.6 (7.0 to 12.9)	10.1 (7.1 to 13.8)	776 (625 to 899)	989 (851 to 1 138)	76.0 (61.6 to 86.4)	44.4 (38.4 to 50.8)
Other transport injuries	58 (41 to 78)	62 (44 to 83)	7.0 (5.0 to 9.2)	3.0 (2.2 to 4.1)	635 (576 to 701)	1 096 (955 to 1 238)	62.1 (56.6 to 68.1)	48.5 (42.4 to 54.6)
**Unintentional injuries**	**2 759 (2 011 to 3 666)**	**5 936 (4 214 to 8 060)**	**367.8 (266.6 to 488.6)**	**301.7 (213.7 to 406.4)**	**19 710 (18 259 to 21 245)**	**20 171 (18 107 to 22 678)**	**1 908.3 (1 776.9 to 2 060.1)**	**971.2 (869.7 to 1 094.3)**
Falls	1 700 (1 248 to 2 240)	4 271 (3 063 to 5 674)	238.5 (174.6 to 311.8)	222.0 (158.7 to 296.0)	4 286 (3 795 to 4 833)	7 366 (6 122 to 8 780)	572.3 (504.7 to 646.9)	380.3 (316.7 to 454.0)
Drowning	15 (11 to 21)	15 (10 to 22)	2.1 (1.4 to 2.8)	0.8 (0.5 to 1.1)	4 462 (4 067 to 4 913)	2 847 (2 546 to 3 205)	372.1 (345.3 to 401.6)	127.5 (114.3 to 143.6)
Fire, heat, and hot substances	271 (168 to 427)	379 (228 to 618)	30.8 (18.6 to 47.4)	17.7 (10.4 to 28.8)	1 429 (1 250 to 1 654)	1 030 (856 to 1 295)	131.8 (116.6 to 151.0)	49.2 (41.1 to 61.8)
Poisonings	45 (28 to 69)	68 (40 to 105)	4.4 (2.7 to 6.5)	3.1 (1.8 to 4.7)	892 (804 to 999)	609 (545 to 679)	72.7 (67.1 to 79.4)	27.5 (24.7 to 30.6)
Poisoning by carbon monoxide	7 (4 to 10)	9 (5 to 14)	0.6 (0.4 to 1.0)	0.4 (0.2 to 0.6)	303 (269 to 343)	233 (207 to 263)	25.4 (23.0 to 28.2)	10.5 (9.4 to 11.8)
Poisoning by other means	39 (23 to 59)	60 (34 to 93)	3.7 (2.3 to 5.6)	2.7 (1.5 to 4.2)	589 (530 to 662)	376 (333 to 424)	47.3 (43.2 to 52.1)	17.1 (15.1 to 19.3)
Exposure to mechanical forces	351 (249 to 499)	689 (460 to 1 002)	46.9 (32.2 to 67.2)	33.0 (21.9 to 48.0)	2 283 (2 103 to 2 484)	1 942 (1 675 to 2 289)	233.6 (214.8 to 256.9)	89.0 (76.5 to 105.5)
Unintentional firearm injuries	34 (22 to 51)	52 (33 to 82)	4.5 (2.9 to 6.9)	2.5 (1.6 to 3.9)	698 (619 to 830)	382 (316 to 448)	66.6 (59.5 to 79.7)	16.9 (14.0 to 19.7)
Other exposure to mechanical forces	317 (229 to 450)	637 (426 to 929)	42.4 (29.3 to 60.8)	30.5 (20.4 to 44.3)	1 586 (1 410 to 1 763)	1 560 (1 324 to 1 864)	167.1 (149.0 to 188.3)	72.1 (61.2 to 87.1)
Adverse effects of medical treatment	9 (6 to 14)	26 (16 to 40)	1.1 (0.7 to 1.6)	1.3 (0.8 to 1.9)	690 (606 to 790)	772 (663 to 1 150)	66.1 (59.7 to 74.8)	38.6 (33.4 to 56.5)
Animal contact	70 (47 to 100)	90 (60 to 129)	8.0 (5.4 to 11.4)	4.3 (2.9 to 6.1)	576 (494 to 669)	311 (269 to 361)	46.5 (40.9 to 52.8)	14.8 (12.8 to 17.2)
Venomous animal contact	38 (24 to 55)	44 (28 to 66)	3.8 (2.5 to 5.5)	2.0 (1.3 to 3.0)	392 (324 to 469)	160 (134 to 192)	28.9 (24.6 to 33.9)	7.6 (6.4 to 9.1)
Non-venomous animal contact	32 (20 to 52)	46 (27 to 73)	4.2 (2.6 to 6.6)	2.3 (1.4 to 3.7)	185 (147 to 217)	151 (124 to 186)	17.6 (13.9 to 20.6)	7.2 (6.0 to 8.9)
Foreign body	111 (79 to 164)	169 (97 to 267)	13.6 (8.9 to 20.1)	8.3 (5.0 to 13.6)	3 740 (3 318 to 4 187)	4 129 (3 527 to 4 899)	280.4 (255.4 to 306.7)	191.8 (163.8 to 227.7)
Pulmonary aspiration and foreign body in airway	5 (3 to 7)	3 (2 to 5)	0.5 (0.3 to 0.6)	0.2 (0.1 to 0.2)	3 510 (3 105 to 3 958)	3 899 (3 323 to 4 624)	257.4 (232.6 to 283.8)	180.6 (153.8 to 214.2)
Foreign body in eyes	44 (22 to 82)	112 (57 to 206)	6.5 (3.5 to 11.6)	5.8 (3.1 to 10.6)	44 (22 to 82)	112 (57 to 206)	6.5 (3.5 to 11.6)	5.8 (3.1 to 10.6)
Foreign body in other body part	62 (43 to 84)	48 (34 to 63)	6.6 (4.6 to 8.8)	2.3 (1.6 to 3.0)	186 (155 to 219)	119 (97 to 188)	16.5 (13.8 to 19.3)	5.4 (4.4 to 8.2)
Environmental heat and cold exposure	65 (43 to 90)	53 (34 to 77)	8.0 (5.4 to 11.2)	2.7 (1.7 to 3.9)	299 (267 to 334)	187 (160 to 215)	32.9 (29.5 to 36.9)	8.9 (7.7 to 10.3)
Exposure to forces of nature	18 (12 to 25)	33 (23 to 46)	1.8 (1.3 to 2.5)	1.5 (1.1 to 2.1)	57 (50 to 66)	51 (41 to 64)	5.2 (4.6 to 5.9)	2.3 (1.9 to 2.9)
Other unintentional injuries	104 (75 to 138)	149 (105 to 204)	13.4 (9.6 to 17.9)	7.2 (5.1 to 9.9)	995 (914 to 1 091)	927 (821 to 1 047)	94.6 (87.3 to 102.7)	41.1 (36.5 to 46.3)

Death, YLL, incidence, prevalence, YLD, and DALY counts and rates per 100,000 for all injuries in Quintana Roo in 1990 and 2017

Cause	Deaths				Years of life lost			
	All-age counts (95% UI)		Age-standardised rates per 100,000 (95% UI)		All-age counts (95% UI)		Age-standardised rates per 100,000 (95% UI)	
	1990	2017	1990	2017	1990	2017	1990	2017
**Transport and unintentional injuries**	**286 (268 to 308)**	**587 (545 to 628)**	**73.7 (70.8 to 76.9)**	**44.1 (41.1 to 47.0)**	**17 619 (16 042 to 19 378)**	**27 971 (25 836 to 30 128)**	**3 336.7 (3 122.8 to 3 586.5)**	**1 675.4 (1 553.1 to 1 800.9)**
**Transport injuries**	**132 (124 to 140)**	**295 (268 to 321)**	**31.6 (30.1 to 33.2)**	**19.1 (17.4 to 20.7)**	**7 872 (7 321 to 8 469)**	**14 803 (13 459 to 16 139)**	**1 512.4 (1 426.1 to 1 606.5)**	**835.4 (761.4 to 908.0)**
Road injuries	126 (119 to 134)	273 (248 to 297)	30.1 (28.8 to 31.7)	17.6 (16.0 to 19.1)	7 528 (6 997 to 8 127)	13 690 (12 428 to 14 963)	1 445.5 (1 363.9 to 1 538.3)	772.8 (703.5 to 841.4)
Pedestrian road injuries	64 (59 to 69)	123 (111 to 136)	17.2 (16.2 to 18.3)	8.9 (8.1 to 9.7)	3 779 (3 393 to 4 210)	5 740 (5 149 to 6 397)	756.5 (701.9 to 821.3)	342.2 (309.6 to 376.9)
Cyclist road injuries	3 (3 to 3)	11 (9 to 12)	0.7 (0.6 to 0.7)	0.7 (0.6 to 0.8)	165 (146 to 187)	516 (453 to 587)	32.4 (29.1 to 36.2)	30.1 (26.4 to 34.1)
Motorcyclist road injuries	19 (17 to 21)	41 (36 to 47)	3.8 (3.4 to 4.1)	2.2 (1.9 to 2.5)	1 156 (1 036 to 1 293)	2 297 (2 000 to 2 632)	209.4 (189.4 to 231.8)	120.0 (105.0 to 137.3)
Motor vehicle road injuries	38 (34 to 41)	92 (80 to 103)	7.9 (7.3 to 8.5)	5.4 (4.7 to 6.0)	2 281 (2 064 to 2 492)	4 859 (4 264 to 5 449)	419.7 (383.1 to 452.9)	264.4 (231.9 to 295.9)
Other road injuries	2 (2 to 3)	6 (5 to 6)	0.6 (0.5 to 0.6)	0.4 (0.3 to 0.4)	146 (118 to 171)	284 (246 to 329)	27.5 (22.5 to 31.5)	16.0 (14.0 to 18.3)
Other transport injuries	6 (5 to 6)	23 (19 to 26)	1.4 (1.3 to 1.6)	1.4 (1.2 to 1.6)	344 (303 to 388)	1 113 (969 to 1 277)	66.9 (59.9 to 74.3)	62.6 (54.3 to 71.3)
**Unintentional injuries**	**154 (141 to 169)**	**291 (271 to 312)**	**42.1 (40.0 to 44.4)**	**25.0 (23.5 to 26.6)**	**9 746 (8 591 to 11 007)**	**13 168 (12 120 to 14 298)**	**1 824.2 (1 666.8 to 1 999.3)**	**840.0 (777.6 to 909.6)**
Falls	23 (21 to 24)	74 (68 to 81)	11.6 (11.0 to 12.3)	8.8 (8.0 to 9.5)	1 013 (919 to 1 111)	2 319 (2 106 to 2 551)	286.4 (269.2 to 304.2)	177.8 (162.2 to 193.8)
Drowning	41 (37 to 47)	56 (51 to 62)	8.7 (8.0 to 9.5)	3.7 (3.3 to 4.0)	2 719 (2 367 to 3 158)	2 980 (2 664 to 3 301)	474.1 (422.1 to 537.0)	174.1 (156.3 to 191.8)
Fire, heat, and hot substances	9 (8 to 10)	13 (12 to 14)	2.5 (2.3 to 2.7)	1.3 (1.2 to 1.4)	586 (482 to 717)	517 (468 to 566)	106.1 (91.8 to 124.3)	35.1 (32.0 to 38.2)
Poisonings	6 (5 to 7)	9 (8 to 10)	1.3 (1.2 to 1.4)	0.6 (0.6 to 0.7)	421 (361 to 497)	455 (410 to 504)	71.6 (62.7 to 81.8)	26.9 (24.4 to 29.7)
Poisoning by carbon monoxide	2 (2 to 2)	3 (3 to 4)	0.4 (0.4 to 0.5)	0.2 (0.2 to 0.3)	124 (105 to 145)	175 (152 to 198)	21.9 (19.1 to 25.3)	10.1 (8.8 to 11.4)
Poisoning by other means	4 (4 to 5)	6 (5 to 6)	0.9 (0.8 to 1.0)	0.4 (0.3 to 0.4)	298 (252 to 356)	280 (252 to 311)	49.7 (43.2 to 57.7)	16.8 (15.1 to 18.6)
Exposure to mechanical forces	17 (15 to 18)	26 (23 to 29)	4.2 (3.9 to 4.4)	1.8 (1.6 to 1.9)	978 (890 to 1 069)	1 234 (1 105 to 1 372)	193.6 (178.9 to 209.3)	71.0 (64.1 to 78.2)
Unintentional firearm injuries	7 (6 to 8)	8 (6 to 9)	1.5 (1.3 to 1.9)	0.5 (0.4 to 0.5)	395 (346 to 497)	391 (325 to 461)	74.9 (66.5 to 85.6)	21.3 (17.9 to 25.2)
Other exposure to mechanical forces	10 (8 to 11)	18 (16 to 20)	2.7 (2.2 to 2.9)	1.3 (1.2 to 1.4)	583 (459 to 660)	843 (737 to 949)	118.7 (94.7 to 131.5)	49.7 (43.8 to 55.5)
Adverse effects of medical treatment	7 (5 to 9)	23 (19 to 25)	2.6 (1.9 to 2.9)	2.4 (2.0 to 2.7)	452 (303 to 545)	849 (724 to 972)	90.4 (64.2 to 104.6)	62.6 (53.9 to 70.8)
Animal contact	5 (4 to 6)	4 (4 to 5)	1.0 (0.9 to 1.2)	0.4 (0.3 to 0.4)	341 (268 to 434)	192 (171 to 219)	55.3 (45.1 to 68.0)	12.8 (11.4 to 14.5)
Venomous animal contact	4 (3 to 5)	3 (3 to 3)	0.8 (0.7 to 1.0)	0.3 (0.2 to 0.3)	299 (231 to 383)	136 (115 to 160)	47.2 (37.6 to 59.0)	9.2 (7.8 to 10.7)
Non venomous animal contact	1 (1 to 1)	1 (1 to 2)	0.2 (0.2 to 0.2)	0.1 (0.1 to 0.1)	43 (34 to 54)	56 (47 to 72)	8.1 (6.6 to 9.8)	3.6 (3.1 to 4.6)
Foreign body	33 (28 to 38)	60 (54 to 68)	6.7 (6.0 to 7.4)	4.5 (4.1 to 4.9)	2 409 (1 986 to 2 886)	3 370 (2 931 to 3 924)	383.4 (326.0 to 449.3)	209.2 (181.8 to 242.8)
Pulmonary aspiration and foreign body in airway	32 (27 to 37)	59 (53 to 66)	6.5 (5.8 to 7.2)	4.4 (4.0 to 4.9)	2 339 (1 924 to 2 810)	3 293 (2 866 to 3 847)	371.8 (316.0 to 436.1)	204.9 (178.1 to 238.2)
Foreign body in eyes								
Foreign body in other body part	1 (1 to 1)	1 (1 to 2)	0.2 (0.2 to 0.2)	0.1 (0.1 to 0.1)	70 (56 to 86)	76 (62 to 130)	11.6 (9.6 to 13.9)	4.2 (3.4 to 7.3)
Environmental heat and cold exposure	3 (3 to 4)	6 (5 to 6)	1.2 (1.1 to 1.3)	0.4 (0.4 to 0.5)	173 (152 to 198)	226 (197 to 256)	40.9 (37.4 to 45.0)	14.4 (12.6 to 16.1)
Exposure to forces of nature	1 (0 to 1)	0 (0 to 0)	0.1 (0.1 to 0.2)	0.0 (0.0 to 0.0)	30 (27 to 33)	14 (13 to 15)	6.0 (5.5 to 6.5)	0.8 (0.8 to 0.9)
Other unintentional injuries	10 (9 to 11)	19 (17 to 22)	2.3 (2.1 to 2.5)	1.2 (1.0 to 1.3)	625 (562 to 690)	1 018 (889 to 1 154)	116.5 (106.4 to 127.1)	55.3 (48.8 to 62.5)

Cause	Incidence				Prevalence			
	All-age counts (95% UI)		Age-standardised rates per 100,000 (95% UI)		All-age counts (95% UI)		Age-standardised rates per 100,000 (95% UI)	
	1990	2017	1990	2017	1990	2017	1990	2017
**Transport and unintentional injuries**	**18 462 (17 122 to 19 873)**	**66 937 (62 340 to 72 215)**	**4 408.6 (4 136.3 to 4 686.5)**	**4 476.5 (4 167.5 to 4 823.8)**	**34 505 (32 190 to 36 934)**	**147 049 (137 435 to 158 968)**	**10 800.2 (10 175.0 to 11 585.9)**	**10 278.8 (9 639.9 to 11 101.9)**
**Transport injuries**	**1 928 (1 645 to 2 269)**	**10 124 (8 726 to 11 752)**	**443.0 (386.2 to 501.5)**	**598.8 (523.2 to 684.4)**	**4 149 (3 863 to 4 422)**	**25 809 (24 083 to 27 599)**	**1 389.3 (1 299.3 to 1 480.4)**	**1 760.6 (1 644.5 to 1 886.6)**
Road injuries	1 823 (1 539 to 2 163)	9 918 (8 517 to 11 572)	420.2 (362.7 to 478.7)	584.8 (508.5 to 669.2)	3 717 (3 458 to 3 970)	24 857 (23 121 to 26 622)	1 261.4 (1 176.7 to 1 351.4)	1 691.9 (1 578.8 to 1 817.4)
Pedestrian road injuries	305 (244 to 375)	1 411 (1 132 to 1 731)	77.9 (65.5 to 92.7)	86.0 (70.1 to 103.2)	973 (834 to 1 138)	5 463 (4 719 to 6 438)	345.7 (302.1 to 399.2)	385.6 (335.4 to 448.7)
Cyclist road injuries	333 (259 to 434)	1 695 (1 351 to 2 107)	71.6 (57.1 to 88.1)	104.1 (84.0 to 126.8)	508 (420 to 607)	3 280 (2 731 to 3 819)	155.7 (131.5 to 180.8)	218.6 (186.6 to 253.0)
Motorcyclist road injuries	292 (230 to 362)	1 602 (1 254 to 2 037)	62.0 (50.0 to 75.3)	90.5 (72.1 to 113.3)	880 (760 to 1 016)	6 219 (5 333 to 7 239)	286.2 (248.1 to 328.3)	404.2 (347.8 to 469.7)
Motor vehicle road injuries	611 (494 to 750)	4 119 (3 372 to 4 948)	143.4 (117.6 to 170.1)	235.4 (194.5 to 278.1)	898 (783 to 1 051)	7 695 (6 691 to 9 065)	322.4 (279.6 to 374.4)	521.5 (453.1 to 611.6)
Other road injuries	276 (198 to 363)	1 090 (767 to 1 481)	65.3 (49.2 to 81.4)	68.7 (50.3 to 89.7)	458 (378 to 557)	2 309 (1 920 to 2 796)	151.4 (126.1 to 180.2)	161.9 (135.2 to 195.0)
Other transport injuries	105 (82 to 139)	207 (165 to 260)	22.8 (18.8 to 28.0)	14.0 (11.4 to 17.2)	432 (354 to 520)	952 (806 to 1 109)	127.9 (108.5 to 147.9)	68.8 (59.4 to 78.7)
**Unintentional injuries**	**16 533 (15 280 to 17 870)**	**56 812 (52 278 to 61 739)**	**3 965.6 (3 701.2 to 4 251.4)**	**3 877.8 (3 587.6 to 4 216.1)**	**30 356 (28 173 to 32 820)**	**121 240 (112 055 to 133 217)**	**9 410.9 (8 782.5 to 10 163.6)**	**8 518.2 (7 896.1 to 9 307.6)**
Falls	7 053 (6 090 to 8 066)	29 996 (26 352 to 34 363)	1 965.4 (1 755.6 to 2 199.2)	2 219.0 (1 962.8 to 2 530.2)	11 592 (10 036 to 13 466)	59 300 (51 535 to 67 789)	4 007.0 (3 582.1 to 4 586.8)	4 440.6 (3 951.4 to 5 077.7)
Drowning	34 (29 to 39)	42 (34 to 53)	7.1 (6.2 to 8.1)	2.8 (2.3 to 3.4)	136 (118 to 158)	227 (200 to 263)	45.5 (40.2 to 52.0)	17.1 (15.1 to 19.6)
Fire, heat, and hot substances	773 (624 to 968)	1 888 (1 568 to 2 250)	130.3 (107.0 to 158.1)	116.2 (96.7 to 138.7)	6 729 (5 521 to 8 578)	21 424 (17 734 to 27 082)	1 732.2 (1 436.1 to 2 105.8)	1 359.5 (1 142.1 to 1 689.7)
Poisonings	259 (193 to 340)	633 (472 to 831)	40.2 (30.9 to 51.6)	39.0 (29.2 to 51.6)	213 (164 to 261)	734 (558 to 908)	46.5 (36.5 to 56.8)	43.8 (33.4 to 54.1)
Poisoning by carbon monoxide	52 (34 to 80)	118 (63 to 195)	9.0 (6.3 to 12.9)	7.4 (4.2 to 12.0)	34 (26 to 45)	94 (72 to 122)	7.9 (6.1 to 10.1)	5.7 (4.5 to 7.4)
Poisoning by other means	207 (153 to 274)	514 (375 to 696)	31.2 (23.4 to 41.2)	31.6 (23.0 to 42.9)	178 (130 to 225)	640 (458 to 811)	38.6 (28.6 to 48.6)	38.1 (27.3 to 48.1)
Exposure to mechanical forces	4 106 (3 483 to 4 935)	11 625 (10 018 to 13 523)	841.8 (727.2 to 987.3)	676.2 (588.0 to 781.7)	7 065 (6 098 to 8 184)	26 168 (22 448 to 30 667)	2 265.5 (1 987.1 to 2 598.1)	1 740.1 (1 531.2 to 2 016.7)
Unintentional firearm injuries	248 (178 to 344)	386 (269 to 533)	48.5 (35.8 to 64.5)	22.4 (16.0 to 29.7)	803 (628 to 969)	1 789 (1 433 to 2 171)	264.3 (211.8 to 318.7)	122.7 (99.9 to 147.8)
Other exposure to mechanical forces	3 858 (3 263 to 4 611)	11 239 (9 654 to 13 110)	793.3 (682.4 to 927.9)	653.8 (568.0 to 755.6)	6 262 (5 258 to 7 428)	24 375 (20 570 to 28 921)	2 001.2 (1 714.0 to 2 351.1)	1 617.4 (1 382.2 to 1 902.3)
Adverse effects of medical treatment	390 (324 to 459)	1 904 (1 607 to 2 206)	106.8 (91.6 to 123.1)	128.6 (109.1 to 146.5)	30 (23 to 38)	146 (111 to 181)	8.2 (6.3 to 10.1)	9.8 (7.5 to 12.1)
Animal contact	1 537 (1 269 to 1 829)	4 560 (3 939 to 5 300)	326.6 (285.2 to 376.7)	293.1 (255.5 to 337.4)	1 460 (1 246 to 1 712)	5 378 (4 699 to 6 182)	434.1 (380.3 to 494.4)	369.0 (326.2 to 420.0)
Venomous animal contact	577 (445 to 740)	1 744 (1 380 to 2 147)	123.5 (98.5 to 152.9)	111.1 (88.8 to 136.5)	371 (302 to 447)	1 288 (1 063 to 1 535)	100.1 (83.5 to 117.1)	84.8 (71.1 to 98.5)
Non venomous animal contact	940 (763 to 1 158)	2 816 (2 338 to 3 404)	203.1 (170.2 to 243.7)	182.1 (153.2 to 218.3)	1 089 (878 to 1 334)	4 089 (3 448 to 4 855)	334.0 (280.5 to 391.7)	284.2 (242.3 to 333.3)
Foreign body	1 055 (712 to 1 496)	3 523 (2 339 to 4 853)	251.6 (170.7 to 335.2)	231.8 (159.9 to 331.0)	576 (460 to 731)	1 649 (1 310 to 2 064)	158.5 (131.9 to 191.8)	114.8 (92.8 to 141.5)
Pulmonary aspiration and foreign body in airway	81 (57 to 114)	76 (56 to 102)	13.2 (9.7 to 17.9)	5.2 (3.8 to 6.7)	53 (36 to 74)	62 (50 to 77)	11.9 (8.5 to 16.1)	4.2 (3.4 to 5.0)
Foreign body in eyes	845 (508 to 1 279)	3 178 (2 008 to 4 509)	213.8 (135.3 to 299.6)	208.9 (135.3 to 288.6)	150 (72 to 259)	621 (338 to 986)	48.1 (28.8 to 73.2)	46.4 (27.5 to 69.5)
Foreign body in other body part	129 (92 to 184)	269 (210 to 345)	24.6 (18.4 to 32.5)	17.8 (13.7 to 22.4)	373 (297 to 474)	966 (795 to 1 198)	98.5 (81.2 to 121.8)	64.2 (53.6 to 78.5)
Environmental heat and cold exposure	218 (158 to 298)	327 (249 to 423)	51.7 (40.6 to 63.8)	23.6 (18.4 to 30.0)	705 (562 to 862)	1 201 (1 018 to 1 422)	210.7 (177.0 to 248.5)	87.5 (75.5 to 102.6)
Exposure to forces of nature	79 (64 to 98)	18 (15 to 22)	15.4 (13.1 to 18.2)	1.1 (0.9 to 1.3)	473 (341 to 638)	1 270 (886 to 1 775)	93.6 (67.4 to 128.2)	79.3 (55.7 to 109.5)
Other unintentional injuries	1 051 (877 to 1 285)	2 295 (1 958 to 2 690)	228.8 (196.9 to 267.2)	146.3 (125.5 to 169.7)	1 379 (1 183 to 1 613)	3 746 (3 247 to 4 310)	429.2 (372.1 to 491.2)	256.8 (224.8 to 291.2)

Cause	Years lived with disability				Disability adjusted life years			
	All-age counts (95% UI)		Age-standardised rates per 100,000 (95% UI)		All-age counts (95% UI)		Age-standardised rates per 100,000 (95% UI)	
	1990	2017	1990	2017	1990	2017	1990	2017
**Transport and unintentional injuries**	**1 512 (1 093 to 2 004)**	**6 135 (4 401 to 8 342)**	**464.9 (339.2 to 616.3)**	**426.9 (306.7 to 579.6)**	**19 130 (17 469 to 20 967)**	**34 106 (31 341 to 37 157)**	**3 801.6 (3 533.4 to 4 101.5)**	**2 102.3 (1 932.1 to 2 298.3)**
**Transport injuries**	**289 (211 to 380)**	**1 593 (1 151 to 2 145)**	**91.0 (66.6 to 119.9)**	**104.8 (75.5 to 140.2)**	**8 161 (7 588 to 8 782)**	**16 396 (14 931 to 17 913)**	**1 603.4 (1 515.2 to 1 697.2)**	**940.3 (858.6 to 1 024.7)**
Road injuries	261 (191 to 345)	1 542 (1 114 to 2 077)	83.3 (61.2 to 109.7)	101.3 (72.8 to 135.6)	7 789 (7 246 to 8 381)	15 232 (13 843 to 16 661)	1 528.8 (1 444.2 to 1 620.0)	874.1 (796.6 to 954.9)
Pedestrian road injuries	71 (51 to 97)	359 (246 to 489)	23.7 (16.9 to 31.8)	24.0 (16.9 to 32.8)	3 850 (3 466 to 4 273)	6 099 (5 500 to 6 696)	780.2 (722.9 to 843.9)	366.2 (332.2 to 402.0)
Cyclist road injuries	36 (25 to 48)	196 (137 to 268)	10.4 (7.4 to 13.7)	12.8 (9.0 to 17.1)	201 (178 to 224)	711 (619 to 809)	42.8 (38.2 to 47.8)	42.9 (37.6 to 49.2)
Motorcyclist road injuries	62 (43 to 84)	385 (266 to 524)	19.1 (13.3 to 26.0)	24.2 (16.8 to 33.1)	1 218 (1 093 to 1 362)	2 681 (2 359 to 3 028)	228.5 (207.7 to 251.4)	144.3 (127.2 to 162.6)
Motor vehicle road injuries	65 (47 to 87)	491 (356 to 655)	21.9 (16.0 to 28.9)	32.2 (23.3 to 43.0)	2 347 (2 124 to 2 553)	5 344 (4 736 to 5 965)	441.6 (403.7 to 475.7)	296.6 (264.0 to 330.2)
Other road injuries	26 (19 to 36)	118 (83 to 163)	8.2 (5.9 to 11.1)	8.0 (5.7 to 11.0)	173 (145 to 199)	402 (348 to 467)	35.7 (30.4 to 40.7)	24.0 (20.7 to 27.9)
Other transport injuries	28 (20 to 37)	51 (36 to 69)	7.7 (5.5 to 10.2)	3.6 (2.5 to 4.8)	372 (330 to 416)	1 164 (1 010 to 1 324)	74.6 (67.5 to 82.4)	66.2 (57.7 to 74.5)
**Unintentional injuries**	**1 223 (896 to 1 626)**	**4 542 (3 238 to 6 221)**	**373.9 (270.0 to 501.3)**	**322.1 (230.7 to 437.8)**	**10 969 (9 798 to 12 281)**	**17 710 (16 115 to 19 655)**	**2 198.2 (2 001.2 to 2 413.7)**	**1 162.1 (1 050.7 to 1 290.1)**
Falls	695 (508 to 921)	3 020 (2 176 to 4 022)	229.5 (167.7 to 301.5)	223.2 (160.1 to 297.5)	1 708 (1 490 to 1 970)	5 339 (4 473 to 6 332)	515.9 (450.9 to 591.6)	401.0 (335.2 to 471.9)
Drowning	8 (5 to 11)	12 (8 to 17)	2.5 (1.7 to 3.4)	0.9 (0.6 to 1.2)	2 726 (2 373 to 3 164)	2 992 (2 678 to 3 314)	476.6 (425.1 to 539.4)	174.9 (157.1 to 192.7)
Fire, heat, and hot substances	146 (91 to 230)	415 (243 to 681)	35.5 (22.0 to 56.0)	26.0 (15.3 to 42.3)	731 (608 to 887)	932 (750 to 1 198)	141.6 (121.2 to 169.8)	61.1 (49.6 to 77.8)
Poisonings	25 (16 to 38)	84 (53 to 127)	5.2 (3.3 to 7.8)	5.0 (3.1 to 7.5)	446 (382 to 521)	540 (480 to 597)	76.8 (68.0 to 87.3)	31.9 (28.6 to 35.1)
Poisoning by carbon monoxide	4 (2 to 6)	9 (6 to 15)	0.8 (0.5 to 1.2)	0.6 (0.3 to 0.9)	127 (109 to 149)	184 (161 to 208)	22.7 (19.9 to 26.1)	10.7 (9.4 to 11.9)
Poisoning by other means	21 (13 to 33)	75 (45 to 116)	4.5 (2.7 to 6.7)	4.4 (2.7 to 6.8)	319 (272 to 378)	355 (313 to 404)	54.1 (47.1 to 62.5)	21.2 (18.8 to 24.0)
Exposure to mechanical forces	167 (115 to 238)	564 (380 to 819)	51.8 (35.6 to 74.1)	37.1 (25.2 to 54.2)	1 144 (1 038 to 1 265)	1 797 (1 569 to 2 083)	245.4 (222.2 to 271.9)	108.1 (93.8 to 126.7)
Unintentional firearm injuries	17 (11 to 25)	33 (21 to 52)	5.1 (3.4 to 7.9)	2.2 (1.4 to 3.4)	411 (363 to 512)	423 (359 to 492)	80.0 (71.1 to 100.5)	23.5 (20.0 to 27.3)
Other exposure to mechanical forces	150 (104 to 215)	531 (356 to 775)	46.6 (31.8 to 67.1)	34.9 (23.6 to 51.0)	733 (608 to 837)	1 374 (1 177 to 1 646)	165.4 (138.4 to 189.0)	84.6 (72.8 to 101.5)
Adverse effects of medical treatment	4 (2 to 6)	19 (12 to 29)	1.1 (0.7 to 1.6)	1.3 (0.8 to 2.0)	456 (306 to 549)	869 (724 to 990)	91.5 (65.2 to 105.6)	64.0 (55.2 to 72.2)
Animal contact	41 (27 to 58)	126 (85 to 181)	10.4 (6.9 to 14.6)	8.1 (5.4 to 11.6)	382 (306 to 477)	318 (267 to 381)	65.7 (54.6 to 79.3)	21.0 (17.7 to 25.0)
Venomous animal contact	24 (15 to 35)	69 (45 to 100)	5.4 (3.5 to 7.7)	4.2 (2.7 to 6.0)	323 (252 to 410)	205 (172 to 243)	52.5 (42.5 to 64.7)	13.4 (11.3 to 15.8)
Non venomous animal contact	17 (10 to 27)	57 (34 to 92)	5.0 (3.1 to 8.0)	4.0 (2.4 to 6.4)	59 (48 to 77)	113 (87 to 151)	13.1 (10.4 to 16.9)	7.6 (5.8 to 10.2)
Foreign body	35 (24 to 50)	90 (60 to 131)	9.4 (6.5 to 12.9)	6.2 (4.1 to 8.9)	2 444 (2 018 to 2 933)	3 460 (3 020 to 4 016)	392.8 (334.7 to 459.0)	215.4 (187.7 to 249.5)
Pulmonary aspiration and foreign body in airway	3 (2 to 4)	3 (2 to 4)	0.6 (0.4 to 0.8)	0.2 (0.1 to 0.2)	2 341 (1 926 to 2 814)	3 296 (2 869 to 3 850)	372.4 (316.5 to 436.8)	205.1 (178.3 to 238.4)
Foreign body in eyes	10 (5 to 18)	42 (23 to 70)	3.2 (1.8 to 5.2)	3.1 (1.8 to 5.0)	10 (5 to 18)	42 (23 to 70)	3.2 (1.8 to 5.2)	3.1 (1.8 to 5.0)
Foreign body in other body part	23 (16 to 30)	46 (32 to 61)	5.5 (3.9 to 7.3)	2.9 (2.1 to 3.9)	93 (77 to 111)	122 (100 to 177)	17.1 (14.5 to 20.2)	7.2 (5.9 to 10.3)
Environmental heat and cold exposure	28 (18 to 39)	45 (30 to 65)	8.2 (5.5 to 11.6)	3.3 (2.2 to 4.8)	201 (177 to 229)	271 (236 to 308)	49.1 (44.4 to 54.6)	17.7 (15.4 to 20.0)
Exposure to forces of nature	21 (15 to 30)	40 (29 to 57)	4.2 (3.1 to 5.8)	2.5 (1.8 to 3.4)	51 (45 to 61)	54 (43 to 70)	10.2 (8.9 to 11.9)	3.3 (2.6 to 4.3)
Other unintentional injuries	53 (38 to 72)	125 (88 to 172)	16.0 (11.5 to 21.6)	8.5 (6.0 to 11.7)	679 (612 to 749)	1 138 (1 003 to 1 289)	132.5 (121.2 to 144.6)	63.8 (56.8 to 71.7)

Death, YLL, incidence, prevalence, YLD, and DALY counts and rates per 100,000 for all injuries in San Luis Potosí in 1990 and 2017

Cause	Deaths				Years of life lost			
	All-age counts (95% UI)		Age-standardised rates per 100,000 (95% UI)		All-age counts (95% UI)		Age-standardised rates per 100,000 (95% UI)	
	1990	2017	1990	2017	1990	2017	1990	2017
**Transport and unintentional injuries**	**1 139 (1 096 to 1 185)**	**1 016 (952 to 1 084)**	**65.8 (63.9 to 67.8)**	**35.7 (33.4 to 38.2)**	**61 318 (58 081 to 64 909)**	**44 564 (41 263 to 48 294)**	**2 906.6 (2 782.7 to 3 039.1)**	**1 549.0 (1 433.5 to 1 679.2)**
**Transport injuries**	**512 (488 to 537)**	**521 (480 to 564)**	**28.7 (27.5 to 30.0)**	**18.1 (16.7 to 19.6)**	**28 179 (26 513 to 29 821)**	**25 204 (23 099 to 27 378)**	**1 376.1 (1 307.8 to 1 445.2)**	**866.8 (794.4 to 941.2)**
Road injuries	496 (473 to 521)	497 (458 to 539)	27.8 (26.6 to 29.0)	17.3 (16.0 to 18.7)	27 279 (25 645 to 28 904)	24 089 (22 084 to 26 139)	1 332.6 (1 265.7 to 1 400.1)	828.4 (760.2 to 899.6)
Pedestrian road injuries	237 (223 to 252)	216 (196 to 237)	14.0 (13.3 to 14.7)	7.6 (6.9 to 8.3)	12 994 (11 419 to 13 984)	9 315 (8 419 to 10 336)	607.8 (568.9 to 648.6)	324.1 (293.0 to 360.4)
Cyclist road injuries	16 (14 to 18)	26 (23 to 29)	0.9 (0.8 to 1.0)	0.9 (0.8 to 1.0)	885 (790 to 992)	1 181 (1 033 to 1 338)	43.9 (39.4 to 48.7)	40.8 (35.8 to 46.1)
Motorcyclist road injuries	70 (64 to 78)	66 (58 to 75)	3.6 (3.3 to 4.0)	2.3 (2.0 to 2.5)	4 164 (3 755 to 4 614)	3 767 (3 280 to 4 275)	201.0 (182.8 to 220.8)	127.3 (111.2 to 144.3)
Motor vehicle road injuries	152 (141 to 165)	172 (155 to 189)	8.2 (7.6 to 8.8)	6.0 (5.4 to 6.5)	8 751 (7 989 to 9 563)	8 975 (8 032 to 9 912)	428.0 (395.8 to 462.0)	307.1 (275.1 to 338.7)
Other road injuries	20 (16 to 23)	18 (15 to 20)	1.1 (0.9 to 1.2)	0.6 (0.5 to 0.7)	1 085 (903 to 1 271)	851 (727 to 989)	51.9 (43.9 to 60.2)	29.2 (25.0 to 33.8)
Other transport injuries	16 (15 to 18)	23 (21 to 26)	0.9 (0.9 to 1.0)	0.8 (0.7 to 0.9)	900 (814 to 1 006)	1 115 (972 to 1 277)	43.5 (39.7 to 48.4)	38.4 (33.5 to 44.0)
**Unintentional injuries**	**627 (597 to 659)**	**495 (464 to 529)**	**37.1 (35.9 to 38.5)**	**17.6 (16.5 to 18.8)**	**33 139 (30 830 to 35 615)**	**19 360 (17 719 to 21 270)**	**1 530.4 (1 446.9 to 1 618.2)**	**682.2 (623.5 to 750.5)**
Falls	149 (143 to 157)	177 (162 to 190)	11.7 (11.2 to 12.3)	6.3 (5.8 to 6.8)	4 758 (4 475 to 5 058)	4 069 (3 739 to 4 422)	285.2 (270.9 to 300.4)	144.5 (132.8 to 156.9)
Drowning	151 (141 to 163)	81 (74 to 89)	7.4 (7.0 to 7.8)	2.8 (2.6 to 3.1)	9 504 (8 733 to 10 489)	4 356 (3 936 to 4 876)	407.0 (379.1 to 441.6)	150.9 (135.5 to 169.0)
Fire, heat, and hot substances	47 (43 to 51)	31 (29 to 33)	2.8 (2.6 to 3.0)	1.1 (1.0 to 1.2)	2 462 (2 148 to 2 821)	1 032 (927 to 1 140)	110.0 (99.4 to 122.7)	36.5 (32.9 to 40.5)
Poisonings	28 (26 to 31)	17 (15 to 18)	1.4 (1.3 to 1.5)	0.6 (0.5 to 0.6)	1 802 (1 605 to 2 047)	834 (744 to 923)	76.3 (69.3 to 84.8)	29.1 (25.9 to 32.3)
Poisoning by carbon monoxide	12 (11 to 13)	8 (7 to 9)	0.6 (0.6 to 0.7)	0.3 (0.3 to 0.3)	732 (644 to 837)	388 (342 to 435)	31.8 (28.6 to 35.6)	13.5 (11.9 to 15.1)
Poisoning by other means	16 (15 to 18)	9 (8 to 10)	0.8 (0.7 to 0.9)	0.3 (0.3 to 0.3)	1 070 (938 to 1 226)	446 (393 to 501)	44.5 (40.0 to 49.9)	15.7 (13.8 to 17.6)
Exposure to mechanical forces	85 (80 to 90)	45 (41 to 49)	4.8 (4.5 to 5.0)	1.6 (1.4 to 1.7)	4 675 (4 359 to 5 025)	1 977 (1 791 to 2 179)	228.3 (214.5 to 242.9)	68.5 (62.1 to 75.3)
Unintentional firearm injuries	37 (33 to 43)	14 (12 to 16)	1.9 (1.8 to 2.1)	0.5 (0.4 to 0.6)	2 097 (1 896 to 2 435)	689 (567 to 793)	100.6 (91.1 to 118.2)	23.6 (19.4 to 27.2)
Other exposure to mechanical forces	48 (43 to 53)	31 (28 to 34)	2.8 (2.5 to 3.1)	1.1 (1.0 to 1.2)	2 578 (2 248 to 2 842)	1 288 (1 148 to 1 446)	127.7 (112.1 to 140.0)	44.9 (40.1 to 50.2)
Adverse effects of medical treatment	30 (27 to 33)	36 (32 to 40)	2.0 (1.8 to 2.2)	1.3 (1.1 to 1.4)	1 383 (1 187 to 1 581)	1 113 (960 to 1 298)	65.5 (58.0 to 73.4)	39.9 (34.4 to 45.2)
Animal contact	17 (15 to 19)	9 (8 to 10)	0.9 (0.8 to 0.9)	0.3 (0.3 to 0.4)	1 085 (931 to 1 264)	384 (337 to 437)	43.7 (38.6 to 49.7)	13.7 (12.0 to 15.6)
Venomous animal contact	11 (10 to 13)	5 (4 to 6)	0.5 (0.5 to 0.6)	0.2 (0.2 to 0.2)	765 (636 to 921)	220 (186 to 267)	29.6 (25.1 to 35.1)	7.9 (6.7 to 9.6)
Non-venomous animal contact	6 (4 to 6)	4 (3 to 5)	0.3 (0.2 to 0.4)	0.1 (0.1 to 0.2)	319 (233 to 376)	164 (137 to 193)	14.1 (10.3 to 16.1)	5.8 (4.8 to 6.8)
Foreign body	76 (69 to 85)	74 (65 to 85)	3.7 (3.4 to 3.9)	2.7 (2.3 to 3.0)	5 108 (4 460 to 5 793)	4 334 (3 607 to 5 263)	202.2 (180.4 to 224.8)	155.6 (129.9 to 189.5)
Pulmonary aspiration and foreign body in airway	73 (65 to 80)	73 (64 to 83)	3.5 (3.2 to 3.7)	2.6 (2.3 to 3.0)	4 858 (4 233 to 5 528)	4 239 (3 518 to 5 169)	191.7 (171.2 to 213.5)	152.3 (126.0 to 186.3)
Foreign body in eyes								
Foreign body in other body part	4 (3 to 4)	2 (1 to 3)	0.2 (0.1 to 0.2)	0.1 (0.0 to 0.1)	250 (172 to 297)	95 (77 to 156)	10.5 (7.5 to 12.2)	3.3 (2.7 to 5.4)
Environmental heat and cold exposure	14 (13 to 15)	7 (6 to 8)	0.9 (0.9 to 1.0)	0.2 (0.2 to 0.3)	638 (582 to 695)	245 (218 to 273)	32.6 (30.3 to 35.0)	8.7 (7.7 to 9.5)
Exposure to forces of nature	1 (1 to 1)	0 (0 to 0)	0.0 (0.0 to 0.0)	0.0 (0.0 to 0.0)	51 (46 to 57)	23 (21 to 26)	2.3 (2.1 to 2.5)	0.8 (0.7 to 0.9)
Other unintentional injuries	29 (26 to 31)	19 (17 to 21)	1.5 (1.4 to 1.6)	0.7 (0.6 to 0.7)	1 678 (1 580 to 1 880)	992 (883 to 1 117)	77.3 (71.3 to 83.6)	34.0 (30.2 to 38.1)

Cause	Incidence				Prevalence			
	All-age counts (95% UI)		Age-standardised rates per 100,000 (95% UI)		All-age counts (95% UI)		Age-standardised rates per 100,000 (95% UI)	
	1990	2017	1990	2017	1990	2017	1990	2017
**Transport and unintentional injuries**	**79 044 (72 480 to 86 424)**	**120 811 (110 604 to 132 641)**	**4 300.4 (3 913.5 to 4 689.7)**	**4 259.9 (3 894.4 to 4 665.7)**	**152 037 (143 067 to 162 881)**	**257 693 (242 690 to 277 196)**	**9 917.5 (9 348.7 to 10 630.0)**	**9 115.5 (8 587.1 to 9 793.3)**
**Transport injuries**	**7 067 (6 046 to 8 223)**	**16 328 (14 064 to 18 703)**	**379.5 (329.8 to 433.3)**	**566.3 (487.3 to 648.0)**	**17 413 (16 270 to 18 541)**	**46 754 (43 534 to 50 171)**	**1 183.3 (1 105.0 to 1 262.1)**	**1 654.5 (1 541.8 to 1 775.8)**
Road injuries	6 725 (5 753 to 7 877)	16 038 (13 792 to 18 413)	362.7 (312.9 to 415.7)	556.1 (478.1 to 638.1)	15 869 (14 833 to 16 938)	45 288 (42 052 to 48 715)	1 087.7 (1 015.3 to 1 163.5)	1 602.6 (1 488.5 to 1 723.7)
Pedestrian road injuries	993 (810 to 1 213)	1 905 (1 539 to 2 291)	56.2 (46.5 to 67.1)	66.6 (53.7 to 80.1)	3 782 (3 262 to 4 428)	8 856 (7 746 to 10 290)	262.7 (229.7 to 305.6)	314.4 (275.2 to 365.7)
Cyclist road injuries	1 203 (941 to 1 529)	2 647 (2 124 to 3 267)	59.6 (48.0 to 74.5)	92.0 (73.9 to 112.8)	2 122 (1 783 to 2 481)	5 935 (5 044 to 6 895)	137.7 (116.6 to 159.3)	209.3 (178.0 to 242.8)
Motorcyclist road injuries	808 (640 to 1 005)	1 576 (1 214 to 2 007)	42.1 (34.0 to 51.8)	54.0 (41.7 to 68.7)	3 021 (2 621 to 3 479)	7 285 (6 229 to 8 506)	204.6 (177.3 to 235.7)	256.9 (219.7 to 298.9)
Motor vehicle road injuries	2 299 (1 880 to 2 738)	6 958 (5 788 to 8 319)	128.2 (106.4 to 151.6)	240.9 (200.8 to 287.2)	4 284 (3 758 to 4 969)	16 199 (14 068 to 18 948)	302.7 (264.3 to 350.2)	574.1 (498.1 to 670.9)
Other road injuries	1 423 (1 133 to 1 754)	2 951 (2 350 to 3 586)	76.5 (61.5 to 92.4)	102.6 (81.8 to 124.2)	2 660 (2 287 to 3 191)	7 013 (5 847 to 8 521)	179.9 (152.3 to 215.1)	248.0 (206.8 to 301.3)
Other transport injuries	342 (259 to 460)	290 (236 to 356)	16.9 (13.6 to 21.0)	10.2 (8.3 to 12.6)	1 544 (1 281 to 1 831)	1 466 (1 270 to 1 671)	95.6 (80.8 to 111.0)	51.9 (45.0 to 59.1)
**Unintentional injuries**	**71 977 (65 458 to 79 455)**	**104 484 (94 513 to 115 865)**	**3 920.9 (3 540.1 to 4 316.1)**	**3 693.6 (3 336.5 to 4 091.5)**	**134 624 (125 761 to 145 145)**	**210 939 (195 960 to 230 004)**	**8 734.1 (8 164.6 to 9 430.3)**	**7 461.0 (6 929.9 to 8 141.6)**
Falls	31 461 (27 844 to 35 695)	56 632 (50 510 to 63 743)	1 836.8 (1 645.5 to 2 066.0)	2 013.8 (1 793.1 to 2 275.5)	54 594 (48 394 to 62 093)	108 602 (97 283 to 124 296)	3 731.6 (3 324.7 to 4 231.1)	3 857.0 (3 453.2 to 4 417.5)
Drowning	130 (113 to 151)	71 (57 to 90)	6.4 (5.5 to 7.2)	2.5 (2.0 to 3.1)	600 (527 to 687)	442 (389 to 510)	40.4 (35.7 to 46.0)	15.7 (13.8 to 18.2)
Fire, heat, and hot substances	2 854 (2 312 to 3 564)	2 706 (2 230 to 3 250)	117.0 (97.2 to 141.4)	95.9 (78.8 to 115.2)	26 993 (22 406 to 33 782)	32 089 (26 536 to 40 590)	1 566.8 (1 313.1 to 1 907.8)	1 124.4 (930.6 to 1 419.9)
Poisonings	923 (672 to 1 254)	705 (489 to 979)	34.4 (25.9 to 45.4)	25.1 (17.5 to 34.9)	769 (591 to 950)	840 (620 to 1 058)	39.8 (31.0 to 49.2)	29.1 (21.5 to 36.7)
Poisoning by carbon monoxide	180 (115 to 273)	142 (76 to 235)	7.4 (5.1 to 10.6)	5.0 (2.7 to 8.2)	118 (91 to 155)	112 (86 to 144)	6.3 (4.9 to 8.2)	3.9 (3.0 to 5.0)
Poisoning by other means	743 (531 to 1 041)	563 (374 to 815)	26.9 (19.6 to 36.8)	20.1 (13.4 to 29.2)	651 (473 to 831)	727 (504 to 943)	33.5 (24.6 to 42.6)	25.2 (17.5 to 32.7)
Exposure to mechanical forces	16 826 (14 295 to 19 666)	18 518 (15 929 to 21 576)	840.9 (726.8 to 970.4)	642.8 (555.4 to 748.4)	33 366 (29 367 to 38 304)	46 478 (40 490 to 53 764)	2 221.3 (1 952.3 to 2 538.9)	1 640.8 (1 430.8 to 1 899.1)
Unintentional firearm injuries	1 176 (863 to 1 562)	725 (517 to 972)	57.7 (43.7 to 75.7)	25.0 (18.0 to 33.5)	4 433 (3 538 to 5 320)	3 806 (3 056 to 4 623)	300.4 (241.7 to 363.2)	134.8 (108.6 to 164.0)
Other exposure to mechanical forces	15 650 (13 273 to 18 292)	17 793 (15 307 to 20 677)	783.2 (675.8 to 909.8)	617.8 (532.7 to 718.2)	28 932 (24 589 to 34 103)	42 672 (36 445 to 50 305)	1 920.9 (1 642.6 to 2 255.4)	1 506.0 (1 287.0 to 1 774.2)
Adverse effects of medical treatment	1 449 (1 218 to 1 694)	2 824 (2 404 to 3 279)	86.2 (73.5 to 99.6)	100.9 (85.2 to 116.4)	111 (86 to 139)	216 (166 to 269)	6.6 (5.0 to 8.1)	7.7 (5.9 to 9.5)
Animal contact	5 964 (5 016 to 7 217)	6 852 (5 989 to 7 844)	296.2 (255.4 to 343.2)	241.4 (210.9 to 276.2)	6 198 (5 320 to 7 182)	8 457 (7 449 to 9 578)	390.8 (340.0 to 444.6)	298.6 (263.7 to 337.4)
Venomous animal contact	2 119 (1 629 to 2 796)	2 731 (2 199 to 3 339)	105.2 (81.7 to 132.1)	96.1 (77.4 to 117.2)	1 369 (1 129 to 1 624)	2 097 (1 761 to 2 426)	81.4 (68.1 to 94.8)	73.8 (62.1 to 85.2)
Non-venomous animal contact	3 846 (3 148 to 4 729)	4 120 (3 468 to 4 928)	191.1 (160.6 to 229.7)	145.3 (121.8 to 173.7)	4 829 (3 989 to 5 786)	6 359 (5 391 to 7 458)	309.4 (259.7 to 363.5)	224.8 (190.8 to 263.0)
Foreign body	7 682 (3 755 to 12 056)	12 085 (5 326 to 20 767)	466.4 (198.6 to 767.8)	429.2 (187.4 to 742.6)	3 224 (2 440 to 4 323)	4 612 (3 412 to 6 326)	202.6 (155.0 to 270.3)	163.6 (120.9 to 223.5)
Pulmonary aspiration and foreign body in airway	196 (138 to 269)	109 (76 to 156)	7.6 (5.7 to 10.1)	3.8 (2.7 to 5.5)	133 (95 to 181)	99 (81 to 122)	7.0 (5.2 to 9.3)	3.5 (2.8 to 4.2)
Foreign body in eyes	6 944 (3 025 to 11 371)	11 449 (4 616 to 20 117)	435.1 (166.8 to 734.3)	406.6 (161.3 to 729.2)	1 481 (756 to 2 414)	2 595 (1 446 to 4 216)	99.9 (55.7 to 161.7)	92.8 (52.1 to 150.2)
Foreign body in other body part	542 (396 to 758)	527 (421 to 653)	23.7 (18.0 to 31.3)	18.7 (14.9 to 23.3)	1 660 (1 337 to 2 103)	1 917 (1 604 to 2 342)	95.7 (78.5 to 118.8)	67.4 (56.5 to 82.1)
Environmental heat and cold exposure	955 (698 to 1 249)	486 (382 to 595)	49.5 (38.8 to 60.5)	17.2 (13.5 to 20.9)	3 254 (2 675 to 3 918)	1 812 (1 570 to 2 137)	201.7 (171.6 to 239.3)	64.1 (55.7 to 75.7)
Exposure to forces of nature	134 (110 to 168)	31 (26 to 37)	5.8 (4.9 to 7.0)	1.1 (0.9 to 1.3)	377 (279 to 510)	1 062 (745 to 1 466)	19.0 (13.9 to 25.5)	36.9 (26.0 to 51.0)
Other unintentional injuries	3 586 (2 925 to 4 414)	3 573 (2 988 to 4 206)	181.3 (152.1 to 217.0)	124.4 (104.0 to 146.6)	5 138 (4 400 to 5 925)	6 329 (5 488 to 7 237)	332.1 (285.9 to 380.1)	223.1 (193.7 to 255.0)

Cause	Years lived with disability				Disability adjusted life years			
	All-age counts (95% UI)		Age-standardised rates per 100,000 (95% UI)		All-age counts (95% UI)		Age-standardised rates per 100,000 (95% UI)	
	1990	2017	1990	2017	1990	2017	1990	2017
**Transport and unintentional injuries**	**6 744 (4 964 to 8 926)**	**10 988 (7 909 to 14 719)**	**435.2 (319.4 to 577.4)**	**388.6 (279.6 to 521.4)**	**68 061 (64 337 to 72 522)**	**55 552 (50 972 to 60 832)**	**3 341.8 (3 173.2 to 3 538.8)**	**1 937.7 (1 776.6 to 2 123.2)**
**Transport injuries**	**1 201 (879 to 1 580)**	**2 848 (2 074 to 3 811)**	**79.3 (58.6 to 104.4)**	**100.5 (73.2 to 134.5)**	**29 380 (27 610 to 31 145)**	**28 052 (25 775 to 30 591)**	**1 455.5 (1 384.6 to 1 529.4)**	**967.4 (890.1 to 1 054.0)**
Road injuries	1 101 (809 to 1 453)	2 770 (2 014 to 3 711)	73.4 (54.2 to 96.8)	97.8 (71.1 to 130.9)	28 380 (26 673 to 30 068)	26 859 (24 695 to 29 282)	1 406.0 (1 337.3 to 1 478.6)	926.2 (852.4 to 1 010.3)
Pedestrian road injuries	273 (193 to 369)	565 (401 to 768)	18.4 (13.0 to 24.9)	20.0 (14.2 to 27.3)	12 666 (11 705 to 13 641)	9 880 (8 966 to 10 927)	626.2 (585.1 to 667.4)	344.1 (312.5 to 380.5)
Cyclist road injuries	150 (106 to 199)	360 (252 to 481)	9.5 (6.7 to 12.5)	12.7 (8.9 to 16.9)	1 035 (925 to 1 150)	1 542 (1 358 to 1 734)	53.4 (48.1 to 59.1)	53.5 (47.2 to 60.2)
Motorcyclist road injuries	214 (150 to 291)	452 (310 to 617)	14.1 (9.9 to 19.1)	15.9 (10.9 to 21.7)	4 378 (3 956 to 4 830)	4 218 (3 730 to 4 783)	215.2 (195.9 to 235.5)	143.1 (126.3 to 162.3)
Motor vehicle road injuries	309 (223 to 409)	1 030 (742 to 1 370)	21.2 (15.4 to 27.9)	36.4 (26.3 to 48.5)	9 060 (8 302 to 9 861)	10 005 (9 001 to 11 002)	449.2 (415.6 to 483.6)	343.5 (309.4 to 377.6)
Other road injuries	156 (112 to 211)	363 (259 to 503)	10.2 (7.4 to 13.8)	12.8 (9.1 to 17.7)	1 240 (1 051 to 1 433)	1 215 (1 041 to 1 424)	62.1 (53.7 to 70.9)	42.0 (35.9 to 49.2)
Other transport injuries	100 (71 to 133)	78 (56 to 104)	6.0 (4.3 to 7.8)	2.8 (2.0 to 3.7)	1 001 (905 to 1 108)	1 193 (1 043 to 1 349)	49.5 (45.0 to 54.4)	41.2 (36.0 to 46.4)
**Unintentional injuries**	**5 542 (4 034 to 7 322)**	**8 140 (5 777 to 11 021)**	**355.8 (258.4 to 473.1)**	**288.1 (204.5 to 390.1)**	**38 681 (35 867 to 41 937)**	**27 500 (24 615 to 30 715)**	**1 886.3 (1 754.1 to 2 036.0)**	**970.3 (868.0 to 1 083.5)**
Falls	3 296 (2 434 to 4 303)	5 591 (4 018 to 7 408)	218.8 (160.1 to 284.5)	198.4 (142.6 to 263.4)	8 054 (7 147 to 9 137)	9 661 (8 018 to 11 497)	504.1 (445.9 to 573.5)	342.9 (284.6 to 407.3)
Drowning	34 (24 to 47)	23 (16 to 32)	2.2 (1.6 to 3.0)	0.8 (0.6 to 1.1)	9 539 (8 737 to 10 485)	4 380 (3 943 to 4 898)	409.2 (381.1 to 443.7)	151.8 (136.5 to 169.9)
Fire, heat, and hot substances	590 (376 to 914)	622 (368 to 1 025)	33.2 (21.0 to 51.5)	21.8 (12.9 to 35.8)	3 052 (2 658 to 3 516)	1 654 (1 370 to 2 054)	143.2 (125.7 to 164.4)	58.3 (48.3 to 72.4)
Poisonings	91 (55 to 136)	97 (58 to 147)	4.6 (2.8 to 6.8)	3.4 (2.0 to 5.1)	1 892 (1 691 to 2 135)	931 (824 to 1 034)	80.9 (73.6 to 89.7)	32.5 (28.7 to 36.0)
Poisoning by carbon monoxide	13 (8 to 20)	11 (7 to 17)	0.6 (0.4 to 1.0)	0.4 (0.2 to 0.6)	744 (656 to 851)	399 (351 to 446)	32.5 (29.2 to 36.2)	13.9 (12.2 to 15.5)
Poisoning by other means	78 (46 to 117)	86 (50 to 134)	3.9 (2.4 to 5.9)	3.0 (1.7 to 4.6)	1 148 (1 009 to 1 303)	532 (463 to 606)	48.4 (43.5 to 54.0)	18.6 (16.2 to 21.2)
Exposure to mechanical forces	793 (553 to 1 130)	1 006 (681 to 1 464)	51.9 (36.0 to 74.5)	35.5 (24.0 to 51.7)	5 468 (5 062 to 5 952)	2 983 (2 591 to 3 498)	280.2 (259.0 to 307.6)	103.9 (90.2 to 122.0)
Unintentional firearm injuries	92 (61 to 139)	69 (44 to 110)	6.0 (4.0 to 9.2)	2.4 (1.6 to 3.9)	2 189 (1 985 to 2 527)	758 (625 to 868)	106.6 (97.1 to 123.7)	26.0 (21.6 to 29.9)
Other exposure to mechanical forces	702 (485 to 1 001)	937 (632 to 1 370)	45.9 (31.9 to 65.6)	33.0 (22.3 to 48.3)	3 279 (2 892 to 3 647)	2 225 (1 884 to 2 674)	173.6 (154.1 to 195.3)	77.9 (65.9 to 93.7)
Adverse effects of medical treatment	15 (9 to 22)	29 (18 to 44)	0.9 (0.6 to 1.3)	1.0 (0.6 to 1.6)	1 398 (1 205 to 1 591)	1 142 (988 to 1 623)	66.4 (59.0 to 74.3)	40.9 (35.3 to 58.1)
Animal contact	162 (109 to 229)	188 (125 to 269)	9.3 (6.3 to 13.3)	6.6 (4.4 to 9.4)	1 247 (1 083 to 1 445)	572 (490 to 674)	53.0 (46.7 to 60.7)	20.3 (17.4 to 23.9)
Venomous animal contact	87 (55 to 128)	98 (64 to 143)	4.6 (3.0 to 6.6)	3.4 (2.2 to 5.0)	852 (720 to 1 010)	318 (267 to 384)	34.2 (29.5 to 39.9)	11.3 (9.5 to 13.6)
Non-venomous animal contact	75 (46 to 120)	90 (53 to 145)	4.8 (2.9 to 7.5)	3.2 (1.9 to 5.1)	395 (297 to 465)	253 (205 to 319)	18.8 (14.2 to 22.5)	8.9 (7.2 to 11.2)
Foreign body	206 (133 to 303)	267 (164 to 430)	12.8 (8.3 to 19.0)	9.5 (5.8 to 15.3)	5 313 (4 665 to 6 017)	4 600 (3 855 to 5 552)	214.9 (192.6 to 238.6)	165.1 (137.9 to 199.7)
Pulmonary aspiration and foreign body in airway	7 (5 to 10)	4 (3 to 6)	0.4 (0.3 to 0.5)	0.1 (0.1 to 0.2)	4 865 (4 239 to 5 535)	4 243 (3 523 to 5 174)	192.1 (171.5 to 213.8)	152.5 (126.2 to 186.4)
Foreign body in eyes	95 (48 to 167)	178 (91 to 307)	6.7 (3.5 to 11.6)	6.2 (3.3 to 11.0)	95 (48 to 167)	178 (91 to 307)	6.7 (3.5 to 11.6)	6.2 (3.3 to 11.0)
Foreign body in other body part	103 (72 to 137)	90 (64 to 119)	5.7 (4.0 to 7.5)	3.1 (2.2 to 4.2)	353 (282 to 411)	185 (152 to 244)	16.2 (13.3 to 18.6)	6.5 (5.3 to 8.5)
Environmental heat and cold exposure	130 (88 to 183)	70 (46 to 101)	8.1 (5.5 to 11.4)	2.5 (1.6 to 3.6)	768 (692 to 848)	315 (278 to 356)	40.7 (37.1 to 44.9)	11.1 (9.8 to 12.6)
Exposure to forces of nature	19 (14 to 27)	35 (25 to 49)	1.0 (0.7 to 1.3)	1.2 (0.9 to 1.7)	71 (63 to 80)	58 (48 to 72)	3.3 (2.9 to 3.7)	2.0 (1.7 to 2.5)
Other unintentional injuries	206 (148 to 271)	212 (149 to 289)	13.1 (9.5 to 17.2)	7.5 (5.2 to 10.2)	1 884 (1 738 to 2 059)	1 204 (1 072 to 1 353)	90.4 (83.2 to 98.2)	41.4 (36.9 to 46.4)

Death, YLL, incidence, prevalence, YLD, and DALY counts and rates per 100,000 for all injuries in Sinaloa in 1990 and 2017

Cause	Deaths				Years of life lost			
	All-age counts (95% UI)		Age-standardised rates per 100,000 (95% UI)		All-age counts (95% UI)		Age-standardised rates per 100,000 (95% UI)	
	1990	2017	1990	2017	1990	2017	1990	2017
**Transport and unintentional injuries**	**1 335 (1 251 to 1 431)**	**1 045 (973 to 1 117)**	**69.4 (66.5 to 72.7)**	**34.5 (32.2 to 36.9)**	**75 344 (68 571 to 82 952)**	**44 173 (41 009 to 47 476)**	**3 169.5 (2 942.7 to 3 426.7)**	**1 448.0 (1 345.1 to 1 556.9)**
**Transport injuries**	**689 (647 to 739)**	**590 (541 to 641)**	**34.4 (32.7 to 36.4)**	**19.1 (17.6 to 20.8)**	**39 244 (36 120 to 42 966)**	**27 357 (25 068 to 29 727)**	**1 677.7 (1 569.9 to 1 806.5)**	**885.8 (811.9 to 961.4)**
Road injuries	660 (620 to 708)	551 (505 to 598)	32.9 (31.3 to 34.8)	17.9 (16.4 to 19.4)	37 657 (34 636 to 41 225)	25 551 (23 396 to 27 725)	1 608.4 (1 504.6 to 1 735.1)	827.6 (758.3 to 898.4)
Pedestrian road injuries	328 (302 to 359)	264 (243 to 288)	17.3 (16.3 to 18.5)	8.7 (8.0 to 9.4)	18 406 (16 987 to 20 941)	10 958 (10 019 to 11 988)	786.4 (718.3 to 871.7)	358.1 (328.2 to 391.5)
Cyclist road injuries	17 (15 to 19)	23 (20 to 25)	0.8 (0.8 to 0.9)	0.7 (0.7 to 0.8)	947 (841 to 1 064)	985 (876 to 1 110)	41.5 (37.2 to 46.1)	31.9 (28.4 to 35.9)
Motorcyclist road injuries	87 (79 to 95)	64 (56 to 72)	4.0 (3.6 to 4.4)	2.0 (1.8 to 2.3)	5 160 (4 626 to 5 784)	3 548 (3 096 to 4 075)	218.1 (197.5 to 239.9)	113.6 (99.2 to 130.1)
Motor vehicle road injuries	209 (192 to 228)	185 (164 to 206)	9.9 (9.2 to 10.7)	5.9 (5.3 to 6.6)	12 058 (11 026 to 13 241)	9 303 (8 210 to 10 424)	516.4 (475.7 to 564.6)	299.6 (264.2 to 335.4)
Other road injuries	19 (17 to 22)	16 (14 to 18)	1.0 (0.8 to 1.1)	0.5 (0.5 to 0.6)	1 086 (912 to 1 266)	756 (645 to 874)	46.0 (39.5 to 53.3)	24.4 (20.9 to 28.1)
Other transport injuries	29 (26 to 32)	39 (34 to 44)	1.5 (1.4 to 1.6)	1.3 (1.1 to 1.4)	1 587 (1 425 to 1 772)	1 806 (1 579 to 2 072)	69.3 (63.0 to 77.0)	58.2 (51.0 to 66.6)
**Unintentional injuries**	**646 (597 to 700)**	**454 (425 to 485)**	**35.0 (33.2 to 36.8)**	**15.4 (14.4 to 16.4)**	**36 100 (32 152 to 40 634)**	**16 816 (15 518 to 18 270)**	**1 491.7 (1 361.2 to 1 642.8)**	**562.2 (519.2 to 613.0)**
Falls	118 (112 to 125)	148 (136 to 160)	9.5 (9.0 to 10.0)	5.1 (4.7 to 5.6)	3 805 (3 512 to 4 157)	3 134 (2 859 to 3 396)	214.9 (202.2 to 228.9)	104.8 (95.7 to 113.3)
Drowning	206 (184 to 231)	91 (83 to 99)	9.0 (8.2 to 9.8)	3.0 (2.7 to 3.3)	13 380 (11 541 to 15 586)	4 624 (4 172 to 5 128)	509.6 (449.8 to 580.3)	153.8 (138.7 to 171.0)
Fire, heat, and hot substances	46 (41 to 52)	28 (25 to 30)	2.6 (2.5 to 2.9)	1.0 (0.9 to 1.0)	2 506 (2 103 to 3 011)	876 (793 to 956)	104.0 (91.1 to 121.0)	29.5 (26.6 to 32.1)
Poisonings	23 (21 to 27)	12 (11 to 14)	1.1 (1.0 to 1.2)	0.4 (0.4 to 0.4)	1 488 (1 289 to 1 749)	585 (523 to 641)	57.9 (51.4 to 66.7)	19.4 (17.4 to 21.3)
Poisoning by carbon monoxide	6 (5 to 6)	4 (3 to 4)	0.3 (0.2 to 0.3)	0.1 (0.1 to 0.1)	345 (296 to 409)	167 (144 to 188)	13.7 (11.9 to 15.8)	5.5 (4.7 to 6.2)
Poisoning by other means	18 (16 to 20)	9 (8 to 10)	0.8 (0.7 to 0.9)	0.3 (0.3 to 0.3)	1 143 (982 to 1 357)	418 (373 to 464)	44.3 (38.9 to 51.4)	13.9 (12.4 to 15.4)
Exposure to mechanical forces	77 (72 to 82)	52 (47 to 57)	4.0 (3.7 to 4.2)	1.7 (1.5 to 1.8)	4 240 (3 903 to 4 636)	2 302 (2 056 to 2 548)	184.1 (171.6 to 198.8)	74.5 (66.4 to 82.3)
Unintentional firearm injuries	41 (35 to 45)	26 (19 to 29)	2.0 (1.8 to 2.2)	0.8 (0.6 to 0.9)	2 320 (1 990 to 2 557)	1 259 (946 to 1 446)	98.8 (85.7 to 107.9)	40.4 (30.4 to 46.4)
Other exposure to mechanical forces	36 (33 to 40)	26 (23 to 31)	2.0 (1.8 to 2.2)	0.9 (0.8 to 1.0)	1 919 (1 702 to 2 184)	1 044 (914 to 1 308)	85.3 (77.0 to 96.0)	34.0 (29.9 to 42.6)
Adverse effects of medical treatment	32 (29 to 37)	35 (31 to 47)	2.1 (1.9 to 2.4)	1.2 (1.1 to 1.6)	1 468 (1 258 to 1 729)	1 005 (865 to 1 473)	67.1 (59.1 to 77.2)	34.2 (29.8 to 50.4)
Animal contact	23 (19 to 28)	9 (8 to 10)	1.0 (0.9 to 1.2)	0.3 (0.3 to 0.3)	1 560 (1 238 to 1 981)	361 (324 to 405)	57.3 (46.7 to 71.2)	12.3 (11.0 to 14.0)
Venomous animal contact	20 (16 to 25)	7 (6 to 8)	0.9 (0.8 to 1.1)	0.2 (0.2 to 0.3)	1 427 (1 097 to 1 881)	296 (245 to 337)	51.8 (40.9 to 64.9)	10.2 (8.4 to 11.7)
Non-venomous animal contact	2 (2 to 4)	2 (1 to 3)	0.1 (0.1 to 0.2)	0.1 (0.0 to 0.1)	133 (106 to 239)	65 (53 to 114)	5.5 (4.5 to 9.2)	2.2 (1.8 to 3.8)
Foreign body	74 (65 to 85)	53 (48 to 59)	3.3 (2.9 to 3.6)	1.8 (1.7 to 2.0)	5 135 (4 334 to 6 103)	2 720 (2 354 to 3 161)	187.2 (161.2 to 218.2)	94.6 (81.0 to 111.5)
Pulmonary aspiration and foreign body in airway	70 (61 to 81)	52 (47 to 57)	3.1 (2.8 to 3.4)	1.8 (1.6 to 2.0)	4 847 (4 081 to 5 773)	2 626 (2 266 to 3 055)	176.3 (151.6 to 205.8)	91.5 (78.1 to 107.9)
Foreign body in eyes								
Foreign body in other body part	4 (3 to 5)	2 (1 to 2)	0.2 (0.1 to 0.2)	0.1 (0.0 to 0.1)	289 (177 to 357)	94 (77 to 132)	11.0 (6.8 to 13.3)	3.1 (2.6 to 4.4)
Environmental heat and cold exposure	12 (11 to 13)	6 (5 to 7)	0.8 (0.7 to 0.8)	0.2 (0.2 to 0.2)	555 (499 to 628)	210 (184 to 233)	27.2 (24.9 to 29.7)	6.8 (6.0 to 7.5)
Exposure to forces of nature	2 (2 to 2)	0 (0 to 1)	0.1 (0.1 to 0.2)	0.0 (0.0 to 0.0)	77 (66 to 89)	25 (23 to 27)	4.2 (3.4 to 5.2)	0.8 (0.7 to 0.9)
Other unintentional injuries	32 (30 to 35)	20 (18 to 22)	1.5 (1.4 to 1.6)	0.6 (0.6 to 0.7)	1 886 (1 729 to 2 065)	975 (862 to 1 097)	78.2 (71.9 to 84.8)	31.5 (27.8 to 35.3)

Cause	Incidence				Prevalence			
	All-age counts (95% UI)		Age-standardised rates per 100,000 (95% UI)		All-age counts (95% UI)		Age-standardised rates per 100,000 (95% UI)	
	1990	2017	1990	2017	1990	2017	1990	2017
**Transport and unintentional injuries**	**82 089 (76 102 to 88 164)**	**128 455 (119 547 to 138 887)**	**4 052.0 (3 784.5 to 4 327.1)**	**4 296.0 (3 987.1 to 4 648.3)**	**161 639 (152 092 to 172 691)**	**286 637 (269 587 to 308 807)**	**9 623.3 (9 089.8 to 10 290.3)**	**9 360.5 (8 814.5 to 10 079.9)**
**Transport injuries**	**9 585 (8 292 to 11 225)**	**19 270 (16 446 to 22 322)**	**458.3 (401.5 to 525.3)**	**627.5 (535.2 to 726.7)**	**22 887 (21 273 to 24 475)**	**56 415 (52 409 to 60 480)**	**1 409.8 (1 312.0 to 1 504.9)**	**1 823.1 (1 693.4 to 1 953.8)**
Road injuries	9 089 (7 774 to 10 677)	18 902 (16 117 to 21 936)	435.0 (378.8 to 502.3)	615.3 (523.8 to 713.5)	20 705 (19 215 to 22 189)	54 544 (50 710 to 58 564)	1 283.3 (1 192.7 to 1 373.6)	1 762.1 (1 639.8 to 1 891.6)
Pedestrian road injuries	1 516 (1 223 to 1 859)	2 548 (2 060 to 3 100)	75.0 (61.8 to 89.6)	83.2 (67.4 to 101.1)	5 456 (4 642 to 6 482)	11 620 (10 096 to 13 504)	338.2 (291.5 to 396.7)	377.4 (328.4 to 437.6)
Cyclist road injuries	1 392 (1 075 to 1 777)	2 647 (2 129 to 3 278)	61.5 (49.1 to 76.6)	87.8 (70.5 to 108.6)	2 281 (1 908 to 2 704)	5 868 (4 977 to 6 848)	133.3 (112.3 to 155.2)	190.2 (161.6 to 221.6)
Motorcyclist road injuries	1 235 (985 to 1 550)	2 297 (1 780 to 2 921)	56.5 (45.7 to 69.8)	74.6 (57.7 to 95.0)	4 297 (3 721 to 4 958)	10 592 (9 106 to 12 352)	262.2 (227.5 to 302.6)	340.5 (292.8 to 396.4)
Motor vehicle road injuries	3 442 (2 843 to 4 120)	9 092 (7 585 to 10 758)	169.1 (140.7 to 199.3)	293.6 (245.2 to 348.0)	5 951 (5 219 to 6 916)	20 881 (18 167 to 24 357)	382.9 (333.1 to 441.8)	673.0 (585.3 to 784.2)
Other road injuries	1 504 (1 149 to 1 920)	2 318 (1 831 to 2 877)	72.8 (57.6 to 89.6)	76.1 (59.5 to 95.0)	2 719 (2 269 to 3 270)	5 583 (4 643 to 6 787)	166.7 (139.8 to 199.8)	181.1 (151.0 to 219.7)
Other transport injuries	496 (400 to 622)	368 (310 to 438)	23.3 (19.4 to 27.8)	12.2 (10.3 to 14.7)	2 183 (1 819 to 2 570)	1 872 (1 614 to 2 145)	126.4 (107.6 to 145.8)	60.9 (52.6 to 69.7)
**Unintentional injuries**	**72 504 (67 046 to 78 267)**	**109 185 (100 562 to 118 629)**	**3 593.7 (3 338.2 to 3 862.9)**	**3 668.5 (3 376.3 to 3 992.3)**	**138 752 (129 748 to 149 739)**	**230 221 (213 544 to 252 115)**	**8 213.5 (7 672.3 to 8 866.6)**	**7 537.4 (6 992.4 to 8 257.6)**
Falls	31 473 (27 358 to 36 011)	60 548 (53 916 to 68 553)	1 741.5 (1 552.3 to 1 969.6)	2 056.4 (1 829.6 to 2 325.4)	54 530 (48 034 to 62 342)	121 276 (107 982 to 138 245)	3 464.0 (3 102.6 to 3 949.1)	4 009.2 (3 573.8 to 4 576.3)
Drowning	169 (146 to 197)	71 (59 to 87)	7.5 (6.5 to 8.5)	2.4 (2.0 to 2.9)	766 (668 to 887)	449 (397 to 517)	46.8 (41.3 to 53.7)	14.7 (13.0 to 16.9)
Fire, heat, and hot substances	2 947 (2 326 to 3 713)	2 292 (1 885 to 2 778)	111.1 (90.4 to 136.6)	79.2 (64.8 to 96.8)	27 621 (22 758 to 34 790)	28 004 (23 195 to 35 243)	1 434.9 (1 197.9 to 1 754.9)	909.2 (753.4 to 1 145.7)
Poisonings	1 011 (761 to 1 326)	1 158 (872 to 1 496)	35.2 (26.9 to 45.4)	40.9 (30.7 to 53.5)	876 (677 to 1 082)	1 378 (1 030 to 1 700)	40.5 (31.7 to 49.7)	44.9 (33.6 to 55.4)
Poisoning by carbon monoxide	177 (115 to 267)	136 (82 to 204)	6.8 (4.7 to 9.6)	4.7 (2.8 to 7.0)	121 (91 to 158)	105 (81 to 135)	5.8 (4.5 to 7.4)	3.4 (2.6 to 4.4)
Poisoning by other means	833 (616 to 1 113)	1 022 (764 to 1 328)	28.4 (21.4 to 37.2)	36.2 (26.8 to 47.6)	755 (557 to 954)	1 273 (923 to 1 593)	34.7 (25.9 to 43.7)	41.5 (30.0 to 52.0)
Exposure to mechanical forces	17 715 (14 995 to 21 000)	20 697 (17 842 to 24 026)	788.8 (679.0 to 920.4)	678.3 (585.2 to 789.6)	34 215 (30 028 to 39 256)	53 318 (46 836 to 61 713)	2 056.1 (1 814.0 to 2 346.7)	1 719.4 (1 510.8 to 1 989.2)
Unintentional firearm injuries	1 440 (1 057 to 1 918)	1 316 (978 to 1 734)	63.4 (47.8 to 82.1)	42.6 (31.7 to 56.1)	5 085 (4 062 to 6 040)	6 436 (5 173 to 7 855)	313.5 (253.1 to 373.8)	207.2 (166.8 to 252.7)
Other exposure to mechanical forces	16 275 (13 770 to 19 319)	19 381 (16 758 to 22 546)	725.4 (624.1 to 847.0)	635.6 (547.2 to 742.6)	29 130 (24 613 to 34 383)	46 882 (40 232 to 55 557)	1 742.7 (1 492.3 to 2 044.6)	1 512.2 (1 298.3 to 1 789.8)
Adverse effects of medical treatment	1 632 (1 362 to 1 929)	2 980 (2 528 to 3 462)	89.8 (76.5 to 103.6)	97.3 (82.5 to 113.1)	125 (95 to 156)	228 (175 to 284)	6.9 (5.3 to 8.5)	7.4 (5.7 to 9.3)
Animal contact	7 189 (6 073 to 8 561)	8 087 (6 992 to 9 275)	325.2 (282.3 to 374.7)	270.8 (235.4 to 314.3)	7 438 (6 393 to 8 623)	10 509 (9 208 to 11 993)	422.5 (370.7 to 478.9)	342.7 (300.1 to 391.5)
Venomous animal contact	2 888 (2 250 to 3 658)	3 284 (2 639 to 4 033)	130.7 (104.2 to 161.3)	110.4 (88.4 to 136.3)	2 004 (1 639 to 2 427)	2 796 (2 330 to 3 295)	107.7 (90.3 to 127.0)	91.1 (75.9 to 107.2)
Non-venomous animal contact	4 301 (3 497 to 5 280)	4 753 (3 924 to 5 755)	194.5 (162.2 to 234.9)	160.3 (131.5 to 194.0)	5 433 (4 472 to 6 552)	7 712 (6 442 to 9 152)	314.8 (264.7 to 369.3)	251.6 (209.9 to 297.8)
Foreign body	4 840 (3 072 to 6 926)	8 473 (4 649 to 13 049)	238.3 (145.3 to 340.1)	280.1 (155.8 to 426.4)	2 832 (2 262 to 3 570)	3 793 (2 955 to 4 904)	152.2 (124.8 to 186.4)	125.0 (97.7 to 161.8)
Pulmonary aspiration and foreign body in airway	220 (154 to 309)	94 (70 to 122)	8.0 (5.8 to 11.0)	3.3 (2.4 to 4.3)	157 (109 to 216)	86 (69 to 108)	7.4 (5.2 to 10.0)	2.8 (2.3 to 3.5)
Foreign body in eyes	4 012 (2 286 to 6 113)	7 866 (4 024 to 12 520)	205.6 (113.6 to 307.7)	259.3 (134.3 to 406.4)	776 (404 to 1 323)	1 765 (1 022 to 2 766)	46.1 (26.9 to 72.5)	58.8 (34.1 to 91.0)
Foreign body in other body part	608 (441 to 860)	514 (406 to 643)	24.7 (18.6 to 33.0)	17.5 (13.7 to 22.1)	1 899 (1 517 to 2 409)	1 941 (1 620 to 2 380)	98.7 (80.3 to 123.1)	63.3 (52.8 to 77.7)
Environmental heat and cold exposure	1 025 (725 to 1 395)	479 (359 to 636)	48.2 (37.0 to 60.7)	16.3 (12.2 to 21.7)	3 545 (2 864 to 4 331)	1 890 (1 598 to 2 262)	200.0 (166.3 to 238.8)	62.1 (52.5 to 74.2)
Exposure to forces of nature	192 (158 to 233)	33 (28 to 39)	10.1 (8.2 to 12.4)	1.1 (0.9 to 1.3)	690 (487 to 945)	1 313 (922 to 1 822)	30.8 (22.1 to 41.8)	41.6 (29.3 to 57.6)
Other unintentional injuries	4 311 (3 623 to 5 216)	4 416 (3 804 to 5 121)	198.2 (170.0 to 233.1)	145.9 (125.4 to 168.2)	6 115 (5 250 to 7 090)	8 065 (7 009 to 9 238)	358.9 (310.6 to 411.7)	261.2 (227.6 to 298.4)

Cause	Years lived with disability				Disability adjusted life years			
	All-age counts (95% UI)		Age-standardised rates per 100,000 (95% UI)		All-age counts (95% UI)		Age-standardised rates per 100,000 (95% UI)	
	1990	2017	1990	2017	1990	2017	1990	2017
**Transport and unintentional injuries**	**6 977 (5 077 to 9 287)**	**12 083 (8 720 to 16 428)**	**411.5 (298.8 to 544.4)**	**395.1 (285.3 to 537.4)**	**82 321 (75 383 to 90 332)**	**56 256 (51 522 to 61 174)**	**3 581.0 (3 322.4 to 3 860.1)**	**1 843.1 (1 687.9 to 2 006.1)**
**Transport injuries**	**1 531 (1 111 to 2 032)**	**3 358 (2 432 to 4 485)**	**91.0 (66.4 to 120.5)**	**108.2 (78.3 to 144.6)**	**40 775 (37 552 to 44 605)**	**30 716 (28 191 to 33 484)**	**1 768.8 (1 657.1 to 1 906.0)**	**994.0 (911.5 to 1 082.6)**
Road injuries	1 397 (1 018 to 1 848)	3 263 (2 356 to 4 374)	83.6 (61.1 to 110.7)	105.1 (75.9 to 140.8)	39 054 (35 898 to 42 792)	28 814 (26 418 to 31 421)	1 692.0 (1 582.8 to 1 825.7)	932.7 (855.3 to 1 017.2)
Pedestrian road injuries	385 (271 to 521)	723 (510 to 988)	22.9 (16.4 to 30.8)	23.4 (16.6 to 32.0)	18 792 (16 790 to 21 345)	11 681 (10 669 to 12 748)	809.4 (740.9 to 895.2)	381.6 (347.9 to 415.6)
Cyclist road injuries	154 (108 to 203)	342 (239 to 456)	8.7 (6.2 to 11.5)	11.1 (7.8 to 14.7)	1 101 (981 to 1 225)	1 329 (1 179 to 1 500)	50.2 (45.0 to 55.3)	43.0 (38.1 to 48.6)
Motorcyclist road injuries	292 (205 to 395)	634 (436 to 870)	17.3 (12.1 to 23.5)	20.3 (14.0 to 27.9)	5 452 (4 905 to 6 050)	4 182 (3 675 to 4 758)	235.4 (213.9 to 258.6)	133.9 (117.7 to 152.4)
Motor vehicle road injuries	416 (299 to 551)	1 292 (929 to 1 738)	25.7 (18.6 to 34.0)	41.5 (29.8 to 55.8)	12 474 (11 443 to 13 684)	10 596 (9 455 to 11 833)	542.2 (498.7 to 590.8)	341.1 (304.2 to 380.5)
Other road injuries	150 (106 to 203)	271 (193 to 374)	8.9 (6.5 to 12.0)	8.8 (6.2 to 12.1)	1 236 (1 048 to 1 420)	1 027 (888 to 1 170)	54.9 (47.4 to 62.1)	33.2 (28.7 to 37.8)
Other transport injuries	134 (95 to 177)	96 (69 to 129)	7.4 (5.3 to 9.8)	3.1 (2.2 to 4.2)	1 721 (1 550 to 1 913)	1 902 (1 680 to 2 157)	76.7 (69.9 to 84.3)	61.3 (54.2 to 69.5)
**Unintentional injuries**	**5 446 (3 940 to 7 267)**	**8 725 (6 190 to 11 929)**	**320.5 (230.5 to 428.5)**	**286.9 (203.6 to 392.3)**	**41 546 (37 043 to 46 311)**	**25 541 (22 632 to 28 741)**	**1 812.2 (1 661.1 to 1 993.3)**	**849.1 (753.1 to 954.3)**
Falls	3 137 (2 311 to 4 123)	6 025 (4 316 to 8 107)	194.5 (144.1 to 253.7)	199.3 (142.8 to 267.8)	6 942 (6 050 to 7 954)	9 159 (7 429 to 11 212)	409.4 (356.7 to 469.7)	304.1 (246.8 to 371.8)
Drowning	43 (30 to 60)	23 (16 to 33)	2.6 (1.8 to 3.5)	0.8 (0.5 to 1.1)	13 424 (11 597 to 15 634)	4 648 (4 199 to 5 149)	512.2 (451.8 to 582.9)	154.5 (139.3 to 171.7)
Fire, heat, and hot substances	583 (364 to 916)	532 (309 to 859)	29.4 (18.1 to 46.8)	17.3 (10.1 to 27.9)	3 089 (2 617 to 3 651)	1 407 (1 166 to 1 746)	133.5 (115.8 to 155.6)	46.7 (38.9 to 57.6)
Poisonings	103 (64 to 155)	156 (94 to 237)	4.6 (2.9 to 6.8)	5.1 (3.1 to 7.8)	1 591 (1 386 to 1 874)	741 (653 to 827)	62.5 (55.5 to 71.9)	24.5 (21.5 to 27.3)
Poisoning by carbon monoxide	13 (8 to 20)	10 (6 to 16)	0.6 (0.4 to 0.9)	0.3 (0.2 to 0.5)	358 (307 to 424)	177 (154 to 199)	14.2 (12.5 to 16.4)	5.8 (5.1 to 6.6)
Poisoning by other means	90 (56 to 139)	146 (88 to 223)	4.0 (2.5 to 6.1)	4.8 (2.9 to 7.3)	1 233 (1 067 to 1 458)	564 (489 to 647)	48.3 (42.5 to 55.9)	18.7 (16.2 to 21.3)
Exposure to mechanical forces	796 (547 to 1 132)	1 135 (767 to 1 663)	47.0 (32.1 to 67.6)	36.6 (24.8 to 53.6)	5 036 (4 601 to 5 578)	3 437 (2 978 to 3 975)	231.0 (211.6 to 256.1)	111.1 (96.5 to 128.4)
Unintentional firearm injuries	106 (70 to 159)	121 (78 to 190)	6.3 (4.1 to 9.6)	3.9 (2.5 to 6.1)	2 426 (2 094 to 2 671)	1 379 (1 068 to 1 583)	105.1 (91.6 to 114.6)	44.3 (34.3 to 50.8)
Other exposure to mechanical forces	690 (474 to 985)	1 014 (684 to 1 500)	40.7 (27.9 to 58.4)	32.7 (22.1 to 48.4)	2 610 (2 310 to 2 982)	2 058 (1 674 to 2 563)	126.0 (111.1 to 146.4)	66.7 (54.4 to 82.9)
Adverse effects of medical treatment	17 (10 to 26)	30 (19 to 46)	0.9 (0.6 to 1.4)	1.0 (0.6 to 1.5)	1 485 (1 271 to 1 742)	1 035 (893 to 1 506)	68.0 (60.1 to 78.1)	35.2 (30.2 to 51.4)
Animal contact	199 (134 to 285)	245 (163 to 353)	10.2 (6.9 to 14.6)	8.0 (5.3 to 11.5)	1 759 (1 425 to 2 196)	606 (519 to 719)	67.5 (56.0 to 82.0)	20.3 (17.3 to 24.0)
Venomous animal contact	118 (77 to 171)	140 (90 to 202)	5.6 (3.7 to 8.0)	4.6 (2.9 to 6.6)	1 544 (1 208 to 1 971)	435 (360 to 510)	57.3 (46.0 to 71.2)	14.7 (12.3 to 17.2)
Non-venomous animal contact	81 (49 to 133)	105 (61 to 176)	4.7 (2.8 to 7.5)	3.4 (2.0 to 5.7)	215 (169 to 316)	170 (122 to 248)	10.1 (7.9 to 14.6)	5.6 (4.0 to 8.1)
Foreign body	169 (115 to 239)	207 (138 to 310)	8.9 (6.1 to 12.3)	6.8 (4.4 to 10.2)	5 305 (4 491 to 6 276)	2 927 (2 552 to 3 402)	196.1 (169.7 to 227.7)	101.4 (87.7 to 118.8)
Pulmonary aspiration and foreign body in airway	8 (5 to 11)	4 (2 to 5)	0.4 (0.2 to 0.5)	0.1 (0.1 to 0.2)	4 855 (4 088 to 5 780)	2 629 (2 269 to 3 058)	176.7 (151.9 to 206.2)	91.6 (78.2 to 108.0)
Foreign body in eyes	52 (26 to 92)	118 (56 to 198)	3.1 (1.7 to 5.1)	3.9 (2.2 to 6.5)	52 (26 to 92)	118 (56 to 198)	3.1 (1.7 to 5.1)	3.9 (2.2 to 6.5)
Foreign body in other body part	109 (76 to 146)	86 (60 to 114)	5.4 (3.9 to 7.2)	2.8 (2.0 to 3.7)	398 (285 to 473)	180 (147 to 225)	16.4 (12.2 to 19.3)	5.9 (4.8 to 7.4)
Environmental heat and cold exposure	137 (90 to 194)	71 (46 to 102)	7.7 (5.2 to 10.9)	2.3 (1.5 to 3.4)	692 (609 to 784)	281 (244 to 321)	34.9 (31.2 to 39.1)	9.2 (8.0 to 10.5)
Exposure to forces of nature	30 (21 to 41)	40 (29 to 55)	1.4 (1.0 to 1.9)	1.3 (0.9 to 1.7)	106 (92 to 123)	64 (53 to 80)	5.6 (4.7 to 6.7)	2.1 (1.7 to 2.6)
Other unintentional injuries	232 (168 to 314)	261 (188 to 352)	13.3 (9.7 to 17.9)	8.4 (5.9 to 11.7)	2 118 (1 947 to 2 318)	1 236 (1 088 to 1 389)	91.5 (84.6 to 100.2)	39.9 (35.1 to 44.8)

Death, YLL, incidence, prevalence, YLD, and DALY counts and rates per 100,000 for all injuries in Sonora in 1990 and 2017

Cause	Deaths: All-age counts (95% UI) 1990	Deaths: All-age counts (95% UI) 2017	Deaths: Age-standardised rates per 100,000 (95% UI) 1990	Deaths: Age-standardised rates per 100,000 (95% UI) 2017
**Transport and unintentional injuries**	**1 121 (1 068 to 1 181)**	**1 191 (1 111 to 1 271)**	**75.0 (72.5 to 77.8)**	**41.6 (38.9 to 44.2)**
**Transport injuries**	**578 (548 to 612)**	**652 (601 to 705)**	**34.9 (33.4 to 36.5)**	**21.6 (20.0 to 23.3)**
Road injuries	553 (524 to 587)	604 (558 to 652)	33.3 (31.8 to 34.8)	20.1 (18.6 to 21.6)
Pedestrian road injuries	216 (200 to 235)	242 (219 to 265)	14.6 (13.7 to 15.5)	8.3 (7.6 to 9.1)
Cyclist road injuries	17 (16 to 19)	32 (29 to 36)	1.0 (1.0 to 1.1)	1.1 (1.0 to 1.2)
Motorcyclist road injuries	83 (75 to 93)	76 (67 to 86)	4.4 (4.1 to 4.9)	2.4 (2.1 to 2.7)
Motor vehicle road injuries	220 (204 to 239)	234 (213 to 255)	12.2 (11.3 to 13.1)	7.6 (6.9 to 8.3)
Other road injuries	16 (14 to 19)	19 (17 to 22)	1.0 (0.9 to 1.2)	0.6 (0.6 to 0.7)
Other transport injuries	25 (23 to 28)	48 (42 to 54)	1.6 (1.5 to 1.8)	1.6 (1.4 to 1.8)
**Unintentional injuries**	**543 (513 to 579)**	**539 (505 to 572)**	**40.1 (38.5 to 41.7)**	**19.9 (18.7 to 21.1)**
Falls	94 (90 to 99)	146 (134 to 157)	10.7 (10.2 to 11.3)	6.0 (5.5 to 6.5)
Drowning	140 (128 to 155)	98 (89 to 107)	7.8 (7.3 to 8.5)	3.3 (3.0 to 3.6)
Fire, heat, and hot substances	48 (44 to 53)	42 (39 to 45)	4.0 (3.8 to 4.2)	1.6 (1.5 to 1.7)
Poisonings	27 (25 to 30)	22 (20 to 24)	1.6 (1.5 to 1.7)	0.8 (0.7 to 0.8)
Poisoning by carbon monoxide	14 (13 to 16)	13 (12 to 15)	0.9 (0.8 to 0.9)	0.5 (0.4 to 0.5)
Poisoning by other means	13 (11 to 14)	9 (8 to 10)	0.7 (0.7 to 0.8)	0.3 (0.3 to 0.3)
Exposure to mechanical forces	68 (63 to 72)	52 (47 to 57)	4.3 (4.1 to 4.5)	1.7 (1.6 to 1.9)
Unintentional firearm injuries	34 (31 to 37)	20 (16 to 23)	2.0 (1.8 to 2.2)	0.7 (0.5 to 0.7)
Other exposure to mechanical forces	34 (30 to 36)	32 (29 to 36)	2.3 (2.1 to 2.5)	1.1 (1.0 to 1.2)
Adverse effects of medical treatment	31 (25 to 34)	47 (42 to 54)	2.9 (2.3 to 3.2)	1.9 (1.7 to 2.1)
Animal contact	13 (11 to 16)	8 (8 to 9)	0.8 (0.7 to 0.9)	0.3 (0.3 to 0.3)
Venomous animal contact	11 (9 to 13)	6 (5 to 6)	0.7 (0.6 to 0.8)	0.2 (0.2 to 0.2)
Non-venomous animal contact	2 (2 to 3)	2 (2 to 3)	0.2 (0.1 to 0.2)	0.1 (0.1 to 0.1)
Foreign body	72 (64 to 81)	79 (71 to 89)	4.3 (3.9 to 4.7)	2.9 (2.6 to 3.2)
Pulmonary aspiration and foreign body in airway	69 (61 to 78)	77 (69 to 86)	4.1 (3.8 to 4.5)	2.8 (2.5 to 3.1)
Foreign body in eyes				
Foreign body in other body part	3 (2 to 4)	2 (2 to 3)	0.2 (0.1 to 0.2)	0.1 (0.1 to 0.1)
Environmental heat and cold exposure	23 (21 to 24)	18 (16 to 21)	2.0 (1.8 to 2.1)	0.6 (0.6 to 0.7)
Exposure to forces of nature	1 (1 to 1)	0 (0 to 1)	0.1 (0.0 to 0.1)	0.0 (0.0 to 0.0)
Other unintentional injuries	27 (25 to 29)	26 (24 to 29)	1.6 (1.5 to 1.7)	0.9 (0.8 to 1.0)

Cause	Years of life lost: All-age counts (95% UI) 1990	Years of life lost: All-age counts (95% UI) 2017	Years of life lost: Age-standardised rates per 100,000 (95% UI) 1990	Years of life lost: Age-standardised rates per 100,000 (95% UI) 2017
**Transport and unintentional injuries**	**60 352 (56 238 to 65 132)**	**51 649 (47 922 to 55 361)**	**3 161.6 (2 988.2 to 3 364.3)**	**1 701.9 (1 578.5 to 1 825.0)**
**Transport injuries**	**32 031 (29 934 to 34 511)**	**30 686 (28 154 to 33 166)**	**1 651.5 (1 557.9 to 1 760.2)**	**989.3 (908.3 to 1 068.5)**
Road injuries	30 726 (28 693 to 33 176)	28 465 (26 185 to 30 830)	1 580.8 (1 489.5 to 1 686.9)	918.3 (845.0 to 993.1)
Pedestrian road injuries	11 513 (10 802 to 12 922)	10 287 (9 246 to 11 309)	613.3 (561.9 to 676.3)	337.5 (303.9 to 370.1)
Cyclist road injuries	932 (840 to 1 036)	1 401 (1 241 to 1 570)	49.4 (44.9 to 54.6)	45.4 (40.3 to 50.8)
Motorcyclist road injuries	4 816 (4 337 to 5 353)	4 128 (3 638 to 4 655)	240.2 (218.2 to 264.4)	130.4 (114.3 to 147.0)
Motor vehicle road injuries	12 580 (11 518 to 13 739)	11 746 (10 669 to 12 828)	631.8 (583.3 to 688.0)	375.8 (341.3 to 410.4)
Other road injuries	885 (747 to 1 026)	903 (778 to 1 048)	46.0 (39.8 to 52.7)	29.1 (25.2 to 33.7)
Other transport injuries	1 306 (1 167 to 1 443)	2 222 (1 941 to 2 551)	70.7 (64.0 to 77.7)	71.2 (62.3 to 81.5)
**Unintentional injuries**	**28 321 (25 871 to 31 372)**	**20 963 (19 418 to 22 652)**	**1 510.1 (1 406.1 to 1 634.7)**	**712.6 (660.1 to 771.1)**
Falls	2 935 (2 752 to 3 139)	3 517 (3 211 to 3 803)	216.9 (206.5 to 229.1)	124.8 (114.1 to 135.0)
Drowning	8 500 (7 574 to 9 727)	4 842 (4 389 to 5 370)	421.3 (371.8 to 462.0)	159.5 (144.4 to 177.1)
Fire, heat, and hot substances	2 388 (2 077 to 2 804)	1 404 (1 276 to 1 536)	131.4 (118.4 to 148.9)	48.4 (44.1 to 52.9)
Poisonings	1 609 (1 422 to 1 826)	1 040 (941 to 1 142)	79.6 (71.6 to 88.8)	34.5 (31.3 to 37.8)
Poisoning by carbon monoxide	845 (748 to 962)	615 (553 to 684)	42.1 (37.8 to 46.8)	20.3 (18.2 to 22.6)
Poisoning by other means	765 (668 to 890)	425 (379 to 477)	37.6 (33.5 to 42.7)	14.2 (12.6 to 16.0)
Exposure to mechanical forces	3 583 (3 314 to 3 870)	2 263 (2 051 to 2 501)	190.9 (178.5 to 204.2)	73.2 (66.5 to 80.6)
Unintentional firearm injuries	1 891 (1 696 to 2 099)	955 (766 to 1 099)	96.8 (87.8 to 106.6)	30.5 (24.5 to 34.9)
Other exposure to mechanical forces	1 692 (1 474 to 1 862)	1 307 (1 165 to 1 498)	94.1 (83.0 to 102.5)	42.7 (38.1 to 48.7)
Adverse effects of medical treatment	1 274 (1 006 to 1 463)	1 416 (1 261 to 1 726)	77.8 (62.1 to 87.0)	50.3 (44.8 to 61.2)
Animal contact	840 (684 to 1 055)	335 (301 to 374)	40.5 (34.0 to 49.7)	11.6 (10.4 to 13.0)
Venomous animal contact	713 (562 to 912)	238 (205 to 273)	33.7 (26.9 to 42.3)	8.3 (7.2 to 9.6)
Non-venomous animal contact	127 (105 to 164)	98 (83 to 133)	6.9 (5.8 to 8.8)	3.3 (2.8 to 4.5)
Foreign body	4 683 (3 979 to 5 441)	4 163 (3 619 to 4 882)	220.9 (192.2 to 251.8)	146.4 (126.4 to 173.2)
Pulmonary aspiration and foreign body in airway	4 484 (3 810 to 5 224)	4 050 (3 521 to 4 749)	211.3 (183.5 to 241.8)	142.7 (123.0 to 169.0)
Foreign body in eyes				
Foreign body in other body part	198 (148 to 240)	113 (94 to 175)	9.5 (7.2 to 11.4)	3.7 (3.1 to 5.8)
Environmental heat and cold exposure	945 (858 to 1 044)	665 (587 to 745)	60.4 (55.4 to 65.6)	21.8 (19.3 to 24.3)
Exposure to forces of nature	54 (49 to 60)	25 (22 to 27)	2.6 (2.4 to 2.9)	0.8 (0.7 to 0.9)
Other unintentional injuries	1 510 (1 385 to 1 659)	1 294 (1 142 to 1 451)	77.6 (71.7 to 84.3)	41.3 (36.6 to 46.2)

Cause	Incidence: All-age counts (95% UI) 1990	Incidence: All-age counts (95% UI) 2017	Incidence: Age-standardised rates per 100,000 (95% UI) 1990	Incidence: Age-standardised rates per 100,000 (95% UI) 2017
**Transport and unintentional injuries**	**67 265 (61 408 to 74 015)**	**120 816 (109 481 to 133 848)**	**4 143.0 (3 783.5 to 4 551.8)**	**4 205.7 (3 816.0 to 4 637.4)**
**Transport injuries**	**6 945 (5 969 to 8 066)**	**15 228 (13 147 to 17 547)**	**398.1 (347.2 to 454.6)**	**498.5 (431.0 to 572.3)**
Road injuries	6 528 (5 574 to 7 640)	14 806 (12 732 to 17 135)	373.8 (321.8 to 429.1)	484.1 (416.3 to 557.4)
Pedestrian road injuries	991 (797 to 1 216)	1 896 (1 529 to 2 329)	59.5 (48.9 to 71.5)	62.7 (50.7 to 76.3)
Cyclist road injuries	932 (748 to 1 169)	1 933 (1 520 to 2 441)	53.3 (42.5 to 67.6)	64.6 (51.0 to 81.5)
Motorcyclist road injuries	956 (759 to 1 200)	1 411 (1 076 to 1 796)	52.0 (42.0 to 63.8)	45.7 (34.9 to 58.1)
Motor vehicle road injuries	2 504 (2 049 to 3 012)	7 359 (6 107 to 8 730)	144.0 (118.1 to 171.2)	237.7 (198.1 to 282.0)
Other road injuries	1 094 (823 to 1 389)	2 207 (1 722 to 2 725)	64.5 (51.0 to 78.7)	73.3 (57.3 to 89.7)
Other transport injuries	418 (337 to 533)	423 (350 to 519)	24.5 (20.5 to 29.5)	14.4 (11.9 to 17.7)
**Unintentional injuries**	**60 320 (54 733 to 67 081)**	**105 588 (94 799 to 118 592)**	**3 744.7 (3 383.3 to 4 158.6)**	**3 707.2 (3 332.1 to 4 145.7)**
Falls	24 614 (21 662 to 27 869)	58 972 (52 156 to 66 663)	1 709.7 (1 522.7 to 1 915.8)	2 138.4 (1 876.2 to 2 407.0)
Drowning	119 (104 to 137)	77 (62 to 97)	6.6 (5.8 to 7.5)	2.6 (2.1 to 3.3)
Fire, heat, and hot substances	2 592 (2 094 to 3 207)	2 791 (2 311 to 3 364)	124.1 (102.4 to 150.7)	96.6 (79.9 to 117.0)
Poisonings	809 (597 to 1 108)	803 (553 to 1 121)	35.7 (27.1 to 48.0)	28.5 (19.5 to 40.0)
Poisoning by carbon monoxide	162 (107 to 241)	153 (85 to 249)	7.9 (5.5 to 11.2)	5.3 (3.0 to 8.7)
Poisoning by other means	641 (471 to 901)	650 (434 to 948)	27.8 (20.6 to 38.3)	23.2 (15.4 to 34.0)
Exposure to mechanical forces	14 831 (12 471 to 17 654)	19 114 (16 324 to 22 273)	795.7 (679.4 to 925.5)	628.8 (539.0 to 728.6)
Unintentional firearm injuries	1 194 (878 to 1 613)	927 (680 to 1 223)	62.0 (46.7 to 81.1)	30.1 (22.3 to 39.5)
Other exposure to mechanical forces	13 637 (11 489 to 16 225)	18 187 (15 606 to 21 228)	733.7 (624.8 to 855.8)	598.7 (513.4 to 698.1)
Adverse effects of medical treatment	1 399 (1 171 to 1 636)	2 847 (2 412 to 3 310)	93.7 (79.6 to 108.8)	96.5 (82.0 to 111.9)
Animal contact	4 519 (3 667 to 5 567)	4 633 (3 964 to 5 448)	255.7 (214.8 to 304.0)	159.4 (136.5 to 187.6)
Venomous animal contact	1 661 (1 241 to 2 200)	1 696 (1 335 to 2 134)	93.6 (71.5 to 118.7)	58.0 (45.6 to 72.6)
Non-venomous animal contact	2 859 (2 236 to 3 641)	2 937 (2 425 to 3 597)	162.1 (131.1 to 200.6)	101.4 (83.4 to 124.1)
Foreign body	6 883 (3 582 to 11 641)	12 311 (5 176 to 22 215)	456.6 (307.9 to 771.7)	417.9 (179.1 to 754.2)
Pulmonary aspiration and foreign body in airway	193 (137 to 264)	102 (75 to 136)	8.9 (6.6 to 12.2)	3.6 (2.7 to 4.9)
Foreign body in eyes	6 248 (2 904 to 11 033)	11 681 (4 494 to 21 545)	425.1 (175.1 to 747.7)	395.8 (153.6 to 733.1)
Foreign body in other body part	444 (309 to 642)	528 (376 to 703)	22.7 (16.3 to 31.1)	18.5 (13.0 to 24.4)
Environmental heat and cold exposure	982 (764 to 1 236)	590 (475 to 705)	62.5 (50.4 to 75.3)	20.9 (16.9 to 24.7)
Exposure to forces of nature	141 (115 to 176)	33 (28 to 39)	6.8 (5.7 to 8.2)	1.1 (0.9 to 1.3)
Other unintentional injuries	3 437 (2 835 to 4 202)	3 416 (2 858 to 4 106)	197.5 (166.4 to 235.8)	116.5 (97.9 to 139.4)

Cause	Prevalence: All-age counts (95% UI) 1990	Prevalence: All-age counts (95% UI) 2017	Prevalence: Age-standardised rates per 100,000 (95% UI) 1990	Prevalence: Age-standardised rates per 100,000 (95% UI) 2017
**Transport and unintentional injuries**	**132 937 (124 975 to 142 081)**	**264 754 (247 519 to 286 469)**	**9 487.1 (8 957.4 to 10 163.0)**	**9 071.6 (8 497.7 to 9 821.2)**
**Transport injuries**	**17 077 (15 889 to 18 211)**	**43 571 (40 404 to 46 995)**	**1 237.1 (1 153.3 to 1 321.4)**	**1 459.5 (1 354.3 to 1 573.2)**
Road injuries	15 204 (14 083 to 16 296)	41 449 (38 402 to 44 828)	1 106.4 (1 027.8 to 1 186.1)	1 387.2 (1 285.1 to 1 497.3)
Pedestrian road injuries	3 701 (3 152 to 4 352)	8 684 (7 505 to 10 122)	271.8 (235.0 to 317.8)	294.1 (255.0 to 342.4)
Cyclist road injuries	1 669 (1 394 to 1 969)	4 235 (3 580 to 4 980)	116.1 (97.7 to 134.9)	142.3 (121.0 to 165.9)
Motorcyclist road injuries	3 405 (2 934 to 3 959)	6 477 (5 545 to 7 598)	242.3 (209.3 to 281.6)	219.7 (188.6 to 249.8)
Motor vehicle road injuries	4 403 (3 826 to 5 133)	16 810 (14 725 to 19 696)	328.8 (284.5 to 382.6)	560.3 (489.9 to 656.1)
Other road injuries	2 026 (1 687 to 2 446)	5 243 (4 366 to 6 356)	147.4 (123.4 to 176.3)	176.8 (148.0 to 212.7)
Other transport injuries	1 873 (1 557 to 2 197)	2 122 (1 805 to 2 475)	130.7 (111.4 to 150.2)	72.3 (61.9 to 83.8)
**Unintentional injuries**	**115 860 (108 242 to 124 648)**	**221 183 (204 827 to 242 093)**	**8 250.0 (7 704.1 to 8 899.9)**	**7 612.4 (7 044.6 to 8 322.0)**
Falls	43 399 (38 099 to 49 509)	117 660 (104 317 to 134 339)	3 360.0 (3 005.7 to 3 858.7)	4 159.0 (3 703.8 to 4 746.0)
Drowning	559 (489 to 644)	474 (415 to 550)	41.3 (36.5 to 47.3)	16.4 (14.4 to 18.9)
Fire, heat, and hot substances	25 490 (21 133 to 32 098)	33 729 (27 998 to 42 789)	1 592.4 (1 339.9 to 1 957.5)	1 118.2 (932.3 to 1 410.3)
Poisonings	729 (549 to 906)	994 (718 to 1 262)	40.6 (31.0 to 50.1)	32.6 (23.5 to 41.4)
Poisoning by carbon monoxide	113 (88 to 146)	124 (95 to 159)	6.5 (5.1 to 8.3)	4.1 (3.1 to 5.2)
Poisoning by other means	615 (443 to 789)	870 (607 to 1 131)	34.0 (24.8 to 43.4)	28.6 (19.9 to 37.1)
Exposure to mechanical forces	29 187 (25 569 to 33 561)	48 182 (41 846 to 56 023)	2 070.0 (1 824.3 to 2 355.1)	1 601.3 (1 392.1 to 1 858.4)
Unintentional firearm injuries	4 272 (3 419 to 5 152)	4 641 (3 744 to 5 660)	308.0 (248.7 to 372.8)	154.6 (125.1 to 188.4)
Other exposure to mechanical forces	24 915 (21 114 to 29 471)	43 541 (37 138 to 51 694)	1 762.1 (1 506.4 to 2 064.7)	1 446.5 (1 236.7 to 1 711.8)
Adverse effects of medical treatment	107 (82 to 134)	218 (167 to 272)	7.2 (5.5 to 8.9)	7.4 (5.7 to 9.2)
Animal contact	4 825 (4 158 to 5 597)	5 935 (5 159 to 6 808)	331.7 (291.8 to 377.3)	201.3 (175.5 to 230.1)
Venomous animal contact	1 117 (816 to 1 332)	1 359 (1 142 to 1 596)	72.0 (60.3 to 84.1)	45.4 (38.2 to 53.1)
Non-venomous animal contact	3 708 (3 079 to 4 419)	4 576 (3 836 to 5 436)	259.7 (219.2 to 304.7)	155.7 (131.0 to 183.9)
Foreign body	2 818 (2 120 to 3 867)	4 689 (3 453 to 6 714)	194.8 (148.6 to 265.3)	161.9 (120.2 to 227.1)
Pulmonary aspiration and foreign body in airway	141 (100 to 193)	92 (79 to 115)	8.1 (5.9 to 10.8)	3.1 (2.5 to 3.9)
Foreign body in eyes	1 282 (625 to 2 207)	2 528 (1 375 to 4 310)	96.4 (54.0 to 160.5)	89.6 (49.9 to 150.7)
Foreign body in other body part	1 444 (1 154 to 1 847)	2 069 (1 687 to 2 600)	90.3 (73.8 to 112.6)	69.2 (56.7 to 86.8)
Environmental heat and cold exposure	3 346 (2 760 to 4 001)	2 100 (1 841 to 2 461)	237.7 (202.0 to 277.8)	73.6 (64.6 to 85.5)
Exposure to forces of nature	394 (283 to 533)	1 107 (773 to 1 531)	21.3 (15.7 to 28.6)	35.5 (24.8 to 49.0)
Other unintentional injuries	5 004 (4 288 to 5 815)	6 096 (5 273 to 6 976)	352.9 (305.8 to 404.2)	205.6 (178.5 to 234.3)

Cause	Years lived with disability: All-age counts (95% UI) 1990	Years lived with disability: All-age counts (95% UI) 2017	Years lived with disability: Age-standardised rates per 100,000 (95% UI) 1990	Years lived with disability: Age-standardised rates per 100,000 (95% UI) 2017
**Transport and unintentional injuries**	**5 635 (4 141 to 7 536)**	**11 055 (7 950 to 14 982)**	**398.2 (288.8 to 532.9)**	**379.6 (272.7 to 512.5)**
**Transport injuries**	**1 143 (831 to 1 513)**	**2 606 (1 881 to 3 462)**	**79.4 (58.1 to 105.3)**	**86.3 (62.3 to 114.6)**
Road injuries	1 028 (753 to 1 370)	2 496 (1 803 to 3 322)	71.7 (52.5 to 95.8)	82.6 (59.7 to 110.0)
Pedestrian road injuries	262 (185 to 356)	546 (390 to 746)	18.4 (13.1 to 24.7)	18.3 (13.0 to 24.9)
Cyclist road injuries	113 (80 to 150)	250 (175 to 332)	7.6 (5.3 to 10.0)	8.3 (5.8 to 11.0)
Motorcyclist road injuries	232 (162 to 316)	392 (269 to 535)	15.9 (11.1 to 21.8)	12.8 (8.8 to 17.5)
Motor vehicle road injuries	309 (222 to 409)	1 048 (755 to 1 403)	22.0 (16.0 to 29.1)	34.5 (24.9 to 46.3)
Other road injuries	112 (79 to 153)	260 (184 to 361)	7.8 (5.6 to 10.7)	8.7 (6.2 to 12.0)
Other transport injuries	115 (82 to 154)	110 (78 to 147)	7.7 (5.5 to 10.2)	3.7 (2.6 to 5.0)
**Unintentional injuries**	**4 492 (3 269 to 6 013)**	**8 449 (6 019 to 11 461)**	**318.8 (230.5 to 429.1)**	**293.3 (208.7 to 396.9)**
Falls	2 506 (1 833 to 3 304)	5 889 (4 221 to 7 886)	188.2 (137.7 to 245.5)	207.8 (148.6 to 278.4)
Drowning	31 (22 to 43)	25 (17 to 35)	2.2 (1.6 to 3.0)	0.8 (0.6 to 1.2)
Fire, heat, and hot substances	538 (331 to 847)	645 (381 to 1 048)	32.6 (19.9 to 51.4)	21.3 (12.6 to 34.6)
Poisonings	85 (52 to 130)	115 (67 to 173)	4.6 (2.9 to 7.0)	3.8 (2.2 to 5.7)
Poisoning by carbon monoxide	12 (7 to 19)	12 (7 to 19)	0.7 (0.4 to 1.0)	0.4 (0.2 to 0.6)
Poisoning by other means	73 (44 to 114)	102 (58 to 156)	3.9 (2.4 to 6.1)	3.4 (1.9 to 5.1)
Exposure to mechanical forces	679 (465 to 973)	1 026 (688 to 1 513)	47.1 (32.2 to 67.7)	34.0 (22.9 to 50.0)
Unintentional firearm injuries	88 (58 to 134)	85 (54 to 137)	6.1 (4.0 to 9.5)	2.8 (1.8 to 4.5)
Other exposure to mechanical forces	590 (405 to 850)	941 (630 to 1 383)	41.0 (28.0 to 59.3)	31.2 (21.0 to 45.8)
Adverse effects of medical treatment	14 (9 to 22)	29 (18 to 44)	1.0 (0.6 to 1.4)	1.0 (0.6 to 1.5)
Animal contact	125 (84 to 176)	140 (92 to 200)	7.8 (5.3 to 11.0)	4.7 (3.1 to 6.6)
Venomous animal contact	69 (44 to 101)	76 (49 to 111)	3.9 (2.6 to 5.7)	2.5 (1.6 to 3.7)
Non-venomous animal contact	56 (34 to 90)	64 (37 to 104)	3.9 (2.4 to 6.2)	2.2 (1.3 to 3.6)
Foreign body	174 (112 to 264)	267 (162 to 436)	11.8 (7.7 to 18.0)	9.2 (5.6 to 15.0)
Pulmonary aspiration and foreign body in airway	7 (5 to 10)	4 (3 to 5)	0.4 (0.3 to 0.5)	0.1 (0.1 to 0.2)
Foreign body in eyes	82 (41 to 150)	169 (88 to 317)	6.4 (3.5 to 11.5)	5.9 (3.2 to 11.0)
Foreign body in other body part	84 (59 to 113)	94 (67 to 128)	5.0 (3.5 to 6.6)	3.1 (2.2 to 4.2)
Environmental heat and cold exposure	130 (86 to 184)	81 (53 to 117)	9.3 (6.2 to 13.1)	2.8 (1.9 to 4.1)
Exposure to forces of nature	19 (13 to 25)	34 (25 to 47)	1.0 (0.7 to 1.4)	1.1 (0.8 to 1.5)
Other unintentional injuries	190 (137 to 255)	200 (142 to 274)	13.1 (9.4 to 17.6)	6.7 (4.8 to 9.3)

Cause	Disability adjusted life years: All-age counts (95% UI) 1990	Disability adjusted life years: All-age counts (95% UI) 2017	Disability adjusted life years: Age-standardised rates per 100,000 (95% UI) 1990	Disability adjusted life years: Age-standardised rates per 100,000 (95% UI) 2017
**Transport and unintentional injuries**	**65 987 (61 342 to 71 088)**	**62 704 (57 768 to 68 076)**	**3 559.8 (3 339.6 to 3 796.5)**	**2 081.6 (1 913.7 to 2 259.3)**
**Transport injuries**	**33 174 (30 970 to 35 687)**	**33 292 (30 710 to 36 063)**	**1 730.9 (1 634.7 to 1 842.3)**	**1 075.6 (993.8 to 1 164.7)**
Road injuries	31 753 (29 674 to 34 197)	30 961 (28 645 to 33 501)	1 652.6 (1 559.2 to 1 760.5)	1 000.8 (926.4 to 1 081.2)
Pedestrian road injuries	11 775 (10 550 to 13 209)	10 833 (9 784 to 11 890)	631.7 (579.0 to 696.3)	355.8 (321.9 to 388.7)
Cyclist road injuries	1 046 (945 to 1 149)	1 651 (1 478 to 1 834)	57.0 (51.9 to 62.4)	53.7 (48.3 to 59.6)
Motorcyclist road injuries	5 047 (4 565 to 5 581)	4 520 (3 978 to 5 070)	256.1 (234.2 to 279.8)	143.2 (126.1 to 160.5)
Motor vehicle road injuries	12 889 (11 844 to 14 094)	12 794 (11 640 to 13 975)	653.8 (606.7 to 712.0)	410.3 (373.7 to 447.3)
Other road injuries	997 (863 to 1 150)	1 162 (1 009 to 1 348)	53.8 (47.0 to 60.8)	37.7 (32.8 to 43.8)
Other transport injuries	1 421 (1 277 to 1 565)	2 331 (2 050 to 2 650)	78.3 (71.1 to 85.3)	74.9 (66.1 to 84.9)
**Unintentional injuries**	**32 813 (29 872 to 36 147)**	**29 412 (26 462 to 32 549)**	**1 828.9 (1 680.5 to 1 987.9)**	**1 005.9 (903.9 to 1 112.3)**
Falls	5 441 (4 746 to 6 255)	9 406 (7 625 to 11 488)	405.2 (353.1 to 464.9)	332.7 (269.5 to 405.2)
Drowning	8 532 (7 598 to 9 750)	4 867 (4 411 to 5 395)	423.5 (374.0 to 464.3)	160.4 (145.3 to 177.9)
Fire, heat, and hot substances	2 926 (2 539 to 3 416)	2 049 (1 753 to 2 474)	164.0 (145.2 to 189.1)	69.7 (60.1 to 83.7)
Poisonings	1 695 (1 510 to 1 918)	1 155 (1 044 to 1 277)	84.2 (76.4 to 93.9)	38.2 (34.5 to 42.2)
Poisoning by carbon monoxide	857 (759 to 975)	627 (564 to 695)	42.7 (38.5 to 47.5)	20.7 (18.6 to 23.0)
Poisoning by other means	838 (734 to 965)	528 (462 to 601)	41.5 (36.9 to 46.8)	17.6 (15.4 to 20.0)
Exposure to mechanical forces	4 262 (3 911 to 4 668)	3 289 (2 891 to 3 833)	238.0 (217.8 to 263.0)	107.2 (94.1 to 125.0)
Unintentional firearm injuries	1 980 (1 782 to 2 197)	1 041 (839 to 1 189)	102.9 (93.3 to 113.6)	33.3 (26.9 to 38.0)
Other exposure to mechanical forces	2 282 (2 005 to 2 593)	2 248 (1 909 to 2 728)	135.1 (118.0 to 154.5)	73.9 (62.6 to 89.5)
Adverse effects of medical treatment	1 288 (1 022 to 1 478)	1 445 (1 290 to 1 757)	78.7 (63.1 to 87.9)	51.3 (45.8 to 62.3)
Animal contact	965 (795 to 1 178)	475 (416 to 543)	48.4 (41.1 to 57.5)	16.3 (14.3 to 18.6)
Venomous animal contact	782 (623 to 983)	314 (270 to 359)	37.6 (30.8 to 46.1)	10.8 (9.3 to 12.4)
Non-venomous animal contact	184 (150 to 235)	161 (130 to 212)	10.8 (8.8 to 13.9)	5.5 (4.4 to 7.2)
Foreign body	4 856 (4 146 to 5 616)	4 429 (3 870 to 5 150)	232.7 (209.4 to 265.2)	155.6 (135.2 to 182.7)
Pulmonary aspiration and foreign body in airway	4 492 (3 817 to 5 231)	4 054 (3 525 to 4 753)	211.7 (183.9 to 242.1)	142.9 (123.2 to 169.1)
Foreign body in eyes	82 (41 to 150)	169 (88 to 317)	6.4 (3.5 to 11.5)	5.9 (3.2 to 11.0)
Foreign body in other body part	283 (231 to 331)	207 (171 to 277)	14.5 (12.0 to 17.0)	6.8 (5.6 to 9.2)
Environmental heat and cold exposure	1 075 (976 to 1 189)	745 (659 to 835)	69.7 (63.7 to 76.4)	24.6 (21.9 to 27.4)
Exposure to forces of nature	72 (64 to 81)	59 (49 to 72)	3.6 (3.3 to 4.0)	1.9 (1.6 to 2.3)
Other unintentional injuries	1 700 (1 554 to 1 851)	1 493 (1 337 to 1 668)	90.8 (83.4 to 98.2)	48.0 (43.1 to 53.3)

Death, YLL, incidence, prevalence, YLD, and DALY counts and rates per 100,000 for all injuries in Tabasco in 1990 and 2017

Cause	Deaths				Years of life lost			
	All-age counts (95% UI)		Age-standardised rates per 100,000 (95% UI)		All-age counts (95% UI)		Age-standardised rates per 100,000 (95% UI)	
	1990	2017	1990	2017	1990	2017	1990	2017
**Transport and unintentional injuries**	**1 007 (956 to 1 065)**	**1 142 (1 075 to 1 252)**	**77.5 (74.9 to 80.4)**	**48.1 (44.7 to 51.8)**	**60 439 (56 324 to 65 271)**	**56 605 (52 308 to 61 167)**	**3 697.1 (3 505.1 to 3 911.5)**	**2 209.1 (2 042.0 to 2 385.8)**
**Transport injuries**	**464 (440 to 492)**	**695 (631 to 758)**	**35.4 (33.8 to 37.2)**	**27.7 (25.2 to 30.1)**	**27 064 (25 363 to 28 943)**	**35 088 (31 950 to 38 340)**	**1 726.3 (1 637.4 to 1 829.9)**	**1 345.1 (1 221.9 to 1 467.6)**
Road injuries	449 (425 to 476)	667 (605 to 728)	34.2 (32.7 to 36.0)	26.5 (24.2 to 28.8)	26 168 (24 494 to 28 046)	33 699 (30 634 to 36 870)	1 670.0 (1 583.8 to 1 770.4)	1 291.7 (1 175.8 to 1 410.4)
Pedestrian road injuries	253 (237 to 271)	318 (287 to 352)	20.5 (19.4 to 21.7)	13.1 (11.8 to 14.4)	14 582 (13 423 to 15 869)	14 847 (13 336 to 16 405)	939.1 (879.3 to 1003.4)	579.2 (521.9 to 638.4)
Cyclist road injuries	13 (12 to 15)	30 (26 to 34)	1.0 (0.9 to 1.1)	1.2 (1.1 to 1.4)	784 (689 to 882)	1 438 (1 259 to 1 652)	50.5 (45.4 to 56.4)	55.7 (48.8 to 64.0)
Motorcyclist road injuries	61 (56 to 67)	123 (108 to 140)	4.2 (3.8 to 4.6)	4.6 (4.0 to 5.2)	3 654 (3 309 to 4 027)	7 007 (6 085 to 8 008)	229.6 (209.6 to 252.5)	261.8 (227.9 to 298.9)
Motor vehicle road injuries	110 (100 to 122)	182 (161 to 201)	7.7 (7.1 to 8.5)	7.0 (6.2 to 7.8)	6 497 (5 900 to 7 249)	9 682 (8 545 to 10 787)	410.3 (375.3 to 453.4)	367.2 (324.7 to 407.7)
Other road injuries	11 (9 to 13)	14 (12 to 17)	0.8 (0.7 to 0.9)	0.6 (0.5 to 0.7)	653 (542 to 771)	726 (608 to 849)	40.4 (33.5 to 46.7)	27.8 (23.4 to 32.5)
Other transport injuries	15 (14 to 17)	28 (24 to 32)	1.2 (1.1 to 1.3)	1.1 (1.0 to 1.3)	896 (789 to 1 013)	1 389 (1 181 to 1 590)	56.3 (50.4 to 62.9)	53.4 (45.6 to 60.8)
**Unintentional injuries**	**544 (509 to 585)**	**467 (435 to 506)**	**42.1 (40.4 to 44.2)**	**20.5 (19.1 to 22.0)**	**33 375 (30 438 to 36 816)**	**21 517 (19 756 to 23 543)**	**1 970.8 (1 842.6 to 2 124.9)**	**864.0 (793.7 to 944.7)**
Falls	75 (71 to 79)	111 (101 to 121)	9.6 (9.1 to 10.0)	5.5 (5.0 to 6.0)	3 059 (2 885 to 3 317)	3 275 (2 939 to 3 601)	255.1 (242.1 to 270.4)	139.8 (126.3 to 152.7)
Drowning	236 (216 to 258)	146 (133 to 161)	15.3 (14.3 to 16.4)	5.9 (5.4 to 6.5)	15 502 (13 942 to 17 386)	8 032 (7 255 to 8 942)	862.4 (791.1 to 946.3)	313.4 (283.2 to 349.5)
Fire, heat, and hot substances	34 (30 to 39)	25 (22 to 27)	2.8 (2.6 to 3.0)	1.2 (1.1 to 1.2)	2 144 (1 809 to 2 536)	950 (856 to 1 049)	122.3 (107.5 to 140.0)	39.2 (35.4 to 43.3)
Poisonings	17 (15 to 20)	12 (11 to 13)	1.1 (1.0 to 1.2)	0.5 (0.5 to 0.6)	1 176 (1 020 to 1 367)	631 (561 to 706)	63.9 (56.9 to 72.6)	24.9 (22.1 to 27.9)
Poisoning by carbon monoxide	3 (2 to 3)	2 (2 to 3)	0.2 (0.2 to 0.2)	0.1 (0.1 to 0.1)	185 (158 to 216)	125 (107 to 145)	10.3 (9.1 to 11.8)	4.9 (4.2 to 5.6)
Poisoning by other means	15 (13 to 17)	10 (9 to 11)	0.9 (0.9 to 1.0)	0.4 (0.4 to 0.4)	992 (862 to 1 160)	506 (447 to 569)	53.6 (47.6 to 61.2)	20.0 (17.7 to 22.5)
Exposure to mechanical forces	53 (50 to 57)	39 (35 to 43)	4.1 (3.8 to 4.3)	1.6 (1.5 to 1.8)	3 118 (2 876 to 3 394)	1 844 (1 656 to 2 054)	198.2 (184.8 to 213.2)	71.6 (64.5 to 79.6)
Unintentional firearm injuries	16 (14 to 19)	9 (7 to 10)	1.1 (1.0 to 1.3)	0.4 (0.3 to 0.4)	975 (861 to 1 127)	463 (360 to 539)	60.0 (53.3 to 69.7)	17.6 (13.7 to 20.4)
Other exposure to mechanical forces	37 (33 to 40)	30 (27 to 33)	2.9 (2.7 to 3.1)	1.2 (1.1 to 1.4)	2 143 (1 915 to 2 370)	1 381 (1 236 to 1 559)	138.2 (125.1 to 150.4)	54.0 (48.4 to 60.8)
Adverse effects of medical treatment	18 (16 to 21)	25 (22 to 35)	1.8 (1.6 to 2.1)	1.2 (1.1 to 1.7)	985 (828 to 1 162)	891 (765 to 1 321)	62.0 (54.6 to 72.2)	38.1 (32.9 to 56.3)
Animal contact	14 (12 to 17)	8 (7 to 9)	1.0 (0.9 to 1.1)	0.3 (0.3 to 0.4)	1 014 (840 to 1 225)	354 (313 to 403)	52.7 (45.0 to 62.2)	14.6 (12.9 to 16.6)
Venomous animal contact	11 (9 to 13)	5 (4 to 6)	0.7 (0.6 to 0.8)	0.2 (0.2 to 0.3)	810 (651 to 1 015)	231 (197 to 272)	40.8 (33.8 to 50.0)	9.6 (8.2 to 11.4)
Non-venomous animal contact	3 (2 to 4)	3 (2 to 3)	0.3 (0.2 to 0.3)	0.1 (0.1 to 0.1)	204 (140 to 245)	123 (103 to 146)	11.8 (8.4 to 13.9)	5.0 (4.2 to 6.0)
Foreign body	61 (54 to 69)	65 (58 to 74)	3.9 (3.6 to 4.3)	2.8 (2.5 to 3.2)	4 275 (3 727 to 4 955)	3 776 (3 211 to 4 519)	222.9 (198.5 to 252.8)	154.3 (130.4 to 185.5)
Pulmonary aspiration and foreign body in airway	58 (51 to 65)	63 (56 to 72)	3.7 (3.4 to 4.1)	2.7 (2.4 to 3.1)	4 064 (3 533 to 4 719)	3 679 (3 123 to 4 407)	211.6 (187.6 to 240.3)	150.6 (127.1 to 181.2)
Foreign body in eyes								
Foreign body in other body part	3 (2 to 4)	2 (1 to 3)	0.2 (0.1 to 0.2)	0.1 (0.1 to 0.1)	211 (146 to 255)	97 (79 to 152)	11.3 (8.1 to 13.4)	3.8 (3.0 to 5.9)
Environmental heat and cold exposure	8 (7 to 9)	6 (5 to 7)	0.8 (0.7 to 0.8)	0.3 (0.2 to 0.3)	422 (378 to 468)	230 (200 to 258)	29.6 (27.1 to 32.1)	9.3 (8.1 to 10.4)
Exposure to forces of nature	1 (1 to 1)	4 (4 to 5)	0.1 (0.1 to 0.1)	0.2 (0.2 to 0.2)	45 (41 to 50)	227 (206 to 248)	2.8 (2.5 to 3.0)	8.9 (8.1 to 9.8)
Other unintentional injuries	27 (25 to 29)	25 (22 to 28)	1.9 (1.7 to 2.0)	1.0 (0.9 to 1.1)	1 635 (1 492 to 1 797)	1 307 (1 148 to 1 482)	98.9 (91.0 to 107.5)	49.7 (43.7 to 56.3)

Cause	Incidence				Prevalence			
	All-age counts (95% UI)		Age-standardised rates per 100,000 (95% UI)		All-age counts (95% UI)		Age-standardised rates per 100,000 (95% UI)	
	1990	2017	1990	2017	1990	2017	1990	2017
**Transport and unintentional injuries**	**55 494 (50 892 to 60 630)**	**111 906 (102 128 to 122 877)**	**4 179.5 (3 808.9 to 4 569.5)**	**4 700.8 (4 300.1 to 5 163.3)**	**101 330 (95 067 to 108 390)**	**239 201 (224 096 to 258 264)**	**9 699.8 (9 151.7 to 10 402.8)**	**10 234.4 (9 622.9 to 11 049.9)**
**Transport injuries**	**5 791 (4 960 to 6 780)**	**17 371 (14 591 to 20 238)**	**424.0 (364.0 to 483.0)**	**685.7 (579.9 to 793.7)**	**13 011 (12 106 to 13 858)**	**47 730 (44 432 to 51 214)**	**1 322.2 (1 233.6 to 1 410.5)**	**2 015.8 (1 877.3 to 2 163.9)**
Road injuries	5 518 (4 676 to 6 519)	17 106 (14 323 to 19 972)	405.6 (346.6 to 465.1)	674.7 (568.4 to 783.0)	11 856 (11 008 to 12 687)	46 416 (43 164 to 49 858)	1 218.0 (1 134.8 to 1 303.2)	1 959.7 (1 821.1 to 2 107.4)
Pedestrian road injuries	1 009 (807 to 1 242)	2 549 (2 026 to 3 127)	78.5 (65.2 to 94.2)	102.2 (82.0 to 125.4)	3 371 (2 880 to 3 965)	10 812 (9 361 to 12 645)	352.6 (306.4 to 409.1)	464.0 (402.9 to 539.7)
Cyclist road injuries	989 (765 to 1 276)	2 518 (2 008 to 3 130)	65.8 (53.0 to 81.4)	101.1 (80.8 to 124.9)	1 505 (1 255 to 1 794)	5 068 (4 280 to 5 920)	142.1 (119.8 to 165.0)	213.5 (182.0 to 247.8)
Motorcyclist road injuries	827 (650 to 1 030)	2 426 (1 879 to 3 070)	57.8 (46.4 to 70.4)	93.8 (73.0 to 118.3)	2 649 (2 294 to 3 059)	10 287 (8 772 to 12 025)	266.3 (231.0 to 306.6)	425.4 (363.0 to 496.3)
Motor vehicle road injuries	1 819 (1 474 to 2 191)	7 641 (6 241 to 9 245)	138.4 (114.7 to 163.8)	297.8 (244.7 to 355.9)	2 870 (2 519 to 3 346)	15 933 (13 781 to 18 795)	309.6 (269.3 to 360.9)	672.6 (580.5 to 791.3)
Other road injuries	874 (656 to 1 124)	1 978 (1 540 to 2 481)	65.1 (51.6 to 79.2)	79.8 (62.7 to 98.7)	1 461 (1 210 to 1 765)	4 317 (3 578 to 5 277)	147.4 (123.3 to 175.9)	184.2 (153.2 to 224.7)
Other transport injuries	273 (217 to 350)	265 (220 to 318)	18.4 (15.3 to 22.1)	11.0 (9.2 to 13.2)	1 155 (955 to 1 382)	1 314 (1 123 to 1 522)	104.2 (88.4 to 121.0)	56.1 (48.3 to 64.4)
**Unintentional injuries**	**49 702 (45 211 to 54 757)**	**94 535 (85 639 to 105 206)**	**3 755.5 (3 386.4 to 4 144.9)**	**4 011.1 (3 637.5 to 4 453.0)**	**88 319 (82 192 to 95 346)**	**191 471 (177 101 to 210 260)**	**8 377.7 (7 821.3 to 9 047.9)**	**8 218.6 (7 610.5 to 9 018.3)**
Falls	20 335 (17 713 to 23 335)	48 768 (43 292 to 54 990)	1 698.4 (1 520.8 to 1 907.8)	2 152.6 (1 911.9 to 2 414.5)	33 920 (29 707 to 39 028)	95 856 (84 916 to 108 817)	3 483.2 (3 113.2 to 3 988.7)	4 245.2 (3 784.1 to 4 833.7)
Drowning	181 (156 to 210)	97 (79 to 121)	11.8 (10.3 to 13.4)	4.0 (3.3 to 4.9)	758 (664 to 877)	576 (505 to 665)	76.1 (67.0 to 87.1)	25.0 (22.1 to 28.9)
Fire, heat, and hot substances	2 161 (1 712 to 2 669)	2 412 (1 965 to 2 948)	115.1 (93.8 to 137.1)	97.8 (79.9 to 119.3)	19 010 (15 783 to 23 826)	28 157 (23 314 to 35 666)	1 526.6 (1 286.5 to 1 850.6)	1 150.2 (955.0 to 1 448.1)
Poisonings	732 (546 to 963)	1 026 (770 to 1 329)	35.4 (27.3 to 45.6)	41.7 (31.3 to 54.2)	594 (458 to 726)	1 173 (871 to 1 464)	41.5 (32.5 to 50.4)	46.6 (34.7 to 58.1)
Poisoning by carbon monoxide	120 (79 to 184)	111 (63 to 173)	6.5 (4.6 to 9.2)	4.6 (2.7 to 7.1)	78 (60 to 104)	84 (64 to 108)	5.7 (4.4 to 7.4)	3.4 (2.6 to 4.3)
Poisoning by other means	612 (447 to 823)	915 (677 to 1 201)	28.9 (21.6 to 38.1)	37.1 (27.4 to 48.8)	516 (376 to 644)	1 089 (777 to 1 379)	35.9 (26.5 to 44.4)	43.2 (30.9 to 54.4)
Exposure to mechanical forces	11 798 (10 060 to 13 853)	18 907 (16 115 to 21 946)	780.7 (678.5 to 898.8)	746.6 (639.5 to 862.0)	21 242 (18 494 to 24 683)	45 891 (39 589 to 54 046)	2 079.6 (1 825.0 to 2 393.6)	1 913.1 (1 658.2 to 2 244.9)
Unintentional firearm injuries	560 (404 to 748)	492 (344 to 663)	35.3 (26.1 to 46.0)	19.3 (13.7 to 25.9)	2 038 (1 598 to 2 453)	2 596 (2 090 to 3 109)	201.5 (161.4 to 242.9)	110.2 (89.3 to 131.8)
Other exposure to mechanical forces	11 238 (9 564 to 13 201)	18 415 (15 674 to 21 377)	745.4 (646.8 to 859.2)	727.3 (621.3 to 841.2)	19 204 (16 303 to 22 762)	43 295 (36 930 to 51 606)	1 878.1 (1 613.2 to 2 205.6)	1 802.9 (1 546.1 to 2 140.7)
Adverse effects of medical treatment	1 440 (1 217 to 1 670)	4 045 (3 420 to 4 629)	117.4 (101.3 to 134.1)	168.5 (143.1 to 192.5)	110 (85 to 138)	310 (236 to 380)	9.0 (7.0 to 11.0)	12.9 (9.9 to 15.8)
Animal contact	4 202 (3 456 to 5 232)	4 907 (4 276 to 5 730)	284.6 (243.1 to 335.3)	203.6 (176.5 to 233.3)	4 138 (3 536 to 4 832)	6 056 (5 287 to 6 942)	379.8 (332.4 to 432.6)	256.3 (224.9 to 292.5)
Venomous animal contact	1 474 (1 115 to 1 961)	1 693 (1 339 to 2 109)	99.6 (77.1 to 126.7)	69.1 (54.9 to 85.5)	924 (759 to 1 107)	1 215 (1 025 to 1 406)	77.5 (64.9 to 90.2)	50.9 (42.6 to 58.1)
Non-venomous animal contact	2 727 (2 295 to 3 426)	3 214 (2 718 to 3 854)	185.0 (153.9 to 223.9)	132.5 (112.0 to 158.7)	3 214 (2 687 to 3 887)	4 841 (4 093 to 5 724)	302.4 (255.4 to 354.9)	206.0 (174.8 to 242.0)
Foreign body	5 345 (2 803 to 8 590)	9 991 (4 418 to 17 754)	471.4 (203.5 to 775.9)	423.7 (183.3 to 757.9)	2 187 (1 645 to 2 986)	4 068 (3 028 to 5 674)	203.0 (155.0 to 273.5)	175.8 (131.4 to 243.4)
Pulmonary aspiration and foreign body in airway	146 (101 to 205)	74 (56 to 102)	7.4 (5.4 to 10.1)	3.1 (2.3 to 4.2)	94 (66 to 128)	64 (52 to 80)	6.7 (4.9 to 9.0)	2.7 (2.2 to 3.3)
Foreign body in eyes	4 800 (2 256 to 8 034)	9 389 (3 804 to 17 286)	440.2 (170.4 to 747.5)	398.6 (156.7 to 735.1)	928 (464 to 1 649)	2 020 (1 089 to 3 449)	101.1 (57.8 to 164.8)	93.8 (51.6 to 151.9)
Foreign body in other body part	400 (302 to 546)	527 (414 to 672)	23.8 (18.6 to 30.7)	22.0 (17.3 to 27.8)	1 170 (934 to 1 497)	1 984 (1 600 to 2 517)	95.2 (77.9 to 118.6)	81.9 (65.5 to 103.3)
Environmental heat and cold exposure	708 (487 to 955)	482 (357 to 684)	48.5 (36.9 to 60.7)	20.9 (15.6 to 28.7)	2 341 (1 871 to 2 878)	1 868 (1 571 to 2 235)	208.0 (173.7 to 248.8)	80.6 (68.4 to 96.0)
Exposure to forces of nature	118 (96 to 147)	302 (255 to 363)	7.0 (5.9 to 8.3)	11.9 (10.0 to 14.2)	415 (296 to 563)	1 231 (865 to 1 700)	27.6 (20.2 to 37.3)	48.7 (34.3 to 67.2)
Other unintentional injuries	2 682 (2 209 to 3 287)	3 599 (3 060 to 4 142)	185.2 (157.7 to 219.6)	146.0 (124.2 to 167.7)	3 605 (3 098 to 4 193)	6 291 (5 448 to 7 228)	343.4 (297.3 to 393.8)	264.2 (229.5 to 302.1)

Cause	Years lived with disability				Disability adjusted life years			
	All-age counts (95% UI)		Age-standardised rates per 100,000 (95% UI)		All-age counts (95% UI)		Age-standardised rates per 100,000 (95% UI)	
	1990	2017	1990	2017	1990	2017	1990	2017
**Transport and unintentional injuries**	**4 482 (3 284 to 5 943)**	**10 196 (7 355 to 13 785)**	**422.3 (309.3 to 556.8)**	**435.7 (314.1 to 587.5)**	**64 921 (60 524 to 70 016)**	**66 801 (61 520 to 72 647)**	**4 119.4 (3 890.7 to 4 382.0)**	**2 644.8 (2 439.3 to 2 880.2)**
**Transport injuries**	**900 (659 to 1 191)**	**2 895 (2 098 to 3 871)**	**87.2 (64.0 to 115.1)**	**120.4 (87.2 to 160.5)**	**27 964 (26 239 to 29 904)**	**37 983 (34 707 to 41 316)**	**1 813.5 (1 719.1 to 1 919.3)**	**1 465.5 (1 337.3 to 1 594.6)**
Road injuries	826 (605 to 1 097)	2 826 (2 052 to 3 790)	80.9 (59.3 to 107.3)	117.5 (85.2 to 156.8)	26 994 (25 310 to 28 894)	36 525 (33 335 to 39 704)	1 750.9 (1 659.4 to 1 853.2)	1 409.2 (1 285.8 to 1 533.2)
Pedestrian road injuries	245 (174 to 330)	686 (484 to 944)	24.9 (17.5 to 32.6)	29.0 (20.5 to 39.6)	14 826 (13 640 to 16 124)	15 533 (13 979 to 17 138)	963.4 (901.3 to 1032.8)	608.1 (548.8 to 669.4)
Cyclist road injuries	105 (74 to 138)	302 (212 to 403)	9.5 (6.9 to 12.5)	12.5 (8.8 to 16.6)	889 (794 to 996)	1 739 (1 535 to 1 963)	60.1 (54.3 to 66.5)	68.3 (60.1 to 76.8)
Motorcyclist road injuries	186 (131 to 248)	626 (430 to 853)	17.9 (12.6 to 24.1)	25.5 (17.5 to 34.8)	3 839 (3 482 to 4 229)	7 632 (6 712 to 8 619)	247.5 (226.8 to 270.6)	287.3 (252.8 to 324.3)
Motor vehicle road injuries	207 (150 to 277)	1 001 (726 to 1 338)	21.2 (15.5 to 28.0)	41.5 (30.0 to 55.7)	6 704 (6 092 to 7 437)	10 682 (9 535 to 11 883)	431.5 (395.6 to 474.8)	408.7 (365.2 to 453.8)
Other road injuries	83 (60 to 113)	212 (152 to 291)	8.0 (5.8 to 10.8)	8.9 (6.4 to 12.2)	736 (617 to 857)	938 (793 to 1 086)	48.4 (40.9 to 55.3)	36.8 (31.2 to 42.5)
Other transport injuries	74 (53 to 99)	69 (50 to 93)	6.3 (4.5 to 8.3)	2.9 (2.1 to 3.9)	970 (866 to 1 089)	1 459 (1 250 to 1 661)	62.6 (56.4 to 69.2)	56.3 (48.4 to 63.9)
**Unintentional injuries**	**3 582 (2 609 to 4 756)**	**7 301 (5 228 to 9 903)**	**335.1 (242.8 to 445.1)**	**315.3 (225.7 to 421.9)**	**36 957 (33 768 to 40 701)**	**28 818 (25 946 to 32 015)**	**2 305.9 (2 138.5 to 2 500.0)**	**1 179.3 (1 059.3 to 1 315.8)**
Falls	2 032 (1 492 to 2 675)	4 872 (3 534 to 6 481)	200.9 (146.5 to 262.5)	214.8 (155.6 to 284.6)	5 091 (4 483 to 5 779)	8 148 (6 686 to 9 807)	456.0 (399.8 to 518.6)	354.6 (290.4 to 426.2)
Drowning	44 (30 to 61)	30 (21 to 42)	4.2 (2.9 to 5.7)	1.3 (0.9 to 1.8)	15 545 (13 986 to 17 426)	8 062 (7 283 to 8 975)	866.6 (794.9 to 951.4)	314.7 (284.2 to 350.8)
Fire, heat, and hot substances	413 (258 to 648)	545 (318 to 889)	31.9 (19.9 to 50.0)	22.2 (13.0 to 36.0)	2 557 (2 197 to 3 027)	1 495 (1 244 to 1 842)	154.2 (134.6 to 179.0)	61.4 (51.2 to 75.3)
Poisonings	70 (45 to 105)	134 (81 to 202)	4.7 (3.0 to 7.0)	5.3 (3.2 to 7.9)	1 246 (1 085 to 1 433)	765 (681 to 864)	68.6 (61.1 to 77.2)	30.2 (26.9 to 34.0)
Poisoning by carbon monoxide	9 (5 to 13)	8 (5 to 13)	0.6 (0.4 to 0.9)	0.3 (0.2 to 0.5)	193 (166 to 224)	133 (116 to 153)	10.9 (9.6 to 12.4)	5.2 (4.5 to 6.0)
Poisoning by other means	62 (39 to 94)	126 (75 to 193)	4.1 (2.6 to 6.2)	5.0 (3.0 to 7.6)	1 053 (919 to 1 219)	631 (557 to 717)	57.7 (51.5 to 65.0)	25.0 (22.1 to 28.4)
Exposure to mechanical forces	505 (346 to 721)	989 (667 to 1 444)	48.0 (32.9 to 68.9)	41.0 (27.7 to 59.9)	3 623 (3 311 to 3 964)	2 833 (2 449 to 3 342)	246.3 (224.8 to 270.5)	112.6 (97.2 to 133.6)
Unintentional firearm injuries	41 (27 to 63)	46 (29 to 72)	3.8 (2.5 to 6.0)	1.9 (1.2 to 3.0)	1 016 (901 to 1 169)	509 (397 to 586)	63.9 (56.9 to 74.3)	19.5 (15.8 to 22.4)
Other exposure to mechanical forces	464 (318 to 661)	943 (634 to 1 382)	44.2 (30.5 to 63.5)	39.1 (26.5 to 57.3)	2 607 (2 318 to 2 905)	2 324 (1 967 to 2 813)	182.4 (161.9 to 204.2)	93.1 (78.8 to 112.8)
Adverse effects of medical treatment	15 (9 to 22)	41 (26 to 62)	1.2 (0.7 to 1.8)	1.7 (1.1 to 2.6)	1 000 (842 to 1 175)	932 (804 to 1 352)	63.2 (55.8 to 73.5)	39.9 (34.5 to 58.0)
Animal contact	113 (75 to 160)	137 (92 to 198)	9.1 (6.1 to 12.9)	5.7 (3.8 to 8.2)	1 126 (943 to 1 349)	492 (425 to 568)	61.8 (53.3 to 72.2)	20.3 (17.6 to 23.4)
Venomous animal contact	63 (40 to 92)	69 (45 to 99)	4.5 (2.9 to 6.4)	2.8 (1.8 to 3.9)	872 (712 to 1 081)	300 (255 to 355)	45.3 (37.8 to 54.9)	12.4 (10.6 to 14.6)
Non-venomous animal contact	50 (30 to 80)	68 (40 to 111)	4.6 (2.8 to 7.3)	2.9 (1.7 to 4.7)	254 (183 to 306)	192 (155 to 239)	16.5 (11.9 to 19.9)	7.9 (6.4 to 9.9)
Foreign body	138 (80 to 206)	290 (142 to 367)	12.6 (8.1 to 18.8)	10.0 (6.2 to 15.9)	4 413 (3 860 to 5 067)	4 026 (3 438 to 4 745)	235.4 (210.0 to 265.6)	164.3 (140.3 to 194.8)
Pulmonary aspiration and foreign body in airway	5 (3 to 7)	3 (2 to 4)	0.3 (0.2 to 0.5)	0.1 (0.1 to 0.2)	4 069 (3 540 to 4 724)	3 681 (3 126 to 4 409)	211.9 (188.0 to 240.8)	150.7 (127.2 to 181.3)
Foreign body in eyes	61 (30 to 114)	135 (70 to 251)	6.8 (3.6 to 11.9)	6.1 (3.2 to 11.0)	61 (30 to 114)	135 (70 to 251)	6.8 (3.6 to 11.9)	6.1 (3.2 to 11.0)
Foreign body in other body part	72 (51 to 96)	93 (65 to 125)	5.5 (3.9 to 7.2)	3.8 (2.7 to 5.1)	283 (220 to 335)	190 (155 to 247)	16.8 (13.6 to 19.6)	7.5 (6.1 to 9.8)
Environmental heat and cold exposure	92 (61 to 131)	70 (46 to 101)	8.1 (5.4 to 11.4)	3.1 (2.0 to 4.4)	514 (459 to 574)	300 (262 to 344)	37.7 (33.9 to 41.9)	12.3 (10.7 to 14.1)
Exposure to forces of nature	20 (14 to 28)	43 (31 to 59)	1.3 (0.9 to 1.8)	1.7 (1.2 to 2.3)	65 (57 to 74)	270 (245 to 296)	4.1 (3.6 to 4.6)	10.6 (9.7 to 11.7)
Other unintentional injuries	141 (102 to 189)	208 (147 to 285)	13.1 (9.4 to 17.4)	8.7 (6.1 to 11.9)	1 776 (1 617 to 1 956)	1 515 (1 348 to 1 706)	112.0 (103.1 to 122.1)	58.4 (52.0 to 65.7)

Death, YLL, incidence, prevalence, YLD, and DALY counts and rates per 100,000 for all injuries in Tamaulipas in 1990 and 2017

Cause	Deaths: All-age counts (95% UI) 1990	Deaths: All-age counts (95% UI) 2017	Deaths: Age-standardised rates per 100,000 (95% UI) 1990	Deaths: Age-standardised rates per 100,000 (95% UI) 2017	Years of life lost: All-age counts (95% UI) 1990	Years of life lost: All-age counts (95% UI) 2017	Years of life lost: Age-standardised rates per 100,000 (95% UI) 1990	Years of life lost: Age-standardised rates per 100,000 (95% UI) 2017
**Transport and unintentional injuries**	**1 308 (1 250 to 1 372)**	**1 087 (1 020 to 1 165)**	**65.2 (62.9 to 67.6)**	**31.4 (29.5 to 33.6)**	**71 185 (66 718 to 76 249)**	**46 962 (43 581 to 50 802)**	**2 933.4 (2 774.1 to 3 104.8)**	**1 313.4 (1 218.3 to 1 421.7)**
**Transport injuries**	**643 (610 to 681)**	**577 (533 to 624)**	**30.8 (29.3 to 32.4)**	**16.1 (14.9 to 17.4)**	**35 345 (33 108 to 37 976)**	**26 522 (24 310 to 28 903)**	**1 466.1 (1 384.4 to 1 555.2)**	**725.8 (666.7 to 790.5)**
Road injuries	624 (591 to 661)	548 (506 to 592)	29.8 (28.4 to 31.4)	15.3 (14.2 to 16.6)	34 298 (32 110 to 36 832)	25 215 (23 122 to 27 470)	1 421.8 (1 342.1 to 1 507.9)	690.3 (633.6 to 753.0)
Pedestrian road injuries	292 (273 to 315)	254 (235 to 276)	15.2 (14.3 to 16.1)	7.3 (6.8 to 7.9)	15 358 (13 941 to 17 000)	10 567 (9 590 to 11 570)	655.2 (604.9 to 715.0)	294.0 (267.0 to 321.8)
Cyclist road injuries	13 (12 to 14)	20 (18 to 22)	0.6 (0.5 to 0.7)	0.6 (0.5 to 0.6)	699 (622 to 782)	858 (766 to 973)	29.5 (26.6 to 32.7)	23.6 (21.0 to 26.6)
Motorcyclist road injuries	82 (74 to 91)	55 (48 to 62)	3.5 (3.2 to 3.8)	1.5 (1.3 to 1.7)	4 792 (4 306 to 5 349)	2 959 (2 583 to 3 365)	192.0 (173.6 to 211.2)	79.9 (69.8 to 90.2)
Motor vehicle road injuries	221 (204 to 240)	205 (185 to 225)	9.8 (9.1 to 10.6)	5.6 (5.1 to 6.1)	12 602 (11 502 to 13 742)	10 186 (9 158 to 11 302)	510.9 (470.8 to 552.4)	275.9 (248.4 to 306.5)
Other road injuries	15 (13 to 18)	14 (12 to 16)	0.7 (0.6 to 0.8)	0.4 (0.3 to 0.4)	837 (694 to 988)	644 (548 to 749)	34.2 (29.0 to 39.6)	17.6 (15.0 to 20.4)
Other transport injuries	19 (18 to 21)	29 (26 to 33)	1.0 (0.9 to 1.1)	0.8 (0.7 to 0.9)	1 047 (938 to 1 174)	1 307 (1 149 to 1 492)	44.3 (40.0 to 49.2)	35.5 (31.2 to 40.5)
**Unintentional injuries**	**665 (630 to 704)**	**510 (477 to 546)**	**34.4 (33.1 to 35.8)**	**15.2 (14.1 to 16.3)**	**35 840 (33 069 to 39 173)**	**20 440 (18 774 to 22 493)**	**1 467.3 (1 372.6 to 1 577.8)**	**587.5 (537.0 to 646.4)**
Falls	120 (114 to 126)	141 (131 to 153)	8.8 (8.4 to 9.3)	4.5 (4.1 to 4.8)	3 678 (3 449 to 3 941)	3 323 (3 055 to 3 621)	201.4 (191.4 to 212.8)	97.6 (89.7 to 106.3)
Drowning	224 (207 to 243)	115 (105 to 126)	9.5 (8.9 to 10.2)	3.3 (3.0 to 3.6)	13 858 (12 682 to 15 504)	5 894 (5 272 to 6 580)	529.1 (486.1 to 583.5)	164.8 (147.8 to 183.5)
Fire, heat, and hot substances	55 (50 to 61)	38 (34 to 40)	3.1 (2.9 to 3.3)	1.2 (1.0 to 1.2)	2 790 (2 443 to 3 254)	1 281 (1 115 to 1 355)	117.2 (105.0 to 133.6)	35.6 (32.2 to 39.2)
Poisonings	25 (23 to 28)	16 (15 to 18)	1.1 (1.1 to 1.2)	0.5 (0.4 to 0.5)	1 552 (1 375 to 1 745)	756 (682 to 840)	60.4 (54.2 to 66.9)	21.3 (19.2 to 23.7)
Poisoning by carbon monoxide	12 (11 to 14)	9 (8 to 10)	0.6 (0.5 to 0.6)	0.3 (0.2 to 0.3)	737 (654 to 832)	409 (354 to 468)	28.9 (26.0 to 32.2)	11.4 (10.2 to 12.9)
Poisoning by other means	13 (12 to 14)	7 (7 to 8)	0.6 (0.5 to 0.6)	0.2 (0.2 to 0.2)	815 (709 to 938)	347 (308 to 389)	31.4 (27.8 to 35.6)	9.9 (8.8 to 11.1)
Exposure to mechanical forces	75 (70 to 80)	46 (42 to 50)	3.7 (3.5 to 3.9)	1.3 (1.2 to 1.4)	4 035 (3 765 to 4 375)	1 990 (1 789 to 2 191)	170.2 (159.5 to 182.4)	54.5 (49.0 to 60.0)
Unintentional firearm injuries	37 (33 to 40)	17 (13 to 19)	1.7 (1.5 to 1.8)	0.5 (0.4 to 0.5)	2 046 (1 843 to 2 264)	821 (636 to 939)	83.7 (76.0 to 92.3)	22.2 (17.2 to 25.3)
Other exposure to mechanical forces	38 (34 to 42)	29 (26 to 32)	2.0 (1.8 to 2.1)	0.8 (0.7 to 0.9)	1 989 (1 743 to 2 198)	1 169 (1 047 to 1 342)	86.5 (76.4 to 94.6)	32.3 (29.0 to 37.0)
Adverse effects of medical treatment	31 (28 to 35)	39 (34 to 44)	2.0 (1.8 to 2.2)	1.2 (1.1 to 1.7)	1 342 (1 160 to 1 577)	1 198 (1 029 to 1 759)	61.4 (54.1 to 69.9)	35.5 (30.5 to 41.9)
Animal contact	9 (8 to 11)	5 (4 to 5)	0.4 (0.4 to 0.5)	0.1 (0.1 to 0.2)	590 (486 to 725)	189 (167 to 215)	22.5 (19.1 to 27.0)	5.5 (4.9 to 6.3)
Venomous animal contact	6 (5 to 8)	3 (2 to 3)	0.3 (0.2 to 0.3)	0.1 (0.1 to 0.1)	432 (342 to 555)	104 (80 to 125)	16.0 (13.0 to 20.1)	3.1 (2.4 to 3.7)
Non-venomous animal contact	3 (2 to 3)	2 (2 to 3)	0.2 (0.1 to 0.2)	0.1 (0.1 to 0.1)	158 (125 to 187)	85 (73 to 112)	6.6 (5.2 to 7.6)	2.4 (2.0 to 3.3)
Foreign body	90 (79 to 102)	84 (75 to 95)	3.9 (3.6 to 4.3)	2.5 (2.3 to 2.9)	6 059 (5 116 to 7 075)	4 686 (3 978 to 5 551)	223.4 (192.5 to 256.9)	140.5 (118.5 to 168.1)
Pulmonary aspiration and foreign body in airway	86 (75 to 97)	82 (73 to 93)	3.8 (3.4 to 4.1)	2.5 (2.2 to 2.8)	5 798 (4 876 to 6 783)	4 569 (3 880 to 5 430)	213.3 (183.2 to 245.9)	137.3 (115.6 to 164.2)
Foreign body in eyes								
Foreign body in other body part	4 (3 to 5)	2 (2 to 3)	0.2 (0.1 to 0.2)	0.1 (0.0 to 0.1)	260 (179 to 308)	118 (97 to 182)	10.1 (7.0 to 11.7)	3.3 (2.7 to 5.0)
Environmental heat and cold exposure	11 (10 to 12)	6 (5 to 7)	0.7 (0.6 to 0.7)	0.2 (0.2 to 0.2)	464 (421 to 514)	211 (187 to 233)	22.4 (20.7 to 24.4)	5.9 (5.2 to 6.5)
Exposure to forces of nature	1 (1 to 1)	3 (2 to 3)	0.0 (0.0 to 0.1)	0.1 (0.1 to 0.1)	64 (57 to 73)	132 (120 to 145)	2.5 (2.3 to 2.8)	3.7 (3.4 to 4.1)
Other unintentional injuries	25 (23 to 27)	17 (15 to 19)	1.1 (1.1 to 1.2)	0.5 (0.4 to 0.5)	1 406 (1 287 to 1 540)	831 (740 to 933)	56.8 (52.6 to 61.6)	22.5 (20.1 to 25.2)

Cause	Incidence: All-age counts (95% UI) 1990	Incidence: All-age counts (95% UI) 2017	Incidence: Age-standardised rates per 100,000 (95% UI) 1990	Incidence: Age-standardised rates per 100,000 (95% UI) 2017	Prevalence: All-age counts (95% UI) 1990	Prevalence: All-age counts (95% UI) 2017	Prevalence: Age-standardised rates per 100,000 (95% UI) 1990	Prevalence: Age-standardised rates per 100,000 (95% UI) 2017
**Transport and unintentional injuries**	**82 903 (76 784 to 89 402)**	**145 315 (134 530 to 157 064)**	**3 997.5 (3 716.9 to 4 301.9)**	**4 225.6 (3 911.8 to 4 567.9)**	**169 697 (159 182 to 181 852)**	**319 270 (297 503 to 346 536)**	**9 469.8 (8 914.6 to 10 167.0)**	**9 146.6 (8 547.7 to 9 945.7)**
**Transport injuries**	**8 677 (7 512 to 10 061)**	**18 685 (16 270 to 21 404)**	**399.9 (350.0 to 454.5)**	**516.1 (449.7 to 587.9)**	**21 386 (19 950 to 22 775)**	**52 901 (49 120 to 57 215)**	**1 222.2 (1 140.1 to 1 303.9)**	**1 488.4 (1 381.4 to 1 607.8)**
Road injuries	8 261 (7 133 to 9 661)	18 319 (15 857 to 21 039)	381.3 (331.3 to 435.3)	505.5 (439.7 to 577.8)	19 449 (18 078 to 20 803)	51 013 (47 248 to 55 240)	1 119.2 (1 042.5 to 1 199.1)	1 434.6 (1 329.0 to 1 550.8)
Pedestrian road injuries	1 361 (1 116 to 1 658)	2 434 (1 994 to 2 937)	66.0 (54.7 to 78.9)	68.1 (55.5 to 81.7)	5 101 (4 379 to 5 998)	10 982 (9 518 to 12 812)	296.2 (256.8 to 345.0)	312.7 (271.9 to 363.0)
Cyclist road injuries	1 133 (869 to 1 487)	2 248 (1 792 to 2 815)	49.3 (38.8 to 62.7)	63.4 (50.3 to 78.8)	1 951 (1 633 to 2 298)	4 931 (4 182 to 5 779)	106.4 (89.7 to 123.3)	138.8 (118.2 to 161.8)
Motorcyclist road injuries	1 058 (833 to 1 325)	1 680 (1 282 to 2 117)	46.2 (36.9 to 57.0)	46.1 (35.3 to 58.0)	3 825 (3 314 to 4 446)	7 742 (6 633 to 9 084)	215.1 (185.2 to 248.9)	214.5 (183.9 to 251.0)
Motor vehicle road injuries	3 352 (2 760 to 4 017)	9 756 (8 164 to 11 480)	156.3 (130.7 to 183.8)	266.4 (229.8 to 313.1)	5 998 (5 263 to 6 945)	22 044 (19 176 to 25 805)	355.0 (309.7 to 411.6)	618.6 (537.7 to 722.9)
Other road injuries	1 357 (1 041 to 1 713)	2 202 (1 720 to 2 731)	63.5 (49.8 to 78.6)	61.5 (48.8 to 76.0)	2 574 (2 137 to 3 096)	5 314 (4 403 to 6 401)	146.5 (122.4 to 175.6)	150.0 (124.8 to 181.2)
Other transport injuries	416 (315 to 563)	366 (275 to 483)	18.6 (14.8 to 23.9)	10.6 (7.9 to 14.0)	1 937 (1 602 to 2 300)	1 888 (1 610 to 2 175)	103.0 (86.7 to 119.3)	53.8 (46.2 to 61.9)
**Unintentional injuries**	**74 226 (68 388 to 80 619)**	**126 631 (116 372 to 138 297)**	**3 597.6 (3 318.6 to 3 904.0)**	**3 709.5 (3 404.8 to 4 051.8)**	**148 311 (138 173 to 160 489)**	**266 370 (245 614 to 293 435)**	**8 247.6 (7 689.0 to 8 920.5)**	**7 658.2 (7 073.1 to 8 446.1)**
Falls	35 733 (31 373 to 40 659)	80 660 (71 656 to 90 619)	1 902.6 (1 696.7 to 2 142.4)	2 410.1 (2 143.7 to 2 709.2)	64 151 (56 658 to 73 261)	161 479 (143 741 to 184 802)	3 791.5 (3 384.5 to 4 341.6)	4 719.6 (4 207.2 to 5 386.7)
Drowning	182 (156 to 211)	92 (73 to 116)	7.8 (6.8 to 8.9)	2.6 (2.1 to 3.3)	868 (755 to 991)	570 (495 to 666)	49.2 (43.4 to 56.2)	16.4 (14.2 to 19.0)
Fire, heat, and hot substances	3 004 (2 387 to 3 747)	2 866 (2 350 to 3 467)	115.9 (94.7 to 141.4)	83.6 (68.1 to 101.7)	29 940 (24 707 to 37 776)	34 852 (28 900 to 43 706)	1 488.0 (1 250.3 to 1 841.1)	959.2 (790.7 to 1 217.8)
Poisonings	912 (675 to 1 254)	1 008 (727 to 1 354)	32.6 (24.4 to 44.1)	30.4 (21.8 to 41.2)	848 (645 to 1 049)	1 266 (925 to 1 602)	37.8 (29.0 to 46.5)	35.1 (25.7 to 44.4)
Poisoning by carbon monoxide	188 (118 to 287)	158 (86 to 251)	7.2 (4.8 to 10.5)	4.6 (2.5 to 7.4)	137 (104 to 179)	129 (100 to 165)	6.3 (4.8 to 8.1)	3.6 (2.8 to 4.6)
Poisoning by other means	724 (519 to 1 022)	851 (595 to 1 177)	25.4 (18.3 to 35.3)	25.7 (17.9 to 35.9)	711 (517 to 899)	1 137 (805 to 1 470)	31.5 (23.0 to 39.8)	31.5 (22.3 to 40.7)
Exposure to mechanical forces	17 221 (14 484 to 20 634)	18 794 (15 846 to 22 063)	779.3 (690.5 to 868.8)	518.5 (440.5 to 611.0)	34 505 (30 182 to 39 715)	47 128 (40 939 to 55 082)	1 923.1 (1 693.2 to 2 204.0)	1 316.9 (1 144.9 to 1 535.1)
Unintentional firearm injuries	1 321 (970 to 1 787)	767 (542 to 1 039)	55.1 (41.1 to 72.4)	21.1 (15.0 to 28.4)	4 867 (3 870 to 5 844)	3 971 (3 206 to 4 810)	275.6 (221.7 to 333.0)	111.2 (90.3 to 134.1)
Other exposure to mechanical forces	15 900 (13 290 to 18 946)	17 968 (15 208 to 21 240)	684.2 (583.3 to 805.0)	497.4 (421.0 to 586.8)	29 637 (25 085 to 35 097)	43 157 (36 752 to 51 404)	1 647.5 (1 410.7 to 1 935.6)	1 205.7 (1 030.8 to 1 430.1)
Adverse effects of medical treatment	2 081 (1 770 to 2 407)	4 824 (4 052 to 5 537)	107.5 (92.5 to 123.1)	137.6 (115.7 to 157.2)	159 (124 to 198)	369 (281 to 455)	8.2 (6.3 to 10.1)	10.5 (8.0 to 13.0)
Animal contact	4 970 (3 963 to 6 160)	6 143 (5 312 to 7 158)	219.3 (179.8 to 263.2)	175.5 (151.8 to 205.3)	5 444 (4 666 to 6 320)	7 886 (6 860 to 9 074)	291.8 (254.4 to 333.5)	223.3 (194.7 to 255.3)
Venomous animal contact	1 741 (1 287 to 2 329)	2 190 (1 735 to 2 712)	76.4 (57.1 to 99.1)	62.2 (49.2 to 77.2)	1 176 (964 to 1 393)	1 671 (1 410 to 1 930)	59.4 (49.6 to 69.3)	46.9 (39.7 to 54.2)
Non-venomous animal contact	3 230 (2 507 to 4 088)	3 953 (3 304 to 4 770)	143.0 (114.8 to 176.2)	113.3 (94.9 to 137.0)	4 268 (3 530 to 5 109)	6 215 (5 234 to 7 333)	232.4 (195.7 to 273.6)	176.3 (149.2 to 207.6)
Foreign body	5 284 (3 314 to 7 804)	8 160 (4 629 to 12 768)	254.7 (141.9 to 382.1)	233.2 (132.5 to 360.9)	2 947 (2 349 to 3 741)	3 951 (3 126 to 5 103)	151.2 (122.9 to 188.4)	113.2 (89.3 to 145.5)
Pulmonary aspiration and foreign body in airway	242 (171 to 333)	133 (96 to 187)	9.0 (6.6 to 12.1)	4.0 (2.9 to 5.6)	180 (127 to 247)	123 (98 to 154)	8.2 (6.0 to 11.1)	3.5 (2.8 to 4.3)
Foreign body in eyes	4 472 (2 346 to 6 992)	7 446 (3 901 to 12 105)	222.4 (109.3 to 352.2)	212.2 (111.3 to 341.0)	881 (459 to 1 484)	1 611 (913 to 2 625)	50.0 (28.6 to 78.4)	47.2 (27.1 to 75.4)
Foreign body in other body part	571 (410 to 828)	581 (457 to 733)	23.2 (17.2 to 31.6)	17.1 (13.4 to 21.5)	1 886 (1 500 to 2 412)	2 216 (1 825 to 2 752)	93.1 (75.5 to 116.9)	62.5 (51.5 to 77.9)
Environmental heat and cold exposure	940 (666 to 1 282)	474 (359 to 633)	43.7 (33.3 to 55.4)	14.1 (10.8 to 18.8)	3 405 (2 758 to 4 156)	1 882 (1 559 to 2 189)	181.3 (150.8 to 215.4)	52.9 (44.9 to 62.3)
Exposure to forces of nature	166 (136 to 208)	176 (149 to 211)	6.5 (5.4 to 7.9)	4.9 (4.2 to 6.0)	528 (378 to 723)	1 357 (938 to 1 885)	23.0 (16.7 to 31.4)	36.8 (25.5 to 51.0)
Other unintentional injuries	3 730 (3 006 to 4 604)	3 492 (2 827 to 4 441)	167.7 (138.3 to 203.0)	98.9 (79.2 to 125.5)	5 520 (4 708 to 6 381)	6 185 (5 355 to 7 076)	302.6 (261.8 to 346.2)	174.4 (151.5 to 198.5)

Cause	Years lived with disability: All-age counts (95% UI) 1990	Years lived with disability: All-age counts (95% UI) 2017	Years lived with disability: Age-standardised rates per 100,000 (95% UI) 1990	Years lived with disability: Age-standardised rates per 100,000 (95% UI) 2017	Disability adjusted life years: All-age counts (95% UI) 1990	Disability adjusted life years: All-age counts (95% UI) 2017	Disability adjusted life years: Age-standardised rates per 100,000 (95% UI) 1990	Disability adjusted life years: Age-standardised rates per 100,000 (95% UI) 2017
**Transport and unintentional injuries**	**7 409 (5 379 to 9 802)**	**13 610 (9 820 to 18 434)**	**409.7 (297.8 to 538.9)**	**390.4 (281.0 to 527.0)**	**78 594 (73 500 to 84 214)**	**60 573 (55 331 to 66 609)**	**3 343.1 (3 146.4 to 3 568.7)**	**1 703.7 (1 554.5 to 1 871.8)**
**Transport injuries**	**1 427 (1 045 to 1 892)**	**3 126 (2 258 to 4 186)**	**78.8 (58.0 to 104.1)**	**87.2 (62.9 to 117.0)**	**36 772 (34 495 to 39 437)**	**29 648 (27 357 to 32 192)**	**1 544.9 (1 460.7 to 1 646.3)**	**813.0 (750.3 to 884.8)**
Road injuries	1 307 (956 to 1 737)	3 029 (2 185 to 4 059)	72.7 (53.7 to 96.1)	84.5 (60.9 to 113.1)	35 605 (33 385 to 38 184)	28 244 (26 045 to 30 674)	1 494.5 (1 412.5 to 1 593.4)	774.7 (713.7 to 842.3)
Pedestrian road injuries	358 (255 to 484)	678 (477 to 928)	20.0 (14.3 to 26.9)	19.2 (13.5 to 26.2)	15 726 (14 292 to 17 393)	11 245 (10 265 to 12 286)	675.2 (624.3 to 734.8)	313.1 (285.9 to 342.1)
Cyclist road injuries	132 (93 to 174)	285 (198 to 379)	7.0 (5.0 to 9.2)	8.0 (5.5 to 10.6)	831 (746 to 923)	1 143 (1 020 to 1 284)	36.5 (32.9 to 40.3)	31.5 (28.1 to 35.4)
Motorcyclist road injuries	259 (180 to 352)	460 (312 to 630)	14.1 (9.9 to 19.2)	12.6 (8.6 to 17.3)	5 051 (4 545 to 5 597)	3 419 (3 004 to 3 839)	206.1 (187.0 to 226.4)	91.9 (80.8 to 103.2)
Motor vehicle road injuries	418 (304 to 558)	1 352 (967 to 1 803)	23.8 (17.3 to 31.6)	37.6 (27.0 to 50.2)	13 020 (11 913 to 14 180)	11 538 (10 481 to 12 734)	534.8 (493.7 to 576.8)	313.5 (285.1 to 348.2)
Other road injuries	141 (101 to 191)	255 (182 to 354)	7.8 (5.6 to 10.5)	7.1 (5.1 to 9.9)	978 (829 to 1 132)	899 (778 to 1 042)	41.9 (35.9 to 47.9)	24.8 (21.5 to 28.7)
Other transport injuries	120 (84 to 160)	97 (69 to 131)	6.1 (4.4 to 8.2)	2.7 (1.9 to 3.7)	1 167 (1 051 to 1 291)	1 404 (1 241 to 1 591)	50.4 (45.9 to 55.5)	38.3 (33.9 to 43.3)
**Unintentional injuries**	**5 982 (4 328 to 7 985)**	**10 484 (7 438 to 14 378)**	**330.9 (238.6 to 441.8)**	**303.2 (214.6 to 415.6)**	**41 822 (38 410 to 45 573)**	**30 925 (27 440 to 35 016)**	**1 798.2 (1 664.3 to 1 954.1)**	**890.7 (788.5 to 1 010.3)**
Falls	3 699 (2 687 to 4 844)	7 916 (5 698 to 10 619)	213.3 (155.5 to 277.5)	231.2 (166.3 to 309.6)	7 377 (6 350 to 8 580)	11 239 (9 006 to 13 988)	414.7 (355.8 to 481.9)	328.9 (263.0 to 408.3)
Drowning	48 (33 to 67)	30 (20 to 41)	2.7 (1.9 to 3.7)	0.8 (0.6 to 1.2)	13 906 (12 680 to 15 546)	5 924 (5 308 to 6 559)	531.8 (488.5 to 586.1)	165.7 (148.2 to 184.2)
Fire, heat, and hot substances	630 (383 to 1 002)	648 (372 to 1 060)	30.5 (18.7 to 47.8)	18.1 (10.4 to 29.5)	3 419 (2 971 to 4 008)	1 880 (1 582 to 2 310)	147.7 (130.5 to 171.6)	53.7 (45.8 to 65.6)
Poisonings	100 (61 to 152)	148 (86 to 226)	4.3 (2.7 to 6.6)	4.1 (2.4 to 6.2)	1 652 (1 476 to 1 854)	904 (803 to 1 016)	64.7 (58.6 to 71.6)	25.4 (22.6 to 28.6)
Poisoning by carbon monoxide	15 (9 to 23)	13 (8 to 20)	0.6 (0.4 to 1.0)	0.4 (0.2 to 0.6)	752 (669 to 848)	422 (376 to 471)	29.6 (26.6 to 32.9)	11.8 (10.5 to 13.2)
Poisoning by other means	85 (51 to 132)	135 (77 to 210)	3.7 (2.2 to 5.7)	3.7 (2.1 to 5.8)	900 (784 to 1 033)	482 (412 to 565)	35.1 (31.1 to 39.8)	13.6 (11.6 to 16.0)
Exposure to mechanical forces	799 (550 to 1 144)	996 (667 to 1 460)	43.7 (29.9 to 62.9)	27.8 (18.6 to 40.7)	4 834 (4 445 to 5 317)	2 986 (2 602 to 3 470)	213.9 (196.1 to 237.5)	82.3 (71.5 to 95.7)
Unintentional firearm injuries	100 (66 to 151)	71 (45 to 114)	5.5 (3.6 to 8.4)	2.0 (1.3 to 3.2)	2 146 (1 940 to 2 368)	892 (698 to 1 021)	89.1 (80.8 to 98.3)	24.1 (19.0 to 27.6)
Other exposure to mechanical forces	699 (482 to 1 001)	925 (618 to 1 361)	38.2 (26.3 to 55.1)	25.8 (17.2 to 37.9)	2 688 (2 359 to 3 101)	2 093 (1 762 to 2 541)	124.8 (109.7 to 144.7)	58.1 (49.0 to 70.6)
Adverse effects of medical treatment	21 (13 to 32)	49 (30 to 74)	1.1 (0.7 to 1.7)	1.4 (0.9 to 2.1)	1 363 (1 179 to 1 592)	1 247 (1 081 to 1 402)	62.5 (55.2 to 70.9)	36.9 (32.0 to 43.2)
Animal contact	140 (93 to 201)	176 (117 to 254)	6.9 (4.6 to 9.8)	4.9 (3.3 to 7.1)	731 (615 to 877)	365 (303 to 449)	29.4 (25.2 to 35.0)	10.5 (8.6 to 12.9)
Venomous animal contact	76 (48 to 112)	91 (58 to 134)	3.4 (2.2 to 4.9)	2.5 (1.6 to 3.7)	508 (414 to 632)	195 (156 to 241)	19.4 (16.1 to 23.8)	5.6 (4.5 to 6.9)
Non-venomous animal contact	64 (39 to 102)	85 (49 to 140)	3.5 (2.1 to 5.5)	2.4 (1.4 to 4.0)	223 (179 to 274)	170 (129 to 233)	10.0 (8.1 to 12.4)	4.9 (3.7 to 6.7)
Foreign body	177 (121 to 251)	210 (137 to 314)	8.9 (6.1 to 12.5)	6.0 (3.9 to 9.1)	6 236 (5 318 to 7 252)	4 897 (4 175 to 5 787)	232.3 (202.2 to 264.7)	146.5 (123.6 to 174.4)
Pulmonary aspiration and foreign body in airway	9 (6 to 13)	5 (3 to 7)	0.4 (0.3 to 0.5)	0.1 (0.1 to 0.2)	5 802 (4 886 to 6 792)	4 574 (3 885 to 5 436)	213.7 (183.5 to 246.2)	137.4 (115.8 to 164.4)
Foreign body in eyes	59 (30 to 104)	108 (59 to 189)	3.4 (1.8 to 5.6)	3.2 (1.7 to 5.5)	59 (30 to 104)	108 (59 to 189)	3.4 (1.8 to 5.6)	3.2 (1.7 to 5.5)
Foreign body in other body part	109 (76 to 145)	97 (68 to 131)	5.1 (3.6 to 6.8)	2.7 (1.9 to 3.7)	369 (289 to 427)	215 (176 to 290)	15.2 (12.1 to 17.6)	6.0 (4.9 to 8.1)
Environmental heat and cold exposure	132 (88 to 188)	68 (44 to 99)	7.0 (4.7 to 10.0)	2.0 (1.3 to 2.9)	595 (532 to 667)	279 (246 to 318)	29.5 (26.4 to 32.9)	7.8 (6.9 to 8.9)
Exposure to forces of nature	24 (17 to 33)	42 (31 to 59)	1.1 (0.8 to 1.5)	1.2 (0.8 to 1.6)	88 (78 to 99)	175 (159 to 196)	3.6 (3.2 to 4.0)	4.9 (4.4 to 5.4)
Other unintentional injuries	211 (151 to 284)	200 (140 to 278)	11.4 (8.1 to 15.3)	5.6 (3.9 to 7.8)	1 617 (1 479 to 1 778)	1 031 (920 to 1 159)	68.1 (62.5 to 74.7)	28.1 (25.2 to 31.5)

Death, YLL, incidence, prevalence, YLD, and DALY counts and rates per 100,000 for all injuries in Tlaxcala in 1990 and 2017

Cause	Deaths				Years of life lost			
	All-age counts (95% UI)		Age-standardised rates per 100,000 (95% UI)		All-age counts (95% UI)		Age-standardised rates per 100,000 (95% UI)	
	1990	2017	1990	2017	1990	2017	1990	2017
**Transport and unintentional injuries**	**431 (414 to 450)**	**485 (460 to 519)**	**67.0 (65.1 to 69.2)**	**37.9 (35.3 to 40.5)**	**23 206 (21 948 to 24 594)**	**21 780 (20 016 to 23 557)**	**2 884.8 (2 765.1 to 3 015.9)**	**1 595.2 (1 467.3 to 1 725.3)**
**Transport injuries**	**205 (196 to 216)**	**249 (227 to 269)**	**30.4 (29.1 to 31.8)**	**18.6 (17.0 to 20.1)**	**11 393 (10 775 to 12 016)**	**12 413 (11 289 to 13 450)**	**1 443.5 (1 374.0 to 1 517.1)**	**891.3 (811.1 to 965.5)**
Road injuries	200 (191 to 210)	239 (218 to 259)	29.6 (28.3 to 31.0)	17.9 (16.3 to 19.4)	11 088 (10 495 to 11 735)	11 945 (10 872 to 12 951)	1 405.6 (1 337.6 to 1 475.8)	857.7 (781.8 to 929.6)
Pedestrian road injuries	104 (98 to 111)	105 (95 to 115)	16.3 (15.5 to 17.2)	8.2 (7.4 to 8.9)	5 521 (5 091 to 5 944)	4 719 (4 238 to 5 204)	706.2 (661.2 to 752.3)	346.1 (311.9 to 381.5)
Cyclist road injuries	5 (4 to 5)	9 (8 to 11)	0.7 (0.6 to 0.8)	0.7 (0.6 to 0.8)	268 (239 to 300)	444 (389 to 507)	35.0 (31.5 to 38.7)	32.3 (28.3 to 36.6)
Motorcyclist road injuries	26 (23 to 29)	29 (25 to 32)	3.5 (3.2 to 3.8)	2.0 (1.8 to 2.3)	1 597 (1 384 to 1 713)	1 637 (1 410 to 1 862)	190.7 (172.5 to 210.2)	114.4 (98.4 to 129.7)
Motor vehicle road injuries	60 (55 to 65)	90 (79 to 100)	8.3 (7.8 to 9.0)	6.5 (5.8 to 7.2)	3 455 (3 164 to 3 783)	4 833 (4 278 to 5 379)	436.0 (403.2 to 473.2)	342.6 (302.8 to 380.2)
Other road injuries	5 (5 to 6)	6 (5 to 7)	0.8 (0.7 to 0.9)	0.5 (0.4 to 0.5)	308 (260 to 357)	311 (269 to 362)	37.6 (32.3 to 42.9)	22.4 (19.4 to 25.9)
Other transport injuries	5 (5 to 6)	10 (8 to 11)	0.8 (0.7 to 0.9)	0.7 (0.6 to 0.8)	304 (271 to 340)	468 (407 to 531)	38.0 (34.3 to 42.0)	33.7 (29.3 to 38.1)
**Unintentional injuries**	**226 (214 to 237)**	**236 (219 to 253)**	**36.7 (35.3 to 38.0)**	**19.3 (18.0 to 20.6)**	**11 813 (10 928 to 12 741)**	**9 367 (8 528 to 10 290)**	**1 441.3 (1 362.2 to 1 521.9)**	**703.9 (642.1 to 771.7)**
Falls	59 (56 to 62)	86 (79 to 93)	12.8 (12.2 to 13.5)	7.6 (7.0 to 8.2)	1 923 (1 795 to 2 056)	2 129 (1 951 to 2 332)	309.8 (292.5 to 327.3)	169.8 (156.0 to 185.5)
Drowning	39 (36 to 43)	28 (26 to 31)	5.2 (4.8 to 5.5)	2.1 (1.9 to 2.3)	2 447 (2 225 to 2 684)	1 497 (1 330 to 1 683)	275.5 (254.0 to 298.1)	108.3 (96.2 to 121.6)
Fire, heat, and hot substances	18 (17 to 20)	14 (13 to 15)	3.0 (2.8 to 3.2)	1.2 (1.1 to 1.3)	949 (842 to 1 078)	494 (451 to 544)	112.0 (102.4 to 123.2)	37.7 (34.5 to 41.4)
Poisonings	12 (11 to 13)	9 (8 to 10)	1.5 (1.4 to 1.6)	0.7 (0.6 to 0.7)	736 (657 to 816)	443 (399 to 493)	81.4 (74.4 to 88.5)	32.4 (29.2 to 35.8)
Poisoning by carbon monoxide	5 (5 to 6)	5 (4 to 5)	0.7 (0.7 to 0.8)	0.4 (0.3 to 0.4)	336 (299 to 378)	237 (209 to 264)	38.2 (34.6 to 41.8)	17.2 (15.3 to 19.1)
Poisoning by other means	6 (6 to 7)	4 (4 to 4)	0.8 (0.7 to 0.9)	0.3 (0.3 to 0.3)	400 (353 to 454)	207 (184 to 234)	43.1 (39.0 to 47.8)	15.2 (13.5 to 17.2)
Exposure to mechanical forces	25 (24 to 27)	19 (17 to 20)	3.8 (3.6 to 4.1)	1.4 (1.3 to 1.5)	1 379 (1 278 to 1 491)	861 (776 to 949)	178.3 (165.8 to 191.6)	62.4 (56.4 to 68.7)
Unintentional firearm injuries	10 (9 to 11)	6 (4 to 6)	1.4 (1.3 to 1.6)	0.4 (0.3 to 0.5)	571 (505 to 652)	284 (226 to 332)	72.3 (64.5 to 82.3)	20.2 (16.1 to 23.5)
Other exposure to mechanical forces	15 (14 to 17)	13 (12 to 14)	2.4 (2.2 to 2.6)	1.0 (0.9 to 1.1)	808 (717 to 888)	577 (517 to 636)	106.1 (95.1 to 116.2)	42.2 (38.0 to 47.7)
Adverse effects of medical treatment	13 (11 to 14)	19 (17 to 23)	2.4 (2.0 to 2.6)	1.6 (1.4 to 1.9)	599 (474 to 677)	611 (534 to 791)	75.7 (61.9 to 83.7)	47.7 (41.9 to 61.2)
Animal contact	4 (4 to 5)	2 (2 to 3)	0.5 (0.5 to 0.6)	0.2 (0.2 to 0.2)	278 (236 to 329)	102 (90 to 116)	28.4 (24.9 to 32.9)	7.7 (6.8 to 8.8)
Venomous animal contact	2 (2 to 3)	1 (1 to 1)	0.2 (0.2 to 0.3)	0.1 (0.0 to 0.1)	161 (127 to 204)	35 (27 to 52)	15.0 (12.1 to 19.0)	2.7 (2.1 to 3.9)
Non-venomous animal contact	2 (1 to 2)	2 (1 to 2)	0.3 (0.2 to 0.3)	0.1 (0.1 to 0.2)	117 (84 to 137)	67 (53 to 78)	13.4 (9.8 to 15.4)	5.1 (4.0 to 5.9)
Foreign body	38 (34 to 42)	45 (40 to 51)	4.8 (4.5 to 5.2)	3.5 (3.1 to 3.9)	2 568 (2 229 to 2 992)	2 541 (2 154 to 3 031)	262.9 (233.6 to 293.5)	188.3 (159.5 to 224.4)
Pulmonary aspiration and foreign body in airway	37 (33 to 41)	44 (39 to 50)	4.7 (4.3 to 5.0)	3.4 (3.0 to 3.9)	2 474 (2 136 to 2 888)	2 488 (2 108 to 2 967)	252.5 (223.9 to 282.7)	184.5 (156.4 to 220.2)
Foreign body in eyes								
Foreign body in other body part	1 (1 to 2)	1 (1 to 2)	0.2 (0.2 to 0.2)	0.1 (0.1 to 0.1)	94 (77 to 111)	53 (43 to 98)	10.4 (8.7 to 12.1)	3.8 (3.1 to 7.0)
Environmental heat and cold exposure	5 (4 to 5)	3 (3 to 3)	0.8 (0.8 to 0.9)	0.2 (0.2 to 0.2)	210 (192 to 230)	105 (94 to 117)	29.0 (27.1 to 31.1)	7.9 (7.1 to 8.8)
Exposure to forces of nature	0 (0 to 0)	0 (0 to 0)	0.1 (0.1 to 0.1)	0.0 (0.0 to 0.0)	24 (22 to 27)	11 (10 to 12)	2.8 (2.6 to 3.1)	0.8 (0.7 to 0.9)
Other unintentional injuries	12 (11 to 13)	11 (10 to 12)	1.7 (1.6 to 1.8)	0.8 (0.7 to 0.9)	699 (638 to 764)	574 (504 to 647)	85.5 (78.7 to 93.0)	40.8 (35.9 to 45.8)

Cause	Incidence				Prevalence			
	All-age counts (95% UI)		Age-standardised rates per 100,000 (95% UI)		All-age counts (95% UI)		Age-standardised rates per 100,000 (95% UI)	
	1990	2017	1990	2017	1990	2017	1990	2017
**Transport and unintentional injuries**	**29 486 (27 037 to 32 140)**	**56 421 (51 663 to 61 991)**	**4 229.7 (3 860.8 to 4 637.5)**	**4 382.3 (4 006.8 to 4 808.8)**	**54 623 (51 413 to 58 378)**	**115 421 (108 483 to 123 856)**	**9 558.9 (9 019.4 to 10 244.8)**	**9 139.4 (8 590.7 to 9 818.5)**
**Transport injuries**	**2 755 (2 374 to 3 189)**	**7 534 (6 530 to 8 597)**	**385.9 (336.3 to 437.2)**	**555.7 (484.1 to 631.8)**	**6 546 (6 103 to 6 982)**	**20 700 (19 110 to 22 395)**	**1 191.9 (1 112.2 to 1 273.3)**	**1 621.7 (1 499.6 to 1 755.4)**
Road injuries	2 627 (2 257 to 3 056)	7 401 (6 399 to 8 459)	369.9 (321.8 to 422.6)	545.5 (474.7 to 621.8)	5 983 (5 578 to 6 405)	20 041 (18 457 to 21 711)	1 100.9 (1 025.5 to 1 182.1)	1 569.6 (1 448.4 to 1 700.3)
Pedestrian road injuries	427 (349 to 527)	929 (751 to 1 133)	63.1 (52.9 to 75.6)	69.7 (56.4 to 84.9)	1 559 (1 336 to 1 815)	4 209 (3 662 to 4 905)	289.4 (251.5 to 335.2)	333.5 (291.2 to 387.4)
Cyclist road injuries	417 (320 to 534)	1 111 (887 to 1 383)	53.6 (42.8 to 66.8)	82.3 (65.8 to 101.7)	690 (579 to 819)	2 445 (2 056 to 2 887)	119.0 (101.1 to 138.3)	189.3 (160.0 to 222.0)
Motorcyclist road injuries	319 (247 to 404)	563 (425 to 721)	42.8 (33.8 to 52.5)	40.7 (31.0 to 52.0)	1 129 (976 to 1 313)	2 518 (2 153 to 2 947)	204.3 (176.1 to 236.1)	194.2 (166.6 to 226.8)
Motor vehicle road injuries	1 032 (851 to 1 231)	4 043 (3 389 to 4 762)	150.1 (124.7 to 176.8)	295.7 (248.1 to 346.7)	1 820 (1 596 to 2 119)	9 061 (7 890 to 10 390)	346.1 (300.5 to 401.8)	710.0 (614.9 to 828.2)
Other road injuries	432 (323 to 553)	755 (586 to 942)	60.4 (46.5 to 74.3)	57.1 (44.5 to 71.4)	791 (659 to 950)	1 807 (1 519 to 2 183)	142.1 (118.9 to 170.4)	142.7 (120.0 to 171.4)
Other transport injuries	128 (95 to 175)	134 (108 to 164)	16.0 (12.8 to 20.3)	10.2 (8.3 to 12.5)	564 (465 to 679)	660 (566 to 755)	90.9 (76.6 to 106.3)	52.0 (45.0 to 59.2)
**Unintentional injuries**	**26 731 (24 371 to 29 381)**	**48 886 (44 239 to 54 303)**	**3 843.8 (3 478.6 to 4 257.5)**	**3 826.5 (3 455.7 to 4 252.7)**	**48 077 (44 900 to 51 786)**	**94 720 (87 765 to 102 989)**	**8 367.0 (7 839.2 to 9 036.3)**	**7 517.7 (6 976.8 to 8 191.6)**
Falls	11 916 (10 458 to 13 545)	26 564 (23 609 to 30 092)	1 856.8 (1 650.3 to 2 086.0)	2 135.0 (1 898.9 to 2 406.9)	20 269 (18 034 to 23 235)	51 479 (45 987 to 58 562)	3 726.5 (3 338.6 to 4 259.8)	4 170.8 (3 725.0 to 4 760.8)
Drowning	36 (31 to 42)	28 (23 to 36)	4.6 (4.0 to 5.3)	2.1 (1.7 to 2.7)	160 (141 to 185)	169 (148 to 198)	29.1 (25.7 to 33.3)	13.5 (11.8 to 15.7)
Fire, heat, and hot substances	1 041 (835 to 1 294)	1 150 (947 to 1 387)	110.8 (91.2 to 134.2)	86.0 (71.0 to 104.2)	9 425 (7 883 to 11 615)	13 181 (10 959 to 16 805)	1 457.6 (1 237.2 to 1 751.7)	1 002.4 (836.2 to 1 266.6)
Poisonings	348 (259 to 471)	437 (321 to 585)	33.3 (25.5 to 43.7)	32.6 (23.9 to 43.7)	282 (217 to 365)	506 (373 to 635)	38.2 (29.7 to 46.7)	37.3 (27.6 to 46.8)
Poisoning by carbon monoxide	65 (49 to 88)	63 (41 to 93)	7.0 (5.0 to 9.9)	4.7 (3.1 to 6.9)	42 (32 to 54)	46 (36 to 60)	5.9 (4.6 to 7.4)	3.5 (2.7 to 4.5)
Poisoning by other means	283 (206 to 383)	374 (267 to 505)	26.4 (19.6 to 35.4)	27.8 (19.9 to 37.8)	240 (174 to 303)	459 (325 to 587)	32.3 (23.9 to 40.7)	33.9 (24.0 to 43.2)
Exposure to mechanical forces	5 493 (4 667 to 6 578)	7 604 (6 522 to 8 929)	714.9 (617.2 to 844.8)	557.0 (479.7 to 651.1)	10 508 (9 218 to 12 118)	18 149 (15 878 to 20 956)	1 876.7 (1 646.4 to 2 153.3)	1 409.9 (1 233.7 to 1 627.5)
Unintentional firearm injuries	362 (259 to 494)	316 (225 to 426)	45.5 (33.4 to 60.6)	23.0 (16.5 to 30.7)	1 332 (1 067 to 1 591)	1 563 (1 262 to 1 882)	240.8 (194.0 to 290.3)	122.8 (99.5 to 147.0)
Other exposure to mechanical forces	5 130 (4 351 to 6 162)	7 289 (6 266 to 8 560)	669.4 (577.1 to 789.4)	534.0 (461.3 to 626.1)	9 176 (7 808 to 10 793)	16 585 (14 238 to 19 516)	1 636.0 (1 402.2 to 1 912.1)	1 287.1 (1 106.1 to 1 513.7)
Adverse effects of medical treatment	602 (508 to 696)	1 496 (1 264 to 1 720)	94.6 (81.9 to 108.7)	116.6 (98.4 to 133.0)	46 (36 to 58)	115 (88 to 142)	7.2 (5.6 to 9.0)	8.9 (6.9 to 11.0)
Animal contact	2 561 (2 156 to 3 110)	4 126 (3 622 to 4 754)	325.1 (281.7 to 379.0)	315.0 (277.7 to 360.3)	2 588 (2 225 to 2 999)	4 922 (4 341 to 5 573)	430.5 (376.9 to 489.0)	387.3 (342.2 to 436.5)
Venomous animal contact	979 (751 to 1 259)	1 695 (1 362 to 2 079)	123.0 (97.0 to 153.6)	129.1 (104.2 to 158.1)	633 (518 to 759)	1 275 (1 066 to 1 487)	99.4 (82.7 to 116.6)	98.9 (83.0 to 115.1)
Non-venomous animal contact	1 588 (1 292 to 1 972)	2 431 (2 047 to 2 911)	202.2 (167.8 to 244.4)	185.9 (156.6 to 222.3)	1 956 (1 617 to 2 354)	3 647 (3 077 to 4 306)	331.1 (278.8 to 389.5)	288.2 (244.4 to 338.2)
Foreign body	2 866 (1 543 to 4 625)	5 633 (2 604 to 9 855)	456.6 (212.3 to 771.4)	442.5 (197.7 to 765.1)	1 233 (899 to 1 651)	1 997 (1 391 to 2 762)	203.9 (157.0 to 272.9)	155.4 (111.8 to 218.0)
Pulmonary aspiration and foreign body in airway	85 (60 to 118)	54 (40 to 74)	8.6 (6.3 to 11.4)	4.1 (3.1 to 5.6)	55 (40 to 76)	45 (37 to 55)	7.8 (5.8 to 10.3)	3.5 (2.9 to 4.2)
Foreign body in eyes	2 563 (1 282 to 4 337)	5 382 (2 361 to 9 601)	423.9 (176.3 to 739.2)	423.2 (179.3 to 746.0)	519 (267 to 898)	1 173 (646 to 1 972)	97.1 (54.7 to 159.9)	96.5 (54.8 to 157.2)
Foreign body in other body part	218 (146 to 334)	198 (139 to 263)	24.1 (16.9 to 34.6)	15.2 (10.6 to 20.0)	658 (521 to 845)	719 (591 to 897)	99.1 (80.3 to 124.4)	55.5 (45.7 to 68.9)
Environmental heat and cold exposure	386 (274 to 528)	254 (182 to 357)	50.7 (38.1 to 64.2)	20.0 (14.5 to 28.2)	1 308 (1 062 to 1 599)	996 (830 to 1 200)	214.6 (179.5 to 255.4)	78.7 (65.7 to 94.9)
Exposure to forces of nature	63 (52 to 79)	15 (12 to 18)	7.2 (6.1 to 8.7)	1.1 (0.9 to 1.3)	275 (196 to 382)	572 (399 to 797)	38.3 (27.5 to 53.4)	43.6 (30.5 to 60.7)
Other unintentional injuries	1 418 (1 148 to 1 755)	1 579 (1 325 to 1 876)	189.0 (158.0 to 225.9)	118.6 (99.4 to 140.5)	1 982 (1 702 to 2 296)	2 698 (2 338 to 3 080)	344.3 (297.7 to 395.2)	210.2 (183.1 to 239.6)

Cause	Years lived with disability				Disability adjusted life years			
	All-age counts (95% UI)		Age-standardised rates per 100,000 (95% UI)		All-age counts (95% UI)		Age-standardised rates per 100,000 (95% UI)	
	1990	2017	1990	2017	1990	2017	1990	2017
**Transport and unintentional injuries**	**2 452 (1 785 to 3 264)**	**5 015 (3 631 to 6 818)**	**423.5 (307.3 to 559.8)**	**396.7 (287.0 to 536.3)**	**25 658 (24 230 to 27 196)**	**26 795 (24 520 to 29 091)**	**3 308.3 (3 137.7 to 3 482.2)**	**1 991.9 (1 822.4 to 2 164.6)**
**Transport injuries**	**449 (330 to 587)**	**1 266 (913 to 1 692)**	**79.1 (58.4 to 104.0)**	**98.1 (70.9 to 131.1)**	**11 842 (11 217 to 12 510)**	**13 680 (12 502 to 14 845)**	**1 522.7 (1 450.4 to 1 601.8)**	**989.4 (905.0 to 1 072.7)**
Road injuries	413 (303 to 541)	1 231 (889 to 1 647)	73.5 (54.0 to 95.7)	95.3 (68.7 to 127.7)	11 501 (10 892 to 12 163)	13 176 (12 049 to 14 309)	1 479.1 (1 410.8 to 1 555.9)	953.0 (873.8 to 1 033.8)
Pedestrian road injuries	111 (78 to 149)	268 (188 to 362)	20.0 (14.2 to 26.6)	21.0 (14.8 to 28.3)	5 632 (5 196 to 6 052)	4 987 (4 518 to 5 476)	726.2 (679.6 to 773.4)	367.0 (332.9 to 402.8)
Cyclist road injuries	48 (34 to 63)	147 (103 to 195)	8.1 (5.7 to 10.6)	11.2 (7.9 to 15.0)	317 (284 to 351)	591 (519 to 663)	43.1 (38.8 to 47.4)	43.5 (38.3 to 48.8)
Motorcyclist road injuries	79 (55 to 106)	154 (105 to 210)	13.8 (9.6 to 18.8)	11.8 (8.0 to 16.1)	1 615 (1 459 to 1 798)	1 791 (1 560 to 2 029)	204.5 (185.7 to 224.4)	126.1 (110.3 to 142.8)
Motor vehicle road injuries	130 (94 to 172)	575 (412 to 768)	23.9 (17.3 to 31.5)	44.4 (31.9 to 59.3)	3 585 (3 290 to 3 905)	5 407 (4 809 to 5 994)	459.9 (426.9 to 495.2)	387.0 (344.5 to 427.6)
Other road injuries	45 (32 to 61)	88 (63 to 122)	7.8 (5.7 to 10.5)	6.9 (4.9 to 9.5)	352 (301 to 403)	400 (349 to 457)	45.4 (39.2 to 51.3)	29.3 (25.6 to 33.3)
Other transport injuries	37 (26 to 49)	35 (25 to 48)	5.6 (4.0 to 7.4)	2.8 (2.0 to 3.7)	341 (308 to 379)	504 (442 to 568)	43.6 (39.7 to 47.8)	36.4 (32.1 to 41.0)
**Unintentional injuries**	**2 003 (1 441 to 2 686)**	**3 749 (2 693 to 5 125)**	**344.4 (248.7 to 462.6)**	**298.6 (214.5 to 408.4)**	**13 816 (12 826 to 14 899)**	**13 116 (11 775 to 14 571)**	**1 785.7 (1 667.6 to 1 919.5)**	**1 002.5 (901.3 to 1 115.0)**
Falls	1 210 (887 to 1 582)	2 648 (1 910 to 3 497)	216.6 (156.8 to 282.2)	213.4 (153.8 to 284.0)	3 133 (2 791 to 3 524)	4 772 (4 015 to 5 672)	526.4 (466.3 to 594.7)	383.2 (322.7 to 455.3)
Drowning	9 (6 to 12)	9 (6 to 13)	1.6 (1.1 to 2.2)	0.7 (0.5 to 1.0)	2 457 (2 235 to 2 702)	1 506 (1 340 to 1 691)	277.1 (255.6 to 299.9)	109.0 (96.9 to 122.3)
Fire, heat, and hot substances	205 (130 to 321)	256 (150 to 417)	30.6 (19.3 to 47.9)	19.4 (11.4 to 31.5)	1 153 (1 019 to 1 329)	750 (633 to 908)	142.6 (127.4 to 163.0)	57.1 (48.3 to 69.2)
Poisonings	33 (20 to 50)	59 (35 to 89)	4.4 (2.6 to 6.5)	4.3 (2.6 to 6.5)	769 (692 to 851)	502 (450 to 555)	85.7 (78.6 to 93.0)	36.7 (33.0 to 40.6)
Poisoning by carbon monoxide	4 (3 to 7)	5 (3 to 7)	0.6 (0.4 to 0.9)	0.3 (0.2 to 0.5)	340 (303 to 378)	241 (214 to 268)	38.8 (35.1 to 42.4)	17.5 (15.6 to 19.5)
Poisoning by other means	29 (17 to 44)	54 (32 to 82)	3.8 (2.3 to 5.7)	4.0 (2.4 to 6.0)	429 (380 to 482)	261 (229 to 298)	46.9 (42.2 to 51.8)	19.2 (16.8 to 21.9)
Exposure to mechanical forces	249 (172 to 355)	394 (267 to 576)	43.6 (30.0 to 62.3)	30.5 (20.6 to 44.6)	1 628 (1 501 to 1 785)	1 254 (1 098 to 1 455)	221.9 (203.6 to 244.5)	92.9 (81.2 to 108.2)
Unintentional firearm injuries	27 (18 to 41)	29 (18 to 45)	4.8 (3.1 to 7.4)	2.2 (1.4 to 3.5)	599 (532 to 681)	312 (250 to 364)	77.0 (69.0 to 87.8)	22.4 (18.0 to 26.0)
Other exposure to mechanical forces	221 (152 to 317)	365 (246 to 536)	38.8 (26.6 to 55.7)	28.3 (19.0 to 41.5)	1 029 (916 to 1 153)	942 (806 to 1 120)	144.9 (128.5 to 163.7)	70.5 (60.3 to 84.4)
Adverse effects of medical treatment	6 (4 to 9)	15 (9 to 23)	1.0 (0.6 to 1.4)	1.2 (0.7 to 1.8)	606 (480 to 684)	626 (550 to 808)	76.7 (63.0 to 84.5)	48.9 (43.1 to 62.5)
Animal contact	69 (46 to 98)	111 (74 to 159)	10.4 (7.0 to 14.8)	8.5 (5.7 to 12.2)	347 (298 to 402)	213 (179 to 265)	38.8 (33.7 to 45.1)	16.3 (13.2 to 20.2)
Venomous animal contact	39 (25 to 56)	60 (39 to 85)	5.3 (3.5 to 7.6)	4.5 (2.9 to 6.3)	200 (163 to 243)	94 (72 to 124)	20.3 (16.8 to 24.5)	7.1 (5.5 to 9.4)
Non-venomous animal contact	30 (18 to 48)	51 (30 to 84)	5.0 (3.1 to 8.0)	4.1 (2.4 to 6.6)	147 (110 to 175)	118 (92 to 152)	18.5 (13.8 to 22.4)	9.1 (7.1 to 11.8)
Foreign body	78 (51 to 116)	114 (69 to 183)	12.7 (8.3 to 19.0)	9.2 (5.6 to 14.8)	2 646 (2 302 to 3 024)	2 655 (2 259 to 3 156)	275.6 (246.0 to 306.6)	197.5 (168.2 to 234.5)
Pulmonary aspiration and foreign body in airway	3 (2 to 4)	2 (1 to 3)	0.4 (0.3 to 0.5)	0.1 (0.1 to 0.2)	2 477 (2 138 to 2 891)	2 490 (2 110 to 2 969)	252.9 (224.3 to 283.1)	184.7 (156.5 to 220.4)
Foreign body in eyes	34 (18 to 63)	78 (41 to 140)	6.5 (3.5 to 11.6)	6.4 (3.4 to 11.4)	34 (18 to 63)	78 (41 to 140)	6.5 (3.5 to 11.6)	6.4 (3.4 to 11.4)
Foreign body in other body part	41 (28 to 55)	34 (24 to 46)	5.8 (4.1 to 7.7)	2.6 (1.9 to 3.5)	134 (114 to 158)	87 (71 to 132)	16.2 (13.8 to 18.9)	6.4 (5.2 to 9.6)
Environmental heat and cold exposure	52 (34 to 78)	38 (25 to 54)	8.4 (5.6 to 11.8)	3.0 (2.0 to 4.3)	262 (234 to 292)	142 (125 to 163)	37.4 (33.8 to 41.6)	10.9 (9.5 to 12.5)
Exposure to forces of nature	13 (10 to 19)	19 (14 to 26)	1.9 (1.3 to 2.6)	1.4 (1.0 to 2.0)	38 (33 to 44)	30 (25 to 37)	4.7 (4.1 to 5.5)	2.2 (1.8 to 2.8)
Other unintentional injuries	78 (57 to 104)	91 (64 to 125)	13.3 (9.7 to 17.8)	7.1 (5.0 to 9.7)	777 (709 to 848)	665 (593 to 742)	98.8 (90.6 to 107.5)	47.8 (42.7 to 53.5)

Death, YLL, incidence, prevalence, YLD, and DALY counts and rates per 100,000 for all injuries in Veracruz de Ignacio de la Llave in 1990 and 2017

Cause	Deaths: All-age counts (95% UI) 1990	Deaths: All-age counts (95% UI) 2017	Deaths: Age-standardised rates per 100,000 (95% UI) 1990	Deaths: Age-standardised rates per 100,000 (95% UI) 2017	Years of life lost: All-age counts (95% UI) 1990	Years of life lost: All-age counts (95% UI) 2017	Years of life lost: Age-standardised rates per 100,000 (95% UI) 1990	Years of life lost: Age-standardised rates per 100,000 (95% UI) 2017
**Transport and unintentional injuries**	**3 103 (2 962 to 3 258)**	**2 603 (2 425 to 2 795)**	**55.5 (53.7 to 57.4)**	**30.8 (28.7 to 33.0)**	**172 066 (161 440 to 184 472)**	**110 061 (101 891 to 119 463)**	**2 592.4 (2 463.6 to 2 737.5)**	**1 300.8 (1 204.2 to 1 413.3)**
**Transport injuries**	**1 337 (1 265 to 1 414)**	**1 124 (1 030 to 1 225)**	**23.3 (22.2 to 24.3)**	**13.1 (12.0 to 14.3)**	**74 535 (69 679 to 79 621)**	**53 971 (49 251 to 58 950)**	**1 141.3 (1 076.3 to 1 210.8)**	**632.1 (577.4 to 689.9)**
Road injuries	1 283 (1 213 to 1 356)	1 072 (983 to 1 167)	22.3 (21.3 to 23.3)	12.5 (11.5 to 13.6)	71 557 (66 884 to 76 550)	51 573 (47 055 to 56 333)	1 094.9 (1 032.7 to 1 160.1)	604.1 (551.1 to 659.4)
Pedestrian road injuries	664 (621 to 708)	420 (382 to 460)	12.2 (11.5 to 12.8)	4.9 (4.5 to 5.4)	35 909 (32 911 to 38 999)	17 763 (16 041 to 19 570)	557.2 (518.0 to 597.0)	208.9 (188.9 to 230.0)
Cyclist road injuries	31 (28 to 34)	44 (38 to 49)	0.5 (0.5 to 0.6)	0.5 (0.4 to 0.6)	1 725 (1 525 to 1 926)	1 962 (1 731 to 2 227)	26.9 (23.9 to 29.8)	22.9 (20.2 to 26.0)
Motorcyclist road injuries	173 (155 to 192)	164 (144 to 191)	2.8 (2.5 to 3.0)	1.9 (1.7 to 2.2)	10 119 (9 022 to 11 333)	9 161 (7 954 to 10 724)	151.4 (136.2 to 168.6)	106.8 (92.9 to 124.8)
Motor vehicle road injuries	391 (358 to 427)	425 (384 to 472)	6.4 (5.9 to 7.0)	4.9 (4.5 to 5.5)	22 447 (20 418 to 24 626)	21 790 (19 486 to 24 349)	339.0 (310.3 to 370.6)	255.0 (227.9 to 285.0)
Other road injuries	24 (21 to 28)	19 (17 to 22)	0.4 (0.4 to 0.5)	0.2 (0.2 to 0.3)	1 358 (1 136 to 1 603)	896 (764 to 1 038)	20.4 (17.4 to 23.8)	10.5 (8.9 to 12.2)
Other transport injuries	54 (50 to 59)	52 (45 to 60)	1.0 (0.9 to 1.1)	0.6 (0.5 to 0.7)	2 978 (2 698 to 3 283)	2 398 (2 036 to 2 799)	46.4 (42.5 to 50.8)	28.0 (23.8 to 32.7)
**Unintentional injuries**	**1 766 (1 677 to 1 864)**	**1 478 (1 383 to 1 589)**	**32.3 (31.1 to 33.5)**	**17.7 (16.5 to 19.0)**	**97 531 (90 226 to 105 864)**	**56 091 (52 028 to 61 017)**	**1 451.1 (1 365.8 to 1 543.6)**	**668.7 (621.3 to 728.4)**
Falls	347 (331 to 365)	475 (434 to 512)	8.4 (8.0 to 8.8)	5.7 (5.2 to 6.2)	13 114 (12 302 to 14 060)	11 535 (10 434 to 12 573)	245.6 (233.1 to 259.1)	135.4 (122.7 to 147.5)
Drowning	542 (508 to 580)	302 (275 to 329)	8.6 (8.2 to 9.1)	3.6 (3.2 to 3.9)	33 090 (30 565 to 35 921)	15 085 (13 613 to 16 572)	466.6 (435.9 to 501.0)	179.0 (162.0 to 197.4)
Fire, heat, and hot substances	137 (124 to 152)	91 (83 to 97)	2.5 (2.3 to 2.7)	1.1 (1.0 to 1.2)	7 779 (6 753 to 8 985)	2 820 (2 562 to 3 069)	111.6 (99.8 to 125.8)	33.7 (30.7 to 36.8)
Poisonings	75 (69 to 82)	44 (39 to 48)	1.2 (1.1 to 1.3)	0.5 (0.5 to 0.6)	4 688 (4 229 to 5 216)	1 994 (1 783 to 2 199)	65.1 (59.7 to 71.4)	23.8 (21.2 to 26.3)
Poisoning by carbon monoxide	21 (19 to 23)	14 (13 to 16)	0.3 (0.3 to 0.4)	0.2 (0.1 to 0.2)	1 288 (1 133 to 1 462)	640 (559 to 728)	18.1 (16.2 to 20.4)	7.6 (6.6 to 8.6)
Poisoning by other means	54 (49 to 59)	30 (27 to 33)	0.9 (0.8 to 0.9)	0.4 (0.3 to 0.4)	3 401 (3 047 to 3 829)	1 353 (1 205 to 1 507)	46.9 (42.7 to 51.8)	16.2 (14.4 to 18.1)
Exposure to mechanical forces	223 (210 to 237)	189 (173 to 207)	4.0 (3.8 to 4.2)	2.2 (2.0 to 2.4)	12 326 (11 440 to 13 255)	8 441 (7 706 to 9 326)	190.0 (178.1 to 202.3)	98.6 (90.0 to 108.9)
Unintentional firearm injuries	87 (77 to 95)	68 (56 to 79)	1.5 (1.3 to 1.6)	0.8 (0.7 to 0.9)	4 970 (4 389 to 5 462)	3 369 (2 777 to 3 907)	75.1 (66.1 to 83.9)	39.3 (32.4 to 45.6)
Other exposure to mechanical forces	136 (125 to 146)	121 (109 to 135)	2.5 (2.3 to 2.7)	1.4 (1.3 to 1.6)	7 356 (6 642 to 8 023)	5 072 (4 527 to 5 764)	114.9 (105.2 to 124.2)	59.3 (53.0 to 67.4)
Adverse effects of medical treatment	77 (70 to 95)	96 (84 to 141)	1.6 (1.5 to 2.0)	1.2 (1.0 to 1.7)	3 774 (3 282 to 4 629)	2 791 (2 335 to 4 448)	58.2 (51.9 to 71.3)	32.9 (28.0 to 53.8)
Animal contact	41 (37 to 46)	24 (21 to 26)	0.7 (0.6 to 0.8)	0.3 (0.3 to 0.3)	2 544 (2 190 to 2 949)	875 (789 to 972)	35.0 (30.9 to 39.8)	10.6 (9.5 to 11.7)
Venomous animal contact	30 (26 to 34)	16 (13 to 18)	0.5 (0.4 to 0.6)	0.2 (0.2 to 0.2)	1 909 (1 623 to 2 272)	576 (487 to 655)	25.9 (22.6 to 30.0)	7.0 (5.9 to 8.0)
Non-venomous animal contact	11 (9 to 13)	8 (7 to 11)	0.2 (0.2 to 0.2)	0.1 (0.1 to 0.1)	635 (490 to 761)	299 (257 to 397)	9.2 (7.1 to 10.7)	3.6 (3.1 to 4.7)
Foreign body	210 (189 to 236)	186 (172 to 204)	3.3 (3.0 to 3.6)	2.3 (2.1 to 2.5)	13 981 (12 219 to 16 146)	9 316 (8 288 to 10 722)	183.8 (163.8 to 208.0)	115.5 (101.9 to 134.2)
Pulmonary aspiration and foreign body in airway	197 (176 to 222)	180 (166 to 197)	3.1 (2.8 to 3.4)	2.2 (2.0 to 2.4)	13 087 (11 378 to 15 153)	9 014 (7 988 to 10 336)	171.8 (152.0 to 194.6)	111.9 (98.5 to 129.9)
Foreign body in eyes								
Foreign body in other body part	13 (9 to 16)	6 (5 to 9)	0.2 (0.1 to 0.2)	0.1 (0.1 to 0.1)	894 (601 to 1 069)	302 (251 to 472)	12.0 (8.1 to 14.1)	3.6 (3.0 to 5.7)
Environmental heat and cold exposure	38 (35 to 41)	20 (17 to 22)	0.8 (0.7 to 0.8)	0.2 (0.2 to 0.3)	1 932 (1 678 to 2 080)	683 (597 to 763)	30.1 (27.8 to 32.8)	7.9 (6.9 to 8.8)
Exposure to forces of nature	3 (2 to 4)	1 (1 to 1)	0.0 (0.0 to 0.1)	0.0 (0.0 to 0.0)	177 (134 to 229)	68 (62 to 74)	2.6 (2.0 to 3.4)	0.8 (0.7 to 0.9)
Other unintentional injuries	73 (68 to 78)	52 (47 to 58)	1.2 (1.1 to 1.3)	0.6 (0.5 to 0.7)	4 231 (3 916 to 4 573)	2 593 (2 307 to 2 931)	62.5 (58.2 to 67.1)	30.4 (27.1 to 34.3)

Cause	Incidence: All-age counts (95% UI) 1990	Incidence: All-age counts (95% UI) 2017	Incidence: Age-standardised rates per 100,000 (95% UI) 1990	Incidence: Age-standardised rates per 100,000 (95% UI) 2017	Prevalence: All-age counts (95% UI) 1990	Prevalence: All-age counts (95% UI) 2017	Prevalence: Age-standardised rates per 100,000 (95% UI) 1990	Prevalence: Age-standardised rates per 100,000 (95% UI) 2017
**Transport and unintentional injuries**	**219 351 (200 607 to 240 139)**	**367 165 (335 612 to 402 097)**	**3 780.9 (3 452.7 to 4 154.1)**	**4 404.1 (4 032.9 to 4 819.2)**	**432 213 (405 350 to 462 797)**	**819 829 (765 996 to 890 920)**	**8 850.2 (8 325.2 to 9 509.7)**	**9 586.3 (8 961.6 to 10 396.6)**
**Transport injuries**	**17 719 (15 144 to 20 755)**	**37 524 (32 497 to 42 801)**	**295.6 (256.9 to 342.2)**	**441.4 (381.9 to 503.9)**	**43 847 (40 993 to 46 669)**	**111 733 (104 087 to 119 958)**	**929.5 (868.9 to 992.8)**	**1 292.6 (1 204.2 to 1 387.1)**
Road injuries	16 796 (14 277 to 19 793)	36 831 (31 861 to 42 158)	281.2 (244.2 to 325.3)	433.1 (373.9 to 496.9)	39 571 (36 895 to 42 385)	108 052 (100 437 to 116 320)	846.2 (790.1 to 906.8)	1 249.7 (1 162.0 to 1 344.3)
Pedestrian road injuries	2 862 (2 290 to 3 549)	4 803 (3 926 to 5 776)	49.9 (41.0 to 60.4)	56.5 (46.2 to 67.9)	10 671 (9 133 to 12 525)	22 419 (19 593 to 26 134)	230.5 (199.4 to 269.1)	260.1 (227.1 to 302.9)
Cyclist road injuries	2 687 (2 032 to 3 526)	4 914 (3 921 to 6 151)	41.8 (32.8 to 53.7)	58.8 (46.7 to 73.5)	4 560 (3 833 to 5 393)	11 242 (9 530 to 13 137)	92.0 (78.0 to 108.9)	130.6 (110.7 to 152.6)
Motorcyclist road injuries	2 270 (1 769 to 2 882)	4 361 (3 355 to 5 549)	36.5 (28.8 to 44.9)	51.3 (39.5 to 65.2)	8 175 (7 077 to 9 456)	20 831 (17 783 to 24 347)	173.9 (148.8 to 199.0)	240.3 (204.9 to 280.8)
Motor vehicle road injuries	6 047 (4 874 to 7 318)	17 921 (14 968 to 21 156)	103.6 (85.5 to 123.9)	209.5 (175.2 to 247.1)	10 716 (9 338 to 12 458)	41 900 (36 452 to 49 017)	236.7 (206.2 to 276.3)	483.4 (420.9 to 565.9)
Other road injuries	2 930 (2 378 to 3 816)	4 832 (3 835 to 5 905)	49.4 (38.1 to 61.7)	57.0 (44.9 to 69.4)	5 450 (4 517 to 6 594)	11 660 (9 733 to 14 069)	115.2 (96.4 to 138.4)	135.3 (113.1 to 163.1)
Other transport injuries	924 (707 to 1 246)	693 (567 to 856)	14.4 (11.7 to 18.2)	8.3 (6.8 to 10.3)	4 275 (3 593 to 5 062)	3 681 (3 159 to 4 293)	83.3 (70.1 to 97.3)	42.9 (36.9 to 49.3)
**Unintentional injuries**	**201 631 (183 800 to 222 663)**	**329 641 (298 090 to 361 006)**	**3 485.3 (3 149.1 to 3 861.9)**	**3 962.8 (3 592.7 to 4 387.6)**	**388 366 (362 814 to 419 230)**	**708 096 (655 223 to 776 008)**	**7 920.6 (7 398.0 to 8 567.9)**	**8 293.7 (7 671.1 to 9 093.0)**
Falls	91 462 (80 268 to 104 122)	198 831 (175 712 to 224 025)	1 701.5 (1 514.6 to 1 910.1)	2 402.7 (2 118.0 to 2 705.7)	163 250 (144 098 to 185 475)	403 385 (359 670 to 459 238)	3 522.3 (3 148.2 to 4 024.5)	4 747.5 (4 237.6 to 5 408.7)
Drowning	442 (380 to 510)	229 (185 to 287)	6.9 (6.0 to 7.9)	2.7 (2.2 to 3.4)	2 058 (1 805 to 2 364)	1 475 (1 296 to 1 697)	43.9 (38.7 to 50.2)	17.2 (15.1 to 19.7)
Fire, heat, and hot substances	8 335 (6 666 to 10 344)	7 584 (6 227 to 9 131)	110.2 (90.1 to 133.7)	94.4 (77.5 to 115.6)	81 803 (68 344 to 102 104)	95 470 (79 144 to 120 828)	1 480.1 (1 250.8 to 1 823.7)	1 117.1 (925.3 to 1 409.7)
Poisonings	2 456 (1 834 to 3 300)	2 495 (1 905 to 3 185)	30.0 (22.9 to 39.6)	32.1 (24.2 to 41.5)	2 169 (1 680 to 2 671)	3 059 (2 269 to 3 802)	35.3 (27.8 to 43.3)	36.1 (26.8 to 45.0)
Poisoning by carbon monoxide	459 (297 to 693)	353 (200 to 584)	6.1 (4.2 to 8.8)	4.4 (2.5 to 6.7)	316 (249 to 410)	276 (215 to 351)	5.3 (4.2 to 6.8)	3.2 (2.5 to 4.1)
Poisoning by other means	1 997 (1 455 to 2 723)	2 142 (1 582 to 2 838)	23.9 (17.7 to 32.1)	27.7 (20.4 to 36.9)	1 852 (1 347 to 2 333)	2 782 (1 975 to 3 512)	30.0 (22.1 to 37.8)	32.9 (23.3 to 41.6)
Exposure to mechanical forces	44 674 (37 556 to 52 612)	54 392 (46 942 to 63 207)	697.2 (597.6 to 810.3)	645.8 (557.5 to 751.8)	88 218 (77 328 to 101 979)	141 446 (123 576 to 163 840)	1 830.7 (1 611.8 to 2 100.0)	1 637.3 (1 431.0 to 1 899.0)
Unintentional firearm injuries	2 813 (2 029 to 3 793)	2 038 (1 476 to 2 716)	43.0 (31.5 to 56.3)	24.0 (17.4 to 31.9)	10 846 (8 692 to 12 983)	11 295 (9 086 to 13 762)	228.7 (185.1 to 274.0)	130.3 (104.9 to 158.6)
Other exposure to mechanical forces	41 862 (35 285 to 49 310)	52 354 (45 047 to 61 073)	654.2 (562.1 to 761.9)	621.9 (537.7 to 726.3)	77 372 (65 721 to 91 584)	130 151 (111 735 to 153 386)	1 602.0 (1 372.8 to 1 880.3)	1 507.0 (1 294.0 to 1 775.1)
Adverse effects of medical treatment	4 098 (3 421 to 4 811)	7 764 (6 539 to 9 035)	77.4 (65.6 to 90.0)	90.9 (77.0 to 106.0)	314 (239 to 398)	594 (454 to 747)	5.9 (4.5 to 7.4)	7.0 (5.3 to 8.7)
Animal contact	17 031 (14 145 to 20 613)	18 410 (15 986 to 21 298)	272.4 (231.5 to 319.3)	220.9 (192.7 to 255.8)	18 097 (15 582 to 21 053)	23 706 (20 777 to 27 020)	362.4 (316.5 to 413.9)	276.7 (242.4 to 315.6)
Venomous animal contact	5 867 (4 453 to 7 604)	6 002 (4 735 to 7 436)	93.9 (72.2 to 119.4)	71.8 (56.8 to 89.5)	3 763 (3 104 to 4 451)	4 224 (3 574 to 4 870)	70.9 (59.4 to 82.7)	49.4 (41.8 to 56.9)
Non-venomous animal contact	11 164 (8 980 to 13 813)	12 408 (10 523 to 14 773)	178.5 (147.6 to 216.8)	149.1 (126.4 to 177.1)	14 334 (11 874 to 17 202)	19 482 (16 580 to 22 826)	291.5 (246.0 to 342.9)	227.4 (193.7 to 266.5)
Foreign body	21 152 (10 777 to 33 798)	28 390 (12 134 to 52 666)	398.1 (185.9 to 656.7)	334.0 (145.0 to 631.7)	9 096 (6 926 to 12 208)	10 457 (7 650 to 14 841)	179.2 (137.6 to 239.3)	122.9 (89.9 to 173.3)
Pulmonary aspiration and foreign body in airway	516 (351 to 729)	227 (166 to 305)	6.5 (4.6 to 9.0)	2.8 (2.1 to 3.8)	361 (257 to 489)	203 (165 to 251)	6.0 (4.4 to 8.0)	2.4 (1.9 to 3.0)
Foreign body in eyes	19 096 (8 698 to 31 978)	27 092 (10 756 to 51 426)	369.7 (154.4 to 629.6)	318.0 (128.3 to 594.5)	3 825 (1 967 to 6 581)	6 118 (3 437 to 10 264)	83.9 (47.3 to 136.8)	72.0 (40.5 to 120.4)
Foreign body in other body part	1 539 (1 040 to 2 329)	1 071 (822 to 1 353)	21.9 (15.6 to 30.8)	13.2 (10.1 to 16.7)	4 910 (3 912 to 6 261)	4 136 (3 455 to 5 036)	89.3 (72.6 to 111.5)	48.5 (40.4 to 59.0)
Environmental heat and cold exposure	2 328 (1 704 to 3 068)	1 519 (1 245 to 1 793)	38.8 (30.4 to 48.0)	18.5 (15.2 to 21.8)	8 167 (6 678 to 9 919)	6 180 (5 306 to 7 310)	161.1 (135.2 to 191.4)	72.4 (62.1 to 85.8)
Exposure to forces of nature	205 (166 to 256)	91 (76 to 109)	3.1 (2.5 to 3.9)	1.1 (0.9 to 1.3)	1 314 (933 to 1 797)	3 471 (2 441 to 4 806)	20.5 (14.8 to 27.7)	40.3 (28.3 to 55.9)
Other unintentional injuries	9 454 (7 696 to 11 612)	9 987 (8 323 to 12 062)	149.6 (124.4 to 180.3)	119.6 (99.6 to 144.9)	13 885 (11 905 to 16 171)	18 857 (16 259 to 21 590)	279.2 (241.5 to 320.1)	219.2 (189.2 to 250.7)

Cause	Years lived with disability: All-age counts (95% UI) 1990	Years lived with disability: All-age counts (95% UI) 2017	Years lived with disability: Age-standardised rates per 100,000 (95% UI) 1990	Years lived with disability: Age-standardised rates per 100,000 (95% UI) 2017	Disability adjusted life years: All-age counts (95% UI) 1990	Disability adjusted life years: All-age counts (95% UI) 2017	Disability adjusted life years: Age-standardised rates per 100,000 (95% UI) 1990	Disability adjusted life years: Age-standardised rates per 100,000 (95% UI) 2017
**Transport and unintentional injuries**	**19 262 (14 126 to 25 565)**	**35 121 (25 419 to 47 323)**	**389.7 (285.4 to 518.3)**	**411.2 (297.9 to 554.0)**	**191 329 (179 087 to 204 988)**	**145 182 (132 526 to 159 590)**	**2 982.1 (2 813.9 to 3 174.3)**	**1 712.0 (1 563.2 to 1 882.0)**
**Transport injuries**	**3 042 (2 230 to 4 008)**	**6 780 (4 921 to 9 049)**	**62.3 (45.6 to 82.4)**	**78.3 (56.8 to 104.6)**	**77 578 (72 514 to 82 553)**	**60 751 (55 822 to 66 377)**	**1 203.6 (1 136.3 to 1 269.9)**	**710.4 (653.0 to 774.4)**
Road injuries	2 763 (2 036 to 3 637)	6 585 (4 782 to 8 790)	57.1 (41.9 to 75.4)	76.3 (55.2 to 101.6)	74 320 (69 485 to 79 127)	58 158 (53 497 to 63 641)	1 152.0 (1 086.7 to 1 216.6)	680.3 (626.1 to 743.3)
Pedestrian road injuries	776 (548 to 1 054)	1 419 (993 to 1 943)	16.1 (11.5 to 21.7)	16.4 (11.5 to 22.5)	36 685 (33 714 to 39 706)	19 182 (17 458 to 20 960)	573.3 (534.8 to 612.5)	225.3 (204.9 to 246.3)
Cyclist road injuries	324 (231 to 425)	677 (471 to 904)	6.3 (4.5 to 8.3)	7.9 (5.5 to 10.5)	2 048 (1 836 to 2 288)	2 640 (2 332 to 3 033)	33.2 (29.9 to 36.8)	30.8 (27.2 to 35.4)
Motorcyclist road injuries	576 (402 to 778)	1 273 (873 to 1 742)	11.7 (8.2 to 15.8)	14.7 (10.1 to 20.1)	10 694 (9 560 to 11 954)	10 434 (8 978 to 12 041)	163.2 (147.0 to 180.8)	121.5 (104.8 to 140.3)
Motor vehicle road injuries	775 (560 to 1 026)	2 637 (1 914 to 3 515)	16.5 (12.0 to 21.6)	30.4 (22.0 to 40.5)	23 223 (21 154 to 25 492)	24 427 (22 050 to 27 284)	355.5 (326.2 to 388.6)	285.3 (257.7 to 319.0)
Other road injuries	313 (224 to 425)	579 (413 to 800)	6.4 (4.6 to 8.7)	6.7 (4.8 to 9.3)	1 670 (1 420 to 1 940)	1 475 (1 255 to 1 735)	26.8 (23.2 to 30.7)	17.2 (14.6 to 20.2)
Other transport injuries	279 (198 to 371)	195 (140 to 262)	5.2 (3.7 to 6.9)	2.3 (1.6 to 3.0)	3 257 (2 960 to 3 578)	2 593 (2 224 to 2 994)	51.6 (47.3 to 56.4)	30.3 (26.0 to 35.0)
**Unintentional injuries**	**16 220 (11 780 to 21 486)**	**28 341 (20 427 to 38 518)**	**327.4 (237.4 to 435.2)**	**332.9 (239.8 to 452.5)**	**113 751 (105 298 to 123 453)**	**84 431 (75 202 to 94 957)**	**1 778.5 (1 646.4 to 1 916.1)**	**1 001.6 (892.4 to 1 123.9)**
Falls	9 902 (7 258 to 12 893)	20 791 (14 985 to 27 425)	207.6 (152.6 to 269.7)	244.8 (176.1 to 323.5)	23 016 (20 224 to 26 328)	32 325 (26 568 to 39 084)	453.2 (395.8 to 517.1)	380.2 (312.9 to 460.3)
Drowning	117 (81 to 161)	77 (52 to 107)	2.4 (1.7 to 3.3)	0.9 (0.6 to 1.2)	33 207 (30 678 to 36 042)	15 112 (13 682 to 16 643)	469.1 (438.2 to 503.2)	179.9 (162.9 to 198.3)
Fire, heat, and hot substances	1 793 (1 132 to 2 799)	1 858 (1 106 to 3 001)	31.4 (19.6 to 49.2)	21.8 (13.0 to 35.2)	9 572 (8 351 to 11 098)	4 678 (3 835 to 5 836)	143.0 (126.0 to 165.4)	55.5 (46.3 to 69.1)
Poisonings	257 (160 to 375)	349 (216 to 521)	4.1 (2.5 to 6.0)	4.1 (2.6 to 6.2)	4 940 (4 468 to 5 475)	2 343 (2 086 to 2 614)	69.1 (63.3 to 75.7)	27.9 (24.9 to 31.1)
Poisoning by carbon monoxide	34 (21 to 54)	27 (16 to 42)	0.5 (0.3 to 0.9)	0.3 (0.2 to 0.5)	1 317 (1 167 to 1 495)	667 (582 to 752)	18.7 (16.8 to 20.9)	7.9 (6.9 to 8.9)
Poisoning by other means	223 (136 to 333)	323 (193 to 489)	3.5 (2.2 to 5.2)	3.8 (2.3 to 5.8)	3 623 (3 249 to 4 057)	1 676 (1 490 to 1 913)	50.4 (45.9 to 55.6)	20.0 (17.8 to 22.9)
Exposure to mechanical forces	2 107 (1 452 to 3 000)	3 078 (2 086 to 4 481)	42.8 (29.3 to 61.1)	35.6 (24.2 to 51.9)	14 433 (13 335 to 15 719)	11 519 (10 216 to 13 129)	232.8 (215.1 to 254.8)	134.3 (119.2 to 152.9)
Unintentional firearm injuries	223 (147 to 339)	204 (129 to 328)	4.5 (3.0 to 6.9)	2.4 (1.5 to 3.8)	5 193 (4 605 to 5 690)	3 573 (2 946 to 4 092)	79.7 (70.4 to 86.7)	41.7 (34.4 to 47.7)
Other exposure to mechanical forces	1 884 (1 290 to 2 685)	2 874 (1 935 to 4 174)	38.3 (26.3 to 54.7)	33.3 (22.4 to 48.3)	9 240 (8 300 to 10 293)	7 946 (6 876 to 9 429)	153.1 (137.6 to 172.3)	92.6 (80.2 to 109.6)
Adverse effects of medical treatment	42 (26 to 64)	79 (49 to 119)	0.8 (0.5 to 1.2)	0.9 (0.6 to 1.4)	3 816 (3 323 to 4 668)	2 870 (2 414 to 4 517)	59.0 (52.7 to 72.1)	33.9 (28.9 to 54.9)
Animal contact	472 (337 to 657)	517 (341 to 737)	8.6 (5.8 to 12.1)	6.1 (4.0 to 8.6)	3 016 (2 628 to 3 466)	1 392 (1 195 to 1 622)	43.6 (38.3 to 49.2)	16.6 (14.3 to 19.3)
Venomous animal contact	247 (156 to 366)	238 (157 to 349)	4.1 (2.7 to 6.0)	2.8 (1.9 to 4.0)	2 156 (1 852 to 2 535)	814 (697 to 940)	30.0 (26.3 to 34.4)	9.8 (8.4 to 11.3)
Non-venomous animal contact	224 (137 to 353)	279 (168 to 442)	4.5 (2.8 to 7.0)	3.3 (2.0 to 5.2)	859 (689 to 1 046)	578 (458 to 768)	13.7 (10.9 to 16.6)	6.8 (5.4 to 9.0)
Foreign body	584 (381 to 870)	614 (377 to 990)	11.3 (7.5 to 16.8)	7.2 (4.4 to 11.6)	14 564 (12 784 to 16 718)	9 931 (8 835 to 11 375)	195.1 (174.4 to 219.4)	122.8 (108.6 to 141.5)
Pulmonary aspiration and foreign body in airway	20 (13 to 28)	9 (6 to 12)	0.3 (0.2 to 0.4)	0.1 (0.1 to 0.1)	13 107 (11 396 to 15 172)	9 023 (7 996 to 10 348)	172.1 (152.3 to 194.9)	112.0 (98.6 to 130.0)
Foreign body in eyes	255 (128 to 461)	409 (217 to 741)	5.6 (3.0 to 9.8)	4.8 (2.6 to 8.7)	255 (128 to 461)	409 (217 to 741)	5.6 (3.0 to 9.8)	4.8 (2.6 to 8.7)
Foreign body in other body part	309 (215 to 415)	196 (140 to 259)	5.3 (3.8 to 7.0)	2.3 (1.6 to 3.0)	1 203 (912 to 1 411)	498 (419 to 671)	17.4 (13.6 to 20.0)	5.9 (5.0 to 8.1)
Environmental heat and cold exposure	326 (220 to 459)	233 (153 to 335)	6.4 (4.3 to 9.0)	2.7 (1.8 to 3.9)	2 257 (1 955 to 2 384)	915 (793 to 1 038)	36.5 (33.4 to 40.0)	10.7 (9.3 to 12.1)
Exposure to forces of nature	66 (47 to 93)	115 (82 to 161)	1.0 (0.7 to 1.4)	1.3 (0.9 to 1.9)	243 (194 to 301)	183 (149 to 228)	3.7 (2.9 to 4.5)	2.2 (1.8 to 2.7)
Other unintentional injuries	556 (401 to 737)	629 (444 to 868)	10.9 (7.9 to 14.5)	7.3 (5.2 to 10.1)	4 787 (4 416 to 5 194)	3 222 (2 879 to 3 640)	73.4 (67.8 to 79.5)	37.7 (33.7 to 42.5)

Death, YLL, Incidence, prevalence, YLD, and DALY counts and rates per 100,000 for all injuries in Yucatán in 1990 and 2017

Cause	Deaths: All-age counts (95% UI) 1990	Deaths: All-age counts (95% UI) 2017	Deaths: Age-standardised rates per 100,000 (95% UI) 1990	Deaths: Age-standardised rates per 100,000 (95% UI) 2017
**Transport and unintentional injuries**	**646 (621 to 672)**	**730 (678 to 793)**	**58.4 (56.6 to 60.3)**	**34.2 (31.8 to 37.0)**
**Transport injuries**	**314 (299 to 329)**	**388 (353 to 429)**	**26.2 (25.0 to 27.3)**	**17.2 (15.7 to 19.0)**
Road injuries	298 (284 to 314)	360 (327 to 399)	24.8 (23.8 to 26.0)	16.0 (14.5 to 17.7)
Pedestrian road injuries	146 (137 to 155)	156 (142 to 174)	13.1 (12.4 to 13.8)	7.1 (6.5 to 7.9)
Cyclist road injuries	16 (15 to 18)	34 (30 to 39)	1.3 (1.2 to 1.4)	1.5 (1.3 to 1.7)
Motorcyclist road injuries	46 (42 to 51)	63 (55 to 72)	3.5 (3.2 to 3.8)	2.7 (2.3 to 3.1)
Motor vehicle road injuries	85 (78 to 92)	100 (88 to 112)	6.5 (6.0 to 7.1)	4.4 (3.8 to 4.9)
Other road injuries	6 (5 to 7)	7 (6 to 8)	0.5 (0.4 to 0.6)	0.3 (0.3 to 0.3)
Other transport injuries	15 (14 to 17)	27 (23 to 31)	1.3 (1.2 to 1.4)	1.2 (1.1 to 1.4)
**Unintentional injuries**	**332 (316 to 348)**	**341 (322 to 366)**	**32.3 (31.0 to 33.4)**	**17.0 (16.0 to 18.1)**
Falls	87 (83 to 92)	118 (108 to 127)	11.2 (10.6 to 11.8)	6.2 (5.7 to 6.7)
Drowning	57 (53 to 62)	41 (37 to 46)	4.3 (4.0 to 4.6)	1.9 (1.7 to 2.1)
Fire, heat, and hot substances	25 (24 to 27)	22 (20 to 23)	2.6 (2.5 to 2.7)	1.1 (1.0 to 1.2)
Poisonings	11 (10 to 12)	9 (8 to 10)	0.9 (0.8 to 0.9)	0.4 (0.4 to 0.5)
Poisoning by carbon monoxide	3 (2 to 3)	3 (2 to 3)	0.2 (0.2 to 0.2)	0.1 (0.1 to 0.1)
Poisoning by other means	9 (8 to 9)	7 (6 to 7)	0.7 (0.6 to 0.7)	0.3 (0.3 to 0.3)
Exposure to mechanical forces	33 (31 to 35)	26 (24 to 29)	2.8 (2.7 to 3.0)	1.2 (1.1 to 1.3)
Unintentional firearm injuries	12 (10 to 13)	7 (5 to 8)	0.9 (0.8 to 1.0)	0.3 (0.2 to 0.4)
Other exposure to mechanical forces	21 (20 to 23)	19 (17 to 22)	1.9 (1.8 to 2.0)	0.9 (0.8 to 1.0)
Adverse effects of medical treatment	28 (22 to 30)	38 (33 to 43)	3.1 (2.4 to 3.3)	2.0 (1.7 to 2.2)
Animal contact	8 (7 to 9)	5 (5 to 6)	0.7 (0.6 to 0.7)	0.3 (0.2 to 0.3)
Venomous animal contact	6 (6 to 7)	4 (3 to 4)	0.5 (0.4 to 0.6)	0.2 (0.2 to 0.2)
Non venomous animal contact	2 (1 to 2)	2 (1 to 2)	0.2 (0.1 to 0.2)	0.1 (0.1 to 0.1)
Foreign body	51 (46 to 57)	56 (51 to 62)	4.0 (3.7 to 4.3)	2.7 (2.5 to 3.0)
Pulmonary aspiration and foreign body in airway	49 (44 to 55)	55 (50 to 61)	3.8 (3.6 to 4.2)	2.7 (2.4 to 3.0)
Foreign body in eyes				
Foreign body in other body part	2 (1 to 2)	1 (1 to 2)	0.1 (0.1 to 0.2)	0.0 (0.0 to 0.1)
Environmental heat and cold exposure	9 (9 to 10)	6 (6 to 7)	1.0 (1.0 to 1.1)	0.3 (0.3 to 0.3)
Exposure to forces of nature	1 (1 to 1)	0 (0 to 0)	0.0 (0.0 to 0.1)	0.0 (0.0 to 0.0)
Other unintentional injuries	22 (20 to 24)	20 (18 to 23)	1.7 (1.6 to 1.9)	0.9 (0.8 to 1.0)

Cause	Years of life lost: All-age counts (95% UI) 1990	Years of life lost: All-age counts (95% UI) 2017	Years of life lost: Age-standardised rates per 100,000 (95% UI) 1990	Years of life lost: Age-standardised rates per 100,000 (95% UI) 2017
**Transport and unintentional injuries**	**32 011 (30 160 to 33 948)**	**30 114 (27 843 to 32 767)**	**2 308.6 (2 203.6 to 2 422.8)**	**1 340.8 (1 242.0 to 1 459.6)**
**Transport injuries**	**16 296 (15 378 to 17 317)**	**17 834 (16 189 to 19 790)**	**1 180.0 (1 121.1 to 1 245.6)**	**773.5 (702.7 to 855.6)**
Road injuries	15 511 (14 636 to 16 492)	16 591 (15 067 to 18 427)	1 123.4 (1 067.2 to 1 185.3)	719.6 (654.2 to 797.7)
Pedestrian road injuries	6 945 (6 406 to 7 539)	6 411 (5 779 to 7 159)	515.5 (481.2 to 552.7)	282.7 (255.4 to 314.0)
Cyclist road injuries	853 (760 to 951)	1 452 (1 267 to 1 674)	62.5 (56.3 to 69.4)	63.4 (55.4 to 72.9)
Motorcyclist road injuries	2 667 (2 400 to 2 971)	3 445 (3 003 to 3 976)	187.4 (170.3 to 207.0)	145.9 (127.3 to 168.0)
Motor vehicle road injuries	4 735 (4 345 to 5 188)	4 973 (4 345 to 5 605)	335.9 (309.6 to 367.4)	214.1 (187.4 to 241.3)
Other road injuries	312 (262 to 362)	310 (265 to 358)	22.1 (18.7 to 25.8)	13.5 (11.5 to 15.9)
Other transport injuries	785 (704 to 878)	1 242 (1 052 to 1 430)	56.6 (51.4 to 62.8)	53.9 (46.0 to 61.8)
**Unintentional injuries**	**15 715 (14 565 to 16 960)**	**12 280 (11 413 to 13 308)**	**1 128.6 (1 064.2 to 1 197.1)**	**567.3 (521.9 to 616.1)**
Falls	2 530 (2 354 to 2 673)	2 781 (2 504 to 3 035)	228.3 (216.8 to 240.6)	129.5 (117.3 to 141.2)
Drowning	3 296 (2 991 to 3 628)	2 001 (1 791 to 2 242)	219.8 (201.9 to 240.1)	89.5 (80.3 to 100.4)
Fire, heat, and hot substances	1 160 (1 037 to 1 311)	667 (605 to 731)	82.8 (75.7 to 91.5)	31.1 (28.2 to 34.0)
Poisonings	661 (593 to 745)	419 (375 to 466)	43.5 (39.6 to 48.2)	18.9 (16.9 to 21.0)
Poisoning by carbon monoxide	151 (134 to 172)	119 (103 to 136)	10.3 (9.1 to 11.5)	5.3 (4.6 to 6.1)
Poisoning by other means	510 (454 to 578)	300 (268 to 335)	33.3 (30.1 to 37.1)	13.6 (12.1 to 15.2)
Exposure to mechanical forces	1 679 (1 547 to 1 825)	1 122 (1 007 to 1 254)	123.1 (114.6 to 132.8)	49.0 (44.2 to 54.7)
Unintentional firearm injuries	630 (549 to 705)	331 (270 to 394)	45.3 (39.3 to 50.4)	14.2 (9.5 to 16.8)
Other exposure to mechanical forces	1 049 (946 to 1 160)	791 (701 to 906)	78.0 (71.3 to 85.2)	34.8 (31.1 to 39.8)
Adverse effects of medical treatment	1 118 (907 to 1 286)	1 096 (964 to 1 291)	84.0 (68.2 to 93.7)	52.5 (46.0 to 62.0)
Animal contact	483 (404 to 574)	204 (182 to 227)	30.8 (26.5 to 35.7)	9.6 (8.6 to 10.8)
Venomous animal contact	397 (321 to 481)	142 (117 to 164)	24.7 (20.6 to 29.1)	6.8 (5.6 to 7.9)
Non venomous animal contact	87 (69 to 104)	62 (51 to 83)	6.1 (4.8 to 7.2)	2.8 (2.4 to 3.8)
Foreign body	3 186 (2 747 to 3 726)	2 769 (2 422 to 3 216)	199.2 (175.3 to 228.2)	134.0 (115.7 to 157.9)
Pulmonary aspiration and foreign body in airway	3 078 (2 647 to 3 610)	2 712 (2 372 to 3 159)	192.2 (168.8 to 219.9)	131.5 (113.7 to 155.3)
Foreign body in eyes				
Foreign body in other body part	107 (90 to 137)	57 (43 to 125)	7.0 (6.0 to 8.9)	2.5 (1.9 to 5.5)
Environmental heat and cold exposure	387 (354 to 426)	222 (194 to 247)	31.0 (28.7 to 33.5)	9.9 (8.7 to 11.1)
Exposure to forces of nature	40 (36 to 44)	18 (17 to 20)	2.6 (2.4 to 2.8)	0.8 (0.8 to 0.9)
Other unintentional injuries	1 196 (1 090 to 1 313)	982 (857 to 1 129)	83.5 (76.5 to 91.3)	42.4 (37.1 to 48.5)

Cause	Incidence: All-age counts (95% UI) 1990	Incidence: All-age counts (95% UI) 2017	Incidence: Age-standardised rates per 100,000 (95% UI) 1990	Incidence: Age-standardised rates per 100,000 (95% UI) 2017
**Transport and unintentional injuries**	**43 335 (40 073 to 46 741)**	**69 144 (62 836 to 76 141)**	**3 424.2 (3 177.1 to 3 685.9)**	**3 211.4 (2 916.9 to 3 542.4)**
**Transport injuries**	**4 202 (3 626 to 4 870)**	**9 454 (8 178 to 10 895)**	**325.1 (282.8 to 370.5)**	**417.2 (362.0 to 480.9)**
Road injuries	3 868 (3 280 to 4 512)	9 030 (7 734 to 10 451)	300.2 (259.3 to 344.9)	397.5 (340.4 to 459.6)
Pedestrian road injuries	604 (485 to 738)	982 (771 to 1 226)	49.5 (40.9 to 59.8)	43.8 (34.6 to 54.5)
Cyclist road injuries	889 (702 to 1 129)	1 845 (1 467 to 2 280)	65.8 (52.8 to 81.6)	82.9 (65.9 to 102.5)
Motorcyclist road injuries	528 (417 to 673)	1 280 (988 to 1 621)	39.7 (31.9 to 49.4)	55.4 (42.9 to 69.8)
Motor vehicle road injuries	1 176 (945 to 1 426)	3 727 (3 028 to 4 502)	93.4 (75.1 to 113.1)	161.7 (131.9 to 194.0)
Other road injuries	671 (487 to 865)	1 195 (851 to 1 582)	51.8 (39.0 to 65.0)	53.7 (38.2 to 74.7)
Other transport injuries	333 (255 to 445)	424 (317 to 577)	24.8 (20.1 to 31.0)	19.7 (14.6 to 27.0)
**Unintentional injuries**	**39 133 (36 018 to 42 468)**	**59 690 (53 614 to 66 513)**	**3 099.1 (2 860.3 to 3 358.5)**	**2 794.2 (2 518.1 to 3 106.5)**
Falls	17 853 (15 563 to 20 542)	33 584 (28 513 to 39 664)	1 531.0 (1 354.5 to 1 733.1)	1 609.4 (1 362.6 to 1 907.8)
Drowning	54 (47 to 62)	45 (35 to 58)	4.0 (3.5 to 4.6)	2.0 (1.6 to 2.6)
Fire, heat, and hot substances	1 568 (1 231 to 1 968)	1 565 (1 264 to 1 934)	97.7 (78.7 to 120.4)	74.9 (60.4 to 93.4)
Poisonings	480 (347 to 669)	567 (411 to 760)	27.8 (20.7 to 37.9)	27.9 (20.0 to 37.6)
Poisoning by carbon monoxide	96 (57 to 158)	112 (58 to 185)	6.0 (3.8 to 9.3)	5.4 (2.8 to 8.9)
Poisoning by other means	384 (271 to 554)	454 (314 to 646)	21.8 (15.6 to 30.8)	22.6 (15.5 to 32.2)
Exposure to mechanical forces	7 958 (6 624 to 9 516)	9 258 (7 728 to 11 701)	578.9 (489.7 to 688.4)	412.7 (325.7 to 520.9)
Unintentional firearm injuries	466 (323 to 646)	429 (274 to 634)	33.0 (23.6 to 44.5)	18.9 (12.3 to 28.0)
Other exposure to mechanical forces	7 492 (6 255 to 9 019)	8 830 (6 936 to 11 162)	545.8 (462.2 to 651.1)	393.8 (310.3 to 499.5)
Adverse effects of medical treatment	1 134 (954 to 1 309)	2 205 (1 889 to 2 548)	96.5 (82.7 to 110.3)	100.7 (86.2 to 116.1)
Animal contact	3 451 (2 840 to 4 231)	4 015 (3 496 to 4 641)	258.2 (213.5 to 301.7)	186.2 (162.3 to 215.7)
Venomous animal contact	1 216 (914 to 1 607)	1 420 (1 132 to 1 757)	89.3 (68.7 to 113.7)	65.6 (52.2 to 81.6)
Non venomous animal contact	2 235 (1 783 to 2 800)	2 596 (2 190 to 3 135)	168.9 (133.4 to 200.3)	120.6 (101.5 to 145.5)
Foreign body	3 150 (1 966 to 4 391)	5 264 (2 931 to 7 957)	245.6 (148.5 to 355.5)	239.9 (138.0 to 357.5)
Pulmonary aspiration and foreign body in airway	100 (83 to 175)	88 (63 to 119)	7.9 (5.9 to 10.5)	4.2 (3.0 to 5.7)
Foreign body in eyes	2 671 (1 527 to 3 911)	4 827 (2 550 to 7 535)	214.6 (118.2 to 323.0)	219.0 (116.4 to 337.0)
Foreign body in other body part	349 (240 to 515)	350 (262 to 445)	23.0 (16.4 to 32.1)	16.7 (12.5 to 21.3)
Environmental heat and cold exposure	690 (486 to 900)	408 (303 to 549)	52.9 (40.4 to 65.2)	19.7 (14.7 to 26.8)
Exposure to forces of nature	103 (85 to 129)	24 (20 to 29)	6.6 (5.5 to 8.1)	1.1 (0.9 to 1.3)
Other unintentional injuries	2 696 (2 192 to 3 276)	2 755 (2 125 to 3 702)	204.9 (169.8 to 243.9)	125.6 (97.3 to 168.2)

Cause	Prevalence: All-age counts (95% UI) 1990	Prevalence: All-age counts (95% UI) 2017	Prevalence: Age-standardised rates per 100,000 (95% UI) 1990	Prevalence: Age-standardised rates per 100,000 (95% UI) 2017
**Transport and unintentional injuries**	**88 610 (83 089 to 94 834)**	**155 061 (145 480 to 167 359)**	**8 040.1 (7 589.8 to 8 637.0)**	**7 070.1 (6 631.8 to 7 620.7)**
**Transport injuries**	**11 027 (10 302 to 11 790)**	**27 929 (25 932 to 29 844)**	**1 031.6 (965.5 to 1 100.4)**	**1 251.3 (1 161.9 to 1 337.4)**
Road injuries	9 453 (8 839 to 10 112)	25 619 (23 772 to 27 495)	893.9 (835.3 to 958.6)	1 146.7 (1 065.4 to 1 229.8)
Pedestrian road injuries	2 308 (1 998 to 2 692)	4 470 (3 883 to 5 218)	223.2 (195.0 to 259.5)	202.5 (176.2 to 235.7)
Cyclist road injuries	1 608 (1 358 to 1 882)	4 087 (3 471 to 4 736)	146.7 (124.9 to 169.6)	183.4 (156.3 to 212.1)
Motorcyclist road injuries	1 995 (1 731 to 2 308)	5 769 (4 923 to 6 753)	186.1 (161.3 to 215.1)	254.8 (217.9 to 297.7)
Motor vehicle road injuries	2 221 (1 937 to 2 589)	8 392 (7 267 to 9 854)	215.2 (186.8 to 250.7)	375.1 (324.3 to 440.2)
Other road injuries	1 321 (1 099 to 1 583)	2 902 (2 426 to 3 506)	122.6 (102.2 to 146.6)	130.8 (109.3 to 157.8)
Other transport injuries	1 574 (1 303 to 1 857)	2 310 (1 960 to 2 668)	137.7 (115.9 to 159.4)	104.6 (89.1 to 120.5)
**Unintentional injuries**	**77 583 (72 304 to 83 767)**	**127 132 (117 492 to 139 018)**	**7 028.4 (6 561.6 to 7 575.0)**	**5 818.8 (5 376.5 to 6 350.0)**
Falls	32 041 (28 369 to 36 859)	67 534 (60 169 to 77 340)	3 054.8 (2 738.3 to 3 513.9)	3 146.2 (2 808.7 to 3 597.1)
Drowning	265 (234 to 304)	286 (248 to 335)	25.1 (22.3 to 28.6)	13.0 (11.3 to 15.2)
Fire, heat, and hot substances	15 828 (13 114 to 20 009)	19 594 (16 372 to 24 711)	1 300.4 (1 089.3 to 1 604.8)	876.0 (733.0 to 1 104.4)
Poisonings	485 (328 to 541)	712 (532 to 889)	32.5 (24.6 to 40.2)	31.7 (23.7 to 39.6)
Poisoning by carbon monoxide	69 (53 to 90)	95 (73 to 122)	5.3 (4.1 to 6.8)	4.2 (3.3 to 5.4)
Poisoning by other means	366 (261 to 468)	617 (442 to 789)	27.2 (19.5 to 34.8)	27.5 (19.7 to 35.3)
Exposure to mechanical forces	16 589 (14 532 to 19 161)	23 772 (20 645 to 27 544)	1 520.1 (1 333.1 to 1 743.4)	1 062.1 (922.4 to 1 227.7)
Unintentional firearm injuries	1 921 (1 535 to 2 270)	2 200 (1 783 to 2 629)	177.6 (143.3 to 210.5)	98.8 (80.4 to 117.7)
Other exposure to mechanical forces	14 668 (12 452 to 17 249)	21 571 (18 332 to 25 429)	1 342.5 (1 152.0 to 1 574.3)	963.3 (820.6 to 1 131.5)
Adverse effects of medical treatment	87 (68 to 107)	169 (130 to 210)	7.4 (5.7 to 9.1)	7.7 (5.9 to 9.6)
Animal contact	3 775 (3 247 to 4 362)	5 196 (4 530 to 5 927)	333.8 (291.7 to 378.7)	235.3 (205.2 to 267.4)
Venomous animal contact	811 (670 to 953)	1 076 (899 to 1 251)	68.2 (57.2 to 79.3)	48.4 (40.6 to 56.3)
Non venomous animal contact	2 964 (2 455 to 3 536)	4 120 (3 493 to 4 841)	265.5 (222.1 to 312.9)	186.7 (158.7 to 218.2)
Foreign body	1 774 (1 427 to 2 247)	2 489 (1 962 to 3 208)	148.9 (121.8 to 184.4)	113.6 (89.4 to 145.7)
Pulmonary aspiration and foreign body in airway	94 (67 to 127)	81 (67 to 100)	7.2 (5.3 to 9.5)	3.7 (3.0 to 4.5)
Foreign body in eyes	586 (291 to 877)	1 055 (607 to 1 666)	48.3 (28.4 to 75.1)	49.0 (28.4 to 76.2)
Foreign body in other body part	1 144 (916 to 1 458)	1 353 (1 116 to 1 685)	93.4 (76.1 to 117.0)	61.0 (50.4 to 75.8)
Environmental heat and cold exposure	2 475 (2 033 to 2 994)	1 672 (1 387 to 2 044)	217.1 (182.3 to 257.1)	75.7 (63.9 to 93.3)
Exposure to forces of nature	270 (195 to 364)	830 (580 to 1 153)	19.8 (14.5 to 26.7)	35.9 (25.2 to 50.0)
Other unintentional injuries	4 045 (3 469 to 4 645)	4 879 (4 212 to 5 602)	358.6 (318.3 to 420.5)	220.6 (190.6 to 252.2)

Cause	Years lived with disability: All-age counts (95% UI) 1990	Years lived with disability: All-age counts (95% UI) 2017	Years lived with disability: Age-standardised rates per 100,000 (95% UI) 1990	Years lived with disability: Age-standardised rates per 100,000 (95% UI) 2017
**Transport and unintentional injuries**	**3 937 (2 883 to 5 247)**	**6 689 (4 804 to 8 953)**	**354.9 (259.6 to 473.9)**	**306.1 (219.2 to 407.4)**
**Transport injuries**	**748 (546 to 992)**	**1 694 (1 223 to 2 263)**	**68.1 (49.8 to 90.3)**	**75.3 (54.4 to 100.5)**
Road injuries	647 (475 to 861)	1 570 (1 135 to 2 093)	59.6 (43.6 to 79.3)	69.7 (50.4 to 93.0)
Pedestrian road injuries	164 (118 to 223)	286 (201 to 392)	15.4 (11.0 to 20.8)	12.8 (9.0 to 17.6)
Cyclist road injuries	111 (79 to 147)	247 (173 to 332)	9.9 (7.1 to 13.0)	11.0 (7.7 to 14.8)
Motorcyclist road injuries	139 (97 to 189)	356 (243 to 486)	12.6 (8.8 to 17.1)	15.6 (10.7 to 21.3)
Motor vehicle road injuries	158 (113 to 209)	533 (382 to 714)	14.8 (10.7 to 19.6)	23.6 (16.9 to 31.7)
Other road injuries	76 (54 to 103)	148 (104 to 203)	6.8 (5.0 to 9.3)	6.6 (4.7 to 9.1)
Other transport injuries	101 (72 to 135)	124 (87 to 167)	8.5 (6.2 to 11.2)	5.6 (3.9 to 7.5)
**Unintentional injuries**	**3 189 (2 333 to 4 298)**	**4 996 (3 546 to 6 756)**	**286.8 (208.1 to 384.3)**	**229.8 (163.3 to 311.2)**
Falls	1 901 (1 383 to 2 486)	3 469 (2 488 to 4 593)	177.3 (129.1 to 232.0)	161.3 (115.4 to 212.7)
Drowning	15 (10 to 20)	15 (10 to 21)	1.4 (1.0 to 1.9)	0.7 (0.5 to 1.0)
Fire, heat, and hot substances	340 (230 to 535)	381 (224 to 624)	27.8 (16.9 to 42.7)	17.0 (10.0 to 27.8)
Poisonings	51 (31 to 77)	82 (50 to 124)	3.7 (2.2 to 5.6)	3.6 (2.2 to 5.5)
Poisoning by carbon monoxide	7 (4 to 12)	10 (6 to 15)	0.5 (0.3 to 0.9)	0.4 (0.3 to 0.7)
Poisoning by other means	44 (26 to 67)	72 (43 to 111)	3.2 (1.9 to 4.9)	3.2 (1.9 to 5.0)
Exposure to mechanical forces	391 (267 to 556)	513 (348 to 744)	35.3 (24.2 to 50.2)	22.9 (15.5 to 33.2)
Unintentional firearm injuries	39 (25 to 59)	41 (26 to 64)	3.5 (2.3 to 5.3)	1.8 (1.2 to 2.9)
Other exposure to mechanical forces	352 (243 to 503)	472 (319 to 687)	31.8 (21.9 to 45.6)	21.1 (14.2 to 30.6)
Adverse effects of medical treatment	12 (7 to 17)	23 (14 to 34)	1.0 (0.6 to 1.5)	1.0 (0.6 to 1.5)
Animal contact	97 (65 to 137)	119 (78 to 171)	8.0 (5.3 to 11.1)	5.3 (3.5 to 7.7)
Venomous animal contact	51 (33 to 75)	60 (38 to 88)	3.9 (2.5 to 5.6)	2.7 (1.7 to 3.9)
Non venomous animal contact	46 (28 to 73)	58 (35 to 95)	4.0 (2.5 to 6.4)	2.6 (1.6 to 4.3)
Foreign body	111 (76 to 156)	139 (91 to 205)	9.1 (6.2 to 12.7)	6.3 (4.2 to 9.3)
Pulmonary aspiration and foreign body in airway	5 (3 to 7)	3 (2 to 5)	0.4 (0.3 to 0.5)	0.2 (0.1 to 0.2)
Foreign body in eyes	36 (19 to 61)	71 (39 to 121)	3.3 (1.8 to 5.4)	3.3 (1.8 to 5.6)
Foreign body in other body part	70 (49 to 93)	64 (46 to 85)	5.5 (3.9 to 7.2)	2.9 (2.0 to 3.8)
Environmental heat and cold exposure	98 (64 to 138)	63 (41 to 91)	8.5 (5.7 to 12.0)	2.9 (1.9 to 4.2)
Exposure to forces of nature	14 (10 to 19)	27 (20 to 38)	1.0 (0.7 to 1.4)	1.2 (0.9 to 1.6)
Other unintentional injuries	159 (114 to 214)	166 (118 to 227)	14.2 (10.2 to 19.3)	7.5 (5.3 to 10.3)

Cause	Disability adjusted life years: All-age counts (95% UI) 1990	Disability adjusted life years: All-age counts (95% UI) 2017	Disability adjusted life years: Age-standardised rates per 100,000 (95% UI) 1990	Disability adjusted life years: Age-standardised rates per 100,000 (95% UI) 2017
**Transport and unintentional injuries**	**35 947 (33 851 to 38 267)**	**36 803 (33 574 to 40 177)**	**2 663.4 (2 518.5 to 2 818.3)**	**1 646.9 (1 502.6 to 1 795.6)**
**Transport injuries**	**17 043 (16 056 to 18 077)**	**19 527 (17 693 to 21 491)**	**1 248.1 (1 183.0 to 1 316.3)**	**848.8 (770.6 to 932.3)**
Road injuries	16 158 (15 246 to 17 151)	18 161 (16 407 to 19 959)	1 182.9 (1 122.0 to 1 248.9)	789.3 (714.7 to 866.7)
Pedestrian road injuries	7 109 (6 568 to 7 697)	6 697 (6 028 to 7 467)	530.8 (495.3 to 568.7)	295.5 (266.7 to 328.4)
Cyclist road injuries	964 (859 to 1 066)	1 699 (1 489 to 1 936)	72.5 (65.2 to 79.4)	74.4 (65.4 to 84.6)
Motorcyclist road injuries	2 805 (2 545 to 3 111)	3 801 (3 332 to 4 329)	200.0 (182.8 to 220.1)	161.6 (141.8 to 183.8)
Motor vehicle road injuries	4 892 (4 505 to 5 353)	5 505 (4 870 to 6 145)	350.7 (324.5 to 381.5)	237.7 (210.5 to 264.4)
Other road injuries	388 (330 to 444)	458 (392 to 541)	28.9 (24.9 to 33.0)	20.1 (17.2 to 23.8)
Other transport injuries	885 (795 to 982)	1 366 (1 179 to 1 558)	65.1 (58.8 to 71.6)	59.5 (51.5 to 67.5)
**Unintentional injuries**	**18 904 (17 491 to 20 570)**	**17 276 (15 449 to 19 267)**	**1 415.4 (1 314.7 to 1 531.0)**	**797.0 (713.6 to 888.3)**
Falls	4 411 (3 900 to 5 031)	6 250 (5 206 to 7 405)	405.6 (357.1 to 461.0)	290.8 (242.3 to 344.7)
Drowning	3 311 (3 008 to 3 643)	2 016 (1 808 to 2 256)	221.2 (203.7 to 241.4)	90.2 (81.0 to 100.9)
Fire, heat, and hot substances	1 501 (1 312 to 1 743)	1 048 (878 to 1 285)	110.1 (97.1 to 128.1)	48.1 (40.5 to 58.7)
Poisonings	712 (641 to 796)	501 (444 to 565)	47.3 (43.3 to 52.0)	22.6 (20.0 to 25.4)
Poisoning by carbon monoxide	159 (141 to 179)	129 (112 to 147)	10.8 (9.7 to 12.1)	5.7 (5.0 to 6.5)
Poisoning by other means	554 (494 to 622)	372 (325 to 426)	36.4 (33.1 to 40.3)	16.8 (14.7 to 19.3)
Exposure to mechanical forces	2 070 (1 884 to 2 287)	1 635 (1 421 to 1 898)	158.4 (144.3 to 175.1)	71.9 (62.5 to 83.9)
Unintentional firearm injuries	669 (583 to 745)	372 (256 to 435)	48.6 (42.2 to 54.0)	16.0 (11.1 to 18.6)
Other exposure to mechanical forces	1 401 (1 245 to 1 584)	1 263 (1 073 to 1 505)	109.8 (97.5 to 125.1)	55.9 (47.6 to 66.7)
Adverse effects of medical treatment	1 129 (918 to 1 301)	1 118 (987 to 1 311)	84.9 (69.4 to 94.8)	53.5 (47.2 to 62.9)
Animal contact	581 (494 to 673)	322 (273 to 383)	38.7 (33.6 to 44.3)	14.9 (12.7 to 17.7)
Venomous animal contact	448 (372 to 531)	202 (169 to 236)	28.6 (24.4 to 33.1)	9.5 (7.9 to 11.1)
Non venomous animal contact	133 (107 to 166)	120 (94 to 159)	10.1 (8.3 to 12.9)	5.5 (4.3 to 7.2)
Foreign body	3 296 (2 860 to 3 828)	2 907 (2 557 to 3 352)	208.3 (184.7 to 236.0)	140.3 (122.2 to 164.4)
Pulmonary aspiration and foreign body in airway	3 083 (2 652 to 3 615)	2 716 (2 376 to 3 162)	192.6 (169.0 to 220.4)	131.6 (113.9 to 155.5)
Foreign body in eyes	36 (19 to 61)	71 (39 to 121)	3.3 (1.8 to 5.4)	3.3 (1.8 to 5.6)
Foreign body in other body part	177 (150 to 214)	121 (95 to 186)	12.5 (10.6 to 15.0)	5.4 (4.2 to 8.4)
Environmental heat and cold exposure	485 (436 to 542)	285 (249 to 325)	39.5 (35.8 to 43.9)	12.8 (11.2 to 14.7)
Exposure to forces of nature	54 (47 to 61)	45 (38 to 56)	3.6 (3.2 to 4.1)	2.0 (1.7 to 2.5)
Other unintentional injuries	1 355 (1 229 to 1 481)	1 148 (1 003 to 1 297)	97.8 (88.9 to 106.4)	49.9 (43.7 to 56.2)

Death, YLL, Incidence, prevalence, YLD, and DALY counts and rates per 100,000 for all injuries in Zacatecas in 1990 and 2017

Cause	Deaths: All-age counts (95% UI) 1990	Deaths: All-age counts (95% UI) 2017	Deaths: Age-standardised rates per 100,000 (95% UI) 1990	Deaths: Age-standardised rates per 100,000 (95% UI) 2017	Years of life lost: All-age counts (95% UI) 1990	Years of life lost: All-age counts (95% UI) 2017	Years of life lost: Age-standardised rates per 100,000 (95% UI) 1990	Years of life lost: Age-standardised rates per 100,000 (95% UI) 2017
**Transport and unintentional injuries**	**828 (778 to 881)**	**733 (684 to 783)**	**72.5 (69.4 to 75.6)**	**44.9 (41.9 to 47.9)**	**45 924 (41 815 to 50 243)**	**31 533 (29 222 to 33 943)**	**3 337.2 (3 108.8 to 3 578.7)**	**1 915.6 (1 776.5 to 2 061.5)**
**Transport injuries**	**388 (364 to 412)**	**396 (364 to 428)**	**33.6 (31.9 to 35.2)**	**24.2 (22.2 to 26.2)**	**21 991 (20 292 to 23 792)**	**19 270 (17 602 to 20 888)**	**1 654.1 (1 547.9 to 1 760.9)**	**1 165.2 (1 064.6 to 1 263.7)**
Road injuries	378 (355 to 401)	383 (351 to 414)	32.7 (31.1 to 34.2)	23.4 (21.5 to 25.3)	21 422 (19 762 to 23 208)	18 628 (17 033 to 20 200)	1 610.7 (1 507.0 to 1 716.0)	1 126.4 (1 029.0 to 1 220.2)
Pedestrian road injuries	160 (147 to 174)	120 (110 to 132)	14.2 (13.4 to 15.1)	7.4 (6.8 to 8.2)	8 684 (7 728 to 9 802)	4 955 (4 475 to 5 500)	634.9 (579.4 to 699.8)	302.5 (273.5 to 335.1)
Cyclist road injuries	9 (8 to 10)	14 (12 to 16)	0.8 (0.7 to 0.8)	0.9 (0.8 to 1.0)	489 (436 to 554)	627 (547 to 715)	38.0 (34.1 to 42.4)	38.1 (33.4 to 43.4)
Motorcyclist road injuries	56 (51 to 63)	44 (38 to 51)	4.6 (4.2 to 5.0)	2.7 (2.3 to 3.0)	3 403 (3 040 to 3 815)	2 459 (2 117 to 2 852)	256.5 (232.2 to 283.6)	147.2 (126.9 to 170.6)
Motor vehicle road injuries	141 (130 to 151)	194 (176 to 213)	12.1 (11.2 to 12.8)	11.8 (10.7 to 12.9)	8 169 (7 427 to 8 852)	10 098 (9 108 to 11 074)	631.0 (580.7 to 675.2)	608.8 (549.3 to 667.4)
Other road injuries	12 (10 to 14)	11 (9 to 12)	1.0 (0.9 to 1.2)	0.6 (0.6 to 0.7)	676 (571 to 800)	490 (420 to 572)	50.3 (43.4 to 58.1)	29.7 (25.5 to 34.6)
Other transport injuries	10 (9 to 11)	14 (12 to 16)	0.9 (0.8 to 1.0)	0.8 (0.7 to 0.9)	571 (503 to 644)	641 (538 to 733)	43.4 (38.7 to 48.6)	38.9 (32.6 to 44.4)
**Unintentional injuries**	**440 (409 to 476)**	**336 (316 to 359)**	**38.9 (37.1 to 40.9)**	**20.7 (19.4 to 22.0)**	**23 932 (21 347 to 26 851)**	**12 263 (11 346 to 13 365)**	**1 683.1 (1 545.4 to 1 844.9)**	**750.3 (694.2 to 818.0)**
Falls	99 (94 to 104)	121 (110 to 131)	11.5 (10.9 to 12.1)	7.4 (6.7 to 8.1)	2 997 (2 780 to 3 244)	2 531 (2 237 to 2 756)	274.0 (259.6 to 290.7)	156.9 (139.0 to 170.9)
Drowning	82 (74 to 93)	42 (38 to 46)	6.1 (5.6 to 6.7)	2.6 (2.3 to 2.8)	5 357 (4 670 to 6 213)	2 188 (1 974 to 2 428)	350.4 (312.1 to 395.8)	132.4 (119.7 to 147.0)
Fire, heat, and hot substances	31 (28 to 34)	20 (18 to 21)	2.8 (2.6 to 3.0)	1.2 (1.1 to 1.3)	1 633 (1 379 to 1 944)	605 (538 to 663)	112.7 (99.1 to 129.5)	37.1 (33.0 to 40.7)
Poisonings	24 (21 to 27)	13 (12 to 15)	1.8 (1.7 to 2.0)	0.8 (0.7 to 0.9)	1 564 (1 350 to 1 813)	645 (568 to 719)	109.0 (91.1 to 116.4)	39.2 (34.6 to 43.7)
Poisoning by carbon monoxide	12 (11 to 13)	8 (7 to 8)	0.9 (0.8 to 1.0)	0.5 (0.4 to 0.5)	752 (647 to 866)	359 (312 to 402)	50.7 (44.9 to 57.1)	21.8 (18.9 to 24.4)
Poisoning by other means	12 (11 to 14)	6 (5 to 6)	0.9 (0.8 to 1.0)	0.4 (0.3 to 0.4)	813 (687 to 970)	286 (251 to 323)	52.3 (45.4 to 60.8)	17.4 (15.3 to 19.6)
Exposure to mechanical forces	53 (49 to 56)	30 (28 to 34)	4.7 (4.4 to 4.9)	1.9 (1.7 to 2.1)	2 913 (2 677 to 3 189)	1 330 (1 201 to 1 481)	224.3 (208.4 to 241.6)	80.8 (73.0 to 89.9)
Unintentional firearm injuries	23 (21 to 27)	10 (9 to 12)	2.0 (1.8 to 2.3)	0.6 (0.5 to 0.7)	1 340 (1 202 to 1 547)	490 (408 to 568)	102.3 (92.6 to 117.5)	29.6 (24.6 to 34.3)
Other exposure to mechanical forces	29 (26 to 32)	20 (18 to 23)	2.7 (2.3 to 2.9)	1.2 (1.1 to 1.4)	1 573 (1 367 to 1 768)	839 (745 to 954)	122.0 (105.6 to 134.6)	51.2 (45.5 to 58.2)
Adverse effects of medical treatment	23 (19 to 26)	25 (23 to 30)	2.3 (1.9 to 2.5)	1.6 (1.4 to 1.9)	1 048 (821 to 1 228)	718 (634 to 912)	76.9 (62.9 to 87.3)	44.5 (39.4 to 56.5)
Animal contact	22 (19 to 26)	9 (8 to 10)	1.6 (1.4 to 1.8)	0.6 (0.5 to 0.6)	1 518 (1 227 to 1 870)	379 (338 to 426)	92.5 (77.3 to 110.9)	23.2 (20.8 to 26.1)
Venomous animal contact	17 (14 to 21)	6 (5 to 7)	1.2 (1.0 to 1.4)	0.4 (0.3 to 0.4)	1 247 (973 to 1 575)	247 (213 to 289)	74.0 (59.1 to 91.4)	15.2 (13.1 to 17.8)
Non-venomous animal contact	5 (3 to 6)	3 (3 to 4)	0.4 (0.3 to 0.5)	0.2 (0.2 to 0.2)	271 (180 to 344)	132 (112 to 155)	18.5 (12.5 to 22.4)	8.1 (6.9 to 9.5)
Foreign body	71 (61 to 83)	54 (49 to 61)	5.1 (4.5 to 5.7)	3.3 (3.0 to 3.7)	4 955 (4 134 to 5 982)	2 862 (2 479 to 3 357)	302.3 (258.0 to 356.3)	175.1 (151.6 to 205.6)
Pulmonary aspiration and foreign body in airway	68 (59 to 80)	53 (48 to 59)	4.9 (4.4 to 5.5)	3.3 (3.0 to 3.7)	4 775 (3 973 to 5 791)	2 799 (2 422 to 3 275)	290.3 (246.9 to 343.8)	171.3 (148.1 to 200.6)
Foreign body in eyes								
Foreign body in other body part	3 (2 to 3)	1 (1 to 2)	0.2 (0.1 to 0.2)	0.1 (0.1 to 0.1)	181 (131 to 221)	63 (50 to 100)	12.0 (8.7 to 14.2)	3.8 (3.0 to 6.0)
Environmental heat and cold exposure	8 (8 to 9)	4 (4 to 5)	0.9 (0.8 to 0.9)	0.3 (0.2 to 0.3)	389 (344 to 442)	146 (126 to 164)	31.3 (28.3 to 34.5)	9.0 (7.8 to 10.1)
Exposure to forces of nature	1 (1 to 2)	0 (0 to 0)	0.2 (0.1 to 0.2)	0.0 (0.0 to 0.0)	80 (61 to 107)	13 (12 to 15)	7.7 (5.6 to 10.6)	0.8 (0.7 to 0.9)
Other unintentional injuries	25 (23 to 27)	17 (15 to 19)	2.1 (1.9 to 2.2)	1.0 (0.9 to 1.1)	1 477 (1 338 to 1 623)	847 (743 to 956)	108.1 (98.9 to 118.1)	51.1 (44.9 to 57.7)

Cause	Incidence: All-age counts (95% UI) 1990	Incidence: All-age counts (95% UI) 2017	Incidence: Age-standardised rates per 100,000 (95% UI) 1990	Incidence: Age-standardised rates per 100,000 (95% UI) 2017	Prevalence: All-age counts (95% UI) 1990	Prevalence: All-age counts (95% UI) 2017	Prevalence: Age-standardised rates per 100,000 (95% UI) 1990	Prevalence: Age-standardised rates per 100,000 (95% UI) 2017
**Transport and unintentional injuries**	**50 585 (46 411 to 55 118)**	**67 288 (61 023 to 73 885)**	**4 304.5 (3 941.1 to 4 711.8)**	**4 141.4 (3 752.6 to 4 547.0)**	**94 846 (89 140 to 101 405)**	**138 190 (129 912 to 148 914)**	**9 696.8 (9 145.4 to 10 383.9)**	**8 576.8 (8 062.1 to 9 251.2)**
**Transport injuries**	**4 531 (3 894 to 5 255)**	**8 606 (7 442 to 9 861)**	**379.1 (331.6 to 430.8)**	**524.1 (454.8 to 600.1)**	**11 112 (10 359 to 11 846)**	**24 956 (23 188 to 26 850)**	**1 182.6 (1 102.7 to 1 261.0)**	**1 552.1 (1 442.4 to 1 668.4)**
Road injuries	4 316 (3 699 to 5 049)	8 443 (7 282 to 9 698)	362.8 (315.5 to 413.6)	514.1 (444.4 to 590.4)	10 140 (9 442 to 10 852)	24 144 (22 391 to 26 016)	1 088.7 (1 015.6 to 1 165.1)	1 501.6 (1 393.0 to 1 616.7)
Pedestrian road injuries	659 (523 to 810)	913 (728 to 1 105)	56.3 (46.5 to 67.7)	55.9 (44.8 to 67.9)	2 473 (2 119 to 2 920)	4 392 (3 809 to 5 099)	262.6 (228.3 to 307.5)	273.7 (237.6 to 317.6)
Cyclist road injuries	629 (478 to 808)	1 094 (876 to 1 355)	48.1 (37.7 to 60.1)	66.5 (53.4 to 82.4)	1 065 (896 to 1 258)	2 515 (2 142 to 2 949)	108.5 (91.9 to 125.8)	155.7 (132.6 to 182.1)
Motorcyclist road injuries	549 (433 to 687)	732 (569 to 913)	44.9 (36.1 to 55.3)	44.2 (34.4 to 55.0)	2 091 (1 768 to 2 332)	3 494 (2 998 to 4 090)	218.1 (188.6 to 250.7)	216.7 (186.0 to 253.5)
Motor vehicle road injuries	1 541 (1 263 to 1 834)	3 885 (3 271 to 4 610)	136.3 (113.1 to 160.8)	236.5 (199.3 to 279.7)	2 856 (2 492 to 3 311)	9 286 (8 123 to 10 885)	318.9 (276.9 to 368.2)	578.8 (505.3 to 675.3)
Other road injuries	938 (728 to 1 167)	1 819 (1 474 to 2 186)	77.2 (62.4 to 92.5)	110.9 (90.2 to 133.2)	1 715 (1 433 to 2 057)	4 457 (3 716 to 5 383)	180.6 (152.3 to 217.0)	276.7 (231.1 to 334.2)
Other transport injuries	215 (165 to 286)	164 (134 to 199)	16.5 (13.3 to 20.4)	10.0 (8.2 to 12.2)	972 (808 to 1 156)	812 (704 to 926)	93.8 (79.2 to 109.0)	50.5 (43.9 to 57.5)
**Unintentional injuries**	**46 054 (41 912 to 50 430)**	**58 682 (52 664 to 65 269)**	**3 925.2 (3 561.9 to 4 339.6)**	**3 617.3 (3 243.4 to 4 021.2)**	**83 735 (78 190 to 90 268)**	**113 233 (105 067 to 123 820)**	**8 514.2 (7 972.4 to 9 172.7)**	**7 024.7 (6 516.8 to 7 679.7)**
Falls	19 861 (17 516 to 22 470)	34 156 (30 184 to 38 649)	1 804.2 (1 612.2 to 2 017.8)	2 111.2 (1 863.5 to 2 384.9)	34 342 (30 435 to 39 239)	65 352 (58 417 to 74 714)	3 635.5 (3 244.8 to 4 137.7)	4 059.4 (3 634.5 to 4 646.1)
Drowning	71 (61 to 82)	39 (32 to 50)	5.3 (4.6 to 6.0)	2.4 (1.9 to 3.0)	321 (282 to 370)	244 (215 to 285)	33.4 (29.4 to 38.2)	15.2 (13.4 to 17.8)
Fire, heat, and hot substances	1 674 (1 318 to 2 151)	1 100 (868 to 1 386)	106.8 (86.7 to 132.3)	66.9 (52.8 to 84.1)	15 537 (12 814 to 19 723)	12 917 (10 688 to 16 652)	1 420.2 (1 188.0 to 1 743.0)	794.6 (657.5 to 1 021.7)
Poisonings	650 (482 to 853)	468 (344 to 618)	37.7 (28.8 to 48.1)	28.4 (20.8 to 37.4)	546 (414 to 668)	522 (391 to 643)	44.2 (34.1 to 53.8)	31.8 (23.9 to 39.2)
Poisoning by carbon monoxide	129 (86 to 192)	81 (52 to 122)	8.3 (5.9 to 11.5)	5.0 (3.2 to 7.4)	86 (66 to 113)	59 (46 to 75)	7.2 (5.6 to 9.3)	3.6 (2.8 to 4.6)
Poisoning by other means	521 (377 to 711)	386 (276 to 524)	29.5 (21.7 to 39.3)	23.4 (16.8 to 31.7)	459 (331 to 576)	463 (334 to 585)	37.0 (27.1 to 46.3)	28.2 (20.4 to 35.6)
Exposure to mechanical forces	10 064 (8 445 to 11 866)	8 944 (7 630 to 10 467)	796.4 (678.2 to 932.0)	544.1 (465.4 to 637.9)	19 943 (17 499 to 22 927)	22 368 (19 610 to 25 865)	2 109.5 (1 860.9 to 2 401.3)	1 389.6 (1 217.6 to 1 606.0)
Unintentional firearm injuries	742 (540 to 996)	443 (326 to 584)	58.2 (43.2 to 76.1)	26.9 (19.9 to 35.5)	2 771 (2 228 to 3 306)	2 236 (1 819 to 2 702)	300.1 (241.9 to 360.2)	139.2 (113.3 to 168.1)
Other exposure to mechanical forces	9 322 (7 786 to 11 031)	8 501 (7 271 to 9 968)	738.2 (628.6 to 866.9)	517.2 (442.9 to 607.3)	17 172 (14 669 to 20 171)	20 131 (17 201 to 23 653)	1 809.4 (1 552.4 to 2 123.4)	1 250.4 (1 068.6 to 1 467.5)
Adverse effects of medical treatment	902 (760 to 1 054)	1 488 (1 268 to 1 749)	85.2 (73.2 to 99.4)	92.6 (79.0 to 108.2)	69 (54 to 86)	114 (88 to 143)	6.5 (5.0 to 8.1)	7.1 (5.5 to 8.9)
Animal contact	4 700 (3 966 to 5 605)	3 363 (2 935 to 3 852)	363.5 (315.3 to 419.8)	205.8 (181.2 to 236.9)	4 878 (4 185 to 5 663)	4 116 (3 616 to 4 698)	478.3 (417.2 to 545.4)	255.3 (224.7 to 291.1)
Venomous animal contact	1 669 (1 297 to 2 133)	1 106 (880 to 1 368)	128.6 (101.4 to 159.4)	68.0 (54.0 to 83.8)	1 103 (904 to 1 334)	769 (639 to 885)	101.1 (84.4 to 120.1)	47.1 (39.5 to 54.5)
Non-venomous animal contact	3 031 (2 482 to 3 689)	2 257 (1 916 to 2 666)	234.9 (198.0 to 279.3)	138.8 (118.0 to 163.7)	3 776 (3 119 to 4 573)	3 352 (2 854 to 3 920)	377.2 (319.0 to 443.5)	208.2 (178.1 to 243.0)
Foreign body	4 784 (2 540 to 7 634)	6 686 (2 714 to 11 628)	455.8 (211.6 to 768.8)	415.8 (166.0 to 723.9)	2 109 (1 621 to 2 804)	2 290 (1 573 to 3 208)	206.6 (159.6 to 276.7)	138.8 (98.0 to 200.5)
Pulmonary aspiration and foreign body in airway	158 (129 to 227)	69 (51 to 93)	9.6 (7.0 to 13.3)	4.2 (3.1 to 5.7)	107 (75 to 147)	59 (48 to 72)	8.8 (6.4 to 11.9)	3.6 (3.0 to 4.4)
Foreign body in eyes	4 266 (2 016 to 7 100)	6 417 (2 417 to 11 357)	421.6 (173.7 to 735.6)	399.3 (148.8 to 704.5)	895 (476 to 1 536)	1 457 (811 to 2 429)	97.0 (55.0 to 159.2)	93.1 (50.6 to 151.1)
Foreign body in other body part	360 (233 to 561)	200 (139 to 259)	24.6 (16.8 to 35.5)	12.3 (8.6 to 15.9)	1 106 (886 to 1 410)	714 (596 to 866)	100.8 (82.2 to 125.3)	44.2 (36.9 to 53.5)
Environmental heat and cold exposure	613 (443 to 821)	290 (219 to 383)	48.6 (37.7 to 60.4)	17.9 (13.6 to 23.6)	2 072 (1 702 to 2 510)	1 087 (923 to 1 295)	201.4 (169.7 to 239.6)	67.4 (57.3 to 80.3)
Exposure to forces of nature	197 (150 to 259)	18 (15 to 21)	18.5 (13.6 to 25.0)	1.1 (0.9 to 1.3)	305 (226 to 403)	606 (425 to 833)	26.4 (19.9 to 34.9)	37.3 (26.2 to 51.3)
Other unintentional injuries	2 540 (2 112 to 3 080)	2 131 (1 801 to 2 546)	208.2 (172.5 to 240.1)	130.0 (109.4 to 155.2)	3 613 (3 111 to 4 150)	3 681 (3 216 to 4 184)	372.2 (322.3 to 425.5)	228.3 (199.6 to 259.4)

Cause	Years lived with disability: All-age counts (95% UI) 1990	Years lived with disability: All-age counts (95% UI) 2017	Years lived with disability: Age-standardised rates per 100,000 (95% UI) 1990	Years lived with disability: Age-standardised rates per 100,000 (95% UI) 2017	Disability adjusted life years: All-age counts (95% UI) 1990	Disability adjusted life years: All-age counts (95% UI) 2017	Disability adjusted life years: Age-standardised rates per 100,000 (95% UI) 1990	Disability adjusted life years: Age-standardised rates per 100,000 (95% UI) 2017
**Transport and unintentional injuries**	**4 206 (3 077 to 5 565)**	**6 189 (4 470 to 8 280)**	**425.2 (309.8 to 561.3)**	**383.8 (277.6 to 513.4)**	**50 131 (45 882 to 54 793)**	**37 722 (34 748 to 41 068)**	**3 762.4 (3 520.7 to 4 036.1)**	**2 299.4 (2 118.3 to 2 503.4)**
**Transport injuries**	**760 (557 to 999)**	**1 536 (1 122 to 2 062)**	**79.0 (57.8 to 104.2)**	**95.3 (69.7 to 128.0)**	**22 752 (21 022 to 24 603)**	**20 805 (19 101 to 22 488)**	**1 733.1 (1 626.7 to 1 842.0)**	**1 260.6 (1 158.3 to 1 360.3)**
Road injuries	698 (511 to 918)	1 492 (1 092 to 2 005)	73.2 (53.5 to 95.9)	92.6 (67.8 to 124.5)	22 119 (20 422 to 23 918)	20 120 (18 445 to 21 725)	1 683.9 (1 581.5 to 1 790.5)	1 219.0 (1 118.2 to 1 315.4)
Pedestrian road injuries	178 (126 to 241)	284 (199 to 386)	18.4 (13.2 to 24.7)	17.6 (12.4 to 24.0)	8 862 (7 931 to 9 995)	5 238 (4 772 to 5 779)	653.3 (598.5 to 721.8)	320.2 (291.9 to 352.8)
Cyclist road injuries	75 (52 to 98)	155 (108 to 207)	7.5 (5.3 to 9.7)	9.6 (6.7 to 12.8)	564 (506 to 630)	782 (692 to 888)	45.4 (41.2 to 50.4)	47.7 (42.3 to 54.2)
Motorcyclist road injuries	142 (100 to 191)	220 (150 to 296)	14.9 (10.5 to 20.1)	13.6 (9.3 to 18.3)	3 545 (3 174 to 3 951)	2 679 (2 332 to 3 060)	271.5 (246.2 to 298.6)	160.8 (140.3 to 183.8)
Motor vehicle road injuries	204 (148 to 271)	597 (432 to 795)	22.2 (16.1 to 29.3)	37.1 (26.9 to 49.4)	8 373 (7 628 to 9 057)	10 695 (9 685 to 11 666)	653.2 (604.0 to 698.4)	645.9 (585.5 to 704.6)
Other road injuries	99 (71 to 134)	236 (169 to 326)	10.2 (7.4 to 13.8)	14.7 (10.5 to 20.2)	776 (663 to 901)	727 (623 to 852)	60.5 (52.7 to 69.3)	44.3 (38.0 to 52.7)
Other transport injuries	62 (44 to 83)	44 (31 to 59)	5.8 (4.1 to 7.6)	2.7 (1.9 to 3.6)	633 (565 to 714)	685 (578 to 781)	49.2 (44.4 to 54.6)	41.6 (35.1 to 47.3)
**Unintentional injuries**	**3 447 (2 497 to 4 580)**	**4 653 (3 322 to 6 246)**	**346.2 (249.5 to 461.2)**	**288.5 (206.1 to 387.7)**	**27 378 (24 639 to 30 585)**	**16 916 (15 165 to 18 836)**	**2 029.3 (1 868.9 to 2 221.9)**	**1 038.8 (931.0 to 1 156.3)**
Falls	2 044 (1 488 to 2 663)	3 407 (2 448 to 4 522)	211.1 (153.1 to 274.4)	211.5 (152.0 to 281.0)	5 041 (4 451 to 5 704)	5 938 (4 913 to 7 078)	485.1 (426.4 to 551.6)	368.4 (305.0 to 439.4)
Drowning	18 (13 to 25)	13 (9 to 18)	1.9 (1.3 to 2.5)	0.8 (0.6 to 1.1)	5 375 (4 687 to 6 232)	2 201 (1 985 to 2 443)	352.2 (314.2 to 397.9)	133.2 (120.4 to 147.9)
Fire, heat, and hot substances	336 (211 to 523)	253 (150 to 406)	29.9 (18.7 to 46.7)	15.6 (9.2 to 24.9)	1 969 (1 689 to 2 360)	858 (733 to 1 016)	142.6 (125.0 to 166.2)	52.7 (45.0 to 62.3)
Poisonings	65 (41 to 98)	60 (37 to 91)	5.1 (3.2 to 7.7)	3.7 (2.2 to 5.5)	1 629 (1 415 to 1 886)	705 (626 to 787)	108.1 (96.3 to 122.0)	42.9 (38.1 to 47.8)
Poisoning by carbon monoxide	9 (6 to 15)	5 (3 to 9)	0.7 (0.5 to 1.2)	0.3 (0.2 to 0.5)	761 (656 to 875)	365 (317 to 407)	51.5 (45.7 to 57.9)	22.1 (19.2 to 24.7)
Poisoning by other means	56 (34 to 85)	55 (33 to 84)	4.4 (2.7 to 6.7)	3.3 (2.0 to 5.1)	868 (740 to 1 029)	341 (297 to 388)	56.6 (49.3 to 65.4)	20.8 (18.1 to 23.7)
Exposure to mechanical forces	473 (326 to 676)	488 (332 to 710)	49.2 (33.8 to 70.2)	30.3 (20.5 to 44.0)	3 385 (3 110 to 3 712)	1 817 (1 595 to 2 071)	273.5 (251.5 to 299.6)	111.1 (97.4 to 126.8)
Unintentional firearm injuries	58 (38 to 87)	42 (27 to 66)	6.1 (4.0 to 9.4)	2.6 (1.7 to 4.1)	1 397 (1 254 to 1 599)	532 (442 to 612)	108.4 (98.0 to 124.1)	32.2 (26.8 to 37.1)
Other exposure to mechanical forces	415 (286 to 590)	446 (302 to 650)	43.2 (29.8 to 61.9)	27.7 (18.7 to 40.3)	1 988 (1 757 to 2 235)	1 285 (1 103 to 1 519)	165.1 (146.2 to 186.2)	78.9 (67.7 to 93.4)
Adverse effects of medical treatment	9 (6 to 14)	15 (9 to 23)	0.9 (0.5 to 1.3)	0.9 (0.6 to 1.4)	1 058 (827 to 1 238)	734 (652 to 925)	77.8 (63.7 to 88.1)	45.5 (40.4 to 57.3)
Animal contact	130 (88 to 185)	94 (62 to 133)	11.6 (7.9 to 16.7)	5.8 (3.8 to 8.2)	1 648 (1 347 to 2 007)	472 (423 to 530)	104.1 (87.7 to 122.7)	29.0 (25.8 to 32.5)
Venomous animal contact	72 (46 to 106)	45 (29 to 65)	5.9 (3.8 to 8.6)	2.8 (1.8 to 3.9)	1 319 (1 044 to 1 651)	292 (256 to 334)	79.8 (65.1 to 97.2)	17.9 (15.7 to 20.6)
Non-venomous animal contact	59 (36 to 94)	48 (29 to 77)	5.8 (3.5 to 9.2)	3.0 (1.8 to 4.8)	329 (234 to 406)	180 (150 to 215)	24.3 (17.7 to 29.7)	11.1 (9.2 to 13.2)
Foreign body	132 (88 to 196)	134 (79 to 219)	12.8 (8.4 to 19.2)	8.4 (5.0 to 13.7)	5 088 (4 273 to 6 106)	2 996 (2 608 to 3 484)	315.2 (270.9 to 369.2)	183.5 (159.6 to 213.4)
Pulmonary aspiration and foreign body in airway	6 (4 to 8)	3 (2 to 3)	0.5 (0.3 to 0.6)	0.2 (0.1 to 0.2)	4 781 (3 979 to 5 796)	2 802 (2 424 to 3 277)	290.8 (247.4 to 344.3)	171.5 (148.3 to 200.7)
Foreign body in eyes	59 (31 to 107)	97 (51 to 174)	6.5 (3.5 to 11.6)	6.1 (3.2 to 10.9)	59 (31 to 107)	97 (51 to 174)	6.5 (3.5 to 11.6)	6.1 (3.2 to 10.9)
Foreign body in other body part	67 (46 to 90)	35 (25 to 46)	5.9 (4.2 to 7.8)	2.1 (1.5 to 2.8)	248 (197 to 292)	97 (81 to 136)	17.9 (14.6 to 20.6)	5.9 (4.9 to 8.2)
Environmental heat and cold exposure	82 (55 to 116)	42 (27 to 60)	7.9 (5.3 to 11.2)	2.6 (1.7 to 3.7)	472 (419 to 532)	188 (163 to 214)	39.2 (35.2 to 43.4)	11.6 (10.1 to 13.2)
Exposure to forces of nature	16 (11 to 21)	20 (15 to 28)	1.4 (1.0 to 1.8)	1.2 (0.9 to 1.7)	96 (76 to 122)	34 (28 to 41)	9.0 (6.9 to 11.9)	2.1 (1.7 to 2.5)
Other unintentional injuries	141 (101 to 190)	126 (90 to 172)	14.3 (10.2 to 19.3)	7.8 (5.6 to 10.7)	1 618 (1 476 to 1 771)	973 (861 to 1 097)	122.4 (112.5 to 133.0)	58.9 (52.1 to 66.4)
