# Supplementary material for: Unintentional injuries in Mexico, 1990–2017: findings from the Global Burden of Disease Study 2017
Source: Inj Prev. 2020 Apr 1;26(Suppl 1):i154–61. doi: 10.1136/injuryprev-2019-043532 (PMC7571365; doi:10.1136/injuryprev-2019-043532)
Supplement: Supplementary data [file injuryprev-2019-043532supp006.pdf]

Death, YLL, incidence, prevalence, YLD, and DALY counts and rates per 100,000 for all injuries in Baja California Sur in 1990 and 2017

Cause	Deaths				Years of life lost			
	All-age counts (95% UI)		Age-standardised rates per 100,000 (95% UI)		All-age counts (95% UI)		Age-standardised rates per 100,000 (95% UI)	
	1990	2017	1990	2017	1990	2017	1990	2017
**Transport and unintentional injuries**	**165 (159 to 171)**	**256 (237 to 274)**	**66.5 (64.3 to 68.5)**	**36.0 (33.5 to 38.3)**	**8 716 (8 330 to 9 113)**	**11 584 (10 670 to 12 493)**	**2 725.0 (2 619.5 to 2 832.5)**	**1 418.7 (1 308.9 to 1 524.0)**
**Transport injuries**	**96 (92 to 101)**	**155 (141 to 169)**	**35.0 (33.5 to 36.5)**	**19.8 (18.2 to 21.4)**	**5 274 (5 001 to 5 552)**	**7 441 (6 794 to 8 121)**	**1 599.0 (1 521.1 to 1 675.7)**	**875.6 (802.5 to 953.1)**
Road injuries	94 (89 to 98)	147 (135 to 160)	33.9 (32.5 to 35.3)	18.8 (17.3 to 20.3)	5 126 (4 861 to 5 393)	7 091 (6 484 to 7 759)	1 551.8 (1 476.7 to 1 625.1)	834.7 (763.8 to 907.6)
Pedestrian road injuries	30 (28 to 32)	43 (40 to 48)	12.9 (12.2 to 13.7)	6.3 (5.7 to 6.8)	1 483 (1 360 to 1 626)	1 851 (1 674 to 2 054)	490.9 (456.6 to 529.8)	231.3 (209.2 to 256.3)
Cyclist road injuries	2 (2 to 2)	6 (5 to 6)	0.8 (0.7 to 0.9)	0.7 (0.7 to 0.8)	115 (102 to 130)	255 (225 to 287)	35.8 (32.2 to 39.8)	30.8 (27.3 to 35.0)
Motorcyclist road injuries	17 (15 to 18)	18 (16 to 21)	5.1 (4.6 to 5.6)	2.1 (1.8 to 2.3)	973 (872 to 1 089)	970 (851 to 1 111)	274.9 (248.5 to 304.8)	109.0 (95.5 to 125.1)
Motor vehicle road injuries	43 (40 to 46)	76 (68 to 85)	14.3 (13.4 to 15.3)	9.3 (8.4 to 10.3)	2 444 (2 255 to 2 632)	3 852 (3 443 to 4 310)	716.5 (667.8 to 766.8)	444.2 (398.7 to 495.4)
Other road injuries	2 (2 to 2)	3 (3 to 4)	0.8 (0.7 to 0.9)	0.5 (0.4 to 0.5)	110 (95 to 129)	162 (141 to 190)	33.6 (29.3 to 38.7)	19.4 (16.9 to 22.5)
Other transport injuries	3 (3 to 3)	7 (6 to 8)	1.1 (1.0 to 1.2)	0.9 (0.8 to 1.1)	149 (133 to 167)	350 (300 to 402)	47.3 (42.2 to 52.5)	40.9 (35.3 to 46.7)
**Unintentional injuries**	**69 (65 to 72)**	**101 (95 to 108)**	**31.5 (30.2 to 32.6)**	**16.2 (15.2 to 17.2)**	**3 442 (3 221 to 3 668)**	**4 144 (3 811 to 4 492)**	**1 126.0 (1 070.1 to 1 183.1)**	**543.1 (498.3 to 589.6)**
Falls	14 (13 to 15)	27 (25 to 29)	9.4 (8.9 to 9.9)	5.3 (4.9 to 5.7)	435 (410 to 462)	712 (640 to 781)	198.5 (188.5 to 209.6)	105.0 (95.5 to 114.4)
Drowning	16 (14 to 17)	18 (16 to 20)	5.5 (5.1 to 5.9)	2.3 (2.1 to 2.5)	920 (829 to 1 007)	925 (828 to 1 030)	268.8 (247.2 to 292.3)	113.0 (101.4 to 126.0)
Fire, heat, and hot substances	6 (5 to 6)	7 (7 to 8)	2.9 (2.8 to 3.1)	1.2 (1.1 to 1.3)	288 (254 to 316)	258 (228 to 285)	94.9 (87.3 to 103.5)	34.9 (31.1 to 38.3)
Poisonings	2 (2 to 2)	2 (2 to 3)	0.7 (0.6 to 0.7)	0.3 (0.3 to 0.3)	110 (99 to 122)	112 (97 to 126)	32.7 (30.0 to 35.9)	13.9 (12.1 to 15.5)
Poisoning by carbon monoxide	0 (0 to 0)	1 (1 to 1)	0.2 (0.1 to 0.2)	0.1 (0.1 to 0.1)	25 (22 to 29)	30 (25 to 34)	7.4 (6.6 to 8.3)	3.6 (3.1 to 4.1)
Poisoning by other means	1 (1 to 2)	2 (2 to 2)	0.5 (0.5 to 0.6)	0.2 (0.2 to 0.3)	85 (76 to 94)	83 (72 to 93)	25.3 (23.0 to 27.8)	10.2 (8.9 to 11.4)
Exposure to mechanical forces	8 (7 to 9)	8 (7 to 9)	3.0 (2.8 to 3.2)	1.1 (1.0 to 1.2)	415 (381 to 453)	375 (338 to 417)	132.1 (122.4 to 141.9)	44.6 (40.4 to 49.3)
Unintentional firearm injuries	3 (2 to 3)	2 (2 to 2)	0.9 (0.8 to 1.1)	0.2 (0.2 to 0.3)	152 (131 to 184)	101 (77 to 139)	45.5 (39.9 to 54.3)	11.5 (8.8 to 15.5)
Other exposure to mechanical forces	5 (5 to 6)	6 (6 to 7)	2.1 (1.9 to 2.2)	0.8 (0.8 to 0.9)	263 (233 to 289)	275 (245 to 311)	86.6 (78.2 to 93.8)	33.1 (29.7 to 37.2)
Adverse effects of medical treatment	5 (4 to 6)	11 (9 to 12)	3.1 (2.1 to 3.4)	2.0 (1.6 to 2.2)	220 (162 to 247)	355 (292 to 402)	81.7 (58.1 to 90.9)	51.6 (42.5 to 58.1)
Animal contact	1 (1 to 1)	1 (1 to 1)	0.4 (0.4 to 0.5)	0.2 (0.1 to 0.2)	67 (57 to 79)	45 (39 to 50)	19.7 (17.3 to 22.6)	6.1 (5.3 to 6.9)
Venomous animal contact	1 (1 to 1)	1 (0 to 1)	0.3 (0.2 to 0.3)	0.1 (0.1 to 0.1)	48 (39 to 60)	25 (19 to 30)	13.8 (11.4 to 16.5)	3.5 (2.7 to 4.2)
Non-venomous animal contact	0 (0 to 0)	0 (0 to 1)	0.1 (0.1 to 0.2)	0.1 (0.1 to 0.1)	18 (16 to 22)	19 (16 to 26)	5.8 (5.1 to 7.0)	2.6 (2.2 to 3.4)
Foreign body	10 (9 to 11)	19 (17 to 21)	3.8 (3.6 to 4.1)	2.7 (2.4 to 3.0)	655 (582 to 738)	974 (842 to 1 129)	186.8 (169.1 to 206.7)	128.7 (110.5 to 150.7)
Pulmonary aspiration and foreign body in airway	10 (9 to 11)	18 (16 to 20)	3.7 (3.4 to 4.0)	2.7 (2.4 to 2.9)	632 (560 to 714)	953 (825 to 1 106)	180.5 (162.9 to 200.1)	126.3 (108.2 to 147.8)
Foreign body in eyes								
Foreign body in other body part	0 (0 to 0)	0 (0 to 1)	0.1 (0.1 to 0.1)	0.0 (0.0 to 0.1)	22 (18 to 27)	20 (16 to 40)	6.4 (5.3 to 7.7)	2.4 (1.9 to 4.8)
Environmental heat and cold exposure	2 (2 to 2)	2 (2 to 3)	1.2 (1.1 to 1.3)	0.4 (0.3 to 0.4)	91 (84 to 98)	93 (80 to 106)	36.4 (33.6 to 39.4)	11.7 (10.1 to 13.2)
Exposure to forces of nature	0 (0 to 0)	0 (0 to 0)	0.1 (0.1 to 0.1)	0.0 (0.0 to 0.0)	14 (13 to 16)	7 (6 to 7)	4.2 (3.8 to 4.6)	0.8 (0.8 to 0.9)
Other unintentional injuries	4 (4 to 5)	6 (5 to 6)	1.4 (1.3 to 1.6)	0.7 (0.6 to 0.8)	232 (209 to 257)	288 (251 to 330)	69.4 (63.2 to 75.8)	33.0 (28.9 to 37.8)

Cause	Incidence				Prevalence			
	All-age counts (95% UI)		Age-standardised rates per 100,000 (95% UI)		All-age counts (95% UI)		Age-standardised rates per 100,000 (95% UI)	
	1990	2017	1990	2017	1990	2017	1990	2017
**Transport and unintentional injuries**	**11 897 (10 858 to 13 043)**	**35 674 (32 147 to 39 572)**	**4 351.3 (3 970.9 to 4 779.8)**	**4 855.7 (4 419.4 to 5 339.9)**	**22 418 (21 025 to 23 916)**	**75 940 (70 922 to 82 068)**	**9 720.1 (9 161.8 to 10 398.2)**	**10 306.2 (9 673.6 to 11 113.4)**
**Transport injuries**	**1 456 (1 262 to 1 701)**	**5 692 (4 881 to 6 600)**	**495.1 (434.4 to 562.8)**	**690.9 (599.2 to 791.6)**	**3 328 (3 097 to 3 557)**	**15 449 (14 257 to 16 760)**	**1 484.1 (1 379.6 to 1 590.7)**	**2 015.4 (1 863.6 to 2 186.4)**
Road injuries	1 395 (1 199 to 1 635)	5 598 (4 786 to 6 509)	475.0 (414.9 to 542.2)	678.4 (585.3 to 778.4)	3 060 (2 839 to 3 288)	14 985 (13 811 to 16 254)	1 375.4 (1 275.0 to 1 479.5)	1 952.7 (1 809.1 to 2 119.2)
Pedestrian road injuries	178 (144 to 218)	646 (518 to 794)	65.5 (54.1 to 78.2)	81.3 (65.9 to 98.8)	643 (554 to 755)	2 884 (2 476 to 3 389)	296.4 (259.3 to 343.9)	385.2 (332.9 to 449.5)
Cyclist road injuries	159 (120 to 200)	555 (433 to 713)	51.5 (40.8 to 65.7)	70.2 (54.7 to 88.6)	263 (221 to 310)	1 202 (1 011 to 1 427)	112.1 (94.9 to 129.4)	156.6 (132.6 to 183.1)
Motorcyclist road injuries	182 (143 to 226)	523 (393 to 672)	57.3 (46.0 to 69.5)	61.7 (47.5 to 78.3)	622 (538 to 719)	2 335 (1 995 to 2 738)	268.9 (233.0 to 310.0)	294.0 (251.6 to 343.9)
Motor vehicle road injuries	661 (550 to 789)	3 266 (2 757 to 3 882)	225.5 (191.5 to 262.7)	387.1 (326.6 to 456.4)	1 126 (988 to 1 315)	7 099 (6 199 to 8 346)	520.0 (452.3 to 601.4)	921.6 (802.6 to 1 077.2)
Other road injuries	216 (163 to 277)	609 (457 to 768)	75.2 (58.7 to 92.3)	78.0 (59.2 to 97.6)	406 (337 to 496)	1 465 (1 215 to 1 786)	178.1 (148.9 to 214.0)	195.3 (162.6 to 236.6)
Other transport injuries	60 (47 to 79)	94 (78 to 115)	20.2 (16.5 to 24.9)	12.5 (10.4 to 15.3)	268 (222 to 322)	465 (396 to 537)	108.7 (92.3 to 126.4)	62.7 (54.2 to 71.6)
**Unintentional injuries**	**10 442 (9 410 to 11 602)**	**29 982 (26 721 to 33 703)**	**3 856.2 (3 474.0 to 4 278.0)**	**4 164.8 (3 735.8 to 4 641.0)**	**19 090 (17 724 to 20 582)**	**60 490 (55 844 to 66 507)**	**8 236.0 (7 681.3 to 8 884.4)**	**8 290.8 (7 673.5 to 9 105.3)**
Falls	4 527 (3 926 to 5 184)	16 574 (14 518 to 18 799)	1 876.6 (1 664.7 to 2 108.8)	2 451.5 (2 147.9 to 2 755.1)	7 898 (6 928 to 9 037)	33 278 (29 256 to 38 051)	3 737.9 (3 326.6 to 4 242.7)	4 791.6 (4 253.4 to 5 497.5)
Drowning	15 (13 to 17)	19 (16 to 24)	4.9 (4.3 to 5.6)	2.5 (2.1 to 3.0)	66 (58 to 76)	114 (100 to 132)	30.5 (27.1 to 34.7)	15.5 (13.7 to 17.9)
Fire, heat, and hot substances	401 (319 to 505)	710 (581 to 864)	112.3 (92.0 to 136.7)	92.3 (76.1 to 114.0)	3 789 (3 150 to 4 806)	8 528 (7 026 to 10 771)	1 418.6 (1 194.7 to 1 743.2)	1 077.3 (893.6 to 1 358.6)
Poisonings	109 (77 to 154)	232 (156 to 325)	27.8 (20.2 to 38.7)	31.4 (21.3 to 44.4)	97 (74 to 120)	301 (220 to 381)	31.5 (24.4 to 38.9)	36.7 (26.9 to 46.5)
Poisoning by carbon monoxide	22 (13 to 37)	47 (29 to 80)	6.2 (3.8 to 9.6)	6.2 (3.1 to 10.7)	15 (12 to 20)	43 (32 to 56)	5.2 (4.0 to 6.7)	5.3 (4.0 to 6.9)
Poisoning by other means	86 (60 to 127)	185 (123 to 269)	21.6 (15.2 to 31.1)	25.2 (16.6 to 36.9)	82 (59 to 104)	258 (180 to 337)	26.3 (19.3 to 33.3)	31.5 (22.0 to 41.1)
Exposure to mechanical forces	2 310 (1 946 to 2 759)	4 900 (4 154 to 5 766)	720.6 (616.8 to 851.7)	592.6 (504.6 to 694.9)	4 391 (3 882 to 5 089)	11 866 (10 292 to 13 870)	1 876.2 (1 645.5 to 2 147.5)	1 523.1 (1 326.3 to 1 771.0)
Unintentional firearm injuries	140 (98 to 192)	211 (135 to 301)	41.1 (29.9 to 55.0)	25.1 (16.2 to 35.4)	514 (405 to 616)	1 036 (820 to 1 246)	218.7 (176.2 to 261.8)	134.0 (107.0 to 159.7)
Other exposure to mechanical forces	2 170 (1 830 to 2 599)	4 689 (3 964 to 5 547)	679.4 (583.6 to 800.2)	567.5 (482.0 to 667.2)	3 877 (3 275 to 4 535)	10 830 (9 236 to 12 914)	1 657.6 (1 418.7 to 1 938.4)	1 389.1 (1 188.7 to 1 640.9)
Adverse effects of medical treatment	381 (321 to 438)	1 355 (1 156 to 1 558)	148.4 (128.3 to 167.6)	178.9 (154.0 to 203.2)	29 (23 to 36)	104 (80 to 128)	11.4 (8.8 to 14.0)	13.7 (10.6 to 16.9)
Animal contact	665 (530 to 838)	1 359 (1 159 to 1 598)	217.0 (179.3 to 262.4)	177.7 (152.5 to 207.7)	700 (597 to 822)	1 691 (1 472 to 1 944)	285.6 (249.0 to 326.9)	224.5 (196.3 to 256.4)
Venomous animal contact	235 (173 to 317)	489 (385 to 619)	76.3 (57.9 to 100.0)	63.6 (50.2 to 79.7)	155 (127 to 183)	363 (305 to 421)	59.2 (49.8 to 68.7)	47.1 (39.7 to 54.3)
Non-venomous animal contact	431 (327 to 550)	870 (715 to 1 063)	140.7 (111.4 to 174.5)	114.1 (93.7 to 139.5)	544 (448 to 663)	1 328 (1 115 to 1 573)	226.4 (190.8 to 266.9)	177.4 (150.0 to 208.5)
Foreign body	1 205 (590 to 2 091)	3 507 (1 412 to 6 407)	476.0 (209.4 to 793.2)	466.4 (182.1 to 805.5)	537 (406 to 790)	1 331 (977 to 1 912)	218.4 (169.2 to 294.4)	184.2 (137.1 to 257.3)
Pulmonary aspiration and foreign body in airway	34 (25 to 46)	36 (26 to 47)	9.5 (7.2 to 12.3)	4.9 (3.6 to 6.6)	25 (18 to 34)	34 (27 to 42)	8.6 (6.4 to 11.4)	4.3 (3.4 to 5.4)
Foreign body in eyes	1 075 (460 to 1 908)	3 304 (1 208 to 6 191)	438.6 (168.8 to 752.5)	439.3 (154.8 to 782.9)	207 (100 to 384)	679 (344 to 1 264)	98.7 (55.1 to 164.2)	99.5 (54.7 to 169.2)
Foreign body in other body part	95 (70 to 130)	168 (129 to 215)	27.9 (21.3 to 36.3)	22.2 (17.0 to 28.0)	304 (241 to 388)	618 (515 to 746)	111.1 (89.6 to 138.8)	80.5 (67.5 to 96.5)
Environmental heat and cold exposure	159 (116 to 210)	178 (129 to 233)	56.0 (43.6 to 68.5)	25.0 (18.5 to 32.7)	557 (448 to 680)	733 (594 to 858)	226.4 (188.4 to 268.6)	97.3 (82.8 to 115.4)
Exposure to forces of nature	38 (31 to 47)	9 (7 to 11)	10.7 (9.0 to 12.9)	1.1 (0.9 to 1.3)	122 (88 to 167)	547 (375 to 778)	44.1 (32.0 to 60.0)	65.8 (45.6 to 92.8)
Other unintentional injuries	632 (521 to 780)	1 188 (948 to 1 379)	205.9 (172.7 to 245.6)	144.9 (119.5 to 175.4)	905 (778 to 1 058)	1 999 (1 720 to 2 307)	375.4 (323.4 to 430.1)	260.9 (226.0 to 298.9)

Cause	Years lived with disability				Disability adjusted life years			
	All-age counts (95% UI)		Age-standardised rates per 100,000 (95% UI)		All-age counts (95% UI)		Age-standardised rates per 100,000 (95% UI)	
	1990	2017	1990	2017	1990	2017	1990	2017
**Transport and unintentional injuries**	**976 (709 to 1 310)**	**3 315 (2 391 to 4 478)**	**419.5 (302.4 to 556.8)**	**449.8 (323.1 to 601.4)**	**9 692 (9 208 to 10 211)**	**14 900 (13 527 to 16 347)**	**3 144.5 (2 973.5 to 3 321.6)**	**1 868.5 (1 694.9 to 2 064.1)**
**Transport injuries**	**220 (161 to 293)**	**943 (679 to 1 258)**	**94.0 (68.6 to 124.7)**	**120.2 (86.4 to 160.2)**	**5 494 (5 206 to 5 771)**	**8 384 (7 643 to 9 122)**	**1 693.0 (1 612.9 to 1 773.8)**	**995.8 (910.2 to 1 080.5)**
Road injuries	204 (148 to 273)	918 (661 to 1 226)	87.7 (64.0 to 117.1)	117.0 (84.0 to 156.3)	5 329 (5 052 to 5 602)	8 009 (7 300 to 8 722)	1 639.5 (1 560.9 to 1 717.8)	951.8 (869.6 to 1 034.4)
Pedestrian road injuries	45 (31 to 60)	184 (129 to 252)	19.6 (14.0 to 26.7)	24.0 (16.9 to 32.9)	1 528 (1 403 to 1 670)	2 034 (1 848 to 2 259)	510.5 (476.3 to 549.5)	255.2 (232.5 to 280.9)
Cyclist road injuries	18 (12 to 23)	72 (49 to 96)	7.2 (5.1 to 9.5)	9.2 (6.4 to 12.2)	133 (119 to 147)	326 (289 to 367)	43.0 (38.9 to 47.2)	40.0 (35.4 to 45.0)
Motorcyclist road injuries	42 (29 to 56)	142 (96 to 194)	17.3 (12.0 to 23.5)	17.5 (11.9 to 23.9)	1 015 (912 to 1 130)	1 112 (977 to 1 261)	292.2 (264.8 to 322.9)	126.5 (111.5 to 143.4)
Motor vehicle road injuries	78 (57 to 104)	450 (324 to 600)	34.2 (25.0 to 45.5)	56.9 (41.2 to 76.1)	2 522 (2 328 to 2 716)	4 302 (3 857 to 4 780)	750.7 (701.2 to 802.0)	501.1 (449.7 to 555.9)
Other road injuries	22 (16 to 30)	72 (51 to 99)	9.4 (6.8 to 12.7)	9.4 (6.7 to 13.0)	133 (115 to 154)	234 (204 to 270)	43.0 (37.8 to 48.7)	28.8 (25.1 to 33.3)
Other transport injuries	16 (11 to 22)	25 (17 to 33)	6.3 (4.5 to 8.4)	3.2 (2.3 to 4.3)	165 (148 to 183)	375 (322 to 428)	53.5 (48.3 to 59.0)	44.2 (38.4 to 49.9)
**Unintentional injuries**	**756 (548 to 1 020)**	**2 372 (1 696 to 3 225)**	**325.5 (234.2 to 439.8)**	**329.5 (235.3 to 441.8)**	**4 198 (3 895 to 4 541)**	**6 516 (5 729 to 7 456)**	**1 451.5 (1 341.2 to 1 578.8)**	**872.6 (763.0 to 1 004.4)**
Falls	446 (325 to 590)	1 674 (1 210 to 2 225)	204.0 (147.8 to 268.1)	239.9 (171.4 to 319.6)	882 (762 to 1 027)	2 386 (1 906 to 2 957)	402.5 (347.3 to 468.3)	344.9 (274.6 to 429.4)
Drowning	4 (3 to 5)	6 (4 to 8)	1.6 (1.1 to 2.3)	0.8 (0.6 to 1.1)	924 (834 to 1 011)	931 (834 to 1 036)	271.4 (248.7 to 293.9)	113.8 (102.2 to 126.8)
Fire, heat, and hot substances	79 (48 to 125)	164 (96 to 266)	28.6 (17.1 to 45.2)	20.6 (12.1 to 33.5)	361 (318 to 417)	422 (347 to 531)	123.4 (110.2 to 142.3)	55.4 (46.1 to 69.2)
Poisonings	11 (7 to 17)	35 (21 to 54)	3.6 (2.2 to 5.2)	4.3 (2.5 to 6.5)	121 (110 to 135)	148 (126 to 169)	36.3 (33.2 to 39.6)	18.1 (15.6 to 20.7)
Poisoning by carbon monoxide	2 (1 to 3)	5 (3 to 7)	0.5 (0.3 to 0.8)	0.5 (0.3 to 0.9)	27 (24 to 31)	34 (29 to 39)	8.0 (7.1 to 8.9)	4.2 (3.5 to 4.7)
Poisoning by other means	10 (6 to 15)	31 (18 to 48)	3.0 (1.8 to 4.6)	3.7 (2.2 to 5.8)	94 (85 to 105)	113 (96 to 132)	28.3 (25.7 to 31.1)	14.0 (11.9 to 16.2)
Exposure to mechanical forces	101 (68 to 145)	255 (172 to 371)	42.1 (28.6 to 60.5)	32.5 (21.9 to 47.2)	515 (467 to 567)	630 (535 to 760)	174.2 (157.6 to 193.3)	77.0 (65.9 to 93.4)
Unintentional firearm injuries	10 (7 to 16)	19 (12 to 30)	4.2 (2.7 to 6.6)	2.5 (1.6 to 3.8)	162 (141 to 196)	120 (95 to 141)	49.7 (43.8 to 58.8)	13.9 (11.0 to 16.4)
Other exposure to mechanical forces	90 (61 to 130)	236 (158 to 346)	37.9 (25.7 to 54.4)	30.0 (20.2 to 43.8)	353 (311 to 397)	510 (425 to 631)	124.5 (109.7 to 141.3)	63.1 (52.5 to 77.9)
Adverse effects of medical treatment	4 (2 to 6)	14 (9 to 21)	1.5 (0.9 to 2.3)	1.8 (1.1 to 2.8)	224 (166 to 250)	369 (306 to 415)	83.2 (59.5 to 91.9)	53.4 (44.6 to 59.9)
Animal contact	18 (12 to 26)	39 (26 to 56)	6.7 (4.5 to 9.5)	5.0 (3.3 to 7.1)	85 (74 to 99)	84 (68 to 102)	26.4 (23.1 to 30.4)	11.1 (9.1 to 13.5)
Venomous animal contact	10 (6 to 15)	21 (13 to 30)	3.4 (2.2 to 4.9)	2.5 (1.6 to 3.6)	59 (49 to 70)	46 (37 to 56)	17.2 (14.6 to 20.2)	6.0 (4.9 to 7.3)
Non-venomous animal contact	8 (5 to 13)	18 (11 to 30)	3.3 (2.0 to 5.3)	2.5 (1.4 to 4.0)	26 (22 to 33)	38 (29 to 51)	9.2 (7.6 to 11.4)	5.0 (3.9 to 6.8)
Foreign body	32 (21 to 49)	75 (46 to 121)	12.9 (8.4 to 19.5)	10.4 (6.3 to 16.8)	687 (612 to 771)	1 048 (910 to 1 211)	199.7 (181.3 to 220.7)	139.0 (120.0 to 162.1)
Pulmonary aspiration and foreign body in airway	1 (1 to 2)	1 (1 to 2)	0.4 (0.3 to 0.6)	0.2 (0.1 to 0.2)	634 (562 to 715)	955 (827 to 1 108)	180.9 (163.3 to 200.5)	126.5 (108.3 to 147.9)
Foreign body in eyes	14 (7 to 25)	45 (22 to 85)	6.6 (3.4 to 11.8)	6.6 (3.4 to 12.1)	14 (7 to 25)	45 (22 to 85)	6.6 (3.4 to 11.8)	6.6 (3.4 to 12.1)
Foreign body in other body part	17 (12 to 23)	28 (20 to 38)	5.9 (4.2 to 7.8)	3.6 (2.5 to 4.8)	39 (33 to 47)	48 (38 to 69)	12.3 (10.3 to 14.5)	6.0 (4.7 to 8.5)
Environmental heat and cold exposure	21 (14 to 31)	27 (17 to 38)	8.7 (5.8 to 12.3)	3.7 (2.4 to 5.2)	112 (102 to 124)	120 (103 to 138)	45.0 (40.9 to 49.3)	15.4 (13.3 to 17.7)
Exposure to forces of nature	6 (4 to 8)	17 (13 to 25)	2.1 (1.6 to 2.8)	2.1 (1.5 to 2.9)	20 (18 to 23)	24 (19 to 32)	6.3 (5.7 to 7.1)	2.9 (2.3 to 3.7)
Other unintentional injuries	34 (24 to 46)	66 (46 to 90)	13.6 (9.7 to 18.5)	8.6 (6.0 to 11.7)	266 (240 to 293)	354 (313 to 404)	83.0 (75.8 to 90.8)	41.6 (36.8 to 47.3)
