# Supplementary material for: Unintentional injuries in Mexico, 1990–2017: findings from the Global Burden of Disease Study 2017
Source: Inj Prev. 2020 Apr 1;26(Suppl 1):i154–61. doi: 10.1136/injuryprev-2019-043532 (PMC7571365; doi:10.1136/injuryprev-2019-043532)
Supplement: Supplementary data [file injuryprev-2019-043532supp008.pdf]

Death, YLL, incidence, prevalence, YLD, and DALY counts and rates per 100,000 for all injuries in Campeche in 1990 and 2017

Cause	Deaths				Years of life lost			
	All-age counts (95% UI)		Age-standardised rates per 100,000 (95% UI)		All-age counts (95% UI)		Age-standardised rates per 100,000 (95% UI)	
	1990	2017	1990	2017	1990	2017	1990	2017
**Transport and unintentional injuries**	**316 (297 to 338)**	**345 (319 to 369)**	**71.3 (68.5 to 74.5)**	**38.9 (36.1 to 41.6)**	**17 775 (16 297 to 19 571)**	**15 067 (13 875 to 16 260)**	**3 156.7 (2 961.4 to 3 391.3)**	**1 583.0 (1 459.2 to 1 705.8)**
**Transport injuries**	**145 (137 to 154)**	**177 (158 to 194)**	**31.2 (29.8 to 32.7)**	**19.0 (17.0 to 20.7)**	**8 145 (7 519 to 8 852)**	**8 498 (7 598 to 9 292)**	**1 483.9 (1 394.2 to 1 583.3)**	**873.3 (783.1 to 954.1)**
Road injuries	134 (126 to 142)	158 (140 to 173)	28.7 (27.4 to 30.2)	16.9 (15.3 to 18.5)	7 510 (6 923 to 8 160)	7 582 (6 831 to 8 306)	1 367.5 (1 282.8 to 1 459.4)	779.2 (703.1 to 851.3)
Pedestrian road injuries	65 (60 to 70)	67 (61 to 74)	15.1 (14.2 to 16.0)	7.5 (6.8 to 8.3)	3 521 (3 134 to 3 950)	2 893 (2 593 to 3 201)	650.6 (596.3 to 709.5)	303.9 (272.9 to 335.7)
Cyclist road injuries	5 (4 to 5)	9 (8 to 10)	1.0 (0.9 to 1.1)	1.0 (0.8 to 1.1)	266 (235 to 301)	407 (349 to 462)	49.3 (43.8 to 54.8)	42.3 (36.3 to 47.9)
Motorcyclist road injuries	22 (20 to 24)	25 (22 to 29)	4.2 (3.8 to 4.6)	2.5 (2.2 to 2.9)	1 294 (1 149 to 1 441)	1 390 (1 213 to 1 596)	229.6 (206.3 to 253.6)	139.0 (121.1 to 159.2)
Motor vehicle road injuries	38 (35 to 42)	52 (45 to 59)	7.7 (7.1 to 8.3)	5.4 (4.7 to 6.1)	2 212 (2 004 to 2 437)	2 692 (2 322 to 3 010)	399.2 (365.8 to 436.4)	273.4 (236.1 to 305.5)
Other road injuries	4 (3 to 4)	4 (4 to 5)	0.8 (0.7 to 0.9)	0.4 (0.4 to 0.5)	217 (181 to 255)	199 (170 to 231)	38.7 (32.6 to 44.6)	20.5 (17.5 to 23.7)
Other transport injuries	11 (10 to 13)	20 (17 to 22)	2.5 (2.3 to 2.7)	2.1 (1.8 to 2.4)	635 (567 to 707)	916 (782 to 1 054)	116.3 (105.1 to 128.5)	94.1 (80.8 to 107.9)
**Unintentional injuries**	**170 (158 to 185)**	**167 (157 to 179)**	**40.1 (38.2 to 42.2)**	**19.9 (18.6 to 21.2)**	**9 630 (8 619 to 10 868)**	**6 569 (6 081 to 7 116)**	**1 672.9 (1 541.8 to 1 830.8)**	**709.7 (657.7 to 766.9)**
Falls	30 (29 to 32)	50 (46 to 54)	11.0 (10.4 to 11.6)	6.6 (6.0 to 7.1)	1 083 (994 to 1 183)	1 230 (1 132 to 1 335)	255.4 (239.7 to 271.2)	141.8 (130.4 to 153.5)
Drowning	49 (44 to 55)	34 (31 to 37)	9.3 (8.6 to 10.1)	3.6 (3.3 to 4.0)	3 094 (2 709 to 3 572)	1 714 (1 547 to 1 894)	505.3 (454.0 to 565.7)	178.8 (161.6 to 197.5)
Fire, heat, and hot substances	11 (10 to 13)	9 (8 to 10)	2.7 (2.5 to 3.0)	1.1 (1.0 to 1.2)	685 (529 to 778)	297 (272 to 325)	107.1 (93.5 to 124.6)	32.8 (30.0 to 35.9)
Poisonings	7 (7 to 8)	5 (5 to 6)	1.4 (1.3 to 1.6)	0.6 (0.6 to 0.7)	484 (420 to 570)	261 (235 to 290)	77.1 (68.6 to 88.3)	27.5 (24.8 to 30.4)
Poisoning by carbon monoxide	2 (2 to 2)	2 (1 to 2)	0.4 (0.3 to 0.4)	0.2 (0.2 to 0.2)	110 (94 to 130)	75 (65 to 87)	18.4 (16.0 to 21.3)	7.9 (6.8 to 9.0)
Poisoning by other means	6 (5 to 7)	4 (4 to 4)	1.1 (1.0 to 1.2)	0.4 (0.4 to 0.5)	374 (320 to 444)	185 (168 to 206)	58.7 (51.6 to 67.7)	19.6 (17.7 to 21.8)
Exposure to mechanical forces	19 (18 to 21)	14 (13 to 15)	4.2 (4.0 to 4.5)	1.6 (1.4 to 1.7)	1 077 (982 to 1 184)	627 (564 to 692)	197.6 (182.8 to 214.5)	65.1 (58.8 to 71.6)
Unintentional firearm injuries	7 (6 to 9)	4 (3 to 4)	1.5 (1.3 to 1.8)	0.4 (0.3 to 0.5)	421 (368 to 500)	189 (158 to 221)	75.5 (66.7 to 90.3)	19.2 (16.0 to 22.3)
Other exposure to mechanical forces	12 (11 to 13)	10 (9 to 11)	2.7 (2.4 to 3.0)	1.1 (1.0 to 1.3)	656 (571 to 738)	438 (390 to 486)	122.1 (106.9 to 135.3)	45.9 (40.9 to 50.8)
Adverse effects of medical treatment	11 (7 to 18)	17 (14 to 19)	3.1 (2.3 to 3.5)	2.1 (1.8 to 2.4)	551 (351 to 671)	547 (460 to 623)	100.7 (67.5 to 116.9)	61.9 (52.0 to 70.4)
Animal contact	6 (5 to 7)	3 (3 to 4)	1.2 (1.0 to 1.4)	0.4 (0.4 to 0.5)	412 (328 to 527)	139 (124 to 156)	62.4 (51.7 to 77.1)	15.3 (13.7 to 17.2)
Venomous animal contact	5 (4 to 6)	3 (2 to 3)	1.0 (0.8 to 1.1)	0.3 (0.3 to 0.3)	356 (277 to 464)	101 (87 to 116)	52.7 (42.5 to 66.7)	11.2 (9.6 to 12.9)
Non-venomous animal contact	1 (1 to 1)	1 (1 to 1)	0.2 (0.2 to 0.3)	0.1 (0.1 to 0.1)	56 (42 to 71)	37 (31 to 50)	9.6 (7.2 to 11.7)	4.0 (3.4 to 5.5)
Foreign body	22 (19 to 26)	23 (21 to 26)	4.1 (3.8 to 4.6)	2.6 (2.4 to 2.9)	1 525 (1 292 to 1 823)	1 208 (1 052 to 1 405)	229.0 (199.4 to 266.6)	130.4 (113.9 to 151.8)
Pulmonary aspiration and foreign body in airway	21 (19 to 25)	23 (21 to 25)	4.0 (3.6 to 4.4)	2.6 (2.4 to 2.9)	1 468 (1 243 to 1 752)	1 179 (1 025 to 1 379)	220.0 (191.5 to 256.2)	127.4 (110.6 to 148.6)
Foreign body in eyes								
Foreign body in other body part	1 (1 to 1)	1 (0 to 1)	0.1 (0.1 to 0.2)	0.1 (0.0 to 0.1)	57 (44 to 71)	29 (23 to 35)	8.9 (7.1 to 10.8)	3.0 (2.4 to 3.6)
Environmental heat and cold exposure	3 (3 to 3)	2 (2 to 2)	0.9 (0.8 to 0.9)	0.3 (0.2 to 0.3)	149 (132 to 169)	78 (70 to 86)	29.9 (27.4 to 32.7)	8.4 (7.5 to 9.2)
Exposure to forces of nature	0 (0 to 0)	0 (0 to 0)	0.1 (0.1 to 0.1)	0.0 (0.0 to 0.0)	17 (15 to 19)	8 (7 to 8)	2.9 (2.6 to 3.1)	0.8 (0.7 to 0.9)
Other unintentional injuries	10 (9 to 11)	9 (8 to 10)	2.1 (1.9 to 2.2)	1.0 (0.9 to 1.1)	603 (541 to 666)	460 (404 to 523)	105.6 (95.5 to 115.8)	46.8 (41.2 to 53.1)

Cause	Incidence				Prevalence			
	All-age counts (95% UI)		Age-standardised rates per 100,000 (95% UI)		All-age counts (95% UI)		Age-standardised rates per 100,000 (95% UI)	
	1990	2017	1990	2017	1990	2017	1990	2017
**Transport and unintentional injuries**	**20 576 (19 141 to 22 123)**	**41 278 (37 963 to 44 692)**	**4 278.1 (4 003.5 to 4 573.6)**	**4 575.0 (4 222.7 to 4 941.2)**	**39 778 (37 335 to 42 737)**	**88 213 (82 397 to 95 803)**	**10 193.4 (9 598.1 to 10 951.0)**	**9 943.8 (9 293.6 to 10 803.0)**
**Transport injuries**	**1 927 (1 659 to 2 226)**	**4 830 (4 200 to 5 531)**	**391.9 (340.2 to 443.3)**	**508.1 (443.8 to 578.3)**	**4 727 (4 402 to 5 032)**	**13 580 (12 622 to 14 565)**	**1 260.5 (1 176.2 to 1 341.7)**	**1 510.1 (1 401.2 to 1 619.7)**
Road injuries	1 759 (1 493 to 2 054)	4 664 (4 036 to 5 364)	359.0 (303.9 to 410.7)	489.9 (425.5 to 559.3)	3 998 (3 724 to 4 290)	12 757 (11 863 to 13 744)	1 077.8 (1 005.6 to 1 154.0)	1 417.2 (1 317.5 to 1 526.6)
Pedestrian road injuries	278 (222 to 343)	480 (384 to 590)	60.2 (49.0 to 72.1)	51.5 (41.2 to 62.5)	998 (858 to 1 174)	2 118 (1 838 to 2 470)	274.9 (240.7 to 320.8)	240.2 (208.8 to 279.8)
Cyclist road injuries	334 (260 to 425)	758 (630 to 935)	63.1 (53.0 to 77.0)	81.1 (65.2 to 99.2)	557 (465 to 660)	1 637 (1 385 to 1 914)	140.9 (119.3 to 168.6)	181.2 (154.5 to 210.6)
Motorcyclist road injuries	250 (199 to 309)	647 (508 to 808)	48.4 (39.0 to 58.6)	66.5 (52.3 to 82.7)	871 (752 to 1 004)	2 852 (2 438 to 3 348)	229.6 (199.0 to 265.1)	310.6 (266.4 to 364.2)
Motor vehicle road injuries	603 (492 to 720)	2 174 (1 811 to 2 593)	126.1 (104.6 to 148.6)	225.7 (188.4 to 266.3)	1 039 (913 to 1 209)	4 742 (4 122 to 5 551)	290.4 (253.1 to 337.6)	527.2 (457.8 to 615.4)
Other road injuries	294 (222 to 372)	604 (477 to 739)	61.2 (48.4 to 74.7)	65.1 (51.6 to 79.3)	532 (441 to 638)	1 409 (1 177 to 1 699)	142.0 (118.6 to 169.2)	158.0 (132.2 to 189.7)
Other transport injuries	167 (138 to 212)	166 (133 to 209)	32.9 (28.1 to 39.4)	18.3 (14.6 to 23.0)	729 (607 to 853)	823 (703 to 951)	182.7 (155.6 to 211.3)	93.0 (79.8 to 106.7)
**Unintentional injuries**	**18 649 (17 246 to 20 202)**	**36 449 (33 331 to 39 832)**	**3 886.2 (3 606.6 to 4 191.8)**	**4 066.9 (3 722.6 to 4 423.8)**	**35 051 (32 631 to 37 884)**	**74 633 (68 896 to 81 989)**	**8 932.9 (8 350.0 to 9 667.3)**	**8 433.6 (7 800.7 to 9 257.7)**
Falls	7 912 (6 879 to 9 053)	18 896 (16 747 to 21 393)	1 842.7 (1 637.5 to 2 068.2)	2 187.3 (1 937.7 to 2 465.9)	13 555 (11 927 to 15 481)	37 076 (33 027 to 42 165)	3 701.2 (3 311.9 to 4 223.1)	4 299.7 (3 835.3 to 4 904.0)
Drowning	40 (35 to 47)	30 (24 to 39)	7.7 (6.7 to 8.7)	3.3 (2.7 to 4.2)	182 (159 to 211)	183 (160 to 211)	49.0 (43.4 to 56.4)	21.0 (18.5 to 24.3)
Fire, heat, and hot substances	706 (562 to 887)	771 (633 to 940)	109.0 (89.3 to 133.1)	83.4 (68.4 to 101.5)	6 570 (5 417 to 8 366)	8 811 (7 302 to 11 367)	1 447.3 (1 207.1 to 1 783.0)	955.6 (795.5 to 1 227.2)
Poisonings	261 (195 to 337)	336 (254 to 433)	37.0 (28.2 to 46.9)	36.4 (27.4 to 46.8)	220 (171 to 269)	384 (286 to 477)	42.8 (33.6 to 52.1)	40.7 (30.4 to 50.5)
Poisoning by carbon monoxide	51 (33 to 77)	68 (39 to 106)	8.0 (5.6 to 11.4)	7.4 (4.3 to 11.5)	34 (27 to 44)	55 (42 to 72)	6.9 (5.4 to 8.8)	5.9 (4.5 to 7.7)
Poisoning by other means	210 (154 to 278)	268 (193 to 355)	28.9 (21.6 to 37.5)	29.0 (20.8 to 38.6)	185 (137 to 232)	329 (237 to 416)	35.8 (26.7 to 44.6)	34.8 (25.1 to 44.0)
Exposure to mechanical forces	5 020 (4 316 to 5 859)	8 684 (7 479 to 10 007)	936.6 (819.4 to 1 076.1)	912.5 (787.1 to 1 047.6)	9 606 (8 387 to 11 075)	21 159 (18 291 to 24 802)	2 489.4 (2 187.2 to 2 868.9)	2 330.5 (2 014.4 to 2 722.0)
Unintentional firearm injuries	242 (177 to 327)	255 (180 to 340)	44.1 (33.2 to 58.0)	26.5 (18.8 to 35.0)	939 (742 to 1 136)	1 335 (1 083 to 1 606)	248.8 (198.9 to 303.3)	148.7 (121.7 to 178.6)
Other exposure to mechanical forces	4 779 (4 093 to 5 575)	8 429 (7 281 to 9 718)	892.6 (778.7 to 1 025.7)	886.0 (766.4 to 1 018.0)	8 667 (7 383 to 10 202)	19 824 (16 856 to 23 559)	2 240.6 (1 920.6 to 2 628.0)	2 181.8 (1 853.9 to 2 582.3)
Adverse effects of medical treatment	411 (341 to 485)	896 (688 to 1 029)	95.9 (81.0 to 111.2)	98.2 (77.5 to 119.6)	31 (24 to 39)	64 (48 to 83)	7.3 (5.6 to 9.2)	7.1 (5.4 to 8.8)
Animal contact	1 599 (1 348 to 1 924)	1 992 (1 740 to 2 290)	307.3 (266.7 to 356.2)	218.0 (191.4 to 249.2)	1 640 (1 410 to 1 898)	2 448 (2 136 to 2 808)	405.7 (355.4 to 459.1)	273.8 (239.1 to 313.7)
Venomous animal contact	582 (450 to 750)	650 (514 to 806)	111.9 (88.3 to 139.4)	71.0 (56.5 to 87.8)	382 (315 to 457)	445 (376 to 517)	88.0 (74.1 to 102.9)	48.9 (41.3 to 56.6)
Non-venomous animal contact	1 017 (832 to 1 250)	1 341 (1 148 to 1 591)	195.4 (163.1 to 234.4)	147.0 (125.5 to 173.8)	1 258 (1 039 to 1 514)	2 002 (1 695 to 2 361)	317.7 (268.3 to 372.3)	224.9 (191.4 to 263.8)
Foreign body	1 313 (774 to 1 923)	3 373 (1 835 to 5 376)	281.2 (149.8 to 431.3)	365.5 (195.3 to 575.6)	668 (528 to 859)	1 165 (856 to 1 608)	156.4 (126.3 to 197.6)	131.5 (98.1 to 180.0)
Pulmonary aspiration and foreign body in airway	58 (40 to 80)	36 (26 to 49)	8.6 (6.3 to 11.4)	4.0 (2.8 to 5.4)	39 (27 to 54)	33 (25 to 39)	7.8 (5.7 to 10.5)	3.4 (2.8 to 4.2)
Foreign body in eyes	1 120 (602 to 1 719)	3 210 (1 660 to 5 232)	250.0 (117.0 to 402.0)	347.4 (176.4 to 558.2)	216 (110 to 378)	686 (392 to 1 123)	56.8 (32.7 to 89.6)	78.3 (46.5 to 124.5)
Foreign body in other body part	135 (88 to 207)	128 (95 to 166)	22.7 (15.6 to 32.6)	14.1 (10.5 to 18.1)	412 (328 to 526)	447 (376 to 530)	91.8 (74.8 to 114.6)	49.8 (42.1 to 59.1)
Environmental heat and cold exposure	268 (187 to 366)	206 (151 to 288)	52.6 (40.3 to 65.7)	23.3 (17.2 to 32.1)	917 (738 to 1 118)	797 (675 to 951)	221.3 (184.9 to 263.8)	90.5 (76.9 to 108.0)
Exposure to forces of nature	44 (36 to 55)	10 (9 to 12)	7.3 (6.1 to 8.8)	1.1 (0.9 to 1.3)	143 (102 to 196)	337 (239 to 466)	27.0 (19.8 to 36.8)	35.6 (25.2 to 49.0)
Other unintentional injuries	1 074 (895 to 1 299)	1 312 (1 115 to 1 546)	208.9 (177.7 to 244.1)	142.9 (121.4 to 167.3)	1 520 (1 311 to 1 759)	2 209 (1 921 to 2 517)	385.5 (334.9 to 441.8)	247.5 (215.9 to 280.7)

Cause	Years lived with disability				Disability adjusted life years			
	All-age counts (95% UI)		Age-standardised rates per 100,000 (95% UI)		All-age counts (95% UI)		Age-standardised rates per 100,000 (95% UI)	
	1990	2017	1990	2017	1990	2017	1990	2017
**Transport and unintentional injuries**	**1 732 (1 258 to 2 296)**	**3 620 (2 584 to 4 894)**	**437.4 (317.9 to 578.0)**	**408.0 (291.9 to 551.1)**	**19 507 (17 890 to 21 351)**	**18 686 (17 171 to 20 373)**	**3 594.1 (3 349.5 to 3 869.3)**	**1 991.0 (1 826.6 to 2 178.6)**
**Transport injuries**	**323 (235 to 426)**	**818 (596 to 1 092)**	**82.6 (60.4 to 108.7)**	**89.7 (65.4 to 119.7)**	**8 468 (7 848 to 9 172)**	**9 316 (8 417 to 10 156)**	**1 566.5 (1 473.4 to 1 665.5)**	**963.0 (870.4 to 1 052.0)**
Road injuries	276 (203 to 367)	775 (561 to 1 035)	71.6 (52.5 to 94.7)	84.9 (61.6 to 113.3)	7 786 (7 192 to 8 449)	8 356 (7 557 to 9 126)	1 439.1 (1 351.1 to 1 533.1)	864.1 (780.9 to 943.2)
Pedestrian road injuries	72 (51 to 97)	134 (95 to 184)	19.0 (13.5 to 25.6)	15.0 (10.6 to 20.6)	3 593 (3 216 to 4 024)	3 027 (2 719 to 3 330)	669.6 (615.7 to 729.7)	318.9 (287.1 to 350.4)
Cyclist road injuries	39 (28 to 51)	93 (68 to 131)	9.5 (6.7 to 12.4)	10.7 (7.5 to 14.3)	305 (271 to 339)	504 (440 to 568)	58.8 (52.7 to 64.9)	53.0 (46.4 to 59.6)
Motorcyclist road injuries	61 (43 to 82)	174 (120 to 237)	15.5 (10.8 to 20.9)	18.7 (12.9 to 25.5)	1 354 (1 210 to 1 507)	1 564 (1 373 to 1 777)	245.1 (221.7 to 269.5)	157.7 (138.4 to 179.3)
Motor vehicle road injuries	75 (54 to 99)	298 (216 to 399)	19.9 (14.4 to 26.3)	32.7 (23.6 to 44.0)	2 287 (2 082 to 2 511)	2 991 (2 621 to 3 335)	419.2 (385.1 to 455.7)	306.1 (268.3 to 340.9)
Other road injuries	30 (22 to 41)	70 (50 to 97)	7.8 (5.6 to 10.4)	7.8 (5.6 to 10.7)	248 (211 to 287)	269 (232 to 311)	46.5 (40.2 to 52.8)	28.3 (24.4 to 32.6)
Other transport injuries	46 (33 to 62)	43 (31 to 58)	11.0 (7.9 to 14.6)	4.8 (3.5 to 6.5)	682 (615 to 755)	959 (829 to 1 098)	127.4 (116.1 to 139.4)	99.0 (85.7 to 112.7)
**Unintentional injuries**	**1 409 (1 023 to 1 873)**	**2 802 (1 995 to 3 798)**	**354.8 (256.4 to 471.7)**	**318.3 (226.4 to 431.8)**	**11 039 (9 896 to 12 400)**	**9 371 (8 456 to 10 492)**	**2 027.7 (1 859.3 to 2 216.8)**	**1 028.0 (925.6 to 1 153.6)**
Falls	810 (596 to 1 067)	1 875 (1 353 to 2 497)	214.4 (156.5 to 279.4)	216.7 (155.3 to 289.4)	1 893 (1 650 to 2 163)	3 105 (2 572 to 3 729)	469.9 (411.2 to 537.1)	358.5 (295.8 to 429.9)
Drowning	10 (7 to 14)	10 (7 to 14)	2.7 (1.9 to 3.7)	1.1 (0.7 to 1.5)	3 104 (2 719 to 3 580)	1 723 (1 556 to 1 902)	508.0 (456.8 to 567.9)	179.9 (162.6 to 198.3)
Fire, heat, and hot substances	142 (88 to 225)	171 (101 to 276)	30.3 (18.9 to 48.0)	18.4 (10.9 to 29.7)	778 (654 to 932)	468 (392 to 571)	137.4 (119.1 to 160.8)	51.3 (43.0 to 62.3)
Poisonings	26 (16 to 38)	45 (27 to 66)	4.9 (3.1 to 7.3)	4.7 (2.8 to 7.0)	510 (440 to 595)	305 (275 to 341)	82.0 (72.8 to 93.0)	32.1 (28.9 to 35.8)
Poisoning by carbon monoxide	4 (2 to 6)	6 (3 to 9)	0.7 (0.5 to 1.1)	0.6 (0.4 to 0.9)	114 (97 to 135)	81 (71 to 93)	19.1 (16.7 to 22.0)	8.5 (7.4 to 9.7)
Poisoning by other means	22 (14 to 33)	39 (22 to 59)	4.2 (2.6 to 6.3)	4.1 (2.4 to 6.2)	396 (339 to 469)	224 (200 to 253)	62.8 (55.0 to 72.3)	23.7 (21.1 to 26.7)
Exposure to mechanical forces	227 (158 to 324)	456 (311 to 667)	57.5 (39.9 to 82.1)	50.0 (34.1 to 73.2)	1 304 (1 180 to 1 445)	1 083 (922 to 1 288)	255.1 (231.0 to 282.8)	115.1 (98.0 to 137.5)
Unintentional firearm injuries	19 (12 to 28)	24 (15 to 38)	4.7 (3.1 to 7.4)	2.6 (1.7 to 4.2)	440 (387 to 516)	213 (177 to 245)	80.3 (71.5 to 94.6)	21.9 (18.2 to 25.2)
Other exposure to mechanical forces	209 (145 to 298)	432 (293 to 632)	52.8 (36.4 to 75.3)	47.4 (32.1 to 69.6)	864 (754 to 984)	870 (722 to 1 061)	174.8 (152.5 to 200.0)	93.2 (77.0 to 114.5)
Adverse effects of medical treatment	4 (3 to 6)	9 (5 to 13)	1.0 (0.6 to 1.5)	1.0 (0.6 to 1.5)	555 (356 to 675)	555 (468 to 630)	101.7 (68.5 to 117.8)	62.9 (52.9 to 71.3)
Animal contact	44 (29 to 61)	55 (36 to 78)	9.7 (6.5 to 13.7)	6.0 (3.9 to 8.6)	456 (370 to 570)	193 (168 to 221)	72.0 (60.8 to 85.9)	21.2 (18.5 to 24.2)
Venomous animal contact	24 (15 to 35)	26 (17 to 39)	4.8 (3.1 to 7.0)	2.8 (1.8 to 4.1)	380 (300 to 490)	128 (110 to 146)	57.6 (47.1 to 71.3)	14.0 (12.0 to 16.0)
Non-venomous animal contact	19 (12 to 31)	28 (17 to 46)	4.8 (3.0 to 7.7)	3.2 (1.9 to 5.1)	76 (57 to 96)	65 (52 to 85)	14.5 (10.9 to 18.5)	7.2 (5.7 to 9.5)
Foreign body	42 (28 to 60)	68 (42 to 106)	9.6 (6.5 to 13.5)	7.6 (4.8 to 11.8)	1 567 (1 333 to 1 865)	1 276 (1 116 to 1 471)	238.6 (208.9 to 276.2)	138.1 (120.6 to 159.8)
Pulmonary aspiration and foreign body in airway	2 (1 to 3)	1 (1 to 2)	0.4 (0.3 to 0.5)	0.1 (0.1 to 0.2)	1 471 (1 245 to 1 755)	1 180 (1 027 to 1 379)	220.4 (191.8 to 256.6)	127.6 (110.8 to 148.8)
Foreign body in eyes	14 (7 to 26)	46 (25 to 81)	3.8 (2.1 to 6.5)	5.2 (2.9 to 9.1)	14 (7 to 26)	46 (25 to 81)	3.8 (2.1 to 6.5)	5.2 (2.9 to 9.1)
Foreign body in other body part	25 (18 to 34)	21 (15 to 27)	5.3 (3.8 to 7.0)	2.3 (1.6 to 3.0)	82 (68 to 97)	50 (40 to 75)	14.3 (11.9 to 16.8)	5.3 (4.3 to 7.3)
Environmental heat and cold exposure	36 (24 to 51)	30 (20 to 44)	8.7 (5.8 to 12.2)	3.4 (2.2 to 5.0)	185 (164 to 211)	109 (95 to 125)	38.5 (34.8 to 43.3)	11.9 (10.4 to 13.6)
Exposure to forces of nature	8 (5 to 11)	11 (8 to 15)	1.4 (1.0 to 2.0)	1.2 (0.9 to 1.6)	25 (22 to 28)	19 (16 to 23)	4.3 (3.8 to 4.9)	2.0 (1.7 to 2.4)
Other unintentional injuries	59 (42 to 80)	74 (52 to 101)	14.7 (10.4 to 19.8)	8.2 (5.8 to 11.3)	663 (599 to 729)	534 (475 to 602)	120.2 (109.6 to 131.6)	55.1 (49.0 to 61.9)
