# Supplementary material for: Unintentional injuries in Mexico, 1990–2017: findings from the Global Burden of Disease Study 2017
Source: Inj Prev. 2020 Apr 1;26(Suppl 1):i154–61. doi: 10.1136/injuryprev-2019-043532 (PMC7571365; doi:10.1136/injuryprev-2019-043532)
Supplement: Supplementary data [file injuryprev-2019-043532supp009.pdf]

Death, YLL, incidence, prevalence, YLD, and DALY counts and rates per 100,000 for all injuries in Chiapas in 1990 and 2017

Cause	Deaths				Years of life lost			
	All-age counts (95% UI)		Age-standardised rates per 100,000 (95% UI)		All-age counts (95% UI)		Age-standardised rates per 100,000 (95% UI)	
	1990	2017	1990	2017	1990	2017	1990	2017
**Transport and unintentional injuries**	**1 663 (1 583 to 1 752)**	**1 849 (1 726 to 1 979)**	**63.0 (60.7 to 65.2)**	**36.3 (33.9 to 38.7)**	**97 344 (91 241 to 104 387)**	**90 003 (83 302 to 97 195)**	**2 890.8 (2 754.7 to 3 043.0)**	**1 549.5 (1 440.0 to 1 667.8)**
**Transport injuries**	**559 (525 to 591)**	**801 (728 to 875)**	**20.1 (19.0 to 21.1)**	**14.8 (13.4 to 16.1)**	**32 677 (30 360 to 34 786)**	**41 426 (37 555 to 45 379)**	**994.8 (936.9 to 1 050.9)**	**708.5 (643.2 to 775.7)**
Road injuries	537 (504 to 568)	766 (696 to 837)	19.3 (18.3 to 20.3)	14.1 (12.8 to 15.4)	31 353 (29 131 to 33 414)	39 619 (35 913 to 43 478)	956.2 (899.6 to 1 010.6)	677.4 (614.6 to 742.0)
Pedestrian road injuries	228 (210 to 245)	311 (281 to 339)	9.0 (8.4 to 9.6)	6.1 (5.5 to 6.6)	12 892 (11 794 to 14 117)	14 627 (13 155 to 16 073)	401.1 (368.6 to 432.1)	258.1 (232.1 to 283.5)
Cyclist road injuries	12 (11 to 14)	25 (22 to 28)	0.4 (0.4 to 0.5)	0.5 (0.4 to 0.5)	730 (643 to 824)	1 223 (1 060 to 1 390)	21.9 (19.6 to 24.8)	21.3 (18.6 to 24.1)
Motorcyclist road injuries	91 (81 to 101)	133 (115 to 153)	2.9 (2.6 to 3.3)	2.2 (1.9 to 2.6)	5 469 (4 863 to 6 133)	7 714 (6 601 to 8 964)	163.3 (145.8 to 181.5)	126.4 (109.0 to 146.0)
Motor vehicle road injuries	194 (175 to 219)	284 (253 to 318)	6.5 (5.9 to 7.4)	5.1 (4.5 to 5.7)	11 546 (10 336 to 12 961)	15 357 (13 590 to 17 208)	349.1 (314.8 to 393.3)	259.7 (230.3 to 289.5)
Other road injuries	12 (10 to 14)	13 (12 to 16)	0.4 (0.3 to 0.5)	0.2 (0.2 to 0.3)	716 (585 to 838)	698 (590 to 835)	20.8 (17.3 to 23.9)	11.9 (10.2 to 14.1)
Other transport injuries	22 (20 to 24)	35 (31 to 40)	0.8 (0.7 to 0.8)	0.7 (0.6 to 0.7)	1 324 (1 183 to 1 478)	1 806 (1 588 to 2 059)	38.6 (35.0 to 42.3)	31.1 (27.4 to 35.4)
**Unintentional injuries**	**1 104 (1 038 to 1 173)**	**1 048 (987 to 1 115)**	**42.9 (41.1 to 44.8)**	**21.5 (20.3 to 22.8)**	**64 667 (59 846 to 70 012)**	**48 577 (45 316 to 52 287)**	**1 896.0 (1 785.4 to 2 011.2)**	**841.0 (787.2 to 899.3)**
Falls	159 (150 to 169)	258 (239 to 277)	9.7 (9.2 to 10.4)	6.4 (5.9 to 6.8)	6 489 (5 997 to 7 018)	7 769 (7 105 to 8 441)	259.2 (244.3 to 277.7)	155.0 (142.5 to 167.9)
Drowning	243 (225 to 263)	188 (173 to 205)	7.7 (7.2 to 8.2)	3.3 (3.1 to 3.6)	15 577 (14 175 to 17 135)	10 646 (9 611 to 11 752)	419.7 (388.2 to 453.5)	175.1 (158.5 to 192.5)
Fire, heat, and hot substances	66 (59 to 75)	45 (41 to 48)	2.5 (2.3 to 2.7)	1.0 (0.9 to 1.1)	4 186 (3 583 to 4 891)	1 828 (1 645 to 2 018)	108.9 (96.9 to 128.4)	32.6 (29.6 to 35.7)
Poisonings	70 (64 to 76)	50 (45 to 55)	2.2 (2.1 to 2.4)	0.9 (0.8 to 1.0)	4 542 (4 090 to 5 014)	2 682 (2 402 to 2 993)	119.7 (110.0 to 130.0)	44.5 (40.3 to 49.4)
Poisoning by carbon monoxide	10 (9 to 12)	8 (7 to 9)	0.3 (0.3 to 0.3)	0.1 (0.1 to 0.2)	689 (600 to 799)	427 (368 to 487)	17.8 (15.8 to 20.1)	7.1 (6.2 to 8.1)
Poisoning by other means	59 (54 to 65)	42 (38 to 46)	1.9 (1.8 to 2.0)	0.8 (0.7 to 0.8)	3 853 (3 469 to 4 272)	2 255 (2 014 to 2 520)	102.0 (93.4 to 111.1)	37.4 (33.6 to 41.5)
Exposure to mechanical forces	265 (243 to 283)	132 (121 to 144)	9.8 (8.9 to 10.5)	2.6 (2.4 to 2.8)	15 076 (13 689 to 16 188)	6 352 (5 726 to 6 976)	476.6 (432.6 to 510.4)	110.8 (100.2 to 121.2)
Unintentional firearm injuries	141 (126 to 154)	47 (39 to 53)	4.9 (4.4 to 5.4)	0.9 (0.7 to 1.0)	8 191 (7 348 to 8 983)	2 354 (1 948 to 2 694)	253.9 (225.9 to 278.5)	40.5 (33.6 to 46.3)
Other exposure to mechanical forces	124 (96 to 138)	86 (77 to 95)	4.9 (3.7 to 5.5)	1.7 (1.6 to 1.9)	6 884 (5 142 to 7 713)	3 998 (3 576 to 4 505)	222.7 (169.5 to 248.6)	70.2 (63.0 to 78.6)
Adverse effects of medical treatment	55 (42 to 62)	71 (63 to 88)	2.4 (2.0 to 2.6)	1.6 (1.4 to 2.0)	3 209 (2 348 to 3 770)	2 791 (2 415 to 3 568)	88.9 (69.6 to 100.4)	49.9 (44.0 to 63.0)
Animal contact	21 (18 to 24)	12 (11 to 14)	0.6 (0.6 to 0.7)	0.2 (0.2 to 0.3)	1 446 (1 222 to 1 733)	600 (522 to 684)	34.3 (29.9 to 39.9)	10.1 (9.0 to 11.4)
Venomous animal contact	15 (12 to 18)	7 (6 to 9)	0.4 (0.4 to 0.5)	0.1 (0.1 to 0.2)	1 058 (865 to 1 301)	368 (280 to 437)	24.3 (20.5 to 28.9)	6.2 (4.7 to 7.2)
Non-venomous animal contact	6 (5 to 7)	5 (4 to 7)	0.2 (0.2 to 0.2)	0.1 (0.1 to 0.1)	387 (319 to 463)	231 (194 to 321)	10.1 (8.6 to 11.8)	4.0 (3.4 to 5.5)
Foreign body	138 (126 to 153)	143 (129 to 159)	4.4 (4.1 to 4.7)	2.6 (2.4 to 2.9)	9 231 (8 195 to 10 451)	8 240 (7 234 to 9 506)	233.5 (213.4 to 256.1)	132.5 (117.4 to 150.3)
Pulmonary aspiration and foreign body in airway	129 (117 to 142)	138 (125 to 154)	4.2 (3.9 to 4.5)	2.6 (2.3 to 2.8)	8 598 (7 629 to 9 702)	7 944 (6 941 to 9 186)	218.0 (199.0 to 239.3)	127.6 (113.2 to 145.3)
Foreign body in eyes								
Foreign body in other body part	9 (6 to 11)	5 (4 to 7)	0.2 (0.2 to 0.3)	0.1 (0.1 to 0.1)	633 (408 to 764)	296 (247 to 421)	15.5 (10.8 to 18.2)	4.8 (4.1 to 6.9)
Environmental heat and cold exposure	41 (39 to 43)	13 (12 to 15)	2.0 (1.9 to 2.1)	0.3 (0.3 to 0.3)	2 010 (1 869 to 2 178)	548 (492 to 603)	73.0 (69.6 to 76.6)	10.2 (9.2 to 11.3)
Exposure to forces of nature	2 (2 to 2)	99 (91 to 107)	0.1 (0.1 to 0.1)	1.9 (1.7 to 2.0)	105 (94 to 116)	5 106 (4 663 to 5 616)	3.0 (2.8 to 3.3)	86.5 (79.2 to 94.5)
Other unintentional injuries	45 (42 to 49)	37 (33 to 42)	1.5 (1.4 to 1.6)	0.7 (0.6 to 0.7)	2 802 (2 548 to 3 078)	2 015 (1 799 to 2 284)	79.1 (72.5 to 85.9)	33.8 (30.3 to 38.2)

Cause	Incidence				Prevalence			
	All-age counts (95% UI)		Age-standardised rates per 100,000 (95% UI)		All-age counts (95% UI)		Age-standardised rates per 100,000 (95% UI)	
	1990	2017	1990	2017	1990	2017	1990	2017
**Transport and unintentional injuries**	**114 498 (103 981 to 126 608)**	**208 639 (189 154 to 229 318)**	**4 073.9 (3 683.2 to 4 489.4)**	**3 899.3 (3 536.1 to 4 300.4)**	**215 578 (202 070 to 230 894)**	**409 182 (384 343 to 440 637)**	**9 908.1 (9 321.6 to 10 641.5)**	**8 374.7 (7 880.6 to 9 018.0)**
**Transport injuries**	**8 335 (7 030 to 9 750)**	**26 054 (22 454 to 30 218)**	**290.3 (251.9 to 332.2)**	**463.5 (401.0 to 532.3)**	**18 971 (17 713 to 20 196)**	**67 922 (63 292 to 72 822)**	**930.3 (869.3 to 992.9)**	**1 393.2 (1 295.5 to 1 498.5)**
Road injuries	7 916 (6 660 to 9 353)	25 654 (22 039 to 29 840)	277.3 (239.5 to 319.0)	455.9 (393.5 to 525.1)	17 194 (15 988 to 18 345)	66 038 (61 379 to 70 875)	854.0 (792.8 to 914.8)	1 354.4 (1 259.6 to 1 458.9)
Pedestrian road injuries	1 282 (1 029 to 1 595)	3 923 (3 152 to 4 768)	47.8 (39.2 to 57.2)	72.0 (58.3 to 86.9)	4 446 (3 800 to 5 179)	16 224 (14 023 to 18 972)	225.3 (195.6 to 261.4)	338.4 (293.7 to 392.5)
Cyclist road injuries	1 122 (829 to 1 494)	3 090 (2 384 to 3 968)	35.1 (27.4 to 44.7)	53.2 (41.3 to 67.2)	1 755 (1 475 to 2 092)	5 997 (5 022 to 7 116)	79.5 (67.9 to 92.7)	118.9 (100.3 to 139.5)
Motorcyclist road injuries	1 077 (837 to 1 376)	2 886 (2 198 to 3 753)	35.6 (28.3 to 44.3)	49.1 (37.7 to 63.0)	3 628 (3 126 to 4 205)	11 809 (10 045 to 13 856)	175.4 (151.3 to 203.7)	236.8 (201.7 to 278.0)
Motor vehicle road injuries	3 062 (2 477 to 3 689)	12 718 (10 629 to 15 243)	110.9 (91.0 to 130.6)	226.3 (189.3 to 268.9)	5 015 (4 399 to 5 843)	25 625 (22 971 to 29 965)	258.6 (225.5 to 301.1)	528.5 (460.6 to 617.5)
Other road injuries	1 373 (991 to 1 800)	3 037 (2 334 to 3 846)	48.6 (37.8 to 60.5)	55.4 (43.5 to 68.5)	2 351 (1 952 to 2 848)	6 389 (5 361 to 7 681)	115.2 (96.0 to 137.3)	131.3 (110.7 to 157.7)
Other transport injuries	419 (309 to 572)	401 (308 to 523)	13.0 (10.3 to 16.2)	7.5 (5.9 to 9.6)	1 777 (1 467 to 2 114)	1 884 (1 616 to 2 163)	76.3 (64.6 to 88.5)	38.8 (33.7 to 44.3)
**Unintentional injuries**	**106 162 (96 019 to 117 474)**	**182 585 (164 346 to 202 944)**	**3 783.7 (3 391.5 to 4 201.0)**	**3 435.8 (3 084.6 to 3 836.6)**	**196 607 (183 599 to 211 771)**	**341 260 (317 286 to 372 825)**	**8 977.8 (8 396.3 to 9 685.3)**	**6 981.5 (6 518.2 to 7 603.2)**
Falls	38 135 (33 081 to 44 135)	86 918 (76 287 to 98 821)	1 521.1 (1 345.3 to 1 712.1)	1 711.5 (1 510.0 to 1 939.0)	62 689 (55 139 to 71 365)	162 516 (144 708 to 185 851)	3 146.7 (2 813.5 to 3 601.5)	3 494.3 (3 071.2 to 3 921.5)
Drowning	202 (173 to 237)	149 (119 to 185)	6.4 (5.5 to 7.4)	2.7 (2.2 to 3.3)	839 (731 to 975)	822 (719 to 948)	41.7 (36.9 to 47.7)	17.4 (15.3 to 20.1)
Fire, heat, and hot substances	4 167 (3 249 to 5 299)	4 261 (3 396 to 5 311)	101.4 (82.0 to 123.7)	68.8 (55.8 to 84.4)	35 849 (30 511 to 46 814)	44 724 (36 970 to 57 093)	1 437.7 (1 193.8 to 1 724.8)	847.3 (708.1 to 1 060.5)
Poisonings	2 050 (1 574 to 2 609)	3 211 (2 471 to 4 054)	45.9 (36.4 to 56.9)	49.6 (38.6 to 61.9)	1 564 (1 237 to 1 898)	3 029 (2 300 to 3 729)	52.6 (42.4 to 63.4)	53.8 (41.2 to 66.3)
Poisoning by carbon monoxide	341 (240 to 481)	305 (191 to 452)	8.9 (6.7 to 11.8)	5.3 (3.5 to 7.4)	202 (159 to 259)	188 (149 to 237)	7.2 (5.8 to 9.1)	3.5 (2.8 to 4.4)
Poisoning by other means	1 709 (1 291 to 2 208)	2 906 (2 220 to 3 709)	37.1 (28.8 to 46.7)	44.3 (34.3 to 55.8)	1 361 (1 020 to 1 680)	2 834 (2 105 to 3 534)	45.4 (34.6 to 55.9)	50.3 (37.6 to 62.6)
Exposure to mechanical forces	34 227 (29 268 to 40 628)	36 751 (31 595 to 42 553)	1 108.0 (953.9 to 1 290.4)	640.5 (553.3 to 739.1)	61 532 (53 955 to 70 592)	81 415 (71 116 to 93 795)	2 976.7 (2 626.7 to 3 402.5)	1 647.6 (1 448.8 to 1 899.5)
Unintentional firearm injuries	2 972 (2 225 to 3 910)	1 623 (1 199 to 2 129)	95.9 (72.6 to 123.7)	28.7 (21.5 to 37.2)	9 694 (7 764 to 11 748)	7 409 (6 009 to 8 970)	483.1 (392.0 to 591.7)	153.8 (125.1 to 186.8)
Other exposure to mechanical forces	31 255 (26 503 to 37 164)	35 127 (30 288 to 40 720)	1 012.1 (873.9 to 1 183.2)	611.7 (529.7 to 706.5)	51 838 (43 865 to 61 163)	74 006 (63 555 to 87 199)	2 493.6 (2 126.3 to 2 952.8)	1 493.9 (1 289.8 to 1 751.3)
Adverse effects of medical treatment	2 026 (1 691 to 2 380)	4 087 (3 445 to 4 876)	81.4 (69.5 to 94.7)	79.8 (67.7 to 95.2)	155 (120 to 195)	319 (239 to 396)	6.2 (4.8 to 7.7)	6.1 (4.7 to 7.7)
Animal contact	7 781 (6 318 to 9 777)	10 161 (8 720 to 12 021)	246.0 (206.0 to 291.1)	183.2 (156.4 to 211.1)	7 547 (6 449 to 8 829)	11 707 (10 111 to 13 500)	334.9 (292.1 to 382.7)	235.4 (204.5 to 269.7)
Venomous animal contact	2 692 (1 978 to 3 602)	3 061 (2 365 to 3 877)	84.8 (64.8 to 108.7)	54.5 (42.6 to 68.1)	1 605 (1 321 to 1 918)	1 848 (1 564 to 2 156)	64.9 (54.5 to 75.5)	35.6 (30.4 to 41.3)
Non-venomous animal contact	5 089 (3 992 to 6 499)	7 101 (5 946 to 8 586)	161.2 (131.5 to 196.4)	128.6 (107.0 to 151.7)	5 942 (4 893 to 7 176)	9 859 (8 296 to 11 619)	270.0 (226.6 to 318.5)	199.8 (170.2 to 233.2)
Foreign body	11 904 (5 516 to 18 352)	22 248 (10 115 to 37 342)	468.9 (195.3 to 782.8)	442.1 (187.0 to 764.3)	4 426 (3 271 to 6 076)	6 540 (4 421 to 9 744)	199.7 (151.4 to 264.5)	137.6 (94.6 to 199.8)
Pulmonary aspiration and foreign body in airway	261 (183 to 366)	143 (104 to 197)	6.3 (4.8 to 8.5)	2.5 (1.9 to 3.3)	157 (116 to 209)	104 (85 to 130)	5.6 (4.3 to 7.2)	2.0 (1.7 to 2.5)
Foreign body in eyes	10 229 (4 488 to 17 034)	21 534 (9 390 to 36 697)	440.0 (163.7 to 752.2)	429.5 (174.5 to 751.8)	1 954 (939 to 3 450)	4 570 (2 469 to 7 636)	101.1 (57.2 to 161.4)	99.0 (56.3 to 161.6)
Foreign body in other body part	814 (531 to 1 281)	571 (409 to 777)	22.6 (15.6 to 32.4)	10.2 (7.2 to 13.3)	2 315 (1 851 to 2 927)	1 866 (1 541 to 2 273)	93.0 (76.9 to 114.5)	36.6 (30.6 to 44.1)
Environmental heat and cold exposure	1 298 (978 to 1 676)	1 267 (1 040 to 1 500)	44.6 (36.5 to 53.5)	23.6 (19.7 to 27.7)	4 165 (3 377 to 5 059)	4 500 (3 833 to 5 282)	183.9 (155.3 to 216.5)	91.5 (79.4 to 106.4)
Exposure to forces of nature	273 (224 to 342)	6 793 (5 732 to 8 154)	7.7 (6.5 to 9.1)	114.6 (97.9 to 135.6)	10 582 (7 386 to 14 908)	14 879 (10 500 to 20 459)	330.3 (237.8 to 457.1)	291.2 (207.5 to 398.6)
Other unintentional injuries	4 699 (3 875 to 5 848)	6 742 (5 702 to 7 988)	152.2 (128.5 to 180.3)	121.6 (103.0 to 142.6)	6 258 (5 376 to 7 236)	10 821 (9 400 to 12 389)	285.3 (247.4 to 325.3)	219.2 (190.7 to 248.7)

Cause	Years lived with disability				Disability adjusted life years			
	All-age counts (95% UI)		Age-standardised rates per 100,000 (95% UI)		All-age counts (95% UI)		Age-standardised rates per 100,000 (95% UI)	
	1990	2017	1990	2017	1990	2017	1990	2017
**Transport and unintentional injuries**	**9 673 (7 168 to 12 640)**	**18 402 (13 516 to 24 405)**	**431.1 (318.2 to 563.4)**	**374.0 (274.8 to 496.3)**	**107 017 (100 184 to 115 018)**	**108 404 (100 241 to 117 389)**	**3 321.9 (3 143.5 to 3 524.3)**	**1 923.5 (1 774.6 to 2 086.1)**
**Transport injuries**	**1 387 (1 013 to 1 819)**	**4 380 (3 186 to 5 834)**	**64.7 (47.2 to 84.6)**	**88.0 (63.9 to 117.0)**	**34 064 (31 699 to 36 283)**	**45 806 (41 888 to 50 176)**	**1 059.5 (998.8 to 1 119.6)**	**796.5 (727.7 to 870.9)**
Road injuries	1 262 (924 to 1 663)	4 271 (3 100 to 5 694)	59.7 (43.6 to 78.2)	85.8 (62.2 to 114.3)	32 635 (30 370 to 34 739)	43 891 (40 117 to 48 084)	1 015.8 (957.0 to 1 074.5)	763.3 (697.6 to 834.4)
Pedestrian road injuries	337 (239 to 456)	1 084 (767 to 1 467)	16.2 (11.6 to 22.0)	22.1 (15.7 to 30.1)	13 229 (12 068 to 14 483)	15 711 (14 212 to 17 312)	417.3 (385.5 to 446.5)	280.3 (254.0 to 307.8)
Cyclist road injuries	132 (94 to 174)	385 (271 to 518)	5.8 (4.1 to 7.5)	7.5 (5.3 to 10.0)	862 (766 to 959)	1 608 (1 402 to 1 824)	27.7 (24.9 to 30.7)	28.8 (25.2 to 32.8)
Motorcyclist road injuries	269 (188 to 360)	761 (520 to 1 038)	12.4 (8.7 to 16.8)	15.0 (10.3 to 20.4)	5 737 (5 141 to 6 381)	8 475 (7 342 to 9 742)	175.7 (158.1 to 193.7)	141.4 (123.0 to 161.5)
Motor vehicle road injuries	383 (276 to 507)	1 709 (1 247 to 2 260)	18.7 (13.6 to 24.5)	34.5 (25.2 to 45.5)	11 929 (10 718 to 13 344)	17 067 (15 158 to 19 007)	367.8 (332.7 to 412.2)	294.2 (262.4 to 326.7)
Other road injuries	141 (101 to 192)	332 (239 to 453)	6.6 (4.8 to 8.9)	6.7 (4.8 to 9.2)	858 (726 to 983)	1 030 (888 to 1 208)	27.4 (23.5 to 31.1)	18.6 (16.0 to 21.9)
Other transport injuries	124 (88 to 166)	108 (78 to 145)	5.0 (3.6 to 6.6)	2.2 (1.6 to 2.9)	1 449 (1 311 to 1 612)	1 915 (1 691 to 2 171)	43.6 (39.9 to 47.6)	33.3 (29.5 to 37.5)
**Unintentional injuries**	**8 287 (6 139 to 10 828)**	**14 022 (10 200 to 18 660)**	**366.4 (269.1 to 479.0)**	**286.0 (207.7 to 381.3)**	**72 953 (67 262 to 79 197)**	**62 599 (57 714 to 68 416)**	**2 262.4 (2 110.5 to 2 424.5)**	**1 127.0 (1 034.7 to 1 237.0)**
Falls	4 045 (2 950 to 5 275)	8 942 (6 491 to 11 781)	193.9 (140.3 to 251.2)	186.6 (135.5 to 244.2)	10 534 (9 292 to 11 876)	16 711 (14 241 to 19 587)	453.1 (397.6 to 515.2)	341.6 (288.9 to 401.3)
Drowning	50 (34 to 68)	45 (31 to 61)	2.4 (1.7 to 3.2)	0.9 (0.6 to 1.3)	15 626 (14 219 to 17 179)	10 691 (9 646 to 11 798)	422.1 (390.9 to 455.9)	176.0 (159.3 to 193.3)
Fire, heat, and hot substances	847 (540 to 1 299)	914 (552 to 1 486)	31.1 (19.6 to 47.4)	17.1 (10.3 to 27.0)	5 033 (4 351 to 5 858)	2 742 (2 340 to 3 314)	140.0 (123.0 to 161.9)	49.6 (42.9 to 60.1)
Poisonings	183 (114 to 270)	343 (216 to 521)	5.9 (3.7 to 8.6)	6.0 (3.8 to 9.0)	4 725 (4 288 to 5 246)	3 025 (2 719 to 3 389)	125.6 (116.2 to 136.5)	50.5 (45.6 to 56.3)
Poisoning by carbon monoxide	21 (13 to 33)	18 (11 to 28)	0.7 (0.4 to 1.1)	0.3 (0.2 to 0.5)	710 (621 to 820)	444 (386 to 507)	18.5 (16.5 to 20.9)	7.4 (6.5 to 8.4)
Poisoning by other means	162 (100 to 241)	325 (200 to 498)	5.2 (3.2 to 7.7)	5.7 (3.5 to 8.6)	4 015 (3 637 to 4 466)	2 581 (2 312 to 2 891)	107.1 (98.8 to 117.0)	43.1 (38.8 to 48.0)
Exposure to mechanical forces	1 521 (1 059 to 2 165)	1 844 (1 260 to 2 666)	71.4 (49.4 to 101.4)	37.0 (25.2 to 53.6)	16 597 (15 112 to 17 919)	8 196 (7 355 to 9 241)	548.0 (499.5 to 593.2)	147.7 (132.2 to 167.8)
Unintentional firearm injuries	212 (142 to 319)	140 (91 to 221)	10.1 (6.7 to 15.5)	2.9 (1.9 to 4.5)	8 403 (7 550 to 9 208)	2 494 (2 072 to 2 857)	264.0 (236.2 to 289.3)	43.4 (36.1 to 49.4)
Other exposure to mechanical forces	1 309 (909 to 1 860)	1 703 (1 161 to 2 466)	61.3 (42.4 to 87.9)	34.1 (23.2 to 49.5)	8 193 (6 784 to 9 210)	5 702 (4 989 to 6 651)	283.9 (229.6 to 321.4)	104.3 (91.2 to 121.9)
Adverse effects of medical treatment	21 (15 to 31)	42 (26 to 64)	0.8 (0.5 to 1.2)	0.8 (0.5 to 1.2)	3 224 (2 978 to 3 791)	2 833 (2 459 to 3 611)	89.8 (70.4 to 101.2)	50.7 (44.8 to 63.7)
Animal contact	207 (138 to 292)	270 (182 to 386)	8.1 (5.4 to 11.3)	5.2 (3.5 to 7.4)	1 652 (1 415 to 1 942)	869 (743 to 1 024)	42.4 (37.3 to 48.3)	15.3 (13.2 to 18.0)
Venomous animal contact	110 (70 to 163)	122 (79 to 176)	3.8 (2.5 to 5.5)	2.2 (1.4 to 3.2)	1 169 (969 to 1 409)	491 (405 to 580)	28.1 (23.9 to 32.8)	8.4 (6.9 to 9.9)
Non-venomous animal contact	96 (59 to 150)	147 (89 to 233)	4.3 (2.7 to 6.6)	3.0 (1.8 to 4.7)	483 (400 to 577)	378 (304 to 486)	14.3 (11.9 to 17.2)	6.9 (5.5 to 8.9)
Foreign body	297 (196 to 442)	410 (242 to 677)	13.0 (8.5 to 19.3)	8.6 (5.1 to 14.2)	9 529 (8 498 to 10 706)	8 651 (7 597 to 9 914)	246.5 (225.8 to 270.0)	141.1 (125.5 to 159.4)
Pulmonary aspiration and foreign body in airway	9 (6 to 13)	5 (3 to 7)	0.3 (0.2 to 0.4)	0.1 (0.1 to 0.1)	8 608 (7 639 to 9 714)	7 949 (6 947 to 9 189)	218.3 (199.3 to 239.7)	127.7 (113.3 to 145.4)
Foreign body in eyes	130 (62 to 244)	305 (158 to 552)	6.8 (3.6 to 12.1)	6.6 (3.5 to 12.0)	130 (62 to 244)	305 (158 to 552)	6.8 (3.6 to 12.1)	6.6 (3.5 to 12.0)
Foreign body in other body part	158 (112 to 212)	101 (72 to 132)	5.9 (4.3 to 7.8)	1.9 (1.4 to 2.5)	791 (574 to 930)	397 (340 to 520)	21.4 (16.7 to 24.7)	6.8 (5.8 to 8.7)
Environmental heat and cold exposure	172 (116 to 239)	174 (116 to 246)	7.5 (5.2 to 10.5)	3.5 (2.4 to 5.1)	2 183 (2 026 to 2 361)	723 (638 to 814)	80.5 (76.3 to 85.6)	13.8 (12.1 to 15.5)
Exposure to forces of nature	679 (457 to 1 000)	647 (460 to 901)	20.7 (14.6 to 29.4)	12.5 (8.9 to 17.1)	783 (559 to 1 104)	5 753 (5 233 to 6 301)	23.7 (17.5 to 32.4)	98.9 (90.2 to 108.0)
Other unintentional injuries	265 (193 to 350)	391 (280 to 528)	11.7 (8.5 to 15.4)	7.8 (5.6 to 10.6)	3 068 (2 798 to 3 348)	2 406 (2 153 to 2 721)	90.8 (83.4 to 98.5)	41.7 (37.8 to 47.0)
