# Supplementary material for: Unintentional injuries in Mexico, 1990–2017: findings from the Global Burden of Disease Study 2017
Source: Inj Prev. 2020 Apr 1;26(Suppl 1):i154–61. doi: 10.1136/injuryprev-2019-043532 (PMC7571365; doi:10.1136/injuryprev-2019-043532)
Supplement: Supplementary data [file injuryprev-2019-043532supp011.pdf]

Death, YLL, incidence, prevalence, YLD, and DALY counts and rates per 100,000 for all injuries in Coahuila in 1990 and 2017

Cause	Deaths: All-age counts (95% UI) 1990	Deaths: All-age counts (95% UI) 2017	Deaths: Age-standardised rates per 100,000 (95% UI) 1990	Deaths: Age-standardised rates per 100,000 (95% UI) 2017
**Transport and unintentional injuries**	**1 039 (996 to 1 086)**	**981 (920 to 1 046)**	**66.0 (63.9 to 68.1)**	**33.6 (31.6 to 35.8)**
**Transport injuries**	**458 (435 to 484)**	**469 (434 to 506)**	**26.1 (25.0 to 27.2)**	**15.1 (14.0 to 16.3)**
Road injuries	445 (422 to 470)	448 (414 to 484)	25.2 (24.1 to 26.4)	14.4 (13.4 to 15.6)
Pedestrian road injuries	186 (173 to 200)	176 (162 to 192)	11.7 (11.0 to 12.3)	5.9 (5.4 to 6.4)
Cyclist road injuries	12 (11 to 13)	20 (18 to 23)	0.7 (0.6 to 0.7)	0.7 (0.6 to 0.7)
Motorcyclist road injuries	72 (65 to 79)	63 (56 to 71)	3.6 (3.3 to 4.0)	1.9 (1.7 to 2.2)
Motor vehicle road injuries	166 (154 to 179)	180 (163 to 198)	8.7 (8.2 to 9.4)	5.7 (5.1 to 6.2)
Other road injuries	9 (7 to 10)	9 (8 to 10)	0.5 (0.4 to 0.6)	0.3 (0.2 to 0.3)
Other transport injuries	14 (12 to 15)	22 (19 to 25)	0.8 (0.8 to 0.9)	0.7 (0.6 to 0.8)
**Unintentional injuries**	**581 (552 to 611)**	**512 (479 to 548)**	**39.9 (38.5 to 41.3)**	**18.5 (17.3 to 19.7)**
Falls	123 (117 to 130)	154 (143 to 166)	12.6 (12.0 to 13.3)	6.2 (5.7 to 6.7)
Drowning	121 (111 to 132)	69 (63 to 76)	6.2 (5.8 to 6.6)	2.2 (2.0 to 2.5)
Fire, heat, and hot substances	48 (45 to 52)	36 (33 to 39)	3.6 (3.4 to 3.8)	1.3 (1.3 to 1.4)
Poisonings	31 (29 to 34)	22 (20 to 23)	1.7 (1.6 to 1.8)	0.7 (0.7 to 0.8)
Poisoning by carbon monoxide	15 (14 to 16)	12 (11 to 13)	0.8 (0.8 to 0.9)	0.4 (0.4 to 0.4)
Poisoning by other means	16 (15 to 18)	10 (9 to 11)	0.9 (0.8 to 0.9)	0.3 (0.3 to 0.4)
Exposure to mechanical forces	87 (82 to 93)	54 (49 to 59)	5.1 (4.8 to 5.4)	1.8 (1.6 to 1.9)
Unintentional firearm injuries	43 (39 to 51)	19 (17 to 22)	2.3 (2.1 to 2.8)	0.6 (0.5 to 0.7)
Other exposure to mechanical forces	44 (35 to 48)	35 (31 to 38)	2.8 (2.3 to 3.0)	1.2 (1.0 to 1.3)
Adverse effects of medical treatment	33 (28 to 37)	45 (40 to 53)	2.9 (2.4 to 3.1)	1.7 (1.5 to 2.0)
Animal contact	6 (5 to 7)	3 (3 to 4)	0.4 (0.3 to 0.4)	0.1 (0.1 to 0.1)
Venomous animal contact	4 (3 to 4)	1 (1 to 2)	0.2 (0.2 to 0.2)	0.1 (0.0 to 0.1)
Non-venomous animal contact	3 (2 to 3)	2 (2 to 2)	0.2 (0.1 to 0.2)	0.1 (0.1 to 0.1)
Foreign body	92 (82 to 102)	102 (91 to 117)	5.0 (4.6 to 5.4)	3.5 (3.1 to 4.0)
Pulmonary aspiration and foreign body in airway	87 (78 to 97)	100 (88 to 114)	4.8 (4.4 to 5.2)	3.4 (3.1 to 3.9)
Foreign body in eyes				
Foreign body in other body part	5 (3 to 6)	3 (2 to 4)	0.2 (0.2 to 0.3)	0.1 (0.1 to 0.1)
Environmental heat and cold exposure	14 (13 to 15)	8 (7 to 9)	1.1 (1.0 to 1.2)	0.3 (0.2 to 0.3)
Exposure to forces of nature	1 (1 to 1)	0 (0 to 1)	0.1 (0.0 to 0.1)	0.0 (0.0 to 0.0)
Other unintentional injuries	25 (23 to 27)	18 (16 to 20)	1.4 (1.3 to 1.5)	0.6 (0.5 to 0.6)

Cause	Years of life lost: All-age counts (95% UI) 1990	Years of life lost: All-age counts (95% UI) 2017	Years of life lost: Age-standardised rates per 100,000 (95% UI) 1990	Years of life lost: Age-standardised rates per 100,000 (95% UI) 2017
**Transport and unintentional injuries**	**55 290 (51 910 to 58 994)**	**42 864 (39 718 to 46 427)**	**2 705.7 (2 572.9 to 2 848.2)**	**1 371.3 (1 269.4 to 1 483.5)**
**Transport injuries**	**25 296 (23 703 to 27 007)**	**22 397 (20 642 to 24 407)**	**1 224.5 (1 157.7 to 1 297.6)**	**698.0 (641.8 to 760.2)**
Road injuries	24 576 (23 035 to 26 235)	21 397 (19 724 to 23 255)	1 188.4 (1 122.4 to 1 258.4)	666.9 (614.7 to 724.3)
Pedestrian road injuries	9 828 (8 873 to 10 889)	7 566 (6 880 to 8 385)	489.0 (451.3 to 532.1)	240.3 (219.0 to 265.3)
Cyclist road injuries	657 (588 to 739)	896 (787 to 1 019)	32.5 (29.5 to 36.0)	28.1 (24.7 to 31.9)
Motorcyclist road injuries	4 205 (3 771 to 4 648)	3 429 (2 992 to 3 888)	196.2 (177.0 to 215.5)	104.5 (91.2 to 118.2)
Motor vehicle road injuries	9 421 (8 630 to 10 297)	9 103 (8 197 to 10 098)	448.2 (413.6 to 481.5)	281.4 (253.8 to 312.2)
Other road injuries	466 (398 to 544)	403 (350 to 470)	22.4 (19.5 to 25.6)	12.6 (11.0 to 14.6)
Other transport injuries	720 (647 to 809)	1 000 (876 to 1 141)	36.1 (32.8 to 39.9)	31.1 (27.3 to 35.5)
**Unintentional injuries**	**29 994 (27 729 to 32 441)**	**20 467 (18 681 to 22 578)**	**1 481.2 (1 393.6 to 1 576.1)**	**673.3 (613.8 to 743.9)**
Falls	3 776 (3 564 to 4 011)	3 668 (3 386 to 3 963)	258.3 (245.9 to 271.0)	127.9 (118.0 to 137.5)
Drowning	7 398 (6 664 to 8 264)	3 528 (3 141 to 3 935)	328.1 (299.5 to 362.4)	111.9 (99.4 to 124.8)
Fire, heat, and hot substances	2 400 (2 128 to 2 780)	1 206 (1 098 to 1 321)	121.3 (110.5 to 133.7)	40.3 (36.7 to 44.1)
Poisonings	1 881 (1 682 to 2 114)	1 029 (932 to 1 146)	85.7 (78.0 to 94.9)	32.9 (29.8 to 36.6)
Poisoning by carbon monoxide	874 (779 to 991)	546 (487 to 609)	40.3 (36.5 to 44.8)	17.4 (15.5 to 19.3)
Poisoning by other means	1 006 (880 to 1 143)	483 (430 to 541)	45.3 (40.4 to 50.5)	15.5 (13.8 to 17.4)
Exposure to mechanical forces	4 680 (4 366 to 5 031)	2 366 (2 127 to 2 620)	229.7 (216.1 to 244.5)	74.2 (66.8 to 81.9)
Unintentional firearm injuries	2 405 (2 174 to 2 863)	918 (794 to 1 057)	113.4 (103.2 to 135.4)	28.4 (24.6 to 32.6)
Other exposure to mechanical forces	2 275 (1 799 to 2 526)	1 448 (1 277 to 1 637)	116.3 (92.4 to 127.3)	45.8 (40.6 to 51.5)
Adverse effects of medical treatment	1 432 (1 169 to 1 621)	1 415 (1 261 to 1 766)	79.0 (64.5 to 87.4)	48.2 (42.9 to 59.7)
Animal contact	393 (330 to 465)	143 (126 to 164)	17.5 (15.0 to 20.3)	4.8 (4.2 to 5.4)
Venomous animal contact	253 (200 to 314)	66 (48 to 84)	10.7 (8.7 to 13.1)	2.2 (1.6 to 2.8)
Non-venomous animal contact	140 (116 to 163)	77 (65 to 97)	6.8 (5.8 to 7.7)	2.5 (2.1 to 3.2)
Foreign body	6 006 (5 215 to 6 895)	5 905 (4 977 to 7 091)	259.8 (230.0 to 293.4)	195.7 (164.6 to 236.1)
Pulmonary aspiration and foreign body in airway	5 697 (4 936 to 6 545)	5 757 (4 839 to 6 927)	246.0 (217.2 to 278.5)	191.1 (160.1 to 230.5)
Foreign body in eyes				
Foreign body in other body part	309 (222 to 368)	148 (124 to 212)	13.7 (9.8 to 16.2)	4.6 (3.9 to 6.6)
Environmental heat and cold exposure	578 (531 to 632)	277 (249 to 305)	33.7 (31.3 to 36.4)	8.9 (8.0 to 9.8)
Exposure to forces of nature	56 (51 to 62)	26 (23 to 28)	2.5 (2.3 to 2.8)	0.8 (0.7 to 0.9)
Other unintentional injuries	1 395 (1 265 to 1 524)	894 (793 to 1 019)	65.8 (60.2 to 71.4)	27.6 (24.5 to 31.4)

Cause	Incidence: All-age counts (95% UI) 1990	Incidence: All-age counts (95% UI) 2017	Incidence: Age-standardised rates per 100,000 (95% UI) 1990	Incidence: Age-standardised rates per 100,000 (95% UI) 2017
**Transport and unintentional injuries**	**72 623 (66 082 to 80 189)**	**102 134 (91 046 to 114 044)**	**4 138.9 (3 762.4 to 4 544.3)**	**3 422.1 (3 045.2 to 3 819.6)**
**Transport injuries**	**6 393 (5 428 to 7 510)**	**12 210 (10 320 to 14 313)**	**340.7 (292.6 to 390.3)**	**386.0 (327.9 to 450.4)**
Road injuries	6 036 (5 086 to 7 156)	11 830 (9 938 to 13 928)	322.4 (274.2 to 370.8)	373.4 (315.5 to 439.1)
Pedestrian road injuries	935 (749 to 1 165)	1 341 (1 037 to 1 700)	52.7 (42.5 to 64.3)	42.8 (33.4 to 54.3)
Cyclist road injuries	831 (653 to 1 176)	1 580 (1 162 to 2 039)	44.7 (34.4 to 57.3)	49.2 (37.6 to 64.4)
Motorcyclist road injuries	944 (731 to 1 199)	1 423 (1 052 to 1 887)	47.9 (37.9 to 59.6)	44.6 (32.9 to 59.2)
Motor vehicle road injuries	2 260 (1 806 to 2 743)	5 700 (4 548 to 6 900)	122.6 (98.8 to 146.1)	177.9 (142.4 to 214.4)
Other road injuries	1 015 (730 to 1 319)	1 836 (1 386 to 2 358)	54.6 (41.5 to 68.2)	58.8 (44.9 to 74.8)
Other transport injuries	357 (262 to 504)	380 (262 to 527)	18.3 (14.4 to 23.9)	12.6 (8.7 to 17.3)
**Unintentional injuries**	**66 231 (59 949 to 73 553)**	**89 923 (78 729 to 101 710)**	**3 798.2 (3 424.9 to 4 212.1)**	**3 036.1 (2 678.5 to 3 430.9)**
Falls	26 758 (23 392 to 30 620)	48 258 (42 478 to 55 306)	1 736.8 (1 546.4 to 1 967.1)	1 684.2 (1 483.3 to 1 925.0)
Drowning	108 (92 to 127)	71 (56 to 91)	5.5 (4.7 to 6.3)	2.3 (1.8 to 2.9)
Fire, heat, and hot substances	2 734 (2 373 to 3 440)	1 967 (1 565 to 2 496)	119.9 (97.4 to 147.6)	64.1 (51.0 to 81.5)
Poisonings	947 (710 to 1 253)	874 (625 to 1 189)	38.5 (29.3 to 50.1)	28.9 (20.8 to 39.6)
Poisoning by carbon monoxide	193 (125 to 288)	161 (93 to 254)	8.5 (5.9 to 12.2)	5.3 (3.1 to 8.3)
Poisoning by other means	755 (557 to 1 034)	713 (497 to 1 002)	30.0 (22.3 to 40.7)	23.6 (16.4 to 33.2)
Exposure to mechanical forces	17 822 (15 301 to 21 337)	15 640 (13 000 to 18 810)	884.7 (763.4 to 1 040.0)	493.6 (410.9 to 592.5)
Unintentional firearm injuries	1 524 (1 135 to 2 042)	734 (493 to 1 061)	73.3 (54.7 to 95.2)	23.3 (15.8 to 33.2)
Other exposure to mechanical forces	16 298 (13 802 to 19 538)	14 906 (12 405 to 17 967)	811.4 (698.6 to 954.6)	470.3 (392.4 to 566.8)
Adverse effects of medical treatment	1 517 (1 264 to 1 796)	2 659 (2 218 to 3 229)	94.1 (79.9 to 109.2)	87.6 (73.5 to 105.6)
Animal contact	4 045 (3 339 to 5 201)	3 886 (3 200 to 4 739)	205.3 (164.2 to 254.2)	127.0 (104.7 to 154.0)
Venomous animal contact	1 409 (986 to 1 970)	1 275 (870 to 1 621)	70.8 (51.0 to 95.6)	41.4 (31.6 to 52.6)
Non-venomous animal contact	2 636 (1 976 to 3 487)	2 612 (2 097 to 3 285)	134.5 (104.4 to 171.6)	85.6 (68.7 to 107.3)
Foreign body	7 673 (4 077 to 12 312)	12 959 (5 197 to 22 265)	469.5 (215.3 to 770.4)	428.7 (169.7 to 741.7)
Pulmonary aspiration and foreign body in airway	265 (187 to 359)	155 (110 to 214)	11.2 (8.2 to 14.8)	5.2 (3.7 to 7.1)
Foreign body in eyes	6 794 (3 225 to 11 528)	12 388 (4 643 to 21 695)	429.7 (172.5 to 734.4)	409.7 (149.5 to 722.1)
Foreign body in other body part	614 (413 to 941)	415 (289 to 562)	28.6 (20.0 to 40.9)	13.8 (9.5 to 18.5)
Environmental heat and cold exposure	1 057 (744 to 1 427)	654 (465 to 840)	58.1 (44.5 to 72.7)	22.3 (15.9 to 31.7)
Exposure to forces of nature	147 (120 to 184)	34 (29 to 41)	6.5 (5.5 to 7.9)	1.1 (0.9 to 1.3)
Other unintentional injuries	3 428 (2 769 to 4 251)	2 920 (2 292 to 3 764)	179.2 (148.7 to 215.8)	96.2 (75.8 to 123.3)

Cause	Prevalence: All-age counts (95% UI) 1990	Prevalence: All-age counts (95% UI) 2017	Prevalence: Age-standardised rates per 100,000 (95% UI) 1990	Prevalence: Age-standardised rates per 100,000 (95% UI) 2017
**Transport and unintentional injuries**	**142 532 (133 959 to 152 634)**	**211 800 (197 940 to 228 676)**	**9 481.2 (8 916.0 to 10 170.9)**	**7 105.7 (6 653.0 to 7 670.5)**
**Transport injuries**	**15 628 (14 573 to 16 625)**	**35 171 (32 630 to 37 880)**	**1 037.5 (967.0 to 1 126.8)**	**1 151.6 (1 067.8 to 1 241.3)**
Road injuries	13 962 (12 991 to 14 932)	33 144 (30 652 to 35 782)	954.8 (888.0 to 1 023.3)	1 084.6 (1 003.6 to 1 170.8)
Pedestrian road injuries	3 422 (2 930 to 4 036)	6 162 (5 287 to 7 228)	236.9 (205.2 to 278.6)	204.0 (175.5 to 239.1)
Cyclist road injuries	1 466 (1 229 to 1 727)	3 304 (2 807 to 3 875)	95.6 (80.5 to 110.7)	108.4 (92.3 to 126.8)
Motorcyclist road injuries	3 307 (2 857 to 3 808)	6 487 (5 585 to 7 600)	220.6 (190.9 to 254.5)	209.5 (180.4 to 245.0)
Motor vehicle road injuries	3 902 (3 402 to 4 538)	12 798 (11 119 to 14 997)	276.5 (240.9 to 321.2)	418.5 (363.9 to 489.7)
Other road injuries	1 865 (1 547 to 2 247)	4 394 (3 662 to 5 362)	125.2 (104.3 to 149.9)	144.3 (120.5 to 175.3)
Other transport injuries	1 666 (1 369 to 1 999)	2 027 (1 708 to 2 350)	102.7 (86.3 to 119.9)	67.0 (56.9 to 77.3)
**Unintentional injuries**	**126 904 (118 266 to 137 003)**	**176 629 (163 274 to 193 541)**	**8 421.7 (7 850.1 to 9 095.8)**	**5 954.1 (5 515.4 to 6 510.4)**
Falls	46 909 (41 305 to 53 857)	94 733 (84 302 to 108 823)	3 400.5 (3 041.4 to 3 890.9)	3 281.6 (2 923.5 to 3 757.2)
Drowning	466 (433 to 577)	436 (381 to 511)	33.7 (29.8 to 38.5)	14.6 (12.8 to 17.1)
Fire, heat, and hot substances	26 994 (22 487 to 33 948)	23 251 (19 220 to 29 091)	1 568.1 (1 321.6 to 1 933.1)	752.3 (624.5 to 935.4)
Poisonings	856 (660 to 1 057)	1 039 (762 to 1 294)	44.1 (34.3 to 54.3)	33.0 (24.2 to 41.0)
Poisoning by carbon monoxide	137 (105 to 176)	127 (97 to 164)	7.3 (5.7 to 9.2)	4.1 (3.1 to 5.2)
Poisoning by other means	719 (525 to 910)	912 (639 to 1 159)	36.9 (27.2 to 46.5)	28.9 (20.2 to 36.7)
Exposure to mechanical forces	34 593 (30 260 to 39 760)	38 388 (33 423 to 44 372)	2 292.7 (2 018.3 to 2 625.3)	1 250.3 (1 090.8 to 1 441.5)
Unintentional firearm injuries	5 338 (4 183 to 6 437)	3 641 (2 942 to 4 359)	359.0 (285.7 to 433.1)	119.6 (97.3 to 143.2)
Other exposure to mechanical forces	29 254 (24 622 to 34 767)	34 748 (29 619 to 40 838)	1 933.7 (1 647.2 to 2 277.4)	1 130.7 (967.7 to 1 326.2)
Adverse effects of medical treatment	116 (89 to 145)	204 (155 to 261)	7.2 (5.5 to 9.0)	6.7 (5.1 to 8.6)
Animal contact	4 338 (3 727 to 5 048)	5 026 (4 333 to 5 800)	275.1 (239.7 to 334.4)	165.3 (143.0 to 190.0)
Venomous animal contact	924 (752 to 1 089)	939 (791 to 1 096)	54.6 (45.7 to 63.6)	30.4 (25.7 to 35.4)
Non-venomous animal contact	3 414 (2 840 to 4 109)	4 088 (3 416 to 4 858)	220.3 (185.0 to 259.9)	134.7 (112.9 to 159.6)
Foreign body	3 523 (2 712 to 4 685)	4 380 (3 119 to 6 326)	223.3 (174.1 to 293.5)	148.6 (106.5 to 213.1)
Pulmonary aspiration and foreign body in airway	196 (138 to 267)	140 (112 to 174)	10.3 (7.5 to 13.8)	4.5 (3.6 to 5.6)
Foreign body in eyes	1 343 (687 to 2 381)	2 670 (1 439 to 4 528)	97.7 (55.1 to 160.3)	92.7 (52.0 to 152.3)
Foreign body in other body part	1 985 (1 574 to 2 532)	1 571 (1 285 to 1 943)	115.4 (93.3 to 143.8)	51.3 (42.4 to 63.2)
Environmental heat and cold exposure	3 755 (3 042 to 4 567)	2 624 (2 169 to 3 180)	238.5 (199.6 to 283.0)	88.1 (73.2 to 106.6)
Exposure to forces of nature	355 (253 to 486)	1 484 (1 035 to 2 067)	17.2 (12.6 to 23.1)	46.2 (32.3 to 64.4)
Other unintentional injuries	4 968 (4 248 to 5 778)	5 068 (4 369 to 5 798)	323.2 (280.1 to 369.7)	167.3 (145.7 to 190.3)

Cause	Years lived with disability: All-age counts (95% UI) 1990	Years lived with disability: All-age counts (95% UI) 2017	Years lived with disability: Age-standardised rates per 100,000 (95% UI) 1990	Years lived with disability: Age-standardised rates per 100,000 (95% UI) 2017
**Transport and unintentional injuries**	**6 046 (4 373 to 8 025)**	**9 057 (6 482 to 12 154)**	**398.2 (287.7 to 530.1)**	**304.1 (217.7 to 408.7)**
**Transport injuries**	**1 062 (774 to 1 403)**	**2 134 (1 526 to 2 839)**	**68.9 (50.3 to 90.8)**	**69.0 (49.4 to 92.0)**
Road injuries	956 (696 to 1 262)	2 025 (1 453 to 2 707)	62.7 (45.6 to 82.7)	65.5 (47.0 to 87.4)
Pedestrian road injuries	245 (174 to 331)	393 (277 to 540)	16.2 (11.5 to 21.9)	12.8 (9.0 to 17.7)
Cyclist road injuries	101 (71 to 133)	199 (139 to 265)	6.4 (4.5 to 8.3)	6.5 (4.5 to 8.6)
Motorcyclist road injuries	228 (160 to 310)	400 (272 to 547)	14.7 (10.2 to 20.0)	12.8 (8.7 to 17.4)
Motor vehicle road injuries	276 (199 to 369)	810 (581 to 1 083)	18.7 (13.5 to 24.8)	26.1 (18.7 to 34.9)
Other road injuries	105 (75 to 143)	223 (158 to 309)	6.8 (4.9 to 9.2)	7.2 (5.1 to 10.0)
Other transport injuries	106 (75 to 143)	109 (77 to 147)	6.2 (4.4 to 8.3)	3.5 (2.5 to 4.8)
**Unintentional injuries**	**4 984 (3 611 to 6 651)**	**6 923 (4 906 to 9 369)**	**329.3 (237.4 to 439.2)**	**235.1 (166.5 to 318.3)**
Falls	2 760 (2 020 to 3 620)	4 790 (3 492 to 6 351)	193.8 (140.4 to 253.6)	165.5 (117.9 to 218.2)
Drowning	28 (20 to 39)	23 (16 to 32)	1.8 (1.3 to 2.5)	0.8 (0.5 to 1.1)
Fire, heat, and hot substances	577 (355 to 906)	447 (262 to 726)	32.5 (20.1 to 51.0)	14.4 (8.5 to 23.4)
Poisonings	100 (69 to 150)	120 (71 to 183)	5.0 (3.2 to 7.4)	3.8 (2.2 to 5.8)
Poisoning by carbon monoxide	15 (9 to 23)	12 (7 to 20)	0.7 (0.5 to 1.1)	0.4 (0.2 to 0.6)
Poisoning by other means	86 (52 to 129)	107 (63 to 165)	4.3 (2.6 to 6.4)	3.4 (2.0 to 5.2)
Exposure to mechanical forces	812 (561 to 1 163)	827 (557 to 1 209)	52.6 (36.1 to 75.4)	26.3 (18.1 to 39.1)
Unintentional firearm injuries	113 (75 to 170)	68 (44 to 106)	7.3 (4.8 to 11.2)	2.2 (1.4 to 3.5)
Other exposure to mechanical forces	699 (482 to 1 005)	759 (512 to 1 109)	45.3 (31.2 to 64.9)	24.6 (16.6 to 36.0)
Adverse effects of medical treatment	15 (9 to 24)	27 (17 to 41)	1.0 (0.6 to 1.5)	0.9 (0.6 to 1.4)
Animal contact	113 (76 to 161)	115 (76 to 167)	6.5 (4.4 to 9.2)	3.7 (2.5 to 5.4)
Venomous animal contact	61 (39 to 89)	58 (37 to 86)	3.2 (2.1 to 4.6)	1.8 (1.2 to 2.7)
Non-venomous animal contact	52 (31 to 83)	57 (33 to 93)	3.3 (2.0 to 5.2)	1.9 (1.1 to 3.1)
Foreign body	219 (149 to 326)	257 (155 to 425)	13.6 (9.0 to 20.1)	8.7 (5.3 to 14.3)
Pulmonary aspiration and foreign body in airway	10 (7 to 15)	6 (4 to 8)	0.5 (0.3 to 0.7)	0.2 (0.1 to 0.3)
Foreign body in eyes	89 (46 to 162)	178 (93 to 328)	6.5 (3.5 to 11.6)	6.2 (3.2 to 11.3)
Foreign body in other body part	119 (82 to 161)	73 (52 to 97)	6.6 (4.6 to 8.7)	2.4 (1.7 to 3.1)
Environmental heat and cold exposure	147 (98 to 208)	98 (63 to 142)	9.3 (6.2 to 12.9)	3.3 (2.1 to 4.8)
Exposure to forces of nature	19 (13 to 26)	48 (34 to 67)	0.9 (0.6 to 1.2)	1.5 (1.1 to 2.1)
Other unintentional injuries	193 (139 to 258)	170 (119 to 233)	12.2 (8.8 to 16.4)	5.6 (3.9 to 7.7)

Cause	Disability adjusted life years: All-age counts (95% UI) 1990	Disability adjusted life years: All-age counts (95% UI) 2017	Disability adjusted life years: Age-standardised rates per 100,000 (95% UI) 1990	Disability adjusted life years: Age-standardised rates per 100,000 (95% UI) 2017
**Transport and unintentional injuries**	**61 335 (57 425 to 65 437)**	**51 911 (47 551 to 56 377)**	**3 103.9 (2 933.6 to 3 293.5)**	**1 675.4 (1 533.1 to 1 819.6)**
**Transport injuries**	**26 358 (24 697 to 28 119)**	**24 530 (22 521 to 26 620)**	**1 293.4 (1 222.6 to 1 371.5)**	**767.0 (704.7 to 832.0)**
Road injuries	25 532 (23 933 to 27 228)	23 422 (21 504 to 25 464)	1 251.1 (1 183.4 to 1 325.2)	732.4 (673.2 to 794.8)
Pedestrian road injuries	10 073 (9 125 to 11 107)	7 959 (7 270 to 8 781)	505.2 (467.6 to 547.0)	253.2 (231.5 to 279.1)
Cyclist road injuries	758 (683 to 846)	1 095 (973 to 1 230)	38.9 (35.4 to 43.1)	34.6 (30.7 to 38.7)
Motorcyclist road injuries	4 433 (3 985 to 4 881)	3 829 (3 350 to 4 317)	210.9 (191.1 to 230.7)	117.3 (102.8 to 132.3)
Motor vehicle road injuries	9 697 (8 894 to 10 545)	9 913 (8 998 to 10 960)	466.9 (432.4 to 502.1)	307.5 (278.8 to 339.5)
Other road injuries	571 (498 to 658)	626 (537 to 722)	29.2 (25.6 to 33.0)	19.8 (17.0 to 23.0)
Other transport injuries	826 (744 to 917)	1 109 (978 to 1 254)	42.3 (38.5 to 46.6)	34.7 (30.6 to 39.1)
**Unintentional injuries**	**34 978 (32 279 to 37 901)**	**27 381 (24 536 to 30 447)**	**1 810.5 (1 681.3 to 1 949.6)**	**908.4 (812.7 to 1 011.5)**
Falls	6 536 (5 778 to 7 413)	8 459 (7 045 to 10 068)	452.0 (400.9 to 511.5)	293.4 (245.1 to 347.7)
Drowning	7 426 (6 689 to 8 296)	3 551 (3 160 to 3 959)	330.0 (301.4 to 364.5)	112.7 (100.2 to 125.5)
Fire, heat, and hot substances	2 977 (2 618 to 3 429)	1 652 (1 428 to 1 948)	153.8 (136.8 to 176.6)	54.7 (47.5 to 64.6)
Poisonings	1 981 (1 780 to 2 217)	1 149 (1 041 to 1 269)	90.7 (82.8 to 99.9)	36.7 (33.2 to 40.5)
Poisoning by carbon monoxide	889 (794 to 1 007)	558 (499 to 621)	41.1 (37.2 to 45.4)	17.8 (15.9 to 19.8)
Poisoning by other means	1 092 (966 to 1 241)	590 (519 to 674)	49.6 (44.5 to 55.7)	18.9 (16.6 to 21.6)
Exposure to mechanical forces	5 492 (5 061 to 5 958)	3 193 (2 838 to 3 645)	282.3 (259.5 to 308.0)	101.1 (89.8 to 115.5)
Unintentional firearm injuries	2 518 (2 290 to 2 955)	986 (867 to 1 130)	120.7 (110.0 to 141.9)	30.6 (27.0 to 35.0)
Other exposure to mechanical forces	2 974 (2 465 to 3 384)	2 208 (1 908 to 2 599)	161.6 (136.1 to 182.7)	70.5 (60.9 to 83.0)
Adverse effects of medical treatment	1 447 (1 182 to 1 637)	1 443 (1 292 to 1 804)	80.0 (65.4 to 88.2)	49.1 (43.9 to 60.8)
Animal contact	505 (425 to 595)	259 (214 to 312)	24.0 (20.3 to 27.9)	8.5 (7.0 to 10.2)
Venomous animal contact	314 (257 to 380)	125 (97 to 159)	13.9 (11.5 to 16.7)	4.1 (3.2 to 5.2)
Non-venomous animal contact	192 (157 to 232)	134 (105 to 174)	10.1 (8.2 to 12.4)	4.4 (3.5 to 5.7)
Foreign body	6 225 (5 431 to 7 099)	6 163 (5 243 to 7 334)	273.4 (243.0 to 307.1)	204.5 (173.4 to 244.6)
Pulmonary aspiration and foreign body in airway	5 707 (4 947 to 6 554)	5 763 (4 843 to 6 932)	246.6 (217.7 to 279.0)	191.3 (160.2 to 230.7)
Foreign body in eyes	89 (45 to 162)	178 (93 to 328)	6.5 (3.5 to 11.6)	6.2 (3.2 to 11.1)
Foreign body in other body part	428 (335 to 495)	221 (188 to 286)	20.3 (16.1 to 23.2)	7.0 (5.9 to 9.3)
Environmental heat and cold exposure	725 (649 to 805)	375 (330 to 432)	43.0 (38.9 to 47.7)	12.2 (10.7 to 14.1)
Exposure to forces of nature	75 (67 to 84)	74 (60 to 92)	3.4 (3.1 to 3.8)	2.3 (1.9 to 2.9)
Other unintentional injuries	1 588 (1 454 to 1 738)	1 064 (946 to 1 191)	78.0 (71.8 to 84.7)	33.2 (28.5 to 37.2)
