# Supplementary material for: Unintentional injuries in Mexico, 1990–2017: findings from the Global Burden of Disease Study 2017
Source: Inj Prev. 2020 Apr 1;26(Suppl 1):i154–61. doi: 10.1136/injuryprev-2019-043532 (PMC7571365; doi:10.1136/injuryprev-2019-043532)
Supplement: Supplementary data [file injuryprev-2019-043532supp012.pdf]

Death, YLL, incidence, prevalence, YLD, and DALY counts and rates per 100,000 for all injuries in Colima in 1990 and 2017

Cause	Deaths				Years of life lost			
	All-age counts (95% UI)		Age-standardised rates per 100,000 (95% UI)		All-age counts (95% UI)		Age-standardised rates per 100,000 (95% UI)	
	1990	2017	1990	2017	1990	2017	1990	2017
**Transport and unintentional injuries**	**302 (288 to 318)**	**288 (269 to 309)**	**87.4 (84.3 to 90.8)**	**41.1 (38.4 to 43.9)**	**16 406 (15 258 to 17 707)**	**11 916 (11 026 to 12 849)**	**3 683.6 (3 478.4 to 3 900.5)**	**1 573.8 (1 458.0 to 1 694.2)**
**Transport injuries**	**143 (135 to 150)**	**146 (134 to 159)**	**38.0 (36.2 to 39.8)**	**19.4 (17.8 to 21.1)**	**7 836 (7 324 to 8 365)**	**6 748 (6 148 to 7 372)**	**1 771.7 (1 671.4 to 1 871.5)**	**866.0 (790.2 to 947.5)**
Road injuries	136 (128 to 143)	135 (124 to 148)	36.2 (34.5 to 37.9)	18.0 (16.6 to 19.6)	7 492 (7 002 to 8 007)	6 253 (5 695 to 6 839)	1 691.4 (1 593.8 to 1 789.7)	803.2 (733.5 to 879.3)
Pedestrian road injuries	68 (63 to 73)	61 (56 to 67)	19.5 (18.5 to 20.7)	8.5 (7.8 to 9.3)	3 577 (3 247 to 3 957)	2 503 (2 262 to 2 779)	829.9 (760.2 to 895.2)	328.8 (297.9 to 364.1)
Cyclist road injuries	3 (3 to 4)	6 (5 to 7)	0.9 (0.8 to 1.0)	0.8 (0.7 to 0.9)	192 (171 to 214)	270 (236 to 306)	44.1 (39.8 to 48.9)	34.8 (30.4 to 39.3)
Motorcyclist road injuries	20 (18 to 22)	18 (16 to 21)	4.7 (4.3 to 5.2)	2.3 (2.0 to 2.6)	1 172 (1 043 to 1 316)	997 (861 to 1 150)	256.5 (231.2 to 284.2)	124.6 (107.8 to 143.6)
Motor vehicle road injuries	42 (38 to 45)	47 (42 to 51)	10.2 (9.4 to 11.0)	6.0 (5.4 to 6.6)	2 375 (2 164 to 2 577)	2 328 (2 084 to 2 582)	527.4 (484.7 to 568.2)	295.2 (264.2 to 327.5)
Other road injuries	3 (3 to 4)	3 (3 to 4)	0.8 (0.7 to 1.0)	0.4 (0.4 to 0.5)	177 (149 to 207)	155 (132 to 182)	39.5 (33.7 to 45.7)	19.9 (17.0 to 23.4)
Other transport injuries	6 (6 to 7)	11 (9 to 12)	1.8 (1.6 to 2.0)	1.4 (1.1 to 1.6)	344 (306 to 385)	494 (382 to 578)	80.4 (72.5 to 88.9)	62.8 (49.0 to 73.1)
**Unintentional injuries**	**160 (150 to 170)**	**142 (133 to 151)**	**49.4 (47.3 to 51.6)**	**21.6 (20.4 to 22.9)**	**8 570 (7 765 to 9 431)**	**5 168 (4 786 to 5 584)**	**1 911.8 (1 773.7 to 2 061.1)**	**707.8 (656.4 to 763.6)**
Falls	34 (32 to 35)	47 (42 to 51)	15.5 (14.7 to 16.3)	8.0 (7.3 to 8.6)	1 071 (1 001 to 1 148)	1 067 (904 to 1 168)	325.7 (308.6 to 344.6)	152.2 (130.8 to 166.0)
Drowning	42 (38 to 47)	25 (23 to 28)	10.0 (9.2 to 10.8)	3.4 (3.1 to 3.7)	2 635 (2 341 to 2 986)	1 264 (1 134 to 1 415)	539.9 (486.8 to 600.6)	167.5 (150.5 to 187.8)
Fire, heat, and hot substances	11 (10 to 12)	8 (7 to 9)	3.6 (3.4 to 3.8)	1.3 (1.2 to 1.4)	557 (482 to 647)	256 (218 to 281)	124.2 (111.0 to 140.2)	35.6 (30.8 to 38.9)
Poisonings	5 (4 to 5)	3 (3 to 4)	1.2 (1.1 to 1.3)	0.5 (0.4 to 0.5)	289 (256 to 328)	153 (122 to 171)	60.1 (54.0 to 67.2)	20.2 (16.4 to 22.7)
Poisoning by carbon monoxide	1 (1 to 1)	1 (1 to 1)	0.3 (0.2 to 0.3)	0.1 (0.1 to 0.1)	65 (56 to 75)	43 (32 to 51)	13.9 (12.1 to 15.9)	5.7 (4.3 to 6.6)
Poisoning by other means	4 (3 to 4)	2 (2 to 3)	0.9 (0.8 to 1.0)	0.3 (0.3 to 0.4)	224 (196 to 258)	109 (90 to 123)	46.2 (41.3 to 52.1)	14.6 (12.1 to 16.4)
Exposure to mechanical forces	17 (16 to 18)	11 (10 to 13)	4.7 (4.4 to 5.0)	1.6 (1.4 to 1.7)	927 (848 to 1 010)	498 (446 to 559)	215.3 (199.7 to 231.5)	63.9 (57.5 to 71.6)
Unintentional firearm injuries	8 (7 to 9)	4 (3 to 5)	2.1 (1.9 to 2.3)	0.5 (0.4 to 0.6)	450 (398 to 503)	190 (145 to 225)	102.0 (90.9 to 113.7)	24.0 (18.3 to 28.3)
Other exposure to mechanical forces	9 (8 to 10)	7 (7 to 9)	2.6 (2.4 to 2.8)	1.0 (0.9 to 1.2)	477 (419 to 534)	308 (271 to 374)	113.4 (100.2 to 124.8)	39.9 (35.3 to 48.1)
Adverse effects of medical treatment	10 (7 to 11)	14 (12 to 16)	4.1 (3.6 to 4.4)	2.3 (1.9 to 2.6)	459 (329 to 529)	402 (340 to 454)	111.8 (77.5 to 125.9)	57.9 (49.8 to 65.7)
Animal contact	13 (11 to 16)	7 (6 to 7)	3.3 (2.9 to 3.7)	1.0 (0.9 to 1.1)	891 (723 to 1 101)	268 (240 to 300)	174.6 (146.1 to 210.2)	37.7 (33.6 to 42.2)
Venomous animal contact	12 (10 to 15)	6 (5 to 6)	2.9 (2.5 to 3.4)	0.9 (0.7 to 0.9)	831 (663 to 1 037)	229 (199 to 259)	161.2 (132.0 to 196.9)	32.4 (28.1 to 36.8)
Non-venomous animal contact	1 (1 to 2)	1 (1 to 1)	0.3 (0.2 to 0.6)	0.1 (0.1 to 0.2)	60 (38 to 127)	39 (30 to 57)	13.4 (8.3 to 26.1)	5.3 (4.1 to 7.9)
Foreign body	18 (16 to 20)	18 (16 to 19)	4.3 (4.0 to 4.7)	2.6 (2.4 to 2.8)	1 202 (1 034 to 1 389)	887 (779 to 1 013)	236.2 (207.1 to 268.6)	125.1 (108.8 to 145.0)
Pulmonary aspiration and foreign body in airway	17 (15 to 19)	17 (16 to 19)	4.2 (3.8 to 4.5)	2.5 (2.3 to 2.8)	1 148 (986 to 1 327)	863 (758 to 988)	225.3 (197.8 to 256.4)	122.0 (105.8 to 141.4)
Foreign body in eyes								
Foreign body in other body part	1 (1 to 1)	0 (0 to 1)	0.2 (0.1 to 0.2)	0.1 (0.0 to 0.1)	54 (37 to 64)	23 (19 to 37)	10.9 (7.6 to 12.8)	3.0 (2.4 to 4.8)
Environmental heat and cold exposure	2 (2 to 2)	1 (1 to 1)	0.7 (0.7 to 0.8)	0.2 (0.2 to 0.2)	90 (82 to 98)	43 (35 to 48)	23.5 (21.7 to 25.2)	5.7 (4.7 to 6.3)
Exposure to forces of nature	0 (0 to 0)	0 (0 to 0)	0.1 (0.1 to 0.1)	0.0 (0.0 to 0.0)	13 (12 to 15)	6 (6 to 7)	2.8 (2.6 to 3.1)	0.8 (0.7 to 0.9)
Other unintentional injuries	8 (7 to 9)	7 (6 to 7)	2.0 (1.8 to 2.1)	0.8 (0.7 to 1.0)	441 (399 to 491)	326 (284 to 375)	97.6 (89.3 to 107.7)	41.2 (36.0 to 47.3)

Cause	Incidence				Prevalence			
	All-age counts (95% UI)		Age-standardised rates per 100,000 (95% UI)		All-age counts (95% UI)		Age-standardised rates per 100,000 (95% UI)	
	1990	2017	1990	2017	1990	2017	1990	2017
**Transport and unintentional injuries**	**18 432 (16 972 to 20 183)**	**29 757 (26 878 to 32 982)**	**4 847.0 (4 444.9 to 5 295.7)**	**4 138.4 (3 748.3 to 4 567.6)**	**34 506 (32 375 to 36 992)**	**61 967 (58 119 to 67 020)**	**10 661.6 (10 070.2 to 11 450.5)**	**8 479.1 (7 969.7 to 9 157.2)**
**Transport injuries**	**1 664 (1 442 to 1 928)**	**3 728 (3 256 to 4 278)**	**414.8 (362.9 to 470.4)**	**485.2 (424.2 to 556.0)**	**4 083 (3 805 to 4 353)**	**10 832 (10 063 to 11 667)**	**1 294.2 (1 208.7 to 1 382.5)**	**1 435.0 (1 331.9 to 1 543.7)**
Road injuries	1 557 (1 346 to 1 822)	3 610 (3 136 to 4 154)	388.3 (339.7 to 444.5)	469.0 (408.3 to 538.9)	3 600 (3 345 to 3 856)	10 214 (9 466 to 11 029)	1 149.2 (1 073.9 to 1 232.1)	1 351.5 (1 251.9 to 1 458.9)
Pedestrian road injuries	274 (223 to 336)	390 (310 to 489)	71.5 (59.1 to 85.5)	51.7 (41.2 to 64.2)	998 (854 to 1 176)	1 805 (1 557 to 2 112)	321.1 (278.6 to 375.1)	242.8 (210.4 to 283.3)
Cyclist road injuries	207 (157 to 269)	394 (309 to 502)	48.5 (38.4 to 61.2)	53.0 (41.6 to 67.3)	341 (283 to 402)	880 (742 to 1 028)	103.6 (86.7 to 120.0)	117.2 (99.2 to 136.2)
Motorcyclist road injuries	209 (166 to 262)	426 (331 to 536)	49.5 (39.9 to 60.5)	54.8 (42.8 to 68.7)	737 (639 to 851)	1 967 (1 688 to 2 298)	230.8 (199.7 to 266.7)	256.4 (220.2 to 298.3)
Motor vehicle road injuries	587 (484 to 699)	1 802 (1 505 to 2 139)	149.9 (125.0 to 175.6)	230.8 (193.1 to 272.2)	1 019 (888 to 1 190)	4 120 (3 601 to 4 814)	335.8 (290.8 to 391.9)	543.5 (474.5 to 634.7)
Other road injuries	279 (216 to 353)	598 (470 to 741)	69.5 (55.2 to 84.2)	78.7 (62.3 to 96.6)	505 (420 to 609)	1 441 (1 203 to 1 761)	157.9 (132.3 to 189.2)	191.6 (160.0 to 233.3)
Other transport injuries	107 (85 to 139)	118 (90 to 153)	26.5 (22.0 to 32.0)	16.2 (12.4 to 21.1)	484 (402 to 565)	618 (528 to 714)	145.1 (123.7 to 167.4)	83.5 (71.7 to 95.9)
**Unintentional injuries**	**16 768 (15 340 to 18 530)**	**26 029 (23 280 to 29 214)**	**4 432.1 (4 035.3 to 4 884.1)**	**3 651.2 (3 281.9 to 4 087.1)**	**30 423 (28 342 to 32 872)**	**51 134 (47 439 to 55 931)**	**9 367.4 (8 765.7 to 10 110.9)**	**7 044.1 (6 531.8 to 7 716.3)**
Falls	7 152 (6 282 to 8 149)	14 679 (13 088 to 16 621)	2 087.8 (1 865.4 to 2 346.1)	2 130.0 (1 899.2 to 2 410.5)	12 483 (10 964 to 14 260)	28 361 (25 288 to 32 510)	4 139.2 (3 684.5 to 4 704.9)	4 024.6 (3 593.5 to 4 579.0)
Drowning	32 (28 to 36)	21 (17 to 25)	7.5 (6.6 to 8.5)	2.8 (2.3 to 3.4)	146 (128 to 168)	127 (112 to 147)	46.6 (41.2 to 53.0)	17.4 (15.3 to 20.0)
Fire, heat, and hot substances	588 (468 to 745)	497 (394 to 623)	116.5 (94.8 to 143.6)	70.1 (55.4 to 87.5)	5 704 (4 744 to 7 268)	6 172 (5 102 to 7 869)	1 535.2 (1 289.1 to 1 897.3)	813.3 (672.8 to 1 030.6)
Poisonings	171 (124 to 241)	165 (115 to 232)	31.3 (23.0 to 42.7)	23.7 (16.4 to 33.9)	152 (116 to 190)	204 (151 to 254)	36.5 (28.2 to 45.3)	26.7 (19.8 to 33.2)
Poisoning by carbon monoxide	35 (22 to 56)	48 (24 to 78)	7.1 (4.6 to 10.6)	6.7 (3.8 to 11.1)	25 (19 to 32)	42 (32 to 54)	6.1 (4.7 to 7.7)	5.5 (4.2 to 7.1)
Poisoning by other means	136 (95 to 197)	117 (76 to 173)	24.2 (17.4 to 34.5)	17.0 (11.0 to 25.4)	127 (92 to 163)	162 (114 to 209)	30.4 (22.1 to 38.7)	21.2 (14.9 to 27.3)
Exposure to mechanical forces	3 435 (2 921 to 4 078)	4 242 (3 646 to 4 907)	800.2 (687.5 to 929.3)	555.5 (480.7 to 641.6)	6 716 (5 886 to 7 701)	10 656 (9 281 to 12 359)	2 090.5 (1 842.5 to 2 384.3)	1 405.3 (1 226.8 to 1 624.4)
Unintentional firearm injuries	256 (189 to 349)	165 (117 to 230)	58.3 (43.6 to 76.5)	21.5 (15.4 to 29.7)	938 (747 to 1 122)	862 (704 to 1 032)	297.4 (239.0 to 358.6)	114.6 (94.1 to 137.1)
Other exposure to mechanical forces	3 178 (2 696 to 3 778)	4 077 (3 491 to 4 728)	741.9 (637.2 to 864.6)	534.0 (460.8 to 629.1)	5 778 (4 899 to 6 819)	9 793 (8 383 to 11 525)	1 793.0 (1 534.9 to 2 102.2)	1 290.7 (1 107.3 to 1 512.7)
Adverse effects of medical treatment	383 (328 to 439)	857 (727 to 997)	110.7 (95.9 to 125.8)	115.9 (97.9 to 133.5)	29 (23 to 36)	66 (50 to 82)	8.5 (6.5 to 10.4)	8.8 (6.7 to 11.0)
Animal contact	2 260 (1 932 to 2 676)	1 234 (1 007 to 1 483)	544.8 (473.5 to 623.9)	173.2 (140.4 to 205.5)	2 353 (2 035 to 2 719)	1 505 (1 319 to 1 707)	698.1 (614.4 to 789.1)	205.2 (180.6 to 232.9)
Venomous animal contact	1 003 (782 to 1 260)	476 (358 to 607)	241.2 (191.8 to 293.7)	65.8 (49.9 to 84.2)	718 (591 to 870)	348 (291 to 401)	202.9 (170.7 to 240.2)	47.1 (39.7 to 54.5)
Non-venomous animal contact	1 257 (1 014 to 1 562)	758 (597 to 955)	303.6 (249.9 to 367.6)	105.4 (83.4 to 132.2)	1 635 (1 387 to 1 973)	1 157 (980 to 1 357)	495.2 (412.9 to 585.3)	158.1 (133.7 to 184.8)
Foreign body	1 659 (843 to 2 856)	3 250 (1 253 to 5 689)	473.4 (210.6 to 827.5)	436.8 (168.3 to 745.2)	710 (538 to 969)	1 122 (798 to 1 624)	208.9 (159.8 to 283.3)	154.2 (110.1 to 219.9)
Pulmonary aspiration and foreign body in airway	47 (33 to 65)	32 (23 to 44)	9.0 (6.6 to 12.2)	4.6 (3.3 to 6.3)	34 (24 to 46)	31 (25 to 38)	8.3 (6.1 to 11.2)	4.1 (3.3 to 5.0)
Foreign body in eyes	1 495 (676 to 2 684)	3 111 (1 108 to 5 558)	439.8 (175.6 to 789.9)	417.0 (147.5 to 725.5)	300 (156 to 543)	672 (360 to 1 161)	100.1 (55.7 to 168.7)	94.1 (52.0 to 157.3)
Foreign body in other body part	117 (80 to 179)	108 (77 to 142)	24.6 (17.4 to 34.2)	15.1 (10.8 to 20.1)	376 (300 to 484)	420 (346 to 520)	99.9 (80.9 to 125.8)	56.0 (46.4 to 69.7)
Environmental heat and cold exposure	217 (153 to 298)	163 (116 to 222)	51.3 (38.0 to 65.6)	23.1 (16.7 to 31.6)	780 (631 to 955)	676 (556 to 804)	223.4 (185.5 to 268.0)	92.0 (77.5 to 109.1)
Exposure to forces of nature	35 (29 to 44)	8 (7 to 10)	7.2 (6.1 to 8.7)	1.1 (0.9 to 1.3)	140 (100 to 192)	618 (434 to 864)	34.1 (24.7 to 46.8)	78.8 (55.5 to 110.0)
Other unintentional injuries	886 (692 to 1 032)	912 (747 to 1 104)	201.0 (170.3 to 238.0)	123.6 (101.5 to 148.9)	1 210 (1 038 to 1 403)	1 629 (1 412 to 1 865)	367.9 (318.6 to 420.4)	218.0 (190.1 to 247.8)

Cause	Years lived with disability				Disability adjusted life years			
	All-age counts (95% UI)		Age-standardised rates per 100,000 (95% UI)		All-age counts (95% UI)		Age-standardised rates per 100,000 (95% UI)	
	1990	2017	1990	2017	1990	2017	1990	2017
**Transport and unintentional injuries**	**1 525 (1 106 to 2 025)**	**2 688 (1 946 to 3 600)**	**464.5 (338.8 to 614.7)**	**368.6 (265.9 to 495.3)**	**17 930 (16 676 to 19 280)**	**14 604 (13 421 to 15 920)**	**4 148.1 (3 906.4 to 4 421.2)**	**1 942.4 (1 785.4 to 2 120.6)**
**Transport injuries**	**277 (201 to 367)**	**636 (477 to 871)**	**84.5 (61.6 to 111.6)**	**85.9 (62.5 to 114.2)**	**8 113 (7 599 to 8 655)**	**7 403 (6 777 to 8 060)**	**1 856.3 (1 750.3 to 1 962.9)**	**951.9 (873.1 to 1 035.2)**
Road injuries	247 (179 to 328)	623 (452 to 827)	75.8 (55.4 to 100.6)	81.5 (59.2 to 108.4)	7 739 (7 236 to 8 265)	6 876 (6 305 to 7 497)	1 767.1 (1 664.3 to 1 867.6)	884.7 (811.7 to 963.1)
Pedestrian road injuries	72 (51 to 97)	115 (81 to 159)	22.0 (15.8 to 29.8)	15.3 (10.8 to 21.1)	3 648 (3 318 to 4 028)	2 618 (2 378 to 2 885)	845.9 (781.9 to 917.2)	344.1 (313.4 to 377.9)
Cyclist road injuries	24 (17 to 31)	53 (37 to 70)	6.9 (5.0 to 9.1)	7.0 (4.9 to 9.3)	215 (194 to 241)	323 (285 to 365)	51.0 (46.3 to 56.3)	41.8 (37.0 to 47.1)
Motorcyclist road injuries	51 (35 to 69)	121 (83 to 165)	15.4 (10.7 to 20.9)	15.6 (10.8 to 21.3)	1 223 (1 095 to 1 366)	1 118 (975 to 1 274)	271.9 (245.6 to 300.3)	140.2 (122.6 to 159.7)
Motor vehicle road injuries	72 (52 to 96)	261 (188 to 348)	22.8 (16.5 to 30.4)	34.0 (24.6 to 45.4)	2 447 (2 237 to 2 654)	2 589 (2 338 to 2 854)	550.2 (507.4 to 590.9)	329.2 (297.8 to 362.6)
Other road injuries	28 (20 to 38)	73 (52 to 100)	8.5 (6.2 to 11.6)	9.6 (6.9 to 13.1)	206 (176 to 236)	228 (194 to 267)	48.1 (41.6 to 54.6)	29.5 (25.2 to 34.5)
Other transport injuries	31 (22 to 40)	33 (23 to 44)	8.7 (6.3 to 11.5)	4.4 (3.1 to 5.9)	374 (337 to 417)	527 (416 to 610)	89.1 (81.5 to 97.8)	67.2 (53.3 to 77.4)
**Unintentional injuries**	**1 247 (906 to 1 666)**	**2 033 (1 461 to 2 733)**	**380.0 (274.5 to 507.9)**	**282.7 (201.5 to 380.6)**	**9 817 (8 946 to 10 813)**	**7 201 (6 619 to 8 031)**	**2 291.9 (2 112.1 to 2 491.2)**	**990.4 (897.6 to 1 106.6)**
Falls	743 (544 to 973)	1 446 (1 036 to 1 915)	237.6 (173.4 to 309.1)	204.7 (145.9 to 272.5)	1 814 (1 600 to 2 067)	2 512 (2 089 to 3 002)	563.3 (496.8 to 639.2)	356.9 (296.6 to 426.2)
Drowning	8 (6 to 11)	7 (5 to 9)	2.5 (1.8 to 3.5)	0.9 (0.6 to 1.3)	2 643 (2 351 to 2 996)	1 270 (1 139 to 1 420)	542.5 (489.3 to 602.9)	168.4 (151.4 to 188.7)
Fire, heat, and hot substances	123 (77 to 193)	119 (70 to 192)	32.1 (20.1 to 50.1)	15.7 (9.3 to 25.3)	680 (588 to 792)	375 (316 to 449)	156.4 (137.3 to 180.5)	51.2 (43.4 to 61.2)
Poisonings	18 (11 to 27)	23 (14 to 35)	4.2 (2.6 to 6.3)	3.1 (1.8 to 4.6)	307 (271 to 347)	176 (145 to 198)	64.3 (57.7 to 71.4)	23.3 (19.2 to 26.2)
Poisoning by carbon monoxide	3 (2 to 4)	4 (3 to 7)	0.6 (0.4 to 1.0)	0.6 (0.3 to 0.9)	67 (58 to 78)	48 (36 to 56)	14.5 (12.7 to 16.5)	6.2 (4.8 to 7.2)
Poisoning by other means	15 (9 to 24)	19 (11 to 29)	3.6 (2.1 to 5.4)	2.5 (1.4 to 3.8)	239 (211 to 273)	129 (108 to 145)	49.8 (44.6 to 55.7)	17.1 (14.4 to 19.3)
Exposure to mechanical forces	158 (108 to 224)	230 (154 to 335)	48.1 (33.1 to 68.9)	30.2 (20.3 to 44.1)	1 085 (990 to 1 197)	728 (633 to 850)	263.5 (241.2 to 290.8)	94.1 (82.1 to 109.8)
Unintentional firearm injuries	19 (13 to 30)	16 (10 to 25)	5.9 (3.9 to 9.1)	2.1 (1.3 to 3.3)	469 (416 to 522)	206 (160 to 242)	107.9 (96.6 to 120.0)	26.1 (20.4 to 30.5)
Other exposure to mechanical forces	139 (95 to 197)	214 (144 to 314)	42.2 (28.9 to 60.3)	28.1 (18.9 to 41.2)	616 (545 to 701)	521 (440 to 637)	155.6 (137.6 to 178.7)	68.1 (57.5 to 82.9)
Adverse effects of medical treatment	4 (2 to 6)	9 (5 to 13)	1.1 (0.7 to 1.7)	1.2 (0.7 to 1.8)	457 (327 to 528)	410 (347 to 464)	112.9 (78.6 to 127.0)	59.1 (50.2 to 67.1)
Animal contact	64 (43 to 90)	34 (23 to 50)	17.0 (11.5 to 24.0)	4.6 (3.0 to 6.7)	955 (784 to 1 159)	303 (272 to 339)	191.6 (162.5 to 226.1)	42.3 (37.8 to 47.4)
Venomous animal contact	39 (25 to 57)	18 (11 to 26)	9.6 (6.3 to 13.7)	2.4 (1.5 to 3.4)	870 (703 to 1 071)	247 (218 to 278)	170.8 (140.6 to 205.4)	34.7 (30.4 to 39.2)
Non-venomous animal contact	25 (15 to 40)	16 (10 to 27)	7.4 (4.5 to 12.0)	2.2 (1.3 to 3.7)	85 (57 to 158)	55 (43 to 77)	20.8 (14.0 to 35.5)	7.5 (5.8 to 10.5)
Foreign body	45 (30 to 68)	66 (39 to 109)	12.9 (8.5 to 19.6)	9.0 (5.4 to 14.8)	1 247 (1 075 to 1 433)	952 (839 to 1 087)	249.2 (218.8 to 282.1)	134.1 (117.0 to 154.4)
Pulmonary aspiration and foreign body in airway	2 (1 to 3)	1 (1 to 2)	0.4 (0.3 to 0.6)	0.2 (0.1 to 0.2)	1 150 (988 to 1 329)	865 (759 to 989)	225.8 (198.2 to 256.8)	122.2 (105.9 to 141.6)
Foreign body in eyes	20 (10 to 37)	45 (23 to 84)	6.7 (3.6 to 12.2)	6.3 (3.3 to 11.5)	20 (10 to 37)	45 (23 to 84)	6.7 (3.6 to 12.2)	6.3 (3.3 to 11.5)
Foreign body in other body part	23 (16 to 31)	20 (14 to 26)	5.8 (4.1 to 7.7)	2.6 (1.8 to 3.5)	77 (60 to 91)	43 (35 to 58)	16.7 (13.2 to 19.4)	5.6 (4.6 to 7.7)
Environmental heat and cold exposure	31 (20 to 44)	25 (17 to 37)	8.7 (5.8 to 12.2)	3.5 (2.3 to 5.0)	120 (107 to 136)	69 (57 to 83)	32.2 (28.6 to 36.2)	9.2 (7.7 to 11.1)
Exposure to forces of nature	7 (5 to 11)	20 (15 to 28)	1.8 (1.3 to 2.5)	2.5 (1.9 to 3.6)	21 (18 to 24)	26 (21 to 34)	4.6 (4.1 to 5.4)	3.4 (2.7 to 4.4)
Other unintentional injuries	47 (34 to 63)	54 (38 to 75)	13.9 (10.0 to 18.6)	7.3 (5.2 to 10.0)	488 (444 to 539)	380 (337 to 433)	111.5 (102.3 to 122.1)	48.4 (43.1 to 54.9)
