# Supplementary material for: Unintentional injuries in Mexico, 1990–2017: findings from the Global Burden of Disease Study 2017
Source: Inj Prev. 2020 Apr 1;26(Suppl 1):i154–61. doi: 10.1136/injuryprev-2019-043532 (PMC7571365; doi:10.1136/injuryprev-2019-043532)
Supplement: Supplementary data [file injuryprev-2019-043532supp019.pdf]

Death, YLL, incidence, prevalence, YLD, and DALY counts and rates per 100,000 for all injuries in México in 1990 and 2017

Deaths

Cause	All-age counts (95% UI) 1990	All-age counts (95% UI) 2017	Age-standardised rates per 100,000 (95% UI) 1990	Age-standardised rates per 100,000 (95% UI) 2017
**Transport and unintentional injuries**	**4 660 (4 496 to 4 846)**	**4 788 (4 390 to 5 235)**	**60.4 (58.8 to 62.1)**	**30.3 (27.9 to 33.0)**
**Transport injuries**	**2 269 (2 153 to 2 386)**	**2 430 (2 111 to 2 684)**	**27.2 (26.1 to 28.4)**	**14.4 (12.6 to 15.8)**
Road injuries	2 222 (2 107 to 2 337)	2 348 (2 040 to 2 593)	26.6 (25.5 to 27.8)	13.9 (12.2 to 15.3)
Pedestrian road injuries	1 279 (1 204 to 1 355)	1 239 (1 098 to 1 360)	16.7 (15.9 to 17.4)	7.6 (6.8 to 8.3)
Cyclist road injuries	42 (38 to 47)	72 (62 to 82)	0.5 (0.4 to 0.5)	0.4 (0.4 to 0.5)
Motorcyclist road injuries	264 (236 to 291)	227 (192 to 263)	2.7 (2.4 to 2.9)	1.3 (1.1 to 1.5)
Motor vehicle road injuries	576 (526 to 628)	745 (614 to 850)	6.1 (5.7 to 6.6)	4.2 (3.5 to 4.8)
Other road injuries	61 (50 to 71)	65 (55 to 76)	0.7 (0.6 to 0.8)	0.4 (0.3 to 0.4)
Other transport injuries	48 (44 to 52)	82 (69 to 94)	0.6 (0.6 to 0.6)	0.5 (0.4 to 0.5)
**Unintentional injuries**	**2 390 (2 295 to 2 499)**	**2 358 (2 186 to 2 585)**	**33.1 (32.2 to 34.4)**	**15.9 (14.8 to 17.5)**
Falls	538 (514 to 567)	823 (754 to 894)	11.2 (10.6 to 11.9)	6.2 (5.7 to 6.7)
Drowning	476 (441 to 514)	282 (257 to 310)	5.0 (4.7 to 5.3)	1.7 (1.5 to 1.8)
Fire, heat, and hot substances	181 (168 to 196)	130 (121 to 139)	2.6 (2.5 to 2.8)	0.9 (0.9 to 1.0)
Poisonings	98 (91 to 105)	68 (62 to 74)	1.1 (1.0 to 1.1)	0.4 (0.4 to 0.5)
Poisoning by carbon monoxide	46 (42 to 50)	35 (32 to 39)	0.5 (0.5 to 0.5)	0.2 (0.2 to 0.2)
Poisoning by other means	52 (47 to 56)	33 (30 to 36)	0.6 (0.5 to 0.6)	0.2 (0.2 to 0.2)
Exposure to mechanical forces	277 (260 to 293)	188 (171 to 204)	3.3 (3.1 to 3.5)	1.1 (1.0 to 1.2)
Unintentional firearm injuries	121 (107 to 132)	60 (49 to 68)	1.3 (1.2 to 1.4)	0.4 (0.3 to 0.4)
Other exposure to mechanical forces	156 (144 to 169)	128 (114 to 149)	2.0 (1.9 to 2.1)	0.8 (0.7 to 0.9)
Adverse effects of medical treatment	96 (88 to 126)	170 (143 to 285)	1.8 (1.6 to 2.3)	1.2 (1.0 to 2.0)
Animal contact	49 (43 to 56)	27 (24 to 29)	0.6 (0.5 to 0.6)	0.2 (0.2 to 0.2)
Venomous animal contact	33 (28 to 39)	12 (9 to 15)	0.3 (0.3 to 0.4)	0.1 (0.1 to 0.1)
Non-venomous animal contact	16 (14 to 18)	14 (13 to 18)	0.2 (0.2 to 0.2)	0.1 (0.1 to 0.1)
Foreign body	488 (446 to 531)	531 (470 to 609)	5.4 (5.1 to 5.8)	3.3 (2.9 to 3.8)
Pulmonary aspiration and foreign body in airway	471 (429 to 514)	520 (461 to 592)	5.2 (4.9 to 5.6)	3.3 (2.9 to 3.7)
Foreign body in eyes				
Foreign body in other body part	17 (15 to 21)	11 (8 to 21)	0.2 (0.1 to 0.2)	0.1 (0.0 to 0.1)
Environmental heat and cold exposure	35 (33 to 37)	21 (19 to 24)	0.6 (0.5 to 0.6)	0.1 (0.1 to 0.2)
Exposure to forces of nature	6 (5 to 7)	3 (3 to 3)	0.1 (0.1 to 0.1)	0.0 (0.0 to 0.0)
Other unintentional injuries	147 (138 to 157)	117 (105 to 131)	1.6 (1.5 to 1.7)	0.7 (0.6 to 0.8)

Years of life lost

Cause	All-age counts (95% UI) 1990	All-age counts (95% UI) 2017	Age-standardised rates per 100,000 (95% UI) 1990	Age-standardised rates per 100,000 (95% UI) 2017
**Transport and unintentional injuries**	**258 929 (246 638 to 272 675)**	**217 560 (197 791 to 241 182)**	**2 584.8 (2 485.7 to 2 694.0)**	**1 265.2 (1 150.0 to 1 402.3)**
**Transport injuries**	**125 308 (117 867 to 132 810)**	**118 960 (103 273 to 131 893)**	**1 249.9 (1 186.9 to 1 316.0)**	**671.1 (584.3 to 743.5)**
Road injuries	122 720 (115 298 to 130 084)	114 992 (99 853 to 127 740)	1 223.2 (1 161.2 to 1 288.6)	649.1 (564.1 to 719.1)
Pedestrian road injuries	68 523 (63 747 to 73 588)	56 149 (49 682 to 62 118)	709.3 (668.6 to 750.7)	321.7 (285.4 to 355.7)
Cyclist road injuries	2 344 (2 096 to 2 611)	3 338 (2 857 to 3 828)	23.3 (21.0 to 25.7)	18.9 (16.2 to 21.6)
Motorcyclist road injuries	15 403 (13 677 to 17 138)	12 940 (10 907 to 15 057)	144.0 (129.2 to 157.9)	71.3 (60.1 to 83.0)
Motor vehicle road injuries	32 947 (29 799 to 36 124)	39 310 (32 578 to 44 807)	313.3 (286.6 to 340.5)	218.9 (182.1 to 249.4)
Other road injuries	3 504 (2 802 to 4 125)	3 254 (2 724 to 3 823)	33.3 (27.3 to 38.5)	18.3 (15.3 to 21.4)
Other transport injuries	2 588 (2 374 to 2 837)	3 968 (3 295 to 4 640)	26.7 (24.7 to 28.9)	22.2 (18.5 to 25.8)
**Unintentional injuries**	**133 621 (125 945 to 142 139)**	**98 620 (90 160 to 110 864)**	**1 334.9 (1 274.0 to 1 406.0)**	**593.9 (542.3 to 669.0)**
Falls	20 993 (19 838 to 22 271)	23 317 (21 027 to 25 822)	284.7 (271.7 to 300.5)	145.1 (132.3 to 160.6)
Drowning	29 767 (27 096 to 32 829)	14 933 (13 467 to 16 499)	266.7 (245.9 to 289.7)	85.8 (77.4 to 94.9)
Fire, heat, and hot substances	10 337 (9 335 to 11 597)	4 569 (4 154 to 5 000)	101.7 (93.5 to 111.7)	28.0 (25.5 to 30.6)
Poisonings	6 130 (5 591 to 6 719)	3 285 (2 983 to 3 636)	55.4 (51.3 to 59.9)	19.1 (17.3 to 21.2)
Poisoning by carbon monoxide	2 829 (2 561 to 3 110)	1 688 (1 518 to 1 889)	25.6 (23.3 to 27.8)	9.7 (8.8 to 10.9)
Poisoning by other means	3 301 (2 963 to 3 666)	1 597 (1 428 to 1 787)	29.8 (27.2 to 32.6)	9.3 (8.4 to 10.5)
Exposure to mechanical forces	15 415 (14 391 to 16 449)	8 445 (7 638 to 9 298)	152.7 (143.6 to 161.7)	48.0 (43.4 to 52.4)
Unintentional firearm injuries	6 973 (6 111 to 7 695)	2 906 (2 085 to 3 334)	65.5 (57.7 to 71.8)	16.2 (11.7 to 18.6)
Other exposure to mechanical forces	8 441 (7 724 to 9 258)	5 540 (4 865 to 6 614)	87.2 (80.5 to 94.3)	31.7 (28.0 to 37.8)
Adverse effects of medical treatment	4 546 (4 054 to 5 994)	5 635 (4 561 to 9 905)	52.0 (47.5 to 68.6)	35.6 (29.0 to 61.9)
Animal contact	3 354 (2 864 to 3 948)	1 108 (993 to 1 242)	29.5 (25.7 to 34.0)	6.7 (6.1 to 7.6)
Venomous animal contact	2 440 (1 995 to 2 943)	526 (392 to 652)	20.5 (17.1 to 24.3)	3.3 (2.4 to 4.0)
Non-venomous animal contact	914 (777 to 1 056)	582 (496 to 753)	9.0 (7.6 to 10.2)	3.5 (3.0 to 4.5)
Foreign body	32 354 (28 880 to 35 990)	30 700 (26 136 to 36 511)	288.8 (262.8 to 316.6)	187.2 (157.5 to 224.8)
Pulmonary aspiration and foreign body in airway	31 276 (27 875 to 34 949)	30 116 (25 606 to 35 866)	279.2 (252.9 to 306.6)	183.9 (154.8 to 221.1)
Foreign body in eyes				
Foreign body in other body part	1 078 (924 to 1 311)	583 (427 to 1 196)	9.6 (8.4 to 12.0)	3.3 (2.4 to 6.8)
Environmental heat and cold exposure	1 698 (1 555 to 1 829)	787 (693 to 887)	19.8 (18.4 to 21.1)	4.6 (4.1 to 5.1)
Exposure to forces of nature	350 (315 to 391)	141 (129 to 153)	3.5 (3.1 to 3.9)	0.8 (0.7 to 0.9)
Other unintentional injuries	8 682 (8 115 to 9 272)	5 922 (5 288 to 6 704)	80.1 (75.4 to 85.0)	33.0 (29.5 to 37.2)

Incidence

Cause	All-age counts (95% UI) 1990	All-age counts (95% UI) 2017	Age-standardised rates per 100,000 (95% UI) 1990	Age-standardised rates per 100,000 (95% UI) 2017
**Transport and unintentional injuries**	**339 687 (313 334 to 369 579)**	**654 236 (600 009 to 712 299)**	**4 004.1 (3 708.3 to 4 319.9)**	**4 047.5 (3 726.2 to 4 414.3)**
**Transport injuries**	**30 156 (25 589 to 35 707)**	**62 817 (53 306 to 73 692)**	**336.1 (288.5 to 386.4)**	**362.3 (308.2 to 424.5)**
Road injuries	28 748 (24 323 to 34 405)	61 330 (51 859 to 72 225)	322.3 (274.9 to 371.7)	353.2 (299.3 to 414.1)
Pedestrian road injuries	5 485 (4 387 to 6 821)	9 595 (7 542 to 12 063)	66.8 (55.0 to 80.5)	56.2 (44.3 to 70.1)
Cyclist road injuries	3 831 (2 753 to 5 205)	7 190 (5 288 to 9 624)	39.9 (30.1 to 52.3)	42.2 (31.3 to 55.8)
Motorcyclist road injuries	3 983 (3 033 to 5 098)	6 649 (4 793 to 9 132)	41.2 (32.6 to 51.0)	37.9 (27.3 to 52.0)
Motor vehicle road injuries	9 578 (7 585 to 11 805)	29 704 (23 988 to 36 063)	108.6 (87.3 to 131.3)	168.4 (136.8 to 203.4)
Other road injuries	5 871 (4 257 to 7 688)	8 193 (5 899 to 11 362)	65.8 (50.1 to 81.7)	48.5 (35.5 to 67.7)
Other transport injuries	1 408 (943 to 2 105)	1 486 (947 to 2 154)	14.1 (10.3 to 19.3)	9.0 (5.8 to 12.9)
**Unintentional injuries**	**309 531 (284 042 to 337 307)**	**591 419 (539 213 to 649 150)**	**3 667.7 (3 378.3 to 3 989.1)**	**3 685.2 (3 363.1 to 4 038.4)**
Falls	145 077 (126 303 to 165 684)	365 999 (323 946 to 413 096)	1 950.1 (1 743.0 to 2 194.3)	2 353.3 (2 092.4 to 2 644.2)
Drowning	408 (353 to 473)	294 (230 to 383)	4.2 (3.7 to 4.8)	1.7 (1.4 to 2.3)
Fire, heat, and hot substances	11 913 (9 888 to 15 307)	12 129 (9 872 to 14 877)	108.8 (84.0 to 128.8)	72.5 (58.9 to 89.2)
Poisonings	3 712 (2 679 to 5 203)	5 345 (3 820 to 7 292)	29.7 (22.0 to 40.6)	32.4 (23.0 to 44.2)
Poisoning by carbon monoxide	744 (457 to 1 170)	695 (389 to 1 124)	6.5 (4.3 to 9.6)	4.2 (2.4 to 6.7)
Poisoning by other means	2 968 (2 133 to 4 297)	4 650 (3 292 to 6 425)	23.2 (16.7 to 32.9)	28.2 (19.9 to 39.1)
Exposure to mechanical forces	68 139 (56 708 to 81 585)	83 594 (70 731 to 98 453)	679.0 (574.4 to 799.2)	476.7 (405.5 to 557.3)
Unintentional firearm injuries	5 047 (3 584 to 6 843)	6 697 (4 792 to 8 902)	48.2 (35.2 to 63.5)	37.8 (27.4 to 50.1)
Other exposure to mechanical forces	63 092 (52 520 to 76 350)	76 897 (64 668 to 90 569)	630.8 (535.0 to 743.2)	438.9 (372.0 to 515.9)
Adverse effects of medical treatment	7 836 (6 593 to 9 145)	20 156 (17 364 to 23 184)	100.1 (85.7 to 114.8)	122.1 (105.2 to 139.5)
Animal contact	21 310 (17 007 to 26 937)	24 322 (20 427 to 29 070)	223.1 (185.6 to 267.4)	147.4 (124.5 to 175.2)
Venomous animal contact	7 289 (5 363 to 9 884)	8 316 (6 395 to 10 615)	75.4 (56.9 to 97.4)	50.0 (38.6 to 63.9)
Non-venomous animal contact	14 022 (10 807 to 17 897)	16 007 (12 974 to 19 789)	147.8 (119.1 to 181.9)	97.3 (79.4 to 119.9)
Foreign body	25 782 (15 395 to 40 130)	46 781 (24 415 to 77 365)	307.4 (171.1 to 483.9)	282.7 (148.2 to 466.2)
Pulmonary aspiration and foreign body in airway	1 057 (757 to 1 415)	621 (445 to 857)	9.3 (7.1 to 12.1)	3.8 (2.7 to 5.2)
Foreign body in eyes	22 037 (11 783 to 36 021)	44 480 (22 161 to 75 140)	273.0 (136.8 to 453.3)	268.6 (134.8 to 450.6)
Foreign body in other body part	2 688 (1 804 to 4 154)	1 680 (1 224 to 2 248)	25.0 (17.4 to 35.6)	10.3 (7.5 to 13.6)
Environmental heat and cold exposure	6 321 (5 358 to 7 784)	11 537 (9 912 to 13 464)	67.0 (56.5 to 78.9)	70.3 (60.2 to 83.2)
Exposure to forces of nature	908 (754 to 1 105)	188 (158 to 225)	8.8 (7.4 to 10.4)	1.1 (0.9 to 1.3)
Other unintentional injuries	18 125 (14 832 to 22 240)	21 076 (17 808 to 25 014)	194.4 (164.2 to 229.3)	125.1 (105.7 to 147.9)

Prevalence

Cause	All-age counts (95% UI) 1990	All-age counts (95% UI) 2017	Age-standardised rates per 100,000 (95% UI) 1990	Age-standardised rates per 100,000 (95% UI) 2017
**Transport and unintentional injuries**	**651 718 (609 582 to 698 709)**	**1 402 299 (1 305 243 to 1 520 101)**	**9 314.8 (8 740.8 to 9 991.7)**	**8 689.0 (8 104.9 to 9 409.7)**
**Transport injuries**	**68 821 (64 224 to 73 561)**	**175 093 (163 005 to 188 057)**	**1 023.8 (957.6 to 1 095.2)**	**1 058.3 (986.6 to 1 135.8)**
Road injuries	62 255 (58 016 to 66 671)	167 213 (155 334 to 179 994)	941.9 (877.9 to 1 010.7)	1 010.4 (940.4 to 1 087.5)
Pedestrian road injuries	18 357 (15 709 to 21 623)	41 245 (35 730 to 48 359)	289.1 (251.0 to 336.5)	259.7 (220.8 to 295.0)
Cyclist road injuries	5 879 (4 932 to 6 948)	14 870 (12 500 to 17 585)	82.7 (70.3 to 95.6)	89.5 (76.0 to 105.2)
Motorcyclist road injuries	12 965 (11 213 to 15 058)	29 477 (25 152 to 34 912)	186.2 (161.4 to 214.7)	179.9 (148.8 to 205.5)
Motor vehicle road injuries	15 050 (13 159 to 17 651)	63 282 (55 057 to 74 414)	236.9 (205.4 to 277.2)	380.6 (330.8 to 447.4)
Other road injuries	10 003 (8 342 to 12 094)	18 339 (15 343 to 22 208)	146.9 (122.6 to 175.6)	112.6 (94.4 to 135.7)
Other transport injuries	6 566 (5 283 to 8 068)	7 881 (6 660 to 9 083)	81.9 (67.7 to 96.8)	47.9 (40.9 to 54.9)
**Unintentional injuries**	**582 897 (541 151 to 630 126)**	**1 227 206 (1 132 760 to 1 345 133)**	**8 291.0 (7 732.8 to 8 962.6)**	**7 630.8 (7 051.7 to 8 337.2)**
Falls	248 794 (218 324 to 282 756)	730 843 (647 166 to 831 144)	3 907.7 (3 490.1 to 4 463.9)	4 667.6 (4 162.5 to 5 323.2)
Drowning	1 766 (1 552 to 2 057)	1 773 (1 546 to 2 088)	26.3 (23.4 to 30.1)	11.0 (9.7 to 12.9)
Fire, heat, and hot substances	118 585 (93 184 to 144 865)	143 447 (117 027 to 186 291)	1 360.6 (1 149.2 to 1 684.4)	848.7 (691.0 to 1 082.6)
Poisonings	3 264 (2 464 to 4 037)	6 601 (4 831 to 8 395)	34.2 (26.1 to 42.0)	38.0 (27.8 to 48.3)
Poisoning by carbon monoxide	511 (391 to 666)	560 (429 to 727)	5.6 (4.3 to 7.1)	3.3 (2.5 to 4.2)
Poisoning by other means	2 753 (1 981 to 3 505)	6 041 (4 270 to 7 844)	28.6 (20.9 to 36.3)	34.7 (24.6 to 45.0)
Exposure to mechanical forces	124 704 (108 559 to 144 064)	204 187 (179 602 to 235 454)	1 757.9 (1 549.3 to 2 001.6)	1 215.2 (1 071.1 to 1 395.4)
Unintentional firearm injuries	17 143 (13 622 to 20 493)	29 644 (23 788 to 35 829)	245.2 (198.6 to 294.6)	177.5 (142.7 to 214.1)
Other exposure to mechanical forces	107 561 (90 509 to 127 052)	174 544 (147 884 to 205 902)	1 512.7 (1 295.7 to 1 765.9)	1 037.8 (884.9 to 1 222.1)
Adverse effects of medical treatment	600 (462 to 740)	1 543 (1 190 to 1 899)	7.7 (5.9 to 9.4)	9.3 (7.2 to 11.5)
Animal contact	21 958 (18 761 to 25 682)	30 720 (26 523 to 35 507)	296.1 (258.3 to 338.1)	187.0 (162.0 to 215.0)
Venomous animal contact	4 647 (3 795 to 5 502)	6 393 (5 356 to 7 526)	57.2 (47.3 to 66.7)	38.1 (32.1 to 44.6)
Non-venomous animal contact	17 310 (14 288 to 20 957)	24 327 (20 177 to 29 122)	238.9 (201.8 to 280.8)	149.0 (124.5 to 177.3)
Foreign body	13 388 (10 431 to 17 288)	15 895 (11 518 to 22 392)	170.8 (137.0 to 216.6)	99.5 (73.2 to 137.8)
Pulmonary aspiration and foreign body in airway	749 (538 to 1 016)	544 (438 to 674)	8.3 (6.2 to 10.9)	3.2 (2.6 to 4.0)
Foreign body in eyes	4 110 (2 010 to 7 290)	9 332 (5 141 to 15 639)	60.9 (34.8 to 98.3)	59.8 (33.9 to 95.8)
Foreign body in other body part	8 529 (6 717 to 10 941)	6 020 (5 036 to 7 205)	101.6 (82.2 to 128.1)	36.5 (30.7 to 43.5)
Environmental heat and cold exposure	21 656 (17 529 to 26 329)	44 520 (37 933 to 52 246)	280.5 (235.2 to 330.3)	272.7 (234.8 to 317.4)
Exposure to forces of nature	8 351 (5 900 to 11 767)	10 465 (7 378 to 14 623)	94.3 (67.8 to 131.2)	62.5 (43.8 to 87.5)
Other unintentional injuries	24 892 (21 201 to 28 977)	37 211 (32 208 to 42 727)	345.9 (299.1 to 395.2)	224.1 (194.6 to 255.8)

Years lived with disability

Cause	All-age counts (95% UI) 1990	All-age counts (95% UI) 2017	Age-standardised rates per 100,000 (95% UI) 1990	Age-standardised rates per 100,000 (95% UI) 2017
**Transport and unintentional injuries**	**29 054 (21 133 to 38 522)**	**60 532 (43 273 to 82 464)**	**409.8 (298.1 to 543.4)**	**375.4 (268.4 to 508.5)**
**Transport injuries**	**4 719 (3 440 to 6 265)**	**10 619 (7 623 to 14 255)**	**66.6 (48.8 to 88.1)**	**63.2 (45.4 to 84.9)**
Road injuries	4 293 (3 141 to 5 703)	10 201 (7 327 to 13 715)	61.6 (45.1 to 81.5)	60.7 (43.5 to 81.5)
Pedestrian road injuries	1 320 (939 to 1 786)	2 616 (1 826 to 3 576)	19.6 (14.1 to 26.6)	15.8 (11.1 to 21.7)
Cyclist road injuries	411 (288 to 545)	889 (616 to 1 181)	5.5 (3.9 to 7.3)	5.3 (3.7 to 7.0)
Motorcyclist road injuries	909 (639 to 1 224)	1 800 (1 282 to 2 462)	12.5 (8.7 to 16.9)	10.5 (7.2 to 14.4)
Motor vehicle road injuries	1 076 (776 to 1 432)	3 983 (2 869 to 5 346)	16.0 (11.7 to 21.1)	23.5 (17.0 to 31.4)
Other road injuries	576 (408 to 785)	912 (646 to 1 253)	8.0 (5.8 to 10.9)	5.5 (3.9 to 7.5)
Other transport injuries	427 (297 to 581)	418 (296 to 568)	5.0 (3.5 to 6.7)	2.5 (1.8 to 3.4)
**Unintentional injuries**	**24 334 (17 679 to 32 452)**	**49 913 (35 468 to 68 558)**	**343.2 (249.1 to 456.8)**	**312.3 (223.0 to 426.5)**
Falls	14 714 (10 825 to 19 427)	36 798 (26 461 to 49 617)	222.0 (161.4 to 290.6)	234.1 (166.7 to 314.4)
Drowning	101 (71 to 139)	94 (65 to 132)	1.4 (1.0 to 2.0)	0.6 (0.4 to 0.8)
Fire, heat, and hot substances	2 441 (1 481 to 3 840)	2 766 (1 600 to 4 538)	28.3 (17.4 to 44.4)	16.2 (9.4 to 26.5)
Poisonings	386 (234 to 586)	772 (465 to 1 197)	3.9 (2.4 to 5.9)	4.4 (2.7 to 6.8)
Poisoning by carbon monoxide	55 (33 to 88)	56 (33 to 89)	0.6 (0.3 to 0.9)	0.3 (0.2 to 0.5)
Poisoning by other means	331 (197 to 511)	716 (425 to 1 112)	3.3 (2.0 to 5.1)	4.1 (2.4 to 6.4)
Exposure to mechanical forces	2 939 (2 009 to 4 208)	4 382 (2 950 to 6 355)	40.3 (27.6 to 57.5)	26.0 (17.5 to 37.7)
Unintentional firearm injuries	358 (235 to 544)	577 (374 to 900)	4.9 (3.2 to 7.5)	3.4 (2.2 to 5.3)
Other exposure to mechanical forces	2 581 (1 763 to 3 697)	3 805 (2 554 to 5 572)	35.4 (24.3 to 50.7)	22.5 (15.2 to 32.9)
Adverse effects of medical treatment	80 (49 to 121)	206 (127 to 309)	1.0 (0.6 to 1.5)	1.2 (0.8 to 1.9)
Animal contact	589 (388 to 837)	735 (482 to 1 054)	7.1 (4.7 to 9.9)	4.4 (2.9 to 6.2)
Venomous animal contact	322 (201 to 479)	395 (252 to 576)	3.4 (2.2 to 5.0)	2.3 (1.5 to 3.3)
Non-venomous animal contact	267 (160 to 425)	340 (197 to 558)	3.6 (2.2 to 5.7)	2.1 (1.2 to 3.4)
Foreign body	833 (551 to 1 206)	929 (560 to 1 485)	10.3 (6.9 to 14.7)	5.8 (3.5 to 9.2)
Pulmonary aspiration and foreign body in airway	40 (27 to 55)	23 (16 to 31)	0.4 (0.3 to 0.6)	0.1 (0.1 to 0.2)
Foreign body in eyes	273 (133 to 508)	626 (327 to 1 118)	4.1 (2.2 to 7.2)	4.0 (2.1 to 7.0)
Foreign body in other body part	520 (355 to 696)	280 (199 to 372)	5.8 (4.1 to 7.7)	1.7 (1.2 to 2.2)
Environmental heat and cold exposure	833 (562 to 1 185)	1 648 (1 092 to 2 346)	10.8 (7.3 to 15.3)	10.1 (6.7 to 14.5)
Exposure to forces of nature	436 (316 to 607)	355 (264 to 482)	4.9 (3.5 to 6.7)	2.1 (1.6 to 2.8)
Other unintentional injuries	978 (703 to 1 303)	1 229 (862 to 1 692)	13.2 (9.5 to 17.7)	7.4 (5.2 to 10.2)

Disability adjusted life years

Cause	All-age counts (95% UI) 1990	All-age counts (95% UI) 2017	Age-standardised rates per 100,000 (95% UI) 1990	Age-standardised rates per 100,000 (95% UI) 2017
**Transport and unintentional injuries**	**287 983 (272 469 to 304 412)**	**278 112 (260 231 to 306 227)**	**2 994.6 (2 844.2 to 3 169.5)**	**1 640.6 (1 472.7 to 1 807.3)**
**Transport injuries**	**130 027 (122 488 to 137 342)**	**129 579 (113 694 to 143 064)**	**1 316.5 (1 247.1 to 1 382.4)**	**734.4 (645.6 to 809.5)**
Road injuries	127 013 (119 557 to 134 287)	125 193 (109 602 to 138 375)	1 284.8 (1 217.1 to 1 350.9)	709.7 (623.1 to 782.9)
Pedestrian road injuries	69 843 (65 068 to 74 938)	58 765 (52 158 to 64 921)	729.0 (687.4 to 771.2)	337.5 (300.6 to 371.5)
Cyclist road injuries	2 755 (2 485 to 3 049)	4 227 (3 653 to 4 773)	28.8 (26.1 to 31.7)	24.2 (20.9 to 27.8)
Motorcyclist road injuries	16 312 (14 646 to 18 068)	14 741 (12 693 to 16 846)	156.4 (141.5 to 171.4)	81.8 (70.5 to 93.5)
Motor vehicle road injuries	34 023 (30 786 to 37 315)	43 294 (36 346 to 48 914)	329.3 (301.4 to 357.5)	242.5 (203.9 to 274.0)
Other road injuries	4 080 (3 371 to 4 721)	4 166 (3 570 to 4 818)	41.3 (35.0 to 47.1)	23.8 (20.4 to 27.5)
Other transport injuries	3 014 (2 751 to 3 304)	4 386 (3 706 to 5 067)	31.7 (29.0 to 34.4)	24.7 (21.0 to 28.5)
**Unintentional injuries**	**157 955 (147 186 to 169 415)**	**148 533 (130 870 to 167 011)**	**1 678.1 (1 564.0 to 1 804.1)**	**906.2 (799.4 to 1 022.1)**
Falls	35 707 (31 460 to 40 450)	59 916 (49 279 to 71 839)	506.7 (446.2 to 573.7)	379.2 (312.8 to 454.6)
Drowning	29 868 (27 191 to 32 916)	15 004 (13 573 to 16 578)	268.1 (247.2 to 290.8)	86.4 (78.0 to 95.5)
Fire, heat, and hot substances	12 777 (11 335 to 14 556)	7 334 (6 072 to 9 123)	130.1 (116.4 to 148.6)	44.2 (36.7 to 54.6)
Poisonings	6 516 (5 959 to 7 108)	4 057 (3 616 to 4 602)	59.3 (54.8 to 64.1)	23.5 (21.0 to 26.6)
Poisoning by carbon monoxide	2 884 (2 609 to 3 166)	1 744 (1 568 to 1 946)	26.1 (23.9 to 28.4)	10.1 (9.1 to 11.2)
Poisoning by other means	3 632 (3 278 to 4 016)	2 312 (1 974 to 2 739)	33.1 (30.1 to 36.5)	13.4 (11.5 to 15.9)
Exposure to mechanical forces	18 353 (16 895 to 20 085)	12 827 (11 141 to 15 049)	193.0 (176.6 to 212.8)	73.9 (64.0 to 86.9)
Unintentional firearm injuries	7 331 (6 466 to 8 069)	3 483 (2 608 to 4 006)	70.4 (62.2 to 77.1)	19.6 (14.8 to 22.7)
Other exposure to mechanical forces	11 022 (9 834 to 12 415)	9 344 (7 845 to 11 269)	122.6 (109.1 to 139.8)	54.3 (45.5 to 65.6)
Adverse effects of medical treatment	4 626 (4 139 to 6 074)	5 841 (4 762 to 10 069)	53.1 (48.5 to 69.6)	36.9 (30.2 to 62.9)
Animal contact	3 943 (3 402 to 4 576)	1 843 (1 552 to 2 168)	36.5 (32.0 to 42.0)	11.1 (9.4 to 13.1)
Venomous animal contact	2 762 (2 306 to 3 286)	921 (715 to 1 151)	23.9 (20.4 to 28.0)	5.5 (4.3 to 6.9)
Non-venomous animal contact	1 181 (985 to 1 407)	922 (743 to 1 168)	12.6 (10.6 to 15.2)	5.6 (4.5 to 7.1)
Foreign body	33 187 (29 629 to 36 805)	31 628 (27 130 to 37 384)	299.1 (272.5 to 326.2)	193.0 (164.4 to 229.6)
Pulmonary aspiration and foreign body in airway	31 316 (27 913 to 34 978)	30 139 (25 626 to 35 881)	279.6 (253.2 to 307.1)	184.1 (155.0 to 221.2)
Foreign body in eyes	273 (133 to 508)	626 (327 to 1 118)	4.1 (2.2 to 7.2)	4.0 (2.1 to 7.0)
Foreign body in other body part	1 598 (1 363 to 1 915)	863 (674 to 1 448)	15.4 (13.1 to 18.4)	4.9 (3.9 to 8.4)
Environmental heat and cold exposure	2 531 (2 203 to 2 928)	2 435 (1 850 to 3 177)	30.5 (26.5 to 35.2)	14.7 (11.2 to 19.1)
Exposure to forces of nature	786 (658 to 954)	496 (399 to 623)	8.4 (7.0 to 10.2)	2.9 (2.4 to 3.6)
Other unintentional injuries	9 660 (9 031 to 10 374)	7 151 (6 402 to 7 986)	93.3 (87.4 to 100.0)	40.4 (36.1 to 45.0)
