# Supplementary material for: Unintentional injuries in Mexico, 1990–2017: findings from the Global Burden of Disease Study 2017
Source: Inj Prev. 2020 Apr 1;26(Suppl 1):i154–61. doi: 10.1136/injuryprev-2019-043532 (PMC7571365; doi:10.1136/injuryprev-2019-043532)
Supplement: Supplementary data [file injuryprev-2019-043532supp020.pdf]

Death, YLL, incidence, prevalence, YLD, and DALY counts and rates per 100,000 for all injuries in Michoacán de Ocampo in 1990 and 2017

Cause	Deaths				Years of life lost			
	All-age counts (95% UI)		Age-standardised rates per 100,000 (95% UI)		All-age counts (95% UI)		Age-standardised rates per 100,000 (95% UI)	
	1990	2017	1990	2017	1990	2017	1990	2017
**Transport and unintentional injuries**	**2 252 (2 128 to 2 389)**	**1 889 (1 769 to 2 013)**	**71.4 (69.0 to 74.3)**	**39.4 (36.8 to 42.0)**	**128 032 (118 261 to 139 464)**	**86 668 (80 363 to 93 635)**	**3 311.1 (3 120.3 to 3 528.3)**	**1 796.8 (1 666.4 to 1 939.7)**
**Transport injuries**	**999 (944 to 1 061)**	**897 (828 to 972)**	**31.1 (29.7 to 32.6)**	**18.7 (17.3 to 20.3)**	**57 398 (53 346 to 62 022)**	**45 254 (41 614 to 49 411)**	**1 533.8 (1 447.9 to 1 632.4)**	**935.4 (861.9 to 1 019.1)**
Road injuries	958 (914 to 1 029)	848 (784 to 919)	30.1 (28.8 to 31.6)	17.7 (16.3 to 19.2)	55 634 (51 674 to 60 234)	42 873 (39 423 to 46 739)	1 485.5 (1 401.4 to 1 582.1)	886.0 (815.0 to 965.6)
Pedestrian road injuries	495 (459 to 537)	372 (341 to 405)	15.9 (15.0 to 16.8)	7.8 (7.2 to 8.5)	27 864 (25 067 to 31 155)	17 160 (15 602 to 19 010)	725.9 (668.0 to 790.7)	357.3 (324.9 to 395.0)
Cyclist road injuries	21 (19 to 23)	31 (27 to 34)	0.6 (0.6 to 0.7)	0.6 (0.6 to 0.7)	1 184 (1 050 to 1 335)	1 466 (1 309 to 1 662)	33.0 (29.7 to 36.7)	30.5 (27.1 to 34.5)
Motorcyclist road injuries	113 (102 to 124)	108 (94 to 123)	3.4 (3.1 to 3.7)	2.2 (1.9 to 2.5)	6 740 (6 026 to 7 478)	6 212 (5 383 to 7 141)	185.4 (168.0 to 203.9)	126.9 (110.1 to 145.5)
Motor vehicle road injuries	310 (284 to 339)	314 (284 to 346)	9.3 (8.6 to 10.1)	6.5 (5.9 to 7.2)	18 181 (16 558 to 19 926)	16 830 (15 128 to 18 671)	496.9 (456.3 to 541.2)	346.5 (311.8 to 383.6)
Other road injuries	29 (25 to 33)	24 (21 to 28)	0.9 (0.8 to 1.0)	0.5 (0.4 to 0.6)	1 665 (1 371 to 1 948)	1 205 (1 033 to 1 416)	44.4 (37.8 to 51.2)	24.9 (21.3 to 29.3)
Other transport injuries	31 (28 to 35)	49 (42 to 55)	1.0 (0.9 to 1.1)	1.0 (0.9 to 1.1)	1 763 (1 574 to 1 970)	2 381 (2 027 to 2 719)	48.3 (43.4 to 53.6)	49.3 (42.0 to 56.4)
**Unintentional injuries**	**1 252 (1 173 to 1 343)**	**992 (928 to 1 067)**	**40.3 (38.7 to 42.1)**	**20.7 (19.3 to 22.0)**	**70 634 (64 027 to 78 149)**	**41 413 (37 993 to 45 439)**	**1 777.3 (1 652.1 to 1 918.4)**	**861.4 (790.3 to 944.4)**
Falls	246 (235 to 259)	303 (274 to 327)	11.0 (10.5 to 11.6)	6.3 (5.7 to 6.8)	8 058 (7 527 to 8 666)	7 147 (6 265 to 7 737)	273.0 (259.8 to 287.9)	151.1 (132.4 to 163.6)
Drowning	259 (233 to 290)	157 (142 to 171)	7.0 (6.5 to 7.7)	3.3 (3.0 to 3.6)	16 871 (14 772 to 19 416)	8 628 (7 706 to 9 656)	398.6 (353.9 to 445.3)	178.7 (160.0 to 199.7)
Fire, heat, and hot substances	93 (85 to 103)	60 (54 to 64)	3.1 (2.9 to 3.3)	1.2 (1.1 to 1.3)	5 186 (4 511 to 5 883)	2 062 (1 820 to 2 297)	127.4 (114.4 to 142.3)	43.1 (38.1 to 47.9)
Poisonings	49 (44 to 54)	29 (26 to 32)	1.4 (1.3 to 1.5)	0.6 (0.5 to 0.7)	3 150 (2 759 to 3 594)	1 484 (1 301 to 1 645)	74.7 (67.2 to 83.2)	30.8 (27.0 to 34.2)
Poisoning by carbon monoxide	14 (12 to 15)	10 (8 to 11)	0.4 (0.4 to 0.4)	0.2 (0.2 to 0.2)	850 (739 to 981)	475 (407 to 538)	20.9 (18.6 to 23.7)	9.8 (8.4 to 11.1)
Poisoning by other means	35 (31 to 39)	20 (17 to 22)	1.0 (0.9 to 1.0)	0.4 (0.4 to 0.5)	2 300 (2 001 to 2 658)	1 009 (882 to 1 137)	53.8 (48.1 to 60.4)	20.9 (18.3 to 23.6)
Exposure to mechanical forces	187 (176 to 199)	125 (114 to 138)	6.0 (5.7 to 6.3)	2.6 (2.4 to 2.9)	10 542 (9 801 to 11 304)	5 855 (5 265 to 6 511)	289.7 (271.7 to 307.5)	121.8 (109.5 to 135.4)
Unintentional firearm injuries	104 (95 to 114)	61 (49 to 70)	3.2 (2.9 to 3.5)	1.3 (1.0 to 1.5)	6 053 (5 445 to 6 601)	3 047 (2 449 to 3 556)	164.3 (149.4 to 179.3)	63.1 (50.8 to 73.6)
Other exposure to mechanical forces	83 (75 to 93)	65 (58 to 75)	2.8 (2.5 to 3.0)	1.4 (1.2 to 1.6)	4 489 (3 966 to 5 041)	2 809 (2 472 to 3 349)	125.4 (112.7 to 138.4)	58.7 (51.7 to 69.9)
Adverse effects of medical treatment	58 (48 to 64)	63 (57 to 79)	2.2 (1.8 to 2.4)	1.3 (1.2 to 1.7)	2 749 (2 191 to 3 159)	2 042 (1 793 to 2 620)	72.3 (58.8 to 80.5)	42.8 (37.7 to 55.0)
Animal contact	76 (64 to 91)	28 (25 to 30)	1.9 (1.7 to 2.2)	0.6 (0.5 to 0.6)	5 530 (4 503 to 6 743)	1 263 (1 103 to 1 464)	114.7 (95.4 to 136.9)	26.3 (23.1 to 30.5)
Venomous animal contact	65 (53 to 78)	19 (17 to 22)	1.5 (1.3 to 1.8)	0.4 (0.4 to 0.5)	4 842 (3 811 to 6 019)	903 (769 to 1 068)	97.8 (78.9 to 119.3)	18.8 (16.1 to 22.2)
Non-venomous animal contact	12 (8 to 16)	8 (7 to 10)	0.4 (0.3 to 0.4)	0.2 (0.2 to 0.2)	688 (466 to 1 001)	360 (305 to 430)	16.9 (11.9 to 22.8)	7.5 (6.4 to 9.0)
Foreign body	195 (173 to 221)	176 (157 to 200)	5.0 (4.6 to 5.5)	3.6 (3.3 to 4.1)	13 570 (11 672 to 15 756)	10 402 (8 847 to 12 439)	292.6 (258.3 to 332.3)	214.4 (182.8 to 256.1)
Pulmonary aspiration and foreign body in airway	186 (164 to 210)	172 (153 to 195)	4.8 (4.4 to 5.3)	3.6 (3.2 to 4.0)	12 927 (11 096 to 15 051)	10 166 (8 625 to 12 191)	277.4 (244.0 to 316.1)	209.5 (178.2 to 250.7)
Foreign body in eyes								
Foreign body in other body part	9 (7 to 11)	4 (3 to 6)	0.2 (0.2 to 0.3)	0.1 (0.1 to 0.1)	644 (454 to 779)	236 (197 to 329)	15.2 (11.0 to 17.8)	4.9 (4.1 to 6.8)
Environmental heat and cold exposure	22 (21 to 24)	11 (9 to 12)	0.8 (0.8 to 0.9)	0.2 (0.2 to 0.2)	1 054 (956 to 1 174)	395 (337 to 440)	30.4 (28.2 to 33.1)	8.4 (7.1 to 9.3)
Exposure to forces of nature	4 (4 to 5)	1 (1 to 1)	0.1 (0.1 to 0.2)	0.0 (0.0 to 0.0)	257 (214 to 308)	39 (36 to 43)	7.2 (6.0 to 8.8)	0.8 (0.7 to 0.9)
Other unintentional injuries	62 (58 to 67)	41 (36 to 46)	1.8 (1.7 to 2.0)	0.8 (0.8 to 0.9)	3 667 (3 368 to 3 980)	2 096 (1 858 to 2 361)	96.7 (90.1 to 104.4)	43.3 (38.5 to 48.8)

Cause	Incidence				Prevalence			
	All-age counts (95% UI)		Age-standardised rates per 100,000 (95% UI)		All-age counts (95% UI)		Age-standardised rates per 100,000 (95% UI)	
	1990	2017	1990	2017	1990	2017	1990	2017
**Transport and unintentional injuries**	**144 110 (132 060 to 156 598)**	**184 266 (166 923 to 202 563)**	**4 431.0 (4 051.8 to 4 829.1)**	**3 877.7 (3 509.4 to 4 267.9)**	**269 948 (253 694 to 289 131)**	**373 763 (350 031 to 404 330)**	**10 111.6 (9 535.9 to 10 828.3)**	**7 918.8 (7 414.0 to 8 574.2)**
**Transport injuries**	**11 449 (9 803 to 13 443)**	**19 176 (16 609 to 21 946)**	**344.9 (299.7 to 395.4)**	**400.5 (347.7 to 457.7)**	**27 975 (26 092 to 29 855)**	**56 034 (52 158 to 60 304)**	**1 085.1 (1 015.6 to 1 158.0)**	**1 191.2 (1 108.7 to 1 282.5)**
Road injuries	10 748 (9 146 to 12 722)	18 564 (15 934 to 21 360)	325.4 (281.1 to 375.7)	387.7 (333.7 to 445.7)	24 862 (23 131 to 26 626)	52 836 (49 079 to 56 981)	974.0 (907.3 to 1 043.4)	1 123.4 (1 043.6 to 1 212.0)
Pedestrian road injuries	2 055 (1 629 to 2 577)	2 377 (1 885 to 2 932)	63.2 (51.9 to 75.9)	50.0 (39.7 to 61.6)	7 903 (6 226 to 8 586)	11 031 (9 594 to 12 868)	283.6 (244.3 to 330.6)	234.7 (204.1 to 274.1)
Cyclist road injuries	1 418 (1 043 to 1 863)	2 116 (1 645 to 2 702)	39.4 (30.1 to 50.5)	44.2 (34.3 to 56.3)	2 271 (1 917 to 2 686)	4 687 (3 936 to 5 438)	84.6 (71.8 to 98.1)	98.1 (83.3 to 115.0)
Motorcyclist road injuries	1 398 (1 096 to 1 764)	1 950 (1 457 to 2 516)	41.1 (32.5 to 51.2)	40.2 (30.3 to 51.7)	4 976 (4 308 to 5 745)	9 109 (7 755 to 10 712)	194.2 (168.6 to 224.2)	193.1 (164.6 to 227.2)
Motor vehicle road injuries	3 748 (3 049 to 4 545)	9 303 (7 776 to 11 031)	118.4 (97.6 to 140.8)	194.2 (162.5 to 229.6)	6 565 (5 751 to 7 624)	21 364 (18 651 to 24 961)	267.5 (233.4 to 310.8)	455.3 (396.6 to 531.8)
Other road injuries	2 128 (1 600 to 2 702)	2 818 (2 228 to 3 451)	63.3 (50.0 to 77.4)	59.0 (46.7 to 72.2)	3 748 (3 117 to 4 483)	6 695 (5 623 to 8 048)	144.1 (120.6 to 172.3)	142.2 (119.5 to 171.0)
Other transport injuries	701 (523 to 958)	612 (446 to 814)	19.5 (15.6 to 24.9)	12.8 (9.3 to 17.0)	3 113 (2 586 to 3 668)	3 198 (2 729 to 3 687)	111.1 (93.9 to 128.6)	67.8 (57.9 to 78.2)
**Unintentional injuries**	**132 661 (121 083 to 145 469)**	**165 090 (147 738 to 183 363)**	**4 086.1 (3 717.7 to 4 484.8)**	**3 477.2 (3 113.1 to 3 866.2)**	**241 973 (226 112 to 260 333)**	**317 729 (294 675 to 348 283)**	**9 026.5 (8 441.2 to 9 710.0)**	**6 727.6 (6 234.0 to 7 379.8)**
Falls	54 973 (48 249 to 62 887)	100 146 (89 158 to 112 104)	1 833.0 (1 631.3 to 2 063.9)	2 112.2 (1 880.5 to 2 367.0)	94 092 (83 320 to 107 597)	194 076 (173 764 to 220 869)	3 680.8 (3 298.0 to 4 207.3)	4 105.3 (3 670.7 to 4 684.8)
Drowning	216 (185 to 250)	127 (108 to 152)	5.9 (5.2 to 6.8)	2.7 (2.2 to 3.4)	972 (852 to 1 119)	798 (706 to 918)	37.6 (33.2 to 43.3)	17.0 (15.0 to 19.5)
Fire, heat, and hot substances	5 097 (4 010 to 6 398)	2 547 (1 927 to 3 383)	116.5 (94.3 to 142.1)	53.2 (40.2 to 70.8)	46 936 (38 893 to 59 071)	30 049 (24 851 to 39 812)	1 560.2 (1 314.3 to 1 907.0)	633.3 (525.2 to 826.7)
Poisonings	1 493 (1 099 to 2 024)	1 025 (746 to 1 406)	30.8 (23.3 to 40.4)	21.3 (15.5 to 29.3)	1 229 (935 to 1 513)	1 147 (835 to 1 430)	36.1 (27.8 to 44.2)	24.0 (17.5 to 29.9)
Poisoning by carbon monoxide	286 (187 to 434)	202 (135 to 321)	6.6 (4.6 to 9.5)	4.2 (2.4 to 6.7)	185 (141 to 241)	150 (116 to 191)	5.6 (4.3 to 7.3)	3.2 (2.4 to 4.0)
Poisoning by other means	1 207 (863 to 1 671)	822 (573 to 1 156)	24.2 (17.8 to 32.8)	17.0 (11.9 to 24.0)	1 043 (752 to 1 321)	996 (700 to 1 275)	30.4 (22.2 to 38.4)	20.8 (14.7 to 26.7)
Exposure to mechanical forces	31 705 (27 045 to 37 275)	25 700 (21 661 to 30 404)	899.3 (773.9 to 1 042.8)	536.2 (452.0 to 634.6)	61 623 (54 265 to 70 479)	64 117 (56 102 to 73 755)	2 373.5 (2 090.5 to 2 697.4)	1 363.7 (1 191.9 to 1 568.8)
Unintentional firearm injuries	2 920 (2 163 to 3 814)	1 566 (1 147 to 2 114)	82.5 (62.2 to 105.9)	32.5 (23.8 to 43.7)	10 183 (8 122 to 12 280)	7 639 (6 158 to 9 235)	407.1 (323.5 to 485.2)	162.7 (131.2 to 196.9)
Other exposure to mechanical forces	28 784 (24 333 to 34 063)	24 133 (20 479 to 28 563)	816.8 (702.3 to 952.7)	503.7 (426.4 to 596.1)	51 440 (43 594 to 60 780)	56 478 (48 325 to 66 510)	1 971.4 (1 681.2 to 2 316.9)	1 201.0 (1 027.2 to 1 415.3)
Adverse effects of medical treatment	2 429 (2 024 to 2 848)	3 899 (3 301 to 4 652)	83.4 (70.6 to 97.1)	83.0 (70.2 to 99.2)	186 (144 to 232)	298 (228 to 377)	6.4 (4.9 to 7.9)	6.4 (4.9 to 8.0)
Animal contact	14 405 (11 922 to 17 470)	6 460 (5 268 to 7 908)	398.4 (340.5 to 466.6)	135.6 (110.7 to 166.1)	14 782 (12 685 to 17 296)	7 989 (6 994 to 9 142)	528.2 (459.0 to 601.8)	169.1 (147.9 to 193.4)
Venomous animal contact	5 407 (4 317 to 7 096)	2 296 (1 728 to 2 995)	148.8 (115.8 to 187.4)	48.3 (36.3 to 62.9)	3 618 (2 960 to 4 401)	1 702 (1 419 to 2 007)	120.8 (100.5 to 144.5)	35.9 (30.0 to 42.3)
Non-venomous animal contact	8 998 (7 257 to 11 136)	4 164 (3 249 to 5 230)	249.7 (206.4 to 301.4)	87.4 (68.4 to 110.2)	11 163 (9 093 to 13 581)	6 287 (5 315 to 7 436)	407.3 (341.4 to 482.0)	133.2 (112.7 to 157.5)
Foreign body	19 212 (16 673 to 20 648)	19 644 (17 748 to 33 409)	455.4 (201.5 to 746.0)	417.7 (169.0 to 709.7)	5 815 (4 450 to 7 713)	6 298 ([illegible] to 9 127)	208.9 (160.7 to 276.0)	133.8 (92.9 to 194.5)
Pulmonary aspiration and foreign body in airway	417 (287 to 585)	205 (149 to 280)	9.0 (6.5 to 12.3)	4.3 (3.1 to 5.8)	274 (195 to 360)	177 (144 to 217)	8.2 (6.0 to 10.9)	3.7 (3.0 to 4.6)
Foreign body in eyes	11 759 (5 196 to 19 165)	18 932 (6 843 to 32 704)	421.0 (165.3 to 714.6)	402.8 (144.2 to 695.4)	2 416 (1 252 to 4 096)	4 308 (2 395 to 7 145)	96.8 (54.4 to 158.0)	91.7 (50.9 to 152.0)
Foreign body in other body part	1 036 (697 to 1 569)	507 (358 to 687)	25.5 (18.0 to 35.8)	10.6 (7.5 to 14.4)	3 125 (2 494 to 3 951)	1 813 (1 521 to 2 187)	103.8 (84.7 to 129.0)	38.3 (32.1 to 46.3)
Environmental heat and cold exposure	1 778 (1 273 to 2 460)	692 (515 to 1 007)	50.6 (39.2 to 63.1)	14.5 (10.8 to 20.9)	6 017 (4 932 to 7 333)	2 656 (2 241 to 3 154)	214.1 (179.8 to 255.4)	56.0 (47.3 to 66.5)
Exposure to forces of nature	608 (502 to 730)	52 (44 to 62)	17.2 (14.1 to 20.6)	1.1 (0.9 to 1.3)	850 (618 to 1 141)	1 879 (1 332 to 2 584)	24.4 (18.0 to 32.4)	39.7 (28.1 to 54.4)
Other unintentional injuries	6 745 (5 514 to 8 268)	4 807 (3 735 to 6 298)	195.6 (164.4 to 233.0)	100.0 (77.5 to 131.1)	9 471 (8 182 to 10 925)	8 423 (7 255 to 9 656)	356.4 (308.6 to 408.4)	178.3 (153.5 to 204.3)

Cause	Years lived with disability				Disability adjusted life years			
	All-age counts (95% UI)		Age-standardised rates per 100,000 (95% UI)		All-age counts (95% UI)		Age-standardised rates per 100,000 (95% UI)	
	1990	2017	1990	2017	1990	2017	1990	2017
**Transport and unintentional injuries**	**11 719 (8 493 to 15 468)**	**16 665 (11 990 to 22 375)**	**433.2 (313.8 to 578.6)**	**352.8 (253.7 to 474.1)**	**139 750 (128 513 to 151 750)**	**103 333 (95 096 to 112 500)**	**3 744.3 (3 510.2 to 3 988.9)**	**2 149.6 (1 977.1 to 2 340.8)**
**Transport injuries**	**1 925 (1 410 to 2 542)**	**3 409 (2 474 to 4 535)**	**72.4 (53.1 to 96.0)**	**72.4 (52.6 to 96.3)**	**59 323 (55 238 to 64 017)**	**48 663 (44 770 to 52 738)**	**1 606.2 (1 518.8 to 1 715.3)**	**1 007.8 (927.5 to 1 090.7)**
Road injuries	1 724 (1 266 to 2 296)	3 237 (2 346 to 4 326)	65.5 (48.1 to 86.9)	68.7 (49.8 to 91.9)	57 358 (53 302 to 62 013)	46 110 (42 321 to 50 039)	1 551.1 (1 466.7 to 1 656.7)	954.8 (877.0 to 1 034.5)
Pedestrian road injuries	531 (375 to 723)	704 (496 to 960)	19.9 (14.1 to 26.7)	15.0 (10.6 to 20.4)	28 395 (25 594 to 31 699)	17 864 (16 238 to 19 649)	745.8 (686.8 to 811.0)	372.2 (338.8 to 409.3)
Cyclist road injuries	160 (114 to 211)	281 (196 to 374)	5.8 (4.1 to 7.6)	5.9 (4.2 to 7.9)	1 344 (1 210 to 1 501)	1 747 (1 551 to 1 958)	38.8 (35.3 to 42.7)	36.5 (32.4 to 40.7)
Motorcyclist road injuries	349 (243 to 470)	561 (385 to 766)	13.3 (9.2 to 17.9)	11.9 (8.1 to 16.2)	7 089 (6 382 to 7 820)	6 772 (5 901 to 7 702)	198.7 (180.2 to 217.4)	138.8 (121.1 to 157.4)
Motor vehicle road injuries	471 (342 to 626)	1 354 (979 to 1 807)	18.6 (13.4 to 24.6)	28.8 (20.7 to 38.5)	18 652 (17 028 to 20 447)	18 184 (16 525 to 20 095)	515.5 (474.5 to 561.7)	375.3 (341.4 to 414.2)
Other road injuries	214 (154 to 288)	337 (239 to 462)	8.0 (5.8 to 10.7)	7.2 (5.1 to 9.8)	1 879 (1 593 to 2 178)	1 543 (1 328 to 1 785)	52.4 (45.3 to 59.5)	32.1 (27.7 to 37.1)
Other transport injuries	201 (143 to 268)	172 (123 to 230)	6.9 (4.9 to 9.1)	3.6 (2.6 to 4.9)	1 965 (1 763 to 2 179)	2 553 (2 196 to 2 885)	55.1 (50.1 to 60.5)	53.0 (45.6 to 59.9)
**Unintentional injuries**	**9 794 (7 040 to 13 101)**	**13 256 (9 494 to 17 906)**	**360.8 (260.4 to 483.1)**	**280.4 (200.9 to 379.0)**	**80 428 (72 994 to 88 716)**	**54 670 (49 207 to 60 669)**	**2 138.1 (1 973.4 to 2 318.4)**	**1 141.8 (1 026.1 to 1 267.1)**
Falls	5 652 (4 098 to 7 396)	10 049 (7 285 to 13 376)	215.8 (156.6 to 280.8)	212.5 (154.0 to 283.0)	13 711 (11 941 to 15 521)	17 196 (14 213 to 20 501)	488.8 (427.0 to 556.7)	363.6 (300.4 to 433.3)
Drowning	55 (39 to 77)	42 (29 to 58)	2.1 (1.5 to 2.9)	0.9 (0.6 to 1.2)	16 927 (14 831 to 19 467)	8 670 (7 737 to 9 692)	400.7 (360.8 to 447.5)	179.6 (160.7 to 200.6)
Fire, heat, and hot substances	1 022 (639 to 1 606)	585 (349 to 958)	32.9 (20.5 to 52.0)	12.3 (7.3 to 20.1)	6 208 (5 382 to 7 198)	2 647 (2 330 to 3 054)	160.3 (141.3 to 182.1)	55.4 (48.4 to 64.0)
Poisonings	146 (89 to 221)	132 (78 to 206)	4.1 (2.5 to 6.2)	2.8 (1.6 to 4.3)	3 296 (2 917 to 3 746)	1 616 (1 424 to 1 795)	78.9 (71.2 to 87.7)	33.5 (29.6 to 37.3)
Poisoning by carbon monoxide	20 (12 to 32)	15 (9 to 23)	0.6 (0.3 to 0.9)	0.3 (0.2 to 0.5)	870 (760 to 1 001)	490 (422 to 554)	21.5 (19.2 to 24.2)	10.1 (8.8 to 11.5)
Poisoning by other means	126 (75 to 194)	117 (68 to 183)	3.6 (2.2 to 5.5)	2.5 (1.4 to 3.8)	2 426 (2 139 to 2 790)	1 126 (984 to 1 263)	57.4 (51.7 to 64.2)	23.4 (20.5 to 26.2)
Exposure to mechanical forces	1 462 (1 006 to 2 077)	1 393 (995 to 2 042)	55.3 (38.3 to 78.7)	29.6 (19.8 to 43.4)	12 003 (11 115 to 12 996)	7 248 (6 511 to 8 089)	345.0 (319.3 to 373.6)	151.4 (136.0 to 169.4)
Unintentional firearm injuries	218 (144 to 330)	144 (93 to 228)	8.4 (5.5 to 12.7)	3.1 (2.0 to 4.8)	6 271 (5 662 to 6 809)	3 190 (2 568 to 3 705)	172.7 (157.1 to 187.3)	66.1 (53.2 to 76.7)
Other exposure to mechanical forces	1 244 (854 to 1 767)	1 249 (843 to 1 826)	46.9 (32.2 to 67.5)	26.5 (17.9 to 38.9)	5 732 (5 056 to 6 498)	4 057 (3 486 to 4 831)	172.3 (151.5 to 197.5)	85.2 (73.3 to 101.6)
Adverse effects of medical treatment	25 (15 to 38)	40 (25 to 61)	0.9 (0.5 to 1.3)	0.8 (0.5 to 1.3)	2 774 (2 219 to 3 184)	2 082 (1 830 to 2 665)	73.1 (59.6 to 81.3)	43.7 (38.4 to 55.8)
Animal contact	400 (262 to 567)	185 (121 to 266)	13.0 (8.7 to 18.4)	3.9 (2.5 to 5.6)	5 931 (4 886 to 7 161)	1 448 (1 283 to 1 657)	127.7 (108.5 to 151.0)	30.2 (26.8 to 34.6)
Venomous animal contact	227 (144 to 335)	95 (58 to 142)	6.7 (4.3 to 9.8)	2.0 (1.2 to 3.0)	5 069 (4 022 to 6 252)	998 (860 to 1 173)	104.5 (85.1 to 126.9)	20.8 (18.0 to 24.4)
Non-venomous animal contact	173 (106 to 263)	90 (54 to 146)	6.2 (3.9 to 9.9)	1.9 (1.1 to 3.1)	862 (616 to 1 183)	449 (380 to 544)	23.2 (16.9 to 30.2)	9.4 (8.0 to 11.4)
Foreign body	369 (240 to 548)	382 (224 to 617)	13.1 (8.5 to 19.2)	8.1 (4.8 to 13.2)	13 940 (12 049 to 16 179)	10 784 (9 239 to 12 822)	305.6 (270.5 to 346.6)	222.5 (190.9 to 264.2)
Pulmonary aspiration and foreign body in airway	15 (10 to 21)	8 (5 to 10)	0.4 (0.3 to 0.6)	0.2 (0.1 to 0.2)	12 942 (11 111 to 15 065)	10 174 (8 633 to 12 199)	277.8 (244.4 to 316.5)	209.6 (178.4 to 250.9)
Foreign body in eyes	160 (82 to 286)	287 (150 to 502)	6.5 (3.5 to 11.4)	6.1 (3.2 to 10.7)	160 (82 to 285)	287 (150 to 502)	6.5 (3.5 to 11.4)	6.1 (3.2 to 10.7)
Foreign body in other body part	194 (136 to 258)	87 (62 to 115)	6.1 (4.4 to 8.1)	1.8 (1.3 to 2.4)	838 (644 to 984)	323 (272 to 419)	21.3 (16.8 to 24.7)	6.7 (5.7 to 8.7)
Environmental heat and cold exposure	240 (161 to 339)	101 (66 to 146)	8.5 (5.7 to 11.9)	2.1 (1.4 to 3.1)	1 294 (1 158 to 1 484)	497 (428 to 561)	38.9 (35.1 to 42.7)	10.5 (9.0 to 11.8)
Exposure to forces of nature	46 (32 to 64)	62 (45 to 86)	1.3 (0.9 to 1.8)	1.3 (0.9 to 1.8)	303 (255 to 355)	101 (84 to 125)	8.5 (7.2 to 10.1)	2.1 (1.8 to 2.6)
Other unintentional injuries	377 (271 to 500)	286 (202 to 392)	13.9 (9.9 to 18.5)	6.0 (4.3 to 8.3)	4 043 (3 727 to 4 388)	2 382 (2 136 to 2 675)	110.6 (102.2 to 119.7)	49.4 (44.3 to 55.4)
