# Supplementary material for: Unintentional injuries in Mexico, 1990–2017: findings from the Global Burden of Disease Study 2017
Source: Inj Prev. 2020 Apr 1;26(Suppl 1):i154–61. doi: 10.1136/injuryprev-2019-043532 (PMC7571365; doi:10.1136/injuryprev-2019-043532)
Supplement: Supplementary data [file injuryprev-2019-043532supp026.pdf]

Death, YLL, incidence, prevalence, YLD, and DALY counts and rates per 100,000 for all injuries in Morelos in 1990 and 2017

Cause	Deaths: All-age counts (95% UI) 1990	Deaths: All-age counts (95% UI) 2017	Deaths: Age-standardised rates per 100,000 (95% UI) 1990	Deaths: Age-standardised rates per 100,000 (95% UI) 2017	Years of life lost: All-age counts (95% UI) 1990	Years of life lost: All-age counts (95% UI) 2017	Years of life lost: Age-standardised rates per 100,000 (95% UI) 1990	Years of life lost: Age-standardised rates per 100,000 (95% UI) 2017
**Transport and unintentional injuries**	**652 (627 to 683)**	**785 (729 to 838)**	**63.6 (61.8 to 65.9)**	**39.6 (36.9 to 42.2)**	**34 560 (32 597 to 36 897)**	**32 850 (30 262 to 35 398)**	**2 760.0 (2 637.7 to 2 909.4)**	**1 630.1 (1 502.1 to 1 755.1)**
**Transport injuries**	**283 (268 to 300)**	**325 (288 to 358)**	**26.1 (24.9 to 27.4)**	**16.0 (14.2 to 17.6)**	**15 412 (14 496 to 16 498)**	**15 215 (13 442 to 16 931)**	**1 236.5 (1 171.7 to 1 315.2)**	**745.0 (659.4 to 828.4)**
Road injuries	275 (260 to 291)	309 (274 to 340)	25.3 (24.1 to 26.6)	15.3 (13.5 to 16.8)	14 973 (14 085 to 16 021)	14 464 (12 793 to 16 069)	1 203.0 (1 137.0 to 1 277.2)	708.4 (627.9 to 786.9)
Pedestrian road injuries	142 (133 to 152)	155 (140 to 172)	14.0 (13.3 to 14.8)	7.7 (7.0 to 8.6)	7 380 (6 894 to 8 040)	6 515 (5 841 to 7 250)	604.2 (566.9 to 647.6)	321.3 (288.4 to 357.5)
Cyclist road injuries	5 (4 to 5)	10 (8 to 11)	0.4 (0.4 to 0.5)	0.5 (0.4 to 0.5)	269 (239 to 304)	430 (373 to 495)	21.9 (19.7 to 24.3)	21.0 (18.2 to 24.2)
Motorcyclist road injuries	39 (35 to 43)	38 (32 to 44)	3.2 (2.9 to 3.6)	1.8 (1.6 to 2.1)	2 271 (2 025 to 2 559)	2 101 (1 765 to 2 484)	176.7 (159.1 to 197.2)	101.9 (85.7 to 120.3)
Motor vehicle road injuries	83 (76 to 91)	100 (83 to 113)	7.1 (6.5 to 7.7)	4.9 (4.1 to 5.5)	4 713 (4 286 to 5 233)	5 075 (4 230 to 5 828)	371.5 (339.7 to 409.0)	247.4 (207.0 to 283.5)
Other road injuries	6 (5 to 7)	7 (6 to 8)	0.5 (0.5 to 0.6)	0.4 (0.3 to 0.4)	340 (283 to 402)	343 (286 to 407)	26.7 (22.6 to 31.4)	16.7 (14.0 to 19.8)
Other transport injuries	8 (7 to 9)	16 (13 to 19)	0.8 (0.7 to 0.8)	0.8 (0.6 to 0.9)	439 (393 to 498)	751 (611 to 888)	35.5 (32.0 to 39.8)	36.6 (29.9 to 43.4)
**Unintentional injuries**	**369 (352 to 389)**	**460 (434 to 486)**	**37.6 (36.3 to 39.0)**	**23.6 (22.3 to 24.9)**	**19 148 (17 741 to 20 696)**	**17 635 (16 589 to 18 797)**	**1 523.5 (1 437.4 to 1 618.7)**	**885.1 (831.3 to 946.5)**
Falls	90 (85 to 94)	140 (126 to 152)	12.1 (11.5 to 12.8)	7.4 (6.7 to 8.0)	2 921 (2 747 to 3 118)	3 247 (2 898 to 3 542)	299.5 (284.5 to 315.7)	163.3 (146.1 to 177.8)
Drowning	76 (70 to 83)	55 (50 to 60)	6.3 (5.9 to 6.8)	2.7 (2.5 to 3.0)	4 698 (4 249 to 5 280)	2 740 (2 484 to 3 034)	342.8 (315.2 to 375.6)	136.5 (123.3 to 151.3)
Fire, heat, and hot substances	25 (23 to 27)	24 (22 to 26)	2.6 (2.5 to 2.8)	1.2 (1.1 to 1.3)	1 254 (1 117 to 1 416)	741 (663 to 808)	99.2 (90.4 to 109.0)	37.3 (33.4 to 40.7)
Poisonings	15 (14 to 17)	13 (11 to 14)	1.3 (1.2 to 1.4)	0.6 (0.6 to 0.7)	931 (837 to 1 039)	600 (531 to 667)	68.8 (62.6 to 75.7)	29.8 (26.4 to 33.2)
Poisoning by carbon monoxide	3 (3 to 4)	3 (3 to 4)	0.3 (0.3 to 0.3)	0.2 (0.1 to 0.2)	199 (175 to 227)	156 (133 to 180)	15.0 (13.3 to 17.0)	7.7 (6.6 to 8.9)
Poisoning by other means	12 (11 to 13)	10 (8 to 11)	1.0 (0.9 to 1.1)	0.5 (0.4 to 0.5)	732 (657 to 824)	444 (392 to 495)	53.8 (48.9 to 59.5)	22.1 (19.6 to 24.7)
Exposure to mechanical forces	54 (51 to 58)	41 (37 to 45)	5.1 (4.8 to 5.4)	2.0 (1.8 to 2.2)	2 897 (2 675 to 3 129)	1 759 (1 587 to 1 955)	238.0 (221.6 to 255.1)	86.1 (77.9 to 95.6)
Unintentional firearm injuries	28 (26 to 32)	16 (13 to 19)	2.5 (2.3 to 2.9)	0.8 (0.6 to 0.9)	1 561 (1 406 to 1 752)	778 (621 to 897)	126.2 (114.3 to 142.3)	37.9 (30.3 to 43.6)
Other exposure to mechanical forces	26 (22 to 28)	24 (22 to 28)	2.6 (2.2 to 2.8)	1.2 (1.1 to 1.4)	1 336 (1 141 to 1 494)	981 (867 to 1 148)	111.9 (95.4 to 123.8)	48.2 (42.7 to 56.1)
Adverse effects of medical treatment	19 (16 to 21)	30 (27 to 33)	2.2 (1.9 to 2.4)	1.6 (1.4 to 2.0)	892 (686 to 950)	859 (755 to 1 159)	70.1 (59.0 to 77.8)	43.9 (38.4 to 59.3)
Animal contact	18 (15 to 20)	11 (10 to 12)	1.5 (1.3 to 1.7)	0.6 (0.5 to 0.6)	1 142 (951 to 1 381)	445 (398 to 496)	79.2 (67.8 to 93.6)	22.7 (20.2 to 25.3)
Venomous animal contact	14 (12 to 17)	8 (7 to 9)	1.2 (1.0 to 1.4)	0.4 (0.4 to 0.5)	972 (782 to 1 205)	322 (282 to 367)	66.0 (54.6 to 80.1)	16.5 (14.4 to 18.9)
Non-venomous animal contact	3 (2 to 4)	3 (3 to 4)	0.3 (0.2 to 0.4)	0.2 (0.1 to 0.2)	169 (118 to 220)	123 (105 to 149)	13.2 (9.1 to 16.4)	6.2 (5.2 to 7.5)
Foreign body	50 (45 to 55)	55 (50 to 61)	4.2 (3.8 to 4.5)	2.8 (2.6 to 3.1)	3 239 (2 826 to 3 713)	2 680 (2 355 to 3 068)	226.0 (201.0 to 254.3)	137.9 (120.5 to 159.7)
Pulmonary aspiration and foreign body in airway	47 (42 to 53)	54 (49 to 59)	4.0 (3.6 to 4.3)	2.8 (2.5 to 3.0)	3 085 (2 680 to 3 548)	2 602 (2 282 to 2 980)	214.9 (190.4 to 242.3)	134.0 (116.9 to 155.2)
Foreign body in eyes								
Foreign body in other body part	2 (2 to 3)	1 (1 to 2)	0.2 (0.1 to 0.2)	0.1 (0.1 to 0.1)	154 (116 to 182)	79 (65 to 123)	11.1 (8.5 to 12.9)	3.9 (3.2 to 6.2)
Environmental heat and cold exposure	6 (6 to 6)	4 (4 to 5)	0.7 (0.7 to 0.7)	0.2 (0.2 to 0.2)	267 (245 to 290)	151 (128 to 166)	24.1 (22.4 to 25.9)	7.4 (6.3 to 8.1)
Exposure to forces of nature	2 (1 to 2)	71 (66 to 77)	0.1 (0.1 to 0.2)	3.6 (3.3 to 3.9)	92 (72 to 121)	3 680 (3 356 to 4 043)	7.4 (5.8 to 10.0)	184.4 (168.2 to 202.9)
Other unintentional injuries	15 (14 to 17)	15 (13 to 17)	1.3 (1.2 to 1.5)	0.7 (0.6 to 0.8)	881 (802 to 960)	732 (637 to 841)	68.5 (62.7 to 75.3)	35.7 (31.0 to 40.9)

Cause	Incidence: All-age counts (95% UI) 1990	Incidence: All-age counts (95% UI) 2017	Incidence: Age-standardised rates per 100,000 (95% UI) 1990	Incidence: Age-standardised rates per 100,000 (95% UI) 2017	Prevalence: All-age counts (95% UI) 1990	Prevalence: All-age counts (95% UI) 2017	Prevalence: Age-standardised rates per 100,000 (95% UI) 1990	Prevalence: Age-standardised rates per 100,000 (95% UI) 2017
**Transport and unintentional injuries**	**45 856 (42 701 to 49 103)**	**76 146 (70 448 to 82 101)**	**4 122.1 (3 851.4 to 4 408.6)**	**3 856.7 (3 571.8 to 4 163.5)**	**89 969 (84 481 to 96 258)**	**155 480 (145 558 to 168 245)**	**9 629.5 (9 065.9 to 10 320.6)**	**7 752.5 (7 259.1 to 8 387.8)**
**Transport injuries**	**3 737 (3 207 to 4 336)**	**7 613 (6 554 to 8 772)**	**326.7 (282.8 to 373.4)**	**375.8 (324.3 to 433.0)**	**9 221 (8 601 to 9 816)**	**22 339 (20 857 to 23 937)**	**1 011.5 (947.2 to 1 079.9)**	**1 099.7 (1 027.0 to 1 178.5)**
Road injuries	3 529 (3 012 to 4 127)	7 390 (6 328 to 8 529)	310.1 (265.4 to 355.5)	364.5 (313.1 to 420.8)	8 261 (7 682 to 8 844)	21 165 (19 661 to 22 721)	918.3 (856.3 to 983.6)	1 041.5 (968.1 to 1 118.5)
Pedestrian road injuries	658 (532 to 815)	1 008 (788 to 1 244)	61.1 (50.6 to 73.8)	50.0 (39.2 to 61.5)	2 375 (2 097 to 2 802)	4 606 (4 021 to 5 388)	268.5 (234.1 to 312.6)	228.0 (199.3 to 266.1)
Cyclist road injuries	503 (377 to 654)	998 (777 to 1 276)	41.2 (32.1 to 52.7)	49.9 (38.7 to 64.0)	824 (693 to 976)	2 191 (1 859 to 2 564)	86.8 (73.4 to 100.9)	108.1 (92.0 to 126.2)
Motorcyclist road injuries	512 (403 to 647)	844 (684 to 1 075)	42.5 (33.8 to 52.6)	41.4 (31.3 to 52.8)	1 799 (1 554 to 2 079)	3 896 (3 327 to 4 570)	195.2 (168.5 to 225.2)	190.4 (162.8 to 223.5)
Motor vehicle road injuries	1 181 (946 to 1 435)	3 235 (2 617 to 3 898)	106.1 (85.8 to 127.8)	158.5 (128.3 to 191.0)	2 041 (1 784 to 2 380)	7 391 (6 428 to 8 669)	234.9 (203.9 to 274.5)	362.9 (315.6 to 425.2)
Other road injuries	677 (513 to 860)	1 305 (1 022 to 1 596)	59.2 (46.8 to 72.2)	64.7 (50.9 to 79.0)	1 222 (1 015 to 1 471)	3 080 (2 562 to 3 728)	133.1 (110.7 to 159.7)	152.0 (126.6 to 183.6)
Other transport injuries	207 (151 to 286)	223 (169 to 292)	16.6 (12.9 to 21.4)	11.3 (8.5 to 14.8)	960 (793 to 1 151)	1 174 (1 009 to 1 347)	95.2 (80.1 to 111.4)	58.2 (50.2 to 66.8)
**Unintentional injuries**	**42 119 (38 939 to 45 519)**	**68 534 (63 390 to 74 491)**	**3 795.4 (3 521.3 to 4 082.4)**	**3 480.9 (3 215.0 to 3 783.6)**	**80 748 (75 377 to 86 945)**	**133 140 (123 442 to 145 611)**	**8 616.0 (8 056.2 to 9 278.1)**	**6 652.8 (6 170.6 to 7 286.2)**
Falls	18 793 (16 480 to 21 329)	39 092 (34 739 to 44 296)	1 867.9 (1 662.1 to 2 099.2)	2 006.8 (1 781.7 to 2 272.5)	32 966 (29 110 to 37 645)	76 228 (68 022 to 86 922)	3 741.4 (3 339.1 to 4 292.9)	3 845.3 (3 437.7 to 4 380.5)
Drowning	68 (59 to 79)	47 (38 to 58)	5.5 (4.8 to 6.3)	2.3 (1.9 to 2.9)	315 (276 to 363)	295 (260 to 339)	34.4 (30.3 to 39.2)	14.7 (12.9 to 16.8)
Fire, heat, and hot substances	1 506 (1 199 to 1 903)	1 280 (957 to 1 596)	105.4 (86.1 to 129.8)	63.7 (49.4 to 83.6)	14 700 (12 249 to 18 494)	15 222 (12 496 to 20 041)	1 391.9 (1 173.9 to 1 712.6)	751.5 (617.0 to 991.0)
Poisonings	510 (373 to 713)	454 (320 to 646)	32.9 (24.6 to 45.0)	23.8 (16.7 to 34.0)	453 (338 to 559)	541 (400 to 677)	38.3 (28.9 to 47.0)	26.7 (19.7 to 33.5)
Poisoning by carbon monoxide	107 (66 to 165)	117 (63 to 195)	7.5 (5.0 to 10.9)	6.0 (3.2 to 10.2)	75 (58 to 98)	96 (74 to 123)	6.5 (5.1 to 8.4)	4.8 (3.7 to 6.1)
Poisoning by other means	403 (288 to 577)	337 (221 to 498)	25.4 (18.4 to 35.5)	17.8 (11.7 to 26.4)	378 (270 to 482)	444 (313 to 573)	31.8 (22.8 to 40.5)	22.0 (15.5 to 28.3)
Exposure to mechanical forces	10 277 (8 786 to 12 125)	10 977 (9 404 to 12 803)	841.2 (724.0 to 977.0)	544.1 (467.4 to 634.9)	20 285 (17 869 to 23 272)	27 826 (24 419 to 32 010)	2 193.5 (1 939.0 to 2 504.9)	1 367.3 (1 200.2 to 1 571.4)
Unintentional firearm injuries	891 (657 to 1 177)	546 (398 to 726)	71.8 (54.1 to 93.7)	27.0 (19.7 to 35.8)	3 191 (2 567 to 3 855)	2 800 (2 262 to 3 385)	351.6 (284.3 to 426.8)	137.6 (111.4 to 166.2)
Other exposure to mechanical forces	9 386 (7 999 to 11 108)	10 431 (8 904 to 12 157)	769.4 (662.6 to 895.2)	517.1 (442.4 to 603.0)	17 094 (14 535 to 20 272)	25 026 (21 613 to 29 252)	1 841.9 (1 578.2 to 2 174.4)	1 229.8 (1 068.2 to 1 437.0)
Adverse effects of medical treatment	875 (725 to 1 039)	1 712 (1 421 to 2 038)	86.7 (72.9 to 102.8)	85.1 (70.7 to 104.5)	67 (51 to 84)	131 (99 to 170)	6.6 (5.1 to 8.3)	6.5 (4.9 to 8.5)
Animal contact	4 493 (3 744 to 5 462)	2 858 (2 330 to 3 488)	374.2 (320.4 to 436.8)	145.0 (117.0 to 177.0)	4 741 (4 096 to 5 494)	3 570 (3 137 to 4 061)	487.4 (428.9 to 553.4)	177.6 (155.1 to 201.9)
Venomous animal contact	1 789 (1 379 to 2 323)	1 031 (769 to 1 357)	148.3 (115.8 to 186.7)	52.1 (38.6 to 68.3)	1 247 (1 029 to 1 504)	736 (621 to 861)	121.3 (101.3 to 143.3)	36.6 (30.9 to 42.9)
Non-venomous animal contact	2 704 (2 166 to 3 389)	1 828 (1 435 to 2 310)	225.8 (185.2 to 274.9)	93.0 (72.7 to 117.3)	3 494 (2 868 to 4 217)	2 834 (2 394 to 3 315)	366.1 (307.6 to 430.9)	141.0 (119.2 to 164.8)
Foreign body	2 818 (1 921 to 3 858)	4 443 (2 839 to 6 246)	246.5 (165.8 to 346.2)	222.6 (144.1 to 312.3)	1 820 (1 468 to 2 272)	1 861 (1 460 to 2 381)	174.5 (143.5 to 214.1)	93.1 (72.9 to 118.4)
Pulmonary aspiration and foreign body in airway	135 (97 to 187)	74 (55 to 98)	9.1 (6.7 to 12.2)	3.8 (2.8 to 5.1)	97 (68 to 134)	65 (53 to 79)	8.4 (6.0 to 11.3)	3.2 (2.6 to 3.9)
Foreign body in eyes	2 285 (1 411 to 3 354)	4 122 (2 530 to 5 949)	208.2 (129.8 to 307.7)	206.2 (128.6 to 297.0)	443 (287 to 704)	903 (528 to 1 418)	46.2 (27.6 to 70.9)	45.5 (26.6 to 71.3)
Foreign body in other body part	398 (260 to 619)	247 (157 to 350)	29.2 (19.8 to 42.7)	12.5 (8.0 to 17.9)	1 280 (1 012 to 1 652)	893 (754 to 1 063)	119.9 (96.3 to 150.8)	44.3 (37.5 to 52.6)
Environmental heat and cold exposure	589 (411 to 797)	426 (299 to 619)	49.3 (37.4 to 62.6)	21.8 (15.5 to 31.9)	2 118 (1 714 to 2 584)	1 736 (1 454 to 2 075)	212.1 (176.5 to 252.9)	86.6 (72.7 to 103.6)
Exposure to forces of nature	229 (178 to 292)	4 896 (4 132 to 5 878)	18.5 (14.2 to 23.5)	245.6 (207.2 to 295.9)	323 (235 to 483)	1 502 (1 140 to 1 971)	27.7 (20.6 to 37.0)	74.1 (56.3 to 97.3)
Other unintentional injuries	2 021 (1 652 to 2 483)	2 389 (2 000 to 2 918)	167.5 (139.6 to 201.7)	120.0 (100.1 to 146.5)	2 959 (2 521 to 3 424)	4 228 (3 686 to 4 819)	303.1 (266.1 to 352.4)	209.2 (182.5 to 237.6)

Cause	Years lived with disability: All-age counts (95% UI) 1990	Years lived with disability: All-age counts (95% UI) 2017	Years lived with disability: Age-standardised rates per 100,000 (95% UI) 1990	Years lived with disability: Age-standardised rates per 100,000 (95% UI) 2017	Disability adjusted life years: All-age counts (95% UI) 1990	Disability adjusted life years: All-age counts (95% UI) 2017	Disability adjusted life years: Age-standardised rates per 100,000 (95% UI) 1990	Disability adjusted life years: Age-standardised rates per 100,000 (95% UI) 2017
**Transport and unintentional injuries**	**3 887 (2 830 to 5 157)**	**6 662 (4 791 to 8 946)**	**411.1 (299.7 to 545.4)**	**332.7 (239.3 to 446.7)**	**38 447 (36 308 to 40 947)**	**39 513 (36 416 to 42 878)**	**3 171.1 (3 007.9 to 3 354.7)**	**1 962.8 (1 809.3 to 2 131.3)**
**Transport injuries**	**625 (454 to 828)**	**1 335 (974 to 1 778)**	**66.4 (48.5 to 88.0)**	**65.5 (47.8 to 87.1)**	**16 036 (15 102 to 17 138)**	**16 550 (14 682 to 18 236)**	**1 302.8 (1 234.7 to 1 379.9)**	**810.5 (720.6 to 893.2)**
Road injuries	563 (403 to 748)	1 274 (927 to 1 702)	60.6 (44.3 to 80.3)	62.5 (45.4 to 83.5)	15 536 (14 642 to 16 608)	15 738 (13 974 to 17 331)	1 263.6 (1 196.1 to 1 336.6)	770.8 (685.5 to 848.9)
Pedestrian road injuries	169 (119 to 229)	290 (203 to 400)	18.4 (13.1 to 24.9)	14.3 (10.0 to 19.7)	7 550 (6 994 to 8 225)	6 805 (6 115 to 7 532)	622.7 (584.2 to 667.0)	335.6 (302.0 to 371.8)
Cyclist road injuries	57 (40 to 75)	130 (92 to 173)	5.8 (4.1 to 7.6)	6.4 (4.5 to 8.5)	326 (293 to 365)	560 (493 to 636)	27.7 (25.0 to 31.0)	27.4 (24.0 to 31.0)
Motorcyclist road injuries	124 (88 to 169)	237 (162 to 324)	13.1 (9.2 to 17.7)	11.6 (7.9 to 15.8)	2 395 (2 140 to 2 677)	2 338 (2 004 to 2 733)	189.8 (170.6 to 210.1)	113.4 (97.3 to 132.4)
Motor vehicle road injuries	144 (104 to 192)	462 (333 to 622)	16.0 (11.6 to 21.1)	22.6 (16.3 to 30.4)	4 857 (4 404 to 5 400)	5 537 (4 682 to 6 290)	387.4 (355.1 to 425.4)	270.0 (228.9 to 305.9)
Other road injuries	69 (50 to 95)	155 (110 to 213)	7.3 (5.3 to 9.8)	7.6 (5.4 to 10.5)	409 (349 to 477)	497 (424 to 588)	34.0 (29.3 to 39.2)	24.4 (20.7 to 28.7)
Other transport injuries	61 (43 to 81)	62 (44 to 82)	5.8 (4.1 to 7.7)	3.0 (2.2 to 4.1)	500 (448 to 563)	813 (671 to 950)	41.3 (37.2 to 45.9)	39.7 (32.9 to 46.3)
**Unintentional injuries**	**3 261 (2 347 to 4 318)**	**5 327 (3 799 to 7 171)**	**344.8 (247.4 to 460.4)**	**267.2 (190.6 to 359.6)**	**22 411 (20 720 to 24 190)**	**22 962 (21 013 to 25 069)**	**1 868.3 (1 739.5 to 2 008.2)**	**1 152.3 (1 053.7 to 1 260.8)**
Falls	1 939 (1 426 to 2 542)	3 864 (2 763 to 5 099)	214.5 (156.6 to 281.1)	194.9 (139.2 to 258.9)	4 860 (4 316 to 5 502)	7 110 (6 004 to 8 419)	514.0 (453.9 to 582.6)	358.2 (302.5 to 423.8)
Drowning	18 (12 to 25)	15 (11 to 21)	1.9 (1.3 to 2.6)	0.8 (0.5 to 1.0)	4 711 (4 264 to 5 248)	2 756 (2 498 to 3 050)	344.7 (317.3 to 377.5)	137.2 (124.3 to 152.1)
Fire, heat, and hot substances	314 (194 to 494)	293 (170 to 477)	28.9 (17.8 to 45.3)	14.5 (8.4 to 23.6)	1 569 (1 384 to 1 813)	1 034 (830 to 1 226)	128.1 (114.4 to 146.8)	51.8 (44.6 to 61.2)
Poisonings	54 (33 to 80)	62 (37 to 96)	4.4 (2.7 to 6.6)	3.1 (1.8 to 4.7)	985 (890 to 1 098)	662 (590 to 735)	73.2 (66.8 to 80.5)	32.9 (29.3 to 36.5)
Poisoning by carbon monoxide	8 (5 to 13)	10 (6 to 15)	0.7 (0.4 to 1.1)	0.5 (0.3 to 0.7)	207 (182 to 236)	165 (143 to 190)	15.7 (13.9 to 17.8)	8.2 (7.1 to 9.4)
Poisoning by other means	45 (27 to 70)	52 (31 to 82)	3.7 (2.2 to 5.7)	2.6 (1.5 to 4.0)	777 (696 to 872)	497 (442 to 553)	57.5 (52.0 to 63.6)	24.7 (22.0 to 27.6)
Exposure to mechanical forces	476 (329 to 680)	596 (399 to 869)	50.5 (34.9 to 72.2)	29.3 (19.6 to 42.6)	3 373 (3 108 to 3 665)	2 355 (2 084 to 2 694)	288.5 (265.8 to 314.2)	115.4 (102.1 to 131.8)
Unintentional firearm injuries	67 (44 to 101)	52 (33 to 82)	7.2 (4.7 to 10.9)	2.5 (1.6 to 4.0)	1 628 (1 475 to 1 813)	829 (666 to 953)	133.3 (121.3 to 148.4)	40.4 (32.5 to 46.3)
Other exposure to mechanical forces	409 (280 to 587)	545 (365 to 794)	43.3 (29.8 to 62.5)	26.8 (17.9 to 39.0)	1 745 (1 517 to 1 973)	1 526 (1 302 to 1 822)	155.2 (134.7 to 177.4)	75.0 (64.1 to 89.5)
Adverse effects of medical treatment	9 (5 to 14)	17 (11 to 27)	0.9 (0.5 to 1.3)	0.9 (0.5 to 1.3)	841 (695 to 961)	877 (772 to 1 178)	70.9 (59.8 to 78.7)	44.8 (39.3 to 60.9)
Animal contact	127 (84 to 178)	80 (53 to 114)	11.8 (8.0 to 16.7)	3.9 (2.6 to 5.7)	1 268 (1 070 to 1 518)	525 (473 to 584)	91.0 (79.0 to 105.9)	26.6 (23.8 to 29.7)
Venomous animal contact	73 (47 to 106)	39 (25 to 58)	6.3 (4.1 to 9.0)	2.0 (1.2 to 2.9)	1 046 (848 to 1 279)	362 (317 to 410)	72.3 (60.2 to 86.3)	18.5 (16.2 to 21.0)
Non-venomous animal contact	53 (32 to 86)	40 (24 to 64)	5.5 (3.4 to 8.7)	2.0 (1.2 to 3.2)	222 (160 to 288)	164 (138 to 201)	18.7 (13.6 to 23.5)	8.2 (6.9 to 10.1)
Foreign body	112 (77 to 157)	105 (67 to 156)	10.4 (7.3 to 14.4)	5.2 (3.4 to 7.8)	3 351 (2 934 to 3 830)	2 785 (2 456 to 3 169)	236.4 (211.5 to 265.4)	143.2 (125.9 to 164.7)
Pulmonary aspiration and foreign body in airway	5 (3 to 7)	3 (2 to 4)	0.4 (0.3 to 0.6)	0.1 (0.1 to 0.2)	3 091 (2 686 to 3 553)	2 604 (2 284 to 2 982)	215.3 (190.8 to 243.2)	134.2 (117.0 to 155.4)
Foreign body in eyes	30 (15 to 51)	61 (34 to 99)	3.1 (1.7 to 5.1)	3.1 (1.7 to 5.0)	30 (15 to 51)	61 (34 to 99)	3.1 (1.7 to 5.1)	3.1 (1.7 to 5.0)
Foreign body in other body part	77 (54 to 104)	41 (30 to 54)	6.9 (4.8 to 9.2)	2.1 (1.5 to 2.7)	231 (191 to 269)	120 (100 to 168)	18.0 (15.0 to 20.9)	6.0 (5.0 to 8.3)
Environmental heat and cold exposure	83 (54 to 117)	66 (43 to 94)	8.2 (5.5 to 11.6)	3.3 (2.1 to 4.7)	350 (314 to 393)	216 (184 to 253)	32.3 (29.1 to 36.3)	10.7 (9.1 to 12.5)
Exposure to forces of nature	17 (12 to 23)	89 (61 to 128)	1.4 (1.0 to 2.0)	4.4 (3.0 to 6.4)	108 (87 to 138)	3 769 (3 443 to 4 138)	8.9 (7.1 to 11.4)	188.9 (172.2 to 207.7)
Other unintentional injuries	115 (82 to 154)	140 (99 to 192)	11.7 (8.4 to 15.7)	6.9 (4.9 to 9.5)	996 (910 to 1 096)	873 (769 to 996)	80.2 (73.4 to 88.0)	42.6 (37.6 to 48.7)
