# Supplementary material for: Unintentional injuries in Mexico, 1990–2017: findings from the Global Burden of Disease Study 2017
Source: Inj Prev. 2020 Apr 1;26(Suppl 1):i154–61. doi: 10.1136/injuryprev-2019-043532 (PMC7571365; doi:10.1136/injuryprev-2019-043532)
Supplement: Supplementary data [file injuryprev-2019-043532supp027.pdf]

Death, YLL, incidence, prevalence, YLD, and DALY counts and rates per 100,000 for all injuries in San Luis Potosí in 1990 and 2017

Cause	Deaths				Years of life lost			
	All-age counts (95% UI)		Age-standardised rates per 100,000 (95% UI)		All-age counts (95% UI)		Age-standardised rates per 100,000 (95% UI)	
	1990	2017	1990	2017	1990	2017	1990	2017
**Transport and unintentional injuries**	**1 139 (1 096 to 1 185)**	**1 016 (952 to 1 086)**	**65.8 (63.9 to 67.8)**	**35.7 (33.4 to 38.2)**	**61 318 (58 081 to 64 909)**	**44 564 (41 263 to 48 294)**	**2 906.6 (2 782.7 to 3 039.1)**	**1 549.0 (1 433.6 to 1 679.2)**
**Transport injuries**	**512 (488 to 537)**	**521 (480 to 564)**	**28.7 (27.5 to 30.0)**	**18.1 (16.7 to 19.6)**	**28 179 (26 513 to 29 821)**	**25 204 (23 099 to 27 378)**	**1 376.1 (1 307.8 to 1 445.2)**	**866.8 (794.4 to 941.2)**
Road injuries	496 (473 to 521)	497 (458 to 539)	27.8 (26.6 to 29.0)	17.3 (16.0 to 18.7)	27 279 (25 645 to 28 904)	24 089 (22 084 to 26 139)	1 332.6 (1 265.7 to 1 400.1)	828.4 (760.2 to 899.6)
Pedestrian road injuries	237 (223 to 252)	216 (196 to 237)	14.0 (13.3 to 14.7)	7.6 (6.9 to 8.3)	12 994 (11 419 to 13 984)	9 315 (8 419 to 10 336)	607.8 (568.3 to 648.6)	324.1 (293.0 to 360.4)
Cyclist road injuries	16 (14 to 18)	26 (23 to 29)	0.9 (0.8 to 1.0)	0.9 (0.8 to 1.0)	885 (790 to 992)	1 181 (1 033 to 1 338)	43.9 (39.4 to 48.7)	40.8 (35.8 to 46.1)
Motorcyclist road injuries	70 (64 to 78)	66 (58 to 75)	3.6 (3.3 to 4.0)	2.3 (2.0 to 2.5)	4 164 (3 755 to 4 614)	3 767 (3 280 to 4 275)	201.0 (182.8 to 220.8)	127.3 (111.2 to 144.3)
Motor vehicle road injuries	152 (141 to 165)	172 (155 to 189)	8.2 (7.6 to 8.8)	6.0 (5.4 to 6.5)	8 751 (7 989 to 9 563)	8 975 (8 032 to 9 912)	428.0 (395.8 to 462.0)	307.1 (275.1 to 338.7)
Other road injuries	20 (16 to 23)	18 (15 to 20)	1.1 (0.9 to 1.2)	0.6 (0.5 to 0.7)	1 085 (903 to 1 271)	851 (727 to 989)	51.9 (43.9 to 60.2)	29.2 (25.0 to 33.8)
Other transport injuries	16 (15 to 18)	23 (21 to 26)	0.9 (0.9 to 1.0)	0.8 (0.7 to 0.9)	900 (814 to 1 006)	1 115 (972 to 1 277)	43.5 (39.7 to 48.4)	38.4 (33.5 to 44.0)
**Unintentional injuries**	**627 (597 to 659)**	**495 (464 to 529)**	**37.1 (35.9 to 38.5)**	**17.6 (16.5 to 18.8)**	**33 139 (30 830 to 35 615)**	**19 360 (17 719 to 21 270)**	**1 530.4 (1 446.9 to 1 618.2)**	**682.2 (623.5 to 750.5)**
Falls	149 (143 to 157)	177 (162 to 190)	11.7 (11.2 to 12.3)	6.3 (5.8 to 6.8)	4 758 (4 475 to 5 058)	4 069 (3 739 to 4 422)	285.2 (270.9 to 300.4)	144.5 (132.8 to 156.9)
Drowning	151 (141 to 163)	81 (74 to 89)	7.4 (7.0 to 7.8)	2.8 (2.6 to 3.1)	9 504 (8 733 to 10 489)	4 356 (3 916 to 4 876)	407.0 (379.1 to 441.6)	150.9 (135.5 to 169.0)
Fire, heat, and hot substances	47 (43 to 53)	31 (29 to 33)	2.8 (2.6 to 3.0)	1.1 (1.0 to 1.2)	2 462 (2 148 to 2 821)	1 032 (927 to 1 140)	110.0 (99.4 to 122.7)	36.5 (32.9 to 40.5)
Poisonings	28 (26 to 31)	17 (15 to 18)	1.4 (1.3 to 1.5)	0.6 (0.5 to 0.6)	1 802 (1 605 to 2 047)	834 (744 to 923)	76.3 (69.3 to 84.8)	29.1 (25.9 to 32.3)
Poisoning by carbon monoxide	12 (11 to 13)	8 (7 to 9)	0.6 (0.6 to 0.7)	0.3 (0.3 to 0.3)	792 (644 to 887)	388 (342 to 435)	31.8 (28.6 to 35.6)	13.5 (11.9 to 15.1)
Poisoning by other means	16 (15 to 18)	9 (8 to 10)	0.8 (0.7 to 0.9)	0.3 (0.3 to 0.3)	1 070 (938 to 1 226)	446 (393 to 503)	44.5 (40.0 to 49.9)	15.7 (13.8 to 17.6)
Exposure to mechanical forces	85 (80 to 90)	45 (41 to 49)	4.8 (4.5 to 5.0)	1.6 (1.4 to 1.7)	4 675 (4 359 to 5 025)	1 977 (1 791 to 2 179)	228.3 (214.5 to 242.9)	68.5 (62.1 to 75.3)
Unintentional firearm injuries	37 (33 to 43)	14 (12 to 16)	1.9 (1.8 to 2.3)	0.5 (0.4 to 0.6)	2 097 (1 896 to 2 485)	689 (567 to 799)	100.6 (91.1 to 118.2)	23.6 (19.4 to 27.2)
Other exposure to mechanical forces	48 (43 to 53)	31 (28 to 34)	2.8 (2.5 to 3.1)	1.1 (1.0 to 1.2)	2 578 (2 248 to 2 842)	1 288 (1 148 to 1 446)	127.7 (112.1 to 140.0)	44.9 (40.1 to 50.2)
Adverse effects of medical treatment	30 (27 to 33)	36 (32 to 40)	2.0 (1.8 to 2.2)	1.3 (1.1 to 1.4)	1 383 (1 187 to 1 581)	1 119 (960 to 1 398)	65.5 (58.0 to 73.4)	39.9 (34.4 to 47.2)
Animal contact	17 (15 to 19)	9 (8 to 10)	0.9 (0.8 to 0.9)	0.3 (0.3 to 0.4)	1 085 (931 to 1 264)	384 (337 to 437)	43.7 (38.6 to 49.7)	13.7 (12.0 to 15.6)
Venomous animal contact	11 (10 to 13)	5 (4 to 6)	0.5 (0.5 to 0.6)	0.2 (0.2 to 0.2)	765 (636 to 921)	220 (186 to 267)	29.6 (25.1 to 35.1)	7.9 (6.7 to 9.6)
Non venomous animal contact	6 (4 to 6)	4 (3 to 5)	0.3 (0.2 to 0.4)	0.1 (0.1 to 0.2)	319 (283 to 376)	164 (137 to 193)	14.1 (12.3 to 16.1)	5.8 (4.8 to 6.8)
Foreign body	76 (69 to 85)	74 (65 to 85)	3.7 (3.4 to 3.9)	2.7 (2.3 to 3.0)	5 108 (4 460 to 5 793)	4 934 (3 607 to 5 263)	202.2 (180.4 to 224.8)	155.6 (129.3 to 189.5)
Pulmonary aspiration and foreign body in airway	73 (65 to 80)	73 (64 to 83)	3.5 (3.2 to 3.7)	2.6 (2.3 to 3.0)	4 858 (4 233 to 5 528)	4 239 (3 518 to 5 169)	191.7 (171.2 to 213.5)	152.3 (126.0 to 186.3)
Foreign body in eyes								
Foreign body in other body part	4 (3 to 4)	2 (1 to 3)	0.2 (0.1 to 0.2)	0.1 (0.0 to 0.1)	250 (172 to 297)	95 (77 to 136)	10.5 (7.5 to 12.2)	3.3 (2.7 to 5.4)
Environmental heat and cold exposure	14 (13 to 15)	7 (6 to 8)	0.9 (0.9 to 1.0)	0.2 (0.2 to 0.3)	638 (582 to 695)	245 (218 to 273)	32.6 (30.3 to 35.0)	8.7 (7.7 to 9.5)
Exposure to forces of nature	1 (1 to 1)	0 (0 to 0)	0.0 (0.0 to 0.0)	0.0 (0.0 to 0.0)	51 (46 to 57)	23 (21 to 26)	2.3 (2.1 to 2.5)	0.8 (0.7 to 0.9)
Other unintentional injuries	29 (26 to 31)	19 (17 to 21)	1.5 (1.4 to 1.6)	0.7 (0.6 to 0.7)	1 678 (1 580 to 1 880)	992 (888 to 1 117)	77.3 (71.3 to 83.6)	34.0 (30.2 to 38.1)

Cause	Incidence				Prevalence			
	All-age counts (95% UI)		Age-standardised rates per 100,000 (95% UI)		All-age counts (95% UI)		Age-standardised rates per 100,000 (95% UI)	
	1990	2017	1990	2017	1990	2017	1990	2017
**Transport and unintentional injuries**	**79 044 (72 480 to 86 424)**	**120 811 (110 604 to 132 641)**	**4 300.4 (3 913.5 to 4 689.7)**	**4 259.9 (3 894.4 to 4 665.7)**	**152 037 (143 057 to 162 881)**	**257 693 (242 690 to 277 196)**	**9 917.5 (9 348.7 to 10 630.0)**	**9 115.5 (8 587.1 to 9 793.3)**
**Transport injuries**	**7 067 (6 046 to 8 223)**	**16 328 (14 064 to 18 703)**	**379.5 (329.8 to 433.3)**	**566.3 (487.3 to 648.0)**	**17 413 (16 270 to 18 541)**	**46 754 (43 534 to 50 171)**	**1 183.3 (1 105.0 to 1 262.1)**	**1 654.5 (1 541.8 to 1 775.8)**
Road injuries	6 725 (5 753 to 7 877)	16 038 (13 792 to 18 413)	362.7 (312.9 to 415.7)	556.1 (478.3 to 638.1)	15 869 (14 833 to 16 938)	45 288 (42 052 to 48 715)	1 087.7 (1 015.3 to 1 163.5)	1 602.6 (1 488.5 to 1 723.7)
Pedestrian road injuries	999 (810 to 1 213)	1 905 (1 539 to 2 291)	56.2 (46.5 to 67.1)	66.6 (53.7 to 80.1)	3 782 (3 262 to 4 428)	8 856 (7 746 to 10 290)	262.7 (229.7 to 305.6)	314.4 (275.2 to 365.7)
Cyclist road injuries	1 203 (941 to 1 529)	2 647 (2 124 to 3 267)	59.6 (48.0 to 74.5)	92.0 (73.9 to 112.8)	2 122 (1 783 to 2 481)	5 935 (5 044 to 6 895)	137.7 (116.6 to 159.3)	209.3 (178.0 to 242.8)
Motorcyclist road injuries	808 (640 to 1 005)	1 576 (1 234 to 2 007)	42.1 (34.0 to 51.8)	54.0 (41.7 to 68.7)	3 021 (2 621 to 3 479)	7 285 (6 229 to 8 506)	204.6 (177.3 to 235.7)	256.9 (219.7 to 299.9)
Motor vehicle road injuries	2 299 (1 880 to 2 738)	6 958 (5 788 to 8 319)	128.2 (106.4 to 151.6)	240.9 (200.8 to 287.2)	4 284 (3 758 to 4 963)	16 199 (14 068 to 18 948)	302.7 (264.3 to 350.2)	574.1 (498.1 to 670.9)
Other road injuries	1 423 (1 111 to 1 754)	2 951 (2 350 to 3 585)	76.5 (61.5 to 92.4)	102.6 (81.8 to 124.2)	2 660 (2 237 to 3 191)	7 013 (5 847 to 8 521)	179.9 (152.1 to 215.1)	248.0 (206.8 to 301.3)
Other transport injuries	342 (259 to 460)	290 (236 to 356)	16.9 (13.6 to 21.0)	10.2 (8.3 to 12.6)	1 544 (1 281 to 1 831)	1 466 (1 270 to 1 671)	95.6 (80.8 to 111.0)	51.9 (45.0 to 59.1)
**Unintentional injuries**	**71 977 (65 468 to 79 465)**	**104 484 (94 513 to 115 865)**	**3 920.9 (3 540.1 to 4 316.1)**	**3 693.6 (3 336.5 to 4 093.5)**	**134 624 (125 761 to 145 145)**	**210 939 (195 960 to 230 004)**	**8 734.1 (8 164.6 to 9 430.3)**	**7 461.0 (6 929.9 to 8 141.6)**
Falls	31 461 (27 844 to 35 695)	56 632 (50 530 to 63 743)	1 836.8 (1 645.5 to 2 066.0)	2 033.8 (1 793.1 to 2 275.5)	54 594 (48 394 to 62 093)	108 602 (97 283 to 124 296)	3 731.6 (3 324.7 to 4 231.1)	3 857.0 (3 453.2 to 4 417.5)
Drowning	130 (113 to 151)	71 (57 to 90)	6.4 (5.5 to 7.2)	2.5 (2.0 to 3.1)	600 (527 to 687)	442 (389 to 513)	40.4 (35.7 to 46.0)	15.7 (13.8 to 18.2)
Fire, heat, and hot substances	2 864 (2 312 to 3 564)	2 706 (2 230 to 3 250)	117.0 (97.2 to 141.4)	95.9 (79.9 to 115.2)	26 993 (22 406 to 33 782)	32 089 (26 536 to 40 590)	1 566.3 (1 313.1 to 1 907.8)	1 124.4 (930.6 to 1 419.9)
Poisonings	923 (672 to 1 254)	705 (489 to 979)	34.4 (25.9 to 45.4)	25.1 (17.5 to 34.9)	769 (591 to 950)	840 (620 to 1 058)	39.8 (31.0 to 49.2)	29.1 (21.5 to 36.7)
Poisoning by carbon monoxide	180 (135 to 273)	142 (76 to 235)	7.4 (5.1 to 10.6)	5.0 (2.7 to 8.2)	118 (93 to 155)	112 (86 to 144)	6.3 (4.9 to 8.2)	3.9 (3.0 to 5.0)
Poisoning by other means	743 (531 to 1 041)	563 (374 to 815)	26.9 (19.6 to 36.8)	20.1 (13.4 to 29.2)	651 (473 to 831)	727 (504 to 943)	33.5 (24.6 to 42.6)	25.2 (17.5 to 32.7)
Exposure to mechanical forces	16 826 (14 295 to 19 666)	18 518 (15 929 to 21 576)	840.9 (726.8 to 970.4)	642.8 (555.4 to 748.4)	33 366 (29 367 to 38 804)	46 478 (40 490 to 53 764)	2 221.3 (1 952.3 to 2 538.9)	1 640.8 (1 430.8 to 1 899.1)
Unintentional firearm injuries	1 176 (867 to 1 562)	725 (517 to 972)	57.7 (43.7 to 75.7)	25.0 (18.0 to 33.5)	4 433 (3 538 to 5 320)	3 805 (3 056 to 4 623)	300.4 (241.7 to 363.2)	134.8 (108.6 to 164.0)
Other exposure to mechanical forces	15 650 (13 273 to 18 292)	17 793 (15 307 to 20 677)	783.2 (675.8 to 909.8)	617.8 (532.7 to 718.2)	28 932 (24 589 to 34 103)	42 672 (36 445 to 50 305)	1 920.9 (1 642.6 to 2 255.4)	1 506.0 (1 287.0 to 1 774.2)
Adverse effects of medical treatment	1 449 (1 218 to 1 694)	2 824 (2 404 to 3 279)	86.2 (73.5 to 99.6)	100.9 (85.2 to 116.4)	111 (86 to 139)	216 (166 to 269)	6.6 (5.0 to 8.1)	7.7 (5.9 to 9.5)
Animal contact	5 964 (5 016 to 7 217)	6 852 (5 989 to 7 844)	296.2 (255.4 to 343.2)	241.4 (210.9 to 276.2)	6 198 (5 320 to 7 182)	8 457 (7 449 to 9 578)	390.8 (340.0 to 444.6)	298.6 (263.7 to 337.4)
Venomous animal contact	2 119 (1 629 to 2 736)	2 731 (2 199 to 3 339)	105.2 (81.7 to 132.1)	96.1 (77.4 to 117.2)	1 369 (1 129 to 1 624)	2 097 (1 761 to 2 426)	81.4 (68.1 to 94.8)	73.8 (62.1 to 85.2)
Non venomous animal contact	3 846 (3 348 to 4 429)	4 120 (3 463 to 4 928)	191.1 (160.6 to 229.7)	145.3 (121.3 to 173.7)	4 829 (3 989 to 5 786)	6 359 (5 391 to 7 458)	309.4 (259.7 to 363.5)	224.8 (190.8 to 263.0)
Foreign body	7 682 (3 755 to 12 056)	12 085 (5 326 to 20 767)	466.4 (198.6 to 767.8)	429.2 (187.4 to 742.6)	3 224 (2 440 to 4 323)	4 612 (3 412 to 6 326)	202.6 (155.0 to 270.9)	163.6 (120.9 to 223.5)
Pulmonary aspiration and foreign body in airway	196 (138 to 269)	109 (76 to 156)	7.6 (5.7 to 10.1)	3.8 (2.7 to 5.5)	133 (95 to 181)	99 (81 to 122)	7.0 (5.2 to 9.3)	3.5 (2.8 to 4.2)
Foreign body in eyes	6 944 (3 025 to 11 371)	11 449 (4 616 to 20 117)	405.1 (166.8 to 784.3)	406.6 (161.3 to 729.2)	1 481 (756 to 2 414)	2 596 (1 446 to 4 216)	99.9 (55.7 to 161.7)	92.8 (52.1 to 150.2)
Foreign body in other body part	542 (396 to 758)	527 (421 to 653)	23.7 (18.0 to 31.3)	18.7 (14.9 to 23.3)	1 660 (1 337 to 2 109)	1 917 (1 604 to 2 342)	95.7 (78.5 to 118.8)	67.4 (56.5 to 82.1)
Environmental heat and cold exposure	955 (698 to 1 249)	486 (382 to 595)	49.5 (38.8 to 60.5)	17.2 (13.5 to 20.9)	3 254 (2 675 to 3 918)	1 812 (1 570 to 2 137)	203.7 (171.6 to 239.3)	64.1 (55.7 to 75.7)
Exposure to forces of nature	134 (110 to 168)	31 (26 to 37)	5.8 (4.9 to 7.0)	1.1 (0.9 to 1.3)	377 (279 to 510)	1 062 (745 to 1 466)	19.0 (13.9 to 25.5)	36.9 (26.0 to 51.0)
Other unintentional injuries	3 586 (2 925 to 4 414)	3 573 (2 998 to 4 206)	181.3 (152.1 to 217.0)	124.4 (104.0 to 146.6)	5 138 (4 400 to 5 925)	6 329 (5 488 to 7 237)	332.1 (286.9 to 380.1)	223.1 (193.7 to 255.0)

Cause	Years lived with disability				Disability adjusted life years			
	All-age counts (95% UI)		Age-standardised rates per 100,000 (95% UI)		All-age counts (95% UI)		Age-standardised rates per 100,000 (95% UI)	
	1990	2017	1990	2017	1990	2017	1990	2017
**Transport and unintentional injuries**	**6 744 (4 964 to 8 926)**	**10 988 (7 909 to 14 719)**	**435.2 (319.4 to 577.4)**	**388.6 (279.6 to 521.4)**	**68 061 (64 337 to 72 522)**	**55 552 (50 972 to 60 832)**	**3 341.8 (3 173.2 to 3 538.8)**	**1 937.7 (1 776.6 to 2 123.2)**
**Transport injuries**	**1 201 (879 to 1 580)**	**2 848 (2 074 to 3 811)**	**79.3 (58.6 to 104.4)**	**100.5 (73.2 to 134.5)**	**29 380 (27 610 to 31 145)**	**28 052 (25 775 to 30 591)**	**1 455.5 (1 384.6 to 1 529.4)**	**967.4 (890.1 to 1 054.0)**
Road injuries	1 101 (809 to 1 453)	2 770 (2 014 to 3 711)	73.4 (54.2 to 96.8)	97.8 (71.1 to 130.9)	28 380 (26 673 to 30 068)	26 859 (24 695 to 29 282)	1 406.0 (1 337.3 to 1 478.6)	926.2 (852.4 to 1 010.3)
Pedestrian road injuries	273 (193 to 369)	565 (401 to 768)	18.4 (13.0 to 24.9)	20.0 (14.2 to 27.3)	12 666 (11 705 to 13 641)	9 880 (8 966 to 10 927)	626.2 (585.1 to 667.4)	344.1 (312.5 to 380.5)
Cyclist road injuries	150 (106 to 199)	360 (252 to 481)	9.5 (6.7 to 12.5)	12.7 (8.9 to 16.9)	1 035 (925 to 1 150)	1 542 (1 358 to 1 734)	53.4 (48.1 to 59.1)	53.5 (47.2 to 60.2)
Motorcyclist road injuries	214 (150 to 291)	452 (310 to 617)	14.1 (9.9 to 19.1)	15.9 (10.9 to 21.7)	4 378 (3 956 to 4 830)	4 218 (3 710 to 4 783)	215.2 (195.9 to 235.5)	143.1 (126.3 to 162.3)
Motor vehicle road injuries	309 (223 to 409)	1 030 (742 to 1 370)	21.2 (15.4 to 27.9)	36.4 (26.3 to 48.5)	9 060 (8 302 to 9 861)	10 005 (9 001 to 11 002)	449.2 (415.6 to 483.6)	343.5 (309.4 to 377.6)
Other road injuries	156 (112 to 211)	368 (259 to 508)	10.2 (7.4 to 13.8)	12.8 (9.1 to 17.7)	1 240 (1 051 to 1 438)	1 215 (1 041 to 1 424)	62.1 (53.7 to 70.9)	42.0 (35.9 to 49.2)
Other transport injuries	100 (71 to 133)	78 (56 to 104)	6.0 (4.3 to 7.8)	2.8 (2.0 to 3.7)	1 001 (905 to 1 108)	1 193 (1 049 to 1 349)	49.5 (45.0 to 54.4)	41.2 (36.0 to 46.4)
**Unintentional injuries**	**5 542 (4 034 to 7 322)**	**8 140 (5 777 to 11 021)**	**355.8 (258.4 to 473.1)**	**288.1 (204.5 to 390.1)**	**38 681 (35 867 to 41 937)**	**27 500 (24 615 to 30 715)**	**1 886.3 (1 754.1 to 2 036.0)**	**970.3 (868.0 to 1 083.5)**
Falls	3 296 (2 434 to 4 303)	5 591 (4 018 to 7 408)	218.8 (160.1 to 284.5)	198.4 (142.6 to 263.4)	8 054 (7 147 to 9 137)	9 661 (8 018 to 11 497)	504.1 (445.9 to 573.5)	342.9 (284.6 to 407.3)
Drowning	34 (24 to 47)	23 (16 to 32)	2.2 (1.6 to 3.0)	0.8 (0.6 to 1.1)	9 539 (8 737 to 10 485)	4 380 (3 943 to 4 898)	409.2 (381.1 to 443.7)	151.8 (136.5 to 169.9)
Fire, heat, and hot substances	590 (376 to 914)	622 (368 to 1 025)	33.2 (21.0 to 51.5)	21.8 (12.9 to 35.8)	3 052 (2 658 to 3 516)	1 654 (1 370 to 2 054)	143.2 (125.7 to 164.4)	58.3 (48.3 to 72.4)
Poisonings	91 (55 to 136)	97 (58 to 147)	4.6 (2.8 to 6.8)	3.4 (2.0 to 5.1)	1 892 (1 691 to 2 135)	931 (824 to 1 034)	80.9 (73.6 to 89.7)	32.5 (28.7 to 36.0)
Poisoning by carbon monoxide	13 (8 to 20)	11 (7 to 17)	0.6 (0.4 to 1.0)	0.4 (0.2 to 0.6)	744 (656 to 851)	399 (353 to 446)	32.5 (29.2 to 36.2)	13.9 (12.2 to 15.5)
Poisoning by other means	78 (46 to 117)	86 (50 to 134)	3.9 (2.4 to 5.9)	3.0 (1.7 to 4.6)	1 148 (1 009 to 1 303)	532 (469 to 606)	48.4 (43.5 to 54.0)	18.6 (16.2 to 21.2)
Exposure to mechanical forces	793 (551 to 1 130)	1 006 (681 to 1 464)	51.9 (36.0 to 74.5)	35.5 (24.0 to 51.7)	5 468 (5 062 to 5 952)	2 983 (2 593 to 3 498)	280.2 (259.0 to 307.6)	103.9 (90.2 to 122.0)
Unintentional firearm injuries	92 (61 to 139)	69 (44 to 110)	6.0 (4.0 to 9.2)	2.4 (1.6 to 3.9)	2 189 (1 985 to 2 527)	758 (625 to 868)	106.6 (97.3 to 123.7)	26.0 (21.6 to 29.9)
Other exposure to mechanical forces	702 (485 to 1 001)	937 (632 to 1 370)	45.9 (31.9 to 65.6)	33.0 (22.3 to 48.3)	3 279 (2 892 to 3 647)	2 225 (1 884 to 2 674)	173.6 (154.1 to 195.3)	77.9 (65.9 to 93.7)
Adverse effects of medical treatment	15 (9 to 22)	29 (18 to 44)	0.9 (0.6 to 1.3)	1.0 (0.6 to 1.6)	1 398 (1 205 to 1 591)	1 142 (988 to 1 323)	66.4 (59.0 to 74.3)	40.9 (35.3 to 48.1)
Animal contact	162 (109 to 229)	188 (125 to 269)	9.3 (6.3 to 13.3)	6.6 (4.4 to 9.4)	1 247 (1 083 to 1 445)	572 (490 to 674)	53.0 (46.7 to 60.7)	20.3 (17.4 to 23.9)
Venomous animal contact	87 (55 to 128)	98 (64 to 143)	4.6 (3.0 to 6.6)	3.4 (2.2 to 5.0)	852 (720 to 1 010)	318 (267 to 384)	34.2 (29.5 to 39.9)	11.3 (9.5 to 13.6)
Non venomous animal contact	75 (46 to 120)	90 (53 to 145)	4.8 (2.9 to 7.5)	3.2 (1.9 to 5.1)	395 (297 to 465)	253 (205 to 319)	18.8 (14.2 to 22.5)	8.9 (7.2 to 11.2)
Foreign body	206 (133 to 303)	267 (164 to 430)	12.8 (8.3 to 19.0)	9.5 (5.8 to 15.3)	5 313 (4 665 to 6 017)	4 600 (3 855 to 5 552)	214.9 (192.6 to 238.6)	165.1 (137.9 to 199.7)
Pulmonary aspiration and foreign body in airway	7 (5 to 10)	4 (3 to 6)	0.4 (0.3 to 0.5)	0.1 (0.1 to 0.2)	4 865 (4 239 to 5 535)	4 243 (3 523 to 5 174)	192.1 (171.5 to 213.8)	152.5 (126.2 to 186.4)
Foreign body in eyes	95 (48 to 167)	173 (91 to 307)	6.7 (3.5 to 11.6)	6.2 (3.3 to 11.0)	95 (48 to 167)	173 (91 to 307)	6.7 (3.5 to 11.6)	6.2 (3.3 to 11.0)
Foreign body in other body part	103 (72 to 137)	90 (64 to 119)	5.7 (4.0 to 7.5)	3.1 (2.2 to 4.2)	353 (282 to 411)	185 (152 to 244)	16.2 (13.3 to 18.6)	6.5 (5.3 to 8.5)
Environmental heat and cold exposure	130 (88 to 183)	70 (46 to 101)	8.1 (5.5 to 11.4)	2.5 (1.6 to 3.6)	768 (692 to 848)	315 (278 to 356)	40.7 (37.1 to 44.9)	11.1 (9.8 to 12.6)
Exposure to forces of nature	19 (14 to 27)	35 (25 to 49)	1.0 (0.7 to 1.3)	1.2 (0.9 to 1.7)	71 (63 to 80)	58 (48 to 72)	3.3 (2.9 to 3.7)	2.0 (1.7 to 2.5)
Other unintentional injuries	206 (148 to 271)	212 (149 to 289)	13.1 (9.5 to 17.2)	7.5 (5.2 to 10.2)	1 884 (1 738 to 2 059)	1 204 (1 072 to 1 353)	90.4 (83.2 to 98.2)	41.4 (35.9 to 46.4)
