# Supplementary material for: Unintentional injuries in Mexico, 1990–2017: findings from the Global Burden of Disease Study 2017
Source: Inj Prev. 2020 Apr 1;26(Suppl 1):i154–61. doi: 10.1136/injuryprev-2019-043532 (PMC7571365; doi:10.1136/injuryprev-2019-043532)
Supplement: Supplementary data [file injuryprev-2019-043532supp031.pdf]

Death, YLL, incidence, prevalence, YLD, and DALY counts and rates per 100,000 for all injuries in Tlaxcala in 1990 and 2017

Cause	Deaths: All-age counts (95% UI) 1990	Deaths: All-age counts (95% UI) 2017	Deaths: Age-standardised rates per 100,000 (95% UI) 1990	Deaths: Age-standardised rates per 100,000 (95% UI) 2017	Years of life lost: All-age counts (95% UI) 1990	Years of life lost: All-age counts (95% UI) 2017	Years of life lost: Age-standardised rates per 100,000 (95% UI) 1990	Years of life lost: Age-standardised rates per 100,000 (95% UI) 2017
**Transport and unintentional injuries**	**431 (414 to 450)**	**485 (450 to 519)**	**67.0 (65.1 to 69.2)**	**37.9 (35.3 to 40.5)**	**23 206 (21 948 to 24 594)**	**21 780 (20 016 to 23 557)**	**2 884.8 (2 765.1 to 3 015.9)**	**1 596.2 (1 467.3 to 1 726.3)**
**Transport injuries**	**205 (196 to 216)**	**249 (227 to 269)**	**30.4 (29.1 to 31.8)**	**18.6 (17.0 to 20.1)**	**11 393 (10 775 to 12 016)**	**12 413 (11 289 to 13 450)**	**1 443.5 (1 374.0 to 1 517.1)**	**891.3 (811.1 to 965.5)**
Road injuries	200 (191 to 210)	239 (218 to 259)	29.6 (28.3 to 31.0)	17.9 (16.3 to 19.4)	11 088 (10 495 to 11 735)	11 945 (10 872 to 12 951)	1 405.6 (1 337.6 to 1 475.8)	857.7 (783.8 to 929.6)
Pedestrian road injuries	104 (98 to 111)	105 (95 to 115)	16.3 (15.5 to 17.2)	8.2 (7.4 to 8.9)	5 521 (5 091 to 5 944)	4 719 (4 298 to 5 204)	706.2 (661.2 to 752.9)	346.1 (311.9 to 381.5)
Cyclist road injuries	5 (4 to 5)	9 (8 to 11)	0.7 (0.6 to 0.8)	0.7 (0.6 to 0.8)	268 (239 to 300)	444 (389 to 507)	35.0 (31.5 to 38.7)	32.3 (28.3 to 36.6)
Motorcyclist road injuries	26 (23 to 29)	29 (25 to 32)	3.5 (3.2 to 3.8)	2.0 (1.8 to 2.3)	1 537 (1 384 to 1 713)	1 637 (1 410 to 1 862)	190.7 (172.5 to 210.2)	114.4 (98.4 to 129.7)
Motor vehicle road injuries	60 (55 to 65)	90 (79 to 100)	8.3 (7.8 to 9.0)	6.5 (5.8 to 7.2)	3 455 (3 164 to 3 783)	4 833 (4 278 to 5 379)	436.0 (403.2 to 473.2)	342.6 (302.8 to 380.2)
Other road injuries	5 (5 to 6)	6 (5 to 7)	0.8 (0.7 to 0.9)	0.5 (0.4 to 0.5)	308 (260 to 357)	311 (269 to 362)	37.6 (32.3 to 42.9)	22.4 (19.4 to 25.9)
Other transport injuries	5 (5 to 6)	10 (8 to 11)	0.8 (0.7 to 0.9)	0.7 (0.6 to 0.8)	304 (271 to 340)	468 (407 to 531)	38.0 (34.3 to 42.0)	33.7 (29.3 to 38.1)
**Unintentional injuries**	**226 (214 to 237)**	**236 (219 to 253)**	**36.7 (35.3 to 38.0)**	**19.3 (18.0 to 20.6)**	**11 813 (10 928 to 12 741)**	**9 367 (8 528 to 10 290)**	**1 441.3 (1 362.2 to 1 525.9)**	**703.9 (642.1 to 771.7)**
Falls	59 (56 to 62)	86 (79 to 93)	12.8 (12.2 to 13.5)	7.6 (7.0 to 8.2)	1 929 (1 795 to 2 056)	2 129 (1 951 to 2 332)	309.8 (292.5 to 327.3)	169.8 (156.0 to 185.5)
Drowning	39 (36 to 43)	28 (26 to 31)	5.2 (4.8 to 5.5)	2.1 (1.9 to 2.3)	2 447 (2 225 to 2 684)	1 497 (1 330 to 1 688)	275.5 (254.0 to 298.1)	108.3 (96.2 to 121.6)
Fire, heat, and hot substances	18 (17 to 20)	14 (13 to 15)	3.0 (2.8 to 3.2)	1.2 (1.1 to 1.3)	949 (842 to 1 078)	494 (451 to 544)	112.0 (102.4 to 123.2)	37.7 (34.5 to 41.4)
Poisonings	12 (11 to 13)	9 (8 to 10)	1.5 (1.4 to 1.6)	0.7 (0.6 to 0.7)	736 (657 to 816)	443 (399 to 491)	81.4 (74.4 to 88.5)	32.4 (29.2 to 35.8)
Poisoning by carbon monoxide	5 (5 to 6)	5 (4 to 5)	0.7 (0.7 to 0.8)	0.4 (0.3 to 0.4)	336 (299 to 378)	237 (209 to 264)	38.2 (34.6 to 41.8)	17.2 (15.3 to 19.1)
Poisoning by other means	6 (6 to 7)	4 (4 to 4)	0.8 (0.7 to 0.9)	0.3 (0.3 to 0.3)	400 (353 to 454)	207 (184 to 234)	43.3 (39.0 to 47.8)	15.2 (13.5 to 17.2)
Exposure to mechanical forces	25 (24 to 27)	19 (17 to 20)	3.8 (3.6 to 4.1)	1.4 (1.3 to 1.5)	1 379 (1 278 to 1 491)	861 (776 to 949)	178.3 (165.8 to 191.6)	62.4 (56.4 to 68.7)
Unintentional firearm injuries	10 (9 to 11)	6 (4 to 6)	1.4 (1.3 to 1.6)	0.4 (0.3 to 0.5)	571 (505 to 652)	284 (226 to 332)	72.3 (64.5 to 82.3)	20.2 (16.1 to 23.5)
Other exposure to mechanical forces	15 (14 to 17)	13 (12 to 14)	2.4 (2.2 to 2.6)	1.0 (0.9 to 1.1)	808 (717 to 888)	577 (517 to 636)	106.1 (95.1 to 116.2)	42.2 (38.0 to 47.7)
Adverse effects of medical treatment	13 (11 to 14)	19 (17 to 23)	2.4 (2.0 to 2.6)	1.6 (1.4 to 1.9)	599 (474 to 677)	611 (534 to 791)	75.7 (61.9 to 83.7)	47.7 (41.9 to 61.2)
Animal contact	4 (4 to 5)	2 (2 to 3)	0.5 (0.5 to 0.6)	0.2 (0.2 to 0.2)	278 (236 to 329)	102 (90 to 116)	28.4 (24.9 to 32.9)	7.7 (6.8 to 8.8)
Venomous animal contact	2 (2 to 3)	1 (1 to 1)	0.2 (0.2 to 0.3)	0.1 (0.0 to 0.1)	161 (127 to 204)	35 (27 to 52)	15.0 (12.1 to 19.0)	2.7 (2.1 to 3.9)
Non venomous animal contact	2 (1 to 2)	2 (1 to 2)	0.3 (0.2 to 0.3)	0.1 (0.1 to 0.2)	117 (84 to 137)	67 (53 to 78)	13.4 (9.8 to 15.4)	5.1 (4.0 to 5.9)
Foreign body	38 (34 to 42)	45 (40 to 51)	4.8 (4.5 to 5.2)	3.5 (3.1 to 3.9)	2 568 (2 229 to 2 932)	2 541 (2 154 to 3 031)	262.9 (233.6 to 293.5)	188.3 (159.5 to 224.4)
Pulmonary aspiration and foreign body in airway	37 (33 to 41)	44 (39 to 50)	4.7 (4.3 to 5.0)	3.4 (3.0 to 3.9)	2 474 (2 136 to 2 838)	2 488 (2 108 to 2 967)	252.5 (223.9 to 282.7)	184.5 (156.4 to 220.2)
Foreign body in eyes								
Foreign body in other body part	1 (1 to 2)	1 (1 to 2)	0.2 (0.2 to 0.2)	0.1 (0.1 to 0.1)	94 (77 to 111)	53 (43 to 98)	10.4 (8.7 to 12.1)	3.8 (3.1 to 7.0)
Environmental heat and cold exposure	5 (4 to 5)	3 (3 to 3)	0.8 (0.8 to 0.9)	0.2 (0.2 to 0.2)	210 (192 to 230)	105 (94 to 117)	29.0 (27.1 to 31.1)	7.9 (7.1 to 8.8)
Exposure to forces of nature	0 (0 to 0)	0 (0 to 0)	0.1 (0.1 to 0.1)	0.0 (0.0 to 0.0)	24 (22 to 27)	11 (10 to 12)	2.8 (2.6 to 3.1)	0.8 (0.7 to 0.9)
Other unintentional injuries	12 (11 to 13)	11 (10 to 12)	1.7 (1.6 to 1.8)	0.8 (0.7 to 0.9)	699 (638 to 764)	574 (504 to 647)	85.5 (78.7 to 93.0)	40.8 (35.9 to 45.8)

Cause	Incidence: All-age counts (95% UI) 1990	Incidence: All-age counts (95% UI) 2017	Incidence: Age-standardised rates per 100,000 (95% UI) 1990	Incidence: Age-standardised rates per 100,000 (95% UI) 2017	Prevalence: All-age counts (95% UI) 1990	Prevalence: All-age counts (95% UI) 2017	Prevalence: Age-standardised rates per 100,000 (95% UI) 1990	Prevalence: Age-standardised rates per 100,000 (95% UI) 2017
**Transport and unintentional injuries**	**29 486 (27 037 to 32 140)**	**56 421 (51 663 to 61 991)**	**4 229.7 (3 860.8 to 4 637.5)**	**4 382.3 (4 005.8 to 4 808.8)**	**54 623 (51 413 to 58 378)**	**115 421 (108 483 to 123 856)**	**9 558.9 (9 019.4 to 10 244.8)**	**9 139.4 (8 590.7 to 9 818.5)**
**Transport injuries**	**2 755 (2 374 to 3 189)**	**7 334 (6 530 to 8 597)**	**385.9 (336.3 to 437.2)**	**555.7 (484.1 to 631.8)**	**6 546 (6 103 to 6 982)**	**20 700 (19 110 to 22 395)**	**1 191.9 (1 112.2 to 1 273.3)**	**1 621.7 (1 499.6 to 1 755.4)**
Road injuries	2 627 (2 257 to 3 056)	7 401 (6 399 to 8 459)	369.9 (321.8 to 422.6)	545.5 (474.7 to 621.8)	5 983 (5 578 to 6 405)	20 041 (18 457 to 21 711)	1 100.9 (1 025.5 to 1 182.1)	1 569.6 (1 448.4 to 1 700.3)
Pedestrian road injuries	427 (349 to 527)	929 (751 to 1 133)	69.1 (62.9 to 75.6)	69.7 (56.4 to 84.9)	1 559 (1 336 to 1 815)	4 209 (3 662 to 4 905)	269.4 (231.5 to 335.2)	333.5 (291.2 to 387.4)
Cyclist road injuries	417 (320 to 534)	1 111 (887 to 1 383)	53.6 (42.8 to 66.8)	82.3 (65.8 to 101.7)	690 (579 to 819)	2 445 (2 056 to 2 887)	119.0 (101.3 to 138.3)	189.3 (160.0 to 222.0)
Motorcyclist road injuries	319 (247 to 404)	563 (425 to 723)	42.8 (33.8 to 52.5)	40.7 (31.0 to 52.0)	1 129 (976 to 1 313)	2 518 (2 153 to 2 947)	204.3 (175.1 to 236.1)	194.2 (166.6 to 226.8)
Motor vehicle road injuries	1 032 (851 to 1 231)	4 043 (3 388 to 4 762)	150.1 (124.7 to 176.8)	295.7 (248.1 to 346.7)	1 820 (1 596 to 2 119)	9 061 (7 890 to 10 390)	346.1 (300.5 to 401.8)	710.0 (614.9 to 828.2)
Other road injuries	432 (328 to 553)	755 (586 to 942)	60.4 (46.5 to 74.8)	57.1 (44.5 to 71.4)	791 (659 to 950)	1 807 (1 519 to 2 183)	142.1 (118.9 to 170.4)	142.7 (120.0 to 171.4)
Other transport injuries	128 (95 to 175)	134 (108 to 164)	16.0 (12.8 to 20.3)	10.2 (8.3 to 12.5)	564 (465 to 679)	660 (566 to 755)	90.9 (76.6 to 106.3)	52.0 (45.0 to 59.2)
**Unintentional injuries**	**26 731 (24 371 to 29 381)**	**48 886 (44 239 to 54 303)**	**3 843.8 (3 478.6 to 4 257.5)**	**3 826.5 (3 455.7 to 4 252.7)**	**48 077 (44 900 to 51 786)**	**94 720 (87 766 to 102 969)**	**8 367.0 (7 839.2 to 9 036.3)**	**7 517.7 (6 976.8 to 8 191.6)**
Falls	11 916 (10 458 to 13 545)	26 564 (23 609 to 30 092)	1 856.8 (1 650.3 to 2 086.0)	2 135.0 (1 898.9 to 2 406.9)	20 269 (18 034 to 23 235)	51 479 (45 938 to 58 562)	3 726.5 (3 338.6 to 4 259.8)	4 170.8 (3 725.0 to 4 760.8)
Drowning	36 (31 to 42)	28 (23 to 36)	4.6 (4.0 to 5.3)	2.1 (1.7 to 2.7)	160 (141 to 185)	169 (148 to 198)	29.1 (25.7 to 33.8)	13.5 (11.8 to 15.7)
Fire, heat, and hot substances	1 041 (835 to 1 294)	1 150 (947 to 1 397)	110.8 (91.2 to 134.2)	86.0 (71.0 to 104.2)	9 425 (7 888 to 11 615)	13 181 (10 959 to 16 805)	1 457.6 (1 237.2 to 1 751.7)	1 002.4 (836.2 to 1 266.6)
Poisonings	348 (259 to 473)	437 (321 to 585)	33.3 (25.5 to 43.7)	32.6 (23.9 to 43.7)	282 (217 to 345)	506 (379 to 635)	38.2 (29.7 to 46.7)	37.3 (27.6 to 46.8)
Poisoning by carbon monoxide	65 (43 to 98)	63 (41 to 93)	7.0 (5.0 to 9.9)	4.7 (3.1 to 6.9)	42 (32 to 54)	46 (36 to 60)	5.9 (4.6 to 7.4)	3.5 (2.7 to 4.5)
Poisoning by other means	283 (206 to 389)	374 (267 to 505)	26.4 (19.6 to 35.4)	27.8 (19.9 to 37.8)	240 (174 to 303)	459 (325 to 587)	32.3 (23.9 to 40.7)	33.9 (24.0 to 43.2)
Exposure to mechanical forces	5 493 (4 667 to 6 578)	7 604 (6 522 to 8 928)	714.9 (617.2 to 844.8)	557.0 (479.7 to 651.1)	10 508 (9 218 to 12 118)	18 149 (15 878 to 20 956)	1 876.7 (1 646.4 to 2 153.3)	1 409.9 (1 233.7 to 1 627.5)
Unintentional firearm injuries	362 (259 to 494)	316 (225 to 426)	45.5 (33.4 to 60.6)	23.0 (16.5 to 30.7)	1 392 (1 067 to 1 591)	1 563 (1 262 to 1 882)	240.8 (194.0 to 290.3)	122.8 (99.5 to 147.0)
Other exposure to mechanical forces	5 130 (4 353 to 6 162)	7 289 (6 266 to 8 560)	669.4 (577.1 to 789.4)	534.0 (461.3 to 626.1)	9 176 (7 808 to 10 793)	16 585 (14 238 to 19 516)	1 636.0 (1 402.2 to 1 912.1)	1 287.1 (1 106.1 to 1 513.7)
Adverse effects of medical treatment	602 (508 to 696)	1 496 (1 264 to 1 720)	94.6 (81.9 to 108.7)	116.6 (98.4 to 133.0)	46 (36 to 58)	115 (88 to 142)	7.2 (5.6 to 9.0)	8.9 (6.9 to 11.0)
Animal contact	2 561 (2 356 to 3 110)	4 126 (3 622 to 4 754)	325.1 (281.7 to 379.0)	315.0 (277.7 to 360.3)	2 588 (2 225 to 2 993)	4 922 (4 341 to 5 573)	430.5 (375.9 to 489.0)	387.1 (342.2 to 436.5)
Venomous animal contact	979 (751 to 1 259)	1 695 (1 362 to 2 079)	123.0 (97.0 to 153.6)	129.1 (104.2 to 158.1)	633 (518 to 759)	1 275 (1 066 to 1 487)	99.4 (82.7 to 116.6)	98.9 (83.0 to 115.1)
Non venomous animal contact	1 588 (1 292 to 1 972)	2 431 (2 047 to 2 911)	202.2 (167.8 to 244.4)	185.9 (156.6 to 222.3)	1 956 (1 617 to 2 354)	3 647 (3 077 to 4 306)	331.1 (278.8 to 389.5)	288.2 (244.4 to 338.2)
Foreign body	2 866 (1 543 to 4 625)	5 633 (2 604 to 9 855)	456.6 (212.3 to 771.4)	442.5 (197.7 to 765.1)	1 239 (939 to 1 651)	1 937 (1 391 to 2 762)	203.9 (157.0 to 272.9)	155.4 (111.8 to 218.0)
Pulmonary aspiration and foreign body in airway	85 (60 to 118)	54 (40 to 74)	8.6 (6.3 to 11.4)	4.1 (3.1 to 5.6)	55 (40 to 76)	45 (37 to 55)	7.8 (5.8 to 10.3)	3.5 (2.9 to 4.2)
Foreign body in eyes	2 563 (1 232 to 4 337)	5 382 (2 361 to 9 601)	423.9 (176.3 to 739.2)	423.2 (179.3 to 746.0)	519 (267 to 898)	1 178 (646 to 1 972)	97.1 (54.7 to 159.9)	96.5 (54.8 to 157.2)
Foreign body in other body part	218 (146 to 334)	198 (139 to 263)	24.1 (16.9 to 34.6)	15.2 (10.6 to 20.0)	658 (521 to 845)	719 (591 to 897)	99.1 (80.3 to 124.4)	55.5 (45.7 to 68.9)
Environmental heat and cold exposure	385 (274 to 528)	254 (182 to 357)	50.7 (38.1 to 64.2)	20.0 (14.5 to 28.2)	1 308 (1 062 to 1 589)	996 (830 to 1 200)	214.6 (179.5 to 255.4)	78.7 (65.7 to 94.9)
Exposure to forces of nature	63 (52 to 79)	15 (12 to 18)	7.2 (6.1 to 8.7)	1.1 (0.9 to 1.3)	275 (196 to 382)	572 (399 to 797)	38.3 (27.5 to 53.4)	43.6 (30.5 to 60.7)
Other unintentional injuries	1 418 (1 348 to 1 751)	1 579 (1 325 to 1 876)	189.0 (158.0 to 225.9)	118.6 (99.4 to 140.5)	1 982 (1 702 to 2 296)	2 696 (2 338 to 3 080)	344.3 (297.7 to 395.2)	210.2 (183.1 to 239.6)

Cause	Years lived with disability: All-age counts (95% UI) 1990	Years lived with disability: All-age counts (95% UI) 2017	Years lived with disability: Age-standardised rates per 100,000 (95% UI) 1990	Years lived with disability: Age-standardised rates per 100,000 (95% UI) 2017	Disability adjusted life years: All-age counts (95% UI) 1990	Disability adjusted life years: All-age counts (95% UI) 2017	Disability adjusted life years: Age-standardised rates per 100,000 (95% UI) 1990	Disability adjusted life years: Age-standardised rates per 100,000 (95% UI) 2017
**Transport and unintentional injuries**	**2 452 (1 785 to 3 264)**	**5 015 (3 631 to 6 818)**	**423.5 (307.3 to 559.8)**	**396.7 (287.0 to 536.3)**	**25 658 (24 230 to 27 196)**	**26 795 (24 520 to 29 091)**	**3 308.3 (3 137.7 to 3 482.2)**	**1 991.9 (1 822.4 to 2 164.6)**
**Transport injuries**	**449 (330 to 587)**	**1 266 (913 to 1 692)**	**79.1 (58.4 to 104.0)**	**98.1 (70.9 to 131.1)**	**11 842 (11 217 to 12 510)**	**13 680 (12 502 to 14 845)**	**1 522.7 (1 450.4 to 1 601.8)**	**989.4 (905.0 to 1 072.7)**
Road injuries	433 (309 to 541)	1 231 (889 to 1 647)	73.5 (54.0 to 95.7)	95.3 (68.7 to 127.7)	11 521 (10 892 to 12 163)	13 176 (12 043 to 14 309)	1 479.1 (1 410.8 to 1 555.9)	953.0 (873.8 to 1 033.8)
Pedestrian road injuries	111 (78 to 149)	268 (188 to 362)	20.0 (14.2 to 26.6)	21.0 (14.8 to 28.3)	5 632 (5 196 to 6 052)	4 987 (4 518 to 5 476)	726.2 (679.6 to 773.4)	367.0 (332.9 to 402.8)
Cyclist road injuries	48 (34 to 63)	147 (103 to 195)	8.1 (5.7 to 10.6)	11.2 (7.9 to 15.0)	317 (284 to 351)	591 (519 to 669)	43.1 (38.8 to 47.4)	43.5 (38.3 to 48.8)
Motorcyclist road injuries	79 (55 to 106)	154 (105 to 230)	13.8 (9.6 to 18.8)	11.8 (8.0 to 16.3)	1 615 (1 459 to 1 798)	1 791 (1 560 to 2 029)	204.5 (185.7 to 224.4)	126.1 (110.3 to 142.8)
Motor vehicle road injuries	130 (94 to 172)	575 (412 to 768)	23.9 (17.3 to 31.5)	44.4 (31.9 to 59.3)	3 585 (3 290 to 3 905)	5 407 (4 809 to 5 994)	459.9 (426.9 to 493.2)	387.0 (344.5 to 427.6)
Other road injuries	45 (32 to 61)	88 (63 to 122)	7.8 (5.7 to 10.5)	6.9 (4.9 to 9.5)	352 (301 to 403)	400 (349 to 457)	45.4 (39.2 to 51.3)	29.3 (25.6 to 33.3)
Other transport injuries	37 (26 to 49)	35 (25 to 48)	5.6 (4.0 to 7.4)	2.8 (2.0 to 3.7)	341 (308 to 379)	504 (442 to 568)	43.6 (39.7 to 47.8)	36.4 (32.1 to 41.0)
**Unintentional injuries**	**2 001 (1 441 to 2 686)**	**3 749 (2 693 to 5 125)**	**344.4 (248.7 to 462.6)**	**298.6 (214.5 to 408.4)**	**13 816 (12 826 to 14 899)**	**13 116 (11 775 to 14 571)**	**1 785.7 (1 667.6 to 1 919.5)**	**1 002.5 (901.3 to 1 115.0)**
Falls	1 210 (887 to 1 582)	2 643 (1 910 to 3 497)	216.6 (156.8 to 282.2)	213.4 (153.8 to 284.0)	3 139 (2 791 to 3 524)	4 772 (4 015 to 5 672)	526.4 (466.3 to 594.7)	383.2 (322.7 to 455.3)
Drowning	9 (6 to 12)	9 (6 to 13)	1.6 (1.1 to 2.2)	0.7 (0.5 to 1.0)	2 457 (2 235 to 2 702)	1 506 (1 341 to 1 693)	277.1 (255.6 to 299.9)	109.0 (96.9 to 122.3)
Fire, heat, and hot substances	205 (130 to 321)	256 (150 to 417)	30.6 (19.3 to 47.9)	19.4 (11.4 to 31.5)	1 153 (1 019 to 1 329)	750 (633 to 908)	142.6 (127.4 to 163.0)	57.1 (48.3 to 69.2)
Poisonings	33 (20 to 50)	59 (35 to 89)	4.4 (2.6 to 6.5)	4.3 (2.6 to 6.5)	769 (692 to 851)	502 (450 to 555)	85.7 (78.6 to 93.0)	36.7 (33.0 to 40.6)
Poisoning by carbon monoxide	4 (3 to 7)	5 (3 to 7)	0.6 (0.4 to 0.9)	0.3 (0.2 to 0.5)	340 (303 to 378)	241 (214 to 268)	38.8 (35.1 to 42.4)	17.5 (15.6 to 19.5)
Poisoning by other means	29 (17 to 44)	54 (32 to 82)	3.8 (2.3 to 5.7)	4.0 (2.4 to 6.0)	429 (380 to 482)	261 (229 to 298)	46.9 (42.2 to 51.8)	19.2 (16.8 to 21.9)
Exposure to mechanical forces	249 (172 to 355)	394 (267 to 576)	43.6 (30.0 to 62.3)	30.5 (20.6 to 44.6)	1 628 (1 501 to 1 785)	1 254 (1 098 to 1 455)	221.9 (203.6 to 244.5)	92.9 (81.2 to 108.2)
Unintentional firearm injuries	27 (18 to 41)	29 (18 to 45)	4.8 (3.1 to 7.4)	2.2 (1.4 to 3.5)	599 (532 to 681)	312 (250 to 364)	77.0 (69.0 to 87.8)	22.4 (18.0 to 26.0)
Other exposure to mechanical forces	221 (152 to 317)	365 (246 to 536)	38.8 (26.6 to 55.7)	28.3 (19.0 to 41.5)	1 029 (916 to 1 153)	942 (806 to 1 120)	144.9 (128.5 to 163.7)	70.5 (60.3 to 84.4)
Adverse effects of medical treatment	6 (4 to 9)	15 (9 to 23)	1.0 (0.6 to 1.4)	1.2 (0.7 to 1.8)	606 (480 to 684)	626 (550 to 808)	76.7 (63.0 to 84.5)	48.9 (43.1 to 62.5)
Animal contact	69 (46 to 98)	111 (74 to 159)	10.4 (7.0 to 14.8)	8.5 (5.7 to 12.2)	347 (298 to 402)	213 (179 to 265)	38.8 (33.7 to 45.1)	16.3 (13.2 to 20.2)
Venomous animal contact	39 (25 to 56)	60 (39 to 85)	5.3 (3.5 to 7.6)	4.5 (2.9 to 6.3)	200 (163 to 243)	94 (72 to 124)	20.3 (16.8 to 24.5)	7.1 (5.5 to 9.4)
Non venomous animal contact	30 (18 to 48)	51 (30 to 84)	5.0 (3.1 to 8.0)	4.1 (2.4 to 6.6)	147 (110 to 175)	118 (92 to 152)	18.5 (13.8 to 22.4)	9.1 (7.1 to 11.8)
Foreign body	78 (51 to 116)	114 (69 to 183)	12.7 (8.3 to 19.0)	9.2 (5.6 to 14.8)	2 646 (2 302 to 3 024)	2 655 (2 259 to 3 156)	275.6 (246.0 to 306.6)	197.5 (168.2 to 234.5)
Pulmonary aspiration and foreign body in airway	3 (2 to 4)	2 (1 to 3)	0.4 (0.3 to 0.5)	0.1 (0.1 to 0.2)	2 477 (2 138 to 2 841)	2 490 (2 110 to 2 969)	252.9 (224.3 to 283.1)	184.7 (156.5 to 220.4)
Foreign body in eyes	34 (18 to 63)	78 (41 to 140)	6.5 (3.5 to 11.6)	6.4 (3.4 to 11.4)	34 (18 to 63)	78 (41 to 140)	6.5 (3.5 to 11.6)	6.4 (3.4 to 11.4)
Foreign body in other body part	41 (28 to 55)	34 (24 to 46)	5.8 (4.1 to 7.7)	2.6 (1.9 to 3.5)	134 (114 to 158)	87 (71 to 132)	16.2 (13.8 to 18.9)	6.4 (5.2 to 9.6)
Environmental heat and cold exposure	52 (34 to 78)	38 (25 to 54)	8.4 (5.6 to 11.8)	3.0 (2.0 to 4.3)	262 (234 to 292)	142 (125 to 163)	37.4 (33.8 to 41.6)	10.9 (9.5 to 12.5)
Exposure to forces of nature	13 (10 to 19)	19 (14 to 26)	1.9 (1.3 to 2.6)	1.4 (1.0 to 2.0)	38 (33 to 44)	30 (25 to 37)	4.7 (4.1 to 5.5)	2.2 (1.8 to 2.8)
Other unintentional injuries	78 (57 to 104)	91 (64 to 125)	13.3 (9.7 to 17.8)	7.1 (5.0 to 9.7)	777 (709 to 848)	665 (593 to 742)	98.8 (90.6 to 107.5)	47.8 (42.7 to 53.5)
