# Supplementary material for: Unintentional injuries in Mexico, 1990–2017: findings from the Global Burden of Disease Study 2017
Source: Inj Prev. 2020 Apr 1;26(Suppl 1):i154–61. doi: 10.1136/injuryprev-2019-043532 (PMC7571365; doi:10.1136/injuryprev-2019-043532)
Supplement: Supplementary data [file injuryprev-2019-043532supp033.pdf]

Death, YLL, incidence, prevalence, YLD, and DALY counts and rates per 100,000 for all injuries in Yucatán in 1990 and 2017

Cause	Deaths				Years of life lost			
	All-age counts (95% UI)		Age-standardised rates per 100,000 (95% UI)		All-age counts (95% UI)		Age-standardised rates per 100,000 (95% UI)	
	1990	2017	1990	2017	1990	2017	1990	2017
**Transport and unintentional injuries**	**646 (621 to 672)**	**730 (678 to 793)**	**58.4 (56.6 to 60.3)**	**34.2 (31.8 to 37.0)**	**32 011 (30 160 to 33 948)**	**30 114 (27 843 to 32 767)**	**2 308.6 (2 203.6 to 2 422.8)**	**1 340.8 (1 242.0 to 1 459.6)**
**Transport injuries**	**314 (299 to 329)**	**388 (353 to 429)**	**26.2 (25.0 to 27.3)**	**17.2 (15.7 to 19.0)**	**16 296 (15 378 to 17 317)**	**17 834 (16 189 to 19 790)**	**1 180.0 (1 121.1 to 1 245.6)**	**773.5 (702.7 to 855.6)**
Road injuries	298 (284 to 314)	360 (327 to 399)	24.8 (23.8 to 26.0)	16.0 (14.5 to 17.7)	15 511 (14 636 to 16 492)	16 591 (15 067 to 18 427)	1 123.4 (1 067.2 to 1 185.3)	719.6 (654.2 to 797.7)
Pedestrian road injuries	146 (137 to 155)	156 (142 to 174)	13.1 (12.4 to 13.8)	7.1 (6.5 to 7.9)	6 945 (6 406 to 7 599)	6 411 (5 779 to 7 159)	535.5 (481.2 to 552.7)	282.7 (255.4 to 314.0)
Cyclist road injuries	16 (15 to 18)	34 (30 to 39)	1.3 (1.2 to 1.4)	1.5 (1.3 to 1.7)	853 (760 to 951)	1 452 (1 267 to 1 674)	62.5 (56.3 to 69.4)	63.4 (55.4 to 72.9)
Motorcyclist road injuries	46 (42 to 51)	63 (55 to 72)	3.5 (3.2 to 3.8)	2.7 (2.3 to 3.1)	2 667 (2 400 to 2 971)	3 445 (3 003 to 3 976)	187.4 (170.3 to 207.0)	145.9 (127.3 to 168.0)
Motor vehicle road injuries	85 (78 to 92)	100 (88 to 112)	6.5 (6.0 to 7.1)	4.4 (3.8 to 4.9)	4 735 (4 345 to 5 188)	4 979 (4 345 to 5 605)	335.9 (309.6 to 367.4)	214.1 (187.4 to 241.3)
Other road injuries	6 (5 to 7)	7 (6 to 8)	0.5 (0.4 to 0.6)	0.3 (0.3 to 0.3)	312 (262 to 362)	310 (265 to 358)	22.1 (18.7 to 25.3)	13.5 (11.5 to 15.9)
Other transport injuries	15 (14 to 17)	27 (23 to 31)	1.3 (1.2 to 1.4)	1.2 (1.1 to 1.4)	785 (704 to 878)	1 242 (1 052 to 1 430)	56.6 (51.4 to 62.8)	53.9 (46.0 to 61.8)
**Unintentional injuries**	**332 (316 to 348)**	**342 (322 to 366)**	**32.3 (31.0 to 33.4)**	**17.0 (16.0 to 18.1)**	**15 715 (14 565 to 16 960)**	**12 280 (11 413 to 13 308)**	**1 128.6 (1 064.2 to 1 197.1)**	**567.3 (521.9 to 616.1)**
Falls	87 (83 to 92)	118 (108 to 127)	11.2 (10.6 to 11.8)	6.2 (5.7 to 6.7)	2 530 (2 354 to 2 673)	2 781 (2 504 to 3 035)	228.3 (216.8 to 240.6)	129.5 (117.3 to 141.2)
Drowning	57 (53 to 62)	41 (37 to 46)	4.3 (4.0 to 4.6)	1.9 (1.7 to 2.1)	3 296 (2 991 to 3 628)	2 001 (1 791 to 2 242)	219.8 (201.9 to 240.1)	89.5 (80.3 to 100.4)
Fire, heat, and hot substances	25 (24 to 27)	22 (20 to 23)	2.6 (2.5 to 2.7)	1.1 (1.0 to 1.2)	1 160 (1 087 to 1 311)	667 (605 to 731)	82.8 (75.7 to 91.5)	31.1 (28.2 to 34.0)
Poisonings	11 (10 to 12)	9 (8 to 10)	0.9 (0.8 to 0.9)	0.4 (0.4 to 0.5)	661 (593 to 745)	419 (375 to 466)	43.5 (39.6 to 48.2)	18.9 (16.9 to 21.0)
Poisoning by carbon monoxide	3 (2 to 3)	3 (2 to 3)	0.2 (0.2 to 0.2)	0.1 (0.1 to 0.1)	151 (134 to 172)	119 (103 to 136)	10.3 (9.1 to 11.5)	5.3 (4.6 to 6.1)
Poisoning by other means	9 (8 to 9)	7 (6 to 7)	0.7 (0.6 to 0.7)	0.3 (0.3 to 0.3)	510 (454 to 578)	300 (268 to 335)	33.3 (30.1 to 37.1)	13.6 (12.1 to 15.2)
Exposure to mechanical forces	33 (31 to 35)	26 (24 to 29)	2.8 (2.7 to 3.0)	1.2 (1.1 to 1.3)	1 679 (1 547 to 1 825)	1 122 (1 007 to 1 254)	123.1 (114.6 to 132.8)	49.0 (44.2 to 54.7)
Unintentional firearm injuries	12 (10 to 13)	7 (5 to 8)	0.9 (0.8 to 1.0)	0.3 (0.2 to 0.4)	630 (549 to 705)	331 (270 to 394)	45.1 (39.3 to 50.4)	14.2 (11.5 to 16.8)
Other exposure to mechanical forces	21 (20 to 23)	19 (17 to 22)	1.9 (1.8 to 2.0)	0.9 (0.8 to 1.0)	1 049 (946 to 1 160)	791 (701 to 906)	78.0 (71.3 to 85.2)	34.8 (31.1 to 39.8)
Adverse effects of medical treatment	28 (22 to 30)	38 (33 to 43)	3.1 (2.4 to 3.3)	2.0 (1.7 to 2.2)	1 118 (907 to 1 286)	1 096 (964 to 1 291)	84.0 (68.2 to 93.7)	55.5 (46.0 to 62.0)
Animal contact	8 (7 to 9)	5 (5 to 6)	0.7 (0.6 to 0.7)	0.3 (0.2 to 0.3)	483 (404 to 574)	204 (182 to 227)	30.8 (26.5 to 35.7)	9.6 (8.6 to 10.8)
Venomous animal contact	6 (6 to 7)	4 (3 to 4)	0.5 (0.4 to 0.6)	0.2 (0.2 to 0.2)	397 (321 to 481)	142 (117 to 164)	24.7 (20.6 to 29.1)	6.8 (5.6 to 7.9)
Non-venomous animal contact	2 (1 to 2)	2 (1 to 2)	0.2 (0.1 to 0.2)	0.1 (0.1 to 0.1)	87 (69 to 104)	62 (51 to 83)	6.1 (4.8 to 7.2)	2.8 (2.4 to 3.3)
Foreign body	51 (46 to 57)	56 (51 to 62)	4.0 (3.7 to 4.3)	2.7 (2.5 to 3.0)	3 186 (2 747 to 3 726)	2 769 (2 422 to 3 216)	199.2 (175.3 to 228.2)	134.0 (115.7 to 157.9)
Pulmonary aspiration and foreign body in airway	49 (44 to 55)	55 (50 to 61)	3.8 (3.6 to 4.2)	2.7 (2.4 to 3.0)	3 078 (2 647 to 3 610)	2 712 (2 372 to 3 159)	192.2 (168.8 to 219.9)	131.5 (113.7 to 155.3)
Foreign body in eyes								
Foreign body in other body part	2 (1 to 2)	1 (1 to 2)	0.1 (0.1 to 0.2)	0.0 (0.0 to 0.1)	107 (90 to 137)	57 (43 to 125)	7.0 (6.0 to 8.9)	2.5 (1.9 to 5.5)
Environmental heat and cold exposure	9 (9 to 10)	6 (6 to 7)	1.0 (1.0 to 1.1)	0.3 (0.3 to 0.3)	387 (354 to 426)	222 (194 to 247)	31.0 (28.7 to 33.5)	9.9 (8.7 to 11.1)
Exposure to forces of nature	1 (1 to 1)	0 (0 to 0)	0.0 (0.0 to 0.1)	0.0 (0.0 to 0.0)	40 (36 to 44)	18 (17 to 20)	2.6 (2.4 to 2.8)	0.8 (0.8 to 0.9)
Other unintentional injuries	22 (20 to 24)	20 (18 to 23)	1.7 (1.6 to 1.9)	0.9 (0.8 to 1.0)	1 196 (1 090 to 1 313)	982 (857 to 1 129)	83.5 (76.5 to 91.3)	42.4 (37.1 to 48.5)

Cause	Incidence				Prevalence			
	All-age counts (95% UI)		Age-standardised rates per 100,000 (95% UI)		All-age counts (95% UI)		Age-standardised rates per 100,000 (95% UI)	
	1990	2017	1990	2017	1990	2017	1990	2017
**Transport and unintentional injuries**	**43 335 (40 073 to 46 741)**	**69 144 (62 836 to 76 141)**	**3 424.2 (3 177.1 to 3 685.9)**	**3 211.4 (2 916.9 to 3 542.4)**	**88 610 (83 089 to 94 834)**	**155 061 (145 480 to 167 359)**	**8 060.1 (7 589.8 to 8 637.0)**	**7 070.1 (6 631.8 to 7 620.7)**
**Transport injuries**	**4 202 (3 626 to 4 870)**	**9 454 (8 178 to 10 895)**	**325.1 (282.8 to 370.5)**	**417.2 (362.0 to 480.9)**	**11 027 (10 302 to 11 790)**	**27 929 (25 932 to 29 844)**	**1 031.6 (965.5 to 1 100.4)**	**1 251.3 (1 161.9 to 1 337.4)**
Road injuries	3 858 (3 280 to 4 512)	9 030 (7 734 to 10 451)	300.2 (259.3 to 344.9)	397.5 (340.4 to 459.6)	9 453 (8 839 to 10 112)	25 619 (23 772 to 27 495)	893.9 (835.3 to 958.6)	1 146.7 (1 065.4 to 1 229.8)
Pedestrian road injuries	604 (485 to 738)	982 (771 to 1 226)	49.5 (40.3 to 59.8)	43.8 (34.6 to 54.5)	2 308 (1 998 to 2 692)	4 470 (3 883 to 5 218)	223.2 (195.0 to 259.5)	202.5 (176.2 to 235.7)
Cyclist road injuries	889 (702 to 1 129)	1 845 (1 467 to 2 280)	65.8 (52.8 to 81.6)	82.9 (65.9 to 102.5)	1 608 (1 358 to 1 882)	4 087 (3 471 to 4 786)	146.7 (124.9 to 169.6)	183.4 (156.3 to 212.1)
Motorcyclist road injuries	528 (417 to 671)	1 280 (988 to 1 621)	39.7 (31.9 to 49.4)	55.4 (42.9 to 69.8)	1 995 (1 731 to 2 308)	5 769 (4 923 to 6 753)	186.1 (161.3 to 215.1)	254.8 (217.9 to 297.7)
Motor vehicle road injuries	1 176 (945 to 1 426)	3 727 (3 028 to 4 502)	93.4 (75.1 to 113.1)	161.7 (131.9 to 194.0)	2 221 (1 937 to 2 589)	8 392 (7 267 to 9 854)	215.2 (186.8 to 250.7)	375.1 (324.9 to 440.2)
Other road injuries	671 (487 to 865)	1 195 (851 to 1 682)	51.8 (39.0 to 65.0)	53.7 (38.2 to 74.7)	1 321 (1 099 to 1 583)	2 902 (2 426 to 3 506)	122.6 (102.2 to 146.6)	130.3 (109.3 to 157.8)
Other transport injuries	333 (255 to 445)	424 (317 to 577)	24.8 (20.1 to 31.0)	19.7 (14.6 to 27.0)	1 574 (1 303 to 1 857)	2 310 (1 960 to 2 668)	137.7 (115.9 to 159.4)	104.6 (89.3 to 120.5)
**Unintentional injuries**	**39 133 (36 018 to 42 468)**	**59 690 (53 654 to 66 513)**	**3 099.1 (2 860.3 to 3 358.5)**	**2 794.2 (2 518.1 to 3 106.5)**	**77 583 (72 304 to 83 767)**	**127 132 (117 492 to 139 038)**	**7 028.4 (6 561.6 to 7 571.0)**	**5 818.8 (5 376.5 to 6 350.0)**
Falls	17 853 (15 563 to 20 542)	33 584 (28 513 to 39 664)	1 531.0 (1 354.5 to 1 733.1)	1 603.4 (1 362.6 to 1 907.8)	32 041 (28 369 to 36 859)	67 534 (60 169 to 77 340)	3 054.8 (2 738.3 to 3 513.9)	3 146.2 (2 808.7 to 3 597.1)
Drowning	54 (47 to 62)	45 (35 to 58)	4.0 (3.5 to 4.6)	2.0 (1.6 to 2.6)	265 (234 to 304)	286 (248 to 335)	25.1 (22.3 to 28.6)	13.0 (11.3 to 15.2)
Fire, heat, and hot substances	1 563 (1 231 to 1 968)	1 565 (1 264 to 1 934)	97.7 (78.7 to 120.4)	74.9 (60.4 to 93.4)	15 828 (13 114 to 20 009)	19 594 (16 372 to 24 711)	1 300.4 (1 089.3 to 1 604.8)	876.0 (733.0 to 1 104.4)
Poisonings	480 (347 to 669)	567 (411 to 760)	27.8 (20.7 to 37.9)	27.9 (20.0 to 37.6)	485 (328 to 541)	732 (592 to 889)	32.5 (24.6 to 40.2)	31.7 (23.7 to 39.6)
Poisoning by carbon monoxide	96 (57 to 158)	112 (58 to 185)	6.0 (3.8 to 9.3)	5.4 (2.8 to 8.9)	69 (53 to 90)	95 (73 to 122)	5.3 (4.1 to 6.8)	4.2 (3.3 to 5.4)
Poisoning by other means	384 (273 to 554)	454 (314 to 646)	21.8 (15.6 to 30.8)	22.6 (15.5 to 32.2)	366 (261 to 468)	637 (442 to 789)	27.2 (19.5 to 34.8)	27.5 (19.7 to 35.3)
Exposure to mechanical forces	7 958 (6 624 to 9 516)	9 258 (7 287 to 11 701)	578.9 (489.7 to 688.4)	412.7 (325.7 to 520.3)	16 589 (14 582 to 19 161)	23 772 (20 645 to 27 544)	1 520.1 (1 333.3 to 1 743.4)	1 062.1 (922.4 to 1 227.7)
Unintentional firearm injuries	466 (323 to 646)	429 (274 to 634)	33.0 (23.6 to 44.5)	18.9 (12.3 to 28.0)	1 921 (1 535 to 2 270)	2 200 (1 783 to 2 629)	177.6 (143.3 to 210.5)	98.8 (80.4 to 117.7)
Other exposure to mechanical forces	7 492 (6 255 to 9 019)	8 830 (6 936 to 11 162)	545.8 (462.2 to 651.1)	393.8 (310.3 to 499.5)	14 668 (12 452 to 17 249)	21 571 (18 332 to 25 429)	1 342.5 (1 152.0 to 1 574.3)	963.3 (820.6 to 1 131.5)
Adverse effects of medical treatment	1 134 (954 to 1 309)	2 205 (1 883 to 2 548)	96.5 (82.7 to 110.3)	100.7 (86.2 to 116.1)	87 (68 to 107)	169 (130 to 210)	7.4 (5.7 to 9.1)	7.7 (5.9 to 9.6)
Animal contact	3 451 (2 840 to 4 231)	4 015 (3 496 to 4 641)	253.2 (213.5 to 301.7)	186.2 (162.2 to 215.7)	3 775 (3 247 to 4 362)	5 195 (4 530 to 5 927)	333.8 (291.7 to 378.7)	235.3 (205.2 to 267.4)
Venomous animal contact	1 216 (914 to 1 607)	1 420 (1 132 to 1 757)	89.3 (68.7 to 113.7)	65.6 (52.2 to 81.6)	811 (670 to 959)	1 076 (899 to 1 251)	68.2 (57.2 to 79.3)	48.4 (40.6 to 56.3)
Non-venomous animal contact	2 235 (1 781 to 2 800)	2 596 (2 190 to 3 135)	163.9 (133.4 to 200.3)	120.6 (101.5 to 145.5)	2 964 (2 455 to 3 536)	4 120 (3 493 to 4 841)	265.5 (222.3 to 312.9)	186.7 (158.7 to 218.2)
Foreign body	3 150 (1 966 to 4 391)	5 264 (2 991 to 7 957)	245.6 (148.5 to 355.5)	239.9 (138.0 to 357.5)	1 774 (1 427 to 2 247)	2 489 (1 962 to 3 208)	148.9 (121.8 to 184.4)	113.6 (89.4 to 145.7)
Pulmonary aspiration and foreign body in airway	100 (83 to 125)	88 (69 to 119)	7.9 (5.9 to 10.5)	4.2 (3.0 to 5.7)	94 (67 to 127)	83 (67 to 100)	7.2 (5.3 to 9.5)	3.7 (3.0 to 4.5)
Foreign body in eyes	2 671 (1 527 to 3 911)	4 827 (2 550 to 7 535)	214.6 (118.2 to 323.0)	219.0 (116.4 to 337.0)	585 (291 to 877)	1 055 (507 to 1 666)	48.3 (28.4 to 75.1)	49.0 (28.4 to 76.2)
Foreign body in other body part	349 (240 to 515)	350 (262 to 445)	23.0 (16.4 to 32.1)	16.7 (12.5 to 21.3)	1 144 (916 to 1 458)	1 359 (1 116 to 1 685)	93.4 (76.1 to 117.0)	61.0 (50.4 to 75.8)
Environmental heat and cold exposure	690 (486 to 900)	408 (303 to 549)	52.9 (40.4 to 65.2)	19.7 (14.7 to 26.8)	2 475 (2 033 to 2 994)	1 672 (1 387 to 2 044)	217.1 (182.3 to 257.1)	76.7 (63.9 to 93.3)
Exposure to forces of nature	103 (85 to 129)	24 (20 to 29)	6.6 (5.5 to 8.1)	1.1 (0.9 to 1.3)	270 (195 to 364)	830 (580 to 1 153)	19.8 (14.5 to 26.7)	35.9 (25.2 to 50.0)
Other unintentional injuries	2 696 (2 192 to 3 276)	2 755 (2 125 to 3 702)	204.9 (169.8 to 243.9)	125.6 (97.3 to 168.2)	4 045 (3 469 to 4 645)	4 879 (4 212 to 5 602)	368.6 (318.3 to 420.5)	220.6 (190.6 to 252.2)

Cause	Years lived with disability				Disability adjusted life years			
	All-age counts (95% UI)		Age-standardised rates per 100,000 (95% UI)		All-age counts (95% UI)		Age-standardised rates per 100,000 (95% UI)	
	1990	2017	1990	2017	1990	2017	1990	2017
**Transport and unintentional injuries**	**3 937 (2 883 to 5 247)**	**6 689 (4 804 to 8 953)**	**354.9 (259.6 to 473.9)**	**306.1 (219.2 to 407.4)**	**35 947 (33 851 to 38 267)**	**36 803 (33 574 to 40 177)**	**2 663.4 (2 518.5 to 2 818.3)**	**1 646.9 (1 502.6 to 1 795.6)**
**Transport injuries**	**748 (546 to 992)**	**1 694 (1 221 to 2 263)**	**68.1 (49.8 to 90.3)**	**75.3 (54.4 to 100.5)**	**17 043 (16 056 to 18 077)**	**19 527 (17 693 to 21 491)**	**1 248.1 (1 183.0 to 1 316.3)**	**848.8 (770.6 to 932.3)**
Road injuries	647 (475 to 861)	1 570 (1 135 to 2 093)	59.6 (43.6 to 79.3)	69.7 (50.4 to 93.0)	16 158 (15 246 to 17 151)	18 161 (16 407 to 19 959)	1 182.9 (1 122.0 to 1 248.9)	789.3 (714.7 to 866.7)
Pedestrian road injuries	164 (118 to 223)	286 (201 to 392)	15.4 (11.0 to 20.8)	12.8 (9.0 to 17.6)	7 109 (6 568 to 7 697)	6 697 (6 028 to 7 467)	550.8 (495.9 to 568.7)	295.5 (266.7 to 328.4)
Cyclist road injuries	111 (79 to 147)	247 (173 to 332)	9.9 (7.1 to 13.0)	11.0 (7.7 to 14.8)	964 (859 to 1 066)	1 699 (1 489 to 1 936)	72.5 (65.2 to 79.4)	74.4 (65.4 to 84.6)
Motorcyclist road injuries	139 (97 to 189)	356 (243 to 486)	12.6 (8.8 to 17.1)	15.6 (10.7 to 21.3)	2 805 (2 545 to 3 111)	3 801 (3 332 to 4 329)	200.0 (182.8 to 220.1)	161.6 (141.8 to 183.8)
Motor vehicle road injuries	158 (113 to 209)	539 (382 to 714)	14.8 (10.7 to 19.6)	23.6 (16.9 to 31.7)	4 892 (4 505 to 5 353)	5 505 (4 870 to 6 145)	350.7 (324.5 to 381.5)	237.7 (210.5 to 264.4)
Other road injuries	76 (54 to 103)	148 (104 to 203)	6.8 (5.0 to 9.3)	6.6 (4.7 to 9.1)	388 (330 to 444)	458 (392 to 541)	28.9 (24.9 to 33.0)	20.1 (17.2 to 23.8)
Other transport injuries	101 (72 to 135)	124 (87 to 167)	8.5 (6.2 to 11.2)	5.6 (3.9 to 7.5)	885 (795 to 982)	1 366 (1 179 to 1 558)	65.1 (58.8 to 71.6)	59.5 (51.5 to 67.5)
**Unintentional injuries**	**3 189 (2 331 to 4 298)**	**4 996 (3 545 to 6 756)**	**286.8 (208.1 to 384.3)**	**229.8 (163.3 to 311.2)**	**18 904 (17 491 to 20 570)**	**17 276 (15 449 to 19 287)**	**1 415.4 (1 314.7 to 1 531.0)**	**797.0 (713.6 to 888.3)**
Falls	1 901 (1 381 to 2 486)	3 469 (2 488 to 4 593)	177.3 (129.1 to 232.0)	161.3 (115.4 to 212.7)	4 411 (3 900 to 5 031)	6 250 (5 206 to 7 405)	405.6 (357.1 to 461.0)	290.8 (242.3 to 344.7)
Drowning	15 (10 to 20)	15 (10 to 21)	1.4 (1.0 to 1.9)	0.7 (0.5 to 1.0)	3 311 (3 008 to 3 648)	2 016 (1 808 to 2 256)	221.2 (203.7 to 241.4)	90.2 (81.0 to 100.9)
Fire, heat, and hot substances	340 (230 to 535)	381 (224 to 624)	27.8 (16.9 to 42.7)	17.0 (10.0 to 27.8)	1 501 (1 312 to 1 743)	1 048 (878 to 1 285)	110.1 (97.3 to 128.1)	48.1 (40.5 to 58.7)
Poisonings	51 (31 to 77)	82 (50 to 124)	3.7 (2.2 to 5.6)	3.6 (2.2 to 5.5)	712 (641 to 796)	501 (444 to 565)	47.3 (43.3 to 52.0)	22.6 (20.0 to 25.4)
Poisoning by carbon monoxide	7 (4 to 12)	10 (6 to 15)	0.5 (0.3 to 0.9)	0.4 (0.3 to 0.7)	159 (141 to 179)	129 (112 to 147)	10.8 (9.7 to 12.1)	5.7 (5.0 to 6.5)
Poisoning by other means	44 (26 to 67)	72 (43 to 111)	3.2 (1.9 to 4.9)	3.2 (1.9 to 5.0)	554 (494 to 622)	372 (325 to 426)	36.4 (33.1 to 40.3)	16.8 (14.7 to 19.3)
Exposure to mechanical forces	391 (267 to 556)	513 (348 to 744)	35.9 (24.2 to 50.2)	22.9 (15.5 to 33.2)	2 070 (1 884 to 2 287)	1 635 (1 421 to 1 898)	158.4 (144.3 to 175.1)	71.9 (62.5 to 83.9)
Unintentional firearm injuries	39 (25 to 59)	41 (26 to 64)	3.5 (2.3 to 5.3)	1.8 (1.2 to 2.9)	669 (583 to 745)	372 (256 to 435)	48.6 (42.2 to 54.0)	16.0 (13.1 to 18.6)
Other exposure to mechanical forces	352 (243 to 503)	472 (319 to 687)	31.8 (21.9 to 45.6)	21.1 (14.2 to 30.6)	1 401 (1 245 to 1 584)	1 263 (1 073 to 1 505)	109.8 (97.5 to 125.1)	55.9 (47.6 to 66.7)
Adverse effects of medical treatment	12 (7 to 17)	23 (14 to 34)	1.0 (0.6 to 1.5)	1.0 (0.6 to 1.5)	1 129 (918 to 1 301)	1 118 (987 to 1 311)	84.9 (69.4 to 94.8)	56.5 (47.2 to 62.9)
Animal contact	97 (65 to 137)	119 (78 to 171)	8.0 (5.3 to 11.1)	5.3 (3.5 to 7.7)	581 (494 to 673)	322 (273 to 383)	38.7 (33.6 to 44.3)	14.9 (12.7 to 17.7)
Venomous animal contact	51 (33 to 75)	60 (38 to 88)	3.9 (2.5 to 5.6)	2.7 (1.7 to 3.9)	448 (372 to 531)	202 (169 to 236)	28.6 (24.4 to 33.1)	9.5 (7.9 to 11.1)
Non-venomous animal contact	46 (28 to 73)	58 (35 to 95)	4.0 (2.5 to 6.4)	2.6 (1.6 to 4.3)	133 (107 to 166)	120 (94 to 159)	10.1 (8.3 to 12.9)	5.5 (4.3 to 7.2)
Foreign body	111 (76 to 156)	139 (91 to 205)	9.1 (6.2 to 12.7)	6.3 (4.2 to 9.3)	3 296 (2 860 to 3 828)	2 907 (2 557 to 3 352)	208.3 (184.7 to 236.0)	140.3 (122.2 to 164.4)
Pulmonary aspiration and foreign body in airway	5 (3 to 7)	3 (2 to 5)	0.4 (0.3 to 0.5)	0.2 (0.1 to 0.2)	3 083 (2 652 to 3 615)	2 716 (2 376 to 3 162)	192.6 (169.0 to 220.4)	131.6 (113.9 to 155.5)
Foreign body in eyes	36 (19 to 61)	71 (39 to 121)	3.3 (1.8 to 5.4)	3.3 (1.8 to 5.6)	36 (19 to 61)	71 (39 to 121)	3.3 (1.8 to 5.4)	3.3 (1.8 to 5.6)
Foreign body in other body part	70 (49 to 93)	64 (46 to 85)	5.5 (3.9 to 7.2)	2.9 (2.0 to 3.8)	177 (150 to 214)	121 (95 to 186)	12.5 (10.6 to 15.0)	5.4 (4.2 to 8.4)
Environmental heat and cold exposure	98 (64 to 138)	63 (41 to 91)	8.5 (5.7 to 12.0)	2.9 (1.9 to 4.2)	485 (436 to 542)	285 (249 to 325)	39.5 (35.8 to 43.9)	12.8 (11.2 to 14.7)
Exposure to forces of nature	14 (10 to 19)	27 (20 to 38)	1.0 (0.7 to 1.4)	1.2 (0.9 to 1.6)	54 (47 to 61)	45 (38 to 56)	3.6 (3.2 to 4.1)	2.0 (1.7 to 2.5)
Other unintentional injuries	159 (114 to 214)	166 (118 to 227)	14.2 (10.2 to 19.3)	7.5 (5.3 to 10.3)	1 355 (1 229 to 1 481)	1 148 (1 003 to 1 297)	97.8 (88.9 to 106.4)	49.9 (43.7 to 56.2)
